# The features of *Drosophila *core promoters revealed by statistical analysis

**DOI:** 10.1186/1471-2164-7-161

**Published:** 2006-06-21

**Authors:** Naum I Gershenzon, Edward N Trifonov, Ilya P Ioshikhes

**Affiliations:** 1 Department of Biomedical Informatics, The Ohio State University, 333 West 10^th ^Avenue, Columbus OH 43210, USA; 2 Department of Physics, Wright State University, Dayton OH 45435, USA; 3 Genome Diversity Center, Institute of Evolution, University of Haifa, Haifa 31905, Israel

## Abstract

**Background:**

Experimental investigation of transcription is still a very labor- and time-consuming process. Only a few transcription initiation scenarios have been studied in detail. The mechanism of interaction between basal machinery and promoter, in particular core promoter elements, is not known for the majority of identified promoters. In this study, we reveal various transcription initiation mechanisms by statistical analysis of 3393 nonredundant *Drosophila *promoters.

**Results:**

Using *Drosophila*-specific position-weight matrices, we identified promoters containing TATA box, Initiator, Downstream Promoter Element (DPE), and Motif Ten Element (MTE), as well as core elements discovered in Human (TFIIB Recognition Element (BRE) and Downstream Core Element (DCE)). Promoters utilizing known synergetic combinations of two core elements (TATA_Inr, Inr_MTE, Inr_DPE, and DPE_MTE) were identified. We also establish the existence of promoters with potentially novel synergetic combinations: TATA_DPE and TATA_MTE. Our analysis revealed several motifs with the features of promoter elements, including possible novel core promoter element(s). Comparison of Human and *Drosophila *showed consistent percentages of promoters with TATA, Inr, DPE, and synergetic combinations thereof, as well as most of the same functional and mutual positions of the core elements. No statistical evidence of MTE utilization in Human was found. Distinct nucleosome positioning in particular promoter classes was revealed.

**Conclusion:**

We present lists of promoters that potentially utilize the aforementioned elements/combinations. The number of these promoters is two orders of magnitude larger than the number of promoters in which transcription initiation was experimentally studied. The sequences are ready to be experimentally tested or used for further statistical analysis. The developed approach may be utilized for other species.

## Background

Research over the past thirty years has revealed the diversity of transcription initiation scenarios in eukaryotes. Only some of the scenarios have been studied in detail and more are likely to be discovered. So far, six core promoter elements have been experimentally identified in eukaryotes. These elements are TATA box, Initiator (Inr), Downstream Promoter Element (DPE), TFIIB recognition element (BRE), Downstream Core Element (DCE), and Motif Ten Element (MTE) [1-3].

The basal transcriptional machinery includes Pol II and general transcription factors (TF): TFIIA, B, D, E, F, and H [4-7]. TFIID plays the central role in transcription initiation [8,9], acting in cooperation with core promoter elements and/or specific TFs [6,7,10]. TFIID consists of the TATA Binding Protein (TBP) and TBP-associated factors (TAFs) [11]. The universal feature of transcription is binding of TBP to DNA at a specific distance from transcription start cite (TSS) regardless of the presence/absence of the TATA box. In the absence of the TATA box (TATA-less promoters), TAFs bind to DNA and/or to other TFs in order to involve TBP in pre-initiation complex [9,12-14]. From this perspective it is easy to comprehend why TATA box dominates as a core promoter element having the ability to govern transcription initiation alone (at least *in vitro*). The rest of the core elements usually work in cooperation with others. Indeed, strong synergism between DPE and Inr, MTE and Inr, DCE and Inr, MTE and DPE, BRE and TATA, and Inr and TATA has been experimentally established [9,14-19]. It is peculiar, that in spite of the considerable improvement of our knowledge of the transcriptional regulation processes due to emergence of new experimental techniques and computational approaches, the scenarios of the interaction between basal transcription machinery and the core promoter are not known for the majority of identified promoters [20].

The statistics of the core elements still remain obscure even for the most studied eukaryotes like *Drosophila*. So far, two *Drosophila *promoter databases have been analyzed. Kutach and Kadonaga [21] created a small *Drosophila *Core Promoter Database containing 205 sequences with an experimentally defined position of TSS "carefully extracted" from the literature. They visually identified the presence of TATA box, Inr, and DPE in those sequences and found that respectively 42.4%, 67.3% and 40.0% of the promoters contain TATA, Inr, and DPE at their functional positions. The larger database (1941 promoters) was constructed by Ohler *et al*. [22]. In total, 28.3% and 62.8% of promoters from this database have TATA and Inr elements, respectively [22]. These percentages have been found using motif consensuses for respective elements with one mismatch allowed.

The experimental investigation of the core promoter elements is still very labor- and time-consuming. Even for the well-studied elements, such as TATA box, Inr and DPE, only a few promoters have been experimentally examined. Therefore, the statistical analysis of large promoter databases is useful to complement experimental study by identifying new overrepresented motifs [22], revealing potential synergetic combinations [20], and classifying promoters.

The hypothesis behind our research is that in the course of evolution the motifs necessary for promoter regulation have been preserved in the promoter region, thus their occurrence frequencies there are far from random. We will examine the following particular questions:

1) How many known *Drosophila *promoters follow known scenarios of the interaction of the basal machinery and DNA? In particular, the transcription of how many promoters is guided by the TATA box and/or by any of the known synergetic combinations?

2) What are the typical distances between the core elements and TSS and between elements in synergetic combinations?

3) May statistical analysis suggest new synergetic combinations?

4) Are BRE and DCE (elements discovered in human promoters) statistically significant in *Drosophila *promoters?

5) What typical motifs in the core promoter sequences remain unknown?

6) How do *Drosophila *and human promoters differ statistically?

For statistical analysis we used an "Orthomine Database" of *Drosophila melanogaster *promoters [23] composed by P. Cherbas and S. Middha (pers. comm. prior to publication, see Data and Methods for description.)

## Results

Four core promoter elements (TATA box, Inr, DPE, MTE) have been experimentally identified in *Drosophila *promoters [1,2]. First, we considered statistical parameters of each of those elements: positional distribution, functional window, and percentage of promoters containing a particular element. We also examined the DCE and BRE elements in *Drosophila *promoters, although the biological function of those elements has only been observed in human promoters [3,14,17,19]. Second, we analyzed the parameters of synergetic and/or cooperative combinations of each pair of elements: typical distances between the elements and percentage of promoters containing a combination. Finally, we revealed typical motifs in different subsets of *Drosophila *promoters by the MEME program [24] and examined their positional distributions in promoter area.

### TATA box

First we examined the positional distributions of the TATA box sites by different consensus motifs (see Table 1 for consensuses). The distributions obtained by the consensuses with no mismatches, one, and even two mismatches exhibit huge over-representation (see Additional file 1, Supplemental Figures S2a-c) in the window from -33 to -23 bp relative to TSS (we defined the center of the TATA box at the position of the second 'T'). This is consistent with the experimental data [1]. So we consider the window (-33 - -23 bp) as a functional window for the TATA box element.

PWM for the TATA box was derived using a procedure described in the section **Data and Methods**. The PWM utilized only sites extracted from the functional window whose DNA sequences are equivalent to the consensus with one possible mismatch. The respective occurrence frequency table is shown in Supplemental Table S1 (see Additional file 1). See also pictogram at Table 2. Note that the length of PWM is greater than the length of the consensus. We included one extra position from the 5'-end and 3 extra positions from the 3'-end since they exhibit bias nucleotide composition. To define the optimal cutoff value we used a procedure described in detail in the section **Data and Methods**.

Using this new PWM for the TATA box (built specifically for *Drosophila*) we are able to find the number and percentage of TATA+ promoters as well as statistical significance (formula I from **Data and Methods**) of the TATA over-representation in the functional window (see Table 1, first line, columns 7–9). One can see that the percentage of TATA-containing promoters is much less than previous estimates; compare with 42.4% [21] and 28.3% [22]. However, this percentage is comparable with estimation for the human promoters [20]. Note, that if we apply our PWM to *Drosophila *Core Promoter Database at the region from -45 to -15 bp (as in [21]) we find that 40.0% of promoters have the TATA box, which is close to their estimate (42.4%). So the difference between percentages (42.4%, 28.3%, and 16.2%) can be explained by the differences between databases and applied intervals. The positional distribution of the TATA box obtained by PWM is shown at Supplemental Figure S2d (see Additional file 1). The set of promoter sequences potentially utilizing TATA box element is presented in Supplemental Sequences S1 (see Additional file 2).

### Initiator

The analogous analysis with Inr consensus allows building PWM for Inr (see pictogram at Table 2 and also Additional file 1, Supplemental Figures S3a and S3b and Table S2) as well as finding respective statistical parameters (Table 1, second line).

The percentage of promoters with Initiator (66.5%) is comparable with [21] (67.3%) and [22] (62.8%) estimates. Analysis of the Inr positional distribution for the considered database (see Additional file 1, Supplemental Figure S3c) shows significant over-representation for the Inr motif in the area (-1 to +9 bp). Although that differs from the canonical Inr positioning at +1 bp, we consider that window as functional for Inr. The difference may in part be a mere consequence of imprecise TSS mapping for some of the promoters, but may also have other, less trivial reasons (see below). Note, that this window is asymmetric relative to TSS, with Inr often shifted upstream from TSS. The promoter sequences with Inr may be found in Supplemental Sequences S2 (see Additional file 2).

### DPE

The DPE element was discovered and studied mainly in *Drosophila *[9,21]. The positional distribution of DPE (see Additional file 1, Supplemental Figure S4a) exhibits over-representation in the area from +27 to +33 bp with maximum at position +28 bp, which is the experimentally defined functional position for DPE. Note that sites resembling DPE are under-represented almost in the entire promoter area except of the functional window and around TSS. The latter is just an artifact since DPE and Inr motifs partially coincide (compare 'RGWY' in DPE and 'AKTY' in Inr). Since DPE works in cooperation with Inr at a strict distance, the functional window for DPE should have at least the same size as a functional window for Inr. That is why we consider the interval from +27 to +36 as a functional window for DPE despite over-representation of the DPE sites in narrower interval (27–33).

The selection of DPE motif consensus is not straightforward. The initial study based on three *Drosophila *and one human promoters [9] revealed sequence motif G(A/T)CG as a new core promoter element. Later on, the functional significance and universality of this motif were confirmed on 19 *Drosophila *promoters [21]. The experimentation *in vitro *with randomized sequences showed that variety of sequences could function as DPE [21]. Thus, the consensuses RGWYVT or/and RGWYV were suggested, although there is no evidence that all possible sequences from these motifs are indeed functional in real promoters *in vivo*. To choose the sufficient consensus we first applied the most trusted motif G(A/T)CG to the promoter database and extracted all promoters containing this motif in the window from +27 to +33 bp. Then we found the positional distribution of sites with consensus RGWYVT in the remaining (DPE-less) subset of promoters. The positional distribution showed over-representation of motif RGWYVT in the same window suggesting functional significance of this consensus. Then we applied consensus RGWYV to the subset of DPE-less (in this case RGWYVT-less) promoters and found that even this loosest motif is still over-represented in the functional window. Thus, statistics suggest that the consensus RGWYV is viable for DPE, so this information was used for further analyses and the PWM building. Supplemental Table S3 (see Additional file 1) and the pictogram at Table 2 present the frequency table calculated based on the DPE sites at positions from 27 to 29. The positional distribution of DPE obtained by PWM is in Supplemental Figure S4b (see Additional file 1).

The statistical parameters of DPE calculated based on the PWM are presented at Table 1, third line. One can see that the percentage of potential DPE promoters is even larger than percentage of the TATA box promoters. The set of promoter sequences most likely utilizing DPE element is presented in Supplemental Sequences S3 (see Additional file 2).

### MTE

The Motif Ten Element, "CSARCSSAACGS", initially was discovered by statistical analysis of *Drosophila *promoter database [22]. Then the functional significance of MTE as a new core promoter element has been experimentally established [2]. It was shown that the first five nucleotides are important for transcriptional activity, while the seven remaining nucleotides are "sufficient to confer MTE activity to heterologous core promoters" [2]. MTE (at position +18) works in cooperation with Inr and also with DPE. Since the synergetic position for the DPE is +28 the last two nucleotides are overlapped with DPE. Because it is not clear what the functional MTE motif consensus is, we considered three consensuses: first 5, first 10, and 12 bp long. All of them are essentially over-represented in the functional window. For further statistical analysis we used only the 10 bp long consensus (see Table 1, fourth line). Note that in contrast to the DPE, MTE is over-represented practically in whole promoter area (see Additional file 1, Supplemental Figures S5a and S5b). The PWM was obtained based on the frequency table (Table 2 and Additional file 1, Supplemental Table S4) built by sites extracted from positions +18 - +23 by consensus allowing up to two mismatches. The promoter sequences with MTE at its functional position are presented in Supplemental Sequences S4 (see Additional file 2).

Although it was shown that MTE is also functional (*in vitro*) in human promoters [2], the preliminary statistical analysis of two human promoter databases (Eukaryotic Promoter Database [25] and Database of Transcriptional Start Sites [26]) using any of three considered above consensuses did not show overrepresentation of MTE at expected functional positions in human promoters.

### BRE and DCE

We found that these two elements discovered in human promoters are statistically overrepresented in Drosophila promoters too. For the details of their statistical analysis as well as a list of potential promoters utilizing them as core promoter elements see Additional file 1.

### Potential synergetic combinations

The core promoter elements usually work in cooperation with each other. Supposedly, a sizable amount of promoters utilize a similar scenario, i.e. use the same combination of core promoter elements for promoter recognition by the basal machinery. If this is true, statistical analysis of the promoter database should be able to verify the known synergetic combinations as well as to reveal new combinations. It is also important to find the exact distances between the elements as well as to classify known promoters by the combinations they utilize.

**Figure 1:**
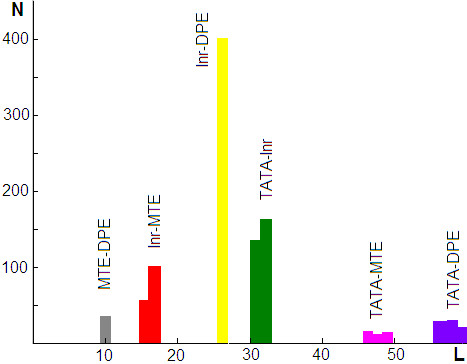
The number of promoters N having one of the combinations of two elements at respective synergetic/cooperative distances L (where statistical significance is meaningful).

The results of the statistical analysis are presented at Table 3 and Figure 1. First, we considered known synergetic combinations. It has been abundantly experimentally confirmed that DPE works with Inr, and their synergism strongly requires an exact spacing, namely 27 bp [21]. We found that statistical significance (*SS*) of over-representation of Inr_DPE promoters (with distance 27 bp between the elements) above number expected by chance is huge (31.2) and, at the same time, the *SS *values for the neighbor distances are negative (see line 1 at Table 3). This data not only strongly supports well-known experimental fact, but also demonstrates the ability of applied statistical analysis to test cooperation of the core elements. Moreover, we confirmed other known experimentally defined combinations: Inr and MTE, and MTE and DPE with spacing distances as expected [2], namely 17 and 10 respectively (see Table 3). The respective subsets of promoter sequences may be found in Supplemental Sequences S6-S8 (see Additional file 2). Note that *SS *of over-representation of Inr_MTE combination at distance 16 bp is comparatively large suggesting that this distance also could be synergetic. The latter has not been shown experimentally. The subset of promoters including Inr and MTE at distance 16 bp is presented at Supplemental Sequences S9 (see Additional file 2).

The combination TATA and Inr also can work synergistically [15]. Since the maximum of occurrence frequency for the TATA and Inr elements are placed at position -29 and +1, respectively, the expected synergetic distance between them is 29 bp. Surprisingly, the *SS *of over-representation of TATA_Inr combination at distance 29 bp is negative, although *SS *at distances from 30 to 34 are positive with a strong maximum at 31 and 32 bp suggesting synergy at those distances. The promoters with TATA_Inr combination are listed in Supplemental Sequences S10 (see Additional file 2).

The statistical analysis of other possible combinations of core promoter elements suggests cooperation between TATA and DPE at distances 58–60 bp, and TATA and MTE at distances 47–49 bp (see Table 3). The respective subsets of promoters can be found in Supplemental Sequences S11 and S12 (see Additional file 2).

### Over-represented motifs

Analysis of the proximal promoter area (-60 - +40 bp) by the program MEME revealed ten over-represented motifs in *Drosophila *promoters [22]. Motifs three, four, and nine resemble TATA, Inr and DPE consensuses, respectively. Motif ten has been shown to be a new core promoter element, namely MTE [19]. We found that only a portion of promoters (~24%) contain considered combinations of the core promoter elements. This suggests existence of other, still unknown elements and/or combinations. Note that positional distributions of nucleotides are essentially nonrandom in the proximal promoter area even for the subset of promoters without known core promoter elements (compare Supplemental Figures S1a and S1b, see Additional file 1). To uncover over-represented motifs different from the known core elements we examined 857 (25.3% of the total number) promoter sequences with no one of four known core elements by the program MEME at positions from -35 to +35 bp from TSS. Analysis revealed four statistically significant motifs. All of them resemble already known over-represented motifs from the article [22] (see Table 4). It is worth closely considering their positional distributions.

Motif 1 is the most over-represented motif. We scanned the entire promoter database and Inr-less subset of promoters by the Motif 1 consensus with two mismatches (see Table 4, line 1). The resulting positional distributions are presented respectively in Supplemental Figures S7a and S7b (see Additional file 1). One can see an essential over-representation of Motif 1 at positive strand in the area from -50 to +30. Indeed *SS*(-50<*l*<30) = 40.9 and *SS*(-50<*l*<30) = 36.9 for the whole promoter database and Inr-less subset of promoters, respectively. The positional distribution of Motif 1 with one mismatch exhibits the same behavior (not shown). Note the large maximum at position -5 (from the 5'-end of the motif consensus), which is the position +1 for the first 'A' in the consensus. Surprisingly, this maximum is even larger in the Inr-less set of promoters, which poses a question if Motif 1 is able to work as a core promoter element instead of Inr. It is interesting that the occurrence frequency of Motif 1 at the proximal distance from TSS is essentially larger at positive strand than at negative strand (see Additional file 1, Supplemental Figure S7c), which also indirectly suggests that Motif 1 is able to interact with the basal machinery.

Motif 2 is essentially over-represented at positive strand in the area from -70 up to +10 bp (see Additional file 1, Supplemental Figure S8a); the occurrence frequency in the area from -40 to +10 is much larger at positive strand than at negative strand (see Additional file 1, Supplemental Figure S8b).

Motif 3 has a huge over-representation in the wide area from -130 to +20 at both strands; the occurrence frequency is up to eight-fold higher than expected by chance (formula I from **Data and Methods**) (see Additional file 1, Supplemental Figures S9a and S9b). Motif 4 is largely overrepresented practically in all promoter area, especially from -150 to +50 bp, at both strands (see Additional file 1, Supplemental Figures S10a and S10b). Usually, transcription factor binding sites that regulate transcription by interacting with the basal machinery exhibit such behavior.

We also examined via the program MEME the TATA-less subset of promoters in the area from -40 to -10 bp as well as DPE-less and MTE-less subset in the area from +10 to +40. In the TATA-less subset of promoters MEME found motif 5 that resembles the motif 6 from the article [22] (Table 4, line 5). The positional distribution of the motif 5 in the TATA-less promoters (positive strand) is presented at Supplemental Figure S11a (see Additional file 1). One can see the large over-representation in upstream area up to -120 bp. Similar to the motifs 1 and 2, the occurrence frequency of motif 5 at positive strand is visibly larger than at negative strand at the upstream area up to -90 bp (see Additional file 1, Supplemental Figure S11b). In DPE-less and MTE-less subset of promoters we found two new motifs (Table 4, lines 6 and 7). These motifs are over-represented in the entire promoter area at both strands (see Additional file 1, Supplemental Figures S12 and S13), which is not typical for the core promoter elements.

### Relation to chromatin structure

Involvement of nucleosomes in the promoter activity (e. g. [27-33]) and regulation [34-43] suggests that the nucleosomes would occupy certain positions in the vicinity of promoters, to provide specific spatial environment for the recognition of the promoters, and for interactions with various transcription factors. In our earlier work [44] we addressed this issue by computational mapping the nucleosomes in the vicinity of the TSS of human genes. For this, the nucleosomal DNA AA/TT periodical pattern was used, derived from a collection of experimentally mapped nucleosomes [45]. Two preferred positions for the nucleosome centers relative the TSS have been detected: 43 ± 3 base pairs upstream from the TSS, and 18 ± 9 downstream. These two positions may correspond to two different types of the chromatin local architecture around the promoters – two types of promoters [44]. Alternatively, the preferred positions could reflect two states (dormant and active?) of the promoters of one dominant type. In this study we mapped computationally the nucleosomes around the *Drosophila *promoters of various regulatory types, to compare the data with those for human promoters.

In the Supplemental Figure S14 (see Additional file 1) the combined (superimposed) map of the nucleosomes near the TSS is shown. It displays two maxima. The more prominent maximum corresponds to the nucleosomes centered at around -43 bp from the TSS. This is, apparently, the same preferred position as observed in human promoters. Such remarkable commonality suggests that, indeed, eukaryotic promoters are involved in a very special 3D organization, being spatially linked with the "promoter nucleosomes". The transcription start sites are located within the nucleosomes, 43 base pairs from the dyad axis of the nucleosome, and oriented outwards from the histone surface. This follows from the almost exact divisibility of the distance by the nucleosome DNA structural period: 4 × 10.4 = 41.6 base pairs.

This major preferred position for the "promoter nucleosomes" is characteristic of all types of Drosophila promoters (TATA+, TATA-, DPE+, DPE-, MTE+, MTE-, Inr+), except for Inr- promoters (see Additional file 1, Supplemental Figure S15). This may mean that the Inr-less promoters are not involved in any specific 3D chromatin structure, being, e.g., permanently exposed for a non-specific, non-regulated initiation.

Second, minor preferred position for the nucleosomes in the vicinity of TSS is around +11 bp. It does not have a counterpart in human promoters, as well as the position +18 of human promoters has no counterpart in Drosophila. Only future detailed 3D study of the promoter structure in its chromatin environment may reveal what the preferred positions +11, and +18 correspond to. They may reflect details of remodeling, somewhat different in human and Drosophila.

Interestingly, the TATA promoters (see Additional file 1, Supplemental Figure S15a) demonstrate a rather elaborate pattern of several preferred positions, in addition to the standard -43 peak. This may reflect, again, a TATA-specific subtype of local promoter architectures, or perhaps, a special path of remodeling of the TATA+ promoters.

TATA, MTE and DCE contain AA and TT dinucleotides, only one per motif. This can have only a small modulatory effect on the nucleosome positioning, since typical nucleosomes require 3–4 AA and/or TT dinucleotides distributed in accordance with the nucleosome sequence pattern [46].

## Discussion

Positional distributions of each of the four core promoter elements (TATA, Inr, DPE, and MTE) exhibit essential overrepresentation at their functional positions (see Table 1 and Additional file 1, Supplemental Figures S2-S5) strongly suggesting that sizable amount of promoters utilize them for interaction with the basal machinery.

Surprisingly, a small number of promoters (~16%) comparing with known statistics for *Drosophila *[21,22] include TATA box, although this percentage is consistent with the percentage of TATA promoters in human genome [20,47].

Every fifth promoter has DPE (22%) and a majority of promoters (66%) have an Inr element, which is also consistent with the percentage of the respective elements in human promoters [20]. There are a considerable amount of promoters (~10%) with MTE. As we already mentioned, the MTE is not over-represented at expected functional positions in human promoters. It seems to be odd since the rest of the known core elements are functional (or at least over-represented) in both human and *Drosophila *promoters; moreover it was specifically shown that MTE is functional (*in vitro*) in one human promoter [2]. This contradiction can be explained if we notice that only the first 5 nucleotides from the MTE consensus are really necessary for the MTE recognition by pre-initiation complex (PIC) [2], and this short version of MTE partially includes the sub-element S3 from the DCE (compare CS**ARC **and **AGC**). It suggests that human and *Drosophila *consensuses of MTE are different and also that S3 could be part of MTE.

Motif consensus for a particular element is derived from the sites experimentally found to be functional. Usually the number of experimental sites is limited, making it difficult to build a reliable PWM. It is expected that the majority of putative sites found in the functional window of aligned promoter sequences are functional which allows using these sites for building more realistic motif consensus and/or PWM. Using an earlier developed technique [25], we obtained PWMs for those four elements specifically for *Drosophila *(see pictograms at Table 2 and Additional file 1, Supplemental Tables S1-S4) using sites extracted from the promoter database.

Promoter elements BRE and DCE discovered in human promoters most likely have functional meaning in some *Drosophila *promoters too. Indeed, the number of promoters having combination BRE_TATA at distance 9 bp (in this case 3'-end of BRE and 5'-end of TATA box are connected just like in human promoters [14]) is visibly over-represented compared with the expected number. The sub-elements of DCE also show statistically significant features. Thus, the over-representation of combination Inr and sub-element one (S1) of DCE at distances +6 and +7 is large. The combination of Inr and S1 at those distances are found to be functional in several human promoters [19]. The sub-element two (S2) shows significant over-representation at certain distances from Inr. The sub-element three (S3) is also overrepresented at expected positions from +19 to +31 from TSS.

Typically, transcription initiation is regulated by a combination of the core promoter elements. The synergism between the elements usually requires exact spacing [1,2]. Statistical analysis of the promoter database allows an identification of synergetic/cooperative distances. Thus, our analysis confirms experimentally defined distances between Inr and DPE – 27 bp; Inr and MTE – 17 bp; MTE and DPE – 10 bp (see Table 3). Surprisingly, the synergetic distances between the TATA and Inr are 31 and 32 bp, not 29 bp as expected based on the position of maximums of the TATA box (-29 bp) and Inr (+1) of respective positional distributions in the promoter area. This finding suggests that in the presence of functional TATA box the TSS position does not necessarily coincide with the center of the Inr element but may be shifted on 2–3 bp in 5' direction. It could be one of the reasons why positional distribution of Inr is asymmetric relative to TSS. The result of analysis also suggests the cooperation between TATA and DPE at distances 58–60 bp as well as the possibility of TATA and MTE cooperation at distances 47–49 bp. The Inr_MTE combination is also over-represented at a distance of 16 bp (not only 17 bp), although experiments showed synergism only at 17 bp [2]. Overall, the proposed technique is sensitive to the spacing between core elements and can be recommended for examination of other elements, as well as for analysis of promoter databases for other species.

Our estimates show that only 24% of promoters utilized known and proposed synergetic combinations while 25% of promoters contain none of the known four core elements. That encourages the search of new elements. The analysis of positional distribution of over-represented motifs revealed by the program MEME leads to several suggestions.

1. Motif 1 (Table 4, first line) could be a core promoter element, since a) the occurrence frequency of this motif obtained on 3393 aligned promoter sequences (on positive strand) has a strong maximum at TSS area (namely, at position +1 for the first 'A' from the 5'-end); b) this maximum is even larger on Inr-less set of promoters, excluding possible interference of Inr element; c) there is no such maximum at negative strand.

2. Motifs 2 and 5 are highly over-represented in the proximal promoter area, namely in the area where pre-initiation complex interacts with DNA. In addition, the occurrence frequency at the DNA positive strand in the over-represented area is essentially larger than at the negative strand. As follows from the previous analysis, the typical features of core promoter elements are a) a narrow functional window and b) distribution on the positive strand is visibly different from those on the negative strand. (Note that TFBS for the majority of specific TFs are placed on both strands). While the motif 1 has both features of the core elements (a and b), the motifs 2 and 5 have only one (b). At the same time the distributions of the motifs 2 and 5 still have a relatively narrow region of overrepresentation covering the basal machinery area. One may speculate that these motifs still could be a target for PIC, or e.g. a target for repressors preventing PIC-DNA interaction.

3. Motifs 3 and 4 are also highly over-represented in the proximal promoter area on both strands. They most likely are transcription factor binding sites for some (not general) TFs.

## Conclusion

Statistical analysis of the *Drosophila *promoter database revealed the major features of *Drosophila *promoters. We summarize here the main results.

1. The sets of promoter sequences utilizing the TATA box, and/or Initiator, and/or DPE, and/or MTE elements for DNA-PIC interaction are presented. The positions of the elements are marked to simplify experimental verification. The position weight matrices for these four elements as well as their optimal cutoff values are obtained.

2. There is statistical evidence that BRE and DCE, the core promoter elements shown to be functional in human promoters, are most likely functional in some *Drosophila *promoters too.

3. The sets of promoter sequences presumably utilizing synergetic combinations of two core elements, TATA and Inr, Inr and DPE, Inr and MTE, and DPE and MTE, are represented. There are also the sets of promoters with suggested synergetic combinations (not shown experimentally but statistically significant): TATA and DPE, TATA and MTE, and TATA and BRE.

4. The synergetic distances between the elements are established. In addition to known from the experiment synergetic distances such as between Inr and DPE (27 bp), Inr and MTE (17 bp), MTE and DPE (10 bp) we found synergetic distances between TATA and Inr (30–34 bp), Inr and MTE (16 bp), TATA and DPE (58–60 bp), and TATA and MTE (47–49 bp).

5. Over-represented motif 1 (Table 4, line 1) can be a new core promoter element.

6. Motifs 2 and 5 (Table 4, lines 2 and 5) could be elements for DNA-PIC interaction or binding sites for silencers or repressors.

7. Motif 3 and 4 (Table 4, lines 3 and 4) are most likely transcription factor binding sites.

8. Some of statistical features are similar between *Drosophila *and Human promoters. Thus, the percentages of promoters containing core promoter elements such as TATA, Inr, and DPE as well as their synergetic combinations are comparable. The functional positions of the core promoter elements as well as the distances between elements in synergetic combinations are the same for *Drosophila *and Human promoters. Exception is the distances between TATA box and others elements (Inr and DPE), which are longer (approximately on two bp) in *Drosophila *promoters than in Human.

9. The relationship of the local chromatin architecture (nucleosome positioning) with certain types of core promoter was elucidated. In particular, TATA+ and Inr- promoters show two distinct types of the chromatin organization.

## Methods

A total of 3393 non-redundant *Drosophila melanogaster *promoter sequences from the "Orthomine Database" (P. Cherbas and S. Middha, pers. comm.) were used for statistical analyses. The database was constructed as the nonredundant union of 3 published *Drosophila *promoter sequence databases [21,22,48]. In the case of Kutach and Kadonaga's database [21] some experimentally-determined TSSs had been rejected in favor of positions suggested by sequence analysis; in those cases the "Orthomine database" employed the original (experimental) TSS. In those few cases where the TSS position could not be unambiguously derived from the published papers, the sequence was omitted. For each sequence the unambiguous genomic sequence was retrieved (*Drosophila *genome annotation v4.1); those sequences that could not be unambiguously assigned to a single genomic location were omitted. In each case the genomic sequence from -250 to +100 (TSS = +1) was recovered. The final database includes 3393 sequences (1908 from Ohler et al. [22], 157 from Kutach and Kadonaga [21], 1328 from the EPD). When the entire set is compared to the current *Drosophila *annotation the modal deviation between the database TSS and the annotated TSS is equal to 0.

The software package Promoter Classifier [49,50] was applied for data manipulation. We also created multiple Windows-based C++ programs to accommodate calculations.

We exploited the idea that motifs necessary for transcription regulation are overrepresented in a particular area of promoter region. So the statistical analysis of averaged positional distribution of the element's occurrence frequency (*OF*_*i *_= *n*_*i*_/*N*_*s*_, where *n*_*i *_is the number of promoters containing a considered element centered at position *i *in *N*_*s *_aligned promoter sequences) is the main method of our investigation. We use the term 'functional window' to designate the positions of the center of the site relative to TSS (the distances between 5'-end and the center of motifs were defined as in Table 1, column 3), where the occurrence frequency of the considered element is much larger than expected. Thus, we suppose that sites appearing in that window are likely to have a functional (biological) meaning. To formalize 'over-representation' we consider parameter of statistical significance derived from Chi-test [51]:

SS=(Nreal−Nrandom)/Nrandom,     (I)
 MathType@MTEF@5@5@+=feaafiart1ev1aaatCvAUfKttLearuWrP9MDH5MBPbIqV92AaeXatLxBI9gBaebbnrfifHhDYfgasaacH8akY=wiFfYdH8Gipec8Eeeu0xXdbba9frFj0=OqFfea0dXdd9vqai=hGuQ8kuc9pgc9s8qqaq=dirpe0xb9q8qiLsFr0=vr0=vr0dc8meaabaqaciaacaGaaeqabaqabeGadaaakeaacqWGtbWucqWGtbWucqGH9aqpcqGGOaakcqWGobGtdaWgaaWcbaGaemOCaiNaemyzauMaemyyaeMaemiBaWgabeaakiabgkHiTiabd6eaonaaBaaaleaacqWGYbGCcqWGHbqycqWGUbGBcqWGKbazcqWGVbWBcqWGTbqBaeqaaOGaeiykaKIaei4la8YaaOaaaeaacqWGobGtdaWgaaWcbaGaemOCaiNaemyyaeMaemOBa4MaemizaqMaem4Ba8MaemyBa0gabeaaaeqaaOGaeiilaWIaaCzcaiaaxMaadaqadaqaaiabbMeajbGaayjkaiaawMcaaaaa@5258@

where *N*_*real *_is the total number of sites found by position weight matrix (PWM) or motif consensus in the considered window and *N*_*random *_is the total number of sites found in the randomly generated control sequences with the same percentage of nucleotides as in the promoter sequences at the same positions. To find the distribution of the element's occurrence frequency we scan each promoter sequence at each position by respective PWM or motif consensus. We examine the presence of the core promoter elements and relations between the elements in different subsets of *Drosophila *promoters. To implement this strategy we divided datasets of promoters to subsets.

To generate the random sequences we first calculated the percentage of nucleotides at each position averaged over all 3393 aligned promoter sequences. Then we generated 100,000 sequences with length equal to promoter length. The probability of finding each nucleotide at each position is proportional to the calculated above percentage. Note that we do not use a conventional model of randomly shuffled sequences as the control. The main reason for this is the essential in-homogeneity of the nucleotide positional distributions in the promoter area (see Additional file 1, Supplemental Figures S1a and S1b). As a result of such distributions, the *SS *values built using shuffled sequences are strongly biased. For example, let's consider a hypothetical motif (with no biological sense) with dominant composition of A and T nucleotides. With shuffled random sequences, such motif will show overrepresentation (large positive SS) at positions from -250 to -150 and from +50 to +100 and under-representation at positions from -25 to -5 (large negative SS). The same motif will not show significant SS values at any positions if our random sequences will be in use. Thus the control sequence set designed here allows eliminating the biases related to strong positional in-homogeneity of promoter area.

The following procedure was applied to obtain PWM for each core promoter element (this is a simplified and modified version of PWM building algorithm we developed earlier [52]). First, the approximate position of a functional window for a particular element was defined by examining the occurrence frequency distribution. Second, we analyzed how many mismatches in an "ideal" consensus (consensus defined by the experiments) are allowed. For this we divided the database to two subsets: one with promoters containing sites at any position in the functional window and matching exactly the motif consensus, and another with promoters without such sites. Then we applied motif consensus to the latter subset allowing one mismatch. If the number of sites in the functional window is still essentially overrepresented, we repeat all previous steps allowing two mismatches. We reiterate this cycle up to *n *times, where *n *is the number of mismatches in consensus for which distribution of occurrence frequency (obtained on the datasets of promoters with no sites matching the motif consensus with *n-1 *mismatches) has no over-representation (*SS *= 5 was taken as cutoff value). We assume that sites found inside a functional window by the consensus with *n-1 *mismatches are most likely functional sites. Note that functional windows of all *n *steps do not necessarily coincide. We used these sites from the functional window of step *n-1 *to construct PWM. There are several different approaches to define PWM [53]. We used the form derived from Staden [54] and Bucher [55]. The next step is to define the cutoff value. We realize that PWM should be "stronger" than consensus with *n *mismatches and "weaker" than consensus with *n-1 *mismatches. Our goal is to find such optimal cutoff value *C*_*op *_that PWM with *C*_*op *_find all functional (over-represented) sites. To implement it, we apply PWM with arbitrary *C *= *C*_1 _(we could start with small values, *a priori *less than *C*_*op*_) to promoter database and divide it to two subsets: with sites in the functional window and without such sites. Then we apply the motif consensus with *n *mismatches to the latter subset of promoters Sn1
 MathType@MTEF@5@5@+=feaafiart1ev1aaatCvAUfKttLearuWrP9MDH5MBPbIqV92AaeXatLxBI9gBaebbnrfifHhDYfgasaacH8akY=wiFfYdH8Gipec8Eeeu0xXdbba9frFj0=OqFfea0dXdd9vqai=hGuQ8kuc9pgc9s8qqaq=dirpe0xb9q8qiLsFr0=vr0=vr0dc8meaabaqaciaacaGaaeqabaqabeGadaaakeaacqWGtbWudaqhaaWcbaGaemOBa4gabaGaeGymaedaaaaa@305D@. Thus, we find the number of promoters Nreal1
 MathType@MTEF@5@5@+=feaafiart1ev1aaatCvAUfKttLearuWrP9MDH5MBPbIqV92AaeXatLxBI9gBaebbnrfifHhDYfgasaacH8akY=wiFfYdH8Gipec8Eeeu0xXdbba9frFj0=OqFfea0dXdd9vqai=hGuQ8kuc9pgc9s8qqaq=dirpe0xb9q8qiLsFr0=vr0=vr0dc8meaabaqaciaacaGaaeqabaqabeGadaaakeaacqWGobGtdaqhaaWcbaGaemOCaiNaemyzauMaemyyaeMaemiBaWgabaGaeGymaedaaaaa@345A@ that do not contain sites defined by PWM with *C *= *C*_1_, yet contain sites defined by consensus with *n *mismatches. We should compare this number with Nrandom1
 MathType@MTEF@5@5@+=feaafiart1ev1aaatCvAUfKttLearuWrP9MDH5MBPbIqV92AaeXatLxBI9gBaebbnrfifHhDYfgasaacH8akY=wiFfYdH8Gipec8Eeeu0xXdbba9frFj0=OqFfea0dXdd9vqai=hGuQ8kuc9pgc9s8qqaq=dirpe0xb9q8qiLsFr0=vr0=vr0dc8meaabaqaciaacaGaaeqabaqabeGadaaakeaacqWGobGtdaqhaaWcbaGaemOCaiNaemyyaeMaemOBa4MaemizaqMaem4Ba8MaemyBa0gabaGaeGymaedaaaaa@3726@ – the number of sites from the randomly generated sequences with the same percentage of nucleotides as in the aforementioned subset of promoter sequences Sn1
 MathType@MTEF@5@5@+=feaafiart1ev1aaatCvAUfKttLearuWrP9MDH5MBPbIqV92AaeXatLxBI9gBaebbnrfifHhDYfgasaacH8akY=wiFfYdH8Gipec8Eeeu0xXdbba9frFj0=OqFfea0dXdd9vqai=hGuQ8kuc9pgc9s8qqaq=dirpe0xb9q8qiLsFr0=vr0=vr0dc8meaabaqaciaacaGaaeqabaqabeGadaaakeaacqWGtbWudaqhaaWcbaGaemOBa4gabaGaeGymaedaaaaa@305D@ at the same positions. If Nreal1
 MathType@MTEF@5@5@+=feaafiart1ev1aaatCvAUfKttLearuWrP9MDH5MBPbIqV92AaeXatLxBI9gBaebbnrfifHhDYfgasaacH8akY=wiFfYdH8Gipec8Eeeu0xXdbba9frFj0=OqFfea0dXdd9vqai=hGuQ8kuc9pgc9s8qqaq=dirpe0xb9q8qiLsFr0=vr0=vr0dc8meaabaqaciaacaGaaeqabaqabeGadaaakeaacqWGobGtdaqhaaWcbaGaemOCaiNaemyzauMaemyyaeMaemiBaWgabaGaeGymaedaaaaa@345A@ <Nrandom1
 MathType@MTEF@5@5@+=feaafiart1ev1aaatCvAUfKttLearuWrP9MDH5MBPbIqV92AaeXatLxBI9gBaebbnrfifHhDYfgasaacH8akY=wiFfYdH8Gipec8Eeeu0xXdbba9frFj0=OqFfea0dXdd9vqai=hGuQ8kuc9pgc9s8qqaq=dirpe0xb9q8qiLsFr0=vr0=vr0dc8meaabaqaciaacaGaaeqabaqabeGadaaakeaacqWGobGtdaqhaaWcbaGaemOCaiNaemyyaeMaemOBa4MaemizaqMaem4Ba8MaemyBa0gabaGaeGymaedaaaaa@3726@, the cutoff value is too small (*C*_1_*<C*_*op*_). We should repeat the procedure every time increasing cutoff value. The value *C*_*m *_is the optimal cutoff value if in the subset of promoters Snm Nrealm≅Nrandomm
 MathType@MTEF@5@5@+=feaafiart1ev1aaatCvAUfKttLearuWrP9MDH5MBPbIqV92AaeXatLxBI9gBaebbnrfifHhDYfgasaacH8akY=wiFfYdH8Gipec8Eeeu0xXdbba9frFj0=OqFfea0dXdd9vqai=hGuQ8kuc9pgc9s8qqaq=dirpe0xb9q8qiLsFr0=vr0=vr0dc8meaabaqaciaacaGaaeqabaqabeGadaaakeaacqWGtbWudaqhaaWcbaGaemOBa4gabaGaemyBa0gaaOGaeeiiaaIaemOta40aa0baaSqaaiabdkhaYjabdwgaLjabdggaHjabdYgaSbqaaiabd2gaTbaakiabgwKiajabd6eaonaaDaaaleaacqWGYbGCcqWGHbqycqWGUbGBcqWGKbazcqWGVbWBcqWGTbqBaeaacqWGTbqBaaaaaa@45EC@.

To define potential synergetic distances between two core promoter elements we examine the statistical significance (*SS*^*l*^) of over-representation of promoters containing a combination:

SSl=(Nreall−Nexp⁡ectl)/Nexp⁡ectl,     (II)
 MathType@MTEF@5@5@+=feaafiart1ev1aaatCvAUfKttLearuWrP9MDH5MBPbIqV92AaeXatLxBI9gBaebbnrfifHhDYfgasaacH8akY=wiFfYdH8Gipec8Eeeu0xXdbba9frFj0=OqFfea0dXdd9vqai=hGuQ8kuc9pgc9s8qqaq=dirpe0xb9q8qiLsFr0=vr0=vr0dc8meaabaqaciaacaGaaeqabaqabeGadaaakeaacqWGtbWucqWGtbWudaahaaWcbeqaaiabdYgaSbaakiabg2da9iabcIcaOiabd6eaonaaDaaaleaacqWGYbGCcqWGLbqzcqWGHbqycqWGSbaBaeaacqWGSbaBaaGccqGHsislcqWGobGtdaqhaaWcbaGagiyzauMaeiiEaGNaeiiCaaNaemyzauMaem4yamMaemiDaqhabaGaemiBaWgaaOGaeiykaKIaei4la8YaaOaaaeaacqWGobGtdaqhaaWcbaGagiyzauMaeiiEaGNaeiiCaaNaemyzauMaem4yamMaemiDaqhabaGaemiBaWgaaaqabaGccqGGSaalcaWLjaGaaCzcamaabmaabaGaeeysaKKaeeysaKeacaGLOaGaayzkaaaaaa@594D@

where Nreal1
 MathType@MTEF@5@5@+=feaafiart1ev1aaatCvAUfKttLearuWrP9MDH5MBPbIqV92AaeXatLxBI9gBaebbnrfifHhDYfgasaacH8akY=wiFfYdH8Gipec8Eeeu0xXdbba9frFj0=OqFfea0dXdd9vqai=hGuQ8kuc9pgc9s8qqaq=dirpe0xb9q8qiLsFr0=vr0=vr0dc8meaabaqaciaacaGaaeqabaqabeGadaaakeaacqWGobGtdaqhaaWcbaGaemOCaiNaemyzauMaemyyaeMaemiBaWgabaGaeGymaedaaaaa@345A@ and Nexp⁡ectl
 MathType@MTEF@5@5@+=feaafiart1ev1aaatCvAUfKttLearuWrP9MDH5MBPbIqV92AaeXatLxBI9gBaebbnrfifHhDYfgasaacH8akY=wiFfYdH8Gipec8Eeeu0xXdbba9frFj0=OqFfea0dXdd9vqai=hGuQ8kuc9pgc9s8qqaq=dirpe0xb9q8qiLsFr0=vr0=vr0dc8meaabaqaciaacaGaaeqabaqabeGadaaakeaacqWGobGtdaqhaaWcbaGagiyzauMaeiiEaGNaeiiCaaNaemyzauMaem4yamMaemiDaqhabaGaemiBaWgaaaaa@37A6@ are the real and expected numbers of pairs of considered elements placed at their functional positions at distance *l *from each other. The expected number is the estimated number of pairs if the presence of one element is independent of the presence of the other. This number may be calculated by formula:

Nexp⁡ectl=∑i=w1w2(pi1*pi+l2),     (IIa)
 MathType@MTEF@5@5@+=feaafiart1ev1aaatCvAUfKttLearuWrP9MDH5MBPbIqV92AaeXatLxBI9gBaebbnrfifHhDYfgasaacH8akY=wiFfYdH8Gipec8Eeeu0xXdbba9frFj0=OqFfea0dXdd9vqai=hGuQ8kuc9pgc9s8qqaq=dirpe0xb9q8qiLsFr0=vr0=vr0dc8meaabaqaciaacaGaaeqabaqabeGadaaakeaacqWGobGtdaqhaaWcbaGagiyzauMaeiiEaGNaeiiCaaNaemyzauMaem4yamMaemiDaqhabaGaemiBaWgaaOGaeyypa0ZaaabCaeaacqGGOaakcqWGWbaCdaqhaaWcbaGaemyAaKgabaGaeGymaedaaOGaeiOkaOIaemiCaa3aa0baaSqaaiabdMgaPjabgUcaRiabdYgaSbqaaiabikdaYaaakiabcMcaPaWcbaGaemyAaKMaeyypa0Jaem4DaCNaeGymaedabaGaem4DaCNaeGOmaidaniabggHiLdGccqGGSaalcaWLjaGaaCzcamaabmaabaGaeeysaKKaeeysaKKaeeyyaegacaGLOaGaayzkaaaaaa@5604@

where *w1 *and *w2 *are the positions of 5'- and 3'-ends of the functional window of element one; pi1
 MathType@MTEF@5@5@+=feaafiart1ev1aaatCvAUfKttLearuWrP9MDH5MBPbIqV92AaeXatLxBI9gBaebbnrfifHhDYfgasaacH8akY=wiFfYdH8Gipec8Eeeu0xXdbba9frFj0=OqFfea0dXdd9vqai=hGuQ8kuc9pgc9s8qqaq=dirpe0xb9q8qiLsFr0=vr0=vr0dc8meaabaqaciaacaGaaeqabaqabeGadaaakeaacqWGWbaCdaqhaaWcbaGaemyAaKgabaGaeGymaedaaaaa@308D@ and pi+l2
 MathType@MTEF@5@5@+=feaafiart1ev1aaatCvAUfKttLearuWrP9MDH5MBPbIqV92AaeXatLxBI9gBaebbnrfifHhDYfgasaacH8akY=wiFfYdH8Gipec8Eeeu0xXdbba9frFj0=OqFfea0dXdd9vqai=hGuQ8kuc9pgc9s8qqaq=dirpe0xb9q8qiLsFr0=vr0=vr0dc8meaabaqaciaacaGaaeqabaqabeGadaaakeaacqWGWbaCdaqhaaWcbaGaemyAaKMaey4kaSIaemiBaWgabaGaeGOmaidaaaaa@32D2@ are the probabilities to find element one at position *i *and element two at position *i+l*, respectively. These probabilities are the respective occurrence frequencies OFi1
 MathType@MTEF@5@5@+=feaafiart1ev1aaatCvAUfKttLearuWrP9MDH5MBPbIqV92AaeXatLxBI9gBaebbnrfifHhDYfgasaacH8akY=wiFfYdH8Gipec8Eeeu0xXdbba9frFj0=OqFfea0dXdd9vqai=hGuQ8kuc9pgc9s8qqaq=dirpe0xb9q8qiLsFr0=vr0=vr0dc8meaabaqaciaacaGaaeqabaqabeGadaaakeaacqWGpbWtcqWGgbGrdaqhaaWcbaGaemyAaKgabaGaeGymaedaaaaa@3160@ and OFi+l2
 MathType@MTEF@5@5@+=feaafiart1ev1aaatCvAUfKttLearuWrP9MDH5MBPbIqV92AaeXatLxBI9gBaebbnrfifHhDYfgasaacH8akY=wiFfYdH8Gipec8Eeeu0xXdbba9frFj0=OqFfea0dXdd9vqai=hGuQ8kuc9pgc9s8qqaq=dirpe0xb9q8qiLsFr0=vr0=vr0dc8meaabaqaciaacaGaaeqabaqabeGadaaakeaacqWGpbWtcqWGgbGrdaqhaaWcbaGaemyAaKMaey4kaSIaemiBaWgabaGaeGOmaidaaaaa@33A5@ calculated based on all promoters from Orthomine Database.

As we see at the **Results **section some of the combinations exhibit over-representation at several distances. To calculate the over-representation of promoters containing both elements at distances from *l *to *l*+Δ*l *we should modify the formula for the expected number:

Nexp⁡ectl,l+Δl=∑i=w1w2((pi1*∏j=1i−1(1−pj1)*(∑k=i+li+l+Δlpk2∏m=1k−1(1−pm1))     (IIb)
 MathType@MTEF@5@5@+=feaafiart1ev1aaatCvAUfKttLearuWrP9MDH5MBPbIqV92AaeXatLxBI9gBaebbnrfifHhDYfgasaacH8akY=wiFfYdH8Gipec8Eeeu0xXdbba9frFj0=OqFfea0dXdd9vqai=hGuQ8kuc9pgc9s8qqaq=dirpe0xb9q8qiLsFr0=vr0=vr0dc8meaabaqaciaacaGaaeqabaqabeGadaaakeaacqWGobGtdaqhaaWcbaGagiyzauMaeiiEaGNaeiiCaaNaemyzauMaem4yamMaemiDaqhabaGaemiBaWMaeiilaWIaemiBaWMaey4kaSIaeuiLdqKaemiBaWgaaOGaeyypa0ZaaabCaeaacqGGOaakcqGGOaakcqWGWbaCdaqhaaWcbaGaemyAaKgabaGaeGymaedaaaqaaiabdMgaPjabg2da9iabdEha3jabigdaXaqaaiabdEha3jabikdaYaqdcqGHris5aOGaeiOkaOYaaebCaeaacqGGOaakcqaIXaqmcqGHsislcqWGWbaCdaqhaaWcbaGaemOAaOgabaGaeGymaedaaOGaeiykaKcaleaacqWGQbGAcqGH9aqpcqaIXaqmaeaacqWGPbqAcqGHsislcqaIXaqma0Gaey4dIunakiabcQcaQiabcIcaOmaaqahabaGaemiCaa3aa0baaSqaaiabdUgaRbqaaiabikdaYaaaaeaacqWGRbWAcqGH9aqpcqWGPbqAcqGHRaWkcqWGSbaBaeaacqWGPbqAcqGHRaWkcqWGSbaBcqGHRaWkcqqHuoarcqWGSbaBa0GaeyyeIuoakmaarahabaGaeiikaGIaeGymaeJaeyOeI0IaemiCaa3aa0baaSqaaiabd2gaTbqaaiabigdaXaaakiabcMcaPiabcMcaPaWcbaGaemyBa0Maeyypa0JaeGymaedabaGaem4AaSMaeyOeI0IaeGymaedaniabg+GivdGccaWLjaGaaCzcamaabmaabaGaeeysaKKaeeysaKKaeeOyaigacaGLOaGaayzkaaaaaa@8B5D@

## Authors' contributions

NIG carried out all calculations related to analysis of the promoter elements, participated in the design of respective part of this study and drafted the manuscript. ENT conceived the idea of the chromatin-related part of the study and drafted the respective chapter. IPI conceived the general idea of the presented study, participated in its design, lead an overall coordination, carried out all chromatin-related calculations and helped to draft the manuscript. All authors read, edited and approved the final manuscript.

## Supplementary Material

Additional File 1 This file includes additional text, graphs and tables representing the mentioned results. Click here for file

Additional File 2 *Drosophila *promoter sequences with mapped core promoter elements and combinations thereof are available as the Supplemental Sequences. Click here for file

## Figures and Tables

**Table 1:** The parameters of core promoter elements. List of the core promoter elements (col. 1); motif consensus in a NC-IUB nomenclature [56] (col. 2); the length of motif (at left) and the distance between center and 5' end (at right) (col. 3); applied windows for the center of motifs (col. 4); the maximal number of allowed mismatches (***n-1***) in order for motif consensus still to remain functional (col. 5); cutoff value for PWM (col. 6); the absolute number (col. 7) and percentage (col. 8) of promoters with respective core element; statistical significance (*SS*) of the occurrence frequency of an element in the respective window (col. 9). All respective P-values are less than 0.0001, which is considered to be extremely statistically significant. The P-values were obtained using P-Value Calculator [57] from respective Chi (χ) values used for SS calculation [51] for a system with 1 degree of freedom (DF = 1).

**1. Name**	**2. Consensus**	**3. Length/Center**	**4. Window**	**5. *n-1***	**6. Cutoff**	**7. *N***	**8. %**	**9. *SS***
TATA	TATAWAAR	12/3	-33 - -23	1	0.79	549	16.2	46.9
Inr	TCAKTY	12/3	-1 - +9	1	0.70	2257	66.5	32.0
DPE	RGWYV	8/0	+27 - +36	0	0.895	749	22.1	8.4
MTE	CSARCSSAAC	10/0	+17 - +26	2	0.79	344	10.1	20.7

**Table 2:** The pictograms of core promoter elements.

Name	Pictogram
TATA	
Inr	
DPE	
MTE	

**Table 3:** The statistical parameters of combinations of core elements. Combination name (col. 1); position of the center of the first element of the combination in bp (col. 2); distance between the centers of the elements in bp (the suggested synergetic distances marked by **bold font **(col. 3); the percentage (%) (col. 4); the absolute number (N) (col. 5); statistical significance of over-representation of promoters having this combination at respective positions with distance as in col. 3 (col. 6); and respective P-values (col. 7). The P-values were calculated as for the Table 1. The P-values < 0.001 are commonly considered to be extremely statistically significant, and those <0.01 – as very statistically significant.

**1. Combination**	**2. Position**	**3. Distance**	**4. %**	**5. N**	**6. SS**	**7. P-value**
Inr_DPE	-1 - +9	25	0.77	26	-4.8	
		26	1.33	45	-3.7	
		**27**	**11.8**	**401**	**31.2**	<0.0001
		28	1.03	35	-4.8	
		29	0.77	26	-4.0	

Inr_MTE	-1 - +9	15	0.41	14	-3.0	
		16	1.6	55	3.3	
		17	3.0	101	10.0	
		18	0.35	12	-3.8	
		19	0.64	22	-1.3	
		**16–17**	**4.3**	**147**	**8.7**	<0.0001

MTE_DPE	17 – 26	9	0.09	3	-2.1	
		**10**	**0.97**	**33**	**7.3**	<0.0001
		11	0.32	11	0.7	
		12	0.12	4	-1.0	
		13	0.06	2	-0.3	

TATA_Inr	-33 - -23	29	1.4	46	-2.8	
		30	2.4	83	1.8	
		31	3.0	135	8.6	
		32	4.8	163	13.9	
		33	2.0	68	2.2	
		34	1.7	58	1.7	
		35	1.1	36	-1.0	
		**30–34**	**11.5**	**389**	**9.7**	<0.0001

TATA_DPE	-33 - -23	57	0.44	15	-1.1	
		58	0.80	27	2.1	
		59	0.83	28	3.2	
		60	0.56	19	1.7	
		61	0.32	11	0.1	
		**58–60**	**1.9**	**66**	**3.2**	0.0014

TATA_MTE	-33 - -23	46	0.12	4	-1.4	
		47	0.44	15	2.3	
		48	0.35	12	1.2	
		49	0.41	14	2.0	
		50	0.12	4	-1.4	
		**47–49**	**1.1**	**37**	**2.6**	0.0093

**Table 4:** The pictograms and consensuses of overrepresented motifs. The numeral in parentheses in the first column is the numeral of overrepresented motif from the article [22].

Motif	Pictogram	Consensus
1(1)		YGGYCACACT
2(7)		MCAKCHCTRR
3(2)		HATCGATA
4(5)		CAGCTGHT
5(6)		TYRGTATTTY
6		TTKTKTTT
7		MAAARYRAAA

## References

[B1] Smale ST, Kadonaga JT (2003). The RNA Polymerase II Core Promoter. Annu Rev Biochem.

[B2] Lim CY, Santoso B, Boulay T, Dong E, Ohler U, Kadonaga JT (2004). The MTE, a new core promoter element for transcription by RNA polymerase II. Genes Dev.

[B3] Lewis BA, Kim TK, Orkin SH (2000). A downstream element in the human beta-globin promoter: evidence of extended sequence-specific transcription factor IID contacts. Proc Natl Acad Sci USA.

[B4] Roeder RG (1996). The role of general initiation factors in transcription by RNA polymerase II. Trends Biochem Sci.

[B5] Orphanides G, Lagrange T, Reinberg D (1996). The general transcription factors of RNA polymerase II. Genes Dev.

[B6] Nikolov DB, Burley SK (1997). RNA polymerase II transcription initiation: a structural view. Proc Natl Acad Sci USA.

[B7] Hampsey M (1998). Molecular genetics of the RNA Polymerase II general transcriptional machinery. Microbiol Mol Biol Rev.

[B8] Burley SK, Roeder RG (1996). Biochemistry and structural biology of transcription factor IID (TFIID). Annu Rev Biochem.

[B9] Burke TW, Kadonaga JT (1997). The downstream core promoter element, DPE, is conserved from *Drosophila *to humans and is recognized by TAF_II _60 of *Drosophila*. Genes Dev.

[B10] Lemon B, Tjian R (2000). Orchestrated response: A symphony of transcription factors for gene control. Genes Dev.

[B11] Green MR (2000). TBP-associated factors (TAF_II_s): multiple, selective transcriptional mediators in common complexes. Trends Biochem Sci.

[B12] Zenzie-Gregory B, Khachi A, Garraway IP, Smale ST (1993). Mechanism of initiator-mediated transcription: evidence for a functional interaction between the TATA-binding protein and DNA in the absence of a specific recognition sequence. Mol Cell Biol.

[B13] Martinez E, Zhou Q, L'Etoile ND, Oelgeschlager T, Berk AJ, Roeder RG (1995). Core promoter-specific function of a mutant transcription factor TFIID defective in TATA-box binding. Proc Natl Acad Sci USA.

[B14] Tsai FTF, Sigler PB (2000). Structural basis of preinitiation complex assembly on human Pol II promoters. EMBO J.

[B15] O'Shea-Greenfield A, Smale ST (1992). Roles of TATA and initiator elements in determining the start site location and direction of RNA polymerase II transcription. J Biol Chem.

[B16] Emami KH, Jain A, Smale ST (1997). Mechanism of synergy between TATA and initiator: synergistic binding of TFIID following a putative TFIIA-induced isomerization. Genes Dev.

[B17] Lagrange T, Kapanidis AN, Tang H, Reinberg D, Ebright RH (1998). New core promoter element in RNA polymerase II-dependent transcription: Sequence-specific DNA binding by transcription factor IIB. Genes Dev.

[B18] Zhou T, Chiang CM (2001). The intronless and TATA-less human TAF_II _55 gene contains a functional initiator and a downstream promoter element. J Biol Chem.

[B19] Lee DH, Gershenzon NG, Gupta M, Ioshikhes IP, Reinberg D, Lewis BA (2005). Functional characterization of core promoter elements: the Downstream Core Element is recognized by TAF1. Mol Cell Biol.

[B20] Gershenzon NI, Ioshikhes IP (2005). Synergy of human Pol II core promoter elements revealed by statistical sequence analysis. Bioinformatics.

[B21] Kutach AK, Kadonaga JT (2000). The downstream promoter element DPE appears to be as widely used as the TATA box in Drosophila core promoters. Mol Cell Biol.

[B22] Ohler U, Liao G, Niemann H, Rubin GM (2002). Computational analysis of core promoters in the *Drosophila *genome. Genome Biol.

[B23] Orthomine: A Dataset of Drosophila Core Promoters. http://bio.informatics.indiana.edu/capstone/may05/talk_smiddha.pdf.

[B24] MEME home page. http://meme.sdsc.edu/meme/intro.html.

[B25] Eukaryotic Promoter Database. http://www.epd.isb-sib.ch/.

[B26] Database of Transcriptional Start Sites. http://dbtss.hgc.jp/index.html.

[B27] Pina B, Bruggemeier U, Beato M (1990). Nucleosome positioning modulates accessibility of regulatory proteins to the mouse mammary tumor virus promoter. Cell.

[B28] Truss M, Bartsch J, Hache RS, Beato M (1993). Chromatin structure modulates transcription factor binding to the mouse mammary tumor virus (MMTV) promoter. J Steroid Biochem Mol Biol.

[B29] Truss M, Bartsch J, Mows C, Chavez S, Beato M (1996). Chromatin structure of the MMTV promoter and its changes during hormonal induction. Cell Mol Neurobiol.

[B30] Truss M, Candau R, Chavez S, Beato M (1995). Transcriptional control by steroid hormones: the role of chromatin. Ciba Found Symp.

[B31] Beato M (1996). Chromatin structure and the regulation of gene expression: remodeling at the MMTV promoter. J Mol Med.

[B32] Beato M, Candau R, Chavez S, Mows C, Truss M (1996). Interaction of steroid hormone receptors with transcription factors involves chromatin remodeling. J Steroid Biochem Mol Biol.

[B33] Chavez S, Beato M (1997). Nucleosome-mediated synergism between transcription factors on the mouse mammary tumor virus promoter. Proc Natl Acad Sci USA.

[B34] Pumfery A, Deng L, Maddukuri A, de la Fuente C, Li H, Wade JD, Lambert P, Kumar A, Kashanchi F (2003). Chromatin remodeling and modification during HIV-1 Tat-activated transcription. Curr HIV Res.

[B35] Georgel PT (2002). Chromatin structure of eukaryotic promoters: a changing perspective. Biochem Cell Biol.

[B36] Sassone-Corsi P (2002). Unique chromatin remodeling and transcriptional regulation in spermatogenesis. Science.

[B37] Biggin MD, Tjian R (2001). Transcriptional regulation in *Drosophila *: the post-genome challenge. Funct Integr Genomics.

[B38] Hassan AH, Neely KE, Vignali M, Reese JC, Workman JL (2001). Promoter targeting of chromatin-modifying complexes. Front Biosci.

[B39] Wallrath LL, Lu Q, Granok H, Elgin SC (1994). Architectural variations of inducible eukaryotic promoters: preset and remodeling chromatin structures. Bioessays.

[B40] Miller JA, Widom J (2003). Collaborative competition mechanism for gene activation in vivo. Mol Cell Biol.

[B41] Polach KJ, Widom J (1995). Mechanism of protein access to specific DNA sequences in chromatin: a dynamic equilibrium model for gene regulation. J Mol Biol.

[B42] Polach KJ, Widom J (1996). A model for the cooperative binding of eukaryotic regulatory proteins to nucleosomal target sites. J Mol Biol.

[B43] Farkas G, Leibovitch BA, Elgin SC (2000). Chromatin organization and transcriptional control of gene expression in *Drosophila*. Gene.

[B44] Ioshikhes I, Trifonov EN, Zhang MQ (1999). Periodical distribution of transcription factor sites in promoter regions and connection with chromatin structure. Proc Natl Acad Sci USA.

[B45] Ioshikhes I, Bolshoy A, Derenshteyn K, Borodovsky M, Trifonov EN (1996). Nucleosome DNA sequence pattern revealed by multiple alignment of experimentally mapped sequences. J Mol Biol.

[B46] Bolshoy A, Ioshikhes I, Trifonov EN (1996). Applicability of the multiple alignment algorithm for detection of weak patterns: periodically distributed DNA pattern as a study case. Comput Appl Biosci.

[B47] Kim TH, Barrera LO, Zheng M, Qu C, Singer MA, Richmond TA, Wu Y, Green RD, Ren B (2005). A high-resolution map of active promoters in the human genome. Nature.

[B48] Schmid CD, Praz V, Delorenzi M, Périer R, Bucher P (2004). The Eukaryotic Promoter Database EPD. The impact of *in silico *primer extension. Nucleic Acids Res.

[B49] Gershenzon NI, Ioshikhes IP (2005). Promoter Classifier: software package for promoter database analysis. Appl Bioinformatics.

[B50] The software package, Promoter Classifier. http://bmi.osu.edu/~ilya/promoter_classifier/.

[B51] Connor-Linton J Chi square tutorial. http://www.georgetown.edu/faculty/ballc/webtools/web_chi_tut.html.

[B52] Gershenzon NI, Stormo GD, Ioshikhes IP (2005). Computational technique for improvement of the position-weight matrices for the DNA/protein binding sites. Nucleic Acids Res.

[B53] Stormo G (2000). DNA binding sites: representation and discovery. Bioinformatics.

[B54] Staden R (1984). Computer methods to locate signals in nucleic acid sequences. Nucleic Acids Res.

[B55] Bucher P (1990). Weight matrix descriptions of four eukaryotic RNA polymerase II promoter elements derived from 502 unrelated promoter sequences. J Mol Biol.

[B56] Nomenclature for incompletely specified bases in nucleic acid sequences. http://www.chem.qmul.ac.uk/iubmb/misc/naseq.html.

[B57] P-Value Calculator. http://www.graphpad.com/quickcalcs/PValue1.cfm.

