# Supplementary material for: The features of Drosophila core promoters revealed by statistical analysis
Source: BMC Genomics. 2006 Jun 21;7:161. doi: 10.1186/1471-2164-7-161 (PMC1538597; doi:10.1186/1471-2164-7-161)
Supplement: Additional File 1 — This file includes additional text, graphs and tables representing the mentioned results. [file 1471-2164-7-161-S1.doc]

**Supplementary Text, Figures and Tables**

***BRE*.** The TFIIB recognition element had been identified in human promoters [17]. It was shown by crystallographic analysis that BRE works in cooperation with TATA box: TFIID binds to TATA box and TFIIB binds to both BRE and TFIID [14]. In this synergetic combination 3’-end of BRE is placed immediately upstream of the TATA box. The statistical analysis of the human promoter databases showed over-representation of BRE in its functional window suggesting that many human promoters utilize BRE [20]. Thus, it is reasonable to expect the presence and functional significance of BRE in *Drosophila* promoters. The whole database analysis did not allow to distinguish over-representation of BRE in *Drosophila* promoters, even though the occurrence frequency of BRE sites are visibly larger in the area upstream and downstream of the TATA box functional area (see Supplemental Figure S6). Since it was shown experimentally that BRE works in cooperation with TATA, we analyzed only TATA+ (TATA-containing) subset of promoters. Using formula II and IIa (see **Data and Methods**) as well as PWM for the TATA box and consensus for the BRE allowing up to two mismatches, we examined the statistical significance of over-representation of BRE_TATA combination at different distances between the elements. We found that *SS* is largest (*SS*=2.7) for the distance *l*=9 bp between 5’-end of BRE and the center of the TATA box, which is the experimentally defined synergetic distance. The statistical significances for the neighbor distances are negligible: *SS*(*l*=8)=0.15 and *SS*(*l*=10)=-0.25. This finding suggests that some of the *Drosophila* promoters utilize synergetic combination BRE_TATA for transcription. The promoter sequences with this combination are presented at Supplemental Sequences S5 (File 2).

***DCE.*** Downstream core element has been discovered and analyzed in human promoters [3,19]. The specific feature of this element is that it includes three short sub-elements (S1-“CTTC”, S2-“CTGT”, and S3-“AGC”) divided by the distance about 10 bp. It was also shown that sub-element S3 can work alone in some promoters [19]. The statistical analysis of the human promoter databases revealed high statistical significance of over-representation of sub-element S3 at positions from about +20 to +30, as well as a detectable over-representation of sub-element S1 in Inr+ subset of promoters at distances from 6 to 9 bp upstream of Inr element [19]. No significant over-representation of sub-element S2 has been found in human promoters.

The analysis of *Drosophila* database showed analogous results. We found significant over-representation of S1 over expected number (formula II and IIa from **Data and Methods**) in Inr+ promoters at distances 6 and 7 from the center of Inr: *SS*(*l*=6)=7.8 and *SS*(*l*=7)=7.3StD. Sub-element S2 is over-represented in Inr+ promoters at distances 13 and 16 from the center of Inr: *SS*(*l*=13)=7.8, *SS*(*l*=16)=2.8. Sub-element S3 is also evidently over-represented in Inr+ promoters at distances 11, 19 and 24: *SS*(*l*=11)=5.5, *SS*(*l*=19)=4.9, and *SS*(*l*=24)=5.3. At the same time, S3 is also overrepresented in the whole database compared with the expected number in the randomly generated sequences (formula I from **Data and Methods**) at positions from +19 to +31 with absolute maximum at position +20. Thus, the analysis suggests that some of *Drosophila* promoters could include functional elements of DCE.

**
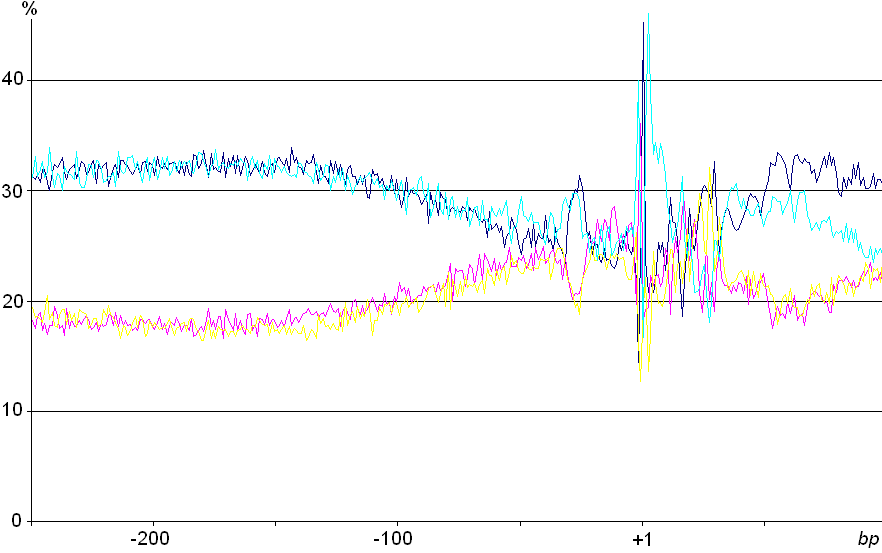
**

**Figure S1a.** The positional distribution of nucleotide averaged on 3393 promoter sequences: C (magenta), G (yellow), A (blue), and T (light blue). The TSS is placed at position +1.

**
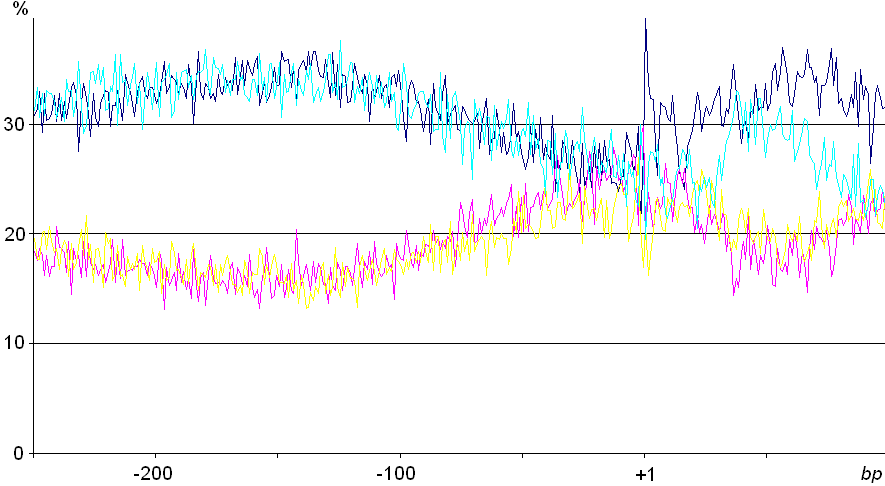
**

**Figure S1b.** The positional distribution of nucleotide averaged on 857 TATA-less, Inr-less, DPE-less, MTE-less promoter sequences: C (magenta), G (yellow), A (blue), and T (light blue). The TSS is placed at position +1.

**
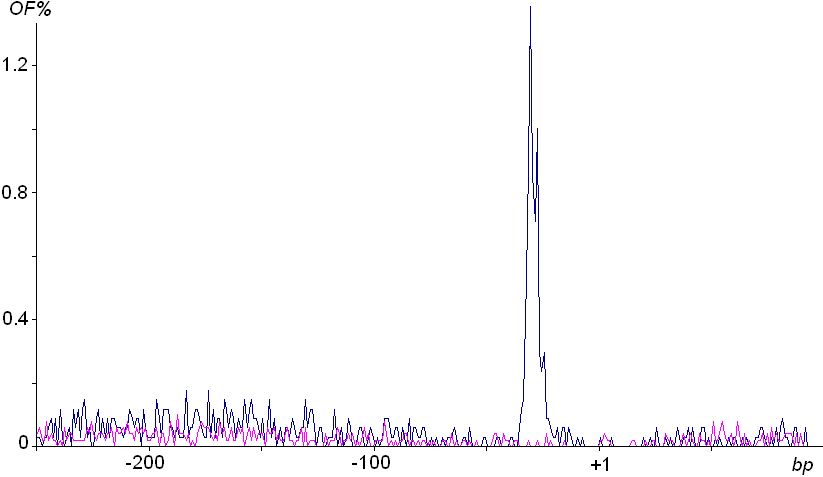
**

**Figure S2a.** The occurrence frequency distribution of the TATA box sites obtained by scanning of 3393 promoters by the consensus “TATAWAAR” with no mismatches (blue curve) and by scanning of randomly generated sequences with the same percentage of nucleotides as in the promoter sequences at the same positions (magenta curve). The TSS is placed at position +1.

**
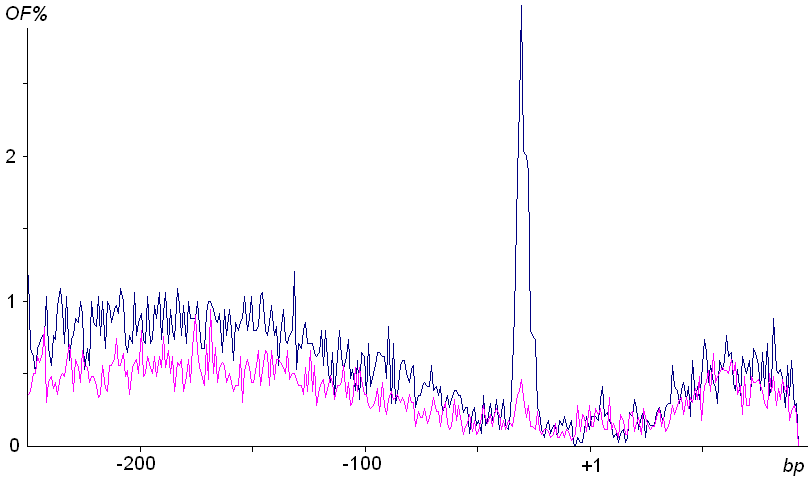
**

**Figure S2b.** The occurrence frequency distribution of the TATA box sites obtained by scanning of 3393 promoters by the consensus “TATAWAAR” with no more than one mismatch (blue curve) and by scanning of randomly generated sequences with the same percentage of nucleotides as in the promoter sequences at the same positions (magenta curve). The TSS is placed at position +1.

**
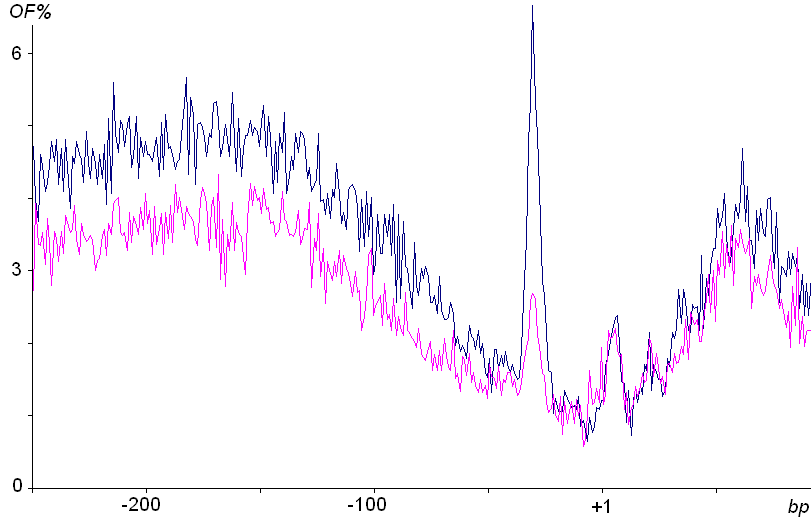
**

**Figure S2c.** The occurrence frequency distribution of the TATA box sites obtained by scanning of 3393 promoters by the consensus “TATAWAAR” with no more than two mismatches (blue curve) and by scanning of randomly generated sequences with the same percentage of nucleotides as in the promoter sequences at the same positions (magenta curve). The TSS is placed at position +1. The local peak at the “functional” position of the magenta curve is a consequence of the identical average local sequence composition of the promoter and random sequences.

**
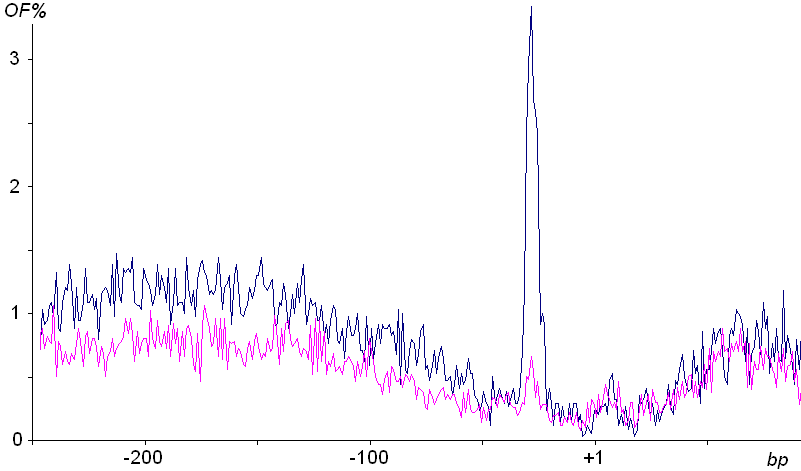
**

**Figure S2d.** The occurrence frequency distribution of the TATA box sites obtained by scanning of 3393 promoters by PWM (blue curve) and by scanning of randomly generated sequences with the same percentage of nucleotides as in the promoter sequences at the same positions (magenta curve). The TSS is placed at position +1.


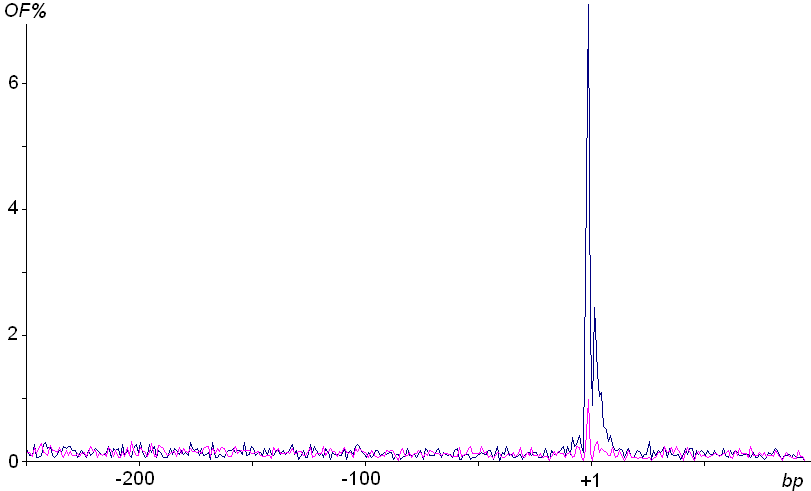


**Figure S3a.** The occurrence frequency distribution of the Inr sites obtained by scanning of 3393 promoters by the consensus “TCAKTY” with no mismatches (blue curve) and by scanning of randomly generated sequences with the same percentage of nucleotides as in the promoter sequences at the same positions (magenta curve). The TSS is placed at position +1.

**
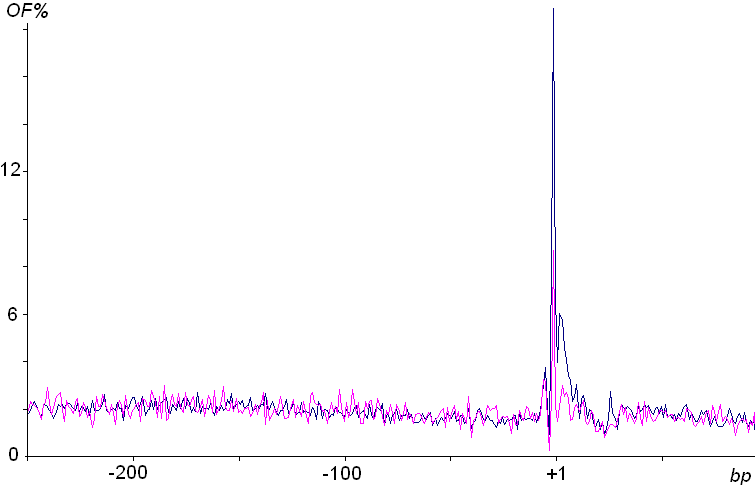
**

**Figure S3b.** The occurrence frequency distribution of the Inr sites obtained by scanning of 3393 promoters by the consensus “TCAKTY” with no more than one mismatch (blue curve) and by scanning of randomly generated sequences with the same percentage of nucleotides as in the promoter sequences at the same positions (magenta curve). The TSS is placed at position +1.

**
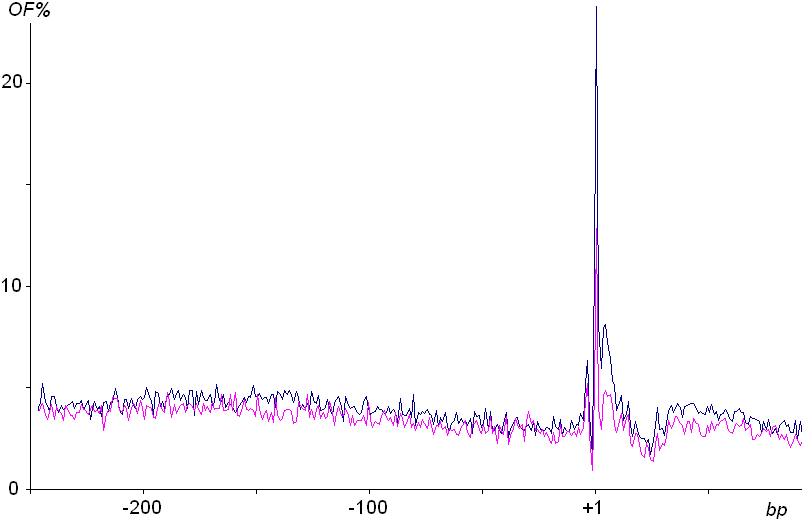
**

**Figure S3c.** The occurrence frequency distribution of the Inr sites obtained by scanning of 3393 promoters by PWM (blue curve) and by scanning of randomly generated sequences with the same percentage of nucleotides as in the promoter sequences at the same positions (magenta curve). The TSS is placed at position +1.


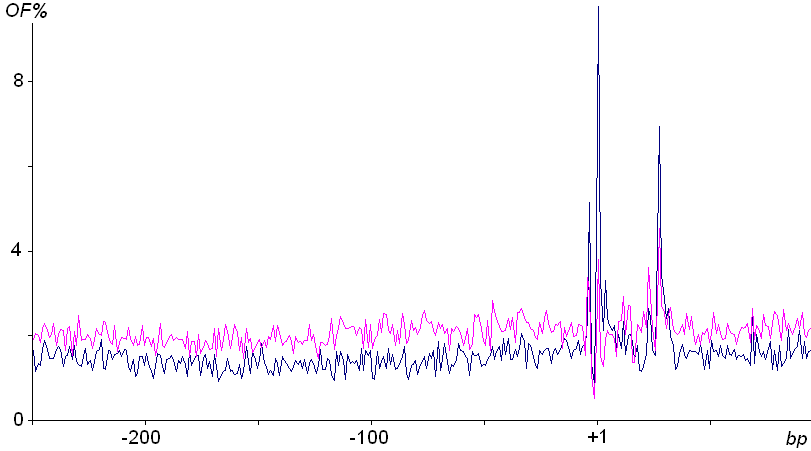


**Figure S4a.** The occurrence frequency distribution of the DPE sites obtained by scanning of 3393 promoters by the consensus “RGWYV” with no mismatches (blue curve) and by scanning of randomly generated sequences with the same percentage of nucleotides as in the promoter sequences at the same positions (magenta curve). The TSS is placed at position +1.


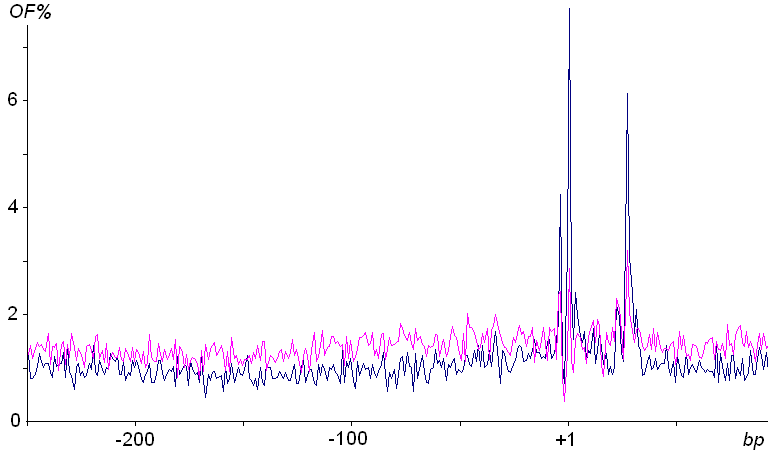


**Figure S4b.** The occurrence frequency distribution of the DPE sites obtained by scanning of 3393 promoters by PWM (blue curve) and by scanning of randomly generated sequences with the same percentage of nucleotides as in the promoter sequences at the same positions (magenta curve). The TSS is placed at position +1.


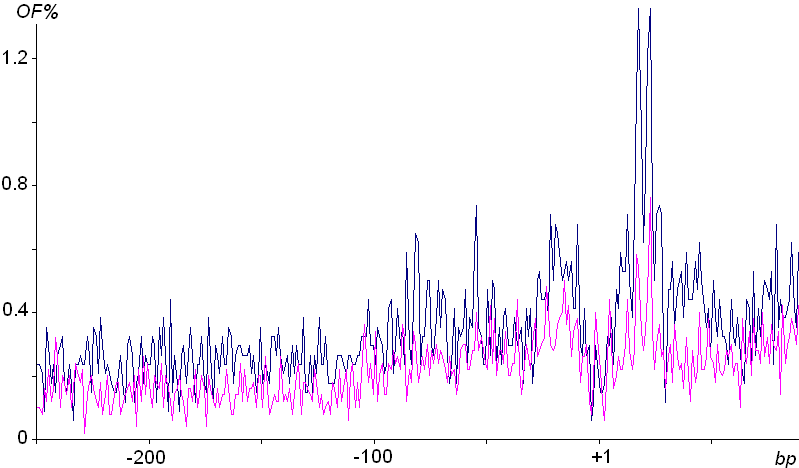


**Figure S5a.** The occurrence frequency distribution of the MTE sites obtained by scanning of 3393 promoters by the consensus “CSARCSSAAC” with no more than two mismatches (blue curve) and by scanning of randomly generated sequences with the same percentage of nucleotides as in the promoter sequences at the same positions (magenta curve). The TSS is placed at position +1.


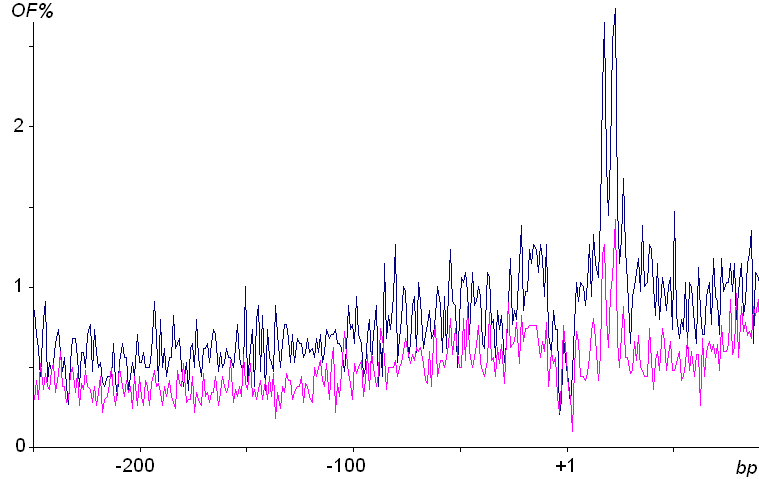


**Figure S5b.** The occurrence frequency distribution of the MTE sites obtained by scanning of 3393 promoters by the PWM built based on consensus “CSARCSSAAC” (blue curve) and by scanning of randomly generated sequences with the same percentage of nucleotides as in the promoter sequences at the same positions (magenta curve). The TSS is placed at position +1.


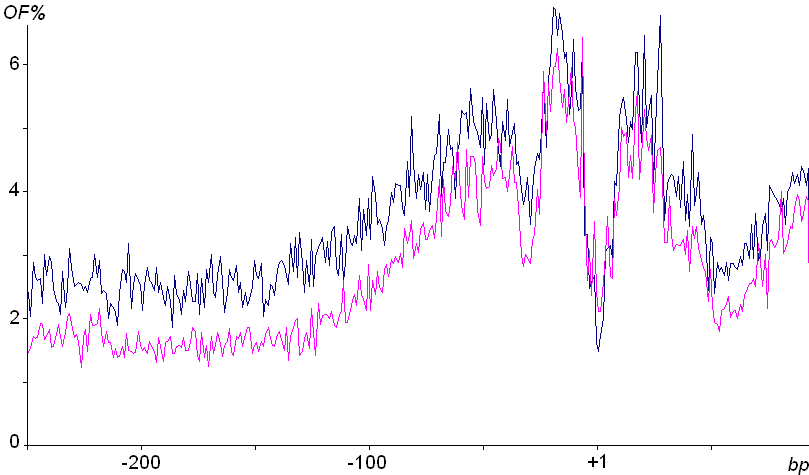


**Figure S6.** The occurrence frequency distribution of the BRE sites obtained by scanning of 3393 promoters by the consensus “SSRCGCC” with no more than two mismatches (blue curve) and by scanning of randomly generated sequences with the same percentage of nucleotides as in the promoter sequences at the same positions (magenta curve). The TSS is placed at position +1.


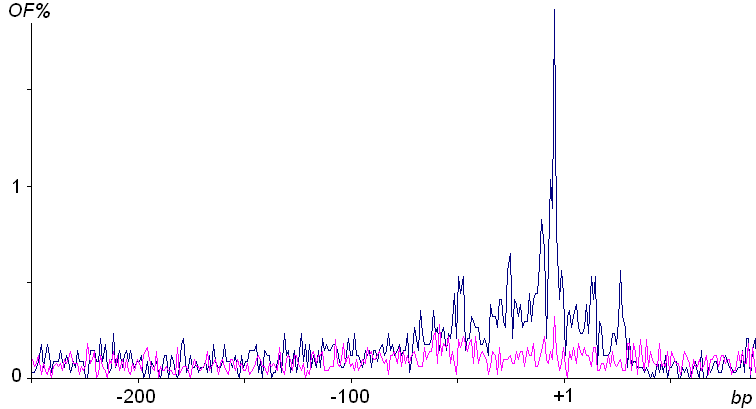


**Figure S7a.** The occurrence frequency distribution of the Motif 1 sites obtained by scanning of positive strand of 3393 promoters by the consensus “YGGYCACACT” with no more than two mismatches (blue curve) and by scanning of randomly generated sequences with the same percentage of nucleotides as in the promoter sequences at the same positions (magenta curve). The TSS is placed at position +1.

**
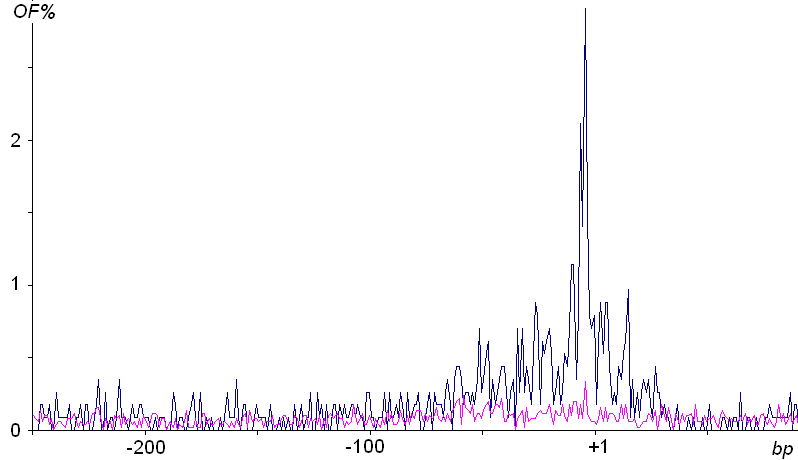
**

**Figure S7b.** The occurrence frequency distribution of the Motif 1 sites obtained by scanning of positive strand of Inr-less promoters by the consensus “YGGYCACACT” with no more than two mismatches (blue curve) and by scanning of randomly generated sequences with the same percentage of nucleotides as in the promoter sequences at the same positions (magenta curve). The TSS is placed at position +1.


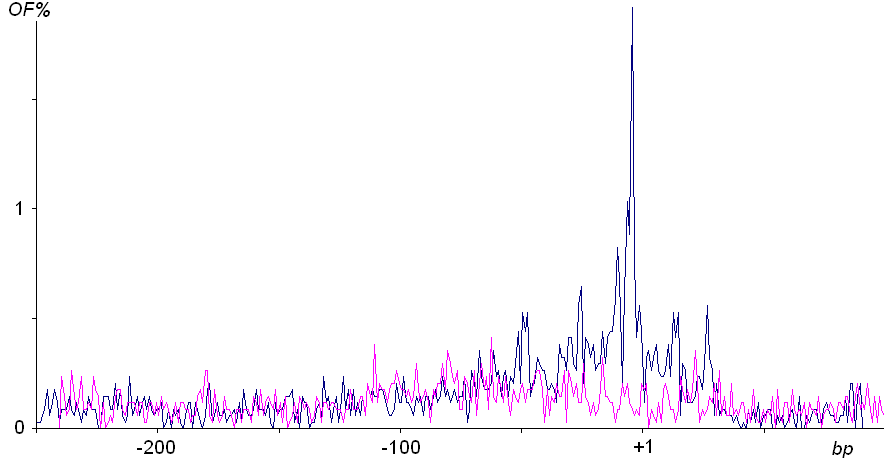


**Figure S7c.** The occurrence frequency distribution of the Motif 1 sites obtained by scanning of 3393 promoters by the consensus “YGGYCACACT” with no more than two mismatches at positive strand (blue curve) and negative strand (magenta curve). The TSS is placed at position +1.


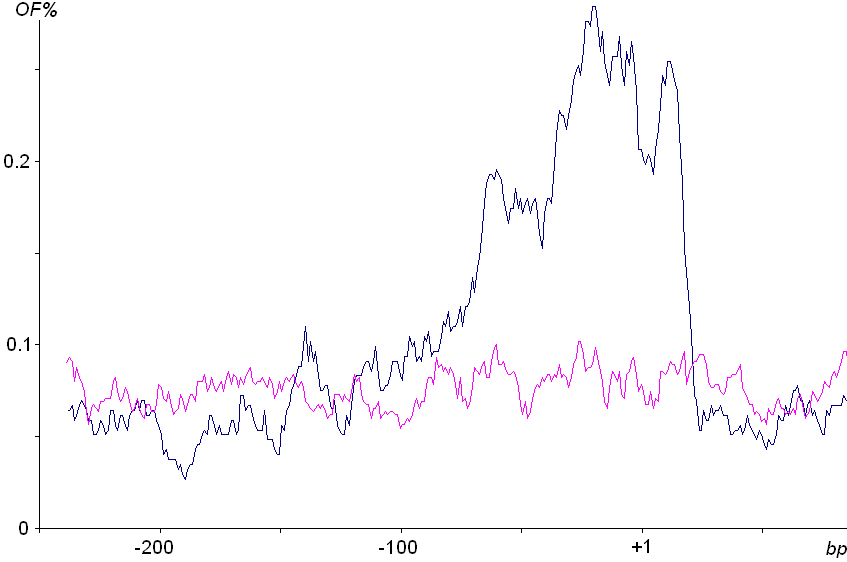


**Figure S8a.** The occurrence frequency distribution of the Motif 2 sites obtained by scanning of positive strand of 3393 promoters by the consensus “MCAKCHCTRR” with no more than one mismatch (blue curve) and by scanning of randomly generated sequences with the same percentage of nucleotides as in the promoter sequences at the same positions (magenta curve). The TSS is placed at position +1.


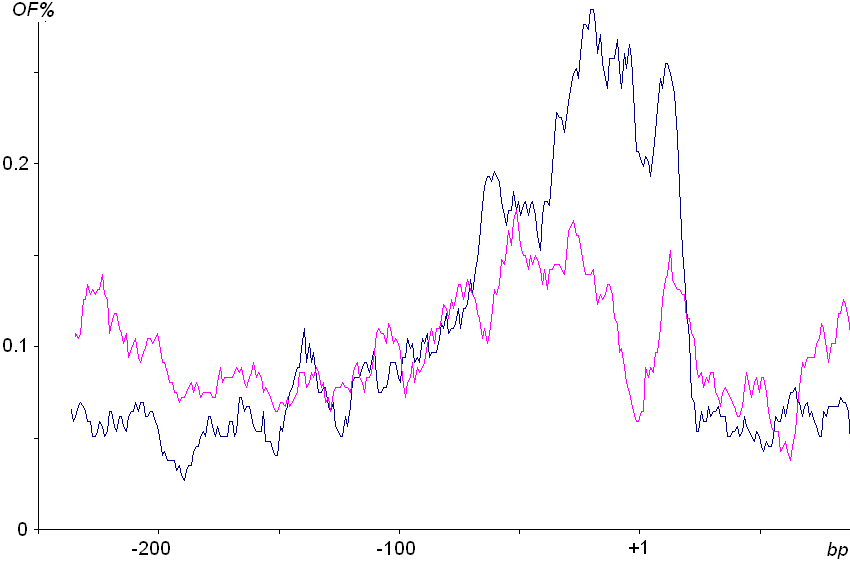


**Figure S8b.** The occurrence frequency distribution of the Motif 2 sites obtained by scanning of 3393 promoters by the consensus “MCAKCHCTRR” with no more than one mismatch at positive strand (blue curve) and negative strand (magenta curve). The TSS is placed at position +1.


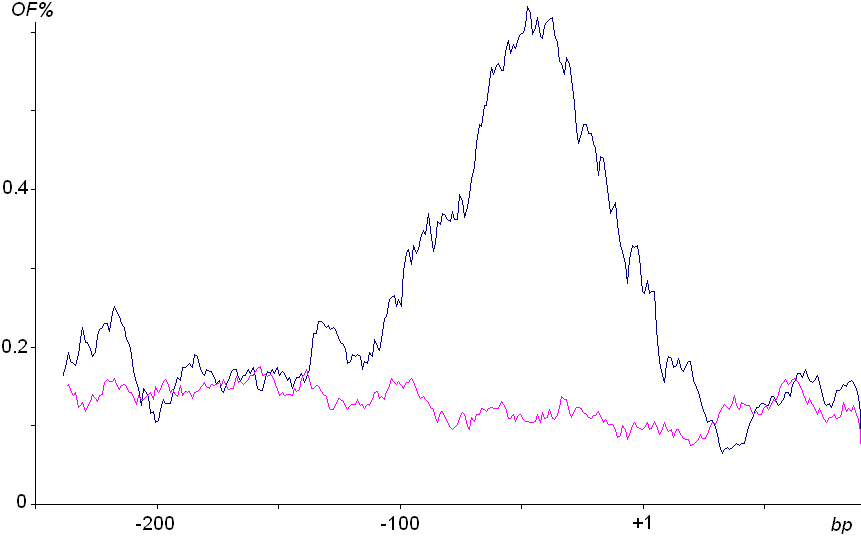


**Figure S9a.** The occurrence frequency distribution of the Motif 3 sites obtained by scanning of positive strand of 3393 promoters by the consensus “HATCGATA” with no more than one mismatch (blue curve) and by scanning of randomly generated sequences with the same percentage of nucleotides as in the promoter sequences at the same positions (magenta curve). The TSS is placed at position +1.


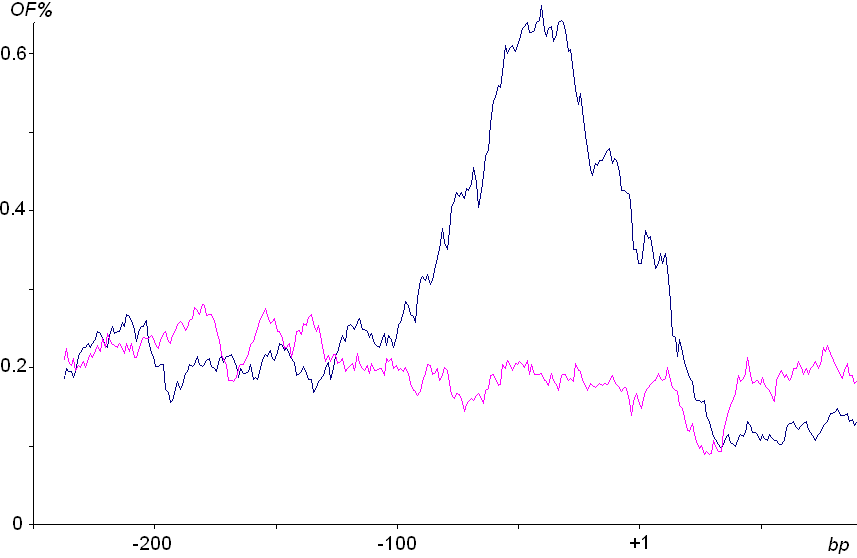


**Figure S9b.** The occurrence frequency distribution of the Motif 3 sites obtained by scanning of negative strand of 3393 promoters by the consensus “HATCGATA” with no more than one mismatch (blue curve) and by scanning of randomly generated sequences with the same percentage of nucleotides as in the promoter sequences at the same positions (magenta curve). The TSS is placed at position +1.


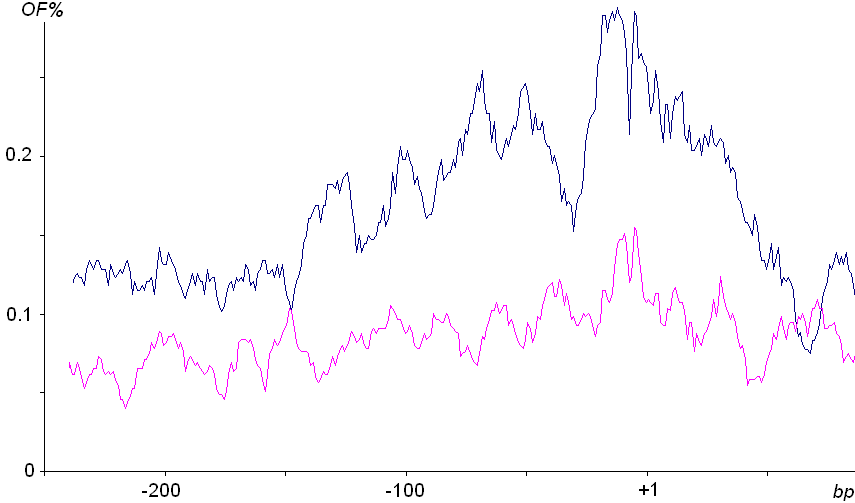


**Figure S10a.** The occurrence frequency distribution of the Motif 4 sites obtained by scanning of positive strand of 3393 promoters by the consensus “CAGCTGHT” with no more than one mismatch (blue curve) and by scanning of randomly generated sequences with the same percentage of nucleotides as in the promoter sequences at the same positions (magenta curve). The TSS is placed at position +1.


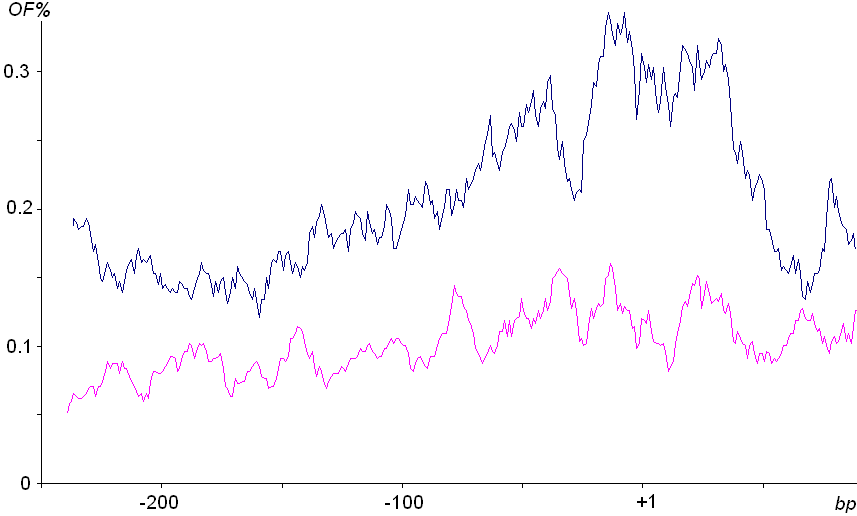


**Figure S10b.** The occurrence frequency distribution of the Motif 4 sites obtained by scanning of negative strand of 3393 promoters by the consensus “CAGCTGHT” with no more than one mismatch (blue curve) and by scanning of randomly generated sequences with the same percentage of nucleotides as in the promoter sequences at the same positions (magenta curve). The TSS is placed at position +1.


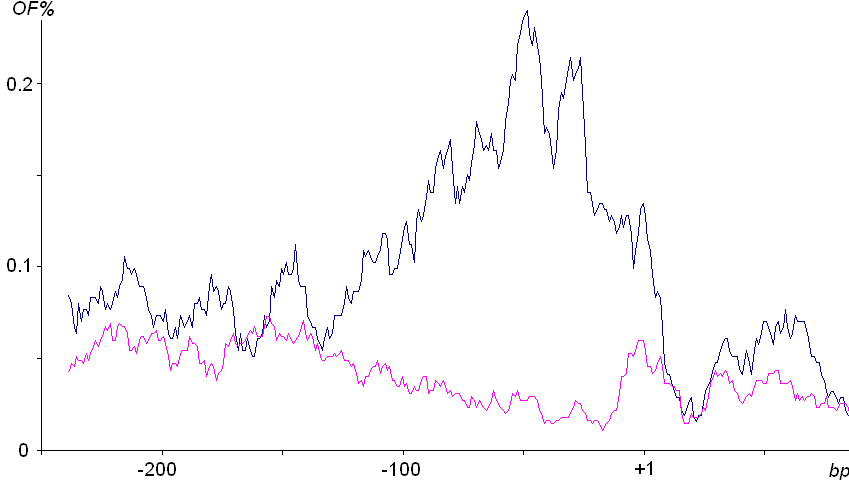


**Figure S11a.** The occurrence frequency distribution of the Motif 5 sites obtained by scanning of positive strand of 2844 TATA-less promoters by the consensus “TYRGTATTTY” with no more than one mismatch (blue curve) and by scanning of randomly generated sequences with the same percentage of nucleotides as in the promoter sequences at the same positions (magenta curve). The TSS is placed at position +1.


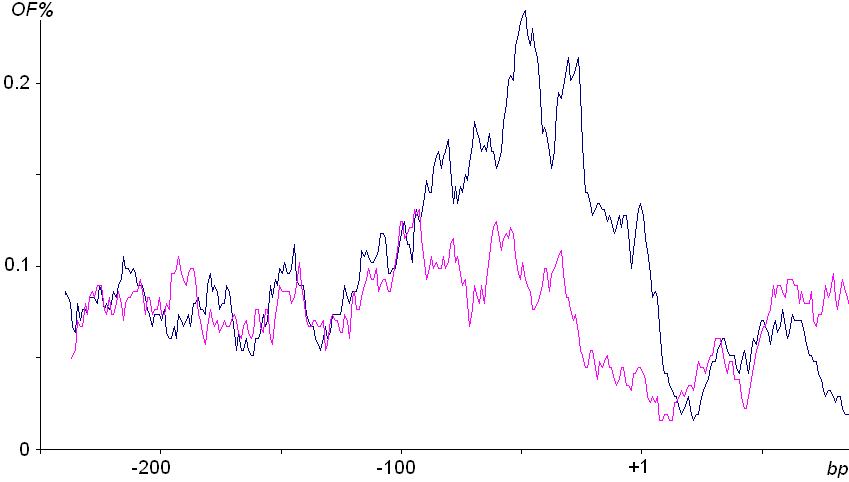


**Figure S11b.** The occurrence frequency distribution of the Motif 5 sites obtained by scanning of 2844 TATA-less promoters by the consensus “TYRGTATTTY” with no more than one mismatch at positive strand (blue curve) and negative strand (magenta curve). The TSS is placed at position +1.


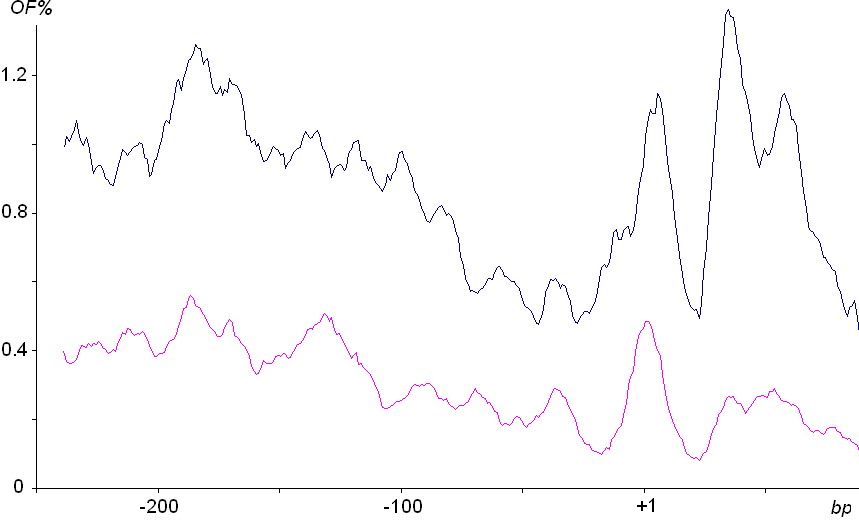


**Figure S12.** The occurrence frequency distribution of the Motif 6 sites obtained by scanning of 2095 TATA-less, DPE-less, MTE-less promoters by the consensus “TTKTKTTT” with no more than one mismatch (blue curve) and by scanning of randomly generated sequences with the same percentage of nucleotides as in the promoter sequences at the same positions (magenta curve). The TSS is placed at position +1.


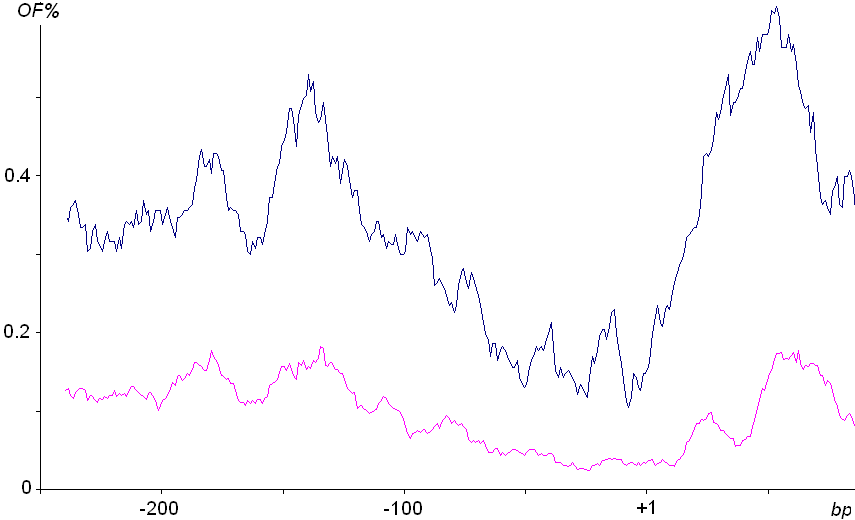


**Figure S13.** The occurrence frequency distribution of the Motif 7 sites obtained by scanning of 2095 TATA-less, DPE-less, MTE-less promoters by the consensus “MAAARYRAAA” with no more than one mismatch (blue curve) and by scanning of randomly generated sequences with the same percentage of nucleotides as in the promoter sequences at the same positions (magenta curve). The TSS is placed at position +1.


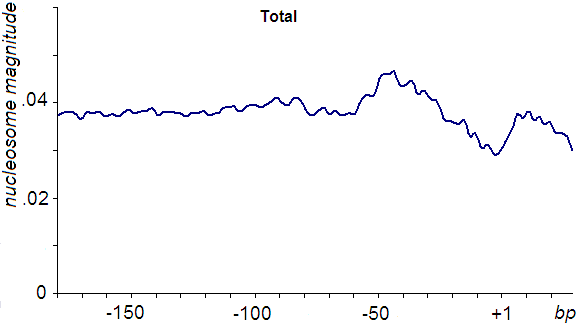


**Figure S14.** Tentative nucleosome positional distribution obtained by scanning of all “Orthomine Database” promoter sequences with the AA/TT nucleosome sequence pattern [45]. Positions refer to the nucleosome center.


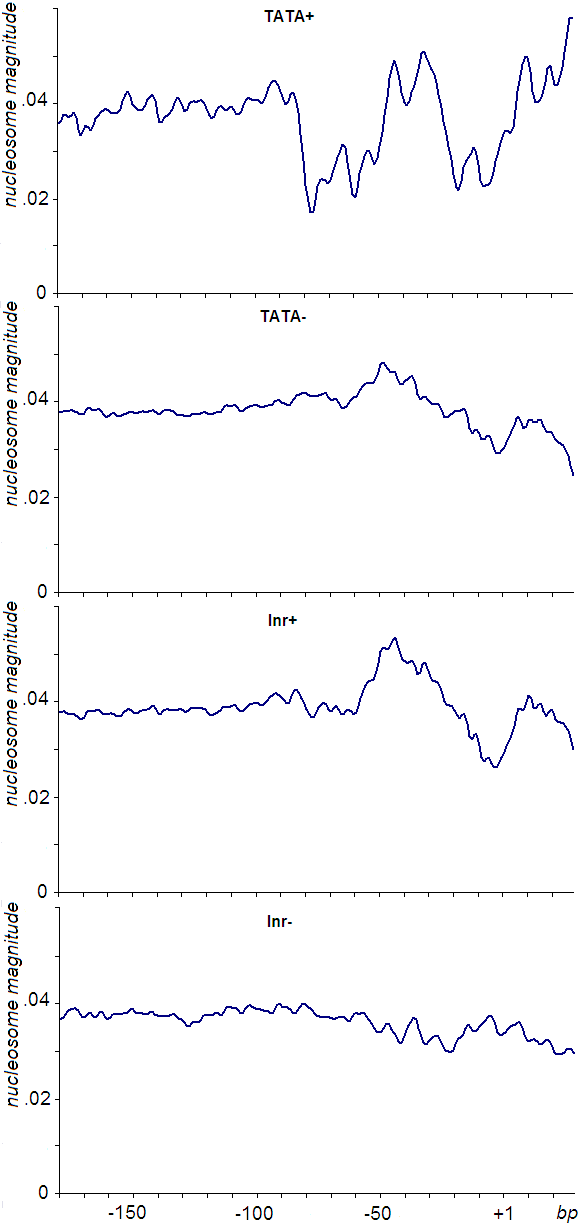


**Figure S15(a).** Tentative nucleosome distributions at the TATA+/- and Inr+/- promoter subsets. Notice distinctive results for the TATA+ and Inr- promoters.


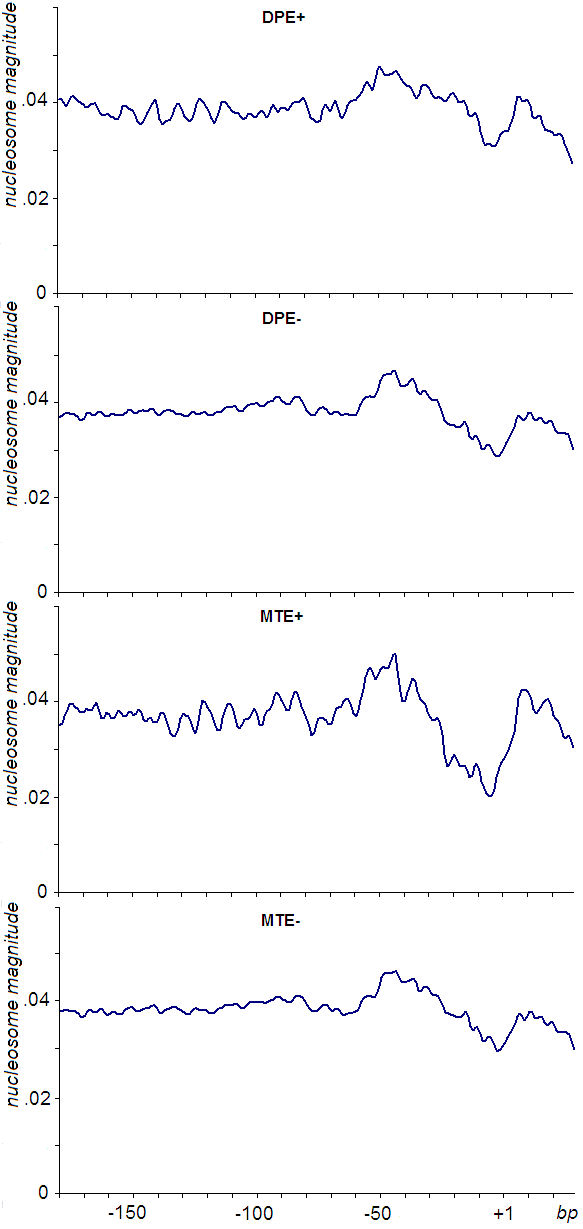


**Figure S15(b).** Tentative nucleosome distributions for remaining promoter subsets.

**Table S1.** The occurrence frequency table built based on sites extracted from DPD sequences at positions from -30 to -27 bp by TATA box motif consensus “TATAWAAR” with one or zero mismatches.

| **position** | **-3** | **-2** | **-1** | **0** | **1** | **2** | **3** | **4** | **5** | **6** | **7** | **8** |
| --- | --- | --- | --- | --- | --- | --- | --- | --- | --- | --- | --- | --- |
| **A** | 80 | 10 | 288 | 4 | 306 | 210 | 314 | 263 | 152 | 65 | 80 | 66 |
| **C** | 85 | 19 | 8 | 3 | 0 | 1 | 0 | 5 | 18 | 110 | 108 | 96 |
| **G** | 114 | 10 | 5 | 2 | 2 | 0 | 4 | 11 | 136 | 87 | 80 | 112 |
| **T** | 41 | 281 | 19 | 311 | 12 | 109 | 2 | 41 | 14 | 58 | 52 | 46 |
| **consensus** |  | **T** | **A** | **T** | **T** | **W** | **A** | **A** | **R** |  |  |  |

**Table S2.** The occurrence frequency table built based on sites extracted from DPD sequences at positions from -1 to +2 bp by Inr motif consensus “TCAKTY” with no more than one mismatch.

| **position** | **-3** | **-2** | **-1** | **0** | **1** | **2** | **3** | **4** | **5** | **6** | **7** | **8** |
| --- | --- | --- | --- | --- | --- | --- | --- | --- | --- | --- | --- | --- |
| **A** | 196 | 54 | 20 | 1052 | 24 | 27 | 43 | 206 | 251 | 285 | 255 | 259 |
| **C** | 230 | 83 | 963 | 13 | 50 | 21 | 440 | 216 | 278 | 285 | 251 | 207 |
| **G** | 260 | 94 | 18 | 16 | 692 | 3 | 60 | 407 | 294 | 197 | 228 | 237 |
| **T** | 456 | 911 | 141 | 61 | 376 | 1091 | 599 | 313 | 319 | 375 | 408 | 439 |
| **consensus** |  | **T** | **C** | **A** | **K** | **T** | **Y** |  |  |  |  |  |

**Table S3.** The occurrence frequency table built based on sites extracted from DPD sequences at positions from -1 to +2 bp by Inr motif consensus “RGWYV” with no mismatches.

| **position** | **0** | **1** | **2** | **3** | **4** | **5** | **6** | **7** |
| --- | --- | --- | --- | --- | --- | --- | --- | --- |
| **A** | 213 | 0 | 210 | 0 | 99 | 92 | 138 | 115 |
| **C** | 0 | 0 | 0 | 277 | 103 | 121 | 106 | 130 |
| **G** | 283 | 496 | 0 | 0 | 294 | 117 | 144 | 81 |
| **T** | 0 | 0 | 286 | 219 | 0 | 166 | 108 | 170 |
| **consensus** | **R** | **G** | **W** | **Y** | **V** |  |  |  |

**Table S4.** The occurrence frequency table built based on sites extracted from DPD sequences at positions from +18 to +23 bp by MTE motif consensus “CSARCSSAAC” with no more than two mismatches.

| **position** | **0** | **1** | **2** | **3** | **4** | **5** | **6** | **7** | **8** | **9** |
| --- | --- | --- | --- | --- | --- | --- | --- | --- | --- | --- |
| **A** | 7 | 15 | 188 | 122 | 21 | 15 | 12 | 159 | 152 | 27 |
| **C** | 176 | 63 | 17 | 2 | 155 | 79 | 81 | 9 | 16 | 160 |
| **G** | 17 | 133 | 4 | 79 | 20 | 109 | 120 | 32 | 29 | 17 |
| **T** | 15 | 4 | 6 | 12 | 19 | 12 | 2 | 15 | 18 | 11 |
| **consensus** | **C** | **S** | **A** | **R** | **C** | **S** | **S** | **A** | **A** | **C** |
