# Supplementary material for: The features of Drosophila core promoters revealed by statistical analysis
Source: BMC Genomics. 2006 Jun 21;7:161. doi: 10.1186/1471-2164-7-161 (PMC1538597; doi:10.1186/1471-2164-7-161)
Supplement: Additional File 2 — Drosophila promoter sequences with mapped core promoter elements and combinations thereof are available as the Supplemental Sequences. [file 1471-2164-7-161-S2.doc]

# Supplementary Sequences for the article

**The features of *Drosophila* core promoters revealed by statistical analysis**

**Naum I. Gershenzon, Edward N. Trifonov and Ilya P. Ioshikhes**

[*(Go to Supplement 2)*](#Supplement2)

[*(Go to Supplement 3)*](#Supplement3)

[*(Go to Supplement 4)*](#Supplement4)

*(*[*Go to Supplement 5*](#Supplement5)*)*

[*(Go to Supplement 6)*](#Supplement6)

[*(Go to Supplement 7)*](#Supplement7)

[*(Go to Supplement 8)*](#Supplement8)

[*(Go to Supplement 9)*](#Supplement9)

[*(Go to Supplement 10)*](#Supplement10)

[*(Go to Supplement 11)*](#Supplement11)

[*(Go to Supplement 12)*](#Supplement12)

**Supplement 1.** [*(Next)*](#Supplement2)

**The list of *Drosophila* promoter sequences with potential TATA box element at its functional position**

The TSS positions are marked by red color

The center of the TATA box positions (second ‘T’) are marked by yellow

>ID_2 P_2 Promoter_2 D1_Promoter for RH51703, -250bp and +50bp []

TACAAGTGCGGGCAGCATAGTATATACACCTATTCCAAGTACTACAGACATTAAAGAATATTTAAAACAAATTAAAGTCCATTCATTCATATACACGTGCCCTAAACGTTTTCCCGCGACTGTCCACACACATTCACACGTAGGCACATGAGAAGTAAATATAGGGGAGACCCGAAGTGGGCAGCAAAACAAGAGCCGAAGTATCGCCAGCGCACGAGAGCTACAAAAATGCAGTAATTCTAGTGTTCGGCTTTCAGTTTCACGAGAGACCTCCAACAGAATACATCGAATTTCGGCGAGAGATTTGCAGCTCAAGAAGAAGAAAGAAGGAAAACCTCTGCGAGTCACCT

>ID_5 P_5 Promoter_5 D1_Promoter for RH54371, -250bp and +50bp [6 ]

GGCTTTTGGGGTGGGATGAAATTATTAGCCAGCGCGAACCTTAACATACAAATAGCCAGCGGATTTGCGCAGCGCTTAAAATTGAGATTTACTGGGGCAATATTTATGGCATAACGCAAGAAGCTGGCGCGAGCTGAACCAATTTGGGGAAAGTACTCACTTATGCCAAGACTCAGCTGCTTATCTAGTGACATTGATAAATGCCCGGGCAATTAGTGCATTGGCTATATAAGGGAAGCTTCCCTCTCAACTTGCCATACAAATCAACATGCGTTCCCTATTCCTGGTCTGTTTGGTCTTCTGCTCCGCTTGGTCCTTGCCAGAATCGGACCTCTTACCCACATCTCCAG

>ID_8 P_8 Promoter_8 D1_Promoter for RH01460, -250bp and +50bp [6 7 ]

AATCTGGAGACTCAAGAATAATACTCGGCCAGAACCCCCAGTGGAAACCCTTGAAATGCCTTTAACTATTGACATTGGCCCATTGCGATGTGCGCTTGCTTTCGTTATGGATATTATGATTATAAGCCAAAAGCTGTCGACACTTTCCTCTGCACATTGCAGACATATTTTTAGGCTGGCCTAATTGAATTTCAAGAGCAGGGGTTACACGAGCACCGCGAAAACTATAAAAGCCATGCGGCAGAATGCAGACATTGCAGGTTTCCAACGACCAATCGCCGCGACTAGTCCGCCCCAGTGAAATATTCAGAATCCAGGAACCCTTTATGTAAAAAGTGTTAGAAATATTG

>ID_10 P_10 Promoter_10 D1_Promoter for RH37353, -250bp and +50bp [5 ]

GGAAAATCGGAAAAAGATACTTGACATTAGCCAATTTGCTCGGGCAAGTGGCAAACTGCGCAGAAAATAGAACAACCCCTGGAAGCAGCTTGTTTGCAGTTTTCCCTTAGTTCCTATGGTTCTATCTTATTGTCGTGTTGGACAAGCCAACAAACAAGTGCATAACGAGCAAATCCAGTTAGTGGCCAGAATGAAGACCCACGGACAGGGCCAGGGCAAAGGTATATAAGCAGGTTGTACGGCACTTGGGTTTCGCATTTTCCTCCGCCGAACGGAAAACTCTATAACGCGCATCGCCCGACTTAGCCTCGAATTGCCCAGTTGCATTTGCCAGGAATCAAAGAAATCCA

>ID_16 P_16 Promoter_16 D1_Promoter for RH26077, -250bp and +50bp [2 ]

TGCTGGTTAAAGAAGTTTCCCATTACCTGTTACCCGATGGATACTTAATAACCAAAAAAAAAAAAAAAAAAGGGGAACGAAATTTTAGAGCTCATCAGAAGTGTTGCAAAATCACTGACGCTGCGAATCAATGCATTTGTGCTGATCCAGAAATCGGTTGCTGAAATCAATTGCTTCCGGCGCGTGCATTATAGTGTGTATAGACTACCGATTCCAAGGGGTATAAAATGCATTGAGTCGCAGCAGTGGGTATGCAGTACAATTTGGTACGGTGTCTGAAAAAGTCGAACTTGGAAGCCACAATGAATCCTCTGCGCACCCTTTGCGTTATGGCCTGCCTTCTGGCGGTC

>ID_17 P_17 Promoter_17 D1_Promoter for RE18044, -250bp and +50bp [5 ]

TATAGCCGAAGATGATAAATCATAGCTTAATCTTGGAGTCAAAGAAAAACGTGAACGTGAAACCGATAACAAATACCTAATCTCGCCCTGGCAACGAAGCGAGTAATCCGCCGGCGGTGGTGCCGCGTTTCTAGCGGGCACTCCACCCACAGAGCTTTTCGGTGAAAAGTTTGCACCGCTCTCGCCGGAGAGCAAAAAGCTCGGCGCTCCGAAAAAGCCGACATATATATACATATCTATGCGAGTGCCTCGGCCAGTTTGGTGCGGCGTCGAGTAGCGAGCGGTTGTGTAGAACAGAATAAAAAAAGAGTTGGAACCCGAAGAATCCAAGCGATTAGCAGCATGTATCA

>ID_19 P_19 Promoter_19 D1_Promoter for RH05106, -250bp and +50bp []

GTGCTATATTTGGATTTTGGAACCACTATGCTATTATGATTGGCTGCGTTTGACTTGATGGCCGTCGAGGTGCATAGCGCTTGGCGGAAGACGTGTGTTGTCCGTGCGGCCAGCGATTGCTGCAATCAGAGCAAGATGGGTGGATGAAACTCGAGTAACTGCATCGTTCGATTTGCGGTCAGTGCGGTTGGAATGAGTTCGGTTCGGTACGGCTTGGTTTCTCTATATAAACGATCCACACTCGCGGCGGTGGCGTAGTGTGCCATGGAACAGCCCAGTGAGAAGACGCAGGAGGGGGAGCAGAAAGATCGCCAAAGTCAGGAAGTGATAGAAGTGGCATCCGATCAAGG

>ID_20 P_20 Promoter_20 D1_Promoter for RE66429, -250bp and +50bp []

ACAAAAACGTCGAAACAAGCCGTTTACTGATAAAGCTTTCAGTTCCAAAACCGAGGGAGAGTATTTGAAACCGGCAGACGATAACGTTTCAGAGTGCTACGATGATCTAATGCTGAATTGTGAGCGCAGTATATGGAACATGGTAGTTAAGAGCGGGGAGCAGGTGAACTTGAACTTGGAGAGGCGGGAGTCTCTTTCGAGGGCCGGAGAACCACTATATATATGGGGCTACGGAAGGCCCATACCCTCAGTCGCTCGCAGTTCGGCGTGGAATCAAACGAGGCTGACAAGTGGACTCATTGCCACATATTGTGAAATAACATAGTTTCCAGAACCCACTATAACCATCC

>ID_27 P_27 Promoter_27 D1_Promoter for RH33713, -250bp and +50bp [1 ]

ACACCGCTGCGGATTACTAAACCACTACCACCACCAAATTAAAATAGTAATAAATAGATTAGTATACATACATACTTTTCTGGGAACTTTTAAATTTATTTTCACTCTGTTTTAAGTTCAGTTTGCTGCTTATCGCACGAAGCTTTGCTTATCAATCGTCCCAGCAACCCCAGAGAGCGATCTCGGCCGGACGCGCGCGCGTTTCGGGCTTAAGAAAAATATTATAAAAGCGAAGACGAGCTTTGGCTTTCGTTTAGTGTCTGAGGCAGAGCTCTGCATGCTTGTCGTTCGAAGCGCTTTTATTCAATCATCGCACTGGGATCGTGAGAAATTGCGCAGATCGTATTGAC

>ID_28 P_28 Promoter_28 D1_Promoter for RE73632, -250bp and +50bp [1 ]

CAAAAATAAATTGGAGAATTGAACATTTCAGTGCTGCACAACATTGCATGGTTGGTGTCCCAACTAAAAAGTAACCCGTGTTAAGAACGTATGAGGGTGGCATCCCTCAAAATCCATGGCGAGCAGTGTTACCGTACTGCGAAAAGTGACCTTAAACCCATCTCAAAAAATATACCAAACGTGTATTTCTCAGCAAAGTTACCACTGGGCACACTACACACTATATAACGAGACGGAAGTCGGAATCGTTTCTTTTCCGTTTTACACGTCTGAAGAGAACAGACATGGTGAGCATCTCGATCGGATAAAATAAATTATATTACTATGTGATGTGTCAAAAAGATGGATGA

>ID_30 P_30 Promoter_30 D1_Promoter for GH19633, -250bp and +50bp [1 ]

CGTATCCATATGTGATTCATTCATAAGCCGGGTGCCCTCGAAACGCGTGAGGCACTTGTGAAATGGCCTCTCTTATCAGGCGGTTAGTTCCCGAATCGATTTCGATGGCGAGCCGTGGAAATTCACGGAAGAGCAGCCTTGCTGGCCCGCGTTCCTATCAGCAGCACTCGCCGCATAATTGAATAATCTGAATCTGCCGAGCGAAGAGCGCGAAACAATAAAAATATCAGATACGCTGCCCGACCGCCGCCAGTTACAGAACAAATTCGCTGCGAACACAACGTGGGCGCATCTCGCAGGTGCCAAAAACGAGGTAGAAAGACAAAGACAAGCAAATGTGCGGGGGGTTT

>ID_36 P_36 Promoter_37 D1_Promoter for RH57538, -250bp and +50bp [2 ]

CATGCCCCCCATTCCGTAGACCCCCCCCCCCCACCCACAACCAACTCATCGTAGCTGCGCCGCCTGTGAAAAACCAAAACAATCGCCGAGCCTCCGCCGGCAGCCAATCAGCTCGTCCGGAGTTTGACCCTCTCACTCGGCGAGGCGATCGTAAAACCCGATTTGCGTGGCGATCGGCCGACTTGTTCTAGTCGCCTCGATGGCCAGGCGTCGCCGGCTGGGTATAAAAGGCCCCGGATCGGAGCAGTTGGCATCAGTTACTCGTGGCCAGAGTAAATGGAAAGATCAAGTGAAAAGCCTACTTTCAGTGGTCTGAAAATCAAGTTGATGTTTGACAAAAAACTGTGGAA

>ID_39 P_39 Promoter_40 D1_Promoter for RH68512, -250bp and +50bp [3 4 ]

ATTCTTGTGTCACCCCATTTTATTTTTAACATTTACATTTACTGTACCCTGAAATTTTAACCCTGACACGGTGTCTTAACGTCATTATTTTATAACCAACCCTTTGCCCGTGATTGTGCGAAATACTTGCTGATATATTTTTACAGCAGACTGGGCAGAAAATCGTCTTTGCGGGCACGCAACCACAAAGTCTAATTGAATTTGGTTGGCAGCACAAAATGCGATATAAACGAGGCATTGCGGTGATTGGAAACAGTTCAGCCCTGTGGCGCACACAGAGCGAAACGGGTAGCGGTTAAGGCGTATCGGTTGGGAATCGGTTCGGTATCGGGTTGACCGATATGGAGCCG

>ID_42 P_42 Promoter_43 D1_Promoter for RH62628, -250bp and +50bp [4 ]

TTCAAGTTCTTTTTTACCTTTCGGAAAATACTTTGTAATATGCAAAACAAGTTCTGTGCCAACAAATGCAGACTAAATGAGACTGTTAACACCCGCTGCATATGACATGATTAATGGTATTTTAGTCTATCTAGTTTGCTTGTGGGTTGCAAGCTCAAGATCAAGATCATCTGTTCCTCTGCCATGGCTGCAACATGACTTGGCTAAACCCAATCTAGTTACTATATAAGCTGGGCTTCGGTTGGGTTTAAGGCATCAGTTACTTGGAAGTCACTACCAGCTACGGATATCCAGTTCATCATGCGCTTCGCTCTACTTGCCGTTCTCCTCATCGGAGTGATCTTTGCCTT

>ID_43 P_43 Promoter_44 D1_Promoter for RH07639, -250bp and +50bp []

CACCGCTCGGCGGTCCGCTAAAAAATGGCGAGCCAAGTAAAGCGGTACCAATGCTAATCCGGCGTCCACACACTGAGCGCCCGCATATGGGTATGTGTGGTATGCTGCGAGCGGTAATTGCAGGGGCTGTAAGAGCAGGAACTGGTTTTTTTTTTTCAGTCATTCTTCGCCAGCCGAATGCGACTGCGCTGCCGGCGGCGACTGCGGCAGCGGCGGATCGGTCGGATATATAAGATAGTCGGGGGCTGGGATTCTGATTAGTCTGCGAGCGCCGTTTACAAGTACATAGCACGTTGAGTTTCCCGAGTGCGTTGCAAGTTTTTTGGGTGCCCCCCAAAATCAAAGTGAAA

>ID_45 P_45 Promoter_46 D1_Promoter for RH19893, -250bp and +50bp [1 ]

CACGTAGATACCGTAGTTTATGTTCTAAAAAAAAATATTTCGAATCTTAAAACTATTTTTTCACTTATTTTGCCTTTCTAAGCAATTTTCTTAAATATTGTAAACCCCGAGTGAGGTTCTGGTTATTGATAGTTCTGATGATGGGCCAGTCAAGTCTTGGATCGATGGCATAGAGAGCCTATTGTATTTTTCCATTGTTGATCTCCCACTGAGATGGCAGGTATAAAATGGGCCACATCGCCGGGTGTTCATCAGTTCTCTCAGAGTCGTCGAGCGAGCAACATGAACGTAGTAAAATCTTTGTGTATTTTGGGTCTGGTTCTCGTCAGCTTGATTGCCACCCAAGCAGC

>ID_50 P_50 Promoter_51 D1_Promoter for RH03284, -250bp and +50bp []

TTACATAAATCTACATTGGTTTTATTTTAAACAATTTAAGGTGTTTTGCTTCCGATTCTCTAAATTTGCTTAGCGTCCAAAGATATAGTGTCACCTCTCTTAATTTCGATTTATCTTGACAATTCCCTACGGTAGTATAAACTGACATTTATAGTGCCTTTGAACTCACAAGTCTACTTTTGCATAAAATTTCTAATTTACATTATAATTATCACCTTAAATCACTATATAAAGCAGATCTTGAAAGGCACAACATCAGTTGTAAAAAGCATTTGCTATGGTGCGACAATTGGTAAAGGCCTATATAAAATACTTAGTCACGAACAATTCTTAGTCTTACTGACCTATTG

>ID_53 P_53 Promoter_54 D1_Promoter for RH45210, -250bp and +50bp [3 ]

ATGAAGTGTTAATAGCTGGCACTTTCACAATTTGTACGAACCTCCTGCCACCCGCACTATTTGCATATACATATGTAAATACACACATACATACATATATAGAGCTATTACCGATTTCACAGCGTCCCCAAAAGTTCTCTCTGTTCTCCCAAAGCTCCCATGATCACTACATGCGTTGATATCGCGGCAGAGGAATCGACGGCGACTGCGGCAGAGCAGCTTATAAATACCAGATGCTCGACGTTCTTGGGCAGTTAGTTTTAAACTGCGACTTGAAGCGGCAACATCGTCGGATCAAGCAACATATAACAACTTTCGTGAGTGCCACAAGTGCCCCTTTTTTACGATTA

>ID_56 P_56 Promoter_57 D1_Promoter for RH48926, -250bp and +50bp [0 1 ]

TTTCCCGATGAGTTATTGATACAAATATAACGAAAATAAGCCGACTCACTAATCATCAGCGAAAAATTGCGATCTCCAGTCTGCTCGAAATTCTGATTAGACAAACTAATCGCCAGTCAGACCCCATGCGTGAAAAAACCCCTTAGGGAGCGATAAGATCCCATGCAGTCACAAATCACTCCCCGCGAAGCCCTCAGATAAAGTAGCAGTGGGGTCCACTATATAAGGAGCGGCTCTGAGTAGTTCCGACCAGAGTGAAACTGAACTTCCATCTGGAATTACCATGTTTCTGGCCAAGAGCATAGTGTGCCTCGCCCTCCTGGCGGTGGCTAACGCCCAATTCGACACCA

>ID_64 P_64 Promoter_65 D1_Promoter for RH52788, -250bp and +50bp [8 ]

TAGAACTAGTTCTTGAATTCAACCGCTTTGAAGGCGTTCAACCTAGCCGTTTGATCCGATTCGATACGATACGATCCGTTCAGATCCGAGGCATGTCATTACAGCTGATCATATGCATGCGGAGCAGTGTCAGCCGTGTCCACCATGTCATGGTGTGTGAACTCTCACTTTCATGAACCCTCCGACTTGGCGGTTTTCTATTGCTGATGTGGTCAGCAGTTGGGTATAAAACTGGCCGGCAGCTCCGTTGACAGATCAGTTCAGAACAAGAGTTCAACCAGAGCGCAACCCAAAACCCACAAACAGCACCATGAAAGTGAGTTATCAAACGGATTTATCCTGATAATTAA

>ID_68 P_68 Promoter_69 D1_Promoter for RH22327, -250bp and +50bp [7 ]

AGAGCGCCAACGGCTTGGCGCCGACATTTCGATACCTTGGCGGCTGTACTTGGCTTTGCATCTGGTAATCCGATGCTCAGCCGATGCCACATCATTAAATTTTTAATTTGTGAGACAAACAAAAAGGAAACGTGCAAAAGCCGTGCCGCAGAACGAGAGCGAATCAAATGAGAGCGAGAGGCCATTAACTTGCATCTGGCAATGCCGTAGCCGCAACGCGAAAGTATATAACAGCCGGCACGCCGCAGAGCTTTTCATTCGCTCAGAAAATTTCAACAGTGAACATCTTGTGCAGAACACAGACCAAATATTCCAAAAATGTCTTTCGTTGGCAAGAAGTACAAGCTGGA

>ID_70 P_70 Promoter_71 D1_Promoter for RH11258, -250bp and +50bp [0 ]

ACGCTTTTAAAAATCTAACTAAACCATAGCACACAAAAAGTAAATAAGGTTGTTAAAACTAAGAATCATTATAATAAATGTAATCATGACTTGTAATTATCTTAGAGTCCCTCTGGATTTGCTGTGGTTTGTTTGTCGTATTTTAAAGCTTTTTCCACCACACAGGTGAATTTATAAGTATGCACTTGAAATTGCTATCTCAGAACTTTTGAGACTTTCGAGTATAAAAACGCAAACAACATTTCAAATCGCCCCAAGTGCGGTGCGTAGAGCTTTAATTGTCGGTTGTGTACGCGGGTGCTCAGAATTTATAGATCCAATAAAAGTTTCCTTGAAATTGCTGGACAAAC

>ID_74 P_74 Promoter_75 D1_Promoter for RH33067, -250bp and +50bp []

AAACATATGTATTATTTTATTAAACTAACGCATAAATGTGTTTCAGTTTTTTTAAGTATAGAACGTGTCTAAAAGCCGAAAATGTTGTAATGTTGGATATGGTCCACGAGTACCTGGTATAAATTCCCGAAACGGCACACGAACCGATCCACGCGCACACATGTGTGAGTGCGCGGACTTCGGGTCGAGCCGATCGTCTGGATGGACAGTTAGTTCGCATATAACGGCGCTTGCTTGCCGACTTCGGTGTTAGTCGCAAGTTGAGAACCACTCGGCATAGAATTAGAATTCCAGAGCCAGAGGAGAGCCGAGAAGAATCGTCCCGATCCGTTCCACTCCGCACTTCGAAT

>ID_76 P_76 Promoter_77 D1_Promoter for RH26328, -250bp and +50bp [1 ]

CATTAAAATTAAGTTAAAAACCACTACTTTGAATTGAAGTTTTTTGCCATGCCCATGAAGACCACTAAAATATGACGTCGGACATCGATTTTCAAGAAACGAGACCGTGTTCTTGTGTGGTTATCACGATCCGTAGGGCCAGCATTCGAGCAGAAATCGTCCCCAAAAAACAGTTTCCCTGCTGGCTGAGACAAGAAAAACGCGTAGATTTGCGAGCAGAGCTATAAAAGGGCGCGCAGACAACGCGCTCTACCCAGTTAAAAAGAGTGCGTTGTGTAGAACAGTCATAAGCAGTCCGCTAAGAGATTAGTAGCAACTACGAAGTGAAGGTGTGTGTATACCAAATTCTC

>ID_78 P_78 Promoter_79 D1_Promoter for RE15249, -250bp and +50bp [6 ]

TTGTAATCGAATCGTTATCAATAGTGCCTTTTTCTATATTTATATATATTTTTACATATGCACAGTAATCAATGGATTGATTATTATTGTTTATTATTGTTTATTATTATTTTGTTTATCTCGAACCTCAGCTGAAATAATCGCAAAAGGGTCGCAACCTGCCAGCAATCACAAATCGTGCGATAAGAAGGTTATCTGCAGCATCAGCACCTTTCTGGGGGCTATAAAAAAGTGATCGCCACCGAATGGGGCGATCAGTTTGACTTTTGAATTCGTCGCGATGTTTTCGATTAACGCCGTGATCCTGGGCATTTTGGTTACTTCCGTTATGGCCCAGGATGCAAGGGGAA

>ID_107 P_107 Promoter_108 D1_Promoter for RH09244, -250bp and +50bp [7 ]

TGTCAATACAGGCATGGAAATTCTCTGAGTGCACTGATGCACTGTATAGTCTAGTCGTTTCCCTTCACTTCCCTCATCACTTAACCACACTCATCACTTTGCGATAATGACGTACGTATGCTTTGGGAGTCCCAACAAAAAAAACACACATGCGCTGCCCCGTCGACGCGGCAGCACAGCAGCAGAGGACGGCGTCAAGAGCGGCTTGAAGCTGGAGCTCGATTTTAAAATGGCCAGCCCGCTCGGCGCACCAGTCACTCTCAGTTGGAACGGCGCGTGAAACGGTTATCGCTCGCTCCACCGGCGCTCTCTCGCCGCTCGCCCCACCAAAGCCCGCATTTAACAGTTGT

>ID_110 P_110 Promoter_111 D1_Promoter for RE03105, -250bp and +50bp []

TTCCGACTTCCGTTATAATTCATTTGTAATATCAAAATACAGTGGCCTCCTCCCGATCAAGCAAATTCAAAATTGTAAGAGAAGAGAAGAATTCAATTACATTACAACAAAAGAGAAGAAAGAAGACAACTTCAAAATGTTCTCTTTTGGTTTCTCTCTTGGTTCTCTGCTCTTTCTCATCAAGATTTTCGAAGCGCGCCCAAAAGTATGCACGAATTGATATAAGTACTACATTACGTTACTTGGCTCACTTCGCTTCGCGCCGCCGAGCAAGAAAGACGTGTGTGCGGAGTCTGCGCGAAACTATAACAGAGTTATATTTGAATATATATCTCAAAATAAAATCGTCG

>ID_111 P_111 Promoter_112 D1_Promoter for RE40695, -250bp and +50bp [8 ]

ACGCTACTGAATAATTAAAATGTTACCGAGGGGATTTTTTACACTATAGAGTACCATCTTCAAAACTCTCGTAGACTAATTGTTTTACAGCCCAATCATACTTCACATTTGACTTGTCCCAGTGCACTCTGGGCCAATAACCGTGGCTTTGCCACGTTTTATCGAGAATTGCGCAACAATCTTTATGGCGATCGATGTTGAGGATTAGATATGGCCAAGTCCATAAAAATGCCGTGTTCAAGCGGCGGCCGCTCAATAACAAAGTGTCTTTTACCGAGCAGCCAACATGGCACGGTGGCTGCTTAACTCGTTTTCGCTGATCCTGCTCCTGGCAGTGTGCAGCCTACAGG

>ID_121 P_121 Promoter_122 D1_Promoter for RH14988, -250bp and +50bp [6 ]

AGTTGCAATATTTTTCGCAGTTTCTTTCTTTCAATAACGGATTTAAATTTAAAAGCCAGCGTTGCCAGATAGCATGTAGCTAGAGTTACAGCTCAATGCCTAGCACAGTCATGCACCCTGGTGTGCGTCTCCGCTGTGCGTATTGTATGGCTATATTTAGTACATTTTCAGTGCCACCACAACATATGGCACAATAAATAAAGCAACTAAACAAGCAACGCGGGCTATAAAAGCGAAACGCAGCCTCGCAGATTGCAATTCAGACGCGAAGCAACAAGCAAGTGACATTGACTCCCAGCCGAAAGTGAGACTTTTGAGTAAACACACAAAGTGTAACTGCGACTAAGAAG

>ID_126 P_126 Promoter_127 D1_Promoter for RE39956, -250bp and +50bp []

GCAACGGACAGCAAATGTTATGCCATCGTCTGGCAATCTTCAAAAAAATATTGTCAGCTTGGATTGGTAGCCAATGGCGGAGTGTGTAAATCACGACCCCTTATCGCTATCGCAGCTTAATCTATAGACCAGGCGAACTTTATCTGGGCCGAAATAAAACGTTAAAGTTTGCAGATAAGCCGCTGATACTAGCAAAGTCGCAAAATGGTTTACATTCACCATATATACCGGGCCCAAAGCCAAAACTCACTTAGTTTTCGAATCGAATCGCTGGCCGAGTGGCGTGCGAAATACTAATTCTAGTCCGAGGTTCGCGATCGAACCCTTGAAAAGTGAGTTTAATAAGTGCT

>ID_128 P_128 Promoter_129 D1_Promoter for RE64788, -250bp and +50bp []

AGAAAAAAGAAGAAAAAATCGCAAAGTAGGGGAGACCACACCCCGCTGAATGGAAAATCGTCGAATCGCTGGGCAAGCGGAAAAGTCACACACGCCAGCATGAAACGCACAGAGCGGCGTTCGCATGCGCCGTCCCGCTCGCACGGCGATAGTGGCGGCCGATGGAGCGATGCGATTGGTCGCTGCCAGCGCTGCTGCTGCGCTGCCGCCTGCGCCGGTATAAAAGGACAATCATCGCCAGTAGCCGAGTCAATTAAGGCCAAAGACTCGAGCGGTGAGCGTGCGGCTCTACTCCCATATATACATATATATATATATATATATTCAACATCCTAACCGATAGAAAAGTA

>ID_130 P_130 Promoter_131 D1_Promoter for RH06505, -250bp and +50bp [7 ]

TTGCATCAGCCGCTTTGTAAATTGTATTGATTTTAAAAAAAAGATGATAATTTATTATATTTTGAAAAGCACTCATTTCCTTTAATCTCCTACACAGGCAACAGTCAATAGTTCAGCTTTGTTTTAAAACAAAGGTGTACTCAATTCCAGCCAGATCGGTTTTAAATTTGTATACATTATATATATAGATTATACAGATTATAAATCTCAGCTAATCGGCACGGCTATAAAAAGAAGGCGACGCCGATCAATCGGCTTAGAGGGAATCATAATGAAGAGCCTGACGTTATTGGCGCTTTTGATCTTGGCCACGTTGGCCTTTGTCCACGGTCGGTTTGAAGGCCAAAAGG

>ID_137 P_137 Promoter_138 D1_Promoter for RE22722, -250bp and +50bp []

ACCATCAACTCTGTCTTTTTAATAAATAGAAATATTAAAATGCCCACCAGCTGCAATGCAACTTGGTTAAAAACTAAAGCTACCAAATACTCTTACTTAAGAGCATATATTGATGTAGTTTAATAGCAAAATTCAAGTTTACATAACGCGACATTGAATAGACTTTCTTTCTCTTTAAAAATGCCGGAAACAGAGTCAGATGACTGATGAATGTCGGGTATAAAAGGCCGATGTCGCCGCTAAGATTTTCAGTTGTTCCGAAATGGCTGTTATGATCGTATTGCTGATCGGTGTTATCACCTTCGTCGCGTGGTATGTGCACCAGCATTTTAACTACTGGAAGCGCCGGG

>ID_145 P_145 Promoter_146 D1_Promoter for RH36834, -250bp and +50bp [1 ]

GGATGGGGCATCTTTGGTAGTGGATCTGTTGTAAAAATATAAAATTGAAAGTTCTTTAGTTATTTTTCTTTTTTTTTTTTAATTAAAACATTGTTGAATGCACATTTGGAGCACTCGGAAGACCCTTCTTCGCTCTCGTATTCTTCTCTGCGCATTCCTCCCTTTCCCGGTCTCTCTTCTCAATTCTTTGTTTTTTTTTGAGTGCCAACGACGTGAGCTGGTATAAATGAAGCCAAAATCGTACGCAACCGTTTAGTCCGAAACGTTTCATTAAAGCCAACGCACGTTGAAGATCGCGCGTTCTTCTCCGATCTTTCGAAATCGAGTGATTCAAGCGATCCACAGAAATA

>ID_154 P_154 Promoter_156 D1_Promoter for RE14453, -250bp and +50bp [6 ]

ACCCGCTTAGGAAATCGACGTAAAACTTCTATACTATATCCTCAAGTTATTTACAATTGTAAAAGCAGAAAAGGTACGCCTGTATAAAATTGGAACACGTAAATTAGATTTAAGAATCCCTTAAAGAAGTAACTGTTGTTTGTGACAATAGGTTGCTGGAGCTTGAACCTAGTGTGACCAAACTTAGCCGGCCAGAGATGTACCGAAAAAGTCGGCGAGAAACGTATAAAAGGAGTAGCACGTTGGTATTTTCTTCAGTTTTATACTAACTTTTGACGACATCAGATCACAAATTGCTGGGAGGTTTGCACCCCAATCAAAGCAAGTTTTACAACAAAAGTCAATTTTCG

>ID_159 P_159 Promoter_162 D1_Promoter for RH08140, -250bp and +50bp [6 6 ]

TAAATGGTTGGCAAACACACACACATGTGAAATAAATCCGGCTATTTGCAATCAATTTTCCCTTGACTTGCACTTTATACACCGGCGACAGATCAGCAGAACGAAAGGGGTGGGGAAAAAACTGGAAGCCTAGACAGCCGACAACGACGACAACGACGACGACGACGACGACTTCCTGGTGGTCAGCAGAAAATCGCTGGCAGTGCGCTATCTGGAATCTGAGCTATATAAGCCAGAGATGGGGCTGAAGGAAGCCATCAAACGTCGTTTAGCGTTTGGCCTGATCTGATTCAATTCCGGATTTGCACCAAAATGATGAGTCTAAGGATTTGCCTGCTGGCCACCTGCCT

>ID_162 P_162 Promoter_165 D1_Promoter for RE43234, -250bp and +50bp []

ACAGCAACAACACGAGCACAGCAGCAATAACAGAAGCAGCAGCAGCGACAACAACGCTAATAACAGCAACAACACTTATATCAGCATAACAGAAGCAGCAGCAGCAACGAGAACGACGGCAGCAGCAACAACATTAATAACAGCAGCAACAACAGCAGTCGCTGCGACAACAGCAACAGCAACATCGACGGTGACGGCGACTGCGACTGCGGCGGAGTATAGAAAAAGCCGCAGACGAGCGGCCGCATTTTCAGTTCAGTTTCAACGCTCTTCCAACGAGCACGCAGCAACAGCAGAGCAACAACAGCAGCAACTGTCCACGGCAGCAACAGCGTTTTGAGTTTCGATTT

>ID_164 P_164 Promoter_167 D1_Promoter for RE68314, -250bp and +50bp []

CGGCTCCCAGTATATTTTGCATTTCCTACTTGTACCGATAGCTACGATGTGAAGTGGTCCAATCCCGCCTGAGATGCTAATTCCATAACAAATGATTAGAAACGGACACTACGAACTCAAGTGCACGGCATGAATGAATCCTCTAAGTATGGGTGAGATACGATTTCATTGTTGATTGATAATAGGAAGCCTTATCAATAAATATTGTCTCAAAAATCGTATAAAAGCAGGTTGCCTCGACTAGAAGAATAAGTTTCTTCACAGCCATGAAACTGTTCCTGCTAACTCTCTCCGCGGCTTTGGCCCTGGTGGCCGCCTCCCCGACCGGTCTGAACCGCACCACCCTCCTG

>ID_174 P_174 Promoter_177 D1_Promoter for RH24992, -250bp and +50bp [5 ]

TAGCCATTTTGACAAAAATTCCTTAAGTGGCATTTTGTTGTCTTTTTACTTAAGGTAGTTAAAAAAATATTTATAAATATTTATAATAAAAATCATAATTTTCCTAAAGGCATAATCTATACCTATAATTTATTACGTATTCTAAAATATAATATTTTTACATCTTGCCTTATAAAATAAAGTAAAATAAATGTCGGCAGCACTGTTGCGCTCTCTCAGTATTTATATTTAAGCAGGGACTGTGTTTCTCATTTTAGTTGCTGTTTGGTGTTCGCGCAGTCCGCACGGCTTTTTGTGTCGCGGGAGATATCTCCGATTTTTTAACTGCCCAAACTGTTGGCATTATTACA

>ID_176 P_176 Promoter_179 D1_Promoter for RH42089, -250bp and +50bp [1 2 ]

TAGAATATTAAAGGCAATTAAATTATAACCCTGATCAAATGTCCCAGCTGTACGATCAATTGTGGCCCCAAGAAACCTGTAGCGCTGCGCAGTTTTAATCAGCGTAGAATCGGGAGAGGGAGAAAGAGACGGGGCCGGCCGCCAGAAGACTGAAGAGCGGGAGAATTGATAACGATAGCGCGGCACTCTCCACATTCGCGTAGAAAGCGGGAGAGGCGGCAGTATAAATTGGACCGACCTTTCTGCAGATGCATCAGTTCAGCTTTAAATGTTAAAAGCGACACATTAAATAAATAACAATTTTAACTAGTGAAATCATGTCTCTGACTATTACTTCCAGTGTCACTCAT

>ID_177 P_177 Promoter_180 D1_Promoter for RE08625, -250bp and +50bp []

GAAGTACTCGTTGATAGCACCAAATATAAACAATGGGTGCGAAGCACAAATGTTTGCTGTAGTCTCACCGCTCGCAAAGATTTTTCACTGAGAGCGGGCTAAGAGATAAGCGTTCTATCTAGCAAAAAAACCTGGCTGAATAAAGCTTGAAACCTGTCGAGAGCGTACATACTTTTCTTACCATATTCATACACACATATATTTGATAGCGGCGTGTATAAATTGGTGCGTCGGGTAATTGTGAAAATCAGTTTGCAGCGTACTGAACAGGTGGTGAAACGGATCTAGCCCAGAAATGGCACTCATCGAAATTTGTCTGGCCCTTGTGGTCATCGGTTATCTCATCTACA

>ID_178 P_178 Promoter_181 D1_Promoter for RE36919, -250bp and +50bp []

CTGTGGTTGTTGTTGCGGCGGCAATGGTGGTTGCTCGATATACCGATATATACCTGCCCCGGCACCCTTAAAGGCGCGCCTGTGTGCGTAGGTAGCCAACCACACCACCATACCGCCCAGGATAATGGGCGGGAAAGAGACGGCGCTAGCGCCGTGCAAGTGGGACGGGGCACAAACGCCGGCAGAGCCGTCGACAGAGGCGGCGAGCAGCGCATTCCACTTTAAATACTGCCAACGATCGCAGCAGAGAATCAGTTCACTCAGCGACTTTAGCGTGTCGAGAACGCACAGAGAAATATATCGTTTTCGACTTTGGAAAACAGAAACAGGCTCGAAAGAAGCAGAGTTCG

>ID_180 P_180 Promoter_183 D1_Promoter for RH59357, -250bp and +50bp []

AGCCCAGTCAAATATTTTAAGCGTAAAGTAACAAACCTAATTTGTATACGGCTATCGTTTTAATTCGCACACGCCTATCAACTGGATGATGTTCACACTAGAGTTACTCCCATTTCAAAGCCGGGCATTGGAAAACTAATCTCATGGCGTGCCCAGATCTCAATTGAGACTGGTTGACTGGTTGTTCAGGTGGCCGGGTGGCGATAAGCCGATCGATGGAATAAAAGGGGCCGCAATTGCCGCATCTCAAGATAGTTGCGGTCTGAATTCGCCGGAGTGAGGAGCAACATGAACTACGTGGGACTGGGACTTATCATTGTGCTGAGCTGCCTTTGGCTCGGTTCGAACGC

>ID_190 P_190 Promoter_193 D1_Promoter for RE57302, -250bp and +50bp [2 ]

CCAGACGGTGAGTACAACGTAAGCCCAAGCCCCGCAGCTGTTGTTATCGAATGAGTCATCGAGTAGTTTCTAGGCACTGAAATAATATCATAAATAATGCTTTCCATGTGATATAAAAATAAAAAATACAGCCATTTGTTATTATGTAAGCCTTTTTTAGTTCATAAATAAATTTTACATAAAAATTCGCCCAAAAATACAAAATACCAAAATAAGCTAAAAAAAAACAAACGGCCACACAAGCGATCTGTCAATGTGACAGCTGCGTCGGCGATTGTGATTTGCAATTTTTGTAGTTTGGGAATACATTAAACATTACATTTGAAATTAATTCAACACAAAGCGCAGTT

>ID_194 P_194 Promoter_197 D1_Promoter for RE50478, -250bp and +50bp []

TTACTTACTCCCGTTCGTTCGCCGGTTCACCCAGAGAGAAATCCACATAGAAGCACACACACACACACACACATGCACAGATGTGCGCGGTGTATCTGTGAGTGCCAGCGTGTTCGGTTTGACTGATAGCGGAATTGGAACTCACGCGATACAGCACGCGCTCTCTCCGGCGATTTGCGAGTGTTGGTGAGAGCGGCTGGGTCCGAGTTCGATCATATTTATAAAAGAATCGACCACCGCCAGCTACTCAAGCAGTCGCAGTCGTTCGCCAGAACGTTCAATACCCGCTCGTCCGCTGAACAACTAGTATACGCAGAGTTTTGAAAAGACCTACGGTCAGAATCAATTTC

>ID_195 P_195 Promoter_198 D1_Promoter for RH52792, -250bp and +50bp []

GAACGGGTTAGCCTCAGACGGAGACCGAGTTTGAGGTCAACGGGGGGAGGGAGTGGTGCGGGGGCGGTGCGAGGGCGGTTTCTGGCGGCTTGGATATGGTGCAGCTGGCACAGCGACATTGACGCTACTTGCTGGTAGAACGGTGACGCAGTTGCTCCGCCAGACATTGCACTCGGATCGCCGGAGATCGTATCGCATCGGAACGGAACGGATTGGTTCGGCGTATTTAAACCAGTTGTGGAAGCCGCTGGAGGGTCAGTAGAAAGTCCAGAGCAGAGCAGATCGGTAACGAGCTGAACGGAGCGTGAGCTGTGAAATTTCCTTTCTTTTCCATCCAATGTGCAATTTGC

>ID_201 P_201 Promoter_204 D1_Promoter for RE59985, -250bp and +50bp []

GTCCAATCATAACAAAGTCCAGCTGCGTATTGGAATTTAGGATTCACTTCTTGAGAACGCTATCGAAAAGATCGAAATATCACCTCACACCTGAAATGATAATATGCCAGATTATAAATATCGAATGCCGGACGAGCATTAAGAACTCATACTGCTTAAGACTGGAGTGGTCAACAAATCTTATCACCGGACAATTTGGGCTGTGATTGATGGCAAGTGCTATAAATTAGACCCGCTACACCGATCGTCAGTTAGTCGAACTCGAGTAGACTAGAAACCAAGAGAATGTTCAAGACCATCGCTGTAGTAGTGCTCCTGGCAGCCCTGGCCAGTGCCGAGCTCCATCGCGT

>ID_206 P_206 Promoter_209 D1_Promoter for RH35407, -250bp and +50bp [5 ]

CAACATTAAATGGAGGAATCAGAAAAACTTCGCAGCGTTAAGTAAATTTAGGCAATATGGGTTTAAAAATAAAAATGTATTTCTTTGTGAAAATGTATTACCATTGACAAAGTATATCTGAGCTAATACCCACACTCATTAAGTGTCGAAAGCACTGATAACCCACGTTGGACTGGAGACTAATTACCCCCAGCCGTAAATAACACATGCAACCATTTCTTCTATAAAAAGCAAAAACCATTGCGATATAGATTTAATTTTAAGCATGTCTAAAAAGCTGGTATTGTTGCTATTGTTTGTTGCCACTGTCTGTGCCCACAGGAATCGCAATCGTACGGCTCATCATGGTG

>ID_208 P_208 Promoter_211 D1_Promoter for RH04014, -250bp and +50bp [8 ]

CAGGTGTAATGGCAACTGCGTCTTTTGATCGGGTGGTCAAGCCTTATGGTGCACATTACACACATTAACAAGATCATTGTCTCTCGTAACTGTTGTTGCTGCAATTAGCGTTGGTAGCACTTGATTTATGCAGGTGCCATGCAAAGTGGCAGCAGCTTCCAAGTATAGACAGTGCCCCTGCTCACAATTAATCAGCAGTCGCAACCAGCGATCTCCGTATGACCGTATAAAAGCTGGCCTAGGGCATTGCCAACCGAGTCAGTCCCGCCGTGGAAGCTGTCGAGAACGGACTCGAAGGGATCGGAAACTAGCTCTCCAAAATGCGTGCCACAGTGGTGAGTGGAGCGACG

>ID_210 P_210 Promoter_213 D1_Promoter for RH23464, -250bp and +50bp [2 ]

AATGAGCAACCAAAACAAAACAGAACCGCGTTGAAGTCAGCTGTTTTTAAATATTTATCGAATATGCATATGATATGATATCGATTTTCAAATTGATCTTTAGTAATTGTAAATATCTTTATAGAATACTAATTAGAAGCACATTTTGGGAGAATTTCGCTTAAATATAACCTTTTTGAAATTGACGAAATCAGTAAGTCAATGTTTAAAGAAGAGCGTTTATAAGAATTACTTCTTAAGCAAATGCGTGCACACTGTAAACAATAACATTAGTGTAGAAGTGTTCTCGTTTGCGTATTTGAATACTCTTAATCTTGGGGTCTTACCAATGGCCACATGTTGCTCCCAGT

>ID_219 P_219 Promoter_222 D1_Promoter for RH03078, -250bp and +50bp [5 ]

ATTGATATTTCGATTTGATAATGTATTTAAATGTAAAATTTTAATCTAGTTTCTTGTGGCTTAAATTGTTATGCAAATCAGCTAGTTGGACAAAAATAGTTTCGTCTGGACATAAATTTTTCTTTCCGTGTGGTCGGTAATCATTTTTTCGTTCCGTGTGGTCAGTGTGAAAAGAGAGAACGACAAAAAGAGAGAAACCGCTTTTGTGTGCGTTGAGCGGGTTTTCTTAAAAGGGCGATCGACTGTTTTTGAGTCACTTGTGAAAAATCAGTCATCGCGAGCAGTTCGACTAAGGAAAACCCGCTGGCACACACAAACCGTAACCACTTATCAAACAAAAACAGCTGGCC

>ID_222 P_222 Promoter_225 D1_Promoter for RE11434, -250bp and +50bp [6 ]

GCTCAATTCAATTAGTTAGGAGCAACGATTCCGGCAATATTCGCGCTAAACGATTTCAAATTGAATGCTGAACAACCAGCGCCGGCAGAGGCTCGACAGAGCGTCGCTCTGCATCCCCGAGATTCCGATTTCGATTCCGAACCCAAATCCGAAGCCGAATCAAAGTCAGAAACAGAATCTTTTCCTGAAGCAGTACGACGTCGACTGCGCCGGAGCTCCAGTTTATAAACCCTCGTCCACCAGACGGCAGCGTTCACTTTACAATCAGCGGTCGAGCAGGCAACAAACGTGAAGAAAGTGCACATAGTACCATACCACATATATGTGCATACCATATCATCGAGTTCTGG

>ID_223 P_223 Promoter_226 D1_Promoter for RH56904, -250bp and +50bp [2 ]

GGGTGTCACTCGTTCACACGATGCCCAGTCGAGGGCATCTCCGCCGGATTCCGTCCCATCCCGTCCAGAGCGGCGGAGTGAAGTGGAGTGCCATGTGCCATGTGCTGCCCATGTAGTTCATAATTGCGCGTAATTGCCGGAGCTGCTTGAGACGCAGCTGGAGATCGGCGATGGATCCGATCTGCCAAATCAATCACGGGACTCGGCTTAGGCAATAGCTCCTATAAAACGCCGACGTTGCCGGCGATTCGCATCCAAGTCAGAGTTCGCACGTCGCGCAGTTCAATCGCAAATCGAAATGTCGCATCTGGTTCACCTGACCGTCCTGCTCCTAGTGGGCATCCTCTGCC

>ID_225 P_225 Promoter_228 D1_Promoter for RH62530, -250bp and +50bp []

TTACTCGGTTTTTAATTTTTATAATTTTTTGTAATTTCTGGAAGAATTGGGGGGTTTGTTTTCGACACGTCTGGGATTTCCGTTGACCTCAAGTTTTGGTGAAATCATCGGCGTCATATAATAATTGCCCTACGGGTTTAACTAATGATACTTGGAAAGCATATATAATGCGCTATAGAACACTTGCTCTTCACACGGGACATCCCCGGGTTATTTGCCTATTTATATAAAAGTTGGAGCCCAGGGTGTTTCGATTGCAGAAAGTGTTTGATCGTCCAATATGAAACAGTACCTGGTGCTTGCCCTCGTCTTTGCGGCCATTCTGGCCATGATCAGCGGCCATCCTCTGG

>ID_229 P_229 Promoter_232 D1_Promoter for RH21615, -250bp and +50bp [5 ]

GGATTGTGGCTGGCGTTTTGGTGGCTTGGTTTGGTTGGGTTTGGTTTCCCTTGGCTTGGCGCCCAAATAGGTCGCTTCTCTATCTCTCGAGTGGATAGGAAAGTTCCGCCGACCACTGGGCTTCCAACTCGATTGGCAGCTACGAAAATATATATATATAAAAAAAATGGAAAAAAAAAAAACGGAAAATAACACAGAAGCACCACAAACCCTCGACGAGGCTATATAAGTTGCGGTGTCGCCGATTCCCTAACCGCAGTTGTGTGACCGCGTTCGGCGGGTGAGCACGGATAGTAAGACAGTGTCTCCACTGTCAGCTTAAAACCGTTCGAAAGCCACTAGATTTGTGT

>ID_233 P_233 Promoter_236 D1_Promoter for RH50620, -250bp and +50bp [2 ]

ACATATGTGGTACGCAAGTAAGAGTGCCTGCGCATGCCCCATGTGCCCCACCAAGAGCTTTGCATCCCATACAAGTCCCCAAAGTGGAGAACCGAACCAATTCTTCGCGGGCAGAACAAAAGCTTCTGCACACGTCTCCACTCGAATTTGGAGCCGGCCGGCGTGTGCAAAAGAGGTGAATCGAACGAAAGACCCGTGTGTAAAGCCGCGTTTCCAAAATGTATAAAACCGAGAGCATCTGGCCAATGTGCATCAGTTGTGGTCAGCAGCAAAATCAAGTGAATCATCTCAGTGCAACTAAAGGCCTAAATAGCCCATACCTACCTTTTTTGTAAACAAGTGAACAAGTT

>ID_234 P_234 Promoter_237 D1_Promoter for RE60675, -250bp and +50bp [7 ]

TAAATCGAAGCGAAGCCCCAGCTCACGTTCATCAAGTATCAAATGAAAATGATTAACAAGTACAAAAAGGAATGTAAAAACCGAATTCACATACGTACATATGTAATGAGATAAGGCAGGAGTGATGTCAGAGCGTACATACATATACATATAACCTATAGTGCTACATTACTACAAAGAGAAGCAAGCTTCCATGGGGTTCTCCACACACTGCTCTCCGGGTATAAAAAGCACCCGCCCTCAAGCAGCGGACTTTCAGTTAGCATCAGCCGCGATCCGCGAGTACAAGTGCCATACCCCATCGACTCGCGAGATACATACATACATACTATCGCCAACATGTTCGCCAA

>ID_236 P_236 Promoter_239 D1_Promoter for RE18078, -250bp and +50bp [4 ]

AAATAAATGTAGTATTAAAAACAATATATTTAACTAAAAATTCTGATATAACCATATTTAAGCCATATAACTTAAAGAATCGATGGACAATCCGTACACATTGTAGGGCACTTGTATATGCCGGCCGGCAAATGATAATTCAGAGGAGGACCACAAGCAAAGTGAGAATCTGATGAACGGCAGTGCAAAACAAATCGCTGATAGCCAGTTATCGGGGCTCTATATAAATCGGCATCTGGCAGCATTTCAGTTTCAGTTTCGCGACGTCTCCCAGACAGCCGGTTTCTATTCCAGTTCTTGAAGGTGTATATATCGCTCAATATGGCCGATCCCAACGCCAAGCCCAAGTA

>ID_238 P_238 Promoter_241 D1_Promoter for RH09052, -250bp and +50bp [7 ]

ACAATGGTATATTATTTTATGCAAGCAATTAAAAGTATTTCTCTTATCATTTTGCGGCTCAACAACTTTTGCGGTATTGCACTTTTTGCTTTTTCAGAAGCTATCGACTAAACCACAACGTTTGGATTTGTGAGAAAAAAGGCTCGACCATTGCAGTTTTGCGGTTGGGACTTGATAATGAATATTTTTAAAAATATTATTTTTATATTCGGACTGCTAGTTAGCTATAAAATGAGTTCGGCTTCCTGGGAAAATCACAGTTCTGCAACTGGCTTCGTTGAGTGAAGTTAAAAGTGTAGTTGAAAGTGTACATCCATAGAGAAAACCATGACTAACACGGATCTGAAAGC

>ID_240 P_240 Promoter_243 D1_Promoter for RH08745, -250bp and +50bp []

CTTAAAAAATGCTTCTTACCAATGATTCTACCGATTTACCTGAAATTAATAATTAATAATTAAAATAATAATTTATATAAAATTCTTATAAAAAAAAAGTCTGAAATATGCGTACTTTTACCTTCTTTGAAGACTTCATCTTAAGTCTACCGATTAAATGCTTGTGTTTTCCCAAATACACGGCTTATCACCAATTCGCGTGGCTCTCTAATCCGACGCTCGACTATATAAGATCGGCTCCAACTAGCCGAGATCATCAGTTCAATCCAATCAATTGCCCAGTGCACTCAGTATCCAAAACCGGAGAAATCCATCCAGAATCAATATGAAATTCTTCTCAGTCGTCACCG

>ID_241 P_241 Promoter_244 D1_Promoter for RH69634, -250bp and +50bp []

TTTACTGATTCGAGTATCTGTTGAACTTTAAACCGAAATGGTAAAAAAAAGTGAGTCAGTTTCGGGTTATCAACAAGATCGGAGAGGGTTTAAGTAAACTAACCGGTGTATTGCCGTGTGTATGTGTAATAATTGCTTATACAGTGTGATCAGCGGAGTAGAGCGAAAACAAAAAGCGATAATCAAAACAAAACTCTGACGAAGCTGCATGCAAAATCGTATAAAAGGCAGATGCTAACAGAATAAATTCAACAGTTTCGATACCAAAGGGTTTCCAACAACATCTCTACCGAAAATGAAGTTCCTGATTGTCCTCTCCGTCATTTTGGCCATTTCGGCTGCTGTGAGTT

>ID_254 P_254 Promoter_257 D1_Promoter for RE03093, -250bp and +50bp [0 ]

TTTTGCCGCCCGCTGAGAACTAGAAACTTGTCGTGTCACACTGAGAAAGCATTGTACGATAATCTCATTGCGAGGAATCCTCCAGCCAACAGCAAACATATATACTGATCGAGCTACACTATAAGGGAATCGCTCGACTCGAGTGGATTTGTTTACGCCCGAGCCCAAAGATACGGTAATCACCCCAGCGATATGGCGAAATTGTTTGCCAACCTAACGACTATAAATCGGCAGTGGACTACGACTAGTCAGTTGCGTTGTAGACCGCTAGAAGTGCGGACATAGTAAACAGTATAGATTCAATATGATCGGAATATATTTGCTGATCGCTGCAGTCACGCTGCTATATG

>ID_255 P_255 Promoter_258 D1_Promoter for RE51323, -250bp and +50bp []

ACAACATTGACTATTTTGAGTAAAAACAATAGGTTGGTCATAAAAAATAATTACTAATTTACTAACCGCGGTCGCCGCATGTCTGAACAGGCCTCCAGCTTTTCCGGCTTGAGTATCCACTGATTCACCCGTGTCTCGAACCCAGAGTTGCCTAAACATATTTCGATGTATACATAAAGAGGGAAATTTATTTTTTAGGCTTGGCAATGCCGCTGCGGGCATATAAAAGTCCGCGCAGTCGTGCGCTTGTGTGAGTCATGGTGGAAAATTCGAGGCAAGCGTACCGATGCGCAAAGAACAAGCATAAGCGAAGAAGATAAAGATATCGATTAAAATTTCTGCTACAAAAA

>ID_265 P_265 Promoter_268 D1_Promoter for RH05865, -250bp and +50bp [5 ]

GACCGAATCCCGCTCCCACCTGCGCCTATAAATGCTAATTAACCTTTCTTCACACATCGCAGAAGCGAAGAGACGCCCGCGTACCTTGGCATTTCCGAATGGGTTCCACAAGACTGCCCCTCGAAAGCTCCGGCGCCGGCGCAGCGGTCGTGGTGAGTTGGCCAGAGGGTGAGTTGCTCGCTACCCACCATGGATTCGAGCCCGACCAAGACACCTCGGCTTGATAAATAGAACTCGTTGCTGGTGGCCAACACCTAGTGTAAGCGTGGCTGTGTATTGGAAGAGTGAGCTCCGTTCACTCCACTTCCATTCGATCCAAGCATTCCCACTCAGAGTCTGGGCAAAAGTCA

>ID_272 P_272 Promoter_275 D1_Promoter for RH47424, -250bp and +50bp [3 3 ]

CTCTTGTCAGAAGATAAGAGGCTAGGTAAGTTGATTATTCAATCAGTTTACTTACTGCAACCCAAAATGGTCACTGCACTAACCTTCAGATGAGCTGCACTACACCCTCAATCGAGAATCAATGCAAACGCAGTGCCAGCGAAAATGTCAGCAAGGGATTAGGCCAATCCCAAACGGGTAATCCCGCTGCGACAATGCTAATCCAATTCCGATGGGCCGTATAAAAGCCCCAAGCTGGGCTGGCTGTGATTTCGTCTTGGCCCGCAGACCGGAGCATGGAGTCCGGTAACGTGTCGTCGAGTCTGTTTGGCAACGTGTCCACCGCGCTGCGGCCGGAGGCGCGGCTCTCC

>ID_280 P_280 Promoter_283 D1_Promoter for RE50527, -250bp and +50bp []

TGAAAGTGAAATTGAACTGGAGAGTGGGGAAGTGCCATTTAAGAGATGATTTCTGGGCCAAGTGCGCGGCGCCTCGTCTTCCCAGGCCGGCCCACCTGCACTTCAGGTCCATCAAAAGTGCCGAACCCGAGGAGCAGTTGGTCGCACACCTGGGCCGCCAGAAGAAGCGAAAGAAACCCAAGCCAAAAACCCGGGCCGGGAACAGTTTTTCAAACGCCTTAAAAGCCCGAGACGGAGCTGCAAACGGTTTAGTTCCTCGCCGCAAACAAAACTAGTCACAACAACGTGGAGCGGAAGCCAGCAGCCCCAAAGTGAACCAGTTAACATACAGAGATACGGTCTGAGGTAAA

>ID_283 P_283 Promoter_286 D1_Promoter for RH21954, -250bp and +50bp []

ATTAAAGTTAAATTTTAATTTTTAAGAGCTTTAAAGCTTTAACTTAAAATGTAAAACAATTGAACTTTTATGCAGCTGTTTCACGCATTCCTTTGTTTCTATCTGAAGCGACTATTCAGCTGCACACCATAGTCTCGTATAGGTATGAATCTGAACGCACATCACATCAGTTTGCAATCGCATCTGGGTAAAGCCCCAAAAGCGATTTCAGCCACAAGATTTAGTATAAAAACGACAGCGGAAACTTTTACAAATCAGTTTAAGGTAGACCGGCAAGATGCTGATGTTATTCGTAGTGGAACTGCTGGTTCTACTGGCCAGTTCATCGGTCCTCTCCATCGAGGTGGACA

>ID_286 P_286 Promoter_289 D1_Promoter for RH18728, -250bp and +50bp [4 ]

AGATGTTCTGAGCAGCTCATCTCGTTGTTTTTGGATTTTGTTTTGCTGCTATATGGCTATATGGCTATATGGCTATATGGGCTGCCTTTTGCCTTTGCTGCTGCCAAAGCTTCGCTGCAGCAGCAGCTGCGGTCAGCGTCGCCGTCGCCGCTGGCGTCGCAGTCGCAGCTTTTTCAAAGCTGCACGAAATGTTGTTGGTGGGCAGCCAGCGCGGCTTCGACTATAAAACAGTTTGCTGCTGCCAATTGACTTTTGTTTTCGTTTGCGTCTGGTCTCGTCTGGTTGTCTGCTTGGACAATTCGGCGACTACAGTATAGAAACCGTGCTGTGCAGTGTTATTCCAAATAGCA

>ID_288 P_288 Promoter_291 D1_Promoter for RE54427, -250bp and +50bp []

TTACTATTGCAATTTACTGCTAGTATTTATTTACTATTACAATTTTCTACTAGTATTTATTTACTATTACAATTTTATACTATTATTTATTTACCATTACTATATAATTCAAATTATCGCGCCCAAATGGACTCTAATGCGATGAATAATCGACAAATCGATAGGCGCGAGCGAGTTCGAATTCGCAGAGAGAGATGGCAAGATATGCCGCTGTGTGTGTTTATAAGCGAGAACGAAACTTGCTTCGGCAACAATTTCAAACAAGACGTTAAAGGCGACCGTGCCTGCCAGCGACTGTCCAGGAGCACCGATTATCCTGCCAGGACTCCAGTCTCAACACACGGCTCAAA

>ID_292 P_292 Promoter_295 D1_Promoter for RH43962, -250bp and +50bp [5 ]

GAAAATCGGAGATGACGAAAAACAGCAACAGCAACGGCAATGGCAACAGCGACTAAGCAAAACATGGGCCAAACCAAAACAAAAACACACGATTTGCCGGCAAAATGCACGTGACACATCCTTTGGCGAGCTTCGTCGAGTGCGATCCTTCGTCGAGTGCCGAATGCAGCCGATTGCAGCCGAACGAAGCAGAGCCGAAGCTGCGCAGAATCCAACGAGAGGTATAAAAGCGAGACGCTCGCCGCACTCGTGGCATTCGATTGTCGTCGCTCGAGAGGATAGGTGCCCAGCTTCGAAAAGGATAAAACCCCGCAAAGGACTCTAAGTAAAAGTGAAACTTTAAAGCCAAA

>ID_294 P_294 Promoter_297 D1_Promoter for RH60072, -250bp and +50bp [4 ]

CTTATTTTTTATAGTTTAAATATATTTATATTTCATTATCGTTTGCTCGTATGTCTCCAGAGATTTCAATATCAGAACAAATTGGGTTTAGGGAAATAGTGAACAAAATAAAGCAAACAAGTGTGTTTCCAGATCAGCCATGAACATATTGCATGCATATTCGATTTTTTCTCAGTGCATTATTGCCGGCCGATTCTCGGGCAGTTCCTCTGCTCTTGGCTATAAAACTCGCACAATCTTACTCGAGCGTTCTCAGTTTTAGTTCAGACCGCTGGCGAACCGGTCACGTTCGATTGGTTGGGAAATATCGCCGGACAGAACGTAAGTTTCTCTTTCGAATTTTGAGAACT

>ID_296 P_296 Promoter_299 D1_Promoter for RE72489, -250bp and +50bp [5 ]

CCTTTAAGGGGTGTATTTTTACGATAATAAAAGTTTGTTAGATCGCATATTGTTTTAAATATTTCATATATGTTATTTTATTTTAACGAATTTATACAATTGACTTCAAAAATATCTGTGCACCATTCCATAAAATGTAAACCGAATTAATATGTTGAAAATTACTCTCGTATTAACTCTTTTTTGGGAATTGATAATTCATTTGCGATACGATATGAGTTCTATATAAGGCTTAAGCTTTGGGGCCAATACTTCAGTCGTGCGGCAAACGCAAAGCTGAAAAGTATTCTAGTTCTATAGATCTCACGGTAAGAGAAAGTATAAAGTCTAAATAAGAGAACATAAAAATG

>ID_302 P_302 Promoter_305 D1_Promoter for RE33992, -250bp and +50bp []

CTACTGACGTCACAAAGTGTTGAGTCACCATTAAAAGCATTAATTGTGCACAGTCTTAATTCCTGCCGATCCAAAACTTATTTTCAAAGTTCGAGCTATCCGGTTTGTTAAACAAAAGTACTTAATCTACTGCTCAGTTTGATTGCATACCACTGTGCGAAGGCTGTTCGCTCACGAACTATCGAACCGGTTCGCCATGCGTTCCCATCTTTAGACGGGTATTTAAGCGCGATTCGCTGCAAAAGTGGCAGTTTCGTTCCAAATTCCGAAGCGAGAGTTTGTCGTGGGAAGTTGTTGCGCGCGCCCTGTTCGCATTTATGTGTTTTTATCGTGATTCCGTCAACCGAAAT

>ID_308 P_308 Promoter_311 D1_Promoter for RE54076, -250bp and +50bp []

GGATTTGTGCAGGTCAATGATGATTTCTTTCACGACTACAGTTATCCAAAAATATGGGAAGCCTTTACTTGGCTTTATTGTAACCTTTGTTCTGCATCATATCTAGTATATGGGTGTTATGGGTACGCCTAAACAAAAACCCGTTTACGCTATCAGTTCGGCCCAAACACAGATACATATGCACACACACTAGTGCACTTGCCATGGGCGACTATTAGTATAAAAGTATGCGAACCGGACGGAGAGTTCAGTATTTGCTTGTCGTTAGAAATCGTCGGACACAAAAGCAGCGTTTTCTCCGAACGGGAATTTAAATTGAAAAGTCGGTCAGTAATAAATAACAATTGTAT

>ID_313 P_313 Promoter_316 D1_Promoter for RH44056, -250bp and +50bp [2 ]

GCAGCCAAGGAGTGCAGCGAAAACTGTGCCAGAAATGTGGACAGAAATTTCTGGCTGCCCTTTAGTGTCAAGAACTAATATTCAAAGGCGGCAAATTAACCTTGCGCTTAGATTATGTAGCGTATGAAATTAAATAAAATTGACTTTTATCTGGCAGCTGTCCGCCGTCTATAAGTCTTCTGCGCCTCTTTTGATGAGCGGCTAATTGGGTTAGCAAACATCTATAAAGGGCTGGGAATTCGGCCTCTTGGCACAGATCCACTTCGATGAGCCATCGTCTAGTCAAAGGGGTTGCGGGTAGCTGAGCATGGAGCGCAGTCATTTGCCGGAGACGCCATTCGACCTGGCCC

>ID_316 P_316 Promoter_319 D1_Promoter for RH09887, -250bp and +50bp [4 ]

AAGTTAATCGGCAAACCGCTTTTGCATAGCAAATAGAGCTGAGCAAATGCTCACATCGAATCCAAGTCTGGGTCTCATCCCAGACTTATCAGCTGGCGGGGATGCAATCAAACAGTGTGGCTAGGAACTGGACTGGAGCGATGATAAGCAACTGAACTCTTGACATACAGAGCGATAGAAATATATAGACATACATATATAGACATCCAGCGATCTGAAGCTATAAAGTCAGGCACTTGGCCGTCGGCGCCTCAGTTCAGAACCTCAACTTCGCCGCGATCGTGCAACTATAACTAACTATATCTCAATATGCTCAAGTATCTGATCGTAGCTCTGGCCCTCTGTGCTGT

>ID_321 P_321 Promoter_324 D1_Promoter for RH09431, -250bp and +50bp [4 ]

TGTATTAATCACGCCGCTAACGTCACATTTTTGCCCGCCATTAGTGGGTATGTGTGGGCAAGTCAGAATATAATAGAAGACAAAAAAAAAACCCAGGCACGACAACAACTAGCAGCCCCCTCTCTCTCTCTCTGTACCTCTCTCCCCACCTTGCCACGCCCCGCGGGGCGCTTCAAGCTACAACTCTTGTTTGCCGGCATTGTGGCGACCACAGCCGGCCCCTATAAAAGTGGGCTCGCTCTTCGAATTCCGTTCAGTGCTACTTTTCATTTGATACAGTGTACATCGCGAGTTTCACAACAGAAGAAAAAAATGTAAGTTCCCCACAGCAATTTCTATATATATTGTGT

>ID_326 P_326 Promoter_329 D1_Promoter for RE43310, -250bp and +50bp []

AGTCGATGCAAAAGCGTTCTCTTTTTTTACAGTGTTACCAGAGCCATCGGTTCCTGAAATATATCCTAAATGGATTCCGTTGTTTTTGGTATCCCAAAATGGATTCAATTTAAACGCAATACACCTGTGCATTGTTTCAAACGAAAAGAATCGTGTGCGAATGCCTCGCCGCAGAGTATCCAAGTTTAAGCCAACCGGCGGAGAGCTTATCTCTTTTGTTTTTATAAGGCATTGCGATGGAGCACCGGAGCTCAGTCTGTTTTCGTTCGTTGCCGTGTTAAGTTCACGCGATTCCACCTCTGATCCGCGGAGTTTGATTCAAATTCCGGGCTGCTGGTGGCTGGAGCGCT

>ID_332 P_332 Promoter_335 D1_Promoter for RH43482, -250bp and +50bp [2 ]

CGTTTCCTTGCGGGAGAACATTTCCAATTTTCATCCTTTTCGTGGTTTTCTCTCAAATTCGGGGTTTTCTTTTCCTCTTTTCGCGTCACGTACGCCGTTTGTTATATCCCTCTCTCTCGCGCTCTTGCGCTCTTGCGCTCTCGTGCTCTTTCGCCGGCATGAGCGCGCATGAGCGAGACGGCGGACGCAGAGATGAGTGAAACAGCTGTAGCGTCGATGAGTATAAAAGGCGGGCGCACCGGCGAGAAATTCATAGTAGCTTCGAAAAAAACACTGAACACAGTACACAAGAAAACAGACTCTCGCAGCCAGAAAATCAAATGAAGCAGCAGCAGCAAAAACGTGCATAG

>ID_333 P_333 Promoter_336 D1_Promoter for RE04871, -250bp and +50bp []

TTCGCCTTATGTTTTCCAAAGAGCCCCCTATGCTGAAGGATCTATTTTTAGGCGGATCGGCCGGTGGGCAGCATTATCGCATCCATGAGCATATTTCCTCGCTGTGTGTATTCGTTTGAGTATTTCTTTTGCCATTGGTATGGGGGCGTGGCGGGGGCGGTGGCGGCGGGGGGCATGGCGACGGCGCTTCTTGGACGAGATGGCATTTCGGCGGCGGTATAAAATCCGAGTACCAACGCGGCGATACTTTAGTCAGTCAGTTGTGTTCCAAGCCGTACACACCGTAACTCTTGATTTAATTCACCCGTGGTGCCGTAAAAAAAACAAAACTAAAACAAAATGTGTGACGA

>ID_334 P_334 Promoter_337 D1_Promoter for RE55038, -250bp and +50bp []

ATCATCGTAATCACAGGCTGAGTCTGAGTCTTCAAATCCCACGACTCCCACGGTAACAAACAAAACAGGCTGAAATCGCTCGCAGATTCGCACCAAAGATATGTGTTTGGCATATATAGGTTCAGAATGTTCTCTAGAATGACGCTCGTTTAAATTTAGATGGGAAATAAATTTTCATATTTTTAGCCCGTGGGGGAAGATGTAAACAATCTGTTGAGGGTATAAAATCGTGTGAGTGCTCGAGGAGCTCCATCAAAATCGCAGCAGCCGCGAATCGAAGCAGACGCGTTTATCCAAGTGAATTGAATCTAAAAAGGTACGAGGAAGGACCAAGCCAACGCGGAATTTAT

>ID_337 P_337 Promoter_340 D1_Promoter for RH05869, -250bp and +50bp [5 ]

AGTCGAGTGTGAGAAGTGCGAATTTGAGCGACGCCGATAATGAACTTCAAATTGCCGCCACTGGCTTTGCGAATCGAGATTCTAAAGCTGAGTAAGAAACCGGACAGGTGAAGCTCAGACTGCTGCTGGTCAGTGAAACAGTCGGCCAGCCAGCACATCTTCATTCAGATTTATGCAAAACGCAAAACAAAAAACAACAATACCAAGTAGATCTAAAAAGTTAGTATATAAGCAGTGCCTTTTGGTTACTCAGTTATTAGTAGCCCGCCAACTGGAGAATAGCGAATACACGTTGTGATACCTGGATAAACCACACATATAACTCATATTTCAAATCGAATCCACTATGA

>ID_352 P_352 Promoter_355 D1_Promoter for RH09027, -250bp and +50bp [2 ]

TTCGTTTAAAAATTCGCTTCGAATTGCGCTTCGTGTAGCGATTGTCTATATCATCGAATATGTATATGTCAATTGCTGTACATAAGAGAAGCCGAGAGAGCAATGCTAAACAGATTTTAAAAATTAGGTATATAGGTGGCTGCCGTGGTAGCACATAGTTTTGTTCTGATAAGCTCTTATTTCTTGCCGACTCATCTTCTCAAATGCTGTCTACAAAAATTTATAAATATTATTCGTCTGTAAAAATCATCCGCAGTTGAACGCAGGTTGAGCAGGAAGCTAGTCGAGACTATAATCCATATCTTGTCTGATCCTTTGTTCAAAACCACAGTGAGTATAAAATAAAGGAT

>ID_353 P_353 Promoter_356 D1_Promoter for RH70249, -250bp and +50bp [-1 ]

GTAGTTTCCAGAACACAAGAGAAAAATCCATATGTATGTGCTTAACCGGCTTACCCACCTTTCTCACGAAATGAGGGTAGTTTTTGTAGCACAGTGATGTAGACACTCGTGAGAAACCGTAGGAGCACGTATACGATGTATGTATATAGTGTACTCGCTCCAACCCCAATGCCGCAGACCCGAGAGACAGGCAGATTTTTCCCGAACCCAGCGGTTGGGTATAAATAGAGCCGCCTCAGTCCGGTCACGTCACTCTCAAATGAAAAGTGTTCAAGTGCATTCAAAGTGAAGCTGAAAAAATAACCAGTTAAAAATAGTACAAAGAAATTTTCTTTCTTGCAATTTGCAAG

>ID_357 P_357 Promoter_360 D1_Promoter for SD04538, -250bp and +50bp [6 ]

TAGTTGTTTTTAAAAGCCAATCTCCAAAAATGCAATGGATACATAATGCTAATTCAGCATATAACATGCTGAATACCTTATACTTGTAATAAAATTAAATTAAATACTTATACAATAATTTTGATTCTATTGCGGTGCCCGCTAGTGTAGAGAGAGATCTCTTTCTCTCCCTCTCTCTCTCTGTCGCACTCTCTCAACAAGTGGCTTAATACAAATATATACAAACAACTCAAAAGGCCAGCGAATGAGCCAAGTTAGTACGATTTCTGGTACCGGACTGACATGTACTAACCGCGCTTAAACAGCGGATGACGCTCGCGTGCGTTCCACAATAACAACAAAAACAGTAT

>ID_358 P_358 Promoter_361 D1_Promoter for RE46380, -250bp and +50bp [3 ]

GTATGCACTTATTTATTCGTCCAATTACAAGTAGATGGTTATTTATATTTAACAAGCTATTCACCTAAGATTAGCTTGAATACCTTATCATCACAGTTAAAGATTAATGTTAGGGAGCTAGTTTAAGTCTGCTTAATTGTCAGGAATCCCCCTGATGGCCCCTTATATCGATTCCTAAACCCAAGTTGGGGCTGTTCAGTATTTTTCCAGAGCCGGGCGGTATAAATTGCCGCTCCAGTGGCGGTCTTATTGCATTGAAGTTGGGCAATGGGAAGTGATCGCACGTTAAAAAAGCAAAAGATGTCGGTATTACAGGAACTCAAGAAAATCACCACAATTGTGGCTGACAC

>ID_362 P_362 Promoter_365 D1_Promoter for RE65433, -250bp and +50bp [1 ]

GATTAATCTGCTCATGATTGGGTTTTATTGCCAATATAGGCCAAATCGATTCACACTTCTTGGTTTGCTTGCCCTGCTTATCACTTGGAATATCGGTAGCATCGGCTCTATCAGTAGTGGCAAATAGACGATTATAAATCACCTCGGTTAACTACAGCCGGGTTAATTCACCAAAGATTATCAATGAATTACGATACCCACGCTCCTTTTAAAATCCGTATAAAAGTGCCCGAGTTCTTGGCCAACACTTTAGTTGTGATCAAAATGAAATTCGCCTGCGCTACGGTTCTGCTCTTGGCCACCATTCTTGGGGCCCAGGCTGTGGACTGGAATTCGGTCAAGAACCTGAA

>ID_367 P_367 Promoter_370 D1_Promoter for RH55123, -250bp and +50bp []

TAATTTTCTTTATCCCTGTGGCAGATTATCGCATAATAGCGGAGAGAGCATGTGAATTTATCAGTAACGACGAACTTCTAGGGATGGACATCTAGTGGCGAAATATGAAAGCTGCTGGGTGCTGTTGTCTGCGGCCAGAGGGCGCCAGCTGTCAACGGCGCACGCGTCGTATACGCGATGCAAGGCGCAGGGAATTGCGCGTGGATGCGAACGGGCAGGATATATACCAGCAGCCAGCAGTGTTGTATTTATGTGTAACTCTGAACTCTCTAGGCACGCGTCACGTCACCTCCGCGAACGAAAATCGCCAGTTTTCCTCCGTTATTTTCCCGGAAAATCCATCCTTTTTG

>ID_368 P_368 Promoter_371 D1_Promoter for RH58267, -250bp and +50bp []

GACGAATATTGTTAACAATAAAACCGGTTATCCCACATTCAAATGAAACAAGCTTTCATTCAAACAAACTAGCAAGTCATTCATTGTTTTTGTTAGATGAATTCAACCACAAATCGTAGCTAGTCGAACGCCAACAGCGGGAACGCCACCGTGCAATCTAATCAGTTCAAGCAGCTTTTAGGTGTAATTTTTGCTACAAAACCGGCTGATAGTATGAGCTGTTTATATAGAAATCTGCGATAGGTGCAATCAGTGTGAAACGAGAGTTCCGTCAACGCGGTTGTGCCAAAATGATTATCTTGTGGCTGATTCTGGCCCTAAGTGCCCTGCTCTACTGGCTCCACAGAGCC

>ID_380 P_380 Promoter_383 D1_Promoter for RH13487, -250bp and +50bp [8 ]

TCTGATCCTGGTCCTTTGTGAAGCTAGCAAAAAACCCCCCGCCCGGTGAATGCCCACTTAGATCCTTTTGCAGGAGGATTTCGACGGCCCTGAAGGTGGGCGCCACCCGTTTCGTGATGATTCACTCTGAGCAATAATACCAAAAACATAGACCTTCCCTCTCTTATTGGCTCTCCCCAATGCCTCAAGCTTTTTCGAAGTTCGATTTCACAACAGGCGGATATAAAAGGGCTGCAATGTGGGAGAGCTGTTCAGTTCAAATCACCGGCCGCATTCGCTACACTGGCTTTGTCCGCCGACTGAACCAAGATTAATTTGATCACCTAACCTCACACAGCAGCGAAAATGGC

>ID_382 P_382 Promoter_385 D1_Promoter for RH02301, -250bp and +50bp [5 ]

CCGCATTACAATATTGCAATTCATTCCGCTCGAGATTGCATTAATTAAGTAATCCACCTGTTCTAGTAAACAAATCCATGCAGGTTTAGCTTAAATATGCGATCAATAACCAGCTAGCTAAGATATTCCGATAATATGGCATTCGAAATAAACAAAATACTAGTTTGGCTTATATTTAAATTAGTACAGGATTTTCACCTGATCCACCTACCTGAGCGCCCTATAAAAAGCCTCCGACTGGCTGAGCTCAGGCCATAATCAATTTGAAGATCGTGTAAGATCGACGGAACGAAGAAATCTATATCGCGTGCGAGGAGAGGATTTGCATAAATTGAGTCTGAGTGATAATG

>ID_389 P_389 Promoter_392 D1_Promoter for RH16704, -250bp and +50bp []

CGCGATTCTCGACCCACTCAGCTGGCTGGCTAGCTGGTTAGCTGGCTGGTGAGAGGAAACCCCACGGCATCGGCATCTTGGCTTTAACCCAGCTACAGCTGGGCAACTACGTCACAGTCGCGTCATGCGAAAACCAGTCATCGTCGTTTGGTGAAGCAAACAATCGTCGGCAATCGGCCGAAACGTTCGTTAACTTGGCCGTCGGAATCGGAATCTGGGGGTATTTAAGCGGCGGCAACTCGACATGAAGCATGCATTGATTCGCTATCATTCGCCCTAAAGACTTGTGCCCAGCCTCAAGTAGTTTGCTGACTCTGTTTGTTGTAATACAATTTAGCTGACAAGGATAA

>ID_390 P_390 Promoter_393 D1_Promoter for RH38644, -250bp and +50bp []

GATTGTCTACCTGGCCGCGGGCCACAAATAGACGAAAGTACTGGCCAAGATTGCAAAAAAAAAGAACCCAGCTAGACCAAAGGAAATGGGAAAGATAGCAAACAGAAATCTAAAACCAAAACCAAAACAAAAATCAAAAAGCAAAGCCATCAGCTGGGCTTAGTTGGATCACTGCCCGGGGCCATAAAGAGAAACCGGCTTTTATTGGCAACCAAGCCGGGTATAAAAGCCGCGATCCCATCCCATGTCGGCATCAGAACTTCCCCAACGTTCTAACAAGTCAAAGTATTTCTCAACATGAACTCCTACTTCGTGATCGCTTTGAGTGCTCTTTTTGTGACTCTGGCTGT

>ID_391 P_391 Promoter_394 D1_Promoter for RH38008, -250bp and +50bp [7 ]

AATCGCTGCCTAATCGTGTTTACTTTATTCAGAACGTAGTTCCTAGATTTATTTGCTATTCCGGCATTATTTTCAGTACAAACACTAGCACTAGCACATAGTACTCGAAGTCTCGTCTGTTTATATACCAAATTCACGAGCACTCGTTAATGAACCGAAAGTTCATGAGCATATAGATAAGATCCCCAGCCAATATGTAGTTCCCATCTGCCGATCGGCATGGCGTATATAACCTAGTGCTACACCTCCGATTTCCATTAGTCGTTCCACGGAAGCTGGGAGATATACATAGTTTTCAAAATCGCAATCAGTATGAAATTCATTGCTGCCGTCTGTTTGATGTTCGCCCT

>ID_399 P_399 Promoter_402 D1_Promoter for RE70318, -250bp and +50bp []

AAGCCAAGAAGACAGCTTTACATTCGGTCAGTCAGTCAGTCAGTAGCCCATGCAGATTCCAACTCTATATATACATATCTATATACATATATAGATATATATGTATCTGCCCAGATCGGCAGACTCTAAGTTCTCTGCCGACGTCGACGGCGGGATGGAGATCGGCTTGGTTTGCATCTCGTTTCTGCGATCTGCCATCTGCGATCAGCAGCTTTGGGGCTTTAAAATCGGCTATCGACCGGCAAGCAGCTCGCAGTTTAAGTTCTCAGCTTCGCATCGACGGACGTTCGAGCACTCCACCCGCAGAAAGTTGATAGCTTCAGATTCCGACTTCGTTGCGTGCTGCTGCA

>ID_416 P_416 Promoter_419 D1_Promoter for RH52364, -250bp and +50bp [4 ]

CTACTCATACACTTACTCACACACGCACACACACACACACACACACACACAGGCTCGTGTCGGGTGCAGCACTCACTCACACCATGACGAGTGCAGAGGCGTCGGCAGAGTCGACCGAGACGGCGACTGCGATAAGATAAAGCCCCTGCGATGACATAGAGCACCCAGAGCGCTCGAGTTCTGCCGACGCTGGCAGAGATGGCGGCGCAGACGAGTAGCGCATATAAAAGCCGCTGACTCGATGGCAACGAGCATCACAAGACGGCCAACCGCGCTGCCGAGCAGACGAGTCCAGTTCGAGAGGGTACCAAAATTTAATCAGAAAGTGTAGAGTCAGTTTAGAAGCAACG

>ID_425 P_425 Promoter_428 D1_Promoter for RH06834, -250bp and +50bp []

AATTAGAAATAAACAATGCAATCTGAAACGTTGAATAACAAACTTTGGCCAAAAGTGCGTCGCATTTCGCCTTGCATTCGGGTTGCATGGCCAGCAAATAAAGGCCTGGTCGTAAGCCAATCAATTGGAAACCCTTTTGAGCAACACACACCTGATAAGCCCCAACTACTTGCAAGATAATGACCATACATATGCCAGTATAAAAGTGCTGATAAGGCATCGCTATAAATAGTGGATCCGGAGCCAAGTGAGGCCACAGAAACGAAACGAGCGCGCCAATGATCGTACGAATTCATTAGGAACAACCCAGATATAGAGACGACTCTATAGCGAAAGCAACCCAACTGTGA

>ID_433 P_433 Promoter_436 D1_Promoter for RH05303, -250bp and +50bp []

CATCCGCTGCTACCGTCCAACGTTTGCCAAGTGCACACATCGTGCTTCGGTCGGTGTCGGTGGCGTATGAGTGATGTTTGAGCACGCCCACCAAGTATCCTGCAAGGAGCGCAACATGTGGTCCCACGTCCCCCATCACCAAGTTTTGTGCGAATCCTGCGTGTATATTGCCGTCAGCCAATCAGAGGCCACCGCGGCATCTGCGCTCGTCCTCGGCGGAAGTAAATAAAAGCGGATGTCCTGGCTGGACAGGACATCATTCGAGTGCTTGCGTTCAACAAGTCCAGCAAACAGAGCAGCAGCTGAACCCCGGTGTTAACAACTAACAAGTTTGTCCATTAACTTCTTTG

>ID_434 P_434 Promoter_437 D1_Promoter for RE65325, -250bp and +50bp []

CATTGATCATTTTAAATGCATTGCGTACTATTTGAAAACCTTCGAGTATTAACAAATCGTGATGAATCCAAATGTGATCTAAATGTGATCCAAATGTCAAAGAAGATTTTTGTTGAAGCTCCTATTCTCACCACTTAAAGCGAATTCCAGCAAAGAGAGCAGCAGTGAGCATGCGCAGTGAGAGCGCAGAGAGCGCGGCAGTCTGGTACGATAGAAATTTTATTTAAGCCACAGACAACATGAAACGGGCACTATTTCTGTGGCGTCGCGTGTTCAGTTCACCGCGGGTAATTCAGAGAATCGCTTTGTGGATTGGATTTTTGCCTGTTTTCCGCCCGATACAAAAAAAA

>ID_436 P_436 Promoter_439 D1_Promoter for RH70241, -250bp and +50bp [2 ]

AGCTTCCCAATTTGAATGCCAGTCACTCATTGAATCCTATTTTTCACTTGCTCTGTGGGCGACAAATTGGTGGCGCTTCCGTTTGGGATTTGCCGCTTGATTCGCTTGTTTACGCTGACCAGCGGGCTGCCTTTTGGCCGGTATTAGAGTGCATAACAAATCACTTAGCACCCGTCCATCAGGGACCTAATCAGGCTCGCATGGGGCCGCTAGCATTTGGCTATATAACTTGCTATCTGCCGCGCTGCTGGCATCAGTTGTCCCGCAAAGCAGTCCCAAAGCGCAACGCGGTTCCATTCGATCGTCCGACTATTAAGTTAATAGTTCCAGCGCTTCCGGAGTGTCCTGTC

>ID_440 P_440 Promoter_443 D1_Promoter for RE35249, -250bp and +50bp []

CGAGAAGGAGTGAGATCCTTCAGTATACCAGTTGCCAGTGGGTTTATAGCGCTGTTTTATTATCACAATGAGCCATTATCACCTGGCGTACAGGTATTTATCAGCCAAACGAAGCTCGGCCGGTGAATCAAATTCGAGCGCTTCGTGTTTTCTGGCGCCGACGTCGCTGCCTCGTCGACTGCGAGAAAAAAAAAACTGAAACTGAAACAGAAACAGAATTATAAAAACGTTCAATTAGCCGAGGTAGTAAGTCATTACAGTGCCAACGCTCGTGGCGAAAAGAGCGAGTGAAAAACTCCAGAGAAAAGCTTTCCTTCATCGGTTGGCAGTGCGTGGAAAACTCAGCTAAC

>ID_450 P_450 Promoter_453 D1_Promoter for RH09909, -250bp and +50bp [1 ]

ATTATTTCGTAAAGATCTTGTCTTAAAACTGTGTATGATTTTTTGACCCATCAAATGGCAGTCTTAACTTATTATTCTTATTCCAAATTTCGTTCTGTGCCGCCAACGACGCAGTTGCTCTCGATCTTGATGCCTTGATAAGCGGGAGAGCGCATCATTTACCACTTGTTCAAGTGCGTTCATCGGCGAAAATTGCGGCGATAAGCTGCGATCCGGTGCGCTATAAAGTGGCCAGCAGCCCGGCAAGCGTTTCAGTCCGATCTTGGCCAGCCACCGAGAGAGACAGCCCATAAAAACCAACTCCCATCACCAAACAGTAGTAACACCCATTCTACCTGGCACAGCGGATT

>ID_451 P_451 Promoter_454 D1_Promoter for RH17752, -250bp and +50bp []

AAACAAGTAACCAAATTCTGTTTCGATTTCCTGGAGTCCTCGGACCAATCAGCTGTCGAGCTTAAAACGGAGCCGAGAGCCAAAAGCTCCAAAGCCCAAAGTGCCCCCACAATAGCCGGAGAGCAATTACAGTTAACGCAAAATGATAAAACTGTCGAGCATTGCTCGCCCTCTCTCTCTCTCTCTCTCTCTCGCGGCAGAACTTTCGATTTTTCATAATAAAAAGGTTACAACTAAATCTCAGGCGGTTTAGTCGGCAGATAAATTCGGAGCGAGCGCGCCAAATACAAAAGTCCGCCACGCGTTCAGAGCCCCAATCTCAAGTCCCATAGTTCAAACAGTTCCGAGCC

>ID_469 P_469 Promoter_472 D1_Promoter for RH67410, -250bp and +50bp []

CCCAATCCCCTGACCAGATGATCAATAAAAGCTAGCCGGAGGGCCCCGTCATCTGTGCTGTATTTTAACTGGGCTGCGATGGATGGACGAATGGGTGGTTGGCTGTGGTTGTGGTGGGGCTTGCATGATCCGCCCCAGCAATGTCATGGCTATCGCCGGTTGATCTACATCCGAGGCGATTTGCTTGTACCGGATGTGCTGGACACGATTGCGGCGGGTATAAATTGCCCGGCGCCGATTGCTGTCCGTCCACTATTCACAAGAAGAGCAAAAATGATGCAGTGCAGCCGAATGACGACGACGTTGAAGATGACGAACCTTCTGCTAGCAGTGGCCTGCGCCGCCGTGCT

>ID_472 P_472 Promoter_475 D1_Promoter for RE45038, -250bp and +50bp []

GATTAAAACTGATTAAACTATTTTCAATGAATTCCGGATTTATCAGTTCGCAGTTTCGATGATACTCTTCGAATAATTACAATTTTAGCTACGCTTAGTAAGTTTTCTAAATTTATTATTACGTCGTAAAATTTTGGTTCTTTATCAAATCGATAATACCTAGAGAAGCTTATCTGTTTAATTAAGAAAAAGACACTTGCCTGGCTGATTAGGTGGTGCTATAAAAGCTAGTCCCGCAGATGACCCAGCCAACAGTCTTCAAGATGAGGTCTTCAGTACCGGTTTGTCTAATCCTGCTCGCTCTGGCGTTGGGATCGGCGTGGGCTAATGATAATCCCTGCCAGGATGTG

>ID_474 P_474 Promoter_477 D1_Promoter for RH25931, -250bp and +50bp [2 ]

GTCGAGTGCCAATAGTTAGTAGTCAGTAGCCGGCATTAATTGAATTGCGTAGAAAACCCAGACAGGTTCTCGCTTCACGTGAATTGCACTAAATCGCATGAACAACAGACAAACAAACGAATCTCACTGATTGACGAGCGAAGAGATAAAGCCGATCTTTTAACCTGTGTAATCCCCCAGGTTGTCTTGGAATCGCCCAGATCTCGGCTCGATCGATTCACTATAAAAGAGCTCCGGCACCTTAGTGGGGCATCAGTAACTCAGCATCAGTACTACCAAGCCAACCAACAACCACCAAACCTCAGAAATCAACATGAAGTTCTTCCAAGCCGCCGCCCTTCTCTTGGCCA

>ID_477 P_477 Promoter_480 D1_Promoter for RH52894, -250bp and +50bp [2 ]

GTAGAGAGAACAGCTTGAAGCGAGAGCAGAGTTTATCTTGCTCTCGCTCTCGTACAGCCTCTCTCCACCACGCCCCCCCCCCCTACCAACCACCCCATCCACCTGTTTATCTAAAAATTTAATTTGATTTTTGTTCTTGCTCGAATTGTGTATTATTTCTTTTTTTTGGTTTTCGGTGTCGCGACTGCGCTGCCGACGTAGCCGCCGACTGAGAGTCAAGCTTAAAAGCGCAGCTTGCAAATGGACGGAGCATCATTTATTGTCGGCCAGCGACGGCGATCGTTTTATGGCGTCAACCTGTTGATCACCTCCCCCACAACCCCCTTTCATCAGTGTCTTTTTTATTGTAG

>ID_489 P_489 Promoter_492 D1_Promoter for RH38235, -250bp and +50bp []

ACTGACCACAAAATAGCCCGTCATGTCTGAGTTGTTGCCAAATTCTCGCCGAGTTGTTTGAACAGGTGCTGGCCAAGTGCTAATAGGTCGTCGAAACAAAGTATCCGAAAATCTTGCATCATCATCATTGCAGCGTCCAGAGTTCCCTGAGTTCCAAAGATAATGGCATATAGTATGCATGGGAGGAGGCAGCAGCCAGAACCCTGCGCCCAGCTCCTATATAAATAGACCCACCATACTCGATTGCGACACATTTCCAGTTTAGCCAGGCATTACAAGCACCAGAAATTTCCCACTCCCCGCGGCAGAATTCAAGTTTAAGCCCGGCTCAAGCCACTCGACCACTCAGT

>ID_493 P_493 Promoter_496 D1_Promoter for RH61755, -250bp and +50bp [6 ]

GTAAAGCTTGTAAAAATCTGTAAATTATATATAAATAAAAGCGTAGATTCTTTGTAATTTGTGAATCTAAACTTTTTTTCACGCTGACTGTCGTAACCTGACCGCCTCAGAAATGCCTTAAGCTGCGGCTAAGACGTTGGGATTATCCTTCCTGGAATGCACACGCACCCCAAGGTCAAATGGTCACCACTTAATCCGTCTTAATTAGATTCTTTGGCGGGCTATAAAAGCATTTTGACGGCACTTGCCCCAGACCATAGGTGACTCGGAGGCCAGAATGTCGACCTGCAAAGGAAACTAGATTTTGGAGCGGATCAAGCAGTATGCACATCAACGGCCCAAGTGGGCCA

>ID_501 P_501 Promoter_504 D1_Promoter for RH64195, -250bp and +50bp []

ATTATGGCAAAATAAAAATTCTTTAATTAACAGAGCGCAACTTAGCTTGTTAATATAGATTCTTGTTCATGTTTTTGTATTAAATGTAAAAATATTTATTAAAAATTGCTTTAAATGTTTTTTATCGATAGGCATAGCTATACCGATATTAGTCGATAGATATGCAGCTGGTCTTAGTTTATCGATAGTACTATCGAAATTGTCATCGCCAATTGGATTCTGGTATATATTCGCGGTCGCCGGACGGTCACACTTATTGCACTTACTCCAGTTGGAAAGGCAACAGTGTTCGCGTCGCGGAAGAAACAAGAAAGAAAAACAGCGAGAAGCACTGCAAATGCATTGGCTGC

>ID_505 P_505 Promoter_508 D1_Promoter for RH54796, -250bp and +50bp []

CACTTATACTAACACGTACCCCGCTAAGTAGGCAACACACTTTTCGGCCACTTCCGTTGCACAAACGACAGTCGACAAGCGGATATCCATATCCCCCTGCCATTGTGTGCGTGCGCAGCCGGCATTCCTGTCCACACACACACACACACACACACACCTAAACGAAGCACCCTTCGTGGACACATGGATGGCCGTTCGGGCGTCGATTCGCTTCCGTTTTGATTTATAAGCGACCGCCGCGCCGGGCTGCGTATCATTAGCAGTTGATCAACAGGTTTGCGCACAGCCAACAGCCAGGACAGGACATCCAGTTGCCACGCTCCTCGAAATCTGTTTCACACCGGTCAAGT

>ID_508 P_508 Promoter_511 D1_Promoter for RH49114, -250bp and +50bp []

TCCGCACCTGATTCGCTTTCCCGATTCGCCCTGACCCGATTCCATGACCTCGCCCACTTCTAGATATACAACTCTTAGCAATCTATTGCATCTTTATTGAGCCGCTTAATAAACAAGACATCATGGCATTCCACAATCCCACTTTTGTTCTCAATTATTGTGGCTCGGCCTCCGGGCGAGAATCTCTCGCTGATTAGGCTTATCAGCCGCCCGGGAGAAGCTTTAAATGCCAGCTCCACGGCCGGAGCACCTCAGTTCGAGCAGATCCGGTGTTGAGTGCGAAAGTGAAACCGATTCCCGAGGAGATGGGCCTGCTGCTGCTTCTGGTGCTGATAGCGGGCATTGGCGTA

>ID_514 P_514 Promoter_517 D1_Promoter for RH68992, -250bp and +50bp []

CATTTTTTGATAATGAATGTTGCCTACTCCGCAATTTCGATTTTAAGTACGCATTAAATACTAGTCATAGATTGAATTTAATAGCTCGTTTAGTATATCAATTAAGCTTGTGGTAAGCTGTGTTGCTGTTAAGCTGGAATTTCCCGCACTCACCTCTGTCTAAAAGTATACATATCACCTTTAAACCAACCACTTAGAACTGCTGATAACCCAGGGCAAGGCTATAAAAGCCCAGCTCAAGAGCCGACTGACATTCAGTCAACTGTAGCCAGAGCTAATCGAAAGAAAAATGCAATCCATTCTCCGACTGCTGGTGCTGTTTGGATTAATCTATACCGCGATCGGTGCAT

>ID_520 P_520 Promoter_523 D1_Promoter for RE24635, -250bp and +50bp []

AGCACTATCTGACTCTTTCACTCGCTATCCGTCCCACTCTGGAGCGCTGTCCAGATCGGGGGAATCTATCTGTGATCTCCGATAATATTGAAATTATTTTTCGGCAGTGACTATTGTTTGCATGCTAATTTTTATCCGAAAGCGGAGGCTAGATTAGATTATTGTTTTTTTTTTTTATTTTTTTTTGTTTGTTTGCGTCTGCCTGCCGTCTGGCCACTATAAAAACCAGGCCCTGGGGCATTGGCCATTAAGTTTGTTCAGTCACTCCGATCAAGCAGATCACCATGAAGGTCTTTGTCGTTCTCGCCGTAATCCTGGCCGTGTCCAGTGCCGCCCCGCAGTTTAATAAT

>ID_522 P_522 Promoter_525 D1_Promoter for RE38986, -250bp and +50bp []

CGGGCAAGAAAACGGTAAACAATAATTGTACTTTTCTAATTATGAAATATAAATAGAAACAAACAATTTATTGAATTTTTGTGCACTTCTTTTAATATAAGAAACGTAAGTTCGAAACCCTTCGAACTCGAACTAAATCGGTTGCATCGAACCAAGAGAGTGACCGTTCGCGAGCTCGGCTGTGATGCTAAAAATGCCAGATGGAGCAAGCAACATATACGAATATATACGAACGCATTTATCGCCGCGCGGTCAGTCGTCGAAATGTTCGAGATCCGGTCGTTTTGAAAACCAGCTCGAGCGGTCTATTCGCGTGAAATTGAAACTGAAAAAGCCGGTAAAACTGAAGC

>ID_526 P_526 Promoter_529 D1_Promoter for RH10437, -250bp and +50bp [6 ]

ACGAAATAGCCGCACCGTAAATACGCCTTCCTGACATGCGCATTAAGCGTGAGAGCTGATGGATCGCTCCACCTCCCCGCAGGAACCTTAAGAACTCGATCTCGACTCTAATTAAACTAAATACGGCACAAGAGTTTATCTTATCGGCGGATGCACCCTGTGCCGACCGGCCGTACCAGCAATGTTAACTATAGGGCAAACCGGGAGCCGAGCACTCAACTTTAAAAAACGAGACCCCATCTGGTTTCAGCAGACAGTCGTGTTTGCAACGATCTAGCGAGCGGTAGGTAGCCTCAGCGCGACTAGAATACTTGGTGGGCTAGAGAAAGGAATCCAAACCTGAATCGGAA

>ID_527 P_527 Promoter_530 D1_Promoter for RE46961, -250bp and +50bp [1 ]

GTGACGCCAAAAATGTATATATATACAAATATATATATATATATATATATATATATATATATATATATATAAGTGCATAAATTAAAACATACAAAGAGAGCGAACTGTGAATTTGTTTTGGTTTGAAATTAATAGGCCACTGTTTCGCTTCGTTGAACTTGCTTTGTGTGGGCAATGAGTAAATTTTGGGCGATTCTTCTAATCGTCATTCCCCGATAATTATATATGAAAAAGTTCTTATGTTAGATTAGTTGTCGTTCGTCAGCGAATCGCTGCGGTTGCCTTGCAAATGGGTGGAATATTTTCTGTAACTGGTCTTGGCATTCTGCTAATTGGCCTGATATGGCTGT

>ID_535 P_535 Promoter_538 D1_Promoter for RE04282, -250bp and +50bp []

AACTTACCGCCACGCACACCAACACACAGTGCGTATGCGCAATGTCACGTCTGCGGTGAAGAAGAGGGTTCCAGTTGTAAATGTAATGGTGTGTAGTTGTAATTGAGTTGTTACTGTACCCACATACGTAGTAGCCACCTTTCTTCGGGCTGCTCTGTGTGTGCGTGACTCTTTGGTTTCTCTGCCTTTTTGCATTCTTAATAAATCAACGCGAAGAATATATAAATTGGGCGGCTCGGTGGACGGTGGCAGTTAGTATTTTCCAGCCAACGGTGCCTCACAGGTGTGCAAAAGAGCCCGCAGTGCGAGTGCAAGCCTTGCGTCTTACCCCAATTATATAGCCGGGATAT

>ID_537 P_537 Promoter_540 D1_Promoter for RH57730, -250bp and +50bp []

TTATTTATAACAAATATATACAATATATATGTTGATTCGAAGAAATTGCTGTTTGCATAAAAAAATACAAAATACGCCTATATATGATATCACAGGATCAAGAAGTAGGTGGATCTGTTATCCTGGTGTCGCTGTGTAACTAAATAGCGTTGGGAAAACCCACTCCTCTTCACTTCTCTCTTACGTGCTCTTCCGAGCTTAGATTCCCAGAAAGCAGGTTCTATAAATGCTTCGATTTCAACTCTACTTGATCAGTCGACATCGCGAGTCTGTAGCTACGAGATTGTTTCTAATCGGTGTTCTCTTTGCTTTAAACTGCACTAGCACATAGGTATAAATGGTAGCAGTAT

>ID_551 P_551 Promoter_555 D1_Promoter for RE72980, -250bp and +50bp [3 4 ]

TCCGGATGATATTTGTCGCAAAAATCGGTATTTTTCCAGCAATCAGAAGACATTCGGGAGTGGGCTAATCGCTTTACGTAAAGCTTCGCTGGGAGTACGAAAACAATGACAGTTTCGGTTCGACTCGAGCACCCTGAGAACTCATGGGAGCTGTTTGCACAGCAATTCAAATGGCCACTCAAAATGGCCAGATCTCGATTGGAGCCACCTTATCGACTAGTATAAAAGGCACTGTCAGCTCTCCAGCCCGAACAAAATCGATCAAAATGCGCCCGCAATCAGCTGCGTGTCTATTACTGGCCATCGTTGGCTTCGTGGGAGCCACCGAGTGGTGGGAGAGTGGAAACTAC

>ID_552 P_552 Promoter_556 D1_Promoter for RH57685, -250bp and +50bp []

TTTTTTTTGTTAGCATCTTGTTGTGATCGCAACGCTGCTCCGCTGGCTTGTTTTTGCGGCGCATTTAATGAATGCGCCCCGTTCACCCCTCCCCTAATACTTCACACACACCACACACCGTACACCAAACGCCATACACCATACACCATACACCACACACCACCTCACCCCACCCACCAGTTTGCTGCGCAGTCGGCGTCGACGCAGCGGCATTGCGTTTAAAATGTCGCAGCGACGGCGCTGCGGAGCTCAGTCTAGCAGCAAAAGTCCAGCGATACGGTTCCAAAGCGATCTGATCTCAGTTTCGATTTGAATCCCCCGATCTGGAATTTCGAATTTTGGATTGTACT

>ID_555 P_555 Promoter_559 D1_Promoter for RH46822, -250bp and +50bp [1 ]

ATAAAATAAAAAAAAAAAAAAAGAAACCGCCGCGCAACTTAAATTGGCCATTAACGCAACTTCGACTTGCATCGTATCGAACCCGGCCGAGTGACGCAAAAATCAACTAAAAAAAAAGGTTAAGTATACGCCGTGCGGGCCGTGCCGCGACTGCGCTGCCAGCGTCGCCAGCGACGGCGGCGTCAAATGTTGGCCGGCCTGCGAAGCGCGTTCATTTTGTTTATTTATACAGCCGAGCGGGTAGAACTCCATATTAGTGTCTTCTTGGCGTTTGCCGCAGTCGAGTCCGAGTCCGAGTCCGAATCCGAATTCGAGTTTCGAGTCCGTAAACTGTAACTGAATCTTCAGGC

>ID_559 P_559 Promoter_563 D1_Promoter for RH01239, -250bp and +50bp [8 ]

CTTTTACGAGAAGCAGCGTTGCCGAGAAGCCGGCGCACTTGACGCCGATTTCCTCGGGTTATAATTAGCCAGACTTATCAATATCAACGTAGAAAAGCAGATTTACATTGTGAACGGGTTTCCGCGCACTTGGCGTGCATCTATTCGCAATTAGCACCGAATTACAAACTGGTTAACAGCCGCTGCCAACGGAGTGGAGAAGCCTGCGGTCGCCCATTTGGGTTTATAAAATGGGTCGCCACTTGGCTGAGATCAGCAGTTTGGGTCACATCTCCAAGCGGTCAGCATGAAGTCGGTTGTAAGTCAAGATCTCAGTTCTCTTGAAGATTATCCCTGAGTTTTCCATACAA

>ID_560 P_560 Promoter_564 D1_Promoter for RE74682, -250bp and +50bp []

AAGTTAAAATGATTCTTGTCTAGAAAATTGATTATATTTTTCTGTTGTATTTTACAGTTTTGGAATTTCTAGATATGTTTTAATAAATAAGGGCAGTAAATTATTCCAACTTTATATTATTATTAACCATATCAATGAATGTTGAATTAAAAATTAATATGTTAGATTAAGTAAATTCCTATTTGCGTACTTGAAATCGGTCTTAGGCAACACTGAATTTCAGGTATTTAAGCCTCGAACGTGATTGGTCACTCTAATGCCCACGTGCGTTTTGTTTGGCCTCCGCCGGCGGTAAATTCAACCAAATTACCTGAAATTCCAAGGATTCCCCGGTAAACAAGAGATCAGCG

>ID_573 P_573 Promoter_577 D1_Promoter for RH70420, -250bp and +50bp [6 ]

ATGTCAACCGGTGTGGCTAATTGTCGCGCACTTGAATTGTCGTCAATGAATTGTGGTTATTGATTCGGGAAGGTCAAGGGGTTCGGGTTGGGGTTGGGGTTGGGACTGAAGGTATTACGTGGTTTGTTCTCTGGCCCGGTTTCCGCATTTGTGCAGCAAACATACACATGGCTGATCAATCCGAGAACTGGCCTTATATGCTAAATGAAATTATGGATCGGAGCTATAAAAGCACTTGGCCAACGGGCAGATGTCAGTTAGTTTCGTTTCAAGCGTCACTGAGATCACAAAAGCCAACATGAAGGTGAGATTGAAGGGATATCGGATAACTGGAGCTTAGTTACAGCTAG

>ID_575 P_575 Promoter_579 D1_Promoter for RH09060, -250bp and +50bp []

GAAGTTCAAGCCGGGACCAATGTTTCGGGGAGTGCGCAAGGTCAGTGCAAGTTGGGAAAAAGGTGAAAGTTGAATCTCTACTTTGGCTGGCTGGCGTTGATGCTGCACCACCTGCAATCTGCAGCAGGGAGAGCGGCGAAAAAGCTCTTTGATCCTGGTCGCTGGCTGATAACAAAGTTGAGAGTGAGCGAGCTTTGGCTCGGCAGGGATATGTGCTGCGAGGATATAAAAGGATGCTGGCTGCTGGCGCACTTTCAGTTGGATTCCGAACGAGGCAGCAGCAGCACTAACAACAGCAGCTCAACCAGGAGAGCCCGAACCACCGAAGGGAAATGGACACAGTGCTGAGA

>ID_578 P_578 Promoter_582 D1_Promoter for RH55877, -250bp and +50bp [5 ]

GTTTTCTCTTTAAAAAATGTGGTTGGTATATGATATCACAATTTTTAGAATCATTCTTACAGAGAGCTACATTAATAATGCGAAATTACATAGTAATATATATAAAGAACTAAGAGCGAACCTTCTCTTCCACAGCTTATCACGAAGAGAAGCTTGCTAAGGGAGGCAAATCTATTTTGGAACTCACTCATACCTACATATGTTAAAATCACTTTATGCACTTATAAAGTGCCATTTTTCTAGGAGTTCTATTCATTCAAGCTTGTTAATTGAAGCGAAAAGGATGTCGGGATCACTGCCAGCTACTTTCGTCAAGGGTTTCCACGATGAGGAGAAGGTGCGTCGTATGG

>ID_592 P_592 Promoter_596 D1_Promoter for RH72712, -250bp and +50bp []

GGAGAGAGAGCGAGAAGGCGCAGCCCGCTCAAGTCATTTTATTGCCCTCACATATACAGGCAAAAGTATACAAAAAAACAGAAGCCACCACCACCACTACCACAACCAAGCACCACCAAGCCATCTGAAACCACCGCTGGCAACAGCAGCAGGCCGCGCACGAGGCAAAATGAGTGTCCCGCTCGCACTCACTGTCCAGTTCCCAGGGCGGCAGAGGCTATATAAAGCCGATCCCAGAGCCAGCCGACTCATTCAAAGCTCCGACTTCGTTGCGTGCACACAGAGTCTCCGAGTCCGAATCTAGTGGAACGTTGCGACTGTGGATTACGCGAGTTTACTTTACCCCCAAT

>ID_594 P_594 Promoter_598 D1_Promoter for RH28672, -250bp and +50bp []

AATTAAGTCGCGGCCAAATCGCACCCGAATCGCGTCCGAATCGCGCTAAAGTCGCCTCAAAATCCCCCCTAAATCGCATCTAATCGCGCCTAATTAAGCTCAAACGCTCTAACTAAACATAAATTGCCAAATAGAGTATGACACATTACGGCACGTCGGATATATTTTTTGCTGACCAAAAATAAATATAAATTAAATTATAGAGTATTAAAGTATCGTCATATATGTAGAACTAGTCATTAGGGATATCGCAATATCCCCATCTCTAGCTTAAACGCGATATATGTATTTAAAGTAACAGTGGTGGAAACACACCAAATTATTTCGGCAATAAAATCGAAATTACAAAC

>ID_598 P_598 Promoter_602 D1_Promoter for RH51678, -250bp and +50bp [1 ]

AACAAAATCCGATTTCATTCCAATAGTATATATGTATAGATAAGCTAAAGCAAAACCAAATGAAATACTCTTGAAATAATAAAACTGCATTTTAGAAATATGCTAAAACAACACTGCGGTTTCCATACTATATCCTTATCGCCGCAATTTGTCGACAATTTCAATTCTCAGGCGTTCTTGTGGTTTTCAAAACCGATACTAGTGCCTTCGAAGTTTTGTATAAAATGTGCAGTTCTTTCGGTGAATAGTCACAGTCGTGAAGTTCCCAGACTTCCAGTCACATTCCCCATTTAGTTTGCTCCGCGATCCAGCAGGTCCTCCCTGACATCCCATTGAAAATGGCCGACAAG

>ID_599 P_599 Promoter_603 D1_Promoter for RE69957, -250bp and +50bp []

TTGCAATTTGTTGTTGCATTAAATGAATTAAAAATAACACCCCCAAAATATAGTTTGATTTTATCATGAAACCTGCTTACGTTTTACTACCAAGATGAACGTGTCAGCATAGCCCGCTTCAATTTGAAAATTCCGCTCAATTTTTGACATTTGGCAACACTGTATTTTGGCTAAAAAAATAATCGACGAAGTCGATAGTCCCAACAAGCAATCGATGTTTAAAAAACATTGGCGAACGAGTTGCCAGACCATTGATTTTCTAGCACGTGTGATACATTCATTTAATTGTTATTTTTATGCAGCTTAACTTGCTATTTGGACCATTAAGAGAATAGGAATGCGTGACAACA

>ID_611 P_611 Promoter_616 D1_Promoter for RH64139, -250bp and +50bp []

TTGGGTTTGTTTATTCTATTAGAGATGTTTACTGTCAAATGGAGGCCCGCGTGGCAAACTTGATTTCCATTGACGTACCACTCGTTGGCGTAAACAAGAAATTTGGCACAAGTCTACTGTCTGCTCCTCCGATTTTGCATCAGAGCCCCCAAAAACTAGTTTATCTCGATCACGCTCCCCACTCGTGATGATATCACTAAGTCCGCAACGCTTTCTGATATAAAAGCCCCTCTATCGGTTCGAAAGCCAACACACTAAAGTTCGAAGGCCTAGCAGTCGATACCTAATCAGTGATTTTCAAAAATATTTTCCCCAAAGTGCATAAACAAATGTGTCAATGAAGACACCGG

>ID_620 P_620 Promoter_625 D1_Promoter for RH40504, -250bp and +50bp []

CTTCCTTAACCCTTTTCGTCCAACTTTGACCTCCAAACTCTTACCACCGTGCGTTGCACACCCCTTGTAATTAGAGGGGCTTCATTTGCGAACGAAATGGGGTAACTAAACTAAATTAGGCAGCAGCTCGATAGCGACATTGACTTTGACATTTTAAGGAACTAATGATGATGAGCGTCTTAGATTTGGGTAGTATATATGCATACATATACATATATATATATATACATCCAGTGAAACTTGCAGTGAGCACTGCTTTTCAGTTAGCTAAGCCCAACCAGTCATGTTACCATTCTGGACATCTCTACTCGTCCTTTGCGCAGCCGGAGTCCTGGCACAAAACGATTCCG

>ID_643 P_643 Promoter_650 D1_Promoter for RE30308, -250bp and +50bp []

CATAAAATGGCTTTTCTTAACAGTTTTTGTACTACTACTTTGTGGCGCATGACCTAGGACTAGGTATATTGTAGCCCCTTGATCTTGATAACGAAATCAGGCATTATCTTCAAATGGTGCAATTTTATATACGACAAATATTAACAGCGGTGGTGAATGCAATTTGATCAATTTGTTTACTGGGTGAATCCATCCACTTGACTGGCGCGCAAGTTCAATTTAAATGACTGCGGCAGCTCAATTGAGTTCCAGTTGGTTTTCATCCGCGTTGGCGAGAGGTTGTGCCATGTGTCCGATTTCCACGGCTCTTTTTGTAATTGCGGCCATCCTGGCCTTGATCTATGTCTTTC

>ID_652 P_652 Promoter_660 D1_Promoter for RE20796, -250bp and +50bp []

TGAAATAGTTTAAAATATATTTAAAAATTTATAATTGATTTAAAAGTTGATTTAACACAACTAATACTCATGTTATAGCAAGATTTAAATTTAAGCTGTATAGTATATATAATTATTTGTCTACATATAAAATATTTTCTAAAATTGAGATGATTTCGTTTCAATGGAATTTCCGCGGGTATCTTCCCAACACTGACCATCCGGTGCTTATCGATACCGATAAATGGCGTTGTTGTTGTGCTATTTGGCATTGTATCTTATTTGTTTGTAAGCAAATCATGGTGGTTTGCGGGAACATTTTGGCCAGGAACAACTTCGAGCACTTTGTTCTGGCGTGTGCTCACCAAAAT

>ID_663 P_663 Promoter_671 D1_Promoter for RH74189, -250bp and +50bp []

GGCATAGTTATAAAATGGGAACCATTTACCAATAACCGAAATAGTTCAATAAAACATTAGTTTTCTTATTAATAAAGCACAAAGAGAAATCGGCATAGTAATAAAATTGGAAGCATTTACCAAATAACCGAAAGCACATGCAATGACACCTAAGTTCCGGAATAAAAAACGCCAGATCTAGCTAGAAAATGCTGAGTTGGCAGCACAGCTCGCAGTGTTACAAAAGCTTTGCATGGAGCCGACGGGTCTTACCCTACACTACCATCAGCTGAACGCTGCTGTCATCTGTGGATTCTCAACGTTCAATAGGAAAGTTTACTAAAATAAATGAAAATACTAAATGAAACGTG

>ID_676 P_676 Promoter_685 D1_Promoter for RE34934, -250bp and +50bp []

TCTGCTAAATTACACTGCTCTTCGCCCCGGAATATTGGTATATTAGTGTAATATAGTTTCAGCGATGTACACATATGTATTATGTTTAATTTACTCAAAACTAGACAAAAACTTGTTCGCTAGATAATTAACTAAAGTTTACTCAGAAAAGCTTGTACGGCAGACAGTGAAAAGCCCACTGGTTCTACGAGCTGAATTAGGAAAGCTCCCCAAACGATATAAAACTATTGATCCTCCCTGACTGGCCTCAGTCCACTAACTGTGGAATAACGGGGCGTATACGTGCTTTCTGCTTCTGAGAACAAATCAACTATTATCTACAATGGCGTCCAGTAAAATCCCCGATTGGG

>ID_679 P_679 Promoter_688 D1_Promoter for RH09563, -250bp and +50bp []

ATCCGCGGAGACACAGATCTCAGCTGGTAGTGCCGCCCAATTGCGCAACCAATGGATCCGTGTCTCAATATTAAGTTCACTATTGCAACATCCGTGCGGTAGCGAACCAGAGGCCCCAGAAGCAATCGACACCAATCGGATCGCGGCGGATCGACTTGCGCATTTCGAGGTCATTGTGCAGACCGGGTGGTGGGTGGCGCCTCGAGTTACCCGCAGGTGGTATAAAACACCCAACGAGGAGTCACTGCAGCATCAGTTCCACAAGAGCAATCAAAAGAAGAGGATGCACTGCTTCACCTGGACGATTCTGGGCGGCCTGTTGGCCCTTACCTCAGCTGCAGGATTGCCCC

>ID_692 P_692 Promoter_701 D1_Promoter for RE26662, -250bp and +50bp []

AACATGTCAAAGAGCTTTGCAGGTAAGACAACCTATTGGCCAATGTGCAGGGGAGTTTTCAACGCAATAACAACACTATTTTATTCGCAGAGCCTCGTGGGGATTAGTCATTCCACGGGGCGATAACAGACGTCAGATGTACGTAAATTGGTGACAGAGCCAGGCGCTCCACAAGTGCAGGCATCGATAATGGGCGCAAAAATAAGGGAAAAGATACAAGCCTATAAAAGGCCGTTGCGGATGGGCAGACAGTTCAGTATTAACGACGCATTCGCAGCGAACCGATCGCAGAACCGGAACCTTAAAGCGGAAGATATAGAACTCATATAGACAGTGACATCATGTGCAAT

>ID_694 P_694 Promoter_703 D1_Promoter for RE58715, -250bp and +50bp [5 ]

AGCCATCAACGCAACCAACGAAATATGGCCCAGAGCAGGTGCTCGAGCCCTGCGAGTGACATTGAGAGCACGGCAAAGAGCGAATGCCGCGGGGAAAAGCGAACCGCAGAAGGGTGGCGCCGAAGAGAGGCAAGTGGCTTTCCACTCGTCCGGGAAATTCACTCGACAAGGACTCGTGCGTGGGCGGTGACGAGGCTCAAGTTGAGTGGCGGGAAAAGTGCTTAAAAGACGAGCGCAAACCGCTCAACGCTTCAGTCGAGTTTGAAACTTACACTCAAGCGGAGTTGTAAATAGTTGTTCGAGTTGTTGGCTCGTACTCAAACGTACTGTAACACCTTTATGCATTTAAG

>ID_697 P_697 Promoter_706 D1_Promoter for RE11283, -250bp and +50bp [7 ]

AAAGAGATCGAAACTGGTAAAACGACTTGGCAACACGACCGCGATACCAATACTCGCCCAAAGTTGACTTATTGGAGCACTTAGCTCGATTCTTGTTATTGTTACGCCGACTTGGCCGACTTGTTGTTGCCGTTAACGTTAATAGTACCAACACAATTACATAGCTTGGAGTGCCTAGGCGGAGTCGATCGGCGATCCAGCGATCCAAGGTTCCCGCTCGGACTATAAAAAGCTTGGGCGGCACCGTTGCGGAAGGCAGTTAACTCCACAAATCCGTTGCCGTCGGTCCCATTTCCCTCTTAACCGACTTGTAAGCGTAAAGGCGCGTGTGAATCCCCCTCGAGAAGAAG

>ID_701 P_701 Promoter_710 D1_Promoter for RE12642, -250bp and +50bp []

TTCACAAGAATTATTCAACGTGTGCGAAAGGTAGATAAAGAGATAAATCCGATCCCTTTTGCTGCGTTACTTGCCAATTAATATAATTGCAACCAAAGCTAAATTAATAGCAAAAAAAATTAATTGACTTGCACACGCTGTGAATACGCTATAAAAACCGACATAATCATAATCATACATCAATGGCTGATATTTTTTTTACCTCTCGGTGGGTTCTATAAATTGGGGACCCATGTCGGTTGGAGAATCAGTATCGTTTTTTGAGCTGCCGAACCAAAAATGCTTTCAAAATCGCAGCTATTACTGCTCGTTGTCGGGTTCTGCCTGGTATGATGATGTGTTATCACGCG

>ID_704 P_704 Promoter_713 D1_Promoter for RE07274, -250bp and +50bp []

TTGGGAAATGTTGACATTCAAGCTAAATACTTGAGCACACTTATTTCTATCTGTAAAACCAAACCATTTATTTGCTATCAACAGCTCAACTTTATTTAAATCGTTGGATATTATTTGTTGTATCTGTAAATCAATACCCTTTAATAGACATACCAATGAACGGGACTTGGTTCTCTTTTCAACTTTAGCGAGGCCGCACTGCCTTAACAGTAACATTTCACACTTAAAAGCTGCTGTTAAGACCGTTAGCTGTGCTGTTAGGCGCATATCTTGTTTACAAGCGGTTTAATTTTATAAATATAGCTTTGTTTTAATAAATATCCAAGAAATTTAAACTATTACACATGGAC

>ID_708 P_708 Promoter_717 D1_Promoter for RH44902, -250bp and +50bp []

TATATTTAATTAATGTATATATAATATGTTTTGTATTCCATTTGATATATAGTATGATTATGTGAAGAAAGCTTATAGAAATAGACTTAGATTTGATTGATTTGCAAGCTGAATAAGAAGATATTAATATACACCTACTCGCATTTAATATATTATCATTGTTATTAAAAATATACCTTTTCATTGCAGTTTGAATTAATAAATCCTCAACATTTTTAAAGTATTAAATATCGAAACCAGTCTTAAAACCATCGACTGTTAACTGCGCCTGCGCTGAGTTTTCGCGTAAATTCTTTTCCTCCAATGTCATCCTGCTTGTGAGAGGTCCCTAGAATCAAAAGTTAATAGTT

>ID_722 P_722 Promoter_731 D1_Promoter for RE09086, -250bp and +50bp []

TATTTCTTTATTTCTATTTATATACACAATTAAGAATAATCCCAACACTACACACGTAATTTTCAGTATATGCTTTGTCGGTGAGATTTCATTTCGTATGCTATTCAAATGCCAGCGAGTGTGCTTAAATATGTATGTGTTTTTCACTATTTTTATAACACTTGGGAGAGATGCCGAGAGAAACCCAAGAGAGAGAGAGACGAAAATAGAGAAGTGTAGATAAGATGAGAATCCGATCGTCAGCAGACTCATTTCATATACTGCGGTGGTCGGAGAAGGTTGTTCTATCAATTGAGTCCGATAATTGATGAGATATTTTGTTGCTGTAAAATTGGAAAAATGTCAGCATT

>ID_723 P_723 Promoter_732 D1_Promoter for RE02927, -250bp and +50bp [0 9 ]

AATTGAATCACATGCCGCAACTGATAGGACCCATGGAAGTACACTCTTCATGGCGATATACAAGACACACACAAGCACGAACACCCAGTTGCGGAGGAAATTCTCCGTAAATGAAAACCCAATCGGCGAACAATTCATACCCATATATGGTAAAAGTTTTGAACGCGACTTGAGAGCGGAGAGCATTGCGGCTGATAAGGTTTTAGCGCTAAGCGGGCTTTATAAAACGGGCTGCGGGACCAGTTTTCATATCACTACCGTTTGAGTTCTTGTGCTGTGTGGATACTCCTCCCGACACAAAGCCGCTCCATCAGCCAGCAGTCGTCTAATCCAGAGACACCAAACCGAAA

>ID_729 P_729 Promoter_738 D1_Promoter for RH68882, -250bp and +50bp [4 ]

AAACAGGTAAGCTGAGGTAAATAAAGGTAAGCTAGGTGGGAAAGGCCAGAGAGAGTGAAGGAGAGAGGTACGCCACGCAGCAAGCGAAAAGCTGGCTGCTACGAATACCGTTGCAATGCCGCTGCCCGCGGCGACGCAGAAGCCGACGTAAGCGCCGCTGCGCCAACGACGGCAGCGACGTTAACGTGTGGATTGTTCGCGACGTTGCGCATGCGCAGCGGTATAAAAAGCTACCGCTCGACGAAAACCACACACATTTCAAGTCGCGGTGCCAGTGGAGCGCAGTCAGTTCAGAGTTTTTGAGCTTGAATTTCAGTCTCAGTTTTTTCAAGTGCGGTTCCGTCGGTCCC

>ID_736 P_736 Promoter_745 D1_Promoter for RH01575, -250bp and +50bp []

GTGACCACAAAACATTATTGCAAAATCTCGCAATCTCAGTGCAATGCCTAGCCCTCCCATGAACATATGTAGATGCAAACCAGTTCGGTTGAACATACATATATGTATATATATATATATAATTATATATTATATAGAAACCGATCTATAAGAGCTTCGCTTCTCTGGACACGAAGTCTTATCACCATGCTGTCGGCTCCATCGATCGACTCACGTGTTGTCATTTATAAAACGATTACAATCCAAGGCATGAATGACACTCCAGTACCGGTGGCTGCACACGAAAGACACTCAAGACCCCAAGTAAAACTGGAACTGAAGCCGACCAAGCAGAACTCAACTTGAATCGA

>ID_738 P_738 Promoter_747 D1_Promoter for RE38425, -250bp and +50bp [2 ]

GACTCAAAAATTCATCATACCCTGCTTTCTCAAGGGTAAGGACATGCTCCTGATTCCGAGACCGCTTTCTCTTGGCTCTCTTTTGTCTCCCGCTCTCACTGTTTGTGCAACCACCTGCAGGCAGTCTGCCTTCCCATTGGCTGGGTCCATCGATCGAGAGAACCGCCGCTCTGTGCGAGAATTCTCCCCCGCCGAGAGAAACCAAAGAGAGCAAATCGTATATAAGGAGTCGGTCGTGCAGCAGGAAAACCAGAATACTACCAACTCTCAACCGAGTGCAACCAACTAAGATCCCAAGTTTTATTATAAATCTCAACAATCCTCAACACAATGTCGTTCATCACACGGGA

>ID_746 P_746 Promoter_755 D1_Promoter for RE02508, -250bp and +50bp []

CCCAGATCCGAGGTGGTGATCACTGGAGACGGAGAGCCGGTTTCGATCGAGAGCGGGAGATGGAGATAGAGATAGAGGTGGAGAATTGGCCGCGAAGATTAGTGGGGTCTCGCGAAGATTTCTCGGGTCTCGAGAGGAGAGAGGTGCAGATAGCATAACGAAAGCCGGCGGATAGTCTCGAATGCGCTGCCAGCGATTCGCGAGCGATGCCTATTTACTATATAATCGGGTCCCAGCAGGCGGGCGGTTGTTAGTGCTGCTCTTTCCGCGGCTCCAAATCATTCCCGCAGTGGATGGCCCCGCGATCGGTTCGTTTCGTTTCGGTCTTTGGTATCAGATCAGATCAGGGT

>ID_751 P_751 Promoter_760 D1_Promoter for RH54329, -250bp and +50bp []

ATTATAGCTTCGATGTATAATTTTTGAAATATTTTCTAGATCACTTTCAAGTATGTTTTTCTGTGCATTTCACCCTAATGTTAATGATACCCAAATGTAAGAGAGCGTAAAAATGCTGAAGAGAGTCACGAAAGAGCGCAGAGTGACCGTTTCGGCGGGCCAAAAGCGAAAGTAAAGCGTTATCGATGCGGGAGCAACTGGTCACACTGCGCCATATAAGCTATATAACTTGCTGAATCCCTGGTCTCTGCGCGATTCAGTCTTTGACTCTCCACCAATTCGGTCGCTTGCGTTTGCTCCAGCTGCGTTCTTTTCGCTCTCTTTGGCTCGCGTGAAAGTCGTAAAGTTCA

>ID_754 P_754 Promoter_763 D1_Promoter for RH48285, -250bp and +50bp []

AGTGAACCTGCGGAGAGCACAAGCGTTGCGCCGGCGTCGGTTGGCGACGCTCGGTGGCAACGCAGACGTTGACTCATCTGCTGAGACGGGCAGCCAGCCTCAGTGATTCACTTGTTCGCGTTCGCATCTTTGGCAGCTGAGGCTGCGGCTTCGGCTTCTGATGCAGGCGTCGGCTGCGGAGCTTCTGCGACGCGCTGCGGTTGGGGCTTTGCTGGGCACATATAAAACAAACGATCTCGGAGAAATATTCACAATCATATTCAAGTTGTTCGTTCGCGCGTTCGAGTTCCAGTCCAAAAAAGATTTGGATTTCCATAAGTGTAAGAAAAGAAACAGACAGGCGACAGTGA

>ID_757 P_757 Promoter_766 D1_Promoter for RE07837, -250bp and +50bp []

TTTAGTGAAATACCTACATACCTAACATATATGTTATTAAAATGTTATACAGTGCGTATAAGTGATTAGATTGTCGAATATGTTGTAGGTGAAGTGAATATGAGTAAGTGCAACGATGCGTATGAGTAATAAACTTTAAAAGAGTTTTACGCTACATATACACTAACATCAGGTTATTGTTCTCTCGCCACTCTCTCAGTGTACTCTCCCGCCAGTCGGTCAGCGTATAAGTAGAAGCGGCCACAGCGGGACGGCCTCAAAACGAACAGAGCCATCGAACGGCGAAGGTGCGCGCATTAAAACTCTCCCCCGCAAAAACCAAACAGAAAAAAGTGAAGAACGAAAACCGA

>ID_758 P_758 Promoter_767 D1_Promoter for RH53426, -250bp and +50bp []

TACTAAAATAAATAATTTCTTATGATGCAAATCAAAAGACTTAGAGAACGAACTTGGCTTCAATGGGGAAAACTCCACTCAACGTGCACGGTCTTTTGAGTTGAGAAACGAAGAGTTTGGCTCAAAGAGAGAAGCCCGAGTCATAGAGAGACCCAAAGAAGTGGCTGTTCTCTCAGCCAATCCTCTCAATGGGCGGCTTCGGCTGTGGGGTTACTTTTTGGTATAAAACATAAACAAGCCAAAGAAAGTCATTCATTTTGTTGTTCGAGCCAGACGAGTGAACCGTTAGCAGCAGCATTAGATAGAAGAAGAGCCGAGCTGCCGAGCAGTGCAACACAGTGATAAATATT

>ID_809 P_809 Promoter_818 D1_Promoter for RE49811, -250bp and +50bp []

AAAGCTCATATTCAGTATATGAGAGTATAGCATTCAAATTAAGCCTTGTTAGTTTGTTGTTGTAAATTTATTGTAAATATTTGTGGTAATACCCTCAATAAATTTTTGTTCAGCTTTTTTGCTATTAGCACCTCTATCTGGAAGTGAACAGTTAAATTTTCTTGGTTACGACTAAAAAAGAGGAGCAGGCGATAGCTAGATGGAAAGCTCTTCTTCATAAAAGCAACGCTTGGAATTGGTTTCTCAAATCAGTCTGCTCAAAATGGCGATCCTGCTCGGTTTGGTGGTCGGTGTCCTTACGCTCGTCGCTTGGTGGGTGCTCCAGAACTACACCTACTGGAAACGGCGAG

>ID_812 P_812 Promoter_821 D1_Promoter for RE36451, -250bp and +50bp []

TTGAAAAAAAAAGTTATTTCCATTAAGTTAAGATATGTTAATTAAGAATTTTCTTATTATTACCTCTAAATATTATAGATTTTCTTAACTTAATAGAAGTTAATAATTATGTAACAAACAATGTCGAAATGATGAAATTACAATCTTTTTATTTTTTTAGAGAGGAATTTTTTATTCGTTAAAAAAAACCCGATAAGCTGAAAACAAACATCGCGATTGAAGATAAATATCGGTTAAAAATATGTTATTGTGATTACAGAGGAAAACTATCGACTGAGCGCACCCAGGAGCCCATCTCCAGCACCAACAAGAAAAAATATGTATATTTATAACTATATGTAAAAAGGAAA

>ID_823 P_823 Promoter_832 D1_Promoter for RH31218, -250bp and +50bp [1 ]

GGAATTAGCCATATCACAGAAAACAAAGCCTATATTCTGCGTATTTTTCTCGCATTTTTTTTTTTTTTTGCCCGGACGAAATAACACATGAGAGAAGAAAAAGCCCAGGAAATCTCTGGCGAAAAAGAAAAACAATTCAACAGTTTGTTGCCAGCAACATGTTGCCAACGCGCTGCTGCAACACTCTCCTACGCTCTCTCCGTGTTGAGTTTTTCACTTTGATAAAAGCAAAAGACCTGGCCATAATCGAAACAGTCTGCAAAGGATTCTAGGCGCCAGCGGTTCTCCAAAGATAAAATACCGTCAAGTATTAGGCAACGGCAATCGTCGCGTGCGTTTTAACCAAATAT

>ID_824 P_824 Promoter_833 D1_Promoter for RE04976, -250bp and +50bp [6 ]

CTTAATTCCACATTCTTGTCCACCAAAGCAGACCTCTCCGCAACTCACCTCTTAGGAACTCTCCTAAATTCCGGTTTCAAGTTGTGGTCCAAGTTCCACATGGACACCAATGAGACACATTCCCCAGTCTCCACTCTCCAACCAAGTACATATCTCTCTCCAGCCGAACGAACCCGAGAGCGAAGACTCCGCCTATCGGATTCGGAAATGCCCGTTCGGGGGGTATAAAAGCGGGCGCTCTGAGCGAGCACCACTCATTCGTTGTCTGACCGCTCGCAGCAAAAGATATAGAAATCGAGAACGTAAACGCAGAAAACACGCTGGATTAAAGTGTTTCGTTTCGAACATCG

>ID_827 P_827 Promoter_836 D1_Promoter for RE67967, -250bp and +50bp [6 ]

TATGGCCAGCCAGAGTTCAATTGGTGCTTGAGACACTTTTTGACAATAGCAACTAGACAATCCAAATCTGAGTTCGGCCCCAGTAGAAGAAGCCATCGATCGATGCGGTAGTTCGCCGCTTGTTAACCGGTTTGCTCAGCGATCATCATAGCTCATCTCAGTTCATCTGATCTCGCAAAGTGAATGGACATCGGTCAGCCGAGAACTTTTCGCGCAGTTTGTGTATAAATAGTGTCGTTGGGTCTGGTTTGAAAACAGAATCAACTTTGGATTAATCTAGCAGACATCGTCTTGGATCTTCTGGAAAAATGGTTAGTTCTCCAGCAGTCATCCTGCGACCAGGACTAACC

>ID_835 P_835 Promoter_844 D1_Promoter for RH61375, -250bp and +50bp []

CTCACACTAAAAATCCTGAAAATAAAAAGTTAAATTAAAAGAAAGTATATGGTTTTTATAAAGAAAAAAGAAGTGTTCAACAGTAGGACTTCATGGAATAAACATTTTCGAGTGTAGTACTGCCGCCAAACCAAAAAAAAAGCCCCACCACTCATTCTCTCTTTTTGGGGTACGGCGCTCTCATCGAATTGAGTGGATGGATCGATGCGACTCTCGGTTCGTATATAAGGCGGGGTTCCCCAATGCTTTTGACATTCCAGTTGATCAGTGCCAGCCGATCGGTCGGTAGAGAGAAGTGCGTCGCGAGTCCAAAAAAGAAAATCGAGCCCGCAGAAAGTGCCAGTGAATAG

>ID_846 P_846 Promoter_855 D1_Promoter for RE22581, -250bp and +50bp []

ATCAATATTTAACGCATAAACATTAGAGTTTTGAACATGTGCCTAATGTTTGAAATTTGTATTCTATATTTAATGTGTTGGTACTTTTGGTAAGTAGTATAATTTAGAAATTAACTATAATTCAGAGATTCAACAGAATTATGAAAATTAGCTAAAAAGGCTTTAAGACTAGCTATATAGGCGGCACTGTTCCGATGAACTGGTTCGGTTCGTTGGCCGTACATATAAGAATTCTCAGTAGAGCAATATGGCGTTGACTTCGCGTCTGGTCACATTACTGGCATAGGTTTTTTTCGCACAACTCATAATTGTCGTGAAAATCCAGGTTTTGGCCCAAAATGACCACAATC

>ID_847 P_847 Promoter_856 D1_Promoter for RH49745, -250bp and +50bp []

ATTTTGTTTTATTTTGTTATAGATTTTTACTAAATAGACTAATTAAAGTGTATGCCAGTTGTTAAACAAATGTATTTCATAATCAGGAAGTCAAATATGTAATGTATATATATAATAAATTTGGATTTTATAGCTAAAAACTAATAAAAGGTTCACCTGTGCTCAATAATAGTTTCAAATACTGTTCAGGTCATTTTTAACACAAGAATTTATAAATATTTAAAATTAGCTATCGATAAGTGGCACCAGGTACTTACGATACTCAACCGATCTGGCAACGACGCGCGGTACGTGGCTTCGGGACACGGAAAACAATTTTACGGGAAAAGCTAAACAAATTATGTACAATT

>ID_852 P_852 Promoter_861 D1_Promoter for RE33959, -250bp and +50bp [4 ]

CGATAATAATATTCATATCGATATCGATAGACCTGCCGTGGTGAATAAGTATGTTCCCCACCCCCCAACACACTCGGTTGGCTTTCCCAACGACCTGGCAACCCTAACACGCCCGCCATTTTGCACTCTCCATGGGCCGCCATTTTGTTTAGCGGCCACGCGAGAATTCAGCATGCAAATTGGCGGGAATTCCACTCTACACACCGCCACTTGGAGACCCCTATAAAGTCGCCTACGCAGCCGCCATTTCAATCAGTTCACAATTTGCAGCGAAAGCGCCAACAGCCCCATCACGTCAAGTTCTTTAATTTAAAAAAAAGAAGAAAAACACTTTGCTAATCCTCGCGACA

>ID_864 P_864 Promoter_873 D1_Promoter for RE46851, -250bp and +50bp [3 ]

TATGAAAATTATATATGTGCAAAATAAAAAAATTAGATAGAGCACAAAAGCATTTGTGTTTCAGAAACCTGCTACTCTGTTAAGTAGTTCAAAATATCAGAGAGTTAACATAGCTAGCGAAAAGAAGAGTGACCGTGTTTGTGGCAGCGAAATATACTGCATGTAAGCAATCCTAAGAAATCAAGGATATTGTGGATCTACGAGACTGGCCACACTTTGCCATATATAAGCCAATGCAAACCGGAAACAACGCGATTTCGGTCGTCGAATTTAGTTTGAAGCGGACGCATCGTCGGGCTGGCATATATCGAAACTGCAATTGCATTTTTTTAATATTTTTACTCGTAATC

>ID_874 P_874 Promoter_883 D1_Promoter for RE38281, -250bp and +50bp [0 ]

AGTTTATTCATTGATTTTCTCTTAACTTGATGCGTTCTCTTTGTCGCTCTCTTTCCTGCTTGTTGCATGCTTCGGGCAGGTGGCTGCTCTCTTCGGCACTCTTCCCCGGCAATTCCCCCGCTTAATTTACCATTGCCTTTACCGCAACTTCGTACTCGTCTGCTCTCTCGCTTTAGCCGAAGCGTGCCGAACGAAGCCGAGCATTCGGCATGGCAGCGAATAAAAGGAAGTGCCGAAGACGCGCCGCTTCAGTCCTTCGAAGAACGCCGCGCTGAAAACATCCTTCCATTGGAAGGTGCTCTAGTGAAATCGGTGATATACTTCATCCAGTGCTGCGAGCGAACTTTCCG

>ID_891 P_891 Promoter_900 D1_Promoter for RE50274, -250bp and +50bp [0 ]

AAGCGATTATTTAAATTTGAACGCAGCGTTGGTCGCCAAGTTAGATAAGATCCCGGACTGTTCAAGCAGTGCATCTGCGCACACGGATCTGTGATTTGGCAACCGCCAATGTGAAACGTTACATATTCGAAGTTGTGAAGTCTATAATAGATTCATACGATTTATGGTAGTGCCTAATAATTTTTTCATTTATTGGGAAAACGATGCCAGCCTGGACAGATATAAAAACATAGCAATGGGAAGGCAGGGCATCATTCGACCCTAGCAGCTCAGACAGTTCAAAGTGAAAATGAAGCAGTTCGTTGTTCTAATGGTAAGCAACTGGCACATGAGCGATCCCCGCAACAACT

>ID_895 P_895 Promoter_904 D1_Promoter for RE28267, -250bp and +50bp [0 ]

CAGTGCTCAATTGTGGCAGCATGTACAGCAAGTGTTACGAGTCAACTCCCCTTGTCAATATAGCGATACACGATCGTTGGCCGGCATCCAATAAAAACAACAACAATAACGAAAAGCGAAAAATGAAAATAAAATCAAATAATATGAAAAAAAAAACTCCCTCTGCCGCAGAGACGCCGACTGCGCAGCAGCAGCAGCAGAGCGGCGCTTATCTGCTCTCATATAAATATAAACTAAAGTCGGCGGGAATAATTTCTCATTTGTAATTTGGCTGGCGGCGTGCGCGGTTCGGTTTGGAATTCGAGTTCGAATTCGGTTAGCACTTCGAATCCGATTTGGAGACAAGTTCG

>ID_909 P_909 Promoter_918 D1_Promoter for RE10126, -250bp and +50bp [4 ]

TTCAGTTCCAGCTTCTTCTGTCCCTTAAGACTCGGCACCTTGCTCTGGGTTTATGAAATTTCTTCACTTTCGGTTTCGATTCGAACTGGGGGACTCGGATATTCCGATTCCGATTCGGATTTGGATTCGGACTCGGAATGGGAATCGGTTTCGGTTTCGGTTTCGGTTTCTACAGCGGCTGCGCGGCGGCAGCGACGTCGACGTTTGGCCGGGCTATTACCTATATGAGCTGGCGTTATCATCTGAGCGGCAGCATTTTCAACTCGCGACTGACAAGCAGCGGTCGCCGTCGCCGATCCGTTGTCGAGAGATCAGATCCGAGATCTGAGTTTAAAGGAGATCCCCAAGCG

>ID_910 P_910 Promoter_919 D1_Promoter for RE60602, -250bp and +50bp []

ATAGTAGAGACGTATATCTTGTTAAATAAATTGAATTTAATAACAGTTGTATATTTGCCCTAAAGTACCTTTTTAAGGAGGTTCCACTGCGCACTGTTCCGTGTTTTCAAAACAAAACCGCAGTGAACCCAAGGCAGCGATACTCTTTTCCATATCAACCGGCGATCAGGTCGATGGCGGCAAAGGCATTATGAAATCTTGAACCTCCCAACCGATCGCTATAAATATCGCAGGTCTCGTGCTAACGACTATCAGTGGCGGAGGAAACGTGCTGGGCAGCGTACAGCCAGCTCGTAAACTGAGAAACTCTAAAACTCAGAAGAAAGTATAGAAAAATGAGGCTGCAGCTC

>ID_919 P_919 Promoter_928 D1_Promoter for RE12209, -250bp and +50bp [3 ]

ATAATGTGTAGTAAAACTTAATAATATAACGATTTTAAAGGAATTTAGAGATTATCTGCAAGATGTGCTTAATGTGGCTCAGTTGGAACAAGGTGTTAGCTCAATGGAATCTAGTGGAAATGTGCAATTGAAAGGCGGTTACCGAAGTTCCGCTCCTTATCAAAGTTCTCTTGCTGTGCTGACTTTTGCTGGCATATTTAGGGCTTGTTTTGGCGAATCGGAGGTTATAAGAGCGCTCCGCCACGGTCACACTGACTAACAGAAAGCTGTTCGACTTGAACGGACGCGTATTTCGTTGGAATATAAATTGGTGATAATTTAAAGTCGAAGGTAATGATGCTCTCTAAACT

>ID_924 P_924 Promoter_933 D1_Promoter for RH09016, -250bp and +50bp []

ATTAGCAATGCGTTTTTAAAGAACAAGAATACAATTGTTGTAATTTAACCGCATAATAAATAATCAAACAATACATAATACATACATACGTTAAAAACTAACTTACACAACTTTTATTGGTGAGAAAACAACGCTTATTTTTTGCTTAAATGTTCATGTTAAAACAAAAAAATTTACTAAATAGATATATGGCTATTAATTAACAGCATTCTGTTATCCGTTTATATAGCTATAAAGTAGCTAAATGTGCCACACTAGTGCGAAGCGGCAATTGTATTTGTTTACTTGCGGAAAAAACAGTCTCAAAACACAGAAATTAATAATAAATAATGGCTGGCGAAGTTATTGTG

>ID_930 P_930 Promoter_939 D1_Promoter for RE18076, -250bp and +50bp []

ATAACTTAAGTGCAATTCCATTTTTACCGATATTCCTGCCACTGGCGCCTTTTAAGTGAAGTGAACCAAACACAAATCACACAAATCGCAGCGATAATAGCAAAGGAACGCAAAGTGCCCCAGGATATTATGCTAGATAGTAGAAATAGAACCAGCTGATCGATGATTGGGCATTTCCGGCAGAGATTGGGCAGGTGACCTTTGTCCAGGGCTAGCTAGTATAAATAGCCCGAGAAAATTCTAAATTGGCACAGTTCAACTGAAACCCTCATCATGGTAAGTCAAACGAGAGAGCGAGTCCTTGGGTAGTACAATTGTTAAGTATTGGCCTTCCAATTTCTCCACCAGCA

>ID_935 P_935 Promoter_944 D1_Promoter for RE52275, -250bp and +50bp [1 ]

GCTCGGAGGCTGCTGAGAAAGAGAGGCAGCGCTGCGGTCAGAAAGAGAGGGCAGCTGCCGTCTTCGCAGAGTCAAAATGCCAGAAAGAGCACGCAGACACGTGTGGAAGAGGGCTCAGGCTCTTCGGCGACGGAGACCCTAGCTGTATGTGTGGGCACCGGCGCTTTTTACATAATTTTACGTTATGCCGCTGCCGAGGCGACCGCTACGTTGGAAACTCTTTAAAAACAGCCTGACAGCGGCGAAAAGTTATCAAACCGCAGCTTGTTCCATTCGAATGAACACACCAGTGATTTCGGTGGTGCCAAAGGAATTCAGTCTCAGAAATCACCAATCCACCAACCCAGCCA

>ID_944 P_944 Promoter_953 D1_Promoter for RE65765, -250bp and +50bp []

TTATTTTTGAAAACAATGCACTCAAAGAGACCGACAGTTTTGCTCAACTCTCAAAACTAAAACACTCAAGAGAGCGAGCCGCTCAACTGCTCTTAAGTGTCTTTAAGAACAGCTGATCACCGGTTGCAAAAGAGTGCCAAGTCGCTCAAGAGAGAGTTCACGGCCAAGGAAAAGGAAAATGAAAAGCTGCGACGCCGGCAGCGCAGCCGGCGTGGAAAATTGGTATAAAAGCAGCAGCACGCGAGCCGGCCGGATAGTAGCTTTGGAAAACTGTTTGGAAAACTGCTCCGCTCCGCTCAGCTCAGCTCTCCAGCGTGTCCCGTTATCGAAGTTGTTGCCGTTGCCGCAGT

>ID_948 P_948 Promoter_957 D1_Promoter for RH51742, -250bp and +50bp []

ATGCACTTTGCGGATAAGAAAGCACGCGATCATGCTGATCTGGACTACTGGGCAACCCACTGGGCACCGCCATTAGTCCCACTGTTGCATCAGCTCCGCCGATTCGATCGGAGTGCAAAATGTAGATCCCGAAGAGAGCGGAGCGTTCGCCGCGTCCGAAGAGAGCGGAAGCAGAGCGGGCAGCGCCGTTGGCGCAAGAACTGGCGTTGTCGGCGGTATATATAAAAGCTAATGGCAGCGGCGACTTCCCATTCAAAATCGCACACCTCAACGCGCCGCGTGTCGCACTTGGTCCCCCCGGAATCCAAAAAAAAAAAAAATAACCACCCCAAATATTAACCCAACCCCAC

>ID_968 P_968 Promoter_977 D1_Promoter for RE44285, -250bp and +50bp []

CAAGTGCAGCTTCGTGGCCAAAAAGGCTTCTGATTCTCGATTCCTGTGTGCAATGCAAGACCAACTTGGTCCAGTTCAATGACACTCAGTGTGCAAGAATTGGCCAAAGACGATGGCACTGATACTACCACTTTTCCCCAACCTCTTGCTCATACCTTAGCCCTGTGTGCTTGTTTGTCTGCCAACAAAGTCGACGGCGGCTGCGCAGCCGGTGGTTTGTATAAAATGCGATGGGCCTAAAGAAATTTCAAACAGTTGCGGCCGTGAGGCGCTAGTGAACACATCTTGAAATCGAAGGAGATATAGGAGATAAGTCGCGATAATGAAGCCCGTTGCCTTGATCCTGGTTT

>ID_988 P_988 Promoter_997 D1_Promoter for RE38333, -250bp and +50bp []

AACTTATGTGGCTATGTCCCCTCAGCCCCTCAGCACCAGTCAGCCGTTTGTTTGTCTTGATTTCATGGTGACGTCTTTTGCGGCGGCTTCTTCTTGGTCCGCTGGCCGCTTAAGCCTGCTCTTCTTCTTGCCCGGCGTCTGCCGTTAGTGCCGGTGTGCCACCGCTGCAGTTGGAACGGGCTCTCCAAAACCGGGGCTTTTCTTCGAAGACGGCCGGCAGCTATAAATTGACTGGCGGCTGGTAGGGACCGGCATCAGTCGCAGCCTCTAGCTCATCCAGCACAGTCGCTTGAAAACGCAGAAACCTCAACTCAAGATGGTCAGTGAAGGATTACAAGTTCAGCTTGAAA

>ID_995 P_995 Promoter_1005 D1_Promoter for RE64786, -250bp and +50bp []

ATCAGCTTCATCAGCCTCGTGGAGGGGGAGTCGCAGTCCACCCGGACAATCCAATCTGGCTGGCCAGCGTCTGGCAGCTGTGCACCGGGCTTATCACCCACTTGGTGGGGGCACTCTCATGTTTGTGTTTGCCGTTTTGTTTTGTTATAAATTATTATAAATATTATAAATTTCGCCCTGATAAGCCGAGCCGCACAGCTGAGTGGCCCTTCGGAAAGTATAAAAGCCCCTCGACGATACGTGAAACATTTAAACGTTCGTCGAGCGTCCATTGCGACTGATCATATCCACCGTTCCGAAAGATTTGTGATAACCACTTGATAACCAACCAGCCAGTCTAGTGTCTAATA

>ID_1016 P_1016 Promoter_1026 D1_Promoter for RE23174, -250bp and +50bp []

CTTAACAACGTAATTATATAAAATAAATAAAATTGGGAATTTGCTAGCATGCCAAGCATGTGATGTGTAATGTGATCCTTGGTCAAGAGATTTACATTCTCTCTTGCTTGCCCAATGCAAATTTCTCTCAGTGTACGGGCCAGCTCTCTCCGACCATCTCGCTCTCGTTCCGCCGTTTTGCTTTGCCAGTGACTTTGCTTTGCTTTGGCGTAGAGAATGCTATAAAGCAGACCGCAGCAGTGGAATAGCAGCAAGTCGACTGCTGGCCGCGTAACAGTAAACACACCAACGAGCGGACGTACACGGAGCGAAAGAAGAAGTTGGCTACCACGAAAGAGTTATTGTTTGTT

>ID_1018 P_1018 Promoter_1028 D1_Promoter for RE05230, -250bp and +50bp [6 ]

TCTCGTTTTTTTTCGCTGAAAAGCATTAAATAATTGTGAATTGTTGGTGGGGAAGGGGGGGCGTTGCAGGGGCGTTGTGGGGGCCTCAACTTCTTGGGCTTCGCTCCGCAAAGCTCGGCTTTGGCTGGTATTTCATTGTAGTTTTGTTTTTGCCCCTGCTGACCGACTGGCATTCGGCTTTTTGTGACTCGGCTCGGTTTGGGCCGCTCGGGCGTTGTCTTGTATATAAAAGCGGCTTCCTTCTTGGTTCTAGTTATCAGTTGAATCCAGCTCAACGTACAGCACACACTCGCGTCTGCAAGAAACAAAATCCCAAAAATCCAAACAATGGCATTCTTCAAAGTGAGTGA

>ID_1024 P_1024 Promoter_1034 D1_Promoter for RE40518, -250bp and +50bp [0 ]

TTTGCAAGAATAGGGCGGCCAGGTATCGATGCGCTTTGTTCACGTCGGCTTGGCCATTGTTTTATCGCACAAGCTTCGTTTTTCGAAGGTGGATAGCATTTCAAGAGACAAAAGCTGCCGAAGAAGTTACCAAAAAATGCTGCGGAGCCAACAAATACAACGATGGTAAAGTCTGCATGAACTTGAGCGCAGTTTAGTGACACCGATCGTTGGTTTTCGTATAAATATTGGACCTTTCGGTGATGCAAACGTCAGACACAACCAAGTGGTCAAAGTCAAAGAACTAAATTCAAAATGCGTGCCTTCATTGTAAGTATACGTTCGATCGCCGACGAAATACTCCAAAAGGA

>ID_1027 P_1027 Promoter_1037 D1_Promoter for RH27607, -250bp and +50bp []

TGAAAAGTTCAAACAAAAAAATCAAATTTGTAAAAGGGAAATTGGCGCCTTAATTTAAGTTTTTGCAGTGCTGGAAATTACTTGCCGCACCAACAGTTCCATTAAGGTTAGAGTCGTTGCTACACCTTATTCCACGCTCACAGCCCTGATTTCTGAACCGGTTCGTCGATACTTATCGATATCGACGTTTGAATGTTGAAACTTAATAGGCGCTGCCACTTAAGCTACAAAAAATCGATTGTCGATAATCATTTTATATCTGGTAGCACTGCCTTTAGTCACATTCATTTGTATTGAATTCTAGATAATTCCAGATAATAATAAAATTAACCCCGTTTAGTGGGCGAAAG

>ID_1044 P_1044 Promoter_1054 D1_Promoter for RH09125, -250bp and +50bp [7 ]

TTTTTTCTGAATAGCCTTCATGGTAATTTCCACGAGATATTCGGCTTCCACCAACTGTTTATTACACTTGACCTTTGAAGTGGTAGACGTATTGTACGCATATGTATATGTCGGGAAATCGGCGGATCGGTCAGCACTATCTTCGATCGGCGGCTTCGGCTGCAGCTTCGGATGCAGCTTTCTGATTTCGGTTACGGTTTCGATTTCTCGCGACGTCTACGTCTTTATAAAAATTGCGTGGGGCGCAGCTGATCCGCTCAGTGGTGCGACGGTTTCCAAGCGACTCAGATCGGCGGAGATATACACGACATATAGCCCAGTCATTGGCGATAGTCAAAATCGAAAGAAAC

>ID_1051 P_1051 Promoter_1061 D1_Promoter for RE16040, -250bp and +50bp [0 ]

ACCTTTTGTATGTTGAATATCCTTAACCTTAGATTAATGCGGGATCGCCTTGGATTAACAACAGTTTATAAAAATTATATAACTATTCGATTAAATTTTATATTTCATGCTACAAATCTTTTAAAGACAAATGTTTAGTTCTCTTCAGAATCGATTTAAGAGAAACCAGTTCAAAACTCTCTTCTCTTCGCCGCCAAATTCGCCGTCGATCGCTTCGGTTAAAAACGTGCTGCGGCAGGAACTGTAAATCATTACGTTCGGAAACGTGCTGCTGCTCGGACGGAAACGCAGAAACTGCGGATAAAAGGCGGACAATATTTTTAGAGCTCGCCAAGTTCAAGTTTGCGACG

>ID_1068 P_1068 Promoter_1078 D1_Promoter for RH54193, -250bp and +50bp []

GGTAAGGCCCCAGTCTGGTTATTTAGAGGCCCCTGTCGAGTGTCGCACAAATATGGCGAGGCCTCTTCATTCCCCTCACAATGTCCCCCAATAATCACTGAGTTGTTTGTTAGTCTACACGCATCACCACGTTATAGTACGTATACAAATGTATATGATACGATAAGCCAGCTCCCGTTCTCTGTCTCTATCGCTCCCTCTCATCAAATTGATCCAGTCGGGTATAAAAGACAGCGAGCTCTCCCAGTCAGTATTCAGTAAGCAACAAGTGTGCGAGCTAATAACAGCTACCAGCTGAGCAACTAACAAGTTTTTTTCCAAGTGAAACTAAAAATCAAACAGCAAGATGC

>ID_1071 P_1071 Promoter_1081 D1_Promoter for RE47075, -250bp and +50bp []

ACAGCCAGACAGCCGGTGGCTCAGGGCCCCGCCCAGATTTGGAATACATTTCGACCGGCCGACTCGTCTTGGGTTGTATTGGCTTGTCTTGTCTCTTTGGCCCATAAGTAACCATTTATGAATTAAGCAAACATCTGTGTTCGGGGCATCTCATCTCGTTGACTGGAGGGAGCATAACATAGTTTAGAACTACACAAACGCACACGGCTGTTGGCCGGCTATAAATTAGTGCCTCGCCTCGATTTGGGACATCAGTTGAGTTTCGTTCAGGTTGCCGGCAGCATCAGTAGCACCACTAGCATCACCAGTATCAACATGAAGCTGTACGTTTGCGTTTCGCTGGCCCTTTT

>ID_1089 P_1089 Promoter_1099 D1_Promoter for RE35848, -250bp and +50bp []

TGTTCAATTTTTTGTTCACAATCCACATGGTCCACCAACGGTCCACATCCGTTAATTCATATTTATGGCCAGGAAATGCTTTCTTTCTATAAATACAACCCCTTTCGAAAAGAATGTTTTTAAATAAAATTATTTCGCGCCACATAATCCACGCTCTCTTAGCACTCTCTTCGCGCTCTCTTTCCTTTTCTCTTCGCCGGCCGACAAGACAATCCAACCATAAAACCTGAAAACCAAAATCCGCTGCCCATTTCTCGTTTGGCTGTTGCCTCGAATGGACGGTTAACGGAAGTTTGCTATAAAGTTGAAAAATATAGAGAAAACCAAAATATATCAGAACCCAAAGCAAT

>ID_1098 P_1098 Promoter_1108 D1_Promoter for AT14067, -250bp and +50bp []

GTAGCATTTAAAATTTGCAATTTTTAAATTATATTAAAGCCATTGACAAACATATAAAGTAATTTGGCTGATTAAGTGATAAATAAGTACAGTTCCAGAAAACAAATCAACCTAGCAATACAGATTCACTCTGATTCCGAACCAACTCAAATCACAGTTTCCCAATACAGTGACTTCGATTGAAAGGAATAACCCTATGTGACAGCTGTCACACGTGCCAGTGATAAAAAATCCCGCACACCTTCCAACGGAACGCGTTAGTGTGTCGTTTTTTCGTAGCAGGCTATGTCGTTGGGCAACGCAGGGCGAATCGCTGTCTGTTGGTTTGTAGAAAAATCGTTTTTAGTTAC

>ID_1102 P_1102 Promoter_1112 D1_Promoter for RH26422, -250bp and +50bp []

CGATAATCGCGGCGAGTGGCGGCGGCAAAGGAAGTGCATCGACGAGCGGTATTTTCTGGGAGCTTTTCCGCTGGCCCGTGGAGCCCGGGGTGGCGACTGGCCGACGTGACCGGCAGCACATCGGACATCCCATGGGCAAAGCTTCCCGCGGGTTCTGGTTCTGCCTCTGCCGGCTCTAGTTGGGGCTCCCACTCTGGGACTACGTTTGCGATTGTGTATATAAGGCCCCATGCAGGGCGTGCGTCCCGCGCATAGTGCAAAAAGCTCTACTCGCGTTCACTTCGCATCCGCTCGCCGCATTCCCGTCCGCACCCGGAGTAGTGTCCAGTCACTCGTCACTCGTCCCGATT

>ID_1106 P_1106 Promoter_1116 D1_Promoter for RE74956, -250bp and +50bp []

AAAAACGGAAACCAGCCGAAAACAAGCTGGGTTTTCCCAGAGCATTTTCCTGCGAAGGGTCGGTTAAAAAACTAGGGGATGAAGGTCGAACCACCACCACCAACGGACTTCTCATTTTATTCGTTTTTTAAGAGAGCGAGAGCGGCGAGATCGGTTTTTTAAGAGAACCGGTGGCGAGAGGGTTGCACTTGGGCATGGATTTGCGCAATTTTTGCTATATTAGCCGGAGGCCGCGGAAGAGTTGAAGCAGTTTGAGCCTCGCAGCCGAACTTTGAGGATCGCTGAGACGAGACGCCGTGCAAGAAAGTCGATCCAAAATCCGAAATTCGAAATCCGTGCTGTGATCTGTG

>ID_1113 P_1113 Promoter_1123 D1_Promoter for RH57909, -250bp and +50bp [6 ]

TGGCTTAGTTCGAGCTCATCTCTCCGGCGCTTGGCCAAATAAATTAACGAATCCGGCGAAAAAAGCAAACTCGTTCCACTCACACTCAGACACACACACACACACACTCACGCACTTTAAGCCAGGTATCTGTATGGGTTAGGAGCTGCTCTCAAGTACGATTTTTAGTCTTTTTTTTGTGTCGAGTGTGTTTTTTCGCCTTTTTGTTTTGCGTCTGCCTCTGTTTATATGGCTAAGACTCGATGCGTTCATCTTCAGTTCGTCCACATCGGTCACCACGGTATGAGCTCTAGTTTAGGCTCCTAAAAGTAAATACTAAAACACTACCCAATCACTTGTGATCGATCAAT

>ID_1126 P_1126 Promoter_1136 D1_Promoter for RE58827, -250bp and +50bp [1 ]

TTAATCTAGATCTTCGAAGTTTCTGACTTTGTCATCGGCCATTGCAATTTGTTTGTTTTTTAATAGCTTTTCCTTATAATAACATTTATGCAAAGCTGAATTGCTCTGCGAGCCCGAACAAAAGCCGTTCGCTGCCTACTTTGTAGGCGGACAAAACATTTAGCAGCATCAGCGCTTTGTCTTCGAAAAACTGCGCCGCCAGCTTCGAAGTTGGGTTAGTATATATAGTTACCGCCGCCGTGGTCATAGGCAGAAGCAATTTGTGCTCCGAACCAAGAGAACCTCAAACCCAAAACTAAAGATGCGCGCCTTCATCGTAAGTGGTGTAGCCAGGACTTAAGACGAATTCA

>ID_1127 P_1127 Promoter_1137 D1_Promoter for RE15263, -250bp and +50bp [-1 ]

CCTAATGATGTTCGTGTATATTTTGTTGCATAAATTATCATAATTGTCACTGCGCCGACTGGGTGATGGCATTTTTCCTTTTGATCGCTTAGCATTTCCCTTTCGATGGTGGGTTAAGAACAGTTATAGAGATGCGGGAAATGTGTTTTCTGTTCGATCTAGAGATTATTTTGTATATTTTCTATGCATATTAAAACGTAGCTCTCGAGTTTTTCAGCTTATAAAAGCCGAATTCGAATTTTGGATCTCAGTCCGATCTGAAGAGAAATCCGAAGTACATCATGAGTTTTTTGGGACTCTTGGCTTTTCTGGTTGCTGGTAAGTTTATAAAACATTTATAAATTTTTCTA

>ID_1130 P_1130 Promoter_1140 D1_Promoter for RE38514, -250bp and +50bp [5 ]

TTATATTTTATATATACCTGTCTCATATTTATTGTTTTATATAGTTGTAGCTGATCTTAAAATGGTAACAACTTTGTATGCTAAAATAACCTTCTTTCTAGAGACAACTCACAAGTGTGAAACTATCGCCGTCTTCCAACATGTTCGACTTCGAGCCTCGGCGGCTGCATTTCTTCGGTTCGATTCCAGGCCCATCGCGAAGACGGTAAAATGGGTCAGGCTATAAAAGCTGAAGTTCAAGGCGATCGCCGTTTCAGACAACTTTAGCAGCCGTCACAGCAACACCTCCATTTAAAGCCAGTAACCACCAGCTGTAGTGCCCAGTTAAAGCCGAACACTCTCGAATCGAA

>ID_1135 P_1135 Promoter_1145 D1_Promoter for AT24624, -250bp and +50bp []

TATAATTCTAATAATTGTTTACAATTAAAGCTTTAAACAATTAAAGCCCCTCATTTTATTAATATCCTTTGCTGAACGAAGGAATTGAATTGAATTTTATTGAACTCAATAAATAAATATAGTTTTAGAAAACGATGTACTTTAAACTGCCAAGAGGTCACCGCGACAAGCTTTTCACTTCGCCACCCTCTGGTAACACTGATTCTCCAGCCACTTTCGATTCTTTAAAAAACATCGATAGGTATCGCCAAAAAAATCAGCTGCTCTGCTTGTAGTTTTGCGATTTCATTGCAATTTTAATTTGCTTTACTGTTCAAAAAGAAATACAGTGCAAAATATGCCGAACGAAA

>ID_1138 P_1138 Promoter_1148 D1_Promoter for RH06401, -250bp and +50bp [1 ]

GCGTATACGCGATGTTCCCAGACAGCGCACATTGCGTATCCGCCGAGTGCGCCGACGTACTTGAGACTTTTTTAAACCAGACAAGTTGCTGGTGAAGCCAGCCGCTTGCCGCATTGTGCCGACATGAAGTGCAGTTCTTAACCTTCCTTTGGGAGCTCCAGCTTGGAGTTGAAGGGATGGCAGGCTGCTGACTCAGCCGAAGTTTCCGCCTTTCGGAGCTATAAAAAGGTGCCTCGATTCTGGGTTAGTTCATCAGTAATATTGAGCCTCCAGCAGTGATTACACAGCGGTTCTAAGTCGGTCTAACAATCTTTTTTTTTACTATATTTTTTTGAGTGACTAAAGAGTTC

>ID_1148 P_1148 Promoter_1158 D1_Promoter for RH54219, -250bp and +50bp [5 ]

GCATTGCCATATAGTCAAAATGTTGCGGGGAACACCTTTTTCGTTTGCTGATGCATTTCTACATATGATCATATTTCTTATTTTATGTTGATGTTATCTTATGTTACGATCGAACAAAATAAATCCGATTTGTTTGCATATCATTCGACTTGTTGCAGCTATGACCGATAATAACCGATGCCGACCGATAACATGCAAATCTCGCTGACCGATAACATGGGTATATTTAAACTGGCCCTGAATGGTCGCACATTTGTTGCTTCAGCTTCACGTTGAGTCGCGTTTATTTTTGTTTTCCTCCTTGGTTCTTGTTTTTTTTTTCTCTTTTTTAAAAACACTGCAATCCTTCA

>ID_1150 P_1150 Promoter_1160 D1_Promoter for RE40068, -250bp and +50bp [0 ]

TGGTACTGAATTAGTATTAGTATTCTATTCAATTCCGGATTTTTCTGTTTTTCCATTTTCCCTCTTCCAATAAACTGGTTTATTTTATTTTCCATCGTAACCAGATGTTGACGGCAATACTTTTCTGTGATTTCCTTTTACAGGTAGAGACTCAAACTTAAGTCCCTCAGCCAGGTAGAATACCTTTCAGCTACCTACTTGATATTACCAAATCGTGGTATAAAAATCAAAAAAATATTAGAGCCGCCTCAGTACCGAAACTTCAGCCACGATGAAACTACTGGTGCGTCTTCTACTATAGAAATCTTAACTAACTTAGAAATATTTTTATAATAACAAATTTATAACTA

>ID_1151 P_1151 Promoter_1161 D1_Promoter for RH55750, -250bp and +50bp []

GAAATATGTTTGAGGTTACGTTCGGCAATGAAATTATGTAACATTAAAAACACGTGATCAATGGAAGTAATTTCCCAAAAACCCATGAATGCGATTTGGTTAAAAATAGCTCAGATGACGTCACATTTATCGTGTGGTTTGTATGAGTATGTGACTGATAAAGAAAGGCAAATGTTTACAGTCCGAAAGTCGCGATTAGCTTGGCGCCAAAGTTGCCTATAAAAGCTCATCGACCGAATGCCGCAGAAGCCAGTCAAATTCAAGTGTTCAAGTGAAGCAGTCGAAACATAAGCAACAGATTCCGCAGCAGTTATATCCAGTTCAGAGAAACAGTAGTTAATCAGTAATTA

>ID_1153 P_1153 Promoter_1163 D1_Promoter for RE26656, -250bp and +50bp [7 ]

ATGCTTTTCCAGCTGCTGGGGTACCGCTGCAGCGCGGCCAACGCTAACGGCGACGTCGATGCCGACTGCGCATTACCGTTGGCCGGCCAAACGAAAGACTTAAGCGAGAAACTTGCACGACTAGTTTAGGGGAACCGCTTCTGGGGGCCAGGGGGCAGGTGGGTCGGAGAAATGCTTTCGCAGCAGATGAGCATGCCGATGCCGAAGTCACTGCCACTGCCGCCGTATATAAATTGTCGTGTGCGGTGGAAAATTGTATCATTCAATCCTTAACCGTCTGAGCAGCTATACGGCAGCAGCAGTCAGCCTAGACCGAAATACACAAAAATACAGTTTTCCATTTAAGAGAA

>ID_1154 P_1154 Promoter_1164 D1_Promoter for RE39927, -250bp and +50bp []

AAATAGAATAAAAAAATAAATTTGCAAATTTGATCAAACGTAATTCCAACGTTATTGAAATACTTCCCGTTTTGGGTGAACCACCGGTAAAGATTTACAAGGTACCAGTACCATTCAATATGCTGTTCTTTTTGCACATTTGGCTGGGAATCTAATCTGGAGAGGTCTACCGCGAATGGGCCGTAAATAAGGGAGTCAGTGCCCAGATAAGGCAGCTATATAACCTCTCTTCAAGTGGCCGCGAGGTTCAGTCGAATTCGAGACATCACCATGAAACTCTTTGCCATTTGTGCCCTCCTCATCCTTCCGCTGGTAAGCGGTGGAATCGTGCCACGCACACCTACTAGGCA

>ID_1155 P_1155 Promoter_1165 D1_Promoter for RE51586, -250bp and +50bp [6 ]

GGAACAGATAGAAATAATGACAGATAAAGAGCTACTTAGTGGTTAATACAACTATACTTTAATTAAAATCGGTTAAATAACACCCCGAAAAGATGTTCAGCGTCTATCTGGTGGAATAAATAAAAAATGGAACAATGTTTCACTGTCTATAATTCCTGAGAATAGCTGGGCTTATCTACATTCAATTTACGATCTCGATGGCCAAGTTCGAATCGTAGACCTATAAAAACCAGGCAATGGGAAAACTCCACCATCAGTCTTCGACAACCTTTCATTGGCCGATACAAGTGGTATTCGATCCCCAACAGCGGCAAAAAGTATTACCAACTAACGATGACAGGCAAGTGTTT

>ID_1157 P_1157 Promoter_1167 D1_Promoter for RE31002, -250bp and +50bp []

CCGTTCACCACCCGCAAACCTGTCGAATAAACGAAAAATTAACAAAAAGTCAAAGCCAAGAGCGATCTCAACGAGTTAGTCTTCGATCTGTCTCGAGACCAACGCGACTACCAACAACCAAATCCGAAGCTTGTGAAACCAGAACCAGCCCAAAGTTCAGATACGGAGATTCGGTGACTCGGAGAATCGCAGATCCGATCCCCTAGCAATTCTACGCCTATAAAGCCGGGCCAACAAAATGCCGCGTTCGCATTTCACCCGACGGCATTGTCTCGCGATGACGGGCGGACTAATTGCATCGGCGGTCATAATCTGGTGCGTCGCTCACTCGACGGTCGAGTCGACGGCCA

>ID_1161 P_1161 Promoter_1171 D1_Promoter for RE74157, -250bp and +50bp []

AATATTATCTTTTATTTGTTGGTGTTCCTTCCAAGAAGTATTTACGTATCCTAAGTTCAGGGCAAACAGTAAACAACCAATCCGAATTCATGTTCAGATAAATATGATAATTACGATTATTGTTGGTCTGACTATCAGACAGGTTACCTGCGATTAGATAAGAATGTGGCAGTGTGAGGTCGAAATGTCTGGCAGTGAATGGAAGTGGTTATCAGCTTTCTATAAAAGCTATGTCCATCTGCAGAACCCGGTAGTATAATCGAAACAATAGCGAAATGAAGGTCCTAACTCTTATCCTCTTCACTGCCTTGGTGGCAGTGGCTTCTGGCCGCCCCAATGGCGGTTGGTTC

>ID_1162 P_1162 Promoter_1172 D1_Promoter for RE59221, -250bp and +50bp []

AAGCTCCACACGGATCGCAGCCATTTAATTTGGCCCACACTCGGGAATACGCCTAATCATGGGGGCCAAAACTAATTAAATGATAATGCCGGCGGTGGGAAGTTAGCAGCCGAATCTGGGGACACGTGAAGCAATCAAAGCATCAGCAAGTGCCGGTATCAACCCGCAAACATGGTCGCAATCAGGGGAATCTAATCAATCATCGGACATCCAGTTGGTTTAAAAGCACCCGGCCTTCCCGAGGTCCAAATCAGTTGCGAAACATGAAGTGCCTAGTGTTGATTGCCTTCAGTGGCCTCTTCTTGGCCAGTGCCCAGGCCCGAATCCTTCCCGCGGAGGATCTCCCCTGG

>ID_1166 P_1166 Promoter_1176 D1_Promoter for RE50009, -250bp and +50bp []

ATCGTCTACTAGTTTAACGGTCAACAATTGCAGAAAATATCAAGGCCCGTTCGGAAAGGTGCTTGTCGTAATCAAGTGTAAAAAGTCGCAGTTATTTTCTTAAATAGTTAAAACATATTAAATGTCTTCGTTTTATGATATTAAAATAACTGTTTTATTTGTTATATATTGCAAATAGAGCCTGATTCTGTGGACATGTCTAAACAGGCCTTTAAAATAAAAATAGTGCAATCGATTGGGAACGAAATCGATATTTGTTTAGTACTCCGAGTTTTCTGCAGCACTTTCGTAAAAACTTGCTTTGATTTGATTTTTTAAAAGAGAAAACCCCACTTCAAATGGGTACAAAG

>ID_1168 P_1168 Promoter_1178 D1_Promoter for RE46519, -250bp and +50bp []

ATATATGAGCATCTTATAGCAACTGAAGAATAATTTCTTTTGGACTCTTTGATTTCGCGGATTCACAATGAACCGCTGAGTTTTATAAATAGTGACAATATCGATTGCTTGTATTACTACTGTATATATATGCTGATAGTATCACGGATGTTTCGACATTATCACCGCCACATGCTCTGATGCAGGAAGAGACATTCAATTATTTTAATTGCCGGTAAATATAAAAGGCGATATCTTCGAGCGAAGATTCATCAGTGAGCATTCGATACGCAGAGTGCAAAGATGGCCCAATCCAGAACTAAACACTATGGTAAGGACGGAGCAATGATAATTGGGAGGATGATTGTTGA

>ID_1170 P_1170 Promoter_1180 D1_Promoter for RE44854, -250bp and +50bp []

TCACATATAAGCAACAATCTAACCAACATATAATCATAATAAGTATACCCTCTTTAAGGGTATAAACACAGTTCCTATTCGAAACAGTGCCAGTATCTTGTAGAACTTCGTAGACCGCATAAACGAGTTTTTGTTTTGGTCCGCCGATAAGAAGCCGCTCTTATCAGCACTCTATAACTGATATACTATCTACTAGTAGCTAGAGAGAGTCATAAAAATATATAAAAATCAATGAAACTTCAGACAGTCATACCAATTGAAATCGATAGCTACTACAGAGAGAACGCATTCGTCTGTAAAAACCCAAAAAGTAAAAACGAAAGTAAAAAAACAACTTCAAAATGATGAGC

>ID_1171 P_1171 Promoter_1181 D1_Promoter for RE27458, -250bp and +50bp []

GTCATCAAGAAAAAAATATTTTACAAAGCCTATTCTACATTTAAACCAAAAACAGGAAGTGGTGATTGTAGCTTTCCATCGCTAAAGGATAATTTATTAAGCTCGAAACCCTAGTGATTGCTTATCTAATCCATTCTGGAGTTTCTACAAAATTCAAAATAACCTAGATTTATGAGTGCGAATAAAAGATAGCCTTTCGCTGAGCTCCGATAAGGTTGCCATATAAAGAGTTGGTTGGGCCGTCGCTGGCCAGTTGTTCGTTGACCAACATGAAGCTCTTTGTTTTCCTAGCCTGTCTGGCTGTCGCCTCTGCCGGAGTGGTGCCCTCCGAGTCCGCCAGGGCTGTCCCA

>ID_1172 P_1172 Promoter_1182 D1_Promoter for RE08808, -250bp and +50bp []

TTTTTAGTGTGAATGCTGAGTGTTTCCCCCAACAACAGTAAAACAACAACAGCACACGGCAGATCTTTTACTTGTCAAAAATGTAAACACTAACGTCTCGGGTGGGGAAAAAAAATTAAAAAAAAATAAAAAAAAAAACTGAAAAGCTGAAAAGATGTCTAAAAAAGCGAAGCGACGTCGCTGCCGACGCCGCCGCTTTTTGGCCAGCTCTCAAGTGCGATATAAAAGCCCGGCCACAAGCCGCTCAACGATCCACAGTCCAAAGTCGCTTGCAGCGAAATACATCTATCCATACCCAGTGCACAGAAAGTTGAAACACCCCAGAAAATATGTACAAATTCGTGTTGATC

>ID_1173 P_1173 Promoter_1183 D1_Promoter for RE54693, -250bp and +50bp [2 ]

CAAGTGGCCAAAAGTTAATAGCTCTTATCACGACTAAGGACACCCTTCAATTAAAGAACCCAACGCACGCCATCTGATTAGTTTCAATAAATATATGCATACATGCCAGTCGGGTGGTCAACTTTGGTCAATGCCTTTTATCTTTCATTGATACCTACCTTGCACTTAACCTAGGCCATGCTTATCATCTGCAGATAAGCTCTTGGGGATTCGTGTCTTTATAAGTAGCCCCTCCGAGCGAATAGCTCCACAGATAGTGGTCGATCTTTCGGGTGTGCATTCAACCTTTATTGCAGTAAAAATGTCCAAGTTACCTTGCAGCTACGATGCGGAGAACAAGATCTGGAAGG

>ID_1176 P_1176 Promoter_1186 D1_Promoter for RE31679, -250bp and +50bp []

TGGCAGTGGGCAATCGTCAGATTGAGTGGTGGCAAGTCATGAGCCGGAAAGTGTCCAAATCGGATGGGTAGATCATTGGAGTACCCTGTAATGATAGTGCCGCATTTTGGAGGCGTGCGAAATTAATTTGATAAGATTAGTCCGACCGGAGTGTTTAGAACTTTCGAGTGGCGAATGGTGAAGATCTCTAGCGATATGAATACGTCGGATTTGGGGGGTATATAAATCACTGCCCGTCGGCGGAGTATCCACTTGCACTCAAGTAATCAACATGAAGCTGTTCCTAACTGTCCTGGCCGTGGCCATTGCCTGCGCCGCCGCCCAGCCTGAGAAGGTGAAGCCGGTGCCCC

>ID_1177 P_1177 Promoter_1187 D1_Promoter for RH24988, -250bp and +50bp []

AGTTCCTATTGCTTGCTTGCTTACTTGACACCCAGATACGGAATAGAGTCCGCCCCATTGTTATCTGGCCGAGATTACCGAGACTTCGGGCCAACGATTCTAAGGCGCAATTAAGCGTGGTTGGTTGGCATTGCTGATTTCAATTTCGCACCCAGTCCTACAAGACACGAATTAATCTAGAAGCGGGCTGATTAAATGTGTCTAGCATGGGCTGCCTCCACATAAAAAGGCTTTCCAGCTGGCTGGAATGCATCAGTGATCGGTGAGCCGAGTTAGCAGCCAGACGCATTCCTTCTCCAGTCTTGCAAGTAACCCGAAAATCAAACCATCAACATGAAAGCCGGTAAGGA

>ID_1178 P_1178 Promoter_1188 D1_Promoter for RE23674, -250bp and +50bp []

CCTTTGAGACTGGGCCGTGGTCGACCTTGCCGTGTCAACACCTCGGTTAGCACATTACGCACACCCACGTGGGCGCAGATTTCCAGGGCTTTTCCCATTCAAGAACAAGTTACTATTAAATACGCGTACTCATATCGGACAATCGTAATCAATTCAGACGAAATTAACTAATCGTCAAATACTATTCATAAATCTATCTGCTTATCTCTAGTGGTGGGCAAGTATAAAAGGTGCCATTAGTTGGTAGTCAGTATACATTCTCCGTAAATCTGCAGAATGAAACTGATCACTGTGACTTTGGTTATTGCCCTTGTGGCCGCCGCCCAGGGAGCCAAGCTCTCCGACAAGCT

>ID_1179 P_1179 Promoter_1189 D1_Promoter for RH58567, -250bp and +50bp []

TATATATTAAATACATATATATATATAATATATATCAACCTACATGCTTTCAATAAGATCACAGGCCGGGCAGTAACCTTTGGGCTACAGTCGCTAGCAAGAAAATGATTCAAGGCCTATAAAAAATGCCTTTGATGAAAGACACAATACTAGACAAAAAGTTTATTCTTTACAAATATAGTAAAATATGGAGTTTCTTAAACCCCCTAAACCGCAGCCATAAAAAACCTAGCATACGCCCTTGCCTTTTGTATACTTCATCGCTCTGGTCATACAACACCACATATCTGTGGACGGACGGACACTCCCTTGAGCGGACAAGAAAACTGTCTAATTTGGTAGCAATTTCG

>ID_1184 P_1184 Promoter_1194 D1_Promoter for RH38040, -250bp and +50bp []

AGATTGTGTGTCGGCAAGAGAATGAGAGAGAGCGGCTTGGGAACGCATTTGATTTTAACTCGTTTTGTTATTGTCTCGGGTAGTATAAACAAATGTTTGAAAATATTCGGATATTTCCATAAATTGTTAATTCTATTCCGGATCACGGGGCAAGAAGCGATGAGATTAAAGCTCCGATTTAAACTCGATTTCGTTCTCTCATCTGCTGCTGCTGCTGGCGGTATAAAACGCAACTTCTCAGCTTGTTATCTCCCAGTCGCGCTTGAAACGTCTGTTTGCCCAGAAATCCACGTCACACGCACACTACAGGTTCCGAGCAGTCCAGATCAGCGATTCCAGCGAGATGATGA

>ID_1191 P_1191 Promoter_1202 D1_Promoter for RE42596, -250bp and +50bp [4 ]

TTAACATAAGAGATCGTGGGCCTCGCCGCGGCCTCTCACAAGGAAGAGAGCCACGAGCTACGGGCACTTGGAAGACATTAACATACATAACAAGTTCCACTCTTTGATGGCCAGTGTAAGCGGGCAGCGACGCCGGCAGCGCGGCAGAGCGACGCCGGCTGCGGTGGCGGCAGCGTGGAGAGAAATATCGAAAATGAAATGGCAATACTTTCCCAGCGTTGGTATAAAAGCACCACTCCACCAATGGGACATGTCTCATTACGGTTCACAGTCAGATCCCGCTAAGATGTACAACGCGAGATCGCGTGGCAGCGCTGCCAACGAGGGGATTTCCCCCAAAAGAATAGTCA

>ID_1192 P_1192 Promoter_1203 D1_Promoter for RH25462, -250bp and +50bp [0 ]

GCAACTGTTGCGGACTTATTAGATTAGGCTCGACTTCCCAGATAAGATTGATGTTGAAGGGTTCCCCCCGTTGAGCATAGGCATTGATACAGTGCTCAAATATTAATTTCCCAGACGTCTGGAAATTTTTTTCGTATTTTTGTTGGGTTAATTGTACTTGATTTTGTCAGAACATTGATAATACTTCGGCGCGTGACGACATGATGAGATTGCAGGAGAGTATAAAAGATCGGAGGCGTCCAAAAATTTTATTCACATTAGCAAGACTAACCGAAGTATCAGCACCATGAAGTTTTTGGTACGTTTTACATATACGCTTCAAAGAAAAACCTTTAAGAACTAAGTTGATT

>ID_1194 P_1194 Promoter_1205 D1_Promoter for RE40851, -250bp and +50bp [7 ]

GAATTCTTGTAAACTATTGGACGAAACTCGAAATATTTGAATAACCCACGACGGCCAGACTCTGCCCATTGAAACGCAACCAAAAAATTCCACACCGAACATTAAAAAAAACAGAGGAAGCAAAAACCTTTGTAGGCGGCAAAACACAGTGGCTTCAAAGAAATCTCCGTGCAGCAGACGCCGCGGCGGTATCTTTGTCTTTATCAGCCTCCAAGATGGATTTGTATAAATAATAAAGATCGCTCCTAGCCAGAGACACAAGCAACTCATACCTCAAAGCAAAAGAACCAGCAATCTGCTTAAGATGCGCGCATTCATTGTAAGTAGCGCGGTTAAGGATTCAAAGGATT

>ID_1199 P_1199 Promoter_1210 D1_Promoter for RE03056, -250bp and +50bp []

TCGGGAACATAGCCAACAGAAGAATTCGACCAGTGAGCATATGGAATTCGCAGTCTAAATTGCGATCAAACATAAACCTTAACAATTTCCAGAGACTGAACCGAATACGGCGGGGTCGAAGACAAATAGTTGCCGCATTGTTTAGATATAGTAACTATCTTATCTGTACAAGGATTTAACGCATGAAAATACATTTCCCATGACATAAACACCGAGTATAAATGGGCCGAACAAAGCATTTGGGGTTTCAGTTAATGGCCCAAACGAAGACGAGACGGCATAATTATCGACAAAATGAAAACGACAATCGTGGAGGAGAGGAGTGTGAATGAAACGATAGAAACAATAGC

>ID_1204 P_1204 Promoter_1215 D1_Promoter for RE07882, -250bp and +50bp [0 ]

CCTGACCCAATAGACTAGAGTATCGAAATAGAATCAGAGGCCAGAAGCACAGCTAGGGCCATACCTGCACAAAGGTGAAACTCAGGTGGTCAACTTGCGCTTTCCATGACTTTGACAACAAAGCGAAATGGTCGCTAAAGCTGGAGCCCAAAGCGGTGGGCAGAAAAAGAGCTCCGTGCGTACCTGGCATACGCAATTCAATTAGCAAAGTACCGTCGTATAAAAACCAAGCGGTTCCCATGCGACATTTGCATAACCCGCTGAGTGGCCAACGCGTGCGCACCACCAAACCACCTGACCACCGGACCATCGAACCAGAGTGAGAGTTGAGGACTAACCGTCGTCGAAAG

>ID_1210 P_1210 Promoter_1221 D1_Promoter for RE48639, -250bp and +50bp []

TACAGGAACAGGATCGTATACGAACCTCAAACATGCTAGTGATGACAGTTTCTCACCACGATATATCCTATCAAAATAGTAAAAATATTTTGAAAAATAACACATATACATACGGACATACATATTTAAATAATCTCTTATACCTTTTATAATTTTTGTTTGTATTATTAAAAGCAATAAAATATAATTTCAGATTTTTTTCGACGGCTACCTCTAATGGTGTCTAAAAATCGATATTTAACATTGAGCTATCGATAACAGCCAGCAAAATGAAGTAAAGTAGCGAAAGGCAATTATTCCATATACCTTTTACGACACAGGTTGCTTTTATTGATGAACACGATTGAGGA

>ID_1211 P_1211 Promoter_1222 D1_Promoter for RE50262, -250bp and +50bp []

AATCGAGCGATTGGTCACGGAAAGTTGGGTGGGAGTGCGAGCCATGGCCGCAGAGGTGATAAGACGCTATCCGTCGTAATCGGGCTGTAAATAAATAGACATTAGCGTCGAGTTGCGCCAGCTCTGATTTGCGGGTCACTCGTGCGGGAGTTTTGGAGTGGTTTATTATTTATGGATCCATTTGTCGATTAGATATGTGGCCAGTTGAATGTGTCTAGCTATTTAAGTGGCCCGGCACATCCACGCGGTTGCTCAGTTCGTGCGAAGATGAAGTTCTCTCTGGGTTTGTTAGTCCTCTTTTTGGGCGCCACTCTTGTGGCTGCCGAACAGGATTACGAGGGGTAAGTATT

>ID_1212 P_1212 Promoter_1223 D1_Promoter for RE50273, -250bp and +50bp [0 ]

GGTTCGTGTCTCTGCTAAATGAATCGTGTCTAATTCACAGTGAATGTGTTGGTTGATACATGGTGGTTGTATCAGCTAGGCGTGGTTCGTGTCTCCACTAAATGGACTGTGCCAAATTCGCAGTGTGACCATATATAATAATACCACCCAGGCCCAGTGTGACCGTGCAACCGCCCAGCAAAAATGACACAGCCACGCAAACGCGATAGTATCGCGGCCGTATAAAAAGCGGCGCTCACCGCCGAGCGCCAGTATTAAATTTGTGACGTCAAGAGCTCCGCTTCGCCACCATCGAAGGCCCAGCTTCGCCAAGTGTACAGCTATACATAGAAACATATATCAGGCCGACC

>ID_1214 P_1214 Promoter_1225 D1_Promoter for RE54229, -250bp and +50bp []

GTCTTTTGCGGGTGGCGGAGACGGGTGCCATAAGTGGGTGGTGTTATTACACATTTGAGCAACGTCCCTTGAAACAAAAACAAAAACAAAAACAAAAAAAAAAGGGAAGAGGGGGCGGCAGACTCGGCTGGTACAAATGAGACACTCTGGCGGTAAATATTTAATCAAATGTTGATAAAAAAAAAAAAGAGAAGCCAGAACTGCTCTACACCGATTGCCATAAAAGCCAGTTGCCCTGTGGGAATCGCTAGCAATCAGGTCTGGGAATAGAAATGCGCAACTTCGTGATCATATTTAGCCTATCTCTGGCATTTGGCATCGCCTCCGCCACCGATTATTGCAAAAAGAGC

>ID_1223 P_1223 Promoter_1234 D1_Promoter for RE08916, -250bp and +50bp []

TGGCCCGTAGCGGCAGCAGAAAGCGGCCTGAAATGAAAAGTTGGCCGAAAACTTGTTTTTGGAATACCCGAAAACCGCATATTTTGCATTCGGCGAGCTGCTGCCGAGCAAAAGAGCGAGTCTTCCAATCTTCGTGAGCTCTCTTCGACGCTTTTCAAGGCATCGGGCGCTCGAGCGGAGCGACAGCTCCAATGGTAAAGGGGTCAAAACTTTCACGCGATATAAAATCTTGGTTAGCGATACGGCCGGGCCGCATTTTCATTTCAACTTTGTGTGCGACGAGTTGTCTCTCCAGTCGCGTCCTGTTAGCCACCTCCGCGTCCGCAGCGCGTCCCTTCGTTCCGTGATCG

>ID_1227 P_1227 Promoter_1238 D1_Promoter for RE04580, -250bp and +50bp []

TTGTTTATTTCTTACCTACGATCTGGTGAACGTGAAGGTCCAAGGGCTACCCTCCTCCTTTCGATATTCACATTATTAATAGCCTCTGGTGATTAAGAGTCTTGGAAAAATGGGTGCATTAGTGATACAGCTCTTGGGTCAGCGAATGTCACATTTGGAATGGGAATCGGATGATGGGGGCGAGCATTTCTCTTCGCAGTGACCCACATTAGGCGCCGTATAAATAGTAGCCGCAGGGCAGAGCATTGTCATCAGTTTCCGAGCTAAGCTCCCACAACCCACTTCGACCATCGCCAAGTGCCAGTTAAACCCAAGTGTAACCGACAACAATATGAAGGTAGGTGCTCCTT

>ID_1235 P_1235 Promoter_1246 D1_Promoter for RE47568, -250bp and +50bp []

AACAGAAGCAATCTTTTATGGAGGCCTATTAAGATACAAGTTCAATAATGACCAGTAGTAGCAATAAGGTTCATTTGTTTATAAGTTAAACCATTGATCGAAGCGCAGGCGTCATTAAACCATTATGATTAAAATACACTTAACTGAATTTTAAGTGCGCTTGAGCTTACGTAATCCGATAATGCATTCAAGTACCGATTGCTTATCACCAATCTCTGATATAAATGGGCCAGTTTTCAAAACATAAGAATTCAGTTGTGGTTCTAACCTGGAACCATCAGTTACATCGATTTGTAAGATAGAGGAATACCACAATGAAGTTCCTGGGCATCGTCCTTTGCGTTGCCTTT

>ID_1236 P_1236 Promoter_1247 D1_Promoter for RE35358, -250bp and +50bp []

GCCAAAGAAAATTTCAAATACCAAAATCGTTAGCGACATTTTATTTTTTTCGGCCTTTTTTTCTTCTGCCTCTACGTTGTTTTCCAATTGGAAATTTTGTTTTATTGTGAGAAGCGCCCAGCAGCAAAAGAAACAAGTTGCAGACGAAGTTTGGCGATAATGACGTTGCCGAGTGGACGAGTTGGCCCGAAAGCTGAAGAGCAGCTGAGCACCGGCGGGTATAAAGAGTGGCTCTGCTGGCCGTGGAGATGCATTCAGTCGCTGGTTCCCCACTCGGAAGCAACCAAATTCACTCCACTCTACTCTACTCAACTCTTCCCCCCAACAAACACACTCACCCGAAACGTCGC

>ID_1237 P_1237 Promoter_1248 D1_Promoter for RH63365, -250bp and +50bp []

TTTTGCTCATTTTATTCCCCTAGATATTAGATCCCTCCCAGAAAAATGTTGAAATTTCGTGTTCGCATTTACACTAACTGGTATCTGTGAACTCGACTATAGCATTCTCTTGTGCTTTTATTATAAATACAACCATAACCGTTTTATTTTGTTTTTTAGGAACAGTTACGCAGAAGTGGCCCACAGTGCCCGCGAGTTTCAGTATTTTAAAAATAATTATTTAAACGCAAACGTGACCCTTGGTCACACTGTACCATTAGTACTTGAAAAGTAAAACGAACAAAACGATAAAAAACATTCCTACCATCGAATTATAGTATTTTAACTGCCGTTGAATAATGTTTATTGTG

>ID_1239 P_1239 Promoter_1250 D1_Promoter for RE63419, -250bp and +50bp []

TTAACGGGTAGAAAATATGTATCTCATTTGCAAGGTTTATTATATGTATATATATAGGTGTATATAGGTGTATGTATATGTATGATCCACTAGGATCATAGTGGTTTGTCATCCTTATCAGTTCTTGAAATTCCAGAGATCCGCAAAAGTTCTCGTTTGGGATCTCTGGGTTCTCCGCCTTTTACATATGTCCGGTCTTATCAGCATCTGGTGGCGATGGCATAAATACCCGTGGTCAGAAGCCGACCCAATTCATTCGTCGCGATCTCCCTCAGCATGAACGACCTCAAGCCAGCTACAAGTTATAGGAGCACCTCGCTCCACGACGCCGTCAAGCTGCGCCTGGATGA

>ID_1244 P_1244 Promoter_1255 D1_Promoter for RE35023, -250bp and +50bp []

ATATTAAGAGATATGTTGGAATTCTTTTTGGCCAAGGCCTTTACATGAAAATCTTGTTAGTGGTTGATATTTCCTTGTCGTGTTCGACACAAATTCAATTAGCTGTGAATTTTTTTTGGAATTTTTGGGGAGCGGTCTGAGTGTGTCTATCTCGAAGCATCACGCGGAGATCGCTGATCACTGATTTCTGATTTCTGATAGCAAGATGCGAAGTAGTATAAATAATGGTAAGTGGGCCATTGAAAGAGCAGTACCACAGTTCCCCCGAAGCATTACGGATAACCAAACGGACGCAGCCCAGAGATGAAAGGTAGGATGTGAAAAGATCTCTACAGTATGCGGAGCTGACC

>ID_1245 P_1245 Promoter_1256 D1_Promoter for RE43547, -250bp and +50bp []

GCAAGCCATTTGATTAGCAGACGCTGGTGGGGAGACGAGGAGAGGTTACCCCTTATCGGGGGTTTCCAAGACCAGTAGCTACCAATATTTATAGACTCCAGGGGCGGGCGTTGCTTGGTCATCGCTAGAACTGCCCACTCAAGTGCCCACTCCAGTCGTTTCGAAAACGGAACGGAACCGAACTCAAGCGAAAACAAAAAAAAAAAGAAACACTAGCCTATAAAAGGCATGCGATCTTTGCGATCGCCCACTCAGTAACACGAACCACCCTCAGGTGGCCGCGGCGCGCACTCCAGATCCCTAATCCCAGATCACTAATCACTATACCCACCGACAATCCACGCAAAAGG

>ID_1246 P_1246 Promoter_1257 D1_Promoter for RE43931, -250bp and +50bp []

AACTAACTCAATTAGCCATCGAATGCATTCCAAGCTCGTCTCCGGCTAAAAAGACCCCGCTGTTTAGGCCAATTTTGTTGGTGTCTGTTTTTTTGCCCCCACGGAGCACTCGATCACTCGTTTAAGCCAAGCCTCCATCCCGGTTTGAAGCACGCTCGAACATCGAACCGACGACCGTTTTTGCTCGTTTTGGTTTGTTTTGGCTTTTTGGGGCCAAGCTATAAACCCTGGCAAGATCGTCACTGCGTTCAGTTGGTATAAGTGAATCGTTGCGTGCGGTTGTTCCCACAACAACATTTTAAGCTTGGATATCTGTGCTGTTCGCTTATCGAGTTTTGAGCCCCGCTTTG

>ID_1247 P_1247 Promoter_1258 D1_Promoter for RH51767, -250bp and +50bp []

TCAGATAATCGACACTATGAAGCTAAATTTACTTCGGGCTGTGGAAAACTCAAGTACCAGCACTGACGCACTCTTTTTTCCACCGCAATGGAAAACCGAGTGGGAAAAAGAGCCAGCACGAGCTGGGGGCCCGAAGTCGCCGACGAAGCCGAGGAAAATCAAATTGCGATCAACAGGCCGTTGAGCGAGCGCAGAAAGACAGAAAGAAAGAAAGAAAGGTCTATAAAAGAACAAAGCCCGCCAAGGCTCGGCATCAGTTTGATTCGAGCTAGCAGCCAGAGCAAGTGGCTCCCAGTGTCTCTAGACAAGGATTTGTGGCCGAAGGACTAACCACCCACCCACCTGTGTGT

>ID_1250 P_1250 Promoter_1261 D1_Promoter for RE37683, -250bp and +50bp [4 ]

GAGGCGGTCAGCCGATAAGCTACCAACTATACTATACCATTACCAGATAACAGGGCTATTTATGGATGTTTGGGTCACAGAAAAACTTATTAAAAATCAAATCGGAAGGAATCATGGCCGATTTTAAGAACACGATAGCAACTTAATTAGCTTTTAATCAGCATGATTTAAAGATTTCTCGATGACAGTTCCAACTACCGAAAATTATGATAGAAGAGAGCTATAAAAATATGAACCAAAGGCCGGTAGCATTCAGTACGTATTCGAGATCCGTTAAGTCGGTCAGTTAAGTGGGAATACTAAAAATGAAGTTCGCTGTTGTCCTGTGCCTGCTATTGGCCACCAGTGGC

>ID_1251 P_1251 Promoter_1262 D1_Promoter for RE04513, -250bp and +50bp []

TCGCCGCGGCTTTGTCACCGCGCAGCCTTGGCAGCGTGCCGCCCACCGCCGTCCTACCTCAAATTATGGCCCGCTCGTCCCCCACAATGCTCCCCTTAACCGCACCAGCAAGAGCCGCTTCTAGTCTTGAGCGTCTCGCATTTGATATGAGCGGCGTGGCTCTGTGACGCAAGTGCACGTCGCTGACACCGAACCCGGGCAGAGTGCCATCGAGGCGGTATAAAAGCCCCAGTTGGACGCTGTTCAAATCAGTTCAACACCAAATTCGGTATCTAGAGCAACCCAAACAACCAAAAATGGCATTCAAAGTAAGGACACAGTTCTAAAGTGTTGGAGACAGTGATGAATCA

>ID_1255 P_1255 Promoter_1266 D1_Promoter for RE74918, -250bp and +50bp [3 ]

CTTCCCTTTCGTTGACATCGCGACGTGCGTGGAAATATGGTCAAGAAACTGCCGGCCAGGTAGCATACGCATATGCTTTGCATAAATTGCTTTTGGATTTTTGGTATTGCCGTTCGGTATTGTTATTGTTATTGCAGCGGCGAGCGGTGCGTTTTGGATTTTTGGCCTGCCACGCGATTTCTAGCCACAAAATTGGTCAATCGACGTCAAATCGAAATGGTATAAAACGCGGGGGCAGTGCATTCAATTAATCACAAAGCGATTCCCGTTCACATCGGCAGCACTCACCAGCTCCAGGCAGCCGTCCCACGGATTATAACCTACCCAAAGTCAAGACACCCCCAAGTGCT

>ID_1257 P_1257 Promoter_1269 D1_Promoter for RH08424, -250bp and +50bp [5 ]

GAATGCCAATCGCATCGATCTCGAATTAATGTGGCTCGCACTCATGGGGAGGAAAACCCTGCTTCAGGGGTAATTGTGAATTCAACTTACAATCGGGGAATTCTTCCGACAGAACGCGTGGAATGCCGCCGCCTGCCGTAGAAGATTTCGTCCAATTTGAATAAAAGCTCGCCGAGATTGGGCCACCAGTTGCATGCCAATGAGCGCAAGTCAGCACCATTCAGCATAAATAGATGCCATCTTTGATGGGGAGGCACACTCACCATGGATCTTCTCAAGAGGCACCTGCTGCTCCTGGTCATCGGAGCTTGCCTGTGTGTCTGCGCCCAGGCCACGATCACCATCCAGAA

>ID_1258 P_1258 Promoter_1270 D1_Promoter for RE73746, -250bp and +50bp []

ATCGAAGAATCAGTTCGCAAATCGTTCACGAATGGCGCAATTGAAAAGCCAATCAGCCGATAAAAGAGCCATCAGATTTGGAGTTCTTAAAAAAAAAAAAAACAATGGCAGTATGACCGGCAATCAGAACGGCAATTCAAATGACCTAAACCTCCCAGGCAGCCAAAGTACGGCCAACCGATTTAATCACTCCGTGATAAGTTGGCCCACTGGGGACGTATAAAAGCTATGGGTTTCGGACTGAACTACCATCAGTTGTGGGCTCCGCTACGTTATGAAGACATTCCTGATTTTGGCATTCTTTGCTCTGGCCGGTAAGAATTTAAGAATTTCCATTTTTAAAGAATACT

>ID_1262 P_1262 Promoter_1274 D1_Promoter for RH63850, -250bp and +50bp []

GTCTGGAAGATTGTTATATCGAGCCTGTCAACCGGTATTTAAAGCAAAACAATGAATGTTGTATAGTATTTACAAATTTTTATCATGTGCTTAAACCGTGGTGTATTGTATGCTTTATCGACGTAAGTTGAGAAAGTAACAGTATATTTTGTCCCCGTTAACAGAGGCAGAGCAGTCGGCGTCAGAGGGTAATGTTGGCTTTAAAGTTGGGAGAGCTGCGTCTATTTAAGCCCCTCGCAATGACAAATTCGTTGTTATTGCAGAACAGAGTCTCGAACGCGGTGCTTTGGTTTCGGTTCCGTTCGCCTCGTGAATATTTTTTTAAGGAATATAAGAACAATGCACGGATA

>ID_1268 P_1268 Promoter_1280 D1_Promoter for RE71379, -250bp and +50bp []

ACGTTTAAGCCAAGTCGCTGAATCACAAATATGCAAATGTTGTCGCTAAGATGTGAGTGTTTTATTAAGTCACATTCTGCGCGCTTCCATAGAATTTCTTTTGTGTTTTTTCCGTTTTTTCTTTCGCTTTTGTTTTGGGTATATTTCTGTATTTTTTGGTTATGGCGTAATGGGACTGAAAGATGTTAATTCGCGGCTGCTGCGCACATTAAACGCCGGTATATAAAGTGGATGGAAGATGTGGTAAGCCAAGTCGTCCGTCGGTTCCCGCCTAGAAAGCACCAAATCTCTCCCATCTCTCTCACCCCACAAGAATAGCCAACGAAATGTTCAAGATCGCCATCTGCTTG

>ID_1273 P_1273 Promoter_1285 D1_Promoter for RE38426, -250bp and +50bp []

TTAAAGCTATTGTCACGTGTACGATAAGGTTGATTTAAATAACAGAGATTTCGTATGCTTAATCTTACCAAGTATCTATTGGTACTATGTTTTAAAAGAATTGATTAAAGTTACCTGCAAAGATTAGCTTATCAGGCGTTTTCCATTGAACTTGTTAAGAAATGACTAATGTCACTGATGGTGTTCGTAGACGGTGCTTATCTCATTTTAATTCGTATATAAAAGGGGTTCCAAGTTCGGTGAGAAATCATTAGTAAGAGTTAAGCCAGCATGGTTAACATAGTCGGAGTAGTTTTCCTCTGCGGTCTTGGCCTCATCAGTGCCGGACGCTTGAATGACGAGGATGTTCT

>ID_1274 P_1274 Promoter_1286 D1_Promoter for RE11624, -250bp and +50bp []

AGATTAGGTAATAATGTTACGGCTGTGATTTTGATTGGCCGACAATAAATTGGAATTTTCAACTAATCCAATGTTTCGATCGATTTATGATGGACAGCGAATGCGAACATCTAATCGCAACTTAGGAAGCTATCATATCATGTCGCTGGTCACTTATCAACTAATTAATTTACAATCCTTATCGTGGTAGGAAATAACTTTAGAATGTGACACCGTTACCTTTAAAACCATAATCGAACCCGATAGTTGCCTCATTTGTGAAAATGGCTGCTCTGCGTTTGGTCTGCCTTTTTGTGGTCTTGGCCATAGGACTTGTCCAATCCCTGGACATTCCGAAGATCAAAGATGTG

>ID_1306 P_1306 Promoter_1319 D1_Promoter for RH45339, -250bp and +50bp [1 ]

CGGTGACGTCATGCGTTAGCGATTTGTCCTGACTCCAACATGATTTGGCCAATCAGGACGTGCCACCCTAAAAGGAGGTGGGTCCTCTGAATAGAACAGAAAAACTCACTTGTAGCTTTTATATCCTTGAACGAATTTATCTCGCCACGCACACTTAATAAATACAATTGAATATGTATCTCCTGTGTGCCCGCGTGTGTGCGAGTCCTGCGGGCTGCCTATAAAAGGACTTTCGCATCCGAAGGATCTTGCAGTTGGCGCTGAGTTGCGCATACGACGCGTGTGCCCGCGGAAATCGAAAACGAGTAGCTACCAAGTGCAGTGCAGTGATGAGTGCTTCCGGGAAGAGT

>ID_1310 P_1310 Promoter_1323 D1_Promoter for RH23342, -250bp and +50bp [1 ]

ATATAGCCTTATCTCTAGGGGCGTTGTTTGTTTTGGTTTTTTATTATTATACATATGTTCCTCATGTTCAATAAGTCCACGAAAACGAGCTAAACCTAATCTATACACATTACTGACCTGACCTTACCTGTATTAGTGATAAGAAAGCCGGCCGAACCGGTTCTCTTTAGTAAATGCTTTAAGAGAGTATAAAGATAAGTATCTCCGAGAGAGCCCCAATATATAAAGCTCCCGCTCAACATCGTTTGATATTCAGTTTGCCGCTGTTCTTCGACCCACAACAACCGCCAAGCAATGTCCAATAAAAGTAAGAAAAACAAGAGACGTATATGTACACATTGTACTGCAAA

>ID_1329 P_1329 Promoter_1343 D1_Promoter for RE32569, -250bp and +50bp []

CAATAACAAAGTTGAATGAGTAATTTTTGCTTCTCTTTTTTACTCTCTCCAATTGCCATTCAATGTGAGCACGAGCGAACACACATAAGCAACCCCCGTTACCTGCGAAGGTTTACCTAGGATAGCAGAAACAACAACTATGAGTGCAGCTGCAGGCAGATAAAAGGAAAAAGGGAGATAGCAAGAAGAAGCAAGGGACACCGCAATAACAAAAAAAAACACATAAAACAGAGTGCCAACTGTTGGCTTGCATTCATTCGATCGGCAACATTCCAAGCGAACGCATCGCTGCGAGAAAAACAGTGCAAAATAATAGAGAGAGTTGAAGAAGGATTACCAGTGTGAAGATC

>ID_1338 P_1338 Promoter_1352 D1_Promoter for RE19437, -250bp and +50bp []

TGTAATCCTATTTGCGCCAAATAAAGAAAAAGAAGAAGACAAATTTGAAACGAAGAATGAGAAGGAAAGAAAATCCAAGCTGCGCTGCCTCCGTTGAAAAAAAAATGAGAAAAACCAGACTGCTTCTCTCTTCTCTCTTTTCTCTTTTCATTTCAAATTTTACGCTCTCGCTCTCGCGGACAGCGACGCCGCCGGCGGCAGCGCAGCGCAGCCGTTGTATTTATAAATACAACTTTATAAATACAACATCAGTCGATTTTCATCTGCCGAATCGGTTGCGCTGCGGCTGCGCTGAGAATAAGATACAAAACTCGTGCGCGGCGCTTCAAGAAACCAACTCAAAAGCAGAC

>ID_1342 P_1342 Promoter_1356 D1_Promoter for RE18783, -250bp and +50bp []

ACTTGCAATTCGCATTTATTGCCACTAAGCAGCTGCACATTGGGAAAATCCGCTCAATCAGCGTCCTTCGCAGGTAATACACAATTAGAACATTAGATCCCAGTATGAGCCGGACCAAGTTTTAATCCCTTCGAACTGACAGATGTTAGACATTAGAAAGCCTAGATAAAAGTATGGAGTTTACGGCAGGTAGAACCATTTAACTAAACCCATTTACATCTATAAAATAGCTTGCAGCGAGGAGGAGTTCATCAGTTTGCCAAGAACTCTTCACCTATCAAGACATTCAAACAAGACGCGTAGAAAAGAACCAAAGCCAAGCCAATTATATCAATTAAAACTTTGTTCAA

>ID_1343 P_1343 Promoter_1357 D1_Promoter for RE70611, -250bp and +50bp [3 ]

AGCTCGAAGAAAAAACGTTATGAAAATAATTGAAATAAAAATGCGGTGCCTAAAGGTCAAGTCGACGAACGAGGTCCAAAACCTGGACCACTTGACATTGTCCAAAGCAAAACTGAGGCAAGACGAAGCACCCAAGAAGAGCACTCCACTCTGAAGTGGCTCCATTTGTTACTGGCCTTTTTGTTTTTGGTTTCTCTAATTATGGCCGAACTCGCTTTTGGCTATATATTGAGCAGTTGGCCATTAGCAAATCATCAATCAGCTCAGGCAACTTTCAATTGCAAGACGCAAATCGTAAACCCGAAAAGGCAAACTTGAAAAATGGTAAGTCCTCAAGGATTTGAGTTTGC

>ID_1349 P_1349 Promoter_1363 D1_Promoter for RH49718, -250bp and +50bp [2 ]

TCTGGCCAGCCACCTCGTCACCTACCAACCCACCCACCAATCTAGGCACCCATACCCATCCCCCTACTAGCCAAGTTGCGAGTTGGTTGTTAGTTGTTCGTTGTTGGTTTTGGCCGTACTCGTGTCGCAGCTTTGCATCTTGGCTATAACGTGTGTGTGTGTGTGTGCGACGCTTTGCCAACTGAAACTGAATTGTTTGCAGCCTCGCCAGTGCAATTTTCGTATAAATAGACTCACCTGCCGGCATTGGGCATCATTAAATAGAAACGTTCGTTCAATTAACAACTGCAGTGTGCTGAGGACCCAGACGAAAGGATACACTTGCCTTTTGGATTACACTACCGAATTTC

>ID_1355 P_1355 Promoter_1369 D1_Promoter for RE09592, -250bp and +50bp []

CTATGATGAAATAAAAAGCCTCATTAAATATTAAAATCGAGAAATTACAAATTTCTTACGAGCTTTAATGTTAATTTGAGGTAAGCTTTGCTCTCAACTTATATTTCTGTAAATCAAAAAGGCCAAATGGTATCAAGACACCATAAATTATTAGATAGAAAAAAGGCAGTGCGAATCCTAAACCTGATACCTTCTTATCAGATACCGTGTAGTAGCGATTGTATTAAAAAGGCCCGTTCGGAGCAAACCGAATTTAGTCTTGCCGCGAGTTTCAACGAGCTACAATGGCAGTTAGCCAACGTTTTTAGTCTGGCTAAACAGATGCATTTATCAGTCATTTGTATTCGGGC

>ID_1388 P_1388 Promoter_1402 D1_Promoter for RH01917, -250bp and +50bp []

TGCTTTACAACAAAAATATAAAAATATGCAGCAGTTTTGATTTTTTTTCAAAAATTCTAAATACTTTTTGAACGGATCGGTGCTGCAAAGCGCCGTTCCAAAGGCCAGTGCTGCAAGTCGGCTGAGTCGTAGCGCGGTGAAATTCAAATAAATGCGAAATTACTTTGCTTTGCGTGTGTGCGTGTTGCGGATCGCAGCGTCAGCGTCGATCGCTGCTCATAAATAAAAAGCCGCAGCGGAGCCGCTTTGCCAGCCAGTGTGCGTTTTGGCATGAAATGAAACTGTCGCTTTTCCGCGTGGCCCGCTTTTCTCGCAGTGTATCCCAGTGTTTTGCCGGAGATCGTTGATAT

>ID_1394 P_1394 Promoter_1409 D1_Promoter for RE67852, -250bp and +50bp []

TGTGTGTGCGTAGGACTTTCACTCCGTGTGTATGTGTGTGTGTGGAAAAAAGGGTGACTAATGTGTGTGTGATTGCTAACAAAAGATTCCGGCTTTTGCGGCTCTGCCGGCGTCGAAAAAGGACAACGACTTCGGCCGAAAAGGGGGTTGAAATGTATTTCCAGGACGCCGGCACAAACAATGATTTCGAGTTTCGGTTTCGCTTTCCTTTGGCCTGTGGTACACATAAATAGCCGCCACCGAGTCCACCGAACGTCATTCGCAGCACAAACGTCGAGCCGACTGGAGGTCTTCTGCATAGAGTCCCAGTCCGCGAGGATCAGCGTTATCAGTTTGTTGTTAAGTTTGCT

>ID_1416 P_1416 Promoter_1431 D1_Promoter for RE02655, -250bp and +50bp []

AAATTATTAAATTGCTAATAAATTAGATAAATTCCAATTTCTATTCCAATTAGACAAATAACAGAAATTCCCTTACAACTTTTTATCTTTGAGTTCGATTCGATGCCGTTGTTGTTCGGTTCGAACTCGTAGACGAGCAGCAAGCACGTTGCGTTCGAAGTTCGAACGCGACTCTTATACGGCGGCTAACACCCAAGTCCAATTGGAATCGGCAGCATAAAATATAGGCGCCCGCTGCGCTTTTTGGCCATTTGTCGATGCGACGCCGTTAGCCGCGTGTCTCGTCTTCGGGATATCCGATTTCCGTCTTGGCCTGGCCCTAAAAAAAAACCGAGAGAACCGATTGTCGA

>ID_1425 P_1425 Promoter_1440 D1_Promoter for RH71862, -250bp and +50bp [3 ]

CAGCTGCGAATGTGAATGGATCAATGTGAGAATTCAATCCATCCATAAACACACGAGGAGGAAAACAAAATTCGATCAAGAGGCGACTTCTTGGTCATGTGTGTCTGGATGGGTTGGGTGCCTATCGTCACTTAAGTCGATGAACGCGTCCACATTTCACATAACTTCCACATATAGCATGTAGCATATAGCATATGCTACATATGTCTGTCTTGGGGGCTTATAAAAGAACGCGTATCGTCAAGATTCGGTTAGTTGACTCCCAGCTGTGTGTCGCATCACAGACCAGTGCTAGGATTTTCGTGATTGAATCTGCTTTTTTAAACTTCAGCTTAAACTTTTGATCATCA

>ID_1426 P_1426 Promoter_1441 D1_Promoter for RE63284, -250bp and +50bp [2 ]

TAGATGTGCTTTTAGTTTAAGATAAAAATAATACTTTTTCGGGGGAGTAGGTAATATTTTTAAATTACGAACATATAACAAATACTTTTTTATTTTATTACGAAAAATAAAAATGTTGAAATGCTTAAAAATTATACAAATAATTAGAAATAGGAAAATTTATTACGAGTACCGTTAAGTGGTTTAAGTCAGGATAGCTGTTAGTCCAAGACGCGAACCCACATATATTTATGCCCAAACATTTTGGATATAGTTCAGCTCTGGTCCCATTTAATCCTGGCAGCGAAACAAAACCCGACAAATTGATACAAATTTACGGTTAATCGTTAATAATTTCGGGTAAAAAATCA

>ID_1429 P_1429 Promoter_1444 D1_Promoter for RH58075, -250bp and +50bp []

TCTATTTTTGAACTAAATGTAAAGAATTGGATGCAAAAAGAACGCATTTCTCCCGAATGGAGTAATAAGACGAACAATTACGAATACCCTAAAATAATATTGATAGTTTAATTAACATCGTTAAAAATATCGGTCCATAACAGCACTGCTAAGTGTTAACATAACTAAGACGCCATCTACAATTTTGTGAGCGGCGTTAGTGTGACCAGGCGAAAAGAGTGCTAAATATACCGCGCATTGCCCTCCGATTCACATCGTCAAAAAATTTTGGTCTGTGAACTTCGTGCGGATGAAATTCGATTTGAACGCCACCGAAAATATGCAAAATAAAGTGAAATACCAGTGCCTCG

>ID_1431 P_1431 Promoter_1446 D1_Promoter for RH70762, -250bp and +50bp [3 ]

ACTGAGCCCATGGAGGTTATTAGAAGGCATCCATCCAGATTCCAGATTCTTTGTTGTCCACTTGTGCCCCCAAGCATAAAAGCCCATCACAAAAGAATCAGATGCCCAGCTTGGAGTTGAGCATAAAAATAAACTGGGGATCTACAAGCATAATCGCGCCTTGGAGACCCCTCAGGTACCAAGTCCATTCCCGAACTCAAATCGGATCCCTCGCTCTCGTATAAATAGGGGCTGCCTGGCCCTATTTCAAAACAGTCTTCGCTCGATCGCTGGAGGAATACATACATAGGTGGAAAGAAAGTGAAAATGAAGGTTTTCGTTGCCATCTGCGTGCTGATTGGACTGGTGAG

>ID_1448 P_1448 Promoter_1463 D1_Promoter for RH25966, -250bp and +50bp []

TCCCAAATAATATCCATAAATATAAGCAATTTTGATCTGCGCATTTCGGACAAAGCATTTCAATGAAATAATGAATGCAATATTATATATTCAAAGGATACCATTTTATGCATTGTCTTCGCATCGATTGGAATGCTATATCAAAGGTTTGTCAAACATATTCTGTAAGTATTTCAGCAAATTTATGTAAAACAAGTTTCTTTCATTGGTTACACACATTTAAATATCCCGCGTTGCCGTAACTTTTTTTTTTTTGTTGCCAGCCCTGTCATTGGATTTTACACAACCTCACAAACGGTCACATCATCGGCTGACAATTGGAAAATTGGAGAAAAAATTTACTTTGACCC

>ID_1452 P_1452 Promoter_1467 D1_Promoter for RE71854, -250bp and +50bp []

TTTCCCCACCAGATCATGACTGGCCAAACTTTGGACTTGGCTTAAACATTTCAATTTCTTCATGGCTACATGCTGCTGCCGCTCTTTGCCAACTGCTCAGCCAACTTGGCCGACTGCTCGGTCGACTGCGGCAGCAGCTGAGGCAGCTGAGGCGGCTAAAGCAGCAGCGGTCTTGATCGTCGTCTTGAATTTTACTTAATTTTTATTTCACGCGCTGCGCTGCTATAAAAGAAACTGCAACAATCGTTGTGGCATGCAGTAAGAGGTTCCGCGTCGAAGCAGCACCACATTGCCAGTAACCAGTAACCAGTAACAGCCAGTAGCACCTAGCCACCTGGAGCAGTGACAAA

>ID_1454 P_1454 Promoter_1469 D1_Promoter for RE01355, -250bp and +50bp [3 ]

TCAAAATTTAATCAACGCCGCCGCCAACGCCACAGCTTCTGCTTTTGCTTTAGCTTCTGCCAATAGGTCTAGACCAAATCGAGCTTGGGCCAAAGCCGCCAAGTGGAAAAAGAAAAACAGAAAAACAGGTTAGGCTCGCAGACAATGCGAACCTTATCGCGATCTCCAGCACGCTGTCATTCGCCTGTTCTGATTGACGCCCCTGCCGTGTCGGCCAGGCCATATAAATGGCCCGGCGGCTCAGCTCGACATCATTCTGACTTCCGACTCCGGAGCTCCAACTAGAACAGCAATCAGTTAACAGCAAGATGCGCGCCTTCGTCGTCCTGTGCCTGGTGGCCATCGCCTCC

>ID_1456 P_1456 Promoter_1471 D1_Promoter for RH39220, -250bp and +50bp [1 ]

TAAATATTTATGAATAAATAATGTTGTTGTCGATTTGTCTCTATCTCTCTGGCTTATTCGAGTGTTGCTGAAAATGTGCACCATCATAGATTAAAGTTTCTACCTTAATAATTAATGTAGCTGGTGTTTTTGTGTGTTGGTGTCATAAGTTGATAACAGTTGTGTACAAACTTGAATTATTGCTTATCAATTTGCTATGTGTAGGTGTGAGAATGTCTATAAAAACATCTAAACTAACCGGATTCAATTCAGTTTGCAAACGTTAGCTGACCGAGAAGTATTCAACGGTTTCTTTAAGGTGATAAAAAAAAGAGAGAGTGTTACATGTTCAATTAAATTTATATATGGAA

>ID_1457 P_1457 Promoter_1472 D1_Promoter for RE04770, -250bp and +50bp [2 ]

TACAAGCATTTATATCATATGCAAATTAATGAGCGCAGTTATTAGAACGGTGCTTGATCGATCCGAGATCGAGATCGAGTTCGAGCTGGAGCTGGAGCTGGGGATGGAGATGGAGATGGACACAGAAGAAAGAAAGGCATCCCAAATTTGGGCCACTGCATGGGTTATTCTAATGAGGAAACCGGGAACGAGATCCCACACATCTCATCACAGATGCACCGCGTATAAAAGGGCAATGCAACTGGAGTGGTCAGGTTATTCCGAGAGAGATCATGGATCGGAGAGTGACTGCGATTGGTTTGGGTGAGTACGGACGATCGGCTGCCTAGAAAGAGTGACACATCCTTTTC

>ID_1465 P_1465 Promoter_1480 D1_Promoter for RE20251, -250bp and +50bp [2 ]

AAGGACACGGCCGGCACAGTGGTAATTAGCCAGGACATAAATGTTTCACGTGACGGTGTCCCACGGCCAAGGCATCTTCTACCTGAACCCTTACAAGCAGGTAAGGTATGGAAAATTGCTTTGTGTTAGGGATTAGAAAAGAAGTGGGATCTTTAGGATTCCCAGAATTTGTGACATTAATTGCAAAACAAGGACTAAAATGGCCGAACTAGACGAGGCTTTAAAAGAATCGATCGTGTCTGGGCCAGGATCAGTTGTTTTTCCACACCCAAGCGAAGATGAAGTACCTCAAAGACTGCAGTTTCCATGTCCTGTTGGTTCTGCTGCTAATGATCCGTGGATCGCTTTGT

>ID_1468 P_1468 Promoter_1484 D1_Promoter for RE12587, -250bp and +50bp [0 ]

CGGCGGCTGGTCCCAATTATGGAACTGCTATGTTTCCCATTTTTTTTTTTTATCCACCGGACTCATTTGCATTTGCCAGCCGTCGATGCGTTTGTTGTTGGACCAACTCGAATCCAGATGGACGGAGATGGCTTTCGGGTTGGTGGTCAAAAGAGTGGTGGGCAGCAAGATGACGAAAGCAAGTTCACAGACAGACGGAGCATCTCGCAATCCGGCTGCTATATAAACCGCGAATCGCTCGGATTGGAGCATCAGTTGGCTTCCTCATCTGCTACGTGAAGTTCCATAGTTTTCAACCCCCAAGCAAGAAACTTCCAGCAATGAAGGTGAGTCTGCTCGACATAGTGCAC

>ID_1471 P_1471 Promoter_1487 D1_Promoter for RE01906, -250bp and +50bp []

TTAAATTAAAATCATTTCTGTTTTCTTATATTGCCTACGTCGGTTGTAAGCGAAGATAATCAGCTTAGAATTTTTCCTAAAAGCGAGTTATTATTTTTTTTGCCTATCAAGATGTTCTTAGACGTGCTATCAATTAATTTCCATTGGTATCAGAGGGCGAATTTCTTAGAGATAGGATAACGCCGAGTGGGGGCTACTCCAGACAGCGATAAGGGGCGCAATAAAAACGGATGGAAGTCTGGCGCAGACCACGATTGATCCAATCACCATGAAAGTGTTCGTTGTACTGGCTCTGGCTTTGGCCGCTGTCTCCGCCGAGACTGTCCAGCAGGTTCATCCCAAGGACCTGC

>ID_1472 P_1472 Promoter_1488 D1_Promoter for RE01052, -250bp and +50bp []

AGCGCAACTATCCGAAAATGTTGCGCAGCATCCCATGGTAACTAGCCCCAAACTAATGAAAGGTCCCCCTCTGTCCACTGACCACTGGGCAGCTTGGAGTTTGGAGCTTGGCTCGCTTGGTTCGACAAGCAGTGGGGGCAAAATGATGCGGAGATGGAGAGATGAAGATGTCGATCGATGTGAATGAAGATGCGCCGACTAGTTTTCGAATGGCCCTTGTATAAAACGCGTGGCTGGGTCATTGGGGGCATCATTGATCGGTTGGACATCGAGGGCTCAAGCACGACCACTGTGAAGATGAATGTAAGCAGGGCACTTGCCCATGGCTAAGGATTACCATTCAGCCAAGG

>ID_1473 P_1473 Promoter_1489 D1_Promoter for RE01054, -250bp and +50bp []

AAGTGCATCCATTCACCCACATAAAAAACTGTCGGATGCGAGTGGCACGCGTTCGAAATAAAGCCGCATTAAGCATACGACACGTGGTGCGCCGAGGCACGCACACAGGCAAAAATAACAAACTTGGAGCAGCTCTGTCGCGTTCGCTTCATTCATGTCTTCTCCGGCCATTTCTTTTGCCCGGCCATTCGGCAGCGAACGAGTTAAAGATCCAGTGAGTATAAAAATCGTGAACAATAGAAAAACGATTATTAAACGCATTTGTGCCAGCGCAGAGGCCAGTTGTCCGTTGACCAGCGAACCTCGAATTAAAGAACTCGAACATCAAAATGGCCAAGTTGCTCCTTGTG

>ID_1474 P_1474 Promoter_1490 D1_Promoter for RE49895, -250bp and +50bp [3 ]

AAGCCAAGACAAACGATCGCGGGCTGGCGATAGGCCTAGGTCGGGGAATACGCTTGCATATTTAAACGGTGGGGCATTTTAATGACTGTCGAATAGCATCGAAATCAAATCAACAGCGCTGCGACAGCAAGGTCGTTCGTTAACAGGGGATTTCTCTATCTACCTTGGCCAAAAACTGGGTCTATTTCGTGGGAGGGGTAAATGCAGTGCCGCGTCTTCAACTATAAAAGCCAGATTGTGCTGAACTCCCAGCATTAGTTCCTCCTGTCAGCTCCATTTGTACACAGCTTCCAGTCACCATGCGTGCTTTTATCGTAAGTGTCTAGAAAATAAGGGAATCGAATAATACA

>ID_1477 P_1477 Promoter_1493 D1_Promoter for RE04224, -250bp and +50bp [2 ]

TCTATATATCTATCTATCTATCTATCAGCATCTCAATCTTCATTCCTTAGATCCAATAAACCTATTAAGCCCGGATCCATCGAGCGCTTCTTCATTCTGACGACCCCCAAAAACAAAACCAGTAAAAAATCTCATCTGCGCTGAATATTCAGATGAGATGTTGACGATGATCTGCGTTCGGCCTGAGGTTCAGTTCAAGTTTTTGGGCTCGGTCTTGTATAAATAGTTAGCCATTGAGCCTCTTCAGACAGAAGCTACTCGATCGCTCTTGGAGTAACAGATTGCAGCTGTCATCATGGTGAGCTTACCCACATGGATGAAGGATCAAGGATTATATTTCTAAACGCTTT

>ID_1480 P_1480 Promoter_1496 D1_Promoter for RE57116, -250bp and +50bp []

ATCTTCACCTTAGTTGTTTGAATGTGCATCACATCTTGGTTTGATTGAAAACGAAAAGAGTGGATTTTTGAACTTGTGCGTAGCTCTTTGGCTCTCAAGTCACTCTTAGTGCTCCAGTTTGGCCAATTTCGGCTATTCGATTTTATTGTTATTCAGATGAGTTTCCTGCAGCAGAGCGTAAAAGTTGTTTCGGTGGATCTGGTTTGATGTCGACAATTGGTATAAAAACCTAAACAGTCTGCAGTAAAGTCACCATTCAGAGTCGTCTCTCAGTTTAGACAAACGCACTCCAAGCCAGCAAAGATGTTCCGCTACGTAAGTCAAAAGGATATTTACTTCGACCTAAAACG

>ID_1481 P_1481 Promoter_1497 D1_Promoter for RE53127, -250bp and +50bp []

TTGATGCAGCATCATACTTTCATCGGAATCTTTGTTGGTTAAAATAAACTTTTTTTCCAGTGTGCCGGCATCAATGGGACGGTACAGTGTAATTTCAGTACGAGTCGTATGGCTTCGACACGATTTGCTTGTTTTGGCGCACGTTCGTGCTACGCTGCGGCTACGCGATGTCCGGCTCCCGAATTTCAAGTAGCCGCCCAGTAAGCCGGCCCAGACCCTCGATAAAAGCCAATGTCAATGTGAACCCTCCATTCAATTGTTAGCCAGCGTCGGAGTCGAGCAGTGCAGTGTCGAACGGATTACCAGATACTCATCCTCAAGGAATCAAATCACCAACAGTCAAATCAAAT

>ID_1483 P_1483 Promoter_1499 D1_Promoter for RE07247, -250bp and +50bp []

ATTGGGGATTTGAGGAAAAAACCCAGACGAAAAATCAACACTAACAGCGAAAAAGAGGGGTTGCCAAACATTGTCGCCGTCGCCGCTTTTGTATCTTTTGTTTTGGGCTACCGATTCGCAAACTAAGGCGGTATGAAAGCTAGAAGAAGACGGAATCAATGCAGACAGAGACAGCGAAACGTTGGAGCTTGACGTCGACGGCGCGCATGCACAGCTTTTGTATAAAATGTGCGCGGCCAACGAAAGCGAAATCAGTGAACGATCTGCAGAGCATCGAAGAGCACAATCGGTGCAGAAAGTGAATGAAGTGAAAGGATAAGAGACTCAGTAAATTGCATAAGGATTATCCG

>ID_1488 P_1488 Promoter_1504 D1_Promoter for RE69682, -250bp and +50bp []

TTTTTTGCCACAGCAACTGGAATTTACCGGATTTTTAAATTTTAAAGCAGCGCCCACGGTAAAATCAGTAAAGCACTTTAACTATAGTGTAATCATTTAAGTAAATTTGTACGTATAAGGAATTGGAATTGGAAGGAATTTGTTTTCTATTTAAAAAACGATATATACCGCACTGCCAGCTATCGATAGCAAATAATCGGCTGGTCGTGATTAAATGTAAAAATTACTTCCACGCTCTGGTAACACTGCATTGTATACCAGAAAAAAATGGTATTAGCATTAGCAGAGAAAAAATCAAATAAATAAAGAAAAAATCAATTAAAAGTGCGCCATTCCAAAGGATTATCCCA

>ID_1489 P_1489 Promoter_1505 D1_Promoter for RE26879, -250bp and +50bp [3 ]

GCAGCACCACCGAGAGCGATTTGCTCTTCCTCAGCTCCGTGTGCTTTGCATTATTTTGTAATGTTTTTCAAGTGCTCGTTCGGCACTGTGGCACTCATTTTTTTCTCGACTAACCGAAAATTGAAATAACGAACTAAGCTGAACAAAAAACACACACATTTTGGCGCGCAATTTTTTGAAGCCGACGTCGACGCCGCCGCGTTAGCTGCTGCTCATTTCAAATATAAATACTGGCCTACGAAATTGTTTCAGTATCACAATCAAGTCAACCATCTTCGCAAGAAGAACACAGCCAACAGAGCAAAGTTAACAAAAACCGTTTTCGTGTGTGCCAAAATCCCAAACAAGTG

>ID_1491 P_1491 Promoter_1507 D1_Promoter for RH04334, -250bp and +50bp [6 ]

TCACACGGACAACATGGACACTGTCCGTGTGAGGCACCCTCTTTCGAAAAAGCTCGCTTGCTGACGCATTTCCTTTGGGCCACGGGCTCACACGGACAGTTGGCCAAGAGAGAGAGGAGCTGTCGAACGGATGGAGAGCGAGCTTTTTCAACACGGCTGCTGCGATCTCGATTCACGTCGACTGCGCAGCCGCGGCTGAAGACAATCCCGCCACAGCATCGGCCATATAAAAGCTCGCTGCGTCTGTGCGAAATTATTCAGTTAACTGTTGCGGGTTGGTTGAGACATACAAGCCCATCTTGATCGCAAAGTGTTGAGGAGCTAACCTAGTGCAAAATGAAATCCATGGT

>ID_1492 P_1492 Promoter_1508 D1_Promoter for RE30084, -250bp and +50bp []

TAATGAAATAAGCATTTTCAATTCGATTCATTCAGTTCCTTGGGCCTAACCCTCGTTTTATTATTGTTATGGCCCAGCGGCACGCACCCGGTTCTAGCTTCCCAGTATCCAGAATCGAACCCCCACCCAAAGTGTTAAATTCCCCCTCTGCCGGCACGCCTGCGTCATGCGAAAGTATTCGATGGTGGTGCTACGGTGGTGGGTTTTCCACTTGGCACAGTATAAAAGACCGATCCGATCGACATTGCGGCACAGTCCAGTGAAAAGTGTCATTGAGTTACGATCTCGGTCCCAAGATCGCTGAGCCGCCACCGTTGGCGGCATTGGCCGTTGCTTAAAGTTCAATTAAA

>ID_1494 P_1494 Promoter_1510 D1_Promoter for RE05031, -250bp and +50bp []

TTTACAAAGTCCAGTGGTAATATCCAGCAAGTGACCATTCTAGATATTATTCTTATCCAATGACGGCGCTGACATTGATTGCCCTGTCACAAAATGATAATCAGTGTGCGTCTATATCTTTTTACGATATCAGGCTTTATGACATAGATACTTGGGGCGGAAACATTGATTTCGATTTACGATAAAACCCCAAGACTTACGATAAAACGTTCGAGTATAAAAGAAAAATCACTGCCCATATGCAGGCTTAATTGTCGCATTGAAAGTCAAGCACCCACCACCATTATGGTTCGACTGGGTGTTGTGCAGATCCTGGTGATCCTGCTAGCTTGTACTTGCTATAAGCCCAT

>ID_1496 P_1496 Promoter_1512 D1_Promoter for RE15531, -250bp and +50bp [6 ]

TGCAAACTAAAGGAGAAGCGGTTCCTCATCCGAAGTGAGCCGCGCATGCGCACAGATTAGCATAGGCAAACAAAAGAACCGCCGGCCAGCATAACCGAATGTGGGTTATTATGAAATCGCAGCCGAATCAATGATGCTGCTCTGGTCGCTCTGCCCATCAATGGCTTCTAATTGGGCCCCCCCCCCCATCTCATTCGGAGCGACCCATCGATTTTTCGGGGGTATAAAGGTGTGTGCTCCGTCAGCGGTTGGAATTAGTTTGGTCAGCTGTTTGATACCGATTGTATCCCATTCGCTACTCGAGATGAAACCCTTTGCCTGCATCCTGGTGATCCTTGCCGCCATCCTGT

>ID_1501 P_1501 Promoter_1517 D1_Promoter for RH73259, -250bp and +50bp []

AAGTAGACAGGCAACCGCACAACAACAACAACAGAAACAACAACAGCGTACCGCTCACCATTCGCCGCGCCGCGTATGTTTTGGGCCATTGCGATTATTATTGGTGTTGCCGATGGTTTTTGGCCGCCGTATGCTTATGCTCTTCGGCTTAACTTCGCTTCGTGCATCCATTCATTCGGCGGCGTAGTGACTTGGCTGGCCTGCCTTTCGATTGCCAACTATAAAAGTGCGCGCAGGCGCTGTAGTTGGCATCATTAAACCGTTAACCGTCGAACAGTCTACAGCCTAGAGTGGTACGGAACGTGTGGAATAGTGATCAATAGCCACCATCCCCCTCGATAAAAACGGGA

>ID_1503 P_1503 Promoter_1519 D1_Promoter for RE24665, -250bp and +50bp []

ATCGAAATCCTCATTGGGATCCGTTTGGAAGACGGTTTGAGTGCCCATGTCCTCGGTGGAAGCTATGGTTTGGGTTGATTCCGAGGCGATGGCAGCGGGTTTCGGGCGTCCCGAAATGGAACGACAGCTGCAAGTTGCTCCGGCAGGTAGGCGAGTTGCAGTCGCCCCGGTGGGCAGCGAATCGGCCATGGAAGCATCTGCAAAATTAAAAAGGGTAATAAAAGGATTAATCAGTATTTGGAAGAAACTACCTGTAAAGGTTTTCGCATTGGTGGTATATAAGGTGTTGACTTATTAGCAGTAATTCAGTTTGCAAGTGGACAACAAGCAGCACAATGGTAAGTTAAATA

>ID_1505 P_1505 Promoter_1521 D1_Promoter for RE50345, -250bp and +50bp []

AATAATAATAATAATAATCTCCGAACGCCAATCTTCAGCTCTTTAACGATCAATGACCATTGTCAGGGCCCAAAACGTCGAACATATCCGGCTGGGAGTGTCGAACACGTCTGAAGACGTCTTAACTTGGCCAACGGCGTTTCGTCAAATATGTGTTAATGCGGTTCGATTGCGTTCGATTGACCATCGAACTTCTTTCGGCCGCCCTGCTAATTTTTTGTATAAATACTTAAAACGATTTCAACAGCAGCAGCAAAACGCAACCACAGCTCCAATCGATTGAGCAGGTCAGATCCAGATCGCAGAGCAAGTGAAGCAAACATTTAAGTTACAACAAAGATGGTAAGACA

>ID_1508 P_1508 Promoter_1524 D1_Promoter for RH62093, -250bp and +50bp [4 ]

ATGGCCGGCCACCAAGACTGTGCGACTCCGAGTGCGACTTCGGCTGCAACCTGGCCAGGTTCGTGGTCGGGTTGTTTATTTAAATTCTAAGCTCGGCTACTGTGTGCGATGGGCCAAGCGGGGCAGTCGGGGGGCTCAGACCACTTGCGGAGCGCTAAAGCGTCAACGTTGTCGTTGTCGTCGCTGCTGGCCAACGCTGCCGCCGCTCTGCTGGCCGGGAATAAAACTCGATCCGCGTGCGTCTTCTAGCCACAGTTTACCGGTTGCCTTCTGACCGACAGATCGAACACGCCAATCGAGCGCAGTGCACTCAATCAAGTGAACACACACACACGCATCCCCGAATCGCC

>ID_1510 P_1510 Promoter_1526 D1_Promoter for RE27306, -250bp and +50bp []

TTTTTTTTATTTACCCCATTTAGACTGCCTGCAATAATAATAACTAAGTGTTCCCTTTTACAGATTAACATGGTAAGATTATCCTAGATTAAGATTATTACCAACATCTTATTTAAAAGCAACTTTAGCGAGACAGTTAACATAGTTATACTGTGGCCAACTCCAGTCCACAATCTGGTCCAACCCGAGAAATTCGTGTGGGCCGTCGAGCGAGAGCATATAAGATAATGGAAAATCTTGGCGCGACTTAGTCGCTAACCCGTCGGCACATTCGCACTTGAGCCCTCGTCCGAGCCAGAAATGCAGTCCCAATCCCTCCTGCTGATCGTTGCAGCCGTTGCCACGTTTCT

>ID_1512 P_1512 Promoter_1528 D1_Promoter for RE07473, -250bp and +50bp []

AATAGCTTAAGGTTGGCACATTTGTTTATAAATTTTGAAAATAAAAAGTCTTTAAAAATCTCCAGCCTCTGCTATATAAATTAAATACAAATAATCGAAACAATTATTACATTGTAAGTTTACATTTGCTTACATACGTTTGTCAAATCGTAAGTGTACTACGCTTAATAGGTATCTGAAAGTCGAGACATAGTTAAGTCCACGCTTACAGATCGGGTATATAAAGAGGCCACTTTCAGAGCGGATTTCAGTTTAATAATTTAAAGCAAAATGAAACTACTGGTAAGCTAGCTGGGTTATGTGAAAGAAAGTAGAAGGATCTTACGTTCGTCTATTTTATAGATTGTTGT

>ID_1518 P_1518 Promoter_1534 D1_Promoter for RH17287, -250bp and +50bp []

GCATAGTACATATATGTTTGGAGTATCTTTCTAGAATGAGTTGATCAACAGAGGCGCGATGGCTTTATGCTGACTCACGATTACGATACAATTGAGAAAAAAGCCGGCTGAACGGCGAGAATGTTTTGGACTCGGCCCAAAATGGAGACGAGTGGTGAATACTATTACTCTCTCAAATTTGAGCTCGGAACTGATCGGCTCTGCTGGGAACTGCGATGATCTATAAAAGCAAGTGGATCGGCAATGAAAGAGCAGTTCTAGAACAGGTCGCGATCGCAAACCAACCGTATATACATATATATATATATATACATCTCAGTTGCATTAGACCCGTACAAACAAATACTAAA

>ID_1519 P_1519 Promoter_1535 D1_Promoter for RE06169, -250bp and +50bp []

GATAAACATGATTAAAAAGTCAATAGAGATATTCATTCACAACTGGAGTATAATGTTTTCCCTAAAATTTTTTTAACTCACTGTGCCCTTTACAAAATCCAACGTGTTTTTCTAAATAAACATGGTTTAATACGTTTATTAGCTTTCTTAGATTGTAAAGCCCGCGATAAGAGAGATGTAGTTAAGTGAATCTTCACTTTTATTGAAGTAATCTCCGATAAAAGGAGGTGCCATGCGCTCTAGATGATCATTTCTCGTGCAGCATTGCAAGTGTGCAAGTCGGTTGAATAGGAATATTTTGAAAACCCTTAACTCATTGACTTTGAACTTAATTTTTTAAAGAACTTGTG

>ID_1521 P_1521 Promoter_1538 D1_Promoter for RE20037, -250bp and +50bp [6 ]

CGAAGTCAGGGGTGCTTAAAGAAAGTTTTACAACACTAGACCATATTCATGAGTAAAGGGTTGAGTAATAAAATACATAAAAAGTTATAAAAAGGTAAAATTTAGACCAATTTAGACCTACTCATTGCAAACACTCAAAAGCTCCCGGTTCAGACCAAGTTTCAGAGAGCGCAGCTTTGCGGCCAGCTTTAAGCTGTCTTTCGTTGAGTTCGAGCTTTTCGTCAGTTTAAAAAGACTGGCGCCTGCTGGTCAGAAGCTGAGTCGGTAACGGTCTGCGTCTGCGCGCAGTTCGAACAAGTTGAGAAAGAGACCAACAGAAAGCCCATCCAAGTGCAGTGATCAATACGGGT

>ID_1533 P_1533 Promoter_1550 D1_Promoter for RE09471, -250bp and +50bp []

ATTAATTGCCTATTTGTGGTGAATGGTCAAAAATGGTAATTTTTGCGATATGATTATTTAGAAATCTGCCCCACCGGAGGCAGATTGTGGCCACTTCTCATGCCAAAAATATAATCATATAAAAGTTCTACTATTCGAGATTAGGCTAATCTCTGCCATGAATGCGTTTTCCGGCCTGGTTAATGGGGTGGCCATTGGCTGAGAGATAACCGAAAGCTATAAATGGCCCCACACGTGGAAACTATGCTCCAGTTGCTCTGCGAACCATGAAAGCAATCGGAATCCTGCTGGCGATCGCCTTGACCTCCGGGTTCCTGGTGGTCTCCGCCGTAAACTTGCCGGGAGTGCCC

>ID_1536 P_1536 Promoter_1553 D1_Promoter for RE09269, -250bp and +50bp []

TTCGATTAAAGTGATTACTTTAGTGATTTTTATTGGAGTTGTTTCATTGCGCTGAATACATCTTAAGTATATCTTGCTCCGCCAAGAGATGGATTAATTAGTGAGTAACCATGCCTTGCTGTCCGGTTTGGACGCGACTCGACCCACTGTGCGCCGCAGCTGTTGGGGCAGGGCAGGGTTAAGTGAGCCAAGCTGGTTACCGAGCATCCGAGTCGCTGGCCATAAAAGCGGCGGCCAAAACGCTGCAGACCACAGTCGAAGATCGCCCTTCCAGTCCGACACAGTCGCAGTTCTTCCGAAGCAATCAATACTCTCAAACACGCCAACATGAAGGTGCGCCAATCGCGGAT

>ID_1549 P_1549 Promoter_1567 D1_Promoter for RE27077, -250bp and +50bp []

CATTGTAGATAAGCCCTCTACTCAAATGCCGAAAAGAAAATACGTATTTAAATCGCTGATAAGCTAACCAAGCAAAAAGAGCCGAGCTGCAAGAATTCTACAAGCACAGTCGTCGGAAGAAACCCCGCAAGTGGGGTAGTGTGGGTAATAATATTTGGTGTAGCGGCGATGAGAGAGCACAAATGCGAATACGGCATACGACATACGGAATATATGTACATAAAATCGAAACCGCGTGAAACGAGATTTTAGTTGAATTTGAGCAAGCGGCAAACGCGCAACACCCGTCCTGAAGCATCGATAAATCGGATTCGGAATCGGAATCGCCAAACTCGTCGGGTTAAAATACA

>ID_1554 P_1554 Promoter_1572 D1_Promoter for RE63125, -250bp and +50bp []

AGCACTCGCTCTTGGCTGCTTTTGCTCGACTGCTCGGCCTGCTTGACTGCTTTTGCTTTTGCTTTTGCTCTTGCTCTGCTTTTGCTTTGCTTTGCTTGACTTTGCTGCTTTGCTGGCTTGGCGCTTTGCTTTTGGACTTCTCTCGGGCTAAAAGTTTGCATTGTTTAGCCGGCGATGGCAGCGCCGTCGACGTCGCCGCCGTTTGGGCCGTTTTCCTTTTTAAAAACGACGATGGACTGAGCCGTGTTTTAGTTCTGTTCGCCAAACGCAGCCAACTGGACGTGTCCCAAAAAAAAAAAAACAAGCGGCGTTTTTCGAAGTCCAGGGCAACACATTTCCACGGTTCGGAC

>ID_1556 P_1556 Promoter_1574 D1_Promoter for RE09713, -250bp and +50bp []

AGCTCTTCGTAAAATACTATCGTTAATCCTTCATGGTAATAAAAGTAAGTTTGTTAATACTTTTTTTTTCGATTTACATGAGAAAGTTGTTGTACCTGCCAGACTTGATGTCTACCTGATAGCGTTTAAGATTCGGTCAATAGCAGCTGGTACAGATACATATGTACCTACACCTGCTCATTCTTGCTACCTAGCGTATTTTGCAATCTCAATTTATATATAAAATCAGCGGATCGAATCACCAAACATTAGATAGTTTTCAAGCATGGCCCGCCGAACACAATCGCGTTATATCTTTGACATCGAGGATAACTTCCGTGTGTTTCGCCACCAGTTTTTCGTCAACGGTG

>ID_1558 P_1558 Promoter_1576 D1_Promoter for RE18236, -250bp and +50bp []

AGCCGCTCTATCATTTTTAAAACAGATTATACCGTATACACCTATACCTAATAAAACTTTATGACCGCGCAGCTGCTGTTGTACTCAATTATTAGCACAACTTGAATGCTCATAAAATATATGACGGATTAGGTAAAAGGAAAATCCACAAGTAGATTTGTAACACCAAAGGATATATGTACCTTCACAGTCTACCTGTTACCCTGTAGTACTAGTTTGTATATAAGCCAGGTGGACGAAGATAAGGATATCACTCAGCAACTGAATCTTGCCATAGATACATCTCAGTGCTTCCGGAGCAAACTGAAAGAGTAATCATCTATTTGTGCCTGAAATTGGTAAATCGTCCA

>ID_1561 P_1561 Promoter_1579 D1_Promoter for RE11908, -250bp and +50bp []

TCTGATTCTGATCATTCAAATGCTCGCGGACCAGACGGTCGTAGTCCTTTTTCCAGTTCGGTTCCTGAATCATTGGCTTAACTAGGGAATTTCGAATTTTTAAAAACGATAACTGACAAATGCTATCGATGACAAAGTAGTCGTGGTAAAAATATTTAAATAATTGGTTTTAAAAATATTTCGATTCTCGATGATATAGCTATTATATATATCGATAGCTAATTATGAAAGCCGTGTACCATCTCCAATCAGCTGTTTCACCTGCTTTTCGCCGCCTGCTCGTTAGTAAATATCTGGAGTAAAAGCTAAAATTTACCTGGTAGCTCAATACAATTATAAATCACATTCGA

>ID_1566 P_1566 Promoter_1584 D1_Promoter for RE23490, -250bp and +50bp []

AAACATAAACACACCAAGATTTTTTCACAAAGAGAGTGCTTCATAATTTTCTCTTTATCGAACTGCGCTCGCCGGTTGCGAAGACGATCACAATTGGAATCGCGCGGCTCGCAACATGTTGCCTTCAAACCGGTCTGGCCATGAACTTGGTGAAAGAAGCTACCATGGCGAAGCTCCAGTTGGGAGAAAAGTCTGCGCCGCGTTTTCCGCTTGGAAATTTTTAAAAGACGCTGCCATCCAGCGGCCTGGAACATTTGTAATCCGACGTTAGAAGTCTGCGGATCGTGGCCAAAAATCGGAGTGTCCCAGCCAGCTGTCATTCTGACTCCTCCTGACTCTGCTCTGAACTT

>ID_1568 P_1568 Promoter_1586 D1_Promoter for RE15191, -250bp and +50bp []

AAAATTCATATCAAAAGCCACGCACCTGGCGCCTGTTCCCCTAAAAAAAAATGGAAACCGGTCAATAACCACAACAAATTCAAAACCGGATTGGAAGAGAAACGCTGGTCACCTGTGAGGAAAGCCCCCACGTCTGGTCCAGTGTCTGGGACAAGTTTTTATTTCAGTGCGGGTAAAGAACCTCCGAAATTCAAACATTATATGAATTCAAAAAAATCGTATAAATAGAGAAAGACGTGACAGAAAGGGCACAGTATCGAGTGCAACATCGAAGCATCCTCATCTTCTGGGATATTAGTACAAATTGAATTCATTTTCAAATTCAATATGAAATTCTTGGTAAGTTTGGC

>ID_1571 P_1571 Promoter_1589 D1_Promoter for RE15519, -250bp and +50bp []

GGCAACCGGAGGAGTCAAGCAGGGGCCATGTAAACAAACCAAAAAACTTCGAGCAAGGGAAAGCGCAGACGCACGGATAATGTGAAAATTTGGGGGGCGATCGGATAAAAAGCGGATATCCAGCAGCTGGGAACACTCCATCGAGGTTTTTCCCGACGCTTTTCCGAACGCCAACGAGCGGCAAGCCGCTGCCGAAATATCCGCCAACACTTTTGCGAATAAAACTAAAGAAATTTGTTTGTTTTGATTCAGTCGTTGCGGGTTTTTTAAGGCGAACACACAGCCCTGAAAACAAAAACCGGAGACAAACACAAACAATTAACCAATCCAAATTCCGGCTAAAAACTGAA

>ID_1574 P_1574 Promoter_1592 D1_Promoter for RE25411, -250bp and +50bp []

AAGTTGACAAAATGATATAAATTTAAAATAGTGTATTAATTTTAGCTGAGCAGCAAATCCTAATCCATAAAAAAGTTTATATTTGTTTCTTTTGAAGCGCTAGGAAATCCATTACAAATTTGCGGAATATAATGTGGGTATTAAAGATCATAATTTGATATGAGTTTTATTATAGGAATTTAGCAACATCTATTATTTTCGAGTAGAAAACATTTTTAAATAAAAATCATTTTACCAAACTTTGGTTTTGTCCATTTGTGCGTAGTGAACTGCGGTCACACTGTTCTGCTTGGAGCAATTTGGTCGTAGGAAAAACAGAAAGAAATAAAAACAAATCAAATGGCCGGACG

>ID_1577 P_1577 Promoter_1595 D1_Promoter for RH54928, -250bp and +50bp []

ACAGATATAGAGATACAGTATCTCTGTCCTCACCTGTGGCTATATGGCTACATCAAAGGTTGCAGCATCACGACGACGACCAAGATGATGACGATGATGACGACTTAGCTCGGCTGAGCGGCGATTGAGGTAAAACTCGTACTCGGCTGACTCAAAACTTCGGCATCTAACTGAGCGAGCTCCGAGCTTAGCCAACTTAGGGGCTGCTCTGGGCCAGGCCGGTATAAATGAGCCAAGGGAGAACATTTGGGCATCATTCGATTTCTGATTTTGCAACGAAGACGTCGGCTCAAAGGCCCCCTGGAAAAGTAATTTCGAGTGCGGTTCGTCTGCGAAACCGGTAATAGTTC

>ID_1580 P_1580 Promoter_1598 D1_Promoter for RE73239, -250bp and +50bp []

GTTATACAATATATTGTATATAAAAGTATGACGTTAGCCACGTTTCACTGTAATCTTTATACGTTCTCCAAGTTCCGTCAGTCAAATGTTTACGCACGACTTTGCCTCATTTTTTGACGCTCAAACCAAGGCAACAGCTGTCGCGGGGTAAACACAAAACGAACCAAATAAATTGTCACAATGCGCGCTTTTATTTTTCTGCCAATTTAGAGATTTTGCTATAAAAGCGTATTTTCAATGTGGAACTGAACTTAGTTGAAAACTAAATTTGACAATACAATGCGTGCCTATTTGCTGCTTGCCCTGTTTGGATGTGTGCTTTTGGCCACAGTTTCCGCAAATCCGGTGGA

>ID_1593 P_1593 Promoter_1611 D1_Promoter for RE05691, -250bp and +50bp []

GTGGCGTATTTATTCCCTTAAGCCCACATCCGATTCTTAGAGTTCAAATGTGTAATTTTTAGACTAAATTAGATGTTGAACTACGTTAAATAAATAAATATTTGCCCTGGAAATAATACACATACTTCATCATTTAATTGTGTTGATAAGGGTATAAAATTGAGTGCCTCGGGCTTACAAGCATTAAATTCATCTCTTATCTTCCAAGTCTATCTATGTATATATAAGCCTCCCAAATCAAAATCATTTTCAGTTCCAAAATGCAGCTCATCGTGAAATTACTGGTGAGTAAGCTAGTCAATGTAAAAGAACCTTTCGCTAACATTCCATCTCCCTTAGATTGTCCTTTT

>ID_1601 P_1601 Promoter_1619 D1_Promoter for RH48004, -250bp and +50bp []

AAACTATATCACGTTCCCGGAGCTGTTCGAGACCAACCATTCTCATGTCCACAAAAGAGTGCAAAAAAAGTAAATACCAGACTGCCCTGTCACTTTCGTTACTTCATCGAGAGATGCGCTCCACTTTTGGCCCGGCCATTAATTGCATATCCATGCCAAACACACGCCGCACATAAATTTTCCGCTGCTGGCCAAAGCTCCGATTGGCGTTGGAGCTGGAGCTATAAAGCCGCCTGCGCACCTGGCGTGCCCAGCATTTCAGTGATCAACGGTTGGCCAAAAAGTACTACCTAGGTTTACCGTGTGGATAAAATTATTCTCCCCAAATAGTGCCTTCTTTTCGCTTTGCG

>ID_1611 P_1611 Promoter_1629 D1_Promoter for RE38650, -250bp and +50bp []

CAAATCGACGTCCCGTCCCATGCCAAAAAAAAAAAACAAAAAACCCAAAAAATTAAAACACTGGCTGCCAACACCCTAGCCACGAATGCCAGCGAACCACAGCCGTCAGCAGCACAACCACCGAAAGCCAAGCCATCTCTAATAGCCACGATGATCCGGGGCTGGGATCGCGGTTCAGTGCTCTCGGCTTGGGCAGAACCACAGCTCGGGTTGGAGTTCAGATAAATACGGATCCATCGCTTCCGACCACATCATTCGCGGTCCGTCGCCAGTTCGAGAAGCTTTGGCTCGTTGTGATACAGGTGATACAGTGTGAAAGGATACGTTCGGCTTCACCACCACCAATTGGA

>ID_1613 P_1613 Promoter_1631 D1_Promoter for RH70774, -250bp and +50bp []

GTCCAAAACTGCAGATTGCAGTTTGGGTGTGATGGAAAGAAACCCCCAATCTGCTAATTGCCGCGACATCAACGGAAATCTTTCGGATCCCAAAAAAAGTAATGAAAAAACCCGATTTGGCTGAAAGAGCGCAAGAGCGGGAGAGCGAGCCCCCCTAAAAAGCTCTCGCTCTCGCATTTCTGATGAAATTCAGTGGAATTCTCAGGCGGCGGAGAGCGCGGCTATAAAAGCGTTCGCTCGCCAACCGAAAGCATTCATTGTGAAATTGCCACTCAAAATTGAAACAGCGCGGACAAACGGTTGAAGAAACACAAAGCCCAGTGCGAAAAGAATTTCATAAGCAAAAGTTA

>ID_1617 P_1617 Promoter_1635 D1_Promoter for RE15373, -250bp and +50bp []

TTTGTTATCACACGGAGGCTTTGGCGAGTGTTCGCTGTGAGTCGTGCTTTTGGCCATTTAATAATCCGCACCGAAATGAGATGTGTTTTGCATGATTTCATTGTTGGCCATCTGCATTAGCATTCCCCCCGCTCCATGGAGTTGTTTTAACATCTGGCAGATGGCATATGCCTCATGGTATGGAGGCATATCGCACCAATTAAGGGGAGAGCTGCCAACTATAAATGCAGACGGGGCCTCCAAGATGGTCATCAGTTAATCGGTTACGTTCGCTCGGTGCACCTCTAGCTTCCCCAGATTCAGTGACCCCCAGTGAAGATGGTAAGTGGCCATCTCATATCCCTCTCGTA

>ID_1619 P_1619 Promoter_1637 D1_Promoter for RE20374, -250bp and +50bp []

TTTCAAAGGTTGAGGTTGCATAGATAATACGCAATGAACTCATTTCTGTGTAAGTTGCATAGATAATACATAATGAACTATTTGCCGCTTAGAAAAGTTACTTATTCGGATAATAGACGTGTGGGTTTTGCAGATAAGGGCGTTACTATCGCAACCACATACACTTTTGATAAGCATTTAAATTCCTTGACCATAAACATGAAAGCAATCAGTGTGATTATAAAAGCACAGACAATCTTTGTTGCAGCTCAGTTGCTGATGACAGAAGAGTGTCGAAATGAGCTCGGAAAAGTGGGAAAACAATGAGGAAATTAAGATTTTCCGGGAATACTTGCGTATACCAACGGTGC

>ID_1640 P_1640 Promoter_1658 D1_Promoter for RE19290, -250bp and +50bp []

TTATATCTAGAGATGTCGGACCATTGTATTAAAAGAACCACATTATTGCATAGTGTAAATAAATAGTTAGTACATGGTGCAAATGAAAGTAGTGCCAGCGTTGTGGGTCTGTAATTAAGCTTAAGTAAATGAGTGCGTGCGAATTGAAGTCCCCAGGCAAGGACCCATCGGAACTGAAGAGCGCACCCAGTTTTGTGGTGTTCCATCGCGGGGAAACGGCTATAAAAGGACACTCCGCATGTGTGGCGCAGCTTAGTCGAATTCCACGAGTCCTGCTCTTTGGACAATGGCCGAGATCAGGGAGGATGAGGAGGAAAAGAAGTCGGGCATCAGCATTCTGCCCGGTCCGG

>ID_1648 P_1648 Promoter_1666 D1_Promoter for RE21286, -250bp and +50bp []

CCCCCACTTTTTGGAGTTTTTCGTTGTTCCTTTTCGGCATCCACCGCCCCCGCTGCGCCCACGAAAGACAGGTAAAAACAAAGTGCAAGTGCTCCCAGTGTAAATATAAACAAAAGAGGTGAGATGTGATAACGCTCGCTCACTCTCTCTCTCTCTCTTTGGCGATCTCTCGATAATGCCGCGTGCACTGGTGTGCGTGGGTGTTCATCCAGGCGCCTATATAAAGGGCCGTTCATTCACGGCGAGCCCATTAAGTTTTAACGAGAAGCTGCCGCCAGATCCGTCAGGTGTAAAAGTACCGTGAGTCTTCGCCTCTAGGATTTTCCCATTTCTAAAAAGTGACGCGTGTG

>ID_1659 P_1659 Promoter_1678 D1_Promoter for RE52048, -250bp and +50bp []

CACCCCGCTGCAGGCAGTTCTAGCTAGACGACGTTGGTGTGTGAGTTGGCAAAAGCCACCCCCGGGCGGCAATCACGGCGCATATCTGTTGGGATTTGTGTCGGGGTTCTGTGCTCTCTACTTTGCGCTATGGCGCCGCCACGACCGACGACAATGCGCCGAGGGTTGATGCCTGTTAGTCCGGACCCAAATCAGCGGGCTTAATGGCTGGCCGGGCACATAAAACCGGTGCGCACTCCTCGCCAGCTTTTAGTTGGTGCGGGCACGCGAGACGCGAAGAAACAACTCTCTCAAAGCGGAAATCGTAAATCGGAAATGTGCTAAAGGATATACACACCGTGATACAGATA

>ID_1660 P_1660 Promoter_1679 D1_Promoter for RE13893, -250bp and +50bp []

GGGAGTACTTAGCATTCATGACACTCGCCCACAGCGTTCTTGTTTTCTTTTCACTTTTTTTGGTGAAAAACTTTGGCCACTTGCTTTCCGTGGACTTGGCCCGTGGCTCGACTTTCGTTGCTCGCAGTGGCGAGTGCGCCGCGTTGGACTGTCCAAAAACCGAGTTGTGCACTCCAAAAAACCAGCACGGGTCTCTTTGGCGCTTTGGGGGAGCGGCAGTATAAAACGCGAACCAAACGGCGAATCATCCAACATTGTCGCCGCATCGACTCCACAGTGAACACTACGCGCTCTGGTAGTGTCAGTAATATCTAGTTGTATCTTTTTCGATTGGAAACACAATGGCTTTC

>ID_1662 P_1662 Promoter_1681 D1_Promoter for RE41571, -250bp and +50bp []

CTAAACGAGTTTGAAAGGAGCTCAAACATGGTAATTTCCATTACGGAAGAGATACGTTTCTTCGAGGGCAAGGAGATATGACCGTACTGACATTTATAATGATCCCCTATCTGAAGCTTAGTCACCGGGCCAGACTTTCGGCCATCACTATCGACATGCCTACGAATTCTGTCTTTATCAGCTAATCGTTGGCCATCTAGAAGATTGCCACCGTCACTGGCTATAAAAGAGCCCGGCCGATTGTCCGGCCAGTTAGTCCGTCGAACCAAGTGCCGAAAAACGAAACCCCAAACGAAATGTTTAAGCTAACGATGCTGATGGGCCTGGCCCTCATCCTGGCCCCATCAACG

>ID_1664 P_1664 Promoter_1683 D1_Promoter for RH25665, -250bp and +50bp []

CTTAATAAATATGAAGAAAACCAAGTTGGTAATTTACTATTGGCAATTGTAATGCAGATATTGCGCATACGACATACGTGTAGCTCATAATCGGGAACTGCGCTTAAAATTTCAATTATAGACTTTATTTGTTGGTGTGTGGCGAATGTAAAGTAGCATCCAATTATTTAATTAAAATCTCCTGTCTACTTTTGAGCTGAATTAGCAAATCGATGGGTTATATATATAGAGCTGTGATGATGCGCTCATTGTTAGTTGGGAAACAGATTTTCGCCATGAAGGTTGTGTGCAGCATAGCTGTACTATGGATTTGCTTGATAACTATGGTCAGTTCTTAACGAAATAGTTAT

>ID_1666 P_1666 Promoter_1685 D1_Promoter for RE08556, -250bp and +50bp []

TCGCAAGGCAGCGATTAGAACCAATTAAGATACCAATTAAGATATGCATTGACTGGCGATCGCGAATACACTGAACTTGAACTTCGTAGTTAGAGTATCAGGTTTTACCTAAGCTTTTGGTTATCGTTAAACCGTTTTATTGGCCTTAAGCCTTAGCACAAGCCTCTCGAGGGTCTAAAGTGATTGCTTATCAGGCTAAAACCGTGTGGGGCCCTCCTATAAAAGCAGTTCCAATTTCTAAAGAAAGTCAGTTCTGTTCTCGAATCGAAACTGTATTCGCGGTCAGTTCGAAAACAAATTGTAAAGTCGTCGCCATGAAGTATTTGTGCGGCATTGTGCTTTTTGTGGCC

>ID_1670 P_1670 Promoter_1689 D1_Promoter for RE26525, -250bp and +50bp []

TAATTGATCCCTCCATTATTCAACGAAGGTGAGAAATTTACAAAAAGAAATTACGAAAATTTACGAAAAATACGATGACCAAATAGAATACTTTGAATAGTATACCAAGTTCATGTACTAAAACATCATCTCAGTTCATCAAGTTAATGCTCAAATTGTGTTTCGTAGTGATTGCTATGAGACACACTCTTATCGTGACACTGGATCTCTTGACATATATAAGAAGTCAAGTGTAGGGCCAGTCGCACTAGGAAATATAACAGAAAGATGATGAGTCAAACTCTGGGAGTACTTGCTCTGCTTTTGCTGACTTTGGCAGTGAGTTAATAGTAACTTTCCATTTATAGTAC

>ID_1676 P_1676 Promoter_1695 D1_Promoter for RE25803, -250bp and +50bp []

CAAACATTCCCATTTTTTGTGCTCTATCATTTACCTTTTCAAAAATATTATATAGCAAATCCCTAAGCAAAATCAATACATTTGATTGTAATCTTCGAGCTTTTAACAAATGACTTCTATTCATCTTTATTTAACCCATCTTACCCAGTTGAATACCCCTCAAAAACCAATCCGCTTATCTTGCCAGCAAATAGTGATTGCTGCTTATCCTCTGATATTTATAAGTAATCGCAGGTCGACGTTCAGATTCAGTACGAGTTTATGCTTTGTCCAAAGTTGAGCTGTAATGGAAAGTGAGTGGATTGCAAACTGCCAGTGGTGTAAATCTAACTTTATTGTTTTTTCAAATT

>ID_1678 P_1678 Promoter_1697 D1_Promoter for RE73396, -250bp and +50bp []

ATCGAAAGTGCTCGAATCAAGGACTTTTCAAACAGGATCGCAGGTACTCTCCCAATCGCAGTGTTTTTGACCAAATTGCCGGGCCATAAAGTATCGCGTATTTTCATGAAATCCCGTTCGCGGATTAGGCCAGAAAATATCTGAAAAACTAATCAGGTAGCCGCATGCGGTACGGGTGATTCCAGGAACTCCGGGATCCTCCTGCCTCTACCTGCTGGACATATATAAGCCCGCCGGTTTATGGCCATGCAGAACAGTCGACAAGGAATTGTCAAGCGAGTAACATCATACCAGAGTTTACTACACTACAACTTTCAAACGAAACCGAAATCATGGGTTGTTGTGCAAGC

>ID_1679 P_1679 Promoter_1698 D1_Promoter for RE63289, -250bp and +50bp []

CTAAGCTAAGCTGCTGATATCCTCGAGCTTGTTGCTCAAGTTCCTCGAGATCCAAAGACAATTTGCTACCACACACATCCGTTGGCCAAAGAGAGTCTTTTCATCCAAGGAGAGAACGCCGGAGAGAGCGAGTGTCCTTCTGCCCGCCGGGGTGCCTCTTGCGCAACAACCCCTAGCCCCGTAACCCCTAGCCATTAACCCCTGAGCGCCGAGCGGGTATAAAACGCAGCCCGCTCCCCGTACCGCTTCAGTTTGTCAGCGAGAGCGGTCGAGAGCAGAAGACTAGCAGAGTCTAGAAGTACATCTTTTTTTTTTGTGCACCAGGAGCAGCAGAAACAGAACCACACACA

>ID_1680 P_1680 Promoter_1699 D1_Promoter for RE27148, -250bp and +50bp []

ACTTTGGTTGCTTTGTCCGCTATTCCCCGTCCGTTTCCCATTCATAACAATAGCGAAATGGGTGTGACTCGTGGGTGCAAATCTTTTTGGGTAAATCTAGCGATCGGCGGAGAAGGAGGCATGCAAATGAATTACGATCGAAGTGATTTTTGTAAGCAACCGAGTTAAAAAGTGAAAGAAAGGCCGGCGGGGAGACAATGGGCCAACGAAAATTTCGTCTATAAATACAAATGCATCGGCGGTTCAATTATTATTCAAAGCACGCACTCCACAGAAGGAGGACACAGTCTCGCTTCTTTCGCTCTGCAATTTGCCAACCAACCTCAAACGCTCGCCAAATGGCTTTCAAG

>ID_1682 P_1682 Promoter_1701 D1_Promoter for RE28679, -250bp and +50bp []

GAACCCGCTGTGGAAGCCACCTGAAGTCGATATAATTGCCGGTTGCAAACGTTGCTGGCTCTCGGCTGTGAAATCCAAAATGAACTGCCACATGTGATGTGTGATGTGATGAGTTCCCCCAAGCTCCACCGCATCCACAATTATCATCACCATCAGGTCTAATGGCTGCACACCACTTTCCTCACTTTCGTTAGCAGATCAATTCGGATTTTCTGGGGTATAAAAGCGCCGGGTCGAGGTTACTGAGGCATCAAACATAGTTTAGCTCTTTGGGTCAAACCAGCAAATAAACCATAAACCAAATCTCAACCTAAGCCAGGCGAAAATGTCGAAGATCGTAAGTCAAAGGA

>ID_1684 P_1684 Promoter_1703 D1_Promoter for RE27818, -250bp and +50bp []

CCAATTTCTCGCTTGTGAACCCAAAAGACACGTGTCGCAGACTTCTCTCTGCATCCGTATCCGTATCTTTGCTCTGCGCCGCCTTCTACTCGCTTTTGGCCAAGTTCATCCGAAGCTCTTCGCGTCTCTTTTGATGCTTAATTAAATTCATTTTTTCGTTTATTTCGTTTACCTTTTGTAATGAGACTTTGAGCACTCGGAGCTGCGGACATCGAATCATATAAATTCGGACCGCGGCGGCCAAGTCAGCATCAGTCGAGCATCGAGCTTCTCCATCGAAGCATTTCATCGAGCCAAAGGATTACGGGCTTACTAAGCAGCAAAGGATTTCCGATTACCAGAAGCAAAAT

>ID_1685 P_1685 Promoter_1704 D1_Promoter for RE05911, -250bp and +50bp []

CTGAAACGAAATGGAAATTGACGAAAAAAGAGTTGCTAATAAACGCGGCAAATGGAAAGCAAAAGCGGTTAAATTAAAGCCAGCGGCTGCTCACCGATTTCTTGTGCGTTTTTCCAACGGTAGTGGTCTTATAATTCGCTGGTGGATCCCAGTTGGTGCCACCGAGCCGCCGAGTGGCGAACTCGTTTCCACCATCCAACATCCACGCAGTCGAGCGTTTTATAAATAGAAGCCACCGGCTCGAGCAGTCGGCACTCGATCACGAAATTTGAGCGCAGCCATGTCGTGTGCTCCTCACCTGGTGGCGGGATGATGGTGCCGGGAGGTCCTGCTGGCACCGTCATCCCACT

>ID_1686 P_1686 Promoter_1705 D1_Promoter for RE44034, -250bp and +50bp []

GGGAACACTGCATCATTAGGCCGGTCGAAGCTGTTGCTAGCATTCCGTAAACAATAAAAAAGTAATTTAAATTTGCTTAGCGCATAAGTAAGTGGGAATCGGAAATGTGCCCAGCTTCCTGGTGACAGCATTTTCGGATCCAGAAGACCCACTTTCCCAACTCAAACAAAAAGCATTCGACTCGTTTACTCAGCCGCCCAAAAGCTGAACTTTGGATTGCCATAAAAGCCATGTCCAGGACAGTGCAGCAACATAACGCATTCAACAGCCATGTCATTGAAGTGGCTCTTCTTCATAGCCCTAGTGGCGCTGCTCAGCATTGGTACAGGTTCCCTGGCCCAGCCCAGCTG

>ID_1688 P_1688 Promoter_1707 D1_Promoter for RH04437, -250bp and +50bp []

TAAAGTGGTAATTCGATTACTCTGGAACTGGTACCACACATTGTTCTGCTCTTGCTCTCGTTGGCTTTTGTTGGCTTTTGTTGAACGCCCGATATTGCTGGTGTTGCGGTGTTTTCGCTGCTGTTACCAATGTTGCGCATGCGCCGGCGTCTCAGCCGGAGTCTCGGCTGCAGTGTCTCTGCTCCCCAATCCCCAGTCTCCGGGCTCGCTGGTCTCGGGCGGTATATATTAGGGCCGCCAGCCAGCAGTCAGCACAGTTCGTTAACAGTCCTAGTCGTGGTTCTAGGAGGTTCAACCAGCGTCCAGCGACCAGTGCCTGTGCCCGTGTGTCGTGTGTAGTTAGTTTTCCG

>ID_1692 P_1692 Promoter_1711 D1_Promoter for RE63144, -250bp and +50bp []

CAATACGCATTGCTAATTGGCGTGCCATTTGATTTGTGGATCTAAGTAAACACTTAGTCACTGTTTATGTCGCTTCACAAATGAAGCGTTAAAAATTCAATTTGCAAGCTCAGCTTTCGGAGCTTTCGAATTTGGCCAACTTGCTGGTATGGCAGTATAAGCTCGGCTTACAAAGTTGTCTTTGCAAAAGCTACTTCACTACTTTTGATCAGTTTTTTGGTATAAATAGGAAGTTGTTTTAGAATTTTCAGCTCATTTCAAGTTTTTCTTTTCTGCCGCTTGTGTGTGTGTATTTCTGATCGGTAGGTTTAAATGATTAAGCAATTAGTTTACTTGGCCTAAACGATTTT

>ID_1695 P_1695 Promoter_1715 D1_Promoter for RE16136, -250bp and +50bp []

GAAGCTATAGCTTGTGCGAATGTAAGCGTCGCAGTCGCCGTCTATCGTGTTGGCCAAGCTGCGTTTGAGCCGCAAAAGAGCGGCTTAGAACCCGTAGCTTAGCCAAACCAAAGCCGAAGTCGTGCGAATTTACAGACGCTTGCGCCGGCAGCGACGTCTTTTTGGGCAGAGGCGCAAAAAACGCCAGCTCTGTAATAGCCAGCGAAGAGCGGAGAATCTGTATAAATACCAAAAGGTTCTGCGTATTCCTCATTAGAGTTTCGGTTTTACTGCTGTGAACAAGCAACTCATCGGACAATATGGTACGATTCGACAATTTAGGAGCCATCGAATCGCCACTGAAGGACTAA

>ID_1697 P_1697 Promoter_1717 D1_Promoter for RE42506, -250bp and +50bp []

CTAGGATGGTTATGAAATATAAAACGTATCATTATGGATACCAATAACTAATGTGTAGAATCCTAGAACTCCATATGCTCACTTCACCAGTCAAGTCATACAAAGACAGCCCTCGAAAAGCAAGAAGTTGTCTAGTGTAAACGTGACCTCAGCGGAACTTTAGTACAGTAGTTACAACATATCTCAAACGATTTCGATTTAGCAAAATGCAACCCTTTATATACAAATCGTTAACCGGAAGTAAAGTCGTTTTCCTTTCCTTCTATCTACTTGAACAACAACGATCAAAATATAGGTTCTGTGGATTAATATATCTGAAATTGGATTAATGTAAGTATTTCTATTAAAAT

>ID_1702 P_1702 Promoter_1722 D1_Promoter for RH61519, -250bp and +50bp [1 ]

TGTTGCCGTGTTGCTGTTGCCCTTTTGCCACGTGTTGCGTATACGACCAGTTGGCCAGCACCCAACGACCATCCCATGACTGCGCACAATCCCCATTGAAATCGGCCCAAAAGCAGCAAGTATCACGTCGTGGGCGGACCCGAGGACGTGGACCGAACTTCGGAGTTTCCACTCGGGTGCCACCTGAACGCGTTGAAATCTTTCGTATCCTGGACTCGTATAAATCGCGGTCTCCTTGGCTGCCGATTTCACTTGCGTTTCAGTCTCAAAGCGGTGCAGTCAGCAGCAGTAACGGTTAATATTGTAAAGAACCGATTCCAAAAAGTTTTTCAAAATCCCATCGAGCATCG

>ID_1708 P_1708 Promoter_1728 D1_Promoter for RE35237, -250bp and +50bp []

CTGCTCTTCCGAGAGAGAGGTGGGAAATGGAGGAAGAGAGAGAGAGAGAGAGTGAGAGAGCAGGTAGAGGGAAGTGAGGGAAATACGCAATAAGGGTATGGGAAAAGTGCTGTTGTTGTTGCTAGGTAGCGACGCACACGTGCGAGTGTTTTTCTGTTTTGAAGAAGAACCACCACCAAATGGCGACAGCGGCGTCGGCAGAGGCGCAGAGTTCCGGGTATAAAAGAGCGTGCTCGACTGTTGACCTGTCACAGCCACCTCAGCTCTCGTTGAGAACGCAACCACCGCTCTATACTCGATCCCGAACTATATAACTCGCCTCTCGATCGCCGATCTCCCGATTTACCCAT

>ID_1710 P_1710 Promoter_1730 D1_Promoter for RE40129, -250bp and +50bp []

GACAATGGTCTTTAAATCCCAAAACGATGAGTAAACAAATAGAAATGTTTAATATGTGACCAAAAGCGCTCTGTATTGTAAAGCTTTAAAACGTGAAGTGTATATATATTACGTATACGACAGCTAAGAGATATGTGGGTTCCAAGCATTGAGTATGACATACTGCAGAGTCATCACAGATGTGCAAAGCTCTCTTAAGTGAAGAGCACCACCTGGTGGCTATATAAAGCCCCAAGTTTCGACGTTTAGTAAGCAGTTACTAGTCAATCGCCTTACAGCACACTACAGACATGGGAAAACTTACGCTTTACGGAATCGACGGTAGTCCTCCGGTCCGTTCGGTGCTGCTC

>ID_1715 P_1715 Promoter_1735 D1_Promoter for RE41896, -250bp and +50bp []

GCATCTTCCACCGAAAAAAGCCCCACTGAAAATGCTCATTATACCGCAGAATCCAAGGCTTCTGGGCAATGCGAAGTGCTCTCCGCTGGGCGTTGGGAAAAAGAATCCACTGAAAGTGGGAGTAATTTTCTGAGATTTTCTCAGCGGCGGAGCGAAAAGCTCCCGGCTCAGAGGCGCAGAAGCGAAAAGCTCGGAAAACGCGCGCGATTTTCTAATGCCGTATAAAACCGCAAGCTTGACCACAGCCGCTGTTCATTCGCGATTAGAATCGCCAGCGCCGCGCATCCAAAATTTCAAATTCGTCTACGGAAATCGTGTTCGCGTCTGCGTGTCCTGTGTGCGAGCGGGAT

>ID_1717 P_1717 Promoter_1737 D1_Promoter for RE38860, -250bp and +50bp []

CCTTGGAATTATCAACGTTGTCAAAATGTAAAGATTGTAGATAGCATATTCCAAGAAAACTCCCCCATAAAGATGACTAACTGTCTGAATAGTAGTTTCTTATCTCTTGTCCAAATTAGTTACTAAAAGATCGTAGAGGGGATTTAATCAAAAGGGGTTTATCAACTGGCTGGTCGAAAATGACCAATAGTAAAAATATGATAAGAAGTATACAACTGATATAAAATGGTTTGTCTTGGCCAACTGATTTGATAGTCGTTTCGAGCCAACACAATGAAACCCATTTGCTTCGCACTCTGCGTATTTTCGCTAGGCCTTTTCTATTTGGCCAGCGCTGAGATTGGTGAAAA

>ID_1721 P_1721 Promoter_1741 D1_Promoter for RE44773, -250bp and +50bp []

CTTAGACCACTTTTTCCAGGTATTCCCGGTGCGATACGAACCAATCAACAAGAACCAAAGCTCCGAAAGCATAAAAGCTGCCTGTCCTCGCACGAAACTTTCCCATTCTAGACTTTTAGTGGCTGGCAAGGCGTTGCAGGCGGCGGTACGAGAATCGGTCGCGTTTACAGCTGAACGGCGAAAATACACATATATGAAATAAACAATCGCTGCTGCATAAAATAGGTTTTTGAGGGACACAAGAGTGCCAGTGGCAGTGCTGCAAACAAATATTACGCATACGCCGGGTGGGACGGATCGCGGTACTGCTGATTAACACGGGAATCTTTGCAAAATGTGAGTGAATTGAG

>ID_1724 P_1724 Promoter_1744 D1_Promoter for RE45643, -250bp and +50bp []

CCAGCTGTTCGATGCGATTAGATGGAGAGAAAGAAAAACATTTTCCAGCATCCGTCGAGCCAGAGCGAATTTCCCTTTAGTCATACTCTCCGAAAACTCTCAGCATTTCCCGCCCGTGCAGAGCCTGTCGGCTGCTCTTCGTCTGTTTTCCAGCCCCCTTCGGCTCGCGTTCGGCTTTTCGGATTTTCGGGGTTTCGAGTTCGTGGACGACTCTCCATTTAAAAGAGAAGTTGCGCACGTTGCGGCAGTCAGTCGAAAATCAGCCAACGTGCGCGTTACATTACGAACCGGAAAATATCGACGCTGCTGCACCGCTCAAGTCCCAGAAAAAGTGGGTTCAGTTCAGCCAA

>ID_1728 P_1728 Promoter_1749 D1_Promoter for RH08259, -250bp and +50bp [8 ]

ACAACAAAGACTCATAATAATCTGTTGGGCCAATCAAGTAAAATATGCGCGAGATCAGTCAACTACAGAAACAAAAGCAAAAGTAAAGCAAAGCCACTGCAGCAGCAGCAATAGCAAAAGCAACAAGCACAGCAGCCGCAGTAATGAAAGTGAAACCGAGTCTGGCCGAGAGACTCTGGCTGAGATTGAGACCCGGCCAAGAGTCGGTTCTAGCCAGCACCGCTATATAAGCTTGATGGCCGGGCTCGGCAGCAGCAGTGCAGCGCCGACCAGGAACCCAATTGGAAGTTTGAGCTACGACTCCATAGTCCAATTCGGCAAGGATTACCATAAGCCCCACACCAGAACCA

>ID_1730 P_1730 Promoter_1751 D1_Promoter for RE21944, -250bp and +50bp []

CATTCGTTACTAGTTCGACTTCTTTGGTTGCAACCCATTCCTCATCCGCAGTGGTTCCACGTAAAGTGTCAAAGTTAAGTGCCCGCGGGTTATAATTAACGGCCAATGGTTATGGCTTAGACATGGGCCCTTGACTAAATATTGTCACGTCCGTAGGCCAACGAAAGGGTGTCCCTCGGTGAGCCAAATATACCCGAGTGGCCGAAAACTTCAGTTGGTATAAAAGGCGCGAAACTGGCAGCCACTGACAACATTCTAAGACAACTCAGCAGCAGCTCAACATGGCCCACAAGTTCATCTGCTCCCTGCTCCTGATCGCAGCCATGGCTGCCGTTTCTGTTGCAGAGCCT

>ID_1735 P_1735 Promoter_1756 D1_Promoter for RH23846, -250bp and +50bp []

TAAGTCCCTGCATTCCTGGTAACTCTGAGGTCTCAACTCGCAGTCAACGAAAAATCACTTGTTTATTGTTATCAACTTCGATGCCATTTCCCGGCAAGGACATGCGCAAACACCCCCACTTCCGCCCACATCACTTGGCCATCATCCCCTCTGCGACACCAAGGATTAGGGAAAGGAAAAGCAGAAGGGACACATCTCCGAGTGGAGCAGAGTGGCGTATAAATACCACGCAGCTCGTGCGCACAGCCTCAGTTGGCGGACCGTGTTGCTCGCGATTCCCTTTTCCCAGTCGCTGCGTCCGCAGGTGAGAGTTCATCCGGGAAACGGCCAACTGTTGCCAGTTGCACTCG

>ID_1746 P_1746 Promoter_1767 D1_Promoter for RE62495, -250bp and +50bp []

TGCTCCCAATCCCGCTGCCATCTTTTCGCTTTGCCCCTCCAATCCAATATTCATCTTTCCGATCCGCCAACACCGTTCAAGAAATCGGAAAATCGCATCGCCGTGTGGCGAACATCGTGACCACCACCGATTATCGTGAATCTTCGTATGGCGTTCGTTTCTGGCGAACTGGCGATCGTCGACAGCTTGCCGAATTTTCAGCTTTTCGCCGACTTTTCACATAAAAATCATGTAAGGCTGGAAAACGGCGACAGTTTACTCACGGCAGTCGCGAAATAAACGCTGCAGGCGAACCAATCCGTTCACCCGAAAGAATTCAAGACCAGAAACTTAACCACAGATCGGTGGCG

>ID_1753 P_1753 Promoter_1774 D1_Promoter for RE59335, -250bp and +50bp []

GGGTAGGTAGGCACACAGAGACTGAGTAGGAATAGAGGCACCCACCACAGAAAAAGAACCCCTAGAAAGAGAGGAAAAATGTACGATCACTTGTGCAAAGGACTTAGGTCCCGGTTTTTCGAGGGCAGGTAGCCAGGATCCGACCCCGTACCAACCCCTGTAGCTCCTCTGCCGAAGTCGCTGCCTCTGTCGCGGCGCGTTTCCCTCTGCCACTGCCGGGTATTTAAAGCCCTAGATCAGAACAGCAATTATCATTGCGGAATCTGATTCCACACAGTCAACATCTGTAAACTAAATCTTAGAAAACTCTCGCAAGGATTACCATGACGAGCATTTGCAGCAGCAAATTC

>ID_1757 P_1757 Promoter_1778 D1_Promoter for RE65113, -250bp and +50bp []

ATCGAACAGAATCAAAGACATCCAACACATGCACATCCATTGCAGGGTCGCCACGTTCTGGAGGAGTGGCACCAGCCCAATTTTGGTATTTCTTTCGATTTGATTAATTCCCCCTGCTTCGTTCACGGTCATGTGCTGAGGTCGCCAGATAAGGCCATAGCGCTTAACTGTGCATTGCCATGCTATGGAGGCAACTTTTCATTAGTTGGCATTTGTGTATATATTCGCTATCAGGCCGCCAGGTGATCCACTATGCTATAATAATAGCGTCGAACAGTTGGCTGCAAATTGTGGGGCCTAAAATAAGATCTGCCCACAGAAGATATAGAGTTTATAGTTATTGCTGTTCT

>ID_1758 P_1758 Promoter_1779 D1_Promoter for RH05289, -250bp and +50bp []

ACGTGTTTTTTTACTTTTATAAAAATAGTGTGTTCTAAAATAATAATAATATATACATTGATTATAAAAAAGGGTCTGGAATCAAATGTTTGCTTTAGAAAATCATAGTGTATTTTTTGTGCATCAAGTGAAATAAATGGCGAACAGTCTTCCTTAAGAGCTTAAAAGCTAAAGTTGCCGACGAACGCCTTCGGCTGTCGGCTTTGGTTTTCCATGCAAATATAAAAACCGAACCGTCTGGGTAATGGGAAATCATTCATAACGGAAAACTCCTGGCGTGCGGACGTGAAAGTGCCGTGATTTCTACGTTGATTTCTGTCGCGTCTTTCGCTGCTTGTGTTTTGTGTATT

>ID_1765 P_1765 Promoter_1786 D1_Promoter for RE55542, -250bp and +50bp []

CTGAATTACAACAATTGGCCGTCGCGTTGCCAGCAATCATAACCTGCAGACTTTAATGCAAATTTGCGGAAACTAGCACCACCGAGCGTATGAGTGATGTGTGCCCAGCAGAAAAGCTGCTCTGAATGTCCGCCCATAGAGTATAAGAGTTGCAATGCAGCAGGAGTCGACCAAGTTGATAGGTTGTTAGGCCAATATAAGGCAACAAACAACCCACTCTTATAAAATTTGGTGCGCCTGGCAAACGGAACGCAGTCGCAAAACGGCAACCGCTACGCTCGGACGTGTCGCGCGATCAGTAAAAAGAAAAAGAGCAGTAATTTAGGATTAAGTAAAATATAAAGCAGCCA

>ID_1769 P_1769 Promoter_1790 D1_Promoter for RH68619, -250bp and +50bp []

GAGTAAAGTCAACTTATTATTTCGTTGCTTATCGGCTAAATTTGCTAAATTTAGAACTTAGAACTAATTTAATGTTAAATATGGTTTTAAGAATTTAATATGTTTGATTAAAAATATGATCTAACCAAAAATAAATTTATTTTTCTTTTTTTTTTTTTTTAATAATTTCCTAACACATTTGGCCACTTTTGTGGGGTGCGCAGTGAGTGCCCGCTGAGCTACTATTAAAACGACGCTCGTTCGTCGGACCGCGTTCATTCTGCTTTGGGCGGTCGAGGTGTTCGTCTCGCAGTTTGGTCAAAAAACTGGAAAAGCTACGAGAGCTTTGTATTTCGCGCTGAACACGAGCG

>ID_1772 P_1772 Promoter_1793 D1_Promoter for RH19621, -250bp and +50bp []

ATAAATAGGCTGAGAATTTGCCCACCCACTATTGTTTGAAATCTTATCGGCTTCGAATCGCGGTCAAATGTTCTTTATACGCAGTCATTGCAATTGTGCTGAAACCTAATCCTTCGGTTAGCATAACGGCTAATTACTGGGAATCCCAAAGTAAATAGAAATTTAGCTTAATTTACACATATCTTTCTCGCTAAGCTCCCCCGTGCCAGGTTCCAATTTTCCTTTATAACTGGCGCCCCCATAAACAGCACATAGCATAGATAAGATCTACCTGTGCCAAGTTATTTGGGGTCTGGGTCGCAGATCATTGAGTGTGGAGCTCTCGACGCGAACGGTTGATCTGGTCTGAT

>ID_1780 P_1780 Promoter_1805 D1_Promoter for RH09340, -250bp and +50bp []

CTAGAAATATGGTGCAAGTGACGCATTAAGAGCATAAAGGAGACCGAACTGGCCCACCCAACCCACGGCCACCGAACCATAGAGCCAGAGCCATCAAGCCATCGAGCCACTAAGCCGTTAATAACGGGTGCGTTCCTGCGGTCTTTTAGCTGTCGTCGGTGGTTATCTGCATCATAAATTTGCCATCACCCTGTTTAAGTCAGAGGATGTTTGCCCCGAATCGGGTATAAAACGAACTGCGGCGCGGCAAATCGGCACAGTCATCTTCAAATCGCTCCCAAGTGCACCAGGCTAACGGTCAACGGCTGGAATCATGAATCTATATGTGCTCCTGGCGGTGGTATCGGTGT

>ID_1786 P_1786 Promoter_1811 D1_Promoter for RE69086, -250bp and +50bp []

CGTTTATTAAACAAAACGAATTATGGGCTGCCAGCGGGCCAAACGACATGCAAGTGTTGACGAAGGTCAAGTTTAGCCTTTGCCAAGCGTTGGTTTTCCCAACAACAGACGTCAAAAGAGCAAAAGAGCAGTGGGAAAACGTAAGTCCTTACGTTATCACGTAGACGGAGAGAGCTCTCGTGGTAACACGGAAATTTTCGCTCTCATTTTCCTTCGTGCGGTATAAAATCCGAGAGCGCAAACGAAGTCGATTTAGTTTGATGAAGACTTCCACACGATACGGTCGCCAGTTTTTCCGGGCTTTTCCGTCGCGTCCTCGCCTCATTTTCCGTAGCCCGCTTATCAGCGCA

>ID_1787 P_1787 Promoter_1812 D1_Promoter for RE57663, -250bp and +50bp []

AAACTATTGTTTGGCTTTAATTCAGTGATACTAGAAGAAAGAAGATGTTGATAAGTTCTAAGGAATACTCTCCTGTTGCTCTAGAGAGAAAACTCATTTGCTTTGCCATGCAGCAACGTTTCGCGGAAATTTGAAATTCAATTCGTATCCGATAAGCCCTCGCGGGCGATAACGTTTGGGGAAAATCGGAGAGACGCGTCCATTTCTAGGGGATTCTCGATTATAAAAGGTTGCCAGCCCCACCGTTGGTGCTCAGTATTTGTTCGTTTGTTGTTACGGCAGCCGCCAGTCGGTCGTTAGTTTTTGTTTTCGTTCATTTGTTTATACTCGCGCGGCACTCGGCCCCCCTC

>ID_1788 P_1788 Promoter_1813 D1_Promoter for RE58951, -250bp and +50bp [5 ]

AAGAACATCTTAAAGCTAAAGCTAAAGTCAAAGATGAGGATGAGCAAGATGCCAAATAGCCGAGACAAAAGTTCGGCTAAACAAAAACTAAAGCTGAATCTAAACGCCGAACTGAAGATGAACTGAATACAGATACAGAAACAGATACAGATACAAAAACAAATCGGAAGGCAGAGACGAAGAAACAGCAGTGCCAAATCGAACCTTTAGTCGAAATTTTTATACATAGCGCCGTACAACTCGGCGGAGCAGTTCAGTGCGCGTACAAGTCTCGTGGGTAGCGGATGCCAGCAAAGGTATCTCCCCACTATACAATAATATACCATACTAAACTATACTATACCACACCA

>ID_1789 P_1789 Promoter_1814 D1_Promoter for RH62559, -250bp and +50bp [2 ]

AGCTGGAGCGGCCAAAATGCAGCCAGTGCCACCAATTTGGCAATTTGGCCACAAAACACTGGAACTGGAACCCTAGGTTGTGTTTTTTTTTTATTTATTTATTTATTTTTTTTTTTTTTGGTGGCAAAGGGGGCTGCTACGCACGCCGCGATATCGGCAATATTTAGCGAAACCCGGGTCGGGAAACCAGTTCCGCGGGGCGAGGGCATCGAATCGGGCCGTATAAAAGTGAATTGTCACAAAAATTCTGAACAGTCGAAGCGCACGCTCAGCGGCGACAGCAACGAGAATCGAAGCCAACCAAAGCGAATCGATAGCCCCGGGGGCCAACAAGGTGTTAAGCGATCACT

>ID_1790 P_1790 Promoter_1815 D1_Promoter for RH02452, -250bp and +50bp [6 6 ]

GACCATTTGCCGACATTGTCAAGGTTTTTTTTTCAGCCCCGAATACACCTGCTAATCTGTGGTGGTGACTAGCGCGCTCGGAAGGCAGAAAAAACTGCAGCGAAAATCAGTTCCATTGAATGTCCTCTGGCAACATGTTGCCGCAGCACTTGTTGCGCGCGTCCACTCGCAGAGGCACCTCAACATCAGCCTCCTTGGGCGCCAGCACAAAGGACGCAACGCATATAAAACAGGATGTTTCGCCAAACATCCTCACAGAACTGTTCCACCTTCGAGCGGGCAACAAGTGTGTGTGCGGCCCAAAAGGATCCCCAGACCTTCGAATTCACTCTAGTTTCCTAGTAAGGGGA

>ID_1791 P_1791 Promoter_1817 D1_Promoter for RH43577, -250bp and +50bp []

GATATCACAAAACGACAGGCCTGTGCAAAACCAAAGTAAAATATTAACCAATAAACTGAAGATTAAGTAAAAAAAAAGATTCAAAACAAGCGGTTTCAGGTTTGAGTGCACACTACTTGGCGCCTCTCCTTCTTTGGGGGCCGGTTGGCGTTGATCGCGAATAAATAAATATACATACAATATATTCTCGGCGAGCAATCCGATGATCTCGGTTTCCTCTATAAAAGCGGTCTTCTATGCGCCGTGTTTGCTCAGTTTGGAACAAGTGTTTCGAGGGAAATGCAGTCCGTGAAAGTGATCTTTGTTCTGTCTGTGACCCTGGCGGTTGCCTTTGCTAACGTGAGTTCGAG

>ID_1793 P_1793 Promoter_1819 D1_Promoter for RH26067, -250bp and +50bp []

TACAAAAAAAAAGTTCTGTCATTTGAAATCCATAACGTTTACAAAAAATCCATTGGCTTCGAAAAGTGTAAGCATATACTAAAAAGAATTCGATTAAATATAGCATATTTTTATACGTTGTTTTTAAGCAAGCTAAAGAAGTACCAGCACTGCCACTCGCTCTTTCTCTCGCTCCCTCTCTTGCTGCGGAATGCGGAAGTCAACTTGTGTGCAGGAGCATATAAATAGTGGGACCAATCGCAGGACTCGCTCACTCTGCTCGGAGAGCTTGGACTGGACTGTCAGCGCTCCGGACGACGCGCCTTAAGTTAGGCAAAAACAATTTTGTTATTTGCCGCAGTATGGACTTG

>ID_1796 P_1796 Promoter_1822 D1_Promoter for RH01578, -250bp and +50bp []

TTCTGATTGCCATGGGCAGCGAAAAATGAATTATTTTATATTTGTCGCGATCGATGCGAGCATTTCGCATAGGCACAGGCCGCTACAGACTCCACACGCATGCTCACTTTTTAGTTGTTTATTATTATTTTTTGTGTCTTGCTGCGCTTTTTGCATAAATATCTGGTGGCAGGCGGCGGCGGAGGCTGTGGCGGATTTTTGGGCTCGCGTCTATGTGAGGCCTATAAAAAGCAATTGTCGTGCGGCCACAGAACCACAGATCGAGTTCATTTGTCAGTCGAGCAGCAGTAGACCTCCAGTTCTCCACTACTCCATCGCAAACACCAAGCAATCCGTCCAGAATGGCACAG

>ID_1801 P_1801 Promoter_1827 D1_Promoter for RH65683, -250bp and +50bp []

AAAGCCAGTTCCAAGAAATGGCGCAAATATTCCTGGGATCCACTTGTGACGTAGAGCCCATTTGTTTACGCTCCACTAGTGGAATGATAAAGCGTTATCCGACTGATAACCGGCTCAAAGGCCCCACGACGATGCACTCACTTGGGCACTCGGGTGCTTAAGTCTGAGCCCGACTTCGGATCTTGGCAGTTCGATTGCGGTCGGTGCTGTAATACGAGGGTTTATATATAGCGCGATGAGGCGCGCGTCGTCATTTAGTCACGAAGCCTTAACCAAGCCGCATCGAAACGCGAATAGATCGAAGCCTAGCCACCATGTTTCAGAAAAGGATTATCGAGCTGATCACGGTG

>ID_1808 P_1808 Promoter_1835 D1_Promoter for RH63918, -250bp and +50bp []

TCGATCTCTTAGCTCACGCAGGCTGACATCCGTACCCAGCGATATGTTGCCAAAGTAACGCAGACGCGGATGCTCGGCGGTCTTGGTGAAGGTGTTGATTACGTTCTTGACCTCCGGATGATCGGGCGCAACGCCAAAGCTGCAATGTGCGATTTGCATAATGGGACAAGTCGTTAGTCGATAAGTGCGATGGAATGCCGTGCACACGCAATCGTGTGCGGATATAAGCATTTGATTTCGGAGTGGTTGTCACAGTTTGTATTCCAAGATGCAGGTGCTAGGCGGCAATCCACTCAGCTTGTGTCTCTTGGTGATCATCACTCCGTCACTGATTGCAGCCAATCCCCTGT

>ID_1813 P_1813 Promoter_1840 D1_Promoter for RH48406, -250bp and +50bp [0 ]

TTGAGACGATGATCCATTTGGACACCTTTGTACGATCTGGAAGAGCACACTCTCTGTGGAAACTACGCATCTGATCAGTCCGCCTCCCTGGCAATTAAGCAATTAGCAAGTGCTCGCGGAATCCCACTCAATCAAGCAGCTGAAAATGCAAATCACCACTGACTACTGACTGGCTGATTCGCAAACGAGCCGCGCTCCAAGTGGCTCTTTTGGGCAACCTATAAATAGGCAACGGGCTGGGCCAGCTCTCGGTTCAGTCTCGTCACCGCGCTCCAAAGATGCACAACCGTTGCGGATCCATTTGGCTGCTGGCGGCCGTCCTGCTGCTGCTGGCGCTCCTGCTTCCGCAG

>ID_1815 P_1815 Promoter_1842 D1_Promoter for RH07954, -250bp and +50bp []

GCAACATAAATATTCAGCGAGAAACGTCATATTTACATTTAGTCTAGGCTGATAATCCGGGACCGTGGGAAGTCCCCTTTGGGTGGTGCTGGCTGGGTTCCCCTGGCCACAATCGGTTATCTGCCCCCGGCTGACACTTGCCCGTCATTCATTCGGCTGCTTATCGCAGAAGCTCAAATAAAAAGTCCCCAATCTGCGACTCGTTTGTCTGGGACTGAGCTATAAAAGCCTCACCATCTCAACGCTCAAAGCATCAATCAATTCCCGCCACCGAGCTAAGATGCAACTTAATCTTGGAGCGATTTTTCTGGCCCTGCTGGGTGTGATGGCCACGGCTACATCAGTGCTGG

>ID_1821 P_1821 Promoter_1848 D1_Promoter for RH31195, -250bp and +50bp []

AAATACGAAATCACATCTTCACATCTTGGGCAAAACCCACAGCCTAAAACAAAACAATCTTGTAACTTAAAAGCCAAAACAAACAGTCGGCAATCTGGTTTCGCAAAATGGCAAACAAAATAAAAACCATACTCAACAATATCAAGGTTTGTTGTTTTTGTCAGTGCTCAAGAGACCTGACCCTCGTCGATTGATAACCGATCCGGTTTGTGCCTATAGTATATAAAACGACCAACATCTCGGATCGGTCAACAGTTCGGTTTGAGATTTCAGTAAAGCAAGCCCAAGCAAAATCAATATGAAATACCTAGTCGTTTTTGCACTCATCTGCTGTCTTGTGGCATCAGCAT

>ID_1828 P_1828 Promoter_1856 D1_Promoter for RH34103, -250bp and +50bp []

CCTGCGGCAAGTCAAAAGCGTGCAGCACAAACGACACAATCGCGAAATCCCATCAGACGGGCGCACAGTGAACGGCATTTGTCGACCGCGCCCCTGGCCGCCCACGGCCACGCCTCCTCCGCCTGCGCCGCTGGTAGAAACTGCCACTCCATCCCGATGACTCCGCCAGCTAGTGGGCCACAGAATGTCATTTTAAATTTTAAGGACACAATGGCGCTGCGCATAAAAAGCAACCGCAGCGACTCGAGAGATTTATTTCAAAGGCGGCCCACGGAGCCACCGCCACCTCCGCCTCCCAAAGGAGCAGGCAAAGGATTAGAGCCGCTGCCACATGAAATCAATAAGTCGCC

>ID_1829 P_1829 Promoter_1857 D1_Promoter for RH42274, -250bp and +50bp []

TGCACATAAAATGAGAAACATGAAATTGATTGTAAGCAGCGCCTAATACGGACAAGGTGAGCCATAGAAGCATATGATGATCTTGATCTTGAGACGACTGCTCCACTGACTCTCCTACATAACTGCCTAACTGGCTAACTGCCTTACTGACCAGTTTTCCCCGCAGCTGATTGCATTGAGTGGCGCTCACTTTCGGTTCGGTCGCCTGGTGCAAATCCACTATAAAAGCCGCACTCGGAGCTCAAGTTGTCACCAGTTGACAATTTGAAATCATTAGCCTAGCTACCTTTCCTACTTCAGTTATCCCGCCAAAATCCAACGCCTCAAAATGTTCAAAGTAGTGAGTATCA

>ID_1830 P_1830 Promoter_1858 D1_Promoter for RH37294, -250bp and +50bp []

GCGATGGCTTGAAATCCGATATAGTTTCTGAGGAACGCCTCGGGCGGAGTGGGCGAAATGGCGGACCCATTCCTCATTAGGAAGCTACTGGGAATGCCTTTGTTTGCATTCTCCGAGCTTCCCCTTGACCGTCCTTCCGCCGACGTAGTGCCACGCTGAAATGTGCAACATATCGGGGCAGGAAACTCACCACACGGAACGGAAAACGGAGAACGGAGAGCTATTAAAGCCGACCCCCAACGAAGCGTGAGTCAGTTCCCGACTGGGCGTCCGGGCATTCAGACCGCTCGCAAGGATAACGCGCAAGGATCCGAGCGAACGCGACGAGTAAATAAAGCATAAATAATACA

>ID_1842 P_1842 Promoter_1870 D1_Promoter for RH26557, -250bp and +50bp []

CTATTACCTGATAAGAATGGCCGCCGCATCAAGGACGGCTTGGCTACCTAGCGCTTATACGCATTTGGGACAAGTTTGTACAATTGTTTACACATTTTTCTATTTAATAAAAATTTTATATTTTCCTAGAAATACGTGTCATCTTCAATGTTTGTTGGGTTGCGCTTGTCTTAATGGCTTTTTAAGCTTATTTTGATGGGATATTGTGTAGACATGGCCATATAAATGCATTTATTTCTGGACGAGTTCTATTCAGCATGGTTACCATACTGTTCACTATCTTTAGCTTACCTTTCTTGGTAATAGCATCCAGCAACATCAACAATACCGATTTGACTTTTTATCAAAAG

>ID_1845 P_1845 Promoter_1873 D1_Promoter for RH61745, -250bp and +50bp [3 ]

TGATGGCTACCCAGTTGTGGCTTCTGCCTCTGCTTGTAAAGCAGACCCAGTTGACCCTGGTCAAGAATCGGACGCTCAAAGTGGTTTAATAAACTGCAATTACCCGACCTGGCCGGGCCGGGCCTGGCCTGGCCCAGCCACCCGCCACTGGAGATTTATTTTGTGGATTCTCGTCCAGATTAGTCTGGGCCACGCAGTTTGCAGTTCGCCAGTTATTTTTGCGATAAATAGCGAGTGGTGATCCAGTGGTTACAGCATTCGAGTGCCACACTTCGAGACGCACAGCAAAACAAACCAGGATCTACTCTCCGAGTCAGGACTAAAAGCTTAAAATCCCAAACCACACATAA

>ID_1849 P_1849 Promoter_1878 D1_Promoter for RH09818, -250bp and +50bp []

CAATCTCGTTATCATTTGTTTATATTTTGATTCGCGAGCATTTTTGCAGTCAAGCGTATGAGTATCTCTTTGAAAGTGCTCTTAAATCGGTTTGAAAAGTGCCTGCATATTATCGCCGAGTCTGTAATCATGACAACAACTTAAAAGTAGTAGTCATGGTGATAGAAATATTTAGCTAGCTAGCTCACATGCTGTCATGCCTGTGCGTCGCAGGGGAATCTTATAAAAAGTGTGCGAACATATTGTGGTGATCAGTAATTCGTCGTAGGTCGAGCACGACGATTGCGAAAAGGGAGCAGCTACGCAAAATGTTTGTTCTAATATACCTGTTGATCGCGATCTCCTCGCTT

>ID_1851 P_1851 Promoter_1881 D1_Promoter for RH17496, -250bp and +50bp []

TTTAAACAGTTGGCTTACAAATAGCATAGTTTTAGTTCTAGTTACTTCTCTAGTTCATCATCAGTGTATTTCATTTCATTCATTTTCATTTTAAAGTTCATTTTACCACTTGAACAGCATTTCATAGTTTTTGCGAAGTTCGAAGTGAAAAAGAAGAAAACTGTTTTTGAACTATTGTGGCATCGCATCCAAGGCGAGGGGCCACACTGTTTCCAATTAGAATAAAAACACTCACCGCTCGCCTGGTCACACTGGCACTGGCGAACAGCTGTTTTCCCAATCGAAAAGCGCTTGTGTTCCAAATTTTACGTAATTCGTATTTTGAAGCCGATTTCTGCCGTGTAGCCCCA

>ID_1854 P_1854 Promoter_1885 D1_Promoter for RH13728, -250bp and +50bp []

GACATGCGACTGCAGTTTGCTCCGGGCCGGATTTCTCGATTTCTCGCCGAGATGTGGCGAAGGCGACGAATCGAAATGGAAATGGAAACTAATTAAGTGACAAGTGCACCTTATCTGATCGGAACACACATCGATATGGTCACAGTTGACTGGAATCATATATATATATATATATACATATAAGGCAATGTGTTTGTCGCGCGAGCCCCTCCCTCTGGAAGCTATAAAAACAATTGTGATTTGGCTTCCGGCATTCAGTTCTCGTTGAGTTTGTGTCCAGGATGGGACGAGTTCCGGCAAAGATGCACACGCTCCTCGCGCTCCTCCTCCAGCTTCTGGTGGCCTCCATC

>ID_1858 P_1858 Promoter_1890 D1_Promoter for RH10371, -250bp and +50bp []

AGACGCAATGATATGAAATATTTATCATAATACGATCACTTTTTTTCTCTGTGTATCACATTTCCGAGTACCCGAACAAGTAGTTCGCTTGTCTCCGCTGAGCCGTCCACCGACGCTAATTGCTTATTGATCGTTGGCCAGAATCTGATATGGGTATGACTTCGGTCCGAATTGTGACGCACTTTTGTGAGCCGGAAGGTCAAAGCCAAATGGCTGCTGTATAAAAAGATAGATGCAGCCCGGCAAACGGCAACAGTTCTCGCAAGAAACTCAAACCGATCACTCCCAATTCCAAACAGTCAATACAGACCTAACCCATTTCCAAAATGTTCAAGTTGGTGGTATTGTCT

>ID_1863 P_1863 Promoter_1895 D1_Promoter for RH14320, -250bp and +50bp []

CAGCATCGGTATCACCATCGTTGTTACATTAACATTATTATGTGCTCTGGCGTGTGCATACATGTGCAGTCTAGTTAGTTGGCTCGTTACATATTTATTTCTTGTGTGGGAAACTAGTTTTCTCCCAGTCAACCAGCCAGCCAGGGTCCGTGCAGGTTTTTTGTTGGCCATTGATGGCGGAGACTTCCGAGCTTCCGTTTGGAGCATGTAGCGCCAGTATAAATGAATTGGCCAGCTCCAAGGAGGCCTCACATCGTTTCAGATCTAAGAAAGTGAAGGTGGTTCAATTGGAGCACTGCATATGAGGAGCTGAAAGTGAAACGTTCAGAGCTATGGGAATTAAATTGGAC

>ID_1883 P_1883 Promoter_1916 D1_Promoter for RH66281, -250bp and +50bp []

TCCAGCGCCCCACATTGGTTTTGGCCCAGTGGGTGACAATTTCGTACTGGCATGGCTTAAAAGTGAATGCTGCAGTGCAGCTCCAATTAATCTGCATCTGTATCTGTGTATCAGTGCCTCGGGAAGGTCATTTGTAACCTTAACTAAACAAATTTGAAAGCTGGTGCCCGGCATATCAGGTGTGGTCTGGTTCTGGCGATGGCCCTTTTATGCCCTGTCGTTATAAAATGGCCAGTGGGCGTTGGTGGTGGCATCAGTTCGCCATCGGCAGCAAACGGAAAACAGCAGCAGTAGCCAAACAAATAAGCAGCAGCCAACTAGTCGTATACTTAACGTCAGCAGCCATGAAA

>ID_1888 P_1888 Promoter_1921 D1_Promoter for RH45234, -250bp and +50bp []

GCGGGAATCGCTGCCTGGTTCTGCGGGCAGAACTAATTCTCTTAGTGACCAACTGATAGATGGCGATCTTTAATTTGCGAACGCAGTCCGGGAAATTCCGGCAGACGCATCCAACACCTGATGCCAATCGGGTTTTTCGAGAGAGATAGAGAGCTGATTGCTCTGGGGCTGTCTGTAATTGTTGGCTTACCGATTTCCATAATGGTGGGCGATTCTGGAGCTATATAAGTCAGCTATGGCCGGCAGAGAACAATCAGTTTTCGCTTGGCCCTTGCTAGTTGAACTCAGTCGGTTGGGATGTTCATCAAGTTGCTTCTAATTAGCCAGGTTGGTGCCAGATCTTGGCCAGG

>ID_1889 P_1889 Promoter_1922 D1_Promoter for RH49329, -250bp and +50bp [8 ]

ATGAGTGGGTAAAACCCAAAACAGGTTGTTCGAGCTGCGTAGAAATGATGTTTTTATTTTCTTTATTTTTTCCACCCAGTTGTTTACAATTTCAGCTGGGGGCTCTTGAGAGTCGGGGGTTTTCTTCGCCATTTGATTATGCTCAGGAAATTTAATTGCTGGAGGTCGTCGGCACAGAGAACTCGTTTAGAACCCCACAGTCATCGCGGTCAGGTCGAAAGCTATATAAAGAGCCTAGGCGGGGGATCAGCTCGACTATAGTAAGAACTTTAGGTGAACAAGACAACGGAGACGGAACGATGCGTATTTACCACCTGAATCTATTAGTGCTAAGTGCCGGCCTACTGGTC

>ID_1892 P_1892 Promoter_1925 D1_Promoter for RH24258, -250bp and +50bp []

GTCTATTGACTTTTGCATTTGCTTTGTTTTCTACAGCTGCGACGGCGATTAGTTTCAAGCTATTTTTGTTATCCACCCACCACCCACCGCCCACCGCTCCGCCCACAAATCCCTATTATTTGCTAATGCCGAGCATCTGTGTTTGTGCCTTTTTTTATTTTTTTTATTTTACGTTTGTCTTTGCAATTTCGATTTGATTGGATTTGTTTTGTTTTCTTCCCATAAATAAGAGCCCGCCACCCATCGCAGCTCTCAGACGTTGATTCGCATTTGCATCGGTCAGGTTGGGCTATCCAACGGATGTGGAATAACTTCACCTAACGAGGAAGTCAATTAAAAAAAACATCATT

>ID_1893 P_1893 Promoter_1926 D1_Promoter for RE28280, -250bp and +50bp []

ATTATTTTATTAATTTAGCATTATTTTATTAATTGTACATTTAAACTCAAAATATCATTCGTCATCGATTTTCTCAAGAGCAATATGTATGTTGCCCAGTGTAATTAGATATGAGATGGAACACAATCAAAAGAAAACCGGTAGGTGATCGGCCCATGAGCCGAAATTATAAACATTTGACTCTCCAGGTATGTGACGACTTTTCGAAAAAATGCAGCAGGTATAAATACTTTAGCGACTCAGCTGAGAGTCCGTCAGTTCCAGCCCAGACAACCCAGCGATCGCACAGATAGAATCTATCAAACTCACAGAAGAATTCTCGCAATGAAGACTCTGATTGTTCTCGCTCT

>ID_1894 P_1894 Promoter_1927 D1_Promoter for RH03850, -250bp and +50bp []

GTTATTCCTTTTAACTAGGACAGCTTACGCTAATTAAAAATATACTTTAAAGCCACCTGAAGCTTAATTTTTCCCAAATTAAATCTGAAATTATATATTGATATGTAAGAACCTGACAACAAGAAATAACCCGCAAGTCAGCATGGCTAGGTTGTTTCTCAGTGCAGCCAAGTAGATTGAAAGGGAATCACTAAACAAAGTCCAAAGTCCATCATCGCAAATTGTATAAATGCTGACTCGACGAGACATTTAGCCAACAGTTTCTATTCTAAAAGATTCTACACAGCAGATTACGATCAGCATCATGAAAGTATTCTTTGTGTTTGCCGCCCTTGCAGCTCTATCTTTGG

>ID_1902 P_1902 Promoter_1935 D1_Promoter for RH68528, -250bp and +50bp []

GGACTAACCGGTTGCAGCGGGAGATCGAGCAGACCGGGTTTATTGTGTGTAGGGTCCGACGGTCGTACGCTCCGAACTTGCTAATGAGTTGAGCCATAAATATTGATAATTTGTTCTGTTTTTTCTCTTTTTCTCATTTCTATCTACTTTTGCCGCTTAGGAGAGTGAGCTCCAGGGTAGATCACCGAGCAGTCAACGAATCTATTCGCCAACGGGCAACGGAATAAAAGCCGGGTCCGGCGGTGCATTGGACAAGTGTTTGGATCACCTTCGAGGAAGCAACAACTCAACATGAAACTTTTCATCGCGCTCTCTGTTCTGGTGGCTGTGGCATCGGCCCTGCCTCAATT

>ID_1905 P_1905 Promoter_1938 D1_Promoter for RE47575, -250bp and +50bp [-1 ]

CCTTGTTTCTCATGTGGTTATTAATATCAGCCGAGTATTTTTGCTAGTAACAACTGAGTTAGTGAAACCAATTTGAACAACTTGCCTAAACTTGTTGATTTGCATTTTGTGTTAGGTTTTAAGCCATTCAGAGATAGCTGGTACTCAATAATATACATTTTTCACATTATGTTTTGACTAATTCGGATGCGTTCAGCATAATCGAGAGGTTGTCCAACTATTTAAGACGCGCCCTGGCCAACAGAGAGCATCATTCGTCGCGAACTGTTCGATAGCCGCAGATCTAAGCACCAAACAACAATCACCATGAAATTCCTGATTGTCTTCGTCGCCCTCTTCGCCGTGGCTCT

>ID_1906 P_1906 Promoter_1939 D1_Promoter for RE08209, -250bp and +50bp [-1 ]

CAGTTAAATAGAAACTACGAGAAGACAGCTGAAAAAGGAATGCGAAACTATGCAAACTATATAGATGAAGATGTCCGATTTACATATCGTATAGGGCTTTTAACGATTCAGCTTTAGGCCGAATTCAATTTCACACATTTATCACATTTTGCTTTATACACTACAAGTTTGACTAGTTTGGCAGCGTTGGGCTCGGTCGTCAAAGTTTGTCCATCCATCTATATAAAGGGAGTACAACCATCAGAGGGCATCATTCGTCGCGAACTGTTCGAGAACCGCAGATCAAAACACCAAACAACAATCACCATGAAATTCCTGATTGTCTTCGTCGCCCTCTTCGCCGTGGCTCT

>ID_1908 P_1908 Promoter_1941 D1_Promoter for RE50728, -250bp and +50bp []

AATTGTTAAAAATTTGAATAATGAAATATTTATGAACGCGATTCTTTTTGGACTATAGTGTTATTAAAATTTTATATTAGGCCTTTTAATGCTCCTACTTCAAACGAAAATGAAATCCAAACCAGACATTTCACATTAACATTGATTTTGCTCATTATGTCTTGTACACGTTTGACTAGTTCGGCCGCGCTTTGAATAATCGCCAAGAGGGTGCCATCTATAAAAGGGTTTACGCGGCCGTCCATAGTTACCATTCGACTTGAACTGTTCGATAGCCGCAGATCTAAGCACCAAACAACAATCACCATGAAATTCCTGATTGTCTTCGTCGCCCTCTTCGCCGTGGCTCT

>ID_1911 P_1912 Promoter_1945 D2_74F EMBO 3:289 8 [-2 ]

ATATACACTGAATGAATGGAAACTGAGAAGAAGACGGTCAACTTTTAGTTGAAACAGTTGAATATATTGTACGACGAAGCTTCTTGAAACTGACGAGGGAGAACACAAACAAGTTAATTGAATCGAGAATCGTATTACCTACCCGATTCCCCCCTCACAGAACGCATACCATGCCAGCCAGCGAACCAACCATTCAACTTATGTAATCATATAAATTCTATAATAAACAAAGAAACAAAACTAGTTGTAAAACAAACACGATTCCTGTGTGTCATTGCGGGATATGGAAATTGGCTTTCAATCTGTTGCAAAATGAGATTCAGTAGCAGTGTATATATCTATGAGGGCTG

>ID_1917 P_1919 Promoter_1952 D2_Act79B JMB 163:533 0 []

TTTAATTCGTAATCCAACTTGTTGCCGTAGGCGTAATACCCGAAATCAGAACACTTTTGTGAAATCGAAATGATGTGCATCCGACCACCCTCCCCGGAAACGCCTGATCCCCAGCCAGCGTTGCATATCGCGGAATTCATCAACATGTTACTAGATGAACAATTGTTCGAGATGACAGGGACATGGGCGTGGGGCCGGCGGGGCGGGACAGAACTTATTTAAATGCAGCTGCCGGAGCGCATAACGAATCACTCTGATCGCTGTCGCTGTTGGATTTACACGTCGTGAGTGTAGTCTTGTCCGCCCATCCGAAATCCGTAACCCGCATAAGGGATAACCGATCTGTCTGT

>ID_1918 P_1920 Promoter_1953 D2_Act87E Genetics 119:407 -3 [3 ]

CATATTTTGAGCCATCTTTCCTGCAGTGCACCATCTGGGAAATTATGAACGAAGCGAGCAGAAGTCCAAAAGCAAAAATCCAACGAAAACAAATTATTTTTAAAAGAAACTCAGAATCTCCCCCCGCCGGCGCAATGTGCATCCATGTGCACATGTGTGCCGAGAGGCGATTGAGTGTGCGTGCGGAAAATATCTAAAACGACTGAGGGTCGCCAGAATGGTATAAATATTAGCGCATCTCGGTCCAGCGACCACTCGCAGTTCTACAGCGAAAGTGTTGATTTGGATTTCTAGTTTTTCTTCGTCTAACGGTTAGTATACTCCACATCCACCAATTCCGTCTGTCTGGT

>ID_1919 P_1921 Promoter_1954 D2_Act88F Gene 118(2):163 0 []

TTCTATGCTTTATTAAGTATTTAATTGTGTGGCTTCCTTTTTTTGTGAAGCGCATTAATGAGTCGTCTTCGTGCAATGAGGCATCCAAACTTCTGACATGCTCGGCCAGAAGTCTGAAAACTGCTTATATGGATCGATTGTTCCGCAGCACTTTCGCTCAATCTTTTTCTCAGTGCCGCACTGGCATCCAACTCAAATCGCTTCGAGGGAGAGCCGAGATATAAAGGCAGGACAGACCGATCGGCGTGCCATTTGTTGTTGAATCTAGTTGTCAACAGGAATCGAACGTGCGACTCTATCCAATTTTTCTCCTTTCGTTGACCTAAAAGGTGTGTGAGTGCGACCTCAAT

>ID_1920 P_1922 Promoter_1955 D2_ald JBC 267:3959 0 []

GCTCCCAGAATGAGCTTTATTTTGGATACCTTTCGTTTGGGGATCTGGGAATTTAAAGCGCTTGGCTCAGTCGGTTGCCCAACACCGTCGGCGACGACCTCCAAACAACCTTTTGGGGTCTCGCTCGGCATGTCTCGCTTATTTCGCTTTGGAATTTCACGTTGACGCCGCAGTCAGCGGTAGCAGAGGCATCGGCAGCGGCAGTAGCAGCGACACGGGCCGAAAATAAAAGCGTTAACCGCTCTCCTCCAGTGCAGCAGCAGCAGCGGACCCAGCCAGTAGCCAGCAGCTAATCACCACATCCCAGATTCAGTTTCCAGTTCGGTGAGTGGGCGTCATACGCAGTGAAC

>ID_1921 P_1923 Promoter_1956 D2_Adh1 (adult)* Cell 33:125 0 [0 ]

TGGAATATTTTCATTCATTTTATGTTTTTACGTTTTCACTTATTTGTTTCTCAGTGCACTTTCTGGTGTTCCATTTTCTATTGGGCACTTTACCCCGCATTTGTTTGCAGATCACTTGCTTGCGCATTTTTATTGCATTTTACATATTACACATTATTTGAACGCCGCTGCTGCTGCATCCGTCGACGTCGACTGCACTCGCCCCCACGAGAGAACAGTATTTAAGGAGCTGCGAAGGTCCAAGTCATGCATTATTGTCTCAGTGCAGTTGTCAGTTGCAGTTCAGCAGACGGGCTAACGAGTACTTGCATCTCTTCAAATTTACTTAATTGATCAAGTAAGTAGCAAAA

>ID_1922 P_1924 Promoter_1957 D2_Adh2 (larval) Cell 33:125 0 [0 ]

AATACTAATACTAATATAAGAATACTAATATAGAAAAAGCTTTGCCGGTACAAAATCCCAAACAAAAACAAACCGTGTGTGCCGAAAAATAAAAATAAACCATAAACTAGGCAGCGCTGCCGTCGCCGGCTGAGCAGCCTGCGTACATAGCCGAGATCGCGTAACGGTAGATAATGAAAAGCTCTACGTAACCGAAGCTTCTGCTGTACGGATCTTCCTATAAATACGGGGCCGACACGAACTGGAAACCAACAACTAACGGAGCCCTCTTCCAATTGAAACAGATCGAAAGAGCCTGCTAAAGCAAAAAAGAAGTCACCATGTCGTTTACTTTGACCAACAAGAACGTG

>ID_1923 P_1925 Promoter_1958 D2_andropin EMBO 10:163 -1 []

AACCTACAGAATTGTAGAACTTAATTACTATAGAACACTATTGAATGAAAACTTAGTAACTTGTTGAGGTTTTTAGTAATTCCAAGAAATATGCTCTTGAATAAAAAACCTTTTTAAGTCTCTTTCAATGCAAAAACACGAGTTCTTTTTTTTTACATATTGTAATTAATATGTTTAAGGTCTAATTATTATTGTAAACGTTTTTCGGTGGGTTGATTGCCTATAAAGCCACTTGTTTTTCAGTCTAAATCATCAGTGTAAAATTCGGAAAACCCAGCGATCTAGTTATGAAATACTTTGTGGTCCTTGTCGTCCTGGCCCTCATTTTGGCCATCAGCGTGGGTCCTTCG

>ID_1929 P_1932 Promoter_1965 D2_ASC T5 Cell 50:415 0 [-7 ]

AAAAACACTCAGAAACTCTTCCCACTCGACAACGGGAACACTCAGGTCACCAACAGCTGCGTTTTACAGAGAGAACGAGAGATAATATTACTACCTCTCTATTAAAATCAGAGAAAACACTCATCTCAAGAGACGATCCTTCAGTGATGATGCTGTTGCACCTTTTCCAGGGGCAGGTAGGTAGTCACGCAGGTGGGATCCCTAGGCCCTGATACCTATAAATAGCCTGAACGGAACGGGGAAGGGCATCAGAACAGAGCCAGCGCTGAAGCAAGGAGCATCGTCACACAATAACGTTATACTATCTCTTAAAATGGCTTTGGGCAGCGAAAATCACTCTGTTTTCAACG

>ID_1930 P_1933 Promoter_1966 D2_ase EMBO 8:3553 0 []

AAATAAAGATTCGATGCATTTCTGCGTTTTACATGACGAATATTGGAGGTCTAAGGTGATCTATTAGGATATTTTGCAAATTCCTAGGTGGTAGGGCATTCCTTGAAAACCAGGGCTGAAAAAGCTCCCCAGGGAATATACTTTTTATTATATGCATACGTATATGGTTATTATAATGTCCATATATTAATAGGGGCGGTATATAAGCATAATGTGTTTGCTGCCGATAAATAATGAGAGAGAGCGTGGCGAAATCCACCCTTGAACCAGGTGGACTTTTTGGCTCGAGTTGATCAGATGTTAGTTTTCCCAAAAGCCGTACTGTATATAATATATATATATGATAAAGG

>ID_1932 P_1935 Promoter_1968 D2_b1 Tubulin M&GG 211:8 0 []

GATATCTTTTCAACTGAGTTTAATGTGATAAAATGGACAAAAATGTATTCCCTTTTAATACATTTTTCAACATAGCTACTATCTTTCGATTAAACTTTGTATTTATTTCATTGACATTAATTAAAACTTAATGTTATGTCAGGCAAGTGGGATGTTTTAGCACACTAAAACGGGATTTGCGTTTTATTCGTTTTCATGTAGTATGGCCACACTGCGGCCATCGTATAAAAGCCCGCGCTCTCCAAAGCGAATGCACTAATTTTTCCAAGTGTGTGAAGCGGACAGTTTGTGTTGTGTTCGACTGCTATAAGCGAAAGATAATTTTAAGTTAAAAGTTCAAAAGCCTAAAA

>ID_1936 P_1940 Promoter_1973 D2_CecA1 EMBO 9:217 -4 [0 ]

GAGTTATTAAAGCTCTAATTCAAATTGGCCCAGAACCGTTTAAAGGATATTACAATTTGTAATTTACATATTTGGATTATAGCATTGAAATCCCCGATTGTTCCCTAGATGTGCAGATGTGTGCTTGGAATCAGATCGGTTACCTTCAGTGTACTTTTCTCTGCAAAAATCCCCGTGCATGCCTTATCTGTCATTTTGTTTTTCAAGCTGGCTGTTCGCCTATAAAAGCTCTCGCCTTTTGTATCGCAGTCATCAGTCGCTCAGACCTCACTGCAATATCAATATCTTTAGCTTCTCCTAAGAAAAAATCAAGAAAATATCACCATGAACTTCTACAACATCTTCGTTTT

>ID_1937 P_1941 Promoter_1974 D2_CecA2 EMBO 9:217 -4 [1 ]

TGTTTTTATTTTGATTGATTCAAAGAAGGGGTTTCCTCTCTGATTCTTAGTCTCCCGCATTGACGAGGTAAAAAATCCCTATGCATATGAAATATGCAAATTTAAAAATCCCCCAATCCGACAGGTTGGTTTTGATCGGTTTGGATTCCTCTCGTGTACTTTTCAGCCATAAAAATCCCCTTTCGAGCCTTATCAGGCGCTGAACTTAAGCTGATTCGCCTATAAAAGCTCTCGGCGTTCCTGGTGCAATCAACAGTCGATCACTTTCCATTGCAACAGCAACATCAGAGCTATAGCTACTCTTGCAAAATCTAAAGTCAAATAAAACCACCATGAACTTCTACAACATC

>ID_1944 P_1948 Promoter_1981 D2_cytb5 NAR 17:6349 -1 [2 ]

CATGTGGTGCTTAGACATGCCCACTGCTTGAGATTTCACCAAATGAATTAGCGATTAGAACATACTGCTAAAAATATGTGAGAATTAAATAGACGTAATAGTTAAAAGGCGGCAGCTGCGCTGCTCTCCTGATAACACCGGCGCCTTATCAAAATGGAAACGTTTTGTTTGGAAATCTCGATTTTCGGTTTATATAATCAAAAGAGATTTGCGCTACACATAAAATAAACGGACCACTGCCGGTGCCTTTCAGTTAGTCGTTGGAATCCCAGCGGAAAGGTGCTCGGATCGGTAAAGTTTGTGCCACAGACGTCAAGGATCAAAAGGACTCGGCAATATGGTAATCGAGG

>ID_1945 P_1949 Promoter_1982 D2_Ddc MCB 6:4548 0 [-1 ]

TTAAATTCTAAATAAATCCATGGAAAATAAAGCCTTTGATATCCAGTTACTGATTCAGCGCCCAATTAATGCATGTTCCAAAAAAGTGTCAAAAAACGTGCACAAATCAAACGAGAGCTGAATTTGTTTTTACGACAGCGGCTGCGATTCGAAGTTCAGCGGCTGCGGACTGCGATTGAACCGGTCCTGCGGAATTGGCAGCGCTGCTGGACGGGCTTTAAAAGCCATGGCCAAGAGCGGGCAGCGCTCAGTTAAGAGGAGAACGCCAAGCGCACAGCAATCAGCACCGAAATATCAGCATCGAAATATCAGCAAATAAATATTAGCTGTTCTAAACCAGGAGGGCAAAC

>ID_1951 P_1956 Promoter_1989 D2_drosocin EJB 241:701 0 []

TTGGTAAAATAACCAGCCGCCTAAAATGTAAAGTACCGAATGATGTAAACTACGCAGCGAAAACCCACATTAAAAAATAAAAATAATAATTTTCAACACGTTTGCCTTCAGTCGCCTATTCGTTGATAGGCAACATGAAAAGTCCCCAAGATGGGGAATCACCGGCAACCACAGCCAAGACGTGCCACCCATTCGAATCTCTTGTTGCATCGATGGCATCTATATAAAGCCAAGTGCTCTGATTGAAATCAAATCAGTTCGATTTGTCCACCACTCCAAGCACAATGAAGTTCACCATCGTTTTCCTGCTGCTTGCTTGCGTTTTTGCCATGGGTGTGGCCACTCCCGGC

>ID_1961 P_1968 Promoter_2001 D2_E(spl)m7 EMBO 8:203 -3 []

CATTTCTCATACTATTTTTACCGCAGGTGTGTTCCAAACTTGTTTGTGGAGAAAAACCCACCACCGAAAATGCAGGCGAGAGGATCGTGTCCCAGTGACTGAGTAAAAGTAGTTTGCTCCGCAGGTGGTGGTTCTTCGGAGGCTCCGCAGCTCTCTCTTCTCTCTTTCTCCGAGCAGCGTGCGCTGAGCACGAGACGCTCTCGCGGCGGCACGTGCAGCTATAAAAGCAGCGGTAACCGGAGACGAATGCAACATTTCGAACGCAATCGTCGTGCAGTCAACATCTCAGGATAACCTCCTCCGAAAGATCGAAGAAGTTCTTCAACAAGTCGTTCTTCCATTTACCTAAA

>ID_1962 P_1969 Promoter_2002 D2_E(spl)m8 EMBO 8:203 0 []

GCATGTGGCCAAAAGGGAAAACAAACGAGAAAAAATTGTGTGAGAAACTTACTTTCAGCTCGGTTCCCACGCCACGAGCCACAAGGATTGTCCTCCGTCCTACGAAGTTGCAGCTGTTCCTTGTGAAAAAGACAACAACACCGGCGTTTAGCCGTGCGAGCGAAAGAGAGGGCAGGAAAGGTGCGTATCAGAGGGTGGATTTCAGGGACCAGGGAGCGGTATAAAAGGACGGGACCTCGCAGAGCAGAATATCACTCACGATTACGCCGTCGAGCAGGCAGCAACAAGCCGAATACAAAATTTTTCAAAAATTTTTAAAACTACGTAACCTACTACCAAAACAAAAATGG

>ID_1963 P_1970 Promoter_2004 D2_fbp1 JMB 214:359 -2 [0 ]

TACGTACCCTATTTGGTCAGAATCCCGAAAGGCCCCACCGCTTTCGGGAACAATCCATGACAACAATTTATTTAATTTTAATTGCATAATGAGTGAGCGGTTTTTTTAGGAGCTTCTGGGCTGATAAACATATTTTCGCACCAGCAAAATTCATTCAACCCAATCGGGAGTCAAGCGATAGGCCTATAGGATGCACTTTGGGTCGATAGGAATTTCGGGTATAAAAGCTGCAGCGAGCGGACTGAAAAATGCAGTTGCCTGCTGGTCTGTGTTGCAGCAAGTTAAATCCATCTGAGCTCTCCAATCCTTCAGTTTCTCAATTGATCAGTTAACATCAATAGTTGTTACAA

>ID_1964 P_1972 Promoter_2006 D2_ftz Nat 310:25 0 [0 ]

GCAGCACCGTCTCAAGGTCGCCGAGTAGGAGAAGCGCGCGGGCGGATAAATCGCGATGATAATGGGCGCGATGGGTAGGTAATAAGCCGCGCAGCAGGTAGGCACCGTACGGATAAAGTTGCCAGGACCTCGGATAACTTCCCCTCTCCGTGCCTGCAAGGACATTTCGCCGGAGGGGTGGCTGCGAACAGCAGGCGGCAAAGTGTCATGCGCAGGGATATTTATGCGCTATAACGGCGAGCGTGTGCCGAGGGCTCTCTGATTTTGCTATATATGCAGGATCTGCCGCAGGACCAGCTCATTCGCAAACTCACCAGCGTTGCGTGCACATCGCAGAGTTAGAGAAGAAA

>ID_1965 P_1973 Promoter_2007 D2_G42 G&D 3:96 0 []

AAATTTATCTGCTCAAAGGCAGGTTAACGCACTTTTGACGCACTTAGATATTTAAATTCGACAAAAATCCCCGACTATGAAAACTGTGTATATGAGCGCAGAACAGATAACCGATAACTCCTCTATCGCAGGTGGCTGCCAACTTGCTTCAAAATAGAAAGCGCACTTTTTAAATTTCCAAAAGTATTCAAACACTGGTCTTTAATTTCTTAATACATATTTAAGTTGGGCAAGCAATATTGGTTTATGCAGTCGTAGAAGTGGGTTCATCGGTTATGGTCACAGCAGCTGGCGGCGGATCGCCGGCCTTCCGTTGATCCCTAAAGAACATGACCCACGCGCTCTTATGG

>ID_1973 P_1986 Promoter_2021 D2_hb (zyg) Nat 327:383 -1 [-6 ]

CTCGCTGCTAAGCTGGCCATCCGCTAAGCTCCCGGATCATCCAAATCCAAGTGCGCATAATTTTTTGTTTCTGCTCTAATCCAGAATGGATCAAGAGCGCAATCCTCAATCCGCGATCCGTGATCCTCGATTCCCGACCGATCCGCGACCTGTACCTGACTTCCCGTCACCTCTGCCCATCTAATCCCTTGACGCGTGCATCCGTCTACCTGAGCGATATATAAACTAATGCCTGTTGCAATTGTTCAGTCAGTCACGAGTTTGTTACCACTGCGACAACACAACAGAAGCAGCACCAATAATATACTTGCAAATCCTTACGAAAATCCCGACAAATTTGGAATATACTT

>ID_1977 P_1990 Promoter_2026 D2_hsc4 MCB 10:3232 0 [3 ]

AGGAATAGGAAATGTGGTTTTTAAAGTAACAAGCTACGTTCAGCGCTTCTACTACATATTAGCCAATTTCTACGACTTTACAGTTTCGGTGTGAACGTTCTGCGTCAACATTTCTGGACTTCTGTGTGAAGCGCTTATTAGGGTGGCCAAGAATTTTGCGGTTACACCCCTGGGAATTTAGTACTAAAATTGTTGATGGCTTTGGTAACACTTTTGCTTGTATATAAAAGGCATTCGCAAATTTTGTACGGGTGTAATTCAGAAAAAAACGCCAGCCAGTTTGATCGAAGGTGCGGCAGATTAAAAGTGAAGTAGCAATTAAACGGTTATATTTTAGTACTTTCTAAGAA

>ID_1979 P_1992 Promoter_2028 D2_hsp22 NAR 9:1627 -1 [0 ]

CGCTAAATGCTAAAATAAAAGGTAAATAAAGTAATATTTGGACACCCAGAGAGCCCCAGAAACTTCCACGGAGTTCGCTAAAGAACAGTGAACAACCCCTAACTAAATGCCATTGCCCGATTTCAGGCAAAGTGGAAAATTGCATCAGCAAAGGGCGAAGAAAATTCGAGAGAGTGCCGGTATTTTCTAGATTATATGGATTTCCTCTCTGCCAAGAGTATAAATAGCCACCGGTTGGACACTGCGCTCTCAGTTCAAAAAAACCAAACCAACTGCTAACAACTCGAAGAAAGTCAACTAAATTAAAATTTCGCCAGCTAAATAGAAATTTCATAGGATTGAAACTTCAG

>ID_1980 P_1993 Promoter_2029 D2_hsp23 NAR 9:1627 -3 [-1 ]

CCACTCCCCTAGGAGATTGCTCATTTTCCATAGCGATACTCTCACTTTCAATGGCAGATAATGCGTAATTGCGGCAAATTCGAGAACTCTGCGATATTTTCAGCCCGAGAAGTTTCGTGTCCCTTCTCGATGTCGATGTTTGTGCCCCCTAGCACACAGACACGACGCGCACACACACAGCGCCGACGGGCGCACGCACACTACGATAGCGAGCGGTTGTATAAATAACCGGCACTTTCGTGCAACCGGCGTCAGTTGAATTCAAAAAGCCAAAGCGATAACAGCTAAAGCGAAAGTAACCTATCAACAAAAGAAGTTTATTCTTTGAAGGAGAATCATCTTGAAGCAAT

>ID_1981 P_1994 Promoter_2030 D2_hsp26 NAR 9:1627 0 [-1 ]

TGGGAAAGGTTAGTTAGTTTTATTTTTTGTTTTTAGAGCAGCATTCAATTTAGACTTTTATAAAAGAAATTTCTAATTTGATCCCTCGTTTATCAAACGATACAAAGCTATATTCATAATTTTTTCTCTCTGTGCACGTTCTCTCTCTTCTCTTCTCTCTCTCTACTCTTTCCTTTTTCTGTCACTTTCCGGACTCTTCTAGAAAAGCTCCAGCGGGTATAAAAGCAGCGTCGCTTGACGAACAGAGCACAGATCGAATTCAAAAATCGAGCAGTGAACAACTCAAAGCAACTTTGCGCAAAAGCAAAACTTCAAACGAGAAAAAAAAGGATTAAAAACCTTTGCTTACA

>ID_1982 P_1995 Promoter_2031 D2_hsp27 NAR 9:1627 0 [0 ]

GGGGCGTATTCAAAGGGGCTTTTAAATGTCGCTTAAATTTTAAGTTTGACAGGCTAATAATTGCTTGCCTATATCTAAATATTATTATATTTGCATTAGGGGATCATAGGGAAAACCTTCTCTGCAGGCAAAATCTAACGAAGATGGCAACCCCCCATCATTTTATTAAAGTTCCGTCCCTGGTTGCCATGCACTAGTGTGTGTGAGCCCAGCGTCAGTATAAAAGCCGGCGTCAACGTCGCCCGAGCACAGTCTAAACTGAAAAATTGAAGGCAAACGTTGAAGCAAACTTCGCTAAAAAAATTCGAAAAAGCAAAAAAAATTCCTTTGTCTAGACAGGGTTGTGAATA

>ID_1983 P_1996 Promoter_2032 D2_hsp67bhs JMB 200:47 0 []

AAGGTATGTATGTATGTATAAAATGAGTGCAGATAGCAAGCTCTGTATTGGAGATCATACCATAAGATTTTAATTTCAAATTCAAAGTGAAATCGGATACAGATGAGAGGCGCAATCTCCAGTGTCAGCTGGACAAACAAGACGACACCACGATAATCAAAATGCGATAAGCAAGTACGCACATACATATGTACATACCCGAATCTTTAATCTTGAACCTCATAAATGGATCATCTGCGCCCAGCTGGCAAGTCAGTTGTTATTCAGCTGGCGAACCGGTTGAAATTCGTGCTCCGCCCCATAACTACAATGGCCACGTACGAACAGGTTAAGGATGTGCCCAACCATCC

>ID_1985 P_1998 Promoter_2034 D2_hsp83 NAR 11:7011 0 [-1 ]

AAGCAATCCCAGTGGGCTTTGCTCAATCGTTCGGACCACTTAGACGAATTTCCACCAAACTTAGTTCAGTATAATTTTTGAATTCGCCCGCACAGGTTGCGCACTTTTCGACCGTATCACAACACTGATCTACCCTAGTATTCACAGGAAGTTGCATCCCTGGCATCCAGAAGCCTCTAGAAGTTTCTAGAGACTTCCAGTTCGGGTCGGGTTTTTCTATAAAAGCAGACGCGCGGCGTTTGCCGGTTCGAGTCTTGAAAAAAATTTCGTACGGTGTGCGTCGTAACAACAAGCAGCGTCTGAAAAGTTTTGTGAATTTCCAATTCTATACAAAGCAAAGTGAAAATATC

>ID_1988 P_2001 Promoter_2037 D2_janus MCB 9:2526 0 []

TTTGTGTATATTTTGTTGTGAACGCCAGAGCTGTGCCGATAGTGCCGATAGTATCGACTGCGTGCTGTCGGCGTAATCGATAAATTTGCTGTCACTGATAACAACGTTTTCTTTTTGAGTTTATTAATTATTACTAAAATATACTGAGTAGTACAAAAAACGTTTTCCCAAATGTACTAAAGAAATAACTGAATATTATAATTTTAATAGTATCGATACATAAGGTGAACGAGAATAAAAGTATCTGGTCACATTGCTGGACTAAAGCAGCGTTTTTGGAAAATTTGCCGGTTGGTAAGACATTAAATTCTGTTTTCAAACACTTTTCCACAATGAATCGCCTCCAACTG

>ID_1994 P_2007 Promoter_2044 D2_lcp1 Cell 29:1027 0 [0 ]

GACCCGTTTGCCCCTTATTGATGCGATTGTTTCATTTTAGCATCTATTAAGCGATTATATATAGTACTTATCCCGTTGTTTGGCATTTGCTAAGCTGTCGCATGTGACGATGCTTTTTAATGGGTGTGGGCGCATCCGCGAAGTCAACCCATAACTCAGCGAACCAATTGAATGCAAGATGTAGCGTTTTGATATGGGTTCACTTTGGGTGGCAATCATATAAAAAGGCTCTGCCCGACCACAATCAGTTATCAGTCAACGTTCGTTCTCGACCAGACAGAAGTCAGCCAATATGTTCAAGTTTGTAAGTGTCCGCAGGATACGAACCAACATACTCGATCCCTAACGAA

>ID_1995 P_2008 Promoter_2045 D2_lcp2 Cell 29:1027 0 [0 ]

CCGTACTCCATCGCCCCTACAAAATTTCTACCGAAGCATGTTTCATTTCGGAATCTGTTCAGCAGCGCAAGACTTGTTTTTTGACATTTGTATCGCAGAGTCAAGTGGAGAATTTATGGGCCCTGCCTTTTGTTGGCATCATGGGCGTTTCGTGATAACTTAGATTTGGCCCAAAAAGTAATAAGCAATTCGTTTGGAAAGCAACCAAATTGGGAATCATATAAAAAGACTCTGTCGACCAAAGTCAGTTATCAGTCAACGTTCGTTCTCGACCAGACAGAAATCAGCCAACATGTTCAAGTTTGTGAGTGGCTCACAGGACATTTATGAACTCGCCATCTAATTGGTAT

>ID_1996 P_2009 Promoter_2046 D2_lcp3 Cell 29:1027 0 [0 ]

TGTACTGCTTAATAATTATTCTGGTTTCTGGTCCGGTTTGCTTTGCATTTCGGTTAGACTAGGGCGAATATTTCAGTTGAATAAATAACTAAGAATGCTCATCTCCTAATGAAAGTGGTTAAGCCATCTCAAGTCGACTAATTTGCATCCCAGACGGTTTTTATTATATGCATCACATTGACTTAATTATAATACGCACATTGCATCAGCTTTTGATGATATATAAACACCGATTTGAGCATAGATTGTCATCAGTCTTAGAAGATTTCTAGTCCGACAATCCACCCAAATCAAAATGTTCAAGATCGTAAGTATGCCTTGAGGAGCATAGTGACTTCGCAGTCTAATCC

>ID_1997 P_2010 Promoter_2047 D2_lcp4 Cell 29:1027 0 [0 ]

CCGAGTAACCCGATTCTTTTTAGAATAATTACGGTAGCGATTTGCATAGACAATAGAAATCAAAAAGAGTGCAGCAGACGATTTTTATCGCCACCAAGCATGTCACTTGAACCAGTCCGTAAAACCAAACGAGACCTATGCTGGCCGAAATGTTAATTAAAAACGGGTTGCATCAGCTTTTGATCAGCTTTAAGATTTCGTGGGGAGTGCGTATAAGCGTATAAAAGCCGACGAGTGATCCCGAATTGGCATCAGTCTCACGAGTTCTTTAGTCTGACAATCTAACCAAGTCAAAATGTTCAAGATCGTAAGTATCTGAAGTTTAAAGCCGGACAGTTCAATGAGTAATC

>ID_1999 P_2012 Promoter_2049 D2_lsp1 alpha JMB 189:1 -3 [0 ]

AACTTGTGCAGAAATTGCAGACAAAGAACCGAATCTAACGCTATTTTTGGAATGCAAATCTGGACTCGATATGTAGGGACCAACGTAGGCTGTCCAGAGATAAGCTGTTGCGGTGCAGTAGCAACAGATGTTCGTGGCGAGCACCTGAGATACACCCGATGATCCCTGATCCCGTCGTCACAGATATGTTATTTCCGATTACCGCGGGAATCTGTGGCTATATAAGCAGACCCGTGGGCGTCGGTAAGGCAACAGTCTTTAGGCATTGGATCCATCAAGTGCATCCGATCCGGAGCAGTTAAAGTTGAGTGTTTCGAACAAAAGAAGGAGTTTCCAGGATGAAGTTTGCC

>ID_2000 P_2013 Promoter_2050 D2_lsp1 beta JMB 189:1 -3 [-2 ]

TGCACCTGCAGAGCTGGCTCTTGGCAACCAGATGAATAGAAGTCTGGCTTTGATAAGAGACAAGAGGGTAGCAAGGCGATAAGAGGTAGTCCGGCACAGATGGACAGATGGCGCGATCGCAGCACCTGAGATACACCCAGTGACCCCCAAGCTGCACCGCGATATCTCCGAGCAGATTCCGAGCTCCGGACACTGCGATCGTAGGCGGGACTCTTGGGTATAAAAGCAGCCAGGTGGACGGCAGTGAGGCAACAGTCTTTAGGCATTGGATCCACTGGGTGCATCTGATCCGGAGCAGTTGAGTGTCAAGTGTAGAGTTGCAGGAATCCGTCAACATGAAGATCGCCATC

>ID_2001 P_2014 Promoter_2051 D2_lsp1 gamma JMB 189:1 0 [0 ]

GATCGGCCATTATACAACAGAACCGCGTTGAAGACGATCGACGAGGTCGTGGGTCTTATCTTATCACCACCTGAATTGAGGCATGCCTCCAGAATGACGAGGGCATCCGAAGATAATGTGGCCCGCTATTTTCGGCCGGGACTGGACCTATGCGACGACCTATGCTGATGACGGGAGTCTGCCGCTGATATGGTGCAATGCAAGGCTCCAGTCGGGGGTATAAAAGACCCAGTTTCGGTGCAGTCAAGACAACAGACTTTAGGTGTTGGTCGTTGAGCGAACCAAAGCCGGAGCAGTTGAGGAACCAAAGAATAGCAGCGAGAGGACCAAGGATGAAGTTGACCCTTGTT

>ID_2003 P_2017 Promoter_2054 D2_Mp20 JCB 108:521 -3 [0 ]

GAAATGTGTGAAAATAATGAAGCATTTGGATTTTTCCTTTTTCTCAACCGATGATGATCTAAAATTATAACGCACGATTCATACGCGGGAGACGGGCAAAGTCGGCGTTTCGAATATATAGAACGCCCACGCTGCAAAATTTTCACACACTCGACTCCGAATTCGGATATATTTTTGGAAAATTCTTGAACGAATTCGTCGGAGAACGCTCTCTCGCGCTTTAAAAGCCGCATACGCCCCGTTGGCTGGCATCAGTGTTAGCTTGTCAGAGCTTTAGTGAAGATCCCGCAGGACCCGAAACCAAAAACCAAGAATCAAACATGTCTCTTGAGCGTGCCGTTCGTGCCAAG

>ID_2004 P_2018 Promoter_2056 D2_Mst26Aa G&D 2:1063 1 [-3 ]

TAAAAAAAACCGAAGATATCCGTCCGGAGGCCAGAAAAATGTATATATCTTAATATCTAGAATACTACAGGACTAAAATGTATATTCAACGAATATTTATATATTTTGATGATGTTTTTTTTTTTTTTGAAAATTGCAATACTACAAGATCATATTTATAAATATCAAGCAGAACGAAACAAGAGCCGTTGCAAAAGCGCTTATGTAACATGAAATTCGTATATATAAGGAATGCAATAATACCTTACTTCAGCTGAAGCCACTTCTTCGAAATGAACCAGATTTTATTATGCTCTCCAATTTTACGTAAGTTGACTTATGCATTTTTGAGCACATTTAAGAACGAGTAT

>ID_2006 P_2021 Promoter_2060 D2_PCNA (mus209) MCB 10:872 0 []

CTCCTCCCACTTTTATTAGCAATCGTCTGCGTTAATGTATTGTCATTTTGTAATAATTTATTAATTTTAATGTTTGAAATAAATATACTCTGTAAAAAGTGTGAACATAATATGTTAATCAAACCAGTTGGCAGGCCGCTCGCTGGCTGCTATCGATAGATTCAGTCGATATCGCCCGTGGCTTTTCACATCCCTATCCCGCTCATTTAGCCCGCCTGAAAGTAAAAAAAAAAAACAGCCCACGTTAATCATTCATCCCAAAGTCACAGCCGCGGTAACATTACTGCTGTTAAATTCTTAAGCCCGTCATCAGTATTTAAATAATAAAACACATTCAATATGTTCGAGGC

>ID_2008 P_2023 Promoter_2062 D2_Pgd Gene 109:177 1 [-1 ]

GAAGCACTATATTTAGTAGCAGTACTCATTAGAGTCCACTCACAAAATTAGCACCAACCGGCAGTAATTGGTCAAGGATCGGCGATAGCTTCAAACTCCGAAGTTCAAAGTCAAACTGCCGCCCTGCGAAAGCTTCGCGAGTGGAGCTTTTCTGCACTTATCGATAGCTAACATTGTGGCGCGACTATCGATCGACGAGCTGCCGCTTAACAGTGCCATATATAGATTGTAACATTAGAAGCTCAAATCATTGTTGGAGCACAAACCACAAAGAACACACGAAACATGAGCGGGTGAGTAGAGGGAAATTCTCTTTTCCCCGGAGTTTTCCGCGATCCTAACGTCGCCCA

>ID_2018 P_2034 Promoter_2074 D2_rbp9 p3 JN 13:1045 0 [0 ]

TGCGGATATATGTGCTCGTGCTGTAGGAAAACATATAGCCGTGTAAGTTTGATCCATTCCCATCGGGTGGCTTTCTCTTCCCCGAGCGGGCGAAGAGCGCTCGACAATGACGAATTCTGTTTCTGGCCAAGAAGCGGCGCTCTCTTGTAGCTCTTGGGGCTCTGCTCTCCCGATTCTTGGACATGAGGCAGCAGTACGATTGCCCTCGTCGGTGGGGCATAAAAATAATTTCCACGCCGCTCGGCAAATCAGTTTATTTCTGTTCTGTTGAGTTGCAGGAACGCGTTCTTTTCTACTTTCCGTTCGGTTTTATTTCCTTATTATTATTGTTATTACGAATTAGTTTCACG

>ID_2028 P_2047 Promoter_2087 D2_s15 Chromosoma 101:538 0 [0 ]

TTCCCATTTGGAAGAGTCGCGTGTTCGTAGTGCTATCACTACCCAACACCCGGTAGAATAGCACATCGCGTAACCAAGCGATTTTATAATGGCTTGACAACAAGTACATAAATCAAATGTGAGTATATTCCAGCCGGGCAATTATGAAATGCCATTTCTGGGCTGAAACAGAACAATTAGTGTATATAGGTCACGTAAATGTCCAGGCTAAAATTTGCGTATAAAAGCGAGCGTTTCTGGTCGGTAAATCATAGTTTGATTGATTACCCCAAACCAACAAAACTAAGCACTCACCATGAAGTACCTGGTAAGTTGTGGTAGTCCCCGTGAAGGAGTGGCAGCCAACTGAT

>ID_2029 P_2048 Promoter_2088 D2_s36 EMBO 6:1045 0 [0 ]

CGATCTTTGGAAGAGCTATGATTCAAGCCAAGGGAAACAACTGCCAAAAAATAGAAGATTGCGACGAGCGGAAAGCAGAGTGGTGCACCACGGTGCATAGGTGCATAGGAGGTTGGTTGTCTAGAGATCTGGGCACGATGGCGAGACAAAGATGCGGCGCAAAATCGGAAATGGAGATGGATCACGTAGCCGGCCATGGCGGGCAGCGATTATAGGCGCTATAAAGAGCCGGAGTCATCGCCAGACAGCGAGCAGTAGACGATCCACACGTAAACGGCAACATGCAACTCGGTCTCTGGTTTGGCATTTTGGCCATCGCCGCCACGCCGGTTAGTAGCTTTTATCCATGG

>ID_2030 P_2049 Promoter_2089 D2_s38 EMBO 6:1045 -3 [-1 ]

GGGCAATGGCAACTGGGCAGTGGGCAGTGGGGTTTTCGGGTTGTGGCTTCTACGTAAGTGGAAGAGACGCCGTGATATGCGCTGGCAGCGATGCGTGCGATCTATCAACATTTGGCCATGTTTCGTTCGCATCGCGTGGGCCCGGAGCGGAACAGCCGGCACCGGAGTTGGCATCAATCCAAATGTCACGTACCCGGAGCCGGAGACGCGTCCGGAGCATATTTAAAGTAGTCGGCCACCAATGGAGGGCAGCAGAAGACAGCAGACAGTCCAAGCGGGAGCACACCAGAAGCCGAAGAGCAACTGGAACTGCAACTGGGAGACAAGATGACGAGATCGACCTACATTTG

>ID_2034 P_2054 Promoter_2095 D2_sev p3 Cell 54:299 -3 []

AGTTGCTGCGTCAACGAAAAAGAAGATCAATGGTCATTTAAGATCGGTTCGGTTCAGTTCGGTTCGGTTGCGGTGCGCTTCGTCACTCATCGTTCATCGCATCGTATGGTATCGTATGGTATCGTATGGTATCGTATCGCATCGCATCGCTTCGGTTCGGTTTCAGTTCCGTGCTCAAGTTCTCCAGCTGGGGGGTTGGACTTGGACTTGGAGCTTGGGGCTTGGTTAAAAAGCCGCCGCTCGCCGGCAAATCAGTTCTGCTGCGATCGCGCCACTGCGCACTACGTAACGCTTCGGGAATCGCACATCCAGATCCAGATTCAGATCCACATCCAGATATCTGTGACATT

>ID_2035 P_2055 Promoter_2096 D2_Sgs-3 JMB 168:765 0 [0 ]

TTACCGATATTCGTGAAAAATGTTATACATTTTCCATTTCACTTGAACTAGCTAAGTAACGGGTATCTGTTAGTCTCGTTAGCGTTCTCTCTTGTTTTAAAATAAAGTCCAGGCGATCGAGTCGACCCAAAAGTATCAAACAAAGGGGAGAAGGCTTGTGTTTGCATAATCGAAATACTGACTCCATTTTTAGAATTGCAGTTTCAGTGAAAGCGTACCTATAAAAAGGTGAGGTATCCGCAAGAAAAGTATCAGTTTGTGGAGAATTAAGTAAAAAACATGAAGCTGACCATTGCTACCGCCCTAGGTAGGTTTCACCGAATGCTCTTGTTTTCGGTATTTGAGCCACT

>ID_2036 P_2056 Promoter_2097 D2_Sgs-4 Cell 29:1041 1 [1 ]

TCCATATATATTGAATTTAATATTAAAATCATTAAACAAATGGGCTTTAATTACAATGCGCAAACAATTTCAACAAGTTTATCATGATTCAGCATACCCTTAGCTGAATTATATTTGTATATCGCTTACTTTTAGTTTCTTCATTTTAAACCGTAGAAATTGTTATTTTTTTGTTGAGTGTATCTTAAATTGTTCGATGTGTCGATGGCAAAATGCAGTGGGTATATAAAGCGCATCACGCGGTATTGAATTCCAAAGTCAAGATGCGCTTGGAGTTGTTGGTTGTGTTATTGGTGGGGCTGGCTGCACTTGCCCCGTCAGGTTCTACATGCTGTAAAACTGAACCACCG

>ID_2037 P_2057 Promoter_2098 D2_Sgs-5 JMB 190:149 1 [3 ]

CGGTGCATTATTACCTGATTTTATAAGTCTTCGTTGCTTATGTTTTTTAGAGTCTGTTTTTAGGAACCTGTGTGATAAAGCTTTTTTTTGGAGTGGAAAATTTATGGCTGTGTTTTTTTGGCCAGTCAAGGTTGTTTGCGTACGTTCTGCAAACATTTTACTTTCAGATGCACTAAGTCAATAAAGCGCTTTGCCACAACTGCTAAAACAGTGGAGTGTATTCAATATAAATAGCCAAATGAGATATTATGGGGACAGTTATATTCTTAGCCACTTTTACGACATGTTCAATATTAAATTGCTGCTTTTGTTATTGGCCGTTTCGTGGTTCCACCATGGACAAGCCGTCC

>ID_2038 P_2058 Promoter_2099 D2_Sgs-7 JMB 168:765 0 [-1 ]

GAAAGTATTACTCATAAAATGAAATCTAAATTATATCTGAGTAACAAATATATTAAATTAATAAGTATCTATAAAAAGTTAATTCTATAAATAAAGCGCCTGCCGTATAAAAAGCCAAGTGTTTGGTGTTTTATTTATTTTAATACAATTGGTTTGTCCAGTACTTTTTATTTTTGGATGTGCTCACTGAAATTTTCCATTGATCCAGCTAACTTTTTGCGCTATATAAAGGTGTTGCTTTCCTTGAGTTGGTACCATCTGGTAAAGTAGTCTCAATCTAGATAGAACCATGAAACTGATCGCAGTCACCATCATCGGTAACTACATAATAAGATCTTTAATCCACAACC

>ID_2039 P_2059 Promoter_2100 D2_Sgs-8 JMB 168:765 0 [0 ]

CAGATATAATTTAGATTTCATTTTATGAGTAATACTTTCTTTAGTAAAAAGGGCATGTAAAATGTTATATTATATTAGTATGAACGATGACACAGAAAATGGTGAGAACAAAGGGCGAAGTTTAATATCGTGTTTCTATTTGCTTAAGCACAAATGGTTATATATTTTATTTTTGGATGAATTCAATGAAATTTCCATTGATCCTATAACACATTTGTTTTGCTATAAAAGGGGTAGGGTGTTCATAAACGGTTACCATCTGGTAAAGTTAATTTGTTAAAGCAACAACCATGAAGCTGCTCGTTGTCGCCGTCATTGGTAAGTGCCAAAAAGTACTATTTTTTATGTGA

>ID_2044 P_2064 Promoter_2106 D2_sryAC JMB 86:149 0 [0 ]

CAGCTCATGGGTGCATTAGCGCGCGTATGTATCATAAAATTAAATCTAAACAATTATTGACGGAAAGTACCAGTTCTTTGCCGTTCTTCGCCCATTTTCCAGGAACCCCAGTAGGTAAAGTAGCGGATTTCGCGAATTTTCGCGGGTATGGCAATAAAACAGGCAGATGTTTTTTAATCCCCAAAATAGGTCCTTTCTACCTGTGCGCTTGGCAAAGTATATAAAGGTGTTGCGTCGTCCGCCAGAACTTAGTTGAACATTTCTGTTTCCCGGAGCACATCTGATAGAACAGCATGGAACAGCTATTGGCCCAATTACACACTTGCAGTGAGCTGATTGCAGAGGGCTAC

>ID_2059 P_2080 Promoter_2124 D2_ubiquitin MCB 8:4727 0 []

GGTCTCAATTTGAAATTGTCTTAAAAATATTTTATTGTTTTGTACTGTGGTGAGTTTAAACGAAAAACACAAAAAAAAAGTGATACATAGAAATCATAAAAAATTTTAATACAAGGTATTCGTACGTATCAAAAACATTTCGGCACAATTTTTTTTCTCTGTACTAAAGTGTTACGAACACTACGGTATTTTTTAGTGATTTTCAACGGACACCGAAGGTATATAAACAGCGTTCGCGAACGGTCGCCTTCAAAACCAATTGACATTTGCAGCAGCAAGTACAAGCAGAAAGTAAAGCGCAATCAGCGAAAAATTTATACTTAATTGTTGGTGATTAAAGTACAATTAAA

>ID_2060 P_2082 Promoter_2126 D2_uro MCB 10:5114 0 []

CAAAGTTTTGGAGATAGAAAACATAGCCTTGAGTCTTGGTCATGTTTACTTGGCACCAGGCCGCGATTATCAGCGCTACTAGTCGTAATTTGAGTTAGACCTTTAATACTCTAAGTGAGAGTGATGATATACGATTTCCCAGCCACTTGCTTTCTACGAAATGCGCTAAAAAAAATCCCTAACTACACAAAGATTTGTGTTGTTATCCAGGTGTTCTGATATAAAAGGCGGCAAGGAAATTGATGGCATCATCAGTATCAAAGTGAGAGTGATTGCAGTCACAATGTTTGCCACGCCCCTCAGACAGCCAGCTGCGGCTAACCACCAGACCCCAAAGAATTCCGCCGGCA

>ID_2062 P_2084 Promoter_2128 D2_Vm26Aa Gene 136:121 0 []

TTTTAAAGTGCAACGCACTAGATATCAACCACAAATCGCCAACCTAAACGTTCCTCTGCCCGCTGATGACCCCAAAAAGCTGTCGATGTGAAGCTACTAAAGTCAGCGGTATACAATTGTCCATTGGGCTGATGGAGCAGAAAAGTAATCAAGGCAAAAACGAAGAGTAGACCTAGCCATTATGTTCTGAACGTAGTGCTAACTAGGCAGATTGGACAGCTATAAAAGATGGCCAATTGAATGGTATTGGCATCAGTCACCTTTGGTAACTACCAGCAGCCCAACCAGCTCCCATCCGCCTCCAGCTCAATCTTCAACCACCAACAACCAAGATGAAATCCTTCGTGTGC

>ID_2063 P_2085 Promoter_2129 D2_Vm34Ca Gene 136:121 0 []

TTTAGTTCCGTTACTTCATTAATTTACATTTTTGGCATGCGACAAATTGTTTACTCAACAAGTTCAGTGGCCCAAAAAAAGTAGAGGAAATGTTTGTTCTTTTCGCTTTCTGTTGGCCGTGCAAAAAAAGCGCCACTCACGTCGACTTCGAGGGGTCGTTGGGTAAACTGAAAACTTGGTCAGTGCTTGCATCTGCACTTTTGATGGCATTGCATCGGGTATATAAACCGCAAGTGTCGAAGCCAGAAGCATCGCAGTCTGCTACCAACAGTCTAAGAAATCATCAACCAATCAACATGAAGTGCATCGCCATCGTCTCCACCATCTGCCTGCTGGCCGCTTTCGTTGCC

>ID_2064 P_2086 Promoter_2131 D2_yellow EMBO 5:2657 0 [0 ]

TGACTCATTAAATTGGGGGATTCGAGTGTATTTATTAAACATGCGTGAAAATCAATCATGGAAGACAAAACGCAAAGTTGGCCGATCTATGGGAACAGCATAAGCCACCTGATTACCCGAACACTGAACCACCCGAATCACTAAAACCACCGAAGTTGGCGCGCGCCTTCGTTTTCATTTTCATTGGCCTGTCTTCGTCTTCGGAGAAAAAAAACTTCATATAAAACGCGGCCGACATATTATGGCCACCAGTCGTTACCGCGCCACGGTCCACAGAAGAGGATTAAAAAAATATCACACAGCCGAAGGCTAGAGAAGAACCCCCTATAGCTGAACATATATAAACAAAT

>ID_2072 P_2106 Promoter_2153 D3_EP23027 (+) Dm[Gart] cuticle p.; range -250 to 49. []

AATAACGCCGTTGATACCTATATAAAATAATTCCTATAAGAAATAAAATCATTTTATTTATGACTGACTAAGGCGACCAGCAGCGATGAGATGTTTGTAGATGGAGACGATCATGACGATGACGAGCGGAGATGGAGATGGAAACGGCAACGGCAACGGCAACTCGGAACTGGGTTTCCGAGGCGATGTATAGCCAAAAATCCGCTGGTGAGCGGATGGATATAAAAACGAAAGCGTCCGAGAAGCAGGCAAGCAGTTTAGAACCAAACTCGAACGCGACACCATGTATTTGCTTGTAAGCATCACCTGGGAATTTCCCGAAATTGGATTATAATCGCCGACTCTCGTCT

>ID_2073 P_2114 Promoter_2161 D3_EP11027 (+) Dm chorion p. s19; range -250 to 49. []

CGAAGGTCAACCGGCGAATGGAGGCGATACGATACGCTGCGGTGAGCAACCTGCTCGAGCCGAAACGAGCTCAACGTGGAGCCCCGATATCTGGCTAGGAAAAGCTAGAAATCCACAGAAAGTTCCCCAACAAACTGGCCGAGAAGAGACGACGAAGCCAGCTCTTGAGCCGTGATAAATTTCTGGGCGAGATCACGTTTCGAGTGCAACAATAAATTTGCTTATATAAAGAAGTGTGCTTGGCCATTTAATATGTTAATTCAGCCAACTGTGCCAAAACCCATACATCATAGCCATGAACAAGTTCGCTGTAAGTGTCCCTGAGAACCGCTTCCGTATTCCCTGCCGCT

>ID_2074 P_2115 Promoter_2162 D3_EP11025 (+) Dm chorion p. s18; range -250 to 49. []

AGACGATGGCGTAAGCACAGACGCCTGCTATCTGGACCGGCCCGAATTGAGAGCCAGCATTTTGGCCAGTGCGGATTCGGCCTGGCTGCACGTCTCCGGCGGCGTCTCAAGATTGCTGGACAAAGAGGCGAGGCCTGGAACTGCGTCTGCGGGAACCCGGAGAGCCGAAACTTGCATCATATTCGTCACGTAAGAGTTGGGCCTCTGCCTGGATCTGGTATAAAAACAAAACATTGCGCCAGAATAAGACATTAGTTACCTTCGCATCGATCAACTAACCAACTCAGCCTCAGAATGATGAAGTTCATGGTAAGCCTTAAGTTCCAATATTGTTTCTATTGACACCTCAA

>ID_2076 P_2119 Promoter_2166 D3_EP07022 (+) Dm YP1_1; range -250 to 49. []

AGGCGGCAGCAGGTGCTGCTAAGTCATCAGTGGGGTCAGCTATAGGTAGGCCCCGTGTCTATTTTGTATGTATACAATTTATTCCGCTATCGATAGCATATACACTCATCCGATTCCCAGGCACCCGAAAACCCTTACTCAGCACAAGTGACCGATTAAGGCCTGAGCCAGCGAAAAGCGAGTCAGAAAATGGGAAATCGCTCAGCGCAAATTGTGGTATATAAACCACCATCGTTGGATTTGGAAGGCCAGTTCAACTCACTCAGTGTTGAAGTCGCATCCGCAGGACCAAATCCCAAATCCGAACCATGAACCCCATGAGAGTGCTGAGCCTTCTGGCTTGCTTGGCG

>ID_2082 P_2141 Promoter_2190 D3_EP24020 (+) Dm calmodulin; range -250 to 49. []

AGTATGTTGATACAATTTTTGTACACACCTATTTTCGCGAAAAGCGCCGGCGCGACCGTTACAACGGCGAAATCAAACTTATTTTCGCTTTACGAAGGTGAGTGGATAAGCAACAGAAACAAACACATGCAGACGCTGCCGCTGCAAAAGCGAACTTACCCGGTTCGTTCTCGCGAGAGCGAGAGCGGTTATTGCTATTGCTGCTGGCAAAATAAAACCGTATATATGGCGACGCAGCGCTATTTTGAACACAAAGTTTTGAGTCGTCGACCGTTGCGGACGGAAATTCAGATTAGGATCGTGAGTTGTCAGAACAAACAGACCAGACATTGCATATAGAGAGCAGTATT

>ID_2083 P_2144 Promoter_2193 D3_EP15016 (+) Dm[eve]; range -250 to 49. []

GCCGAGCAGAGAACGCGGCTCCATCCCGCTGCTCCGGGTCCTGCTCCTCCGCTTTGTCCCGCCTCGTTATCGCCGCTCAGCACCGAGAGCACAGCAGCGCATCCACTCTCAGCACCGCACGATTAGCACCGTTCCGCTCAGGCTGTCCCGCTCGCACCTGCCTGGGTCGCTGCGATTGGCCGCTCCCAGCGACGGCGGCCATTTGCCTGCAGAGCGCAGCGGTATAAAAGGGCGCGGGGTGGCTGAGAGCAGCACACTCGAGCTGTGACCGCCGCACAGTCAACAACTAACTGCCTTCGTTAATATCCTCTGAATAAGCCAACTTTGAATCACAAGACGCATACCAAACA

>ID_2084 P_2145 Promoter_2194 D3_EP23030 (+) Dm[Krueppel]; range -250 to 49. []

GGAGAGCCAATTGAGTCCCGGCGTGTGTGCGTACGAGGGTGAGTGAGAATCTTTCGCCGAGACAGAGCGTACTTATAGTTAGCTCACGCAGCGAATTTGTATCAGTCGTGATTTGGCTCTGTCAGCGAAAGGAACAACCATTTGTTGTGCCACACACAACCGCGAGTAATATAATCGAATCAAATTTCAACTACCTCATTTTGCTAAATCGGTAAACTTTTATAAAAGACAATTTTTGTGAAATCTCTCTACCTCAAAGTACAAAAGTGTGTACAAAAATTATTCATATCCCTGAAAGTGCACAAATTGCTCAAATGAAATTTTGTTGTCTAAAAAACTGAAGCTCCAAA

>ID_2090 P_2170 Promoter_2219 D3_EP70006 (+) Dm cecropin B; range -250 to 49. []

TTGTTTTTAATTGTAGTTTTACGTTATTTTTAAGCTAGTTTAAATCATCATAATTCAATAGATTAATCAAATCATAGCTTGCAACCAACCAGTTACTCTGAAATATCACTTGAGTAAGTCACTTTCATGGCGGTTCCGAACTGAGTCCATCTGCTGGTGAACTTTTGTCCCGCAGCAAAAAATTCCCGTCTGTGCAGCCGTAGCATCTGTTGGTATCGCTATATAAGCTCAATCTCTTCGATGTCCAATCATCAGTCGCACAGTTCTCACTGCAACAGCTTAAGCTTTCTTTCAATCCGATCGTAAGCCAACAATCTCGTCATGAACTTCAACAAGATCTTCGTCTTTGT

>ID_2091 P_2176 Promoter_2226 D3_EP07031 (+) Dm hsp 68K; range -250 to 49. []

GCTTGCATACGAATTTAGTTTAATGACCACTTGGAATGATGACCTATGTCAATAAAAAAAATATATTTCTCGAAATTTCCTGAACTTTCAGTCCTTTCAACTGACAATTTCCCCTGCGAAGTGCACACTAGTTGACTCGCTCGCACACACACGAACTGACTGGAATGTTCTGACCCTTTCTCGCAGGGAAATCTCGAATTTTCCCCTCCCGGCGACTGAGTATAAATACGGGCGCAAATTTCCCAGACGCTACATTTGAAATCAAACAGCCAAAGTGAAAACACTTCAGCAAACGAAAAATAAAAAGCAGTTCAGAGTGACAGTTGAAAACTGAAACGTCTTTACCAAGT

>ID_2099 P_2188 Promoter_2241 D3_EP77013 (+) Dm SAMS_3; range -250 to 49. []

CCGGAGCGGGGTGGCAACGCTGGGCGGCGGAACAGCTGCTTAAAACCCAAAAACGTGGAGATACTGAAGAAGTAAAATCATTTGAGAGTACCGAGAACGCTAGGAAAGTTTCCCTAAAGAGCGCCCAAATAAGTCTCAAAATAATAATCGAGTCAGCCGAAGAGAACCGCCTCCTCCGTTTGCGACGATAAGCAAAAGCCCGCTCTCTCGTTGGAGGTATAAATTCTGAATGGCATTAAAAAGTTGCGCTCTTTTTCTTCAGAACCGTCGCTAAGATAGGACGTGTGTTGGCTGCGCCGCTGATTTGGTTGCCGTCGCTGAACCAGTATAACTCAGAATCAGTGTGTATA

>ID_2103 P_2192 Promoter_2245 D3_EP77017 (+) Dm BCDNA LD21248; range -250 to 49. []

TTTTGTTGCTGCCCAGTGTTACCAAGTGGTTGAATTCAACTGAATGAAGACGTTCCCACTGCCTGTTTATATTGACGAAAAATGTTATATAAAAGGGATAAATCTATTTATTTTCCCTAGGAATAGTAACTGCGAATAGCATTTGTGTTTTTTAAATGTTTTTTATAATATTTCCCAGATATTTGTTTTTAAGTACAAATTTATTTCCCTAGTTACTCGATTGGTATAAATCCAATGACCTGCCACGGTCACACTAGATTCATAGTTCGGAAATTTGTCGGTTGCTGGAGTTTGTGGACGAATTTATATTTAATTTTTAAGCACTTGGTAATTAACTAATCGACGTTCAT

>ID_2108 P_2197 Promoter_2250 D3_EP77022 (+) Dm CG7611; range -250 to 49. []

TTTCTATAGCTGTGCCGTTAATTACTTAAAATTTAATTATATTTCAAAAATAATAAAAAATCATGTGGTATAGTTAAGTTGAGTTAGAAAAAGTGTTCATTTAAATTTGAAAAGCATATTAGCGATACGGCCACTCAAAGTAAGAAATCTGGTATTTTTATCGTTCGTGAACAAACGAAGTGATTCGGTATTTTCGAAAGGTCCCACTAAGTGCAGCATATATATAAATGCGTTTCATCATCAGATTTGTATGTATATTGTGTCAGTTCGCCTTTGTTTTTTAATTGCTAGTCCGTCAATTAAGAAATGCTTTTACGGTGTGTGTCCAACTAATCGGGATAACCTTTCCT

>ID_2127 P_2222 Promoter_2277 D3_EP77049 (+) Dm CG17238_2; range -250 to 49. []

CCACAAGTGAGAGATAGAGAGAGGGAGAGAGCATGGAAGAGAGCGGGCTCCAACTCTAATCAGCATCAGCTGTAGTTTGTTTTTGTTTTGATGCGCCACATGGCGGGCACTCACTCTCTTGCGCTCCCGCTCTCTTCCCGTGCAGCTTTTGCGCTCTGAGTGAGTTGGGTTTCGGTCGTTGTTTTTGGATCGATAAAACTTCTCGCGCTGCCGTGGTATAAAATGCTTTTCACTTTTCCCACTGCACTTTAGTCGCCAGACATTTATCGAAACGTGCACATCACCAGCGGTCGCACACTCAAACATATTGCATATATATATATATATATATATATTGCTATCCCAACAAA

>ID_2152 P_2251 Promoter_2307 D3_EP77079 (+) Dm APT_2; range -250 to 49. []

CCACATTTAAAGCCAAATACAACGCACACTAGCGAAGCGCGAGAGCGCCGCTCCCGAATTGCTCTCTGCAGCATGTTTCGTTGCTGCACGAAGCGCACACGAAGTGTCTTCGGCTCCGCTCGTGCTTGTGTGCGTTTGTAAATTGATTTTATTAGTTTTTCGTTGTTGTTGTGTGTTTGTCGCTGCCGCAGTCGCCGTCGCCGCTGGCAGAGCAGAATGATTTTAAAAAAATTTTGAAATGCGGCCCTTTAGTCAGTAACTAAACGTGAAGCAGTGCGAACGCGCGCAGCGGCACAATTCCAGCGTTCTTGGCTTTCATCCGGCGTTCTTCCCCCATCAATCACTTAAGA

>ID_2158 P_2258 Promoter_2314 D3_EP77086 (+) Dm CG5547_2; range -250 to 49. []

CGGTACTCGAAATTGTGAGAAAAGAAATGAAAGAATCTAAAAAAAAACCCAAGCTAAACACACCGCACTTACGCCCCAAAGAAGTTCCCATGCGCCCGCGCGCTGATAACGCAAGTCTGAGAATCTGAGACCAATTCCGCTCCGAGTCTGAACGTGAATCCGAGTCCGAAACCGATAACGAAAACTGAACTGAGTAGCTCGGTCGTGAAATGGGAATTGATATATAGCGGGCGTCGTCGTAGCTCAGAACAAATAACTTACAGCTCTCGAAGCGTGAACGTCGCTGTCGTCGAGTTTCCGCCTTCAAAACGAGCACTGAAAACAGAAACATACTCTCCGGAATCTAATGT

>ID_2159 P_2259 Promoter_2315 D3_EP77087 (+) Dm REGUCALCIN; range -250 to 49. []

TCTGTTTTGGAATATTTTTTTTTTTGCAATTTGCCATCGGGTTAATTTGCCCGTTGGCGACTTCTGTTGCTCTGTTATCAGGCCGGGCTATCCAGCCCTCCAATTCTCCGCTTATCAGTGGGCGAAGGAGGAGGAGGTGAAGGTGGAGGCGGAGTAGAGGAGGTGGAGGTGGAGGAGGTTTTGGCGAGATTGTGTGTCACGCCCGTGCCAGCTGGGGTATATAAGAATTGGGCCGGATCGGGTGGCATTCAGTTTGAACGCGCTACTCAACGGAGCCGTGTCCAAGGATTCTCGATTCTCTATTCTCGATTCCCCATCTCTCAGCTCAAGCTTAAGCTATCTCCAAGGTA

>ID_2180 P_2283 Promoter_2339 D3_EP77111 (+) Dm BCDNA GH04962; range -250 to 49. []

ATTTTGATTATTGGAGTCTTTCTCATTAGCTCTTAGCTTCTCTGAGAATTTCGAATATCTGCTTAAAAATAAAAAGCTTAAAAGGGTTTTGCTGCTTTTGAAAAAATAAGAATTGACATTTTTTAAAATTGGAGCCATAAAATAAACGTACAGGCACACTAATAGTAGGGTTGGTATTTTTTGCTAAGTTTGGTATTAAGTGCACACTTCGGCGTATGTACTGCATATTTAAACAGCCAAGCGACGGTCACATTGCTTGGGTCCTCTGCAAAATTTTTAATGAAAGTTATTAACCAGAATTCAACCGGAGGTGAGCTGTTTGAAACTTAAAGGTTTGGAGTCCCACGAAG

>ID_2195 P_2299 Promoter_2355 D3_EP77127 (+) Dm LIM3; range -250 to 49. []

ACTTTTTCGAAGAATCGAAAAAGTTTAAAAACCGCAGATGCATACCAATATGTTTCCATCCCACTCTCTGGCGTTATTTGATGAAAACTGAAATAAAACGAAGGAAAACTGCTCCGTGTTGCCGCATCTTAAGTTTCTCAACTCGGTGTGCTGAGCAGGAAAACTCAGTGGCAGCGGCGAAAAGTCGACCGCGTTGCCGTGCCGTGTGCACTCACAATTATATAGACTAGTTTGCTTTGAGCAATCTTCCAGTTCAGTACTCGGCGCTTCCGTGTGTGGTTTTAGTGCGTTGTAGGTTCCGTTCTATCTTGGCTATAATACACTCACACCCAACGGTTGGATATCGCCAG

>ID_2241 P_2358 Promoter_2415 D3_EP77188 (+) Dm VHA16_1; range -250 to 49. []

AGTTCATGAACGAATTGACCAAGCTATCAGAAATATAAAATAAAATATTAATTTTGATGTGCTTATGTTCTTCAATTTCGTGCATCCTTTTAAAATCGGTGTGAAAGCAATGCTTTAATTGTATTGGCTCTAGTGGAAACATTGTTAGTGCATTTTGACGTCCATCAGGTGGCAGCGCTGGCACAACTATCGCCGAAAATCCGCCCCCGTGACTAACGTTTATAAAAGCAAAGCATAATAACTCGTTGGTGTTCAGTATCCGATCCGCGCTTTTAAGCAGCAGCCAGCAGTAACCCGAAGAGGCCACAGAGCAGCGAATTGCTTTTAATTAGATTTCTAATCACGTGAGT

>ID_2242 P_2359 Promoter_2416 D3_EP77189 (+) Dm VHA16_2; range -250 to 49. []

CATTGCCAATTGGACTTACTATCCGTGATGTGTGCCTGTCATGCGCTCACTCTCGCGCGCACACAAGCACATGACAACATGCACATGCACACAGGTCACACTCCACACAAGCGCAATAATCCGACGAGTGGCCTCTCGCTCAATCTCACTCTCTCTTGCTGCTCTCTCCCAGTTTTCGTTGTTGGCCACTCGCTCCGACTTGCCAACTCTCGATTAAAAAGTATATTTAAGAATATTCCGTGAGCGGCCACTCGCAATTCGTTCGTCGTCGCAGCAGCATTGGTCGCAGAGCAGCGTAAAACCCCACGTATATAGCACATATATTGTACATATAATAAAAATACTTTGTT

>ID_2245 P_2363 Promoter_2420 D3_EP77193 (+) Dm CG8588; range -250 to 49. []

TCGCTAGAAATACAAAGCAAATATCTGATTAGCATGAGCTCATCGTCTCGTTACATTTTGGGTTTAGCACGCTAGCAGCTTTTTAAATTTTGCTCACCAAAGAAGGTGACGGTGGAATACGGGGTATTCATATAAAGGTATGATCCCTAGAGAGCTTTTCTGCCGTCTTGCCTTTTCGGTATTTCGAGTACGATCGTTCAATTAGTGTGCGCCTTAACTTAAAAGCTGGTGATTCCGTGGCACAACTCCATTAGCAGCACAACTGTGGCAACGGAAAGGTGACAACTTAGGGATGGCGAATAGATTAGGAAACCATGAAGTTACCCGCACCGCACGTGAAGTGCAGCAAA

>ID_2268 P_2390 Promoter_2448 D3_EP77222 (+) Dm CG4847; range -250 to 49. []

GACGAGTTCCCAAACCACACGGCGAATATCGCTGGATCGGTCGGATCGCACACATCAAAACAGATATAGCACATACTCGTATACGATATGCATGGTATAATCAACGCGTTTCGATTTCTGTATAATTCCGAATCACTGGACTTGGGATTTATGGATTTGAATGGATCGGGTGGGGGCGAGGTTGGCCAGTTTATAGCCTGAAACCTACTAGTCTAATGGCTATATAAGCCCCGATCGACGGTCGAGACAAAAACATTTGTGTGGACCGTGCGAGCGCGTTAGGTTAGTCTATTGGGAATTACTTGCGATTACTCGCATCAGAAAAATTTGTGCAATAGTATCTCGTATTT

>ID_2273 P_2398 Promoter_2456 D3_EP77230 (+) Dm CG7010_2; range -250 to 49. []

GAAAGCTGCAGTATTTTATTTAGGGTCACACTAATAGGCCAAGTGGTGCGTTATTTTTCGCTATAACGCCAAACGGTTGTTATAGTTGTGCAATATAGTAGTTGATAAACTAATCTCGCTACCTTTTTTTCCGATTGTTTAACTTACAACGACGCGCGGCGGTGCGACTGAGACACCAGATATATAGCGTGCGATTCTAGATCCGGACAATCCGCATATAGAAAGCCCCCATTCACATCCAATTTCTGTCAGTCGAACGAGAATAACACAGCGAAAATGTTGCGTACTCTGTCACGCGTTAGCGAATTGCCCATTATTGTTAAGCAACTGCAAAAAGTACGTAATCCCAA

>ID_2278 P_2406 Promoter_2464 D3_EP77238 (+) Dm TRXR-1_1; range -250 to 49. []

TAAAAAAGCAGTTTATATATGAATAATTTTCCAGTTAAATTACTGTAATTTTTAGAGGCAAATTTGGTATTTCATGAGAGTTTAAATAGCGCGCCCAACGAGCGCGTTAGTGCCATAATAACCGATAGACGATAAGTGCTGCTGGTGTGACCGAACAATAGTCGGCCCAGCAATCGACAAGCCAATCGACGTCTCCCTTTCGCACGCTCGTACGAAAGTACAAAAGCTATTGCAAAAGTTGGCTCCGCTTATTCGTTTCGTGCTTTCGCGAGTGCCGAGAGCCGCTACAATACACGCTTAGCAGTTTTTACATTTCCGCTTCGACTACAACAACATTCACTACCCGCCGT

>ID_2319 P_2452 Promoter_2511 D3_EP77285 (+) Dm GUF_2; range -250 to 49. []

TGGGAAGTAGGGGGCTCCATTTAACCTTGGGGCTCTGACTCACACCGCACCCCGATTCAGATCCACATCCCCTGACCGACCGGGTCGACTGTACCCGTGCTCTCCGGCACATTTGCATATCTGTCGGATAGAACTAGGCTATTATACATTTGTATGTATGTATGTACGTGTATGTAGATATATCGTGAGGCCTCCTAGTCGCTGCTACTTAATCAGATAGATAAATGATGCCGCCACCGTTTGGATGTCGTCAGTCGCCAGACAAACGCCCAGAGCACAGCACCACACAGATCATCAGCGAGCAGTCTCCACATCGCCAGTGGAGGAGAGGCACGGAAACCGGGCCGCCA

>ID_2329 P_2463 Promoter_2522 D3_EP77299 (+) Dm SN; range -250 to 49. []

AACCATTCGATCGATATCGATATTCCTTTGTGAAGGCTAAAAATAAAATTCTTACATGCTCATCTTATACAATAATGGATCGCACATATAGCTACTCCTTTTTCATGTATCCAATTCACTAGGTAAGCTACAAAGATAGCCAGACGGCTGTGCTAACAGAGCGCTGCTATGGTGAGAAGCCGCCGCAATGGCAACAGTGCCATCTCTGGCCGGCGATACAAATAGCGCAACTTCGCTCGCAACGGGTTTCATTCCCACTGGAGTGCAGTTCGTGAGCGGTCGTTCTCTCCTCTCTCTCGCAAAAGTAAAACTTAAAGTTTTGTGCGCGCATGCGTCTCGTCTTAGAAAAA

>ID_2338 P_2474 Promoter_2533 D3_EP77310 (+) Dm CED-6_2; range -250 to 49. []

TCCGAACACGATTTGCAATTAGCCAAAAGAATACATTCAGATTCAGTTTGTTGTATAACGATAATTAGCAGTACAGCGGCAGCTGGTATTTGTATGCACACTGTATTTTATACTCGAGGGATCCGAGAGGAAAAACCCAACACAATGCCGGCGGCGGGCAAAGTTCAATCCGTAGCTCATTTGCCAATTCTAATTCCAATTTCAGCTCCCTTAAACGCTTAAAAACGGCGCCGACCACAACAATGCCTTATCAGCCAGCCAACAGCGGCGGGACGTCTGGTGGCAGCAAGGCGGCAGCCAAGATGGCCCAGCTAAAGTTCTGGAACAAGCAGAACAGCAGCAAGCAGCAG

>ID_2342 P_2480 Promoter_2539 D3_EP77316 (+) Dm PUG_2; range -250 to 49. []

CTTACCATTCGCACTTCTGCGGTTGTTTTGCTCCCAGAGATTGGAGCATGTTGTGGTACTTAGGTGCCTTGGCATACGTCAGCCGGTGTTGTTCCCGGCTACCAATCGCAAGATGTGACCCGCGAGAAACCCGAAACCGATAATCTCGAAGGCGTCTGATAAGGGAGCGGGATTACAAAAAATAGAGAGAGGCACCGGGCGGAGCACCTATGTGGGCTATAGATAGGGGTGTCCGTTGAGTGCTCAGCTCAGTTGCATAGCGCGAGCCGAGTTGGCGTGGCTAGTGAGGCGGTACTTTCGGTCATCTGGACTAAAAAACTTTACGGGCTTGCGTTTTTTCAGCGGAGAGT

>ID_2343 P_2482 Promoter_2541 D3_EP77318 (+) Dm GST2; range -250 to 49. []

TGGCTAAATATGTTAACTAATTCATTAACTTCGTTTCCTTTTCAACGCCCACTGGAACTAGAATCAGAGAGAGAGAGAGAGAGAGAGAGAGAGAGAGAGAGAGAGAGCCAAACTTAAAATTAAAACACTACCTTTTTCCAGTTAACCGGCCGGCTTACACACACAGTCAAACGCCTGAAGCTTGAAAGAGAGTGTTTGCTTGATGAGAGCGGCAAGCGTATAAAAGCAGCGTCGCCGGCAGAAGAAAGTCAGTTGCAGTGCAGTCGTCGTGACGATCGGTTCAGTGATAGCTTCCCAGTGGCTGGAGAGAGTGAGAGTAGAGAGTAACGGAAAGAGAGTGCGAATCGAAC

>ID_2344 P_2483 Promoter_2542 D3_EP77319 (+) Dm CACT; range -250 to 49. []

TAAGCCCCGATTACACACTCCATCGTCGATATATTAAGGCAAACGTTTTCACTTCGGTCTTTTCAAGTCTAGAATTTTATTTTTTTTGGGCAAATATGAAAAAATAGATTTTTTTCAATTAACAGTTTTCTAAGTAAAGAAAATTTAAAATCGAGACTCTTCATTTCGTGATAACTCTTAGTGCAAACGGGGCTTTATAGTATAGACTGCCATATAAATATATACATATCAGCGCGCTCCCTCAATTTCATTCGCTATCGAAACGTGAAAGAAAGCAGACGTGCGTCCGGGTACTATGCGAATTGCAGATTACAAACATTTAAACCATAAATACGCGTTTTAAAGACAGC

>ID_2368 P_2510 Promoter_2570 D3_EP77347 (+) Dm RAS; range -250 to 49. []

GCTAATTCGCTGCCATATTTTAAAGATTAAATAAAGAGGAAATGGATATTGCTCGAGATTCAGGCTATTCAGGCTAATATGTAATGATTATTGTTTAATATGCTTAATAAATTGCATAGTCGATATTGAAAGCGCCTTAGTGTGGCTGCCATGGAAAGCGCGATTAAGGCCAAAGCGTATCGTGGTAATATTTAGTGGGTTTCCCGGTGGTCCAGCGAGTATATTTAAGCGGCCGCACGGCGGCCACACCACTTCACGAACTCGATTCGCGCAGCACACGCGGCACTTGGACTCCAGCGGACTGGTAAGATGGCCCAACCATGTGCTCGCTGGCTTGCCAGCCAATCGGG

>ID_2371 P_2513 Promoter_2573 D3_EP77350 (+) Dm SP1029; range -250 to 49. []

TTTCTTCCTTTTTTATTGTTTTTTTTACCTCCTTTGCTTGGCTGTTCAACTACATTGCATGCTCTACGATAGAACTAGCATACTGAATCTGCGATTCTCTGCGCCGACGCAAAGCAGCGTTTCAATCTCTAAGCCGTCGACGTTTCACTGGCTCTCCATTTCTCGGCTTTCGGCTTTTCCATCTGTATGGGTGAGCGGTGTAACCACGGGCTATCGTATAAGATAAAAGCGCCGTGCTCAGTAAACTTTAGATTCGCGTCGACCGTCGAACAGGTCGCACTCCTGCGTGTGTTTTTTTCGCTTTTCTGTGGCTGTGACTGAGGCTCTGGCAACGGTTGGTCCCAACAATC

>ID_2406 P_2552 Promoter_2613 D3_EP77390 (+) Dm VHASFD_1; range -250 to 49. []

AATAAATTACCAAACAACTACACATAAAGACCACAAAATCTTTAAAAAATCTTTAAAATGTGTGCCTTATGACGGAAATAAAAAGTATAATGAATAAAGATCCAATTTACTTTTTTATATAACTTAATATTTTTCAAATTTAATATACCACAAGTACAGAAGTCGCGTTGGCAGCTCCAAGCTACCGATAACATGGGGCTCTGCTCGTCATATCGATATTCTTATATATCGAATATTCGATTGAAGGCTGCAGAAAAGTCACAAGACCAGTCAGCTGAATTTCGGTGCTGTGTCGCAGTAGTGTGAAAAACACTCGGTTGTATTTGATAGATTTTCATAAATTCTGTGCT

>ID_2435 P_2594 Promoter_2655 D3_EP77432 (+) Dm CG33153; range -250 to 49. []

GTCGCCGTCGTCTGGCGTTTTGTAGCTTTGCGGCTCTGCCGTCGTATCGGTTGGGAATTGAGAACCGACAATGTCTATATAGATGCTATATAGCCAGACTGTGCCACTGCCACTGCCTCTCGCACGGCCCAAAAGCAGCGACGCCGGCAGCGACGACGACTGCGACGGCAGCGATGTGGTTGAAAAGATTTTTGTATTCGAAAACGGCACAAAATGCACATAAAAATTTTCGGTTTCGGTTTTTGCTTTAGTTGAGAGAAAATCGTCGCTTAGTTAACGTCGCCCAAGAACTGAACTGGCAGTGCACCACAAACGTCACGAATATACGAAACCGAGATCCAAGTCCAAAT

>ID_2445 P_2610 Promoter_2671 D3_EP77449 (+) Dm CG2269; range -250 to 49. []

TTGCAACAGTTATTAGTAGAGACATAATATCATTTTGTATATATTCGAAGAAATTTTTGCATACACTTTAACTATTTCTAAGAATTTTGTTCAGTGTCTTCCCGAACGAAGCTCGGCTGCAGCTAAAAGCTTTCTCTCCGCGACTCCCACGCTCCCCGTCTCGCGAGGACGAGAGGCGCTCGGCGAGCTTCGGGTGCCGTCGGTCCTCGCGGGTCCTGTATAAAGCATGGAAGCTCCAGAGACTGGCGACACTCTTGGTTCCGCCTTGGCTGAGCACAGAGGTGGGTACAGTGGCTACTGGCTGGCAGTGACGAGCGCTTCCTTTGTGTTGCTGGCGTAGGCGTGACGCC

>ID_2461 P_2634 Promoter_2695 D3_EP77476 (+) Dm BNB; range -250 to 49. []

CACGGGATCCTTGCGACTTGCGACTCTCACGTACGCATTCCCGCTGCCCCATCCTCTAAAGATTCTGCCTGCTGCGTGCTGCGTCTTGTTGCGGCTGCTGCTGCTGCTGCTGCTACCGCCGCTGCTGCGACGTTCGTGCCTAGCACGCTGTCTATGGCCATACCGCCGATGCTCTGCCGACGTCGACTTCGGGCGTTCGTGATCCGGCGACGAGTGTATATAAACGCCCCGGCGCGCTGGCATCCACTCAAAGTGTTTCTTGAAACTGACCGGTGAGAACACGCGGCAGGCCAGGCCAGCAGCGCAGTGGATTAAGAATATATATATATATATATATATATATACATATA

>ID_2466 P_2639 Promoter_2700 D3_EP77481 (+) Dm CG3168; range -250 to 49. []

AACAGCAATGGCAATAGCAACCGTAGCAGCTGAGTGTACACAAGCTAAGAACCGAGCCGAGTGCCCCGAGAGTGGCAGGCGCACACACAGATTGTGCGATCGCGAATGAGCGAAGAAGAGTCAGCATCCCGAGAACGATGCCGGAAAGGGGGCGTCTGGGTTTCGGGGTGGGCGGATTTCTGTACGGTCTGTTATGGGGCTGTGGCACTCTGGGTTGGCGCTTATAAAAACGCCGGCCCAACGATACTTGGAACGCATTGTCCAATCGCTCTTGGAGCCGCGTGGAGCTCCTCCAACCAGTGATCGTGATCTATATAGCCCGGATTCTTCAATTCGTCTACTAACCGCCG

>ID_2470 P_2645 Promoter_2706 D3_EP77487 (+) Dm KAP-ALPHA-3; range -250 to 49. []

GTGTAATAGTACCGTCACGACAAGCGCCTGCTTGAAAAAACCTAAATAATAAGAATTGCTATAATGCTTTTTAAGACAAATGAAATATTTCCTAAATAATGTTCAACTGGTTCATAAGCTTACAACTCCAACTGAGTAAACTTAAAATTTCTAAATTTAAAAAATAAGTCGACATAAATTCAGATCTGACGATTGGTGCTTCAATCGACCCTGCCTATAAGTGGGCAGTCCCGAATTGCCAACCGCAGCCAACTTCACTCACGTCGTTGTCACTGATTGCAATTTTAATAAAAAGGAAAGAATTTTATCACTTTTAAAAGACGTAGAAAGTGTATGTGTCGTGGGAGAAA

>ID_2481 P_2659 Promoter_2720 D3_EP77504 (+) Dm PRL-1; range -250 to 49. []

TATGTCGGTTTATAAATAAATATATATGTATATATATTGTACAACTTCCTTTAAAATCACATCTTACATTTAAAATAAAAAAAACTGTTTTTAAAAGACAATTGCAAAATAGGTAATTAAATTATTACAAAAAAGTATCGATTCTGCCCGCAATCGATGTTTATCTAACACTTAATTGCACTCGATACTATCGGCTCTATCGAGTGTTCGAGATTATCGAATATAATCACCATCCGTGTCTACCAACACAAACGAGCGAATTGTTCGCAACGCCTACTTCGATTACAAGTACTTTGCTCTTGGAAATCGGTAGAAAAAATTGTGCAATCCGTGCTGGAAACTACAGATAT

>ID_2489 P_2668 Promoter_2729 D3_EP77513 (+) Dm PDI; range -250 to 49. []

ACATTACTGAATTAGTTAAATATAAAAATAAAACTTCAAATATATAAACGAGATCCTAAACTAAAAAAATTTACCTTCGACGATTGCCAACCTTTGGGCACCCAAGCGCCACCTACAGTTCAATGCTCATTCATTGACAAATATCGATATTAGTAAAATTTATCGGAAACTTGTAGAGAAATACCAAAAATCCCTCACCTAATTCAGCTTTATATGTATATATCTCTGCGTTTCACTGTCACTTGAATTCATTTGAATTTCTACTTGCAACCGGGCAAGTTCAGCGCAGTTCGAATCCCAGTTTTGGTTGGCGAGTGTGCTGCACCCACATAGGGCAATAGATTCAATAA

>ID_2524 P_2715 Promoter_2777 D3_EP77562 (+) Dm PDH; range -250 to 49. []

GCTTTATCTACACAATGACATTATCACTGATGCAAAGCTGACATCGAAAAACCGGAGCGGTGTGAAGCAGACTGAACGCTACAGCGACCAGATGGGGATGGAAATTGATATATGGGCATAAGTTGCATAATCCTAACCCAGAGCAGGTGGAAAACTAGCTATTGATTGCAGTCGAACCAAAGTCACTCAACGATATATCATATAGCCCTTGGGAAATAGGTATAAAAGCGGCGAACATTACAGCTCCAAGTCAGCAGTGCAACTGAGGAGCTCAGTGCTTGATGAAGCCACAAGGAATACCACCGACTACTACACGAATCAGCCAGACCAAGATGTCCTTTCGCGGCAAG

>ID_2526 P_2717 Promoter_2779 D3_EP77564 (+) Dm BCDNA GH08312; range -250 to 49. []

TAGCTTCCTCGGAGGTTTTTATGGCTTTTTGAAAGGCAAAAACAGATCTCTGTTATTAACAGAGTGCGGTTCGAGTCCATGCTCCCCCCTGATTGCTAAGTATACACTGGGTTGGTTTTATGCTAAAAGCATTATCCAAGTCTTGCTCCATAAGTATCGTTAAATAAATTTAACAATAATTCAATCGCTGACTGGTCGCACTATTATATCGCTACGGTATATATTTGACGAAACAGACGGCGGCCACACTACAGACAAATAAAAAATTGAACCAATCGTTGCAATTGCACTTCAGTTTTCGTTCAGAAATAAATACGTAGCTGCAAATAGAGGACTATCTCCGCCGACAT

>ID_2555 P_2757 Promoter_2819 D3_EP77604 (+) Dm CG4710_2; range -250 to 49. []

GCTTGTGTTTTGATCTTTGTTACGTATTTTGCACGGCACTTGGATGATTTTTTTTTTTAATTCGTGCGTGTGCTTGTGTTTTGGCAAGTTCAGTTCGTTAGCTTTGCTTTGTCATGCGATTCGGATTTGAGTTCGGGTCCGAGCAATATCACTCGCTCGTCTCTCGCACTCGCTCTCCGCGCTGACTAACGGCTCTCCGCTCTCGCCAGTCGGATCGGCTATAAAAGCGGTGCGATCTCAGCGAGCGCCGTTCATTTGAGTTTCGCGGTTCGAGGTGCTTTGCTTGCCCACTTGGTCTTTCGGCTCTGGAGAAAACTACTCGCATCGAATTGAATTGAATCTGGGGAAAA

>ID_2557 P_2759 Promoter_2821 D3_EP77606 (+) Dm JHI-21; range -250 to 49. []

AGTTATGCCATAAGATAAATTTAGCATTAGCTAGCATTCAAGAGGACAGCTTTAATGTTTTCTTCTTGTAAGTAACCATTTGACATTAATTTCCGGTTTTGAGCACTCATTTCTCATTTTTTTTCTTTACACTCATTCTCTCTCTCTCTCTGTTTCTCTCTCCGTTTGCTGCTCTCACTCTCTCACTGCGTTTTAGGGTGAAAATTGCGTTAATTTTAGTATGAAAATGCCGGTCTCGATGCCAACGCACAGTCGCTGTTCGCAGTCAAAGCCGTTTTGTTGTGTCCGCGCTCTCGAGGTAAAATAAATCGTGTTTTACGTGCAAAGTCGCTGGGAACAAGATAAACCGA

>ID_2611 P_2823 Promoter_2886 D3_EP77671 (+) Dm CG18143; range -250 to 49. []

ATTTTGAAGTTAATTATTGCTTGTTTTGTAGTTTTTACCGATAAATCACACGTGTGCAAGCGATCGCTTATTAAAACAGTAATAAGTAATAATATTTAAAGTAACGACCAAACGATTGGGAATAATCTTTCCAGCGATTTAAACCTTGAAATAAAAAGTAAGTGTCATCCCCGTATGATTATGCGCACATTCGCAGGGCGACGCGTGCACCTAGGCGAGAGTTTTAAAACTTGTTCAGCGGATCTTGATCATTACATTCTCAAGTCTGTCGTACAGCACGTTTAAGCCCGCTGATAGTAAATCAGAGCAATGGCAACCGTGTTTCTTGGAACTGTCGTGCACACCAAGTC

>ID_2628 P_2849 Promoter_2912 D3_EP77697 (+) Dm DF31; range -250 to 49. []

GCAGCCCTTTTTAGGGGACCGTTATTTTGAAATATTTGTCACTGCTATATTTCATCTCTTTAATATATTAAAATAAAATTGTAAAAATCTATCAGCATAAGCTAACTCCGAATTAAATAGAGAACGGGTGATAACGATAACAAACAAAGATATGTTTTCCGGAGGCGGAACTATCGCAAGCGGACTACCGACGTCTACCAACACTACAGGGCGGCGCTTTAAAAGCCGGTCCGACGCTCACCGCTCTCTCATTCGGCGTTTTTCTTTGTGACTGTGTGGACGTTCTTAATTGTACGGCAAAATCTCCAGCAACCAAGCTACTTTCTTAGCAAAGCTACTAAAGAGGAAAC

>ID_2631 P_2852 Promoter_2915 D3_EP77701 (+) Dm GS2; range -250 to 49. []

CTTTTGTTTTCAATGGCGTGCTGCCCGCTTTCGCTGCCGGCGTCGCAGTCGCAGCTAGTTTGTTGTAACTATAGTATACTGTATGTATGGTACATTATATATATACCGCAACAACAACAGCTACAGTGACAACAATAATAATAACAACAACAACAACAACAGGGGGACGACGCGGGCGCGTTGCTTTTGTTTTGCTTTGTTGTTTATTGTTGGTCATTATAAATTGAGTTGGAACAAGGGGCTCCAGCTTAGTCGTGTTCCAGTAACGCTTCCGCGCACACCAAATCCAATTCAGTACCAAAATCGGAATCAGCGCCAGGACACAGCACACAGCCGTCGTCCCGCCATCA

>ID_2634 P_2856 Promoter_2919 D3_EP77705 (+) Dm Retinin; range -250 to 49. []

GTTCTATGTTTGGATATTAAAATGAATTGTGTGAAAAATTTAAGGAACTATTTTTTGTTTTAAGATATTGCCTGAAATGTTACAATATATTTATTAAATCCAATAAAAAAAAATTTACAATACATTTAAGCGCTTTTACAAATCATCGTTGCTCTAAATTAACTGAATTTGTAATCGATAGCCCGATACGTGCTGCTCCATCCCTCATTACCCCAACTCTTCTAGAAAAACACGTCAGCTTTTCACAGCCATTCGGAAAAGAACACGTCAGCAGAAAGGAAAACTGGTCGGTCGATATTGCAAAGGTGGATGAGAAAATTGCGTGAGACGGAGCCAGAAAAGCCCGAGTC

>ID_2637 P_2861 Promoter_2924 D3_EP77710 (+) Dm IM2; range -250 to 49. []

TTTTCTCTCGAACATTAAAGCAATCTTAGCTTCTACCTGTTGGTTCACATAACACGCCCATAAACACGGGACTGGAATTTCCAGATTTAAATACGCAAAACAAATGCTATTGAACGCTCAACTTTCTGTGTTTTCCCAAGCGCTTTCGATTCGATGGGGGTTATCAGTGGCCTCCGTCCAACCAATGCACATTTGCTGCTCCAAGATTCCAGGCTGAGCTATATAAGATCCAATCGAACTAGCCAAGAGCATCAGTTGAATTCAATCGATTGCTTGTGCATTTAGCAAATCAAAGCCACAACAACCAAACCAGAATCAATATGAAGTTCTTCTCAGTCGTCACCGTCTTT

>ID_2640 P_2864 Promoter_2927 D3_EP77713 (+) Dm ND75; range -250 to 49. []

ACTACAGTAACACTTGAACTTGAAGGTGGTAATCGAAGCAGGTAACAATATCAAATATATACTTTTTGAATAGAAAACTAATTTCGGAGATTTATAAATTGGTGTCGCACATAAATTTATATTTAAGGAATAAATTTAAAAACCGATAAACTATTGCGATTGACCAGCTGTCAACGCGGCAGTTGGCAACGCGGTCGGTCTGTCAAAGCAAAATCCGATAAAAAAAAAGTTGTCTCCACATTAAAAGATCACAGGAATATTTTCCAGCATTTCGGTAGGTCTTCGCCCACTTTTGGCATCCTCCAGGATCCCCATTCAGGTGGCAAATGGCGGTGCAGTGGAGGAGCTGA

>ID_2651 P_2880 Promoter_2943 D3_EP77729 (+) Dm CG4523; range -250 to 49. []

AAAAAGGCGATATATCTTTGGATAATCATTGCAAAAATAAACCCTTTCGATTCGACGATTTCGCCTTGTAGTTCTGCCACCACCGCCCCCACACTTCGCCCCGCAGCTGCACCACAACCACCACCACCCACAAACAGAAAATTTATTTGCGACGCAAAAACAAAACAATCGAATCGAAACGAACCGAACCGAAACGAAACGACAGCAGCTGTTTGGTTATAAATCGGCGCTATCGATCGCACACATGAGCAAAATTGGCGGAGAAGAAGAGGAAGCAGCAGCAAGAGTTAAGCACCACAAATCTTAAAGAATAGGTAAAAATTAAAAATTAAATTAATAGATGACAGACC

>ID_2654 P_2883 Promoter_2946 D3_EP77732 (+) Dm AMR1; range -250 to 49. []

CGACGATGCACAGCGACAGGATAAACAGGATGACATGGCCAGCTATCTGGCTTAGCTGCAGCACGTTGGCCAAAGCCATTTTGCTTTTGATTTCAACAAAAACAAATGAAACTCCACAATATTCAGACAAACACGGCAATGCACTTTTGGTGTTATCGATAAGCAGTGTTGGACAGCACCCTGCAGCTGCCTGCATTGGTATGCGCAATTATCGATATATACACCCTGGGGCCATCATTCTCGTTAAGCCATCTCTAGTTCGCCACTGAACTCGTAAAAAAGTGTAAAATTTGTTTACATTGAAAAAAGGTAAAATATTGTTCTTGAGGGCTACCTACGGTGCTCCCTGG

>ID_2666 P_2899 Promoter_2962 D3_EP77748 (+) Dm CG6084; range -250 to 49. []

TAACATATGTAAAATGATACATTTTAGTATCATTTATTGAAATCATTAAATGTTTTAATTGATTTAGTTTAATATATATTTTCTTTTATGCTATATAGAGAGTATATCAGCACTCGATATTTAAGCGTAACTGACGATCGCTCAGCTGTTCGGCTTGTCGACTATGCAGCCCTGTCCTCCGAAAGCTTTTTAGAATTTCGAACAAGCTTTTGCGGTGCGTATATAAGCGGTTAACAGCTGAGTGGAATCGCTCAGTTTGTTGAGAGGCGGCAATCGTTAAAAGATTCCCAATTGTATTATAAATATTTATAAATATCCTTAAACAATAAGCGAAAATATAGTACAATGGC

>ID_2668 P_2902 Promoter_2965 D3_EP77751 (+) Dm CG14207; range -250 to 49. []

ATCAATACAGCGAATATAGTGTTTTGGGTGGTCCGCTAACGATTAGTGGTTAAAGTACGCTTAAACAATTCCCTTTGCGGATAGAAGTTCTTTTTGATCCAATGGCAATAGAAACATAATCAAATCCGCAAATCGATGACTAAATGCGGCTTCATCGGTCTATCGATATCGCCACACTGCTGCGCAGCGGCAATCGAAGTACAATCGGTACGAAAGCTATAAAAGCAAAGACACACTGTGAATGCAAAACAGTTCAGAAATTGAAACGCGGCTATCTGTGCGATACGTCCAACCGAAATCACACACTTGAACGTCTTGAGTTTCGAATACATATAGAAAACCCATCGAGT

>ID_2691 P_2938 Promoter_3003 D3_EP77789 (+) Dm CG5171; range -250 to 49. []

TGGAATTTATGAATTTTTGGTTGCTGTTTTATTGCCCTCCAATGTGAACTTTACGACCCGGGGAGAAGGAGGCAATGCACGCACCGTACACGAAGGCAACGCATGATGCACTGAATCGGCGAGTCGTTGAATCGGCGGGCGGTGGGTGGCGTACTGCCTTTGTTCAAAAGCACAAAAACCCGGCGACAATTTGTTTGAGGAAATCACCGCCGACGGATCATTATAAAACTGCAGCTGCCGGAGAAGTAGCAAGTCAAACCGCCTTGCAGATCCATCAGGGATAGGTGTCGATAGCCGCTCGAGAAGTTCCAAGTACCCGGCTACCTTAAGGTGACTTGATCTTTTGAGCT

>ID_2692 P_2939 Promoter_3004 D3_EP77790 (+) Dm BCDNA GH05095; range -250 to 49. []

AAACCGCAAAGACACATGCTCAGATACCGTCTCGTGTCCACTCACTTCCTCAAGCAGCCGTAGTGTGCGAGCGAGCGAGACAGCCACACCCGTAGAGGGGGGACCGAGAACGAGCACGAGAGCGAGAGAGTGTGGGTGTAACTGCGAATGCACTTGCAACTGCAATTGAGAGAGAGAGAGTGACAGCGAGAGCGAGCGATCGCAGGATACGATTGTGCTATAAAAGTGCGACAGGTGCGCTCTACCTGCCCATTCACCGTTAGTCGTCGTCGGTAACTCATTTAGTTGTCGAGAGTTCCTCTTTACATCCCAGAAAGAGGCAGGATCAAGGAGCGTACTAAAGAAAAGGG

>ID_2715 P_2969 Promoter_3034 D3_EP77820 (+) Dm TRX-2; range -250 to 49. []

CAGACATAACCAGACTAAAGAGAATAGTACTACAAGTTATAATCGCTATCTTCAGCAAGAGGATTTCACATTTAAAGTAATCGCATAGTGGCCAGCAAAACCTATCGCATAGATGTGCCAATCGGTCTATCCATTCGAAATTCACCTTCACCAAACGCGACATACACATATGCAAAAAGAGAGCGTATAGCAATGAGAAGCGTGAGCGTGAGTAAAAAATCTATAAAAGCAACTGCGACGTGCTCATTTTGGTAAAAAATTTAGCTGTGCTGCAAAGAGCTGCCCGAGTGGTAATTAAGTAACTTTTGTACATTTCTACCGGTTCCGTCTCCACATCTCCCATCCAACAT

>ID_2716 P_2971 Promoter_3036 D3_EP77822 (+) Dm CG3107; range -250 to 49. []

GGGATTGCTTAAGAGATAATGATTGTGTGAGTTAGTTAATTTCTGGATATTAATAATATTGAATACTCATTTAGCGCAGACCGCGCTTTTAAATGAAATTGTTAGCATTATATTTTTCATTCAAAGCATTTCATAATTTGTATGAAAACGTCCTAGTGTACCATTTTCCACCGTTCCCCCATCGCAGCTAAGTCATCGACGATAGCTTGACTCAATTATAAGTCGAATAAAATGTAATAATTTAGATTTTAATTGTCTTTTTTTTCTATACGCCGGTGAGTAAAATTATTTAGGTCCATTTCTGTTTGAATCAAAAATTAATGCCAATAAGGCATTGTTATGCCCGAAAC

>ID_2730 P_2992 Promoter_3057 D3_EP77843 (+) Dm CG31626; range -250 to 49. []

ATGTTAATACGTAAGATCAAAGGGAAAAATAAAATAAATATATCTTTGTTTCGTTTTGATCGCCCAACAGCCGCCTCTGGGTTAATGATCACTGCTCCGCTCTCTTCTTAACTGTCTACCTTTTCTTCGACTAGAAGAAGCGCCAGGCCCACACCGAAGACGCGAAGACGGATATCCAGCAATAATAATGGGCCAGACCAAGTTAAGCATGGCTTTTGGTATAAAAGTGCGGGCCCATCTGCAGTTCGACATCATTCAGCTTGAGCGTCTTGCGACAAGAGCTTGCCAAAGCAAATCCAACGAACTTTAAAAGCCCGACCAACAAATCAATAACAATGGTGAGTAAACTG

>ID_2763 P_3037 Promoter_3104 D3_EP77891 (+) Dm CG6330; range -250 to 49. []

AGGCTCGTGGCCAATTGCACTTGATTAGTGATGCATAAGTCGCTCTGAACTGATAACAATCTGTATCTATGTATGTATGTATGCCATTCTATAGAGGTTTCTATTACACGACCATACAGTGTGCAATATATTTTCATTTCCACTATCAAATTGCGCAGCATAACTTCCGATTCGCCGAGAAGATTGTCTTATCTACACAGCTCAGCAGAAGCGGCCTATATAAAGGCCGGCGAAACGCAAGAAATTTTTTCAGTCTCGCTCGAAGCGTTGACGTGTCTACACGAGTAACTAGTTTTGTTTTGGCTCGCTGGCTGCTCCTTCGTCTGGTAGTTGGTAGTTCGTTTGTGAGT

>ID_2765 P_3039 Promoter_3106 D3_EP77893 (+) Dm BEST CK01140; range -250 to 49. []

AAACTTATATGTAGGCATAACCGATTTTCTAACATTTTAACTAAAAAGAAAAGTGAAGAGGTATTACGACGTAAGAGAGATATGCTACACGAACATTTGTGCTAGCGCGGCGTAATAAAATAATTTATTTTTACACTACATGTGAAAATACATCGATATTAGAATATTACAGTTTTGTGAAAACTAAGTGATTTCGATAGTAAAAGTTTGATATATCGATATATATTTCCCTGTGGAAAAATAAATACTGCTGAAAAGGAAGGCGTCACAGTGTCAGTACTTTATATTAAATATTTCGCTATATATTGTGGCGTTTAACTTGAGTCGTGTACAATTATTTCGCCTTCTAG

>ID_2769 P_3046 Promoter_3113 D3_EP77900 (+) Dm CG17108; range -250 to 49. []

AAATCAAACTTCTTTGTAACAAATATTGTACTGAAATGGATTACTAATCCATTTGATTATTCTTTGTTGTCCAGTGCAGTCTGAGTTTTTGCCTGGCCTGACCCTAGGATCCCAGCATTGGGAACGTGACTGAGTTCTGTTCTTTTTTCGCGCTCTGCGAAATTTTTGCAAGTATTTTGCTATTATTTGTGCTTTTTGCACATCGGAGAGTCTCTTATAAAAGCCGGCATCTTTCCAGGGAGCCCAGTTAGTCAGCTGCAGGCAAAGGTTCCGATGCGTCTGTTTATAGTGCTGAGTGTTTGTGTGGCGTTAGCTTCCGCCGGAGGTTACGGCGGTGGAGGCGGAGGTGG

>ID_2774 P_3051 Promoter_3118 D3_EP77905 (+) Dm INOS; range -250 to 49. []

CTCAATGAATTATGGAAATAGGTGTACTACGATCTCCGGCTGGAAAGAGGCGGACAGAAGGACTCCGCGATGCTCGCTAAGAAATCCCACGCTTGCCGGAAAATTTCCGATATCATCTGGTTCCTATAAGTGCGCCGCAGAGCGTTGTGGAGAGTATATGCACATATCGTACATGAGTCGCGGAATAAGAGAGTGCGCCTCTGCCCGCGGGAGACCATAAAAGCGGCCCGATTTCCACTCAGCCGAGTCATTCGCCGCGAACTTTTGCCTCGACCACATCGCAGTCTCTTTTGCAGAAGCCAGGAATTCCAGAAGCAAGCACATTGAAACGCGCTAATTGACAAGACGAG

>ID_2811 P_3094 Promoter_3161 D3_EP77948 (+) Dm CDC25; range -250 to 49. []

CCCGTCCCCGTTATGTGTGTTGGTGTCGCTCGTTGCCCGTCTCCCTTCCTCTTCTATTGGCACTTCTGGGAAGGATAGGAAGGAAAGGATAGGATGGGAAGGCAAAGGAAAGGAGCTGAGAACTAAGAGCTAAAGAGCGAAGCAGAGAGAACAAAAAACGCGCGCCATCTCGCTCGCGCCCATTAGCTCATCAGCTGATCGTGATCGGCCGGGGTGCGGTTATAAAAACCAGCTGCTCGGCATATTCAGCACATTCGTTCTCAGTTCGTCGTCGAGTCAACAGCTCTTCTTGTTCAGCCTTTCGGTTCCTCGTTCGGTGAATTGAGATTGTGCGGTTCAAATTATTGAAG

>ID_2814 P_3100 Promoter_3167 D3_EP77954 (+) Dm CG9686; range -250 to 49. []

ATCGTAGCGATACTAGGATATACATTTAAAAGGGCAACATTATCTGAATGACCATCACGGAATCATCGGCCTGCAGCTGTATCTCCATCCATATCATTGTGTATTTGTATCTTTGTCGAGTTGTGCTTAAATCAGCGCACAATTGCGATGATTGTTGCCGTTCGCCAATTGAATTAAAGAAAATGCGAAGCAAGTGACACACAAAGGCGACGGCGATACGAATAAAATGGGTGGAAATCGAAGCGCCCCAGGTCAGTCCAGTATCAGTGGAGTCAACGTTCAGTCTGGTTTTTGTGGATAAAGCCAAAGGAACCGAATATATCAAGATGAATAGCTTGCAAGGATCGCTG

>ID_2833 P_3131 Promoter_3198 D3_EP77987 (+) Dm CRC_1; range -250 to 49. []

TAATTTAATTTAATTAGCAAACGGTCACTTTAGTGTTTCTAATTCCAGCTCAAGCAATCGATAGCGACAGGGCAGCATGCGATTGCTTATCGAAATCAGCCCTGGCCGGTGAAAAAGAATACGTAGACATCCCTGATTGGCCTGATTTGGGCCAATGAAAACGTTCCAGCAAACCAATTTCAGTAACAAGCCGTCCCGCTCCGACACGCACGATTTAGATGCAATAAATAAATAGCACTTTCTTGCCGGCATTTAGTACTTAACTTTAACCGAGTTCGGACAACCATCGGAGTTGGAAGAGTCAGTCCAAAAAGTGGTGCGGGATCGTTCACATGATGTGGTGCAAAACA

>ID_2847 P_3147 Promoter_3214 D3_EP78003 (+) Dm ACER; range -250 to 49. []

ATATCAGCTGACCACATTTGAGTTTGCTGTACAAAATACCGTTAGCGGTGCACTCAACAGCTGAGCGGCGCTCAAGCTAACCCGCCGATAATGCAGACAACAAAAAAAGTATTAAGCAGGTAGATTGGCAGGCAGGTGTAAAATTTCGGAACACGCACACTCTTGTGATTGTGTATGAATGCGAAACGGCGTACGCTCTCGCAAACGCTCCTACGAGTATAAAAACCGTTCTCCGTCGCAGCAAGCAGTCAGTATCTCCACAGCAGCAGAGGCGACGGGAAGACAAAGCAATCTGAATTGAATACGTCCTAGTTTTGGGTCCGTATAACAAAAAAATTTATAGTGCAAAG

>ID_2879 P_3189 Promoter_3257 D3_EP78046 (+) Dm CG8871; range -250 to 49. []

ATACTTAATATATGGTAAATAATAAAATAATATAATATATATAAATATATATAGTCTAGTGGGGGGGGTCTAGTCCCCCCTAGAGTTTCTTTGATTTCTACTACTGGCTTGGTAGAGTGGTTCCTCCATTCTTATCGATGACATCGTTTATGGGAGTCTTGAGGATTATTTACGAGCCCTTTTCGAGTTGCAGATATGGTTTTAGCTGAAAATGTGTATAAAAGGCTGCCATCCGAGAGTAGAATCCAGTATAAACATCAGCATGAAGGTGTTCCTAGCTATCCTGGCTCTGGCCGTGGCTTCGGCCTCCGCCTTCGACGAGAAGGTCTTCGTAAAGGACCTGCCCAAGG

>ID_2973 P_3324 Promoter_3393 D3_EP78184 (+) Dm CG5447; range -250 to 49. []

AATTATGGAAGAAGAAATAAAGATTTAAGGATATAATGGGAACTTTTAATATTATCCTTTCAAACTTAAAATTGTGTCCAGAAATCCGATTTGAATTATGGCGCCAAAGATATAATAATTTTCTGCCTTAGTGGCAACACTGCACATCCATAGTATTTTTTAGTACATGCAGTATTTTTAGAATATTTATGGAACGATAGGGATGGGGTAACACTATTTTGGTATAAAAGCCAACTCGCCATCTGGTAACACTTGGAGTTTGCAAACATTTATCTCTCGACCAATTTAAGGAATTTGAGGCAATTAGGGCATTCCTGTGAGCTTTTCCAGGCCAACTGCGATTACTTCAA

>ID_2984 P_3341 Promoter_3410 D3_EP78201 (+) Dm RK2; range -250 to 49. []

CCGCGATCCCATCGGTTATCTGCAGAAGTTTTGGCTTGACGACAAGCACAAGTGTGACATTCCATTGCCGCAGAATATTCTTTAACATATAGCTCTGATGTAACCCAATCAAATTTCAAATTGTCAGCTAATTAAATGTTTTTGATTCGACGTTAAGCTTACGCAATGATGCATTTCTTTGATCAGTCAGGCGAAGAGATTCTGTACCGATTTAAGGGCTATAAAAGCAGATATTCCAATCTCAAACTGCATCATTCATTTCACAGTTTGCGGATCTAAAGGAAGACTGCTATAATCATGTCTAGATTGGTGAATTATTTCTTACTTTCATTGAGGGAAAAATTTATTCA

>ID_2990 P_3350 Promoter_3419 D3_EP78213 (+) Dm EF1-BETA; range -250 to 49. []

AATGTAATTGTTTGGCCTTAAATTTACGTAGCCAATAGCTAGCTTAAGTATAAGGTATTAATTATAATCCTTATGTTTTATTAAAAAAAATACAAGTAAGCGTTAAAAATCTATTTGAATGTATGGTATATTTGAACGATATTTACTCAAACTCCGGGTCACCCTGTGTTGCAACTACTTGAAATACTAGGAAAAAGTCAGTATTTTCATTATATGTTTAAAGACCGTTTCCCGCGAAGGTCACTCTGTTCTTCTTTCGTCGGCAACGTTGCGCAGAGCACGGCTCCAGATTTGTTAAACGCGAATTTACCCCACAAAAACGCATTTAAAATGGCATTCGGTGATGTGAC

>ID_2993 P_3354 Promoter_3423 D3_EP78217 (+) Dm GP93; range -250 to 49. []

TTTAACAGTAGAGTTTTGACACTACATCCTCTTGTAAACTAAATCCCAAATAGATTCTATGAATGTATGGGCTTTCAAATAAGCAAACATTAAATTTATTTAAATTACACTATTTTCGTGCTCTGTGCTGTATATATATCCTATTTCCAGTGCAGCCCCTGGTATTTCCTTCCAATGCGCTTAGAATGTTCCAGCGCCGGAATACGGTCACTCTGCGAGGCATTTAAAGGCCTTCCAGCCGCTTGTCGGCGTCAGTAAAAAAAAATCAAAGCAGGCGACGCAAGTGCGGACTAATTTATTGCATCTGTTATTCGTGACGTGGCCTTTCGCCGGACGAACCGATAGCTCGA

>ID_2996 P_3357 Promoter_3426 D3_EP78221 (+) Dm PEBIII; range -250 to 49. []

TTAAGTGTGTATAACTATCGCACATATATTATATAAATTTATAACGACGATTGACTAATCACTGGGTTGACTTTTCAGCAATAGCGATTTTCGCTTCAAAGTCTAAAGTGTGTGCAATCCCGTATGGATACGAGCCGAGCTCGAACACAGGCTCACAGCGCGCGGTTTGTGTTGTGTATTACACTTGAAGTTCAATCCCAGGTAAAAAGTGGGCCCAGGTATAAAAGCCCACTTCATAGAAACATTTCTCAGTTAGTTCCAGCAGCTGAATTGCTTCACATCTATTCGCTCCGAGTCGCCCCACAAGTCGAGCCCCATTGTAAGACCACCGCACCGTTTATAGTTTTTGG

>ID_2998 P_3360 Promoter_3429 D3_EP78224 (+) Dm CG13868; range -250 to 49. []

GTTGCAGTGTTCCCGTCCCAAACAATAACATCCGATTTGTTTACCTACGAAGCGTTGGAGCAAAACAAAAATCGCGTTCAAGTTTGGCTCCCCTTATCGTTTTTTTTTTTTGTTTGTGTGGAATTTTTCAAGCTTTTTTGCGTCATAAATTGAAATATTTTCGAGTTCACGCCAATCGCAGTCGACGTCGGCAGAGCGGCGAGAAAGGCAGAGATCGTTTATATAGCGCAATACTCGCAGCCGATTTTCATCAGTCGCAACGCGTCTCTCGTTCTCAGCGGAGCTCCTGAGCGGAAAAACCAAGGGAAAATCGGTAATCGGAATATTCGAAAATAGCGCAGTGCAAGTGC

>ID_3001 P_3364 Promoter_3433 D3_EP78229 (+) Dm CG10664; range -250 to 49. []

AATATTTCACCATGCTGCGAAATAAAATAAAACATAATAGTTGCCCTTATTTGCTGTTTCTTTATTTTAATATATTTAGATTTAAATACCCCGCCGAATTCCGATTGCTAGCGATTGAATATATCGATATATTAATATTTATATTTTTTCATTCGAGATGTGGTCACACTTCTCTGCATGAAAATACCAAAATATCGAGCAATAATTACCCATCCATATATATATTTTGTTTTGTATATTCCCTCTGGTCACATTGTTCAGCAAGTCTAAGAAAAGTAGGTAAAAGTGCTTTAAAAATTGTATTAATTGCGAAAAATATCAAGTACCTGCGACCACAAAGCTATTGAATA

>ID_3003 P_3366 Promoter_3435 D3_EP78231 (+) Dm CG11700; range -250 to 49. []

ATGCACTACGCATTTTGTTTTCAATGCCCAATTTTGCGTATTGCAGCTAGCCAATTGATTGGCCTTCATGTGTTAAATACTCTTGTCAATGCGAAATCTCATTTCCAATAGACTAGACAAAAGAAACGGTCACTTTAAGTGGCAGAGAGCGAAAACAAAGTAACCTACCGCCCGAGAGTGAGCGAGAGAGCAGGCGACGACTTTCCAGTCAGCTGCCTATAAATACCAGCGTGGGGCTGCGAAACGAATCATAAACTGTGAAAAAGTCGTAGAGAGCGGACGTCCGAGCAAGTAAAATAAAAGCAAGTTTTTGAATATTTCAAAAAGTGCAGACCAACAGTAAAGAAATA

>ID_3009 P_3373 Promoter_3442 D3_EP78239 (+) Dm CYP4E3; range -250 to 49. []

ATGTCTTTGGTATTGCTTCTAATTATAAATTTCACTTTTATTTAACGTTTTGTTTAAATATTCAAATTTAAATTTAAATCAAAATAGCTGTTACCTATCGATATATCGAAACTGTTATTGCTCACTTTTGCGTTTAATTTAGCAAAGTTCTGTACCATTTGCAGCTAAGTTGCACACCTTCGGTTTAAATTAGGTTTATTTTCAATACCACCTGGTCTTTTAAATAACAATTTGGAACTGGTCACACTTCCTTTCCACGCGAAACCAACAACACAAAAAACGTGTGTTTTGGTCAACAGCGTGTTTTCTTTAAAATAAATCTGTATAGAACAGATTTAACTTGACAAGAA

>ID_3013 P_3378 Promoter_3447 D3_EP78244 (+) Dm CABP1_2; range -250 to 49. []

ACCATTTTCCTTTCGTTATTTGTACGTATATATAATTAGAATTACAAATAAAATCACGCCTTTGTTTTTGTAAAAGTATATAAGATTTGGCTTATTTCACATTATCAGCAGTATCCGCATTTGCCGGCTTCTACATCGATAGTCACAAAGTATTGAAACGCCTATCGAACTATCGAGAGTACGCCGCACTCGCAGTGACGTGTACTCTTTTTTTTTAGCAAAATATAAGCCGCATCTCTACACATAGTTTAGTTTAAAAGAGCCTATCGACAAGACGCGTTGATTAAGAATTTGTTGAGATAAACTCGCAAGAGATAGACACGTAAGCGGTTTCGCAAACTCAAAAAAGA

>ID_3016 P_3381 Promoter_3450 D3_EP78247 (+) Dm VHAAC39; range -250 to 49. []

ATATAAGTCCACTGCACTTAGTATATCAATAAAATATAAGTCTACTGCACTTAGTATATCATTAAAATATAAAATGTAGGTTTATATTAGTATAACTGTAACGAATATTCAAGCTCGCGCGGAGTTGCCAGATCACAACTTAACAGCGTGCTGTTATGCGCATGTTGTCACCTGACATTCCGCCTTTTTTAGTACCTTTTGCGCTGACATTTGGTCGAGAAAAATATAAGAAGAGGAAGAGTTGAAAATCATATGACTTGTCGATCGATAGAGAAAAAAGGTTGAATTTTCACCGTCGCTCCGTATCGAACTGCACGCCCTTCGCCACACAATTCGCGCAACTTTCGGTC

>ID_3027 P_3400 Promoter_3469 D3_EP78266 (+) Dm CG9953_1; range -250 to 49. []

AAGAATGTATACTTTTCTTAATTACTTGACAAAAATTTTGCAAAACACGTTCACATTTACGGTGCTATTAAGCTCATCGATAATATCTAGCTAGGAAATAATATAAATAAATAAAAAGCTAAATTAAGTTTTGTAAAGTTACCAGAACACTTACAGCATACAGAAATCTTTCTCTGCTTAAAATTAAATTAATTCAATTAATGATTGAAATCAATAACTATGAATGCCCCTACTGTTTTAGTTTTGCTTTGCCGGCTTTGCGAATATAGATAATACAGCGAGAACTGAATAATCAGGAGAGTGCGACACACATAACGGTGCCTATATAAATAGAACACACCACTCTCCTT

>ID_3028 P_3401 Promoter_3470 D3_EP78267 (+) Dm CG9953_2; range -250 to 49. []

AAATAAAAAGCTAAATTAAGTTTTGTAAAGTTACCAGAACACTTACAGCATACAGAAATCTTTCTCTGCTTAAAATTAAATTAATTCAATTAATGATTGAAATCAATAACTATGAATGCCCCTACTGTTTTAGTTTTGCTTTGCCGGCTTTGCGAATATAGATAATACAGCGAGAACTGAATAATCAGGAGAGTGCGACACACATAACGGTGCCTATATAAATAGAACACACCACTCTCCTTCAGTCGCTATTTACAGTTAGGCACTGTGCGAGGTTCCAACTAATTAAATACTTCAATTACTGGCAAATTCTACTTGACCCGAACAAACAAAGGAAGCAAATATGCTTT

>ID_3030 P_3403 Promoter_3472 D3_EP78269 (+) Dm LSD2; range -250 to 49. []

AATACAAAGTGTATTATAGCCACATGACCCTTGACTAGGCATAAATCTTGGAAAAAACACACTGCGGTTTGAACAAAAAGTAAGCTCTATTGGTAAAAACTGTACTTTTTAGCTATTTGACTAAAATTTCCTCATCCACTTCTTGCCACCTGCGGCATCACTGTTGCTCATAGCTTTTTGTTGCTCGGCGCTGGCAACACTGGCGGCGGCACTCGCTCATATAATAAACCAGCCGCGCAGCTCGCAAATCAGTCAGAGAGTCCCAACGAGCAGACGTGAGCAGACGTGCCAAAAAGTGCTAGGAATAAAATAAAACAGAGTGCTTCCAGAACGCAGACACACGCATACCC

>ID_3031 P_3405 Promoter_3474 D3_EP78271 (+) Dm CG7738; range -250 to 49. []

AATATTTGTGTGGTGAAATATATAGCGATACAAACCAGAACCCGGTTAAAAAAGCCCCTAAACAATCGCACCTAGAGGCCAAGTAGGTATATAGTACATACACAGTATAGTACGACTATGGCAAGAGGTGGTGGTGGAGCTATTGGAGGGGGGTGCTGTGGAGAGACCCGACTCATCGATGCGTATGAGAACAGGAACGTCAAGTGGCGAGATCTTCGCATATAAGGCCCGCGATTGAGACGAACAAATCATCAGTTGCACGAGTCTGTCGAGTCTGTATCAGCAACCCATTGAATCAAGCAGAAAGCAAGATGCGTCTAACACTTCTGGCCCTCATCGGTGTCCTGTGC

>ID_3043 P_3422 Promoter_3491 D3_EP78288 (+) Dm CG11107; range -250 to 49. []

TTTCCAATCAAATTGCTGTCAAATGTCAATTTTAAGACTGTGAGCTCACAACTATTCTTCAAACTGGTGAGTAAAACCGAATTCTATAGATAGATTCTCGTCAAAAGACTTAAGCTTGAACTGCTGTTTTTTTTTAAATGCCAAATTGCTGCCGTGCTGTTGGTTGTGATCTTCGCTAACCATGGGCTTATATAGTATACACAGACAGTAAACTTTGGTATTTTAAAAGTTGCGATCAGCATGGTCGGTATATGCTAAAGGTCTCACTGAGCGCGTGTTTTTTTTTGTGCTAAACGCTTCGTTTTGAGAATTAAGTGCAGTAATCACCATAAATTAGCATGTCCAAGCGT

>ID_3050 P_3434 Promoter_3503 D3_EP78300 (+) Dm CG2765; range -250 to 49. []

TTTGGTGCAAAACTAAAAAAAAATAAAACAATGTTGAATATTTTGCCACTTATCCTAATTTATTTTTAAATTTAAGCATTTTCGAATTAGCTAATTAATTAACGGCGTTGGCCGGTGCTGCCACATTGAACATGACAGCTGATGTTTACATCGATTGCCCCGAGCTGAGTAATCGATAGTTCTATCGGAGCCTCCGCAGCCCTGACTCGGCCCGTACTATAAAAGCAACTGGCAATGCTTCCCTCTCTGTAATTTTTTCAAAATTCTTCTCGCTGACTGCTCGTGTCGAAACAGAAACTGAAATTAAATTAAACTCGGTGCGTGTGCAGACTACTTAGAATTCCCATATT

>ID_3062 P_3449 Promoter_3518 D3_EP78315 (+) Dm DNAJ-1; range -250 to 49. []

TCGTAAAAGCATAAGAAGCATATAAAGAAGAGTCACCGCCAAAAGCATGCACAAATATATATAAATGGGGAGCGATTTAAAAACAGTGCACTGTGTTTAAAACATCGACAGCTATCGGTTAGCATATCGATATTGACATTCGCAGTCAAACGTTTTCGAGATACAACCCTAAAATCCGAGAAGCATCCAGAAATTTCGACGTAGACGAGGGCGAACCTATAAAATGAGGTTGACGCACGAATCCCGCTCATTATTAAACAAAATTTTGAAGAGAAAGAAACTCTGAAGTAGGGTGTGTTTTTAGTGCGCAAGCCACATTTGGTGGATAAAGAAAAAAGCGAAGAAGAACA

>ID_3073 P_3461 Promoter_3530 D3_EP78327 (+) Dm CALPB; range -250 to 49. []

TATTTGTTTTTATTGAAACTGTTTAATAAAACAAAATTAATATTTCTATAATATATTTTCTGAGTTAATAAATGAATTAGAATGTTATAAATATTTCATTCTGCATTTTCAAAGCTTTTTTGATTGCACATGCATATTCAAAGAAAATTTATACAGAACGCGCAAGGGTTAAAGATTATGAATCATTTACCGAGAAAGTTAAATTTGGATACTTCTCCCATTTAAAGCGTACGATTCTCAGCGAGTCAGCAGTTTTACCACGGCAGCGAGTTAATAAACATCTGGATATCATCCAGTTCCGTAGTTTTAGCCGATTTGCAACGCCATTAGTAGTTAGATTTAGGGAAAAA

>ID_3091 P_3482 Promoter_3551 D3_EP78348 (+) Dm HOP; range -250 to 49. []

ATAAACAATACAATAAAATAACATTTAAATTCTATAAAGCTCAAGGTATTATTTATATTTGATCAACAATTTTTTTAATTTATATAAGCAATGGAAAAAATTATTTAAGAAGGCTTGCTGGTTTTGTTCCCCGCTTGAGAACGAGCTAAGTTCTAAACCGCAGTCTGGAAAAAGATTAAATTCAGTCGCAGAAGCATTGACAACCCTATCGATTGCTTCTAGAAAAGTCGGCATTTTTCGCATTTTCACCTGGCACGGCACATTTCGCAGCGTGTTTTATTAAAAAGTCGTACAAAAATACAGGAAAAACTGTAATTCGACAGCGCTATCCTCTCCCTTATCAGAAATGG

>ID_3106 P_3502 Promoter_3571 D3_EP78368 (+) Dm CG13096; range -250 to 49. []

TTTTATCTTCTAAGGGAGAAATAGAGATGGGATACAAATCGAAATCGATATGCTATAACGGTCAATTTTCCAATCGGTTTTGGTGAAACAGCTGCTATTGGTGCTCGGCCCTGCGAAGGGGGTGTGTATTGGCTAAAGTTGTGACCGTGCCTGAGACATCGGTAATGAAAATAGTTCCCGATATCGACTGTAGCTACGTTTTGCGATTCTGAACATATCGATATTAAAGCGTAGCACGTGTGGTTGTGGTGCATTTTAAATATAGTTTTGCTCGAGTTTTTACGCATCTGTCAAAAAAGCAAAAAATAATATGGTTAAAGTACAGAAACCGCAGCCCAAGTCGCTGAACA

>ID_3119 P_3517 Promoter_3587 D3_EP78384 (+) Dm CG31233; range -250 to 49. []

CAGGGCGTCTATCTTGAAATGCCCACACTTATTCGACATTATCTTAGACGTGATTGTTACTAACCCTGCAGAAAATACAAATTCATCAAAGGCTAGTACCATACTTGATTTACTACCCAATGGAGTTTATACCACAGTTCTTGGCTGGTAACGGTCATTCTGGGTAACAAGATTGGGGTAATCTTTATGTCTGCTCCCAGATAAGATGGACTGCCTCGCTTAAAAGTGGCTCCCCGAACTTTGGCAGTTTAGTCTTTCCGCGTTTTTCGCCATGAGTTGCGTTCAGGTCGGGGGCATGTCAGTATTCTTGGTCCTTTTGCTGGCCATTTCCGCCACCCAGGCGGCTGTGG

>ID_3129 P_3531 Promoter_3601 D3_EP78398 (+) Dm CG2961; range -250 to 49. []

TGTTTTATTAATAATCTACGAAAGATCATAAATTTCCCTTATCTCACAGCAAGCACCCTGTGCCGCCCCTTGGAGACCTCAGGTGAATTTCGAGAGCAACATGGTTTTTCGATGGACTTATGACTGGTGGATGACTCCACATCGACGGCTCTCCGTCGCTGACGTCACGGGCCATCGCCGCATCTAACGGTTTGTGGTGAATCTCCGGTGGAACTCTTGTATAAAAACGTTGGATCTGCTAGAGATCGTCATCATTCACTAGCCGGGAACAGCTCGACGCACAGGTGTCGCCTTCGATTCCACCTATTTTTTTCCAAATTCGAAACAGAGATAGCTCAACGCTGAAATGA

>ID_3133 P_3538 Promoter_3608 D3_EP78405 (+) Dm CG8426; range -250 to 49. []

CTTTATTTGCTAATAAATAATATATGTAGTATAAAATATATATATATATAAATATAATTATAATATTATTTAATAAAAATCTATCAATCCTTCGAAAACCAGTATCTAGTATAATTCATTGCTTGCTACGTTAACACTTACTTTAGTGTGGAGAGGACTTTACAAATTTTAATCCAGGGCTGCCACGAAAATAGATAACGTATTCGATATTTTAATATATGGTATATATGGTATTCCTGGTATGTATTTCAAGATGGCGGAAAGTGTGAGGAAGAAGAGAACAAAAAGCAAGCGAAAAATCCATGAGTGTATTTATTGATGTATATATGCGTAAATCTATGTATTTACTA

>ID_3144 P_3555 Promoter_3625 D3_EP78422 (+) Dm BCDNA LD29892; range -250 to 49. []

CATATATATTTTGGCACAATTCAAGCGTGCTGGGCGTTGTATTATTTAAACAGAAACCTACAAAATTCTGCCTTTATAGTTAGTTTTTATCCTGTTTTTATTTAATGAATTTCAAATTTCAAATATTTTTGTAAACAATTTTAAGCTAGGCCAAGGGTTTGTGTCGCGGCTAGTGCAAGCAGTCAACTTGCGGCATTTTTTAGTACATTTCCCTCTGTGCTGGGTTATATATTCGCAGGCTATACTCCACACTGCTTCCAGCGGAATCGTCAAAAAAATAACATTAAAATCGCCGTGTGCATAATTAGAATATTTCCGAATAGTAATCGCCCGTGAAATCGCTAATTAGC

>ID_3170 P_3592 Promoter_3662 D3_EP78459 (+) Dm CG1440; range -250 to 49. []

ATAGGCCTAGGCCTAGGTCCAACGACGCTGCCAGATCAACAGATCGATATAACAGTTACAGTTTGATTTATAAATTAATACGCTTATATGTATGGAACATTTATGAAATTCGGTGTATTAAAACAGTGCTTAATAATTTAAAGAAGTTGGTATATTACCGGTATATCGTATGGCAATCCAATGTGGTATTAAAAGTATATTTTTTTGAAAGCCGGATGGTATAAAAGAGGGTTCATTGTATGGTCATACTATTAGGAAACAAAAAAAACACTCCACAGCAAACCAAAATAAAATCAAATTAAAAAAATTATCCAAATTGCGCATAGTACGACTGCAGTAGCCAGAAGTGG

>ID_3171 P_3594 Promoter_3664 D3_EP78461 (+) Dm RNRS; range -250 to 49. []

TTAATATTTTTCAAGTGACAAGCTGGGAAGCTAAACATAAAATTGTGCAGTAAGGATTCGATTTATGGTTTAAGAAAAGAAAACTACCACCCCATAATTGCATTAGATTTACCCTAAATTTATAAAAAGTGAATTGACGCACTCGACAGCCCTGATTTTCCCATAGTTTTCCCATCACCAAAAATGGCGGCAAATCGAAACAGTTTGCCGCCCGGCATAAAACCCAATGTAGCTGTATTTCCCAGATCATTTGCCACACAACTTTCAAACTGTACACTTAATACACGTCGTGGTTTAAGTGAATTTTACCAGAGAATCAGAGAAGCGCCCCTACCTGCTAATAATAATCC

>ID_3178 P_3609 Promoter_3679 D3_EP78476 (+) Dm CG5295; range -250 to 49. []

CCCAAGAGATAATGTCAAAATTGGGTGCAACACATATACTAGCATATATATTTTTGATAGATGTACTTATATATCGATAATTAAATTTTATCAGTTATAAAGAATTGGCATTATAAATCGATTTAAAGTCGTAATATCTACATGTTTAGAACAAACAAAAGAGTACCCTATACGCACAGCCACTCTAATGACAGTTCTAGTTTAGGTGGTAAAGCGAATAAAATGCGGTCACACCGCGCCGCAATGAATGTATAAAAGGGGGCTGCCATCCGCAGAGCGAGTAAATAACGGTTCGCGAGCAACAACTTTTCATTTCGAGAGCGGAGTAATTTTTCCAGCCGACCGACAAA

>ID_3191 P_3628 Promoter_3698 D3_EP78495 (+) Dm CG16844; range -250 to 49. []

TGGATTTCCCTCTTCCAATCGCGCAGCTCGGGGCTATCAGTCTGCACTTGTTTACGCTCAATTTCATTGAGAACGTCGCACCGCTGCACTCGGGAATTTCTCGATGGAGGTGTTGGCTTTGGCTTGAGCTTGGGAGAATCCCAATCTCGGCCAGACTTTTCCCGGTGTCGCTGCCAGTTGGTTGATAAGACAGATCGGTACACTTGGCTGCTCTATGACTATATAAAGCGTGTTGTTCTCAACACTTAGCATCAGTTGTCAACGGCTAATACGCGGGAGTCAAGCTCAGAATTCAACCACAAAAACCAACAACATGAAATTCCTATCACTCGCCTTCGTTTTGGGTCTGC

>ID_3204 P_3650 Promoter_3721 D3_EP78518 (+) Dm BEST GH09876; range -250 to 49. []

AACAGCGTTGTACACACACACACTTGCACATTTACACCTGGGTATTGAAGTTATCAGCTAAAAGAGAACTGACTTCCTCCGATTGTACATATATATATATATATATATATATATGTATACAAACATATATATGTGTGTAGAGCAGATAACCACTGCATGCGGAGAAGAGCTTGGCAGGTGTTAGTGAGTTCAGGTGAAATTGATTATGCGAATGGGGGTAAAAAAAATATGGCCAAACAGTGAGGGAGCCAGTTAAAAAACGGTGGTCGATCCAGCCGGTCCGACATTTCAGTTGAGAAGCCAGAAAGAATAGAAAACCGAGAGCCATGAGAGAATACAGCCTGTGTATG

>ID_3206 P_3655 Promoter_3726 D3_EP78523 (+) Dm CG11151; range -250 to 49. []

TTCGTGCAGCCACTTTATTTTGAGTTCATGCCCACTGTGCATCAGCTGGCTAACATTTGGAAGAGAGCGAGAGAGAGAGGGAGCGCGACAATAACAGCACAACAAGGCTTATGCGAATGCAACAGGTGCGAGTGAGGGCGACATATCGCGACCGGCAACTTGCTATACGACCATCCCGCTCCCACACAACAGGGGCAGTGTAGTGAAAAAAGCGACAATAAAATCGCCGATTGCACGCCTTCTGCTCATTACTTCGCGTTCCGCTCTCAAAGCGTAAGCACTTTTCTTTTATTTCTACGACGCACAAGCCCACTGAATATTTTCTTTATTCTTCCGGAAAAACAAAACCG

>ID_3233 P_3697 Promoter_3768 D3_EP78565 (+) Dm CG18135; range -250 to 49. []

ATCTTCATGTTTTCTGTATTATCTGGTGTGTTTCGCTGTTTTCTGTTTTTGTTTCGTCGCTGCCCGTCGGTAGAGGCCAACAACATTTCGAAAAAGCCCCAGCCGCAACTGTACCGCCGGACTGAAAACCAAAACAAATCGCCAAGTCGTCGGATACGACTACGACTCTCGCACAAAGAAAAAAGCTCCGCCACTCAGTTTCGATAAAAAGAAATCGTTTAAATTGCGCGCACAACAAAAGCGCCCGAAACAGTTGCGATTGCAACGTCGATTTGTGACGACGGACCGTCTACAGCGTCCTAGTTCCCACTGTCCACAAAGGATACAAACCCGAATAACAAAACCAGCAG

>ID_3234 P_3698 Promoter_3769 D3_EP78566 (+) Dm CG9307; range -250 to 49. []

GCAAGACAACCTACCAGCTAAACTGATCTGGAATTGAGCACACTCAGTCAGCAGAAACAAAACAAAAGCCCCACTTTGCCCCACTCCTACCCGCGACCATTCCCGCAAAGTTGCGATTCTTCGGAGAATCGCACCTCTCACGCACTCTACGCAGGAACAAAGGCGCAATCCAAGAACCGGACTGCAAAAAAAAAAGAGTGTTTCGAGACCGACTCCCACTATAAAAGTCGAGCCGATCGAGAGCTTCACTGTCATTTGACGCCAAACGCGCGTCGCACGCAGAAGTCTCGAGATTCCCCCGATTTCACAATATAGTATATATATAGTTATATATTCAATTCTTCGCCGGA

>ID_3263 P_3747 Promoter_3819 D3_EP78616 (+) Dm CG12391; range -250 to 49. []

AACTTTATTTCGGTTTTATTGTTTTAAGCTTTCGTTTTGGGCTGCTGAATTATGTTATTTGCGAGCATTCTTTAAATAACGAATTTCCTTTACTTTTCAAATTACGCGCCCGCTGTGCAACCCTGCGCACGTTATCGATTGCCAGTGGGCATGCGCACAACTATCGATTACCGATGACAAACAGCTGTGTACCTATACTTATCCAATATGGCTGCAAGTAAAAAAAATTGTAACGCTGTGAAAAAACTGCAATTTGAATTGCATTTTCCGCTTGAGTATTAGATAGAAAAGTGCGAGAAATGTGAATTTGTGCAAACGAAAGTGGAACACTATCCGAAAATCAAGTGCGC

>ID_3271 P_3759 Promoter_3831 D3_EP78628 (+) Dm CG17337; range -250 to 49. []

TTCTTTGGATCAAACTTGGAATTCTGTAACTAACTTATAATACGTAAGAGAAGTGGCGATGGAATCCATTTATTATGGAACATATCAACCAGATGATGGATGCATTTTCGTGGAAATACACTAAAAATTTATGCACATATTTAAGTATATAATCGCGTTATGGCCAAGTATTTTTATTTCTCTTCCGCGGTCACACTACCGCCATTACTTGAGTTTTATAAATATAAGCGCGCGCCGACAGCAGGTTAGATTTAAATAGCTAGAGACCGGAGACGATTACAGCTGTTACAGTATTCCATAGAAAAAAAAGCGCGATGCCCGAGTTATCAAGTGAACTTCAAAAATTCTTT

>ID_3277 P_3767 Promoter_3839 D3_EP78636 (+) Dm CG17597; range -250 to 49. []

TTAGCGCTTTTAAACTTATAAACAAACAAGGAAAGTGCTCTTATAAGCATTATTACATTATTATTAGACTTTCTAAAAGATAGAGATTCCCACCAAATGCTTATGTTGTATGCACGTTTCTCGTTTACAGATGCATATTTATGAACTGCATAGAAGGTGAACAACAAATTTAACGGGAAACAAGCTGAACCGACAGATCTAGCCTTATCATATCTATCGTATATAAGTCCGCAACCTGTTGATCCACTTCAGTCGATACAGAAAGTCCAATCCGTGATCCCGCAAAGAACCAACAGCCGAAAAATGACCAAGACCAGAGTTTACGTTATCGGAGTTGGCATGACCAAGGT

>ID_3280 P_3771 Promoter_3843 D3_EP78640 (+) Dm SSL1; range -250 to 49. []

AGTGCCCAAACTTAGGGTGTAGCGCCCTTTTCAGTTCGCCCGTGTGGCGGTGCACTTAGTTAGCCAATTACGCGTCGTGGTGGGTATATTTATTGATGTGGCAACTTTGGGCGCAATAATAATAATATTGTACTTTTTTTGTTCATATTAGGTAAGAAATTCGAAATTATTTTTTATTATTTCATTTTATTATGTCATTCTTTTGTAGATATTTATTGATATAAATACTTACCCAGGGTTGCCACCCAGTGTATTGTATTTAACGTAGGCCCTGTGTGTACACACTGTTACATTAGCCGGCGCATTTTATTTAGCGTTAATTGCTTTTCTTTCTTGATTTGACTTGGTAG

>ID_3307 P_3827 Promoter_3900 D3_EP78698 (+) Dm CG4617; range -250 to 49. []

AATTAATAATATAATAAAAAAATTAAGTTGTGCGTGCACATCCTGTGCTTATAATTTAAATAATATAACAATAAAAAATATTATATTATTAAATAATTAAGTTTAATGGTTTCCTACTCTGTACAATTGACTGGTGCAATGGTTATTAACTTAGCAATATATTCAAAAACTAAAATGGTTATCTAGTGTTCCATATTTAACCATCGCTTGCAATGCATCGCGTTTTAAAGCTCTGGTCACACCAAACAGCTGTTTTGTTTGCTTCGTTTAATTCGTTCTTTTTGCAACAAATTGCATCCCAATCTTCCAAAGCAGGTATCCAAACATGGAGTCCCCAACCTCGCACCTGG

>ID_3308 P_3828 Promoter_3901 D3_EP78699 (+) Dm CG18107; range -250 to 49. []

AGAATTAAAAATGCAAATCAAAGAAATACATACAAATAGAGGATTCTATTTCCTTTCTAACTAAACAAAAGATGTTTTTTATTTTTAGTATTTTCGAATTAATCAAAACAAAAAGTGTAAATCTATATTTCCCACGAAAAAAATATTTTGTCAGCAGCTATCAATCAATCGCTCTCATCTCATATGGCACTTGATTCACTCCTAGATCCGACTATCTGCTATATAAGATCGGTTTCCCTTAATCGAGAGCATCAGTTGTAGTTGATCGTTTGTCCGGTGTGTTCAGCTTATAAAACCGGATCTATTAATTGAAAATCAATATGCGATTCTTTGCAATCGTCACTGTCTTT

>ID_3317 P_3855 Promoter_3928 D3_EP78727 (+) Dm CG13463; range -250 to 49. []

ATTCTTATTTTCTGATTCATAGCACATTAGCTTGTTTTGCTCCGCTTACTCGAAACTTCTTAATATTTACTCACATGTTCGGTGTCGTATTTGTGGTAAGCCAAAAACAATAACTACTTCTAACTAAATATATCGTAGGCCATGATGGAACCTTGACGATTGTTAGCATAATGTAATCATGTCACAATAAATCGATCGATTTGGACAACTTTTTAAAGATATAAAAACAAAGATAAGCTAATTGAAAATCATAGTCTCAGTTGCACTCAGCCTGTATACTTTTGATAATCATGCAATCCATAGCATTTGCTCTTCTGATCTTTGCCGGTATCCTTGCCTATGGCAATGCA

>ID_3320 P_3862 Promoter_3935 D3_EP78735 (+) Dm ARR1; range -250 to 49. []

TGTACATTCACCTGTCCTGCATTGCTAATACGCCGTGTTGCTCGCGCGTTATTTAATGTTTGAGCCATCGATGTCGATGTCGTGCTGCAATGTCAATATCAAAGACACTGCGCACAGCAAGGTTGCCGAAGCCGTAGTAGTTTACCGCCGTGCAATTGCTGAATTTCTGCTGTGCGGCTAATTGAATTTAGAGGGGCGACAGGTGCCACAATGCCAGGTATAAATGCCGGATTGCCAAAGAGCGCTAATTAATAGCCTAGTGGACCACGCAACGCGGCGTATACCATCGAGAACGAGCGCGAAACGTTAAAGGCACATCCAAAGTTTAAACTATTTCCGCAGAGATTTTG

>ID_3321 P_3863 Promoter_3936 D3_EP78736 (+) Dm TUBA84B; range -250 to 49. []

TGAAACATTCAACAAAAATATTATTAATGTTGGCCCACTTTAGCAACCGGTTAGGTCTACCGGTTGGGCAAGCAAAGATTCACGCCCTGGTTCGAGTCCCAACTAGTCCTGCAAAATACCGCAGCAAGTTTTAGAGAGACCAAGTGCCATTACCTCTCCCACTTCAGTTATCGGTTATGCGGCGTTTAAGTCGACAGCTTGCCGTCTCTAGCTCCGGTGCCTATATAAAGCAGCCCGCTTTCCACATTTCATATTCGTTTTACGTTTGTCAAGCCTCATAGCCGGCAGTTCGAACGTATACGCTCTCTGAGTCAGACCTCGAAATCGTAGCTCTACACAATTCTGTGAAT

>ID_3328 P_3872 Promoter_3945 D3_EP78745 (+) Dm CG10990; range -250 to 49. []

GACCATTCCCAGAAGCCATCAAAATTCAATAAGTTTCGTTTATCTTAGTTTTCCTCATAGGCAGGCTTACTACATAGGATTGAATTTGTATTTACATATATTTGCAAACTTTTTAATAAACTTCCCCGTTTTAAGGGGAAGCTTCGCCAAGTGCGCTTTTATAACCCACCACCTTACCAGAATCGAACAGTCTGGCAGCACGGTCTCGCCGAAAAAGTATAAAAACCCACGTCTCGTCGCTCTCTGGTTCAGTCGAGTTGAGTTTTCACGGCGTGAACAACAGTGTGCGTTATTGGTTCCTTATTCTGCGGTTCTTCAATCCTTTTTGTTCCAATCCTACTGAAAAAGAA

>ID_3332 P_3877 Promoter_3950 D3_EP78750 (+) Dm RPD3; range -250 to 49. []

AAAATATGTCGTGATATTTGATAATTTTTGATAAGTACATAGGTCTGATTGTTTATATATCTAATAAAAAATGACCACTCTGTATGAAGAGGGTAATCGTATTTCATATGTAAATTTATTAAATATATTTCTTTAATAAATAATTAAATATATTTGTTTGGTATTTTCGGTATTCATGCAGTTAGTATTAAAAGTATATTCTGCAGAGCCGGGAAGTGTGGATTTAAAAAAGTCCGCGGTCACACTGCCCATCTGCAGAGGTTTCCTCTTCGCGAATTTGTCTTGTGCGCTCCGATTTTGGCTATATAGTTGCGCCAAAGTGCCAAAAATCGGACCGGCACCAAAGTAAA

>ID_3357 P_3914 Promoter_3988 D3_EP78789 (+) Dm CG6910; range -250 to 49. []

AGGGGTAATAAACCAAAGTGTTATATGGAAGTTAGATCATTTGAGCCTGGTTTCCGACGTAATATTGATTGCGATTGGGAGAGGCCGCACGGGAAGCGGCGCAAGCGATTCTCGTGTTTTGAAAAATATGAAACATACTATAAATCATTTTCGAGTACACTTAGAATAGAAGAGAGTGTACTATATGGAATGCGATTGTGAATTTCCGATGGTCGGCCGACTATAAAAGTCGGAGATCTCTGCGTCCAGCGATTCATAAGTGTTTGCTTCGCTCAAGTGCGCGGTTACTACCCGAAAATATTTTTCAAGTTCTTTTCAATTAAATCTTAAAAATGAGAATCCTTGCGGAG

**Supplement 2.** [*(Next)*](#Supplement3) [*(Back to the top)*](#_top)

**The list of *Drosophila* promoter sequences with potential Initiator at its functional position**

The TSS positions are marked by red color.

The center of the Inr box positions (first ‘A’) are marked by yellow.

If TSS position coincides with position of the Inr center, only the latter is marked.

>ID_1 P_1 Promoter_1 D1_Promoter for RE28544, -250bp and +50bp []

ATTTTAATTATGATGTGATAGAAATATGCTATATAACATAAATTTAAAATCCATTCGTAATATAAATTAATTTCCAGTATCACTTACACCGTAGAAAATATTATTTATTGTTACAAGTTTAATGTTTTGATTTCGCTTAAACCTCAGGGTGGAAAATTTAAAACCAAGTTAGGCGCCTTCAAAAAATCGCAGTTATGACGAATGTAATTCTGTGACATCGATAACAAAAGCTGCATAGTGTAAGTATACTGTTTCTCGATATCGATTCTTAAGAGAGCTGCTGCTTCGAATTGTGGAGAATTTGAATTAAACTTATAAGCGATCGCAAAAGTCAGTTAGCAGTAAGTGAG

>ID_2 P_2 Promoter_2 D1_Promoter for RH51703, -250bp and +50bp []

TACAAGTGCGGGCAGCATAGTATATACACCTATTCCAAGTACTACAGACATTAAAGAATATTTAAAACAAATTAAAGTCCATTCATTCATATACACGTGCCCTAAACGTTTTCCCGCGACTGTCCACACACATTCACACGTAGGCACATGAGAAGTAAATATAGGGGAGACCCGAAGTGGGCAGCAAAACAAGAGCCGAAGTATCGCCAGCGCACGAGAGCTACAAAAATGCAGTAATTCTAGTGTTCGGCTTTCAGTTTCACGAGAGACCTCCAACAGAATACATCGAATTTCGGCGAGAGATTTGCAGCTCAAGAAGAAGAAAGAAGGAAAACCTCTGCGAGTCACCT

>ID_3 P_3 Promoter_3 D1_Promoter for RH40711, -250bp and +50bp [3 ]

ATTCCGTTTATCTCGTCACCGTACTTGAAATACACAATTTGTTGTATTCTGTCTTCTTTTTTTGTGTGTTTCTTCTCTCTCTACCGCTCTCCACGCTCTTCTTGTTCAGTAATTTGTATTCTGATATTTGTGTTAATGTAGTATATTTTTTTTCTATAATATTTTCTTTACTTGTTTTTGCCTATTTTGTGGGCTCTTTTCGCTGGCCGCTCGCCGCTGGAATTCGATGTGAAAAAGTGTGTTTTTAATTTCAGTTTCGGTTCGGAGCTGGCGCGATACAGTACGCAGCTCGCGCCTATTAGTATCTACGTATAGCAAGCAAATCGATCGGATCGTGTGGCGGTGCTCTA

>ID_4 P_4 Promoter_4 D1_Promoter for RH58777, -250bp and +50bp []

AAATTTTATTTTACAGAGTAAAACAGAGTAATTATTGTTAAGATTTTTGTACCGTATAATCTCATTATTTGACTTTAGTGGATCTTTTTCTCAAACTCGAATATTTACTTTTCAAAGTTGTTTACGTTTCTTACATTGGAGATGGAAGCAAGTGCGCCTAACAGCACGCAGATAACAGCACGATATCTTCACGTGGTTGGCGTTATTTAGTCCCTTTTCTGCGCTCTATGACAGCCTCTCTGGCTTTCCCTTCTTTTCCATCGCGTTTCCGTAAGTGTGCTCCGCGTTTTTCCAATTGGCTATTTTTATTTATAATATCTTCAATTGCAGCTAGGAATTCCAGTGTGCGA

>ID_6 P_6 Promoter_6 D1_Promoter for RH20790, -250bp and +50bp [7 ]

GGTTGTCAAAAACAATCCAACTAAGCCGATATTTTCGGATACCTATTTGTAAAAATCATATTCAAATTCGAATTTAAATAAAGCGGCAAGATAGCGAAGATGCCACACACTGTTCCTTTAGCGAGTGGGTCTATGAAACAGCGTTGCCATTTAGTTCCTCAAACAGCGGTGCTAGACGTTCTATAATCGCTGCGATCAATCGTGGAAAATATCGATACTGCCACCCCCAGGTCCGGGTGCCATCGCTAGTTCCTTTTCTTTTCGAATTTCTCGTGGAAAACGCCAACATGGGTTTCGCTACTCTCTGGTACTCGCATCCCCGCAAATATGGCCAAGGCTCCCGATGCTGG

>ID_7 P_7 Promoter_7 D1_Promoter for RH66381, -250bp and +50bp [2 ]

TAACAAGAAAGAATTCATATGCTATGAAGATGTTTGACCCATTTAACGGTAAAGACAGCTAGTTATAAACTAACAAAGCTCTCAACAAGTTCCTACTGATACACACAGTGCAACATCGCTAGCTGGGAAACTGGTGTTCTATAAGGTCTCCGAGCATTTATATTTCAGTAATGCTACTGATAAGGAAGTATCACTTTATTGTTCATAGTATTCCGGACTAAGCAAAAACGGAAACGGAACGGGCCGGATTGCTCAGTTGCTTGATGGAACGCGACGTGCTCACACCGAAAACTAGAACTCCTCTCTGATAAGACGGCACCAAGTTAATTTGACCTCAAACAGAGAAAGAT

>ID_9 P_9 Promoter_9 D1_Promoter for RE38785, -250bp and +50bp [9 ]

TTTTATTCTTTCCAATTCTTTTACCGTTAAAATTGTATTTGAAAATTATTAGTAACAAGAGTAAAATATACATGAAAGTCTAGGCTGCAGTATGTAATACGTCAAATTTTGTTGGGACACTAAACAACAAATAGTCGTCATAAAGGCTTCTTTTAAATTGTTATGATATTTTCCACAATTTCTGCATACTTTTTTAAGCTTGCTGGCAACGCCGCCACCGTACCAATACAACTATCGATAGCCGCCTGTCAGTTTTGACAGCTGTTTTAAGGTTATACTGCGCTGCCGTTGCAATCGCTCCACCAATTTGGTAATTTTGCTTATTTAACATCAAATATTGCATAAAACTA

>ID_10 P_10 Promoter_10 D1_Promoter for RH37353, -250bp and +50bp [5 ]

GGAAAATCGGAAAAAGATACTTGACATTAGCCAATTTGCTCGGGCAAGTGGCAAACTGCGCAGAAAATAGAACAACCCCTGGAAGCAGCTTGTTTGCAGTTTTCCCTTAGTTCCTATGGTTCTATCTTATTGTCGTGTTGGACAAGCCAACAAACAAGTGCATAACGAGCAAATCCAGTTAGTGGCCAGAATGAAGACCCACGGACAGGGCCAGGGCAAAGGTATATAAGCAGGTTGTACGGCACTTGGGTTTCGCATTTTCCTCCGCCGAACGGAAAACTCTATAACGCGCATCGCCCGACTTAGCCTCGAATTGCCCAGTTGCATTTGCCAGGAATCAAAGAAATCCA

>ID_11 P_11 Promoter_11 D1_Promoter for RH15304, -250bp and +50bp [7 ]

GCGTAGGAATAAGCGCTGTCAAAAAGAAAGTATATATTTGAAATTAGCAACTAGCAGTAGGTATAGGCCTAGTCCACCTTATTCACTCTCCACTGTATTTATTTCTAATGATCGCACGTCCGAGCTTAGCTTTGTTTATCAGCTCACACTTTGCTCTCAACCCACAACAGCAACAAAAAAAAAAAAAAACAAGAACAACAATGACGACCACGTCTCAACCTTCGACAAGCTGGGATGCGAAGTAAAGCCCCAGTTTCAGTTCCAAGTCGACACTTGAGCGGCGCACAACTCAACGGTATCAAGTGAAATCCGCGAATCAAAGATGATCATTAAAGCCCTAGCGATCGTTT

>ID_12 P_12 Promoter_12 D1_Promoter for RE66383, -250bp and +50bp []

ACATCGTATACAAAAAAACGTAAGCTTAATTTCGATAGTATCGGAACTTGTATTGCAATCCAACCGTGGGTCAAGTAAATGAAATAAGATAAGACTTTGTTTCAATTAACGTTGGCAGTGGAGCTTCCCCCAATTGAAATATGTGCCAATGGAATCGGGCTATTAGGTCAAGTGAATTCAATCGACTGGCGCGGAACAGCAATCGATGGGTCCACACTTATCGCACATAGTATCGCATAAACATCGACGCAGTGGGCCACCTCTAGCAATTTGTCTGCAACTTCTAATTCGCTGCGGGTATTTTTTTTGTGTTTGTTGTTACATATAATCCGATCAATTTCGATCGCCCG

>ID_13 P_13 Promoter_13 D1_Promoter for RE24408, -250bp and +50bp []

ATGATTGTTTTTAGTGAAAGATTAAAATTACTAGATTTAAATTGAAGTAAATATCTTTCTCCCGAACTTTACTTTCGAACATTTTCGGCAAGGTGTTTTGGTTTGAGTCGTAGCTAAGCAGTGCAACGAACCCAACCTGCAATAAGCTTTGAAGCTCAACAAAGTTATATTTGTTGTTATTTTTTGATTTCGTTGCTACAAATTTTGAGAATTCAAACATTGCAAGCGTAGATTGCCGACTTGAGAAATCAGTTGCCAGCGAGCTTTCAAGCGGACAACAGGTGATCGCGACTGTTTCGCAGAAAAGGTGAAAATCGCGTACAACCGAGAGCGAGTAAAGTCGCCCCAAA

>ID_14 P_14 Promoter_14 D1_Promoter for RE29473, -250bp and +50bp []

AATTTCCAATCATAATTGGGAGATCTTAAATGCAGATCTGCAATAGCAGATAACCATCGTCACTTAGACTTCCTATAAACAATACCTTTGCAAGATTATAATAATAAGAGAGGCATTCGGTGAGACCTCAAACGAGAGATAACGCTTTTGACAGTTGACTCGACTGCTCGTTTTGGAGCCGAATCAAAGCCGATTGCCCGGCTTAAGTCGATTGCGGTTCGAGAACTCGCGATTTCAGAACCACTGATCAGTCAGTCGGGAGTTGTGCAGCCGGTAGATCGTTTCTCCAGGATTTCAAGGCAGATTTGGAAGAAAGTTCTGCCAAAGTGTTTTTTATTATATCCAAAAAT

>ID_15 P_15 Promoter_15 D1_Promoter for RH32426, -250bp and +50bp []

TATTTGTATGTATCGTGTGTACAAACATGTAAATTAAAATCGACAGTTCACACTAATTTTAAACATAGAACTACTTCGAAGTAAAATAATTTTGATTTGCAAAATGTAGCAATAAATGTTTAATTGATTGGCCAATGAGTAGTAATAACCGCTATAAATATAATCATTAAAGCCGCTGTAATTAAAAAGACTAGACAGACATCGATAAGATACGATAAAATACGCTATCGATACTTATCTAACGCATTACTAGTTGGACGGACCGCTTTGCCATCACTAAACCAGGAGAATATTGTTTCGTTGAAAATTTGAGTTATAAATCTTAAAACTTTAGTTATTCTCAGAATTTG

>ID_16 P_16 Promoter_16 D1_Promoter for RH26077, -250bp and +50bp [2 ]

TGCTGGTTAAAGAAGTTTCCCATTACCTGTTACCCGATGGATACTTAATAACCAAAAAAAAAAAAAAAAAAGGGGAACGAAATTTTAGAGCTCATCAGAAGTGTTGCAAAATCACTGACGCTGCGAATCAATGCATTTGTGCTGATCCAGAAATCGGTTGCTGAAATCAATTGCTTCCGGCGCGTGCATTATAGTGTGTATAGACTACCGATTCCAAGGGGTATAAAATGCATTGAGTCGCAGCAGTGGGTATGCAGTACAATTTGGTACGGTGTCTGAAAAAGTCGAACTTGGAAGCCACAATGAATCCTCTGCGCACCCTTTGCGTTATGGCCTGCCTTCTGGCGGTC

>ID_17 P_17 Promoter_17 D1_Promoter for RE18044, -250bp and +50bp [5 ]

TATAGCCGAAGATGATAAATCATAGCTTAATCTTGGAGTCAAAGAAAAACGTGAACGTGAAACCGATAACAAATACCTAATCTCGCCCTGGCAACGAAGCGAGTAATCCGCCGGCGGTGGTGCCGCGTTTCTAGCGGGCACTCCACCCACAGAGCTTTTCGGTGAAAAGTTTGCACCGCTCTCGCCGGAGAGCAAAAAGCTCGGCGCTCCGAAAAAGCCGACATATATATACATATCTATGCGAGTGCCTCGGCCAGTTTGGTGCGGCGTCGAGTAGCGAGCGGTTGTGTAGAACAGAATAAAAAAAGAGTTGGAACCCGAAGAATCCAAGCGATTAGCAGCATGTATCA

>ID_18 P_18 Promoter_18 D1_Promoter for RH01507, -250bp and +50bp [6 ]

CAGCCTCTCAATTTGTTGTCATGAATTATGCAAGTGAGTCTGGATTTCTGGACTCGCAATTGGCGCAAACTCGGAAATTGCGCAGTGCCGCGGAAACTGAAAATGGGAGAGAGTGTTATGTGCAATCTATATGAAATATATGTATTTATACATTCGCATTTGATGGAAGCTTTAATTACGGTTGCACCCGTAATCTGAAAATCTGGACAGAAACGAACTTTTGAGGTTTCTTGGCAAATCTTTGCCATCGGTCGTAGTCAGTTTGCAAAATAAGGTCTTGGCGCAAAGTGTGCCTAGAGGAGCACAGGAGAGTTTACATACCTTAAAAGGTTATACCACAAGGACTAGAT

>ID_20 P_20 Promoter_20 D1_Promoter for RE66429, -250bp and +50bp []

ACAAAAACGTCGAAACAAGCCGTTTACTGATAAAGCTTTCAGTTCCAAAACCGAGGGAGAGTATTTGAAACCGGCAGACGATAACGTTTCAGAGTGCTACGATGATCTAATGCTGAATTGTGAGCGCAGTATATGGAACATGGTAGTTAAGAGCGGGGAGCAGGTGAACTTGAACTTGGAGAGGCGGGAGTCTCTTTCGAGGGCCGGAGAACCACTATATATATGGGGCTACGGAAGGCCCATACCCTCAGTCGCTCGCAGTTCGGCGTGGAATCAAACGAGGCTGACAAGTGGACTCATTGCCACATATTGTGAAATAACATAGTTTCCAGAACCCACTATAACCATCC

>ID_21 P_21 Promoter_21 D1_Promoter for RE08425, -250bp and +50bp []

CCTTTGATTTTTGCGTAACATATTATATGAAATTCTAAAAATATGCCACTGTGTATGTGTACTCCGTTAACTTATAAAAAAGGGGGTATTAGATTTAATATTTTTATAAACAATTGAAGAATTTATAATTCTTTATTAACCAGCATTTCTCGTGAATAGCCCTATTAATGAATATCGATATATATTCGCACAAGCACACGCGCCCACGAAACTATCGCCAGAGGTTTCTCACCTCTAAAAACCGATTTTCACTTCCTTTCTAAATCGCACGTTGAACAGAAAAGATGGCGTAAGTGTAAATCCAGATTTTAATATGTAAAAGAAAGCAACTAAACATTGGGTACATTAAT

>ID_23 P_23 Promoter_23 D1_Promoter for RE01730, -250bp and +50bp [6 ]

TCGGATTACTTTGAGTCTTTATTTTGGGAATTTTTGTTGTTTAAAAGTTCTGAAAAATTCGCTTGTTCAACGATTGCTGGCTCAGCGAAAAGTGGAAGCCAGGGGTTCGTTTACTTAGTGTTGTGGTCGTAAAACGCAAATCGCTTGTCGGCAGCAGCTTGTCGGCTTTTAAAGTCGCGTCGTTTCCCCATTCGCAGTCCTCAGATCGCAGTTCAGTTTGCAGCTCAGCTTGGTCTGCTAACTTGGCTCAGTCTCAGTTTGGGTTCCAAATCTGTCGCGTGCGGATCAGATCAGTCTTTACAGTTTATGCCCGATTTGACGCTAATTTCAGCTGACTTTTCGTCTGGCTG

>ID_24 P_24 Promoter_24 D1_Promoter for RH58949, -250bp and +50bp []

AGGTAATATTTTAATGGGTAATCTCAAGATACGAAACATTTTCATTATTATTAGTAGAATTTTTCTTTTACTCAAGCATTCTTTAGAAGGAAAGTTTGTGAAAGTGTCTTAGCATATATGTATGAACTACATGCATCTGGTACTCTAAGATCAACCCTGCTGACCAGTGTAATCGATACGAAAATCATACTCAAAATAGAATGGCCACTAAGTCAGTTAGCAAGAATGTTCGCCCGCTAATTGTAAAATCAGTTTAGGCGCACTTTTCAATGGACTCAAATCGGGCGCAGCCAACGCGGAATATTCACAAATAATAATTAAGTGAAAACCCATAACAATAAATATTCGAT

>ID_25 P_25 Promoter_25 D1_Promoter for RH05508, -250bp and +50bp [4 ]

TTAGTTGCGACTTCTGGTATGGTATTGTGAAATATTTAATTATTTGTCTTAAGATGTTACTATTGATGATCGGAATTCTACTATATACATGAAACAAATATATTTATATCAAATAATTGGACTGAATTTTATTACATAATATTTATTTTGATAACAATTTAAATTCAAACACGATATAATTTGGTGCTACGTATCGATTCCGACTATCGATTGCATGCAGAGTGTAGTACCTGCACTGACCCGTTCTGCAGACGTTGTTCAACTCTAGCCAGCAGTTGTAGTTCGTGTCGGGATTGTTTTTTAGATTAATATTTCTCAACTTTTAGACCATTGATTGCCGAATTGGTCAA

>ID_26 P_26 Promoter_26 D1_Promoter for RH66848, -250bp and +50bp [2 ]

TATAAACATAGTGTATAGGCATATGCACAGACAGAACAGAGACAGATGAGGTCTCTTTGACGCGTTTTGCTGGCGTCACAAGCGGCTTTCTCGCTCTCTCGCTGTCGCATTAGGTGACCAGAGAATTAGAGAAATAACGTAGAGAACCCTTGCAGCAAAGTGAAAACAAAATGTGGCGAAAACAAAGCCAAAGAAATGTGCCTGGCGGTGGGCTCTTCTGGTCGCAACTGCTTAAATTAAATACATTGCTCACTTTCTGTTCAATTGGCGAGCCCAGCGGACATCGGAGGAGCCGCTTCAGATCGCGTTCAGTTCAGAGTGGATTCCGTTCAAGATTCCGTTCCGTTAGA

>ID_27 P_27 Promoter_27 D1_Promoter for RH33713, -250bp and +50bp [1 ]

ACACCGCTGCGGATTACTAAACCACTACCACCACCAAATTAAAATAGTAATAAATAGATTAGTATACATACATACTTTTCTGGGAACTTTTAAATTTATTTTCACTCTGTTTTAAGTTCAGTTTGCTGCTTATCGCACGAAGCTTTGCTTATCAATCGTCCCAGCAACCCCAGAGAGCGATCTCGGCCGGACGCGCGCGCGTTTCGGGCTTAAGAAAAATATTATAAAAGCGAAGACGAGCTTTGGCTTTCGTTTAGTGTCTGAGGCAGAGCTCTGCATGCTTGTCGTTCGAAGCGCTTTTATTCAATCATCGCACTGGGATCGTGAGAAATTGCGCAGATCGTATTGAC

>ID_28 P_28 Promoter_28 D1_Promoter for RE73632, -250bp and +50bp [1 ]

CAAAAATAAATTGGAGAATTGAACATTTCAGTGCTGCACAACATTGCATGGTTGGTGTCCCAACTAAAAAGTAACCCGTGTTAAGAACGTATGAGGGTGGCATCCCTCAAAATCCATGGCGAGCAGTGTTACCGTACTGCGAAAAGTGACCTTAAACCCATCTCAAAAAATATACCAAACGTGTATTTCTCAGCAAAGTTACCACTGGGCACACTACACACTATATAACGAGACGGAAGTCGGAATCGTTTCTTTTCCGTTTTACACGTCTGAAGAGAACAGACATGGTGAGCATCTCGATCGGATAAAATAAATTATATTACTATGTGATGTGTCAAAAAGATGGATGA

>ID_29 P_29 Promoter_29 D1_Promoter for RE03872, -250bp and +50bp [0 ]

TGCAAGATTCCCACCACGCCCCACTGATAAGTTTTAATTGCCCGTTCGATAAGAAGAGATGTGCTGTTCTATTCACACTCGAAAGGGTGGAGGAGAGGGAGCAAGCACGCCGATCGCATGCTGCTGATAAGAGGGTATCAGTACCAGGTAGCACCCATATCGATGCCGATGCCAACGCAGACGCTGGCAGAGCGATCGGTTGCCCCGCCGGCAGAGGCTTCTCTATAGCATCCGATCCTGAACGGCGTTCAGTTCACGCTTTGCCTTCGCTGAATTCGGTCGTGTTTGCTGTCGTGGTTCTCGAGCGAAAGAAAGAGTGGGAGTATAGAAAATAGACGGCAATCGATTTG

>ID_30 P_30 Promoter_30 D1_Promoter for GH19633, -250bp and +50bp [1 ]

CGTATCCATATGTGATTCATTCATAAGCCGGGTGCCCTCGAAACGCGTGAGGCACTTGTGAAATGGCCTCTCTTATCAGGCGGTTAGTTCCCGAATCGATTTCGATGGCGAGCCGTGGAAATTCACGGAAGAGCAGCCTTGCTGGCCCGCGTTCCTATCAGCAGCACTCGCCGCATAATTGAATAATCTGAATCTGCCGAGCGAAGAGCGCGAAACAATAAAAATATCAGATACGCTGCCCGACCGCCGCCAGTTACAGAACAAATTCGCTGCGAACACAACGTGGGCGCATCTCGCAGGTGCCAAAAACGAGGTAGAAAGACAAAGACAAGCAAATGTGCGGGGGGTTT

>ID_31 P_31 Promoter_32 D1_Promoter for RH07045, -250bp and +50bp [8 ]

TTTAGTATGAGATTTAACTTTGCGCTTAAAAGTTTTTGTAGAAATTGAGACAACTGGACTAAGTTTGTTTTTAATTTACTCAAAATCGGCCGTGACCAATTTTAGATTGAATCCAAACCCCGACATCTTGACACATATGATTAACGTACGCCTTTCAGCTTGGCTCGTTATCGCCTAGCGTGTTATCATTCCGAGTGATTTACATTTTTATTCCAGCACCACTGTCTCTGACAGGGAAAACAAATGGGAAACCGATTAGTTGGGATCTAGTGTGTCTAAGCCGCAGTCTGGAAGCAGAAGCACGGAAGTGGGCCGAGGTATTAAGAGCGACCTTTTGCTGTTCCGACCTC

>ID_35 P_35 Promoter_36 D1_Promoter for RH02953, -250bp and +50bp [7 ]

CGATTTCAAATTCGAAAAGAATTCAAAAATATTCCTTCTATGTTAAAACAAAAGAAATTAGATAAAAACCGATCGATTTTGAGCCAAAAGCAATACAATATGTGTATTTAATTTACGTAGCCGAGTGCTGCCTTCAGATTCTGCCATCCAAAAACAAGTCTCATTTTTCTTCGTGTGTAAAACTTGTGCTCTGCACCGAGCGCAAACCGGAGAGCACTGAAAAGAGACCTCGGTGCAGTGCCTCCCAATCCGAATTATTTAGCAAAAGCAGGCAGAGGCGACAAGACGTCGCAAGAAGAATTCGCAAAAAAGTCCCAGGAGAAAGACTGATTCGTGTGAAGTCGTCTACT

>ID_36 P_36 Promoter_37 D1_Promoter for RH57538, -250bp and +50bp [2 ]

CATGCCCCCCATTCCGTAGACCCCCCCCCCCCACCCACAACCAACTCATCGTAGCTGCGCCGCCTGTGAAAAACCAAAACAATCGCCGAGCCTCCGCCGGCAGCCAATCAGCTCGTCCGGAGTTTGACCCTCTCACTCGGCGAGGCGATCGTAAAACCCGATTTGCGTGGCGATCGGCCGACTTGTTCTAGTCGCCTCGATGGCCAGGCGTCGCCGGCTGGGTATAAAAGGCCCCGGATCGGAGCAGTTGGCATCAGTTACTCGTGGCCAGAGTAAATGGAAAGATCAAGTGAAAAGCCTACTTTCAGTGGTCTGAAAATCAAGTTGATGTTTGACAAAAAACTGTGGAA

>ID_37 P_37 Promoter_38 D1_Promoter for RH08125, -250bp and +50bp []

CGAGGACAAACAGTGTTTTGGCACTTCCTTTGATGAATTTCCAAATTGCAGGTTTTGGTGTTTTGCGCAGCATTTTAAGTGGGAGTTGTAAATATTAAACACGTTTGCTTTCTTACAAAAAAAAAAGGAAAACAAAATCACCTACCAGCTTATCGATAAAACGAATTATCGAACTGGGCCAGTGCTGGCTAACATGCGGCCAACAGCTGGTCGACCGGCTAGTGTGTGTGCGCCTCTTCTTCTTGCCATGTAGTGCAGTGTTTATCTCACGTTTTTGTTTTTGCAAACTGATAACGCGACGCAATTTAGTCTGTAGTGAAAATTGTTTTTTTTTACATCGATGGAAAATC

>ID_39 P_39 Promoter_40 D1_Promoter for RH68512, -250bp and +50bp [3 4 ]

ATTCTTGTGTCACCCCATTTTATTTTTAACATTTACATTTACTGTACCCTGAAATTTTAACCCTGACACGGTGTCTTAACGTCATTATTTTATAACCAACCCTTTGCCCGTGATTGTGCGAAATACTTGCTGATATATTTTTACAGCAGACTGGGCAGAAAATCGTCTTTGCGGGCACGCAACCACAAAGTCTAATTGAATTTGGTTGGCAGCACAAAATGCGATATAAACGAGGCATTGCGGTGATTGGAAACAGTTCAGCCCTGTGGCGCACACAGAGCGAAACGGGTAGCGGTTAAGGCGTATCGGTTGGGAATCGGTTCGGTATCGGGTTGACCGATATGGAGCCG

>ID_40 P_40 Promoter_41 D1_Promoter for RE71584, -250bp and +50bp []

TGCAAATCTAGACTAGTGATGTGCTGCCACCTATCGACAGCAAAATGCCAATTTGACCATTGTAAAGTTAAAATTTTATATAGAATATATTTTAATTTGTTCATATTAACAATTTAATTTTTAATTAATAAATTAACAAGAAATTTAACAAGTTGTTTAATGTTAAGAATTAACTTATTTATTAATAAGGTTAACCGCTTTAGTTACGGTTAAAAGTGGCAACGCTGTGCTTGTTGATTCTGGCAACGCTGTGTCATCTGTGGATTGGTTCGGTTTTTTTGTTTTTATTTATTTTTGGCATGGACTCGGCGGAATCCCCCACCAAAACGCAGGCCGGCGAGGACGAGAAC

>ID_42 P_42 Promoter_43 D1_Promoter for RH62628, -250bp and +50bp [4 ]

TTCAAGTTCTTTTTTACCTTTCGGAAAATACTTTGTAATATGCAAAACAAGTTCTGTGCCAACAAATGCAGACTAAATGAGACTGTTAACACCCGCTGCATATGACATGATTAATGGTATTTTAGTCTATCTAGTTTGCTTGTGGGTTGCAAGCTCAAGATCAAGATCATCTGTTCCTCTGCCATGGCTGCAACATGACTTGGCTAAACCCAATCTAGTTACTATATAAGCTGGGCTTCGGTTGGGTTTAAGGCATCAGTTACTTGGAAGTCACTACCAGCTACGGATATCCAGTTCATCATGCGCTTCGCTCTACTTGCCGTTCTCCTCATCGGAGTGATCTTTGCCTT

>ID_45 P_45 Promoter_46 D1_Promoter for RH19893, -250bp and +50bp [1 ]

CACGTAGATACCGTAGTTTATGTTCTAAAAAAAAATATTTCGAATCTTAAAACTATTTTTTCACTTATTTTGCCTTTCTAAGCAATTTTCTTAAATATTGTAAACCCCGAGTGAGGTTCTGGTTATTGATAGTTCTGATGATGGGCCAGTCAAGTCTTGGATCGATGGCATAGAGAGCCTATTGTATTTTTCCATTGTTGATCTCCCACTGAGATGGCAGGTATAAAATGGGCCACATCGCCGGGTGTTCATCAGTTCTCTCAGAGTCGTCGAGCGAGCAACATGAACGTAGTAAAATCTTTGTGTATTTTGGGTCTGGTTCTCGTCAGCTTGATTGCCACCCAAGCAGC

>ID_47 P_47 Promoter_48 D1_Promoter for RE47058, -250bp and +50bp [2 ]

CACTTAGTGTTGTAGCCGTTTGTAAAGATACATATTCTAAAGATCATTAACAGTACTTCAATGGCCTATACACAATAAATGAGAATTCAACAACGATGTTTTGACTGTATTAATTTGCTGCATCTAGTGTATCTAATTTTAACATTTTTTCAACTCAACCGGTTGCTAAACCGTTTCAGCAATGTTATTTAACAGGGTTTGAGTGCTCGCTAAATGGACTTACGAAATGAGTATGGAATTTTCGTGCAGCCCAGTCATGGTCACGCTGCCTCTCTGAAAAGCCGCAAGAAAATAAAAATAAATATATCTTTCTAGCTATTATTATTGTTGTTATTATTAACAAAAATGTC

>ID_49 P_49 Promoter_50 D1_Promoter for RH48785, -250bp and +50bp []

CATACAAGTGCGGCCACTAAAATAGGTATTCTATTGAATTTACTAAGTTTTAGTTATGAAATAACTTATAATTAGTTCTATAACTTTAATCAATAATTAGATAAATATGTACTTCCTAATTTATTTAATTTAAATGCATTTATTAGACATTTAAATTGTATTTGAGCTATATATTTTTCTAGTATTTTTTCTTTGGTTACTCTTTGGTATTTCCGCAGCCTGGTCACACTGGCTCAAAATCAATAAAAAATAGACAACATTTTAGGATCGGAATTCATTCATAAAGGACACAATGCCAAAGAAAATGGGAATCAACTCGAAGGCGGTGGAGGCCCGCGAGCGCAAGGAGG

>ID_50 P_50 Promoter_51 D1_Promoter for RH03284, -250bp and +50bp []

TTACATAAATCTACATTGGTTTTATTTTAAACAATTTAAGGTGTTTTGCTTCCGATTCTCTAAATTTGCTTAGCGTCCAAAGATATAGTGTCACCTCTCTTAATTTCGATTTATCTTGACAATTCCCTACGGTAGTATAAACTGACATTTATAGTGCCTTTGAACTCACAAGTCTACTTTTGCATAAAATTTCTAATTTACATTATAATTATCACCTTAAATCACTATATAAAGCAGATCTTGAAAGGCACAACATCAGTTGTAAAAAGCATTTGCTATGGTGCGACAATTGGTAAAGGCCTATATAAAATACTTAGTCACGAACAATTCTTAGTCTTACTGACCTATTG

>ID_51 P_51 Promoter_52 D1_Promoter for RE31839, -250bp and +50bp [4 ]

AGCTTTTGCTTATATTCTAAAAATATGCTACAAAAATATTATAAAACTATATAAGCAATTAAATTTGCAAAAATTAAATTCATGTTATGTAGTTTTGAAAATACAAATCAGACAAATTTTAAATATTATCTGCTATGTGTTAGCGTACGAAATGCTTATCGATAGTTGCCCTGCCAACTCGCACAACACAGAATGCAACCCAGTGTTTTCGGCAAGCGCAGCCAACTTCGCGCTATGTCGGGCTGCCATATTTCTTTCCTTCTTGTATTTCAACGAGAAAAGGTGGGCATTTTGCGTTTGTTCTCGATTTGGTTAAGTTCTAGCGTTCTCTCAGCGTCCCAAGCGCTCCG

>ID_52 P_52 Promoter_53 D1_Promoter for RH56522, -250bp and +50bp []

CTTTCATTCCAAATAATTGTACAGATGCATATCTGTGGTTTAAAAGGGTTATGTTCTATGTATCTTTGAAATTTATTGAATTCTTTTTAGCTAGAAATATTTAATTAGGTTGCACAACTCAAAAGTATGCAATGAAATTAGGTTCATTTACATTAAATTTTTCATATAATAATCTATAATTTTTACCAATAAAAGCACCGTCTTATCAGCGCTTCTTTCGTTTGATGTTCATCAAAACAAAGCGTCGTCCCAGATTAGTTGTGAATCTAACTTGAAGCTAAGCGTTACTACCGTGCATGATGTGGATTCAAAAGAACCCATTTTTAGGGTTATTGCTCTGCAGTTTTTTG

>ID_53 P_53 Promoter_54 D1_Promoter for RH45210, -250bp and +50bp [3 ]

ATGAAGTGTTAATAGCTGGCACTTTCACAATTTGTACGAACCTCCTGCCACCCGCACTATTTGCATATACATATGTAAATACACACATACATACATATATAGAGCTATTACCGATTTCACAGCGTCCCCAAAAGTTCTCTCTGTTCTCCCAAAGCTCCCATGATCACTACATGCGTTGATATCGCGGCAGAGGAATCGACGGCGACTGCGGCAGAGCAGCTTATAAATACCAGATGCTCGACGTTCTTGGGCAGTTAGTTTTAAACTGCGACTTGAAGCGGCAACATCGTCGGATCAAGCAACATATAACAACTTTCGTGAGTGCCACAAGTGCCCCTTTTTTACGATTA

>ID_55 P_55 Promoter_56 D1_Promoter for RH33889, -250bp and +50bp [1 ]

ACATTTATGGTGGCTTTGAGGGATGTCAATATGTGCATTTCCTACCAAAAATATATACCTCAAGCTCTTTATGAGAAGAAGTAGCCTGCATCCCACACCAATTGGTAGATTAAGTACTGTTACTTTCGATAAGCATCTGTTCTGTTTTCTCCCTGTGCATGTGGGACTCGCCCCTATGAAAGACCGTAGGGTTCCCATACTATTGGTATTGCTGTGAATCAGGCGGCGTTATTCTCGCTCTGCTTCGCTCATTCACTTTCCATCAGTTGGCCGTGTCGGTTCGTTTTTTCCCCAATTCTCCAGTTCTTTCTCGCTGCCCGGATTCGATTCTCAGTTCTCTCGACGGTCGT

>ID_58 P_58 Promoter_59 D1_Promoter for RH44916, -250bp and +50bp [8 ]

TTTTTATAGTTTTAACAGAGAAGAGCATATGGTTTCTTCAACCCGATTTAAACTAACCCATGTTTCGTATCTTGAGTATGCATAAACTGTGAATACAAATTAAAATTTAAACAATGTATTGTCTTATTTTTACTAAGGGTTTTCTTTTCAACTGAGGTTTATTTTGCCCCTAACAGCACACTTTCGAGCAACGGCGCGCTGTTTCACTTAACATATCGCGTTTTTGTGGCGTCTAGAGGCAGCCACACTATTTCCTTCTTTTCGCTTTCGTTTCCGGCGAAGTAAGTAAATTAAATTTCTCTGTATATTTTCCTTTAATAATATTCTGTTTTTGTTTTTAGCGGTGTTAA

>ID_59 P_59 Promoter_60 D1_Promoter for RE66546, -250bp and +50bp []

CTTAGTTTGTAAGATCATCACAATTTTTACGTGTAACAGCCATTCCGTCCCAAACGAATCTCAAATGTATTGTGCAAATAATTAAAGCAGTTAAATAAATGCCTTGAACTAAATGTTTTCTTATTATTTATTACACCCTTTAATTGTTTGTTGAGTTCAAAATATTTTACTGGGCTTCCCTTATTATTGTGGGACAATATCTTGACCGCTACTGTTTACCGATAACAAATCGATGACAGCTGACTGCACTAGCCCAGTGCTGTGTAATTTTTTAGACAAATCGGCCCAAACCGAACGGAGCGATAGAGTTTTTATTTTGAGCTGAAAATTCTCAATATTTACACATCCCA

>ID_60 P_60 Promoter_61 D1_Promoter for RH28024, -250bp and +50bp [2 ]

CTTTCTTGAGCAAGGTTCCACGCCGATCATCAAAGCTGAATATAAAAAATATTCTATATAGGGCGATGATGTCGCAATTAACTTTCATCACTCGCAGCTTTCTGCACCAATTGCGAGCTCCAATTCTACGTGTACTATCAGTGGCATTCTCTCTAGGTCAGAAATCAATCAGTTCAAGACTGAGTTATACATAAGTGGCTCTTATCACCGCCGTCTGAGGCTTTAAAGCGGAGCTCCCTTCGCTCGATTGCATTAGTACAATTTAAATCGCGAGTGTCGAACGTATTTTTCGGTCAAGAGCTAAAACGAATTGCCAAAATGAATAAGCTGCTGATTTTGGCCCTTTGCCT

>ID_63 P_63 Promoter_64 D1_Promoter for RE71324, -250bp and +50bp []

CATAAAATACGCATAGTCGGAGGTAGTAGAGCGTAATATTTAGATAGATTATTTTAGATTGGTCAGTACTCCAACATAAGCTTGACTAGTTTGAAGCGAATTTTTTTTTGAGCACGAATTATATAAGAGTGCAGTTCATATTGTATAAGAGCTCGGAGATAACATGTTGACCTACCCCAGCACCCTGGACATTGTATAAACAAATCCCGCTGATAAGGTGGCCGCCACCGATCGGGAGCAATTGAAAATTAGTTGAGTTTAATTAGGCGCGTGATGATGACGAGCCGGGTGTTGTGGCTCTGCCTTTTGGTGGCCATAGTTCCAGCTATGGCCAAGCCATCGGATGCTGG

>ID_64 P_64 Promoter_65 D1_Promoter for RH52788, -250bp and +50bp [8 ]

TAGAACTAGTTCTTGAATTCAACCGCTTTGAAGGCGTTCAACCTAGCCGTTTGATCCGATTCGATACGATACGATCCGTTCAGATCCGAGGCATGTCATTACAGCTGATCATATGCATGCGGAGCAGTGTCAGCCGTGTCCACCATGTCATGGTGTGTGAACTCTCACTTTCATGAACCCTCCGACTTGGCGGTTTTCTATTGCTGATGTGGTCAGCAGTTGGGTATAAAACTGGCCGGCAGCTCCGTTGACAGATCAGTTCAGAACAAGAGTTCAACCAGAGCGCAACCCAAAACCCACAAACAGCACCATGAAAGTGAGTTATCAAACGGATTTATCCTGATAATTAA

>ID_65 P_65 Promoter_66 D1_Promoter for RH06344, -250bp and +50bp []

GGCCCAGGGAACTTGGCATGCTTCTATTATAATTAAAGAGTTTTACTGCAATGGATATATTCTAAACTATAATGGTTATATTAAACAAATAACAAAGCTAGCAATTTCAAAAACAAAACACTTAACAGACATTTTCGTTAAATATAAATATTTGCTTGGCTGGAACTTTCGTTAAAACTGATAGGTTCGTGCATTCGCTCAGCGGTGGTAAGAACACTCGCTAAACGTGTGAGCAGCTCTGTTTGTGTTCCATCTCTAGTTCAACAGCTGTTCAAATCGAGTAAATCGTAATTGCTCCTTGAATTTTTTTTATTTTTGTGGGCAACTCCTGCATTGGAGTCGGCGGTTGC

>ID_66 P_66 Promoter_67 D1_Promoter for RH72196, -250bp and +50bp []

TAAAAATAAAGTTTTTTTTTATTTAGACAAAGTTGAAACATTGAAATAATTTTAATTTTAATTTTAGTAAGCAAGTAAACTTATAAGATTTGAATATCATACCAATTAGGTATAATTGAAAATTAAGTATAATTAAGTTAATTTAAAATGATAAATAATAAATATAAAATAAAATTATAAATATGTGTTAGCTTATATTTATTAGTATCTTTTCAGAATAGCGGTATTTTTAGCCGCCCAGCATACGGCCACTCTGCATTTAACTTGCTTGTCATCGCATTCGGCAACTCACAAACAACTTGTAATTTTCGTTAAAAAACCCCGAAAGTATAAATATAATTGCAACATGG

>ID_67 P_67 Promoter_68 D1_Promoter for RE21802, -250bp and +50bp [2 ]

AAGCACAAGTAAACTTATGCCCGCACAACAATTATAATGTAAGAAGACGTCAAACCTGTTGAAAAATGCTGAAATTCCAGTGCGATAATAACATAGCCCTCGAGCTAAATTCAAATTACGCGCCCTGCCCGCTGGCGCCCCCTACCGCCTGCGGTGCTAAGCTACGACGCCCATTTCAACCGCCCGGGGGCGCTAGTCGGCATGCAACCCTGCGCGTCAACAGCTGACCGGCCTATACCCATCTCGCTCGCTCTCTTTCCTTCCACGCCGTCAGCCGCAAAAAACAGCGGACGCTTAAATTTTCGTAAATATTTGTAAATAACAGGGGCTTAAGTTCATTTGGCAATAAC

>ID_68 P_68 Promoter_69 D1_Promoter for RH22327, -250bp and +50bp [7 ]

AGAGCGCCAACGGCTTGGCGCCGACATTTCGATACCTTGGCGGCTGTACTTGGCTTTGCATCTGGTAATCCGATGCTCAGCCGATGCCACATCATTAAATTTTTAATTTGTGAGACAAACAAAAAGGAAACGTGCAAAAGCCGTGCCGCAGAACGAGAGCGAATCAAATGAGAGCGAGAGGCCATTAACTTGCATCTGGCAATGCCGTAGCCGCAACGCGAAAGTATATAACAGCCGGCACGCCGCAGAGCTTTTCATTCGCTCAGAAAATTTCAACAGTGAACATCTTGTGCAGAACACAGACCAAATATTCCAAAAATGTCTTTCGTTGGCAAGAAGTACAAGCTGGA

>ID_69 P_69 Promoter_70 D1_Promoter for RH01814, -250bp and +50bp [8 ]

CTAATGATGTTATTAGCGGCGTTGCCACACTTGTATATCAAGCAAATAAGAGCTTTCACATATAATTTTGTTTTAATTTGATTTAGAGGTTTGGAAGTTTTACCCACAGTATTATTAATGCTAGAAATTTTGTTTATAAACTTAGGATATCGCTTAGAAAATAAATGAAGCTTGCGCCTTAACAGCACGCTGTTATCATAACAGCACAATTGGATATTTTAGCGTCTTGCTTGTGATCGGTGCAGCCACACTATTTTCTTTCTTTTGGCTTTCGTTTCCGGCGAGGTACGTGCAAGAAATTGATGCTCCACGTTTTTAGCTTAAATTAATGTCCAATTCTCTATTTTAGC

>ID_71 P_71 Promoter_72 D1_Promoter for RH06410, -250bp and +50bp []

GGATTAGTGTTGCTACATACGACGACCCGTCGCCCATCCGTTCTATATGCCATGGTGCCACCACCCACCTCCCACCAGCCACCTCGCAGCCATCGCAACCGATCCGATGCCCATCCAATCCCCGTCGAATCACACGGATAGTGTACATGAGCCGTAGACCCGATCGTCGAGATCGTGCCAAGCCGAACGATCGACGAGTGAAGATCGGGGCATCGGCGTTTGGCTTTTGGATCGCTCGAACTTATACTATAATTAGTCGTGTTCCGTCTCCCCGAGTAAGTAGTAGTCTGGTCTCGGTCCGGAGCTGATCTGGCCAGCTGGTTTTAGTCTTTAGTCAGCGCTGTGGAGTG

>ID_73 P_73 Promoter_74 D1_Promoter for RE68692, -250bp and +50bp [4 ]

CTGTTGGGGAATTTTAAGTGCTAAGTTATAAAAAAAGATTTATTTATAGTATCTTAATATTTTATATTTTTATTTATTTATTTATCATTATTTTTTTTTATTTTCAGAACTTATATGAACACATCGTAAAAGCTAAGTTCAGCTATTTCGTATTCGCTTACGTTATGGCAATCGATATTTTCCCACCTAACGCCGTAAACTCAAGTCGATTGCACGCGTTAGCTCCACTTTGTGGACGCTTTAGGACATTGCTCATACGCTCGCTCCATTTGCCAGTCGCCATTATCGATTTGTGGCAGAGGAAAAATAGAGAATTAAAAAATCGCGTTTCAGCGTTAGGCACCGGTTAG

>ID_74 P_74 Promoter_75 D1_Promoter for RH33067, -250bp and +50bp []

AAACATATGTATTATTTTATTAAACTAACGCATAAATGTGTTTCAGTTTTTTTAAGTATAGAACGTGTCTAAAAGCCGAAAATGTTGTAATGTTGGATATGGTCCACGAGTACCTGGTATAAATTCCCGAAACGGCACACGAACCGATCCACGCGCACACATGTGTGAGTGCGCGGACTTCGGGTCGAGCCGATCGTCTGGATGGACAGTTAGTTCGCATATAACGGCGCTTGCTTGCCGACTTCGGTGTTAGTCGCAAGTTGAGAACCACTCGGCATAGAATTAGAATTCCAGAGCCAGAGGAGAGCCGAGAAGAATCGTCCCGATCCGTTCCACTCCGCACTTCGAAT

>ID_75 P_75 Promoter_76 D1_Promoter for RE53135, -250bp and +50bp []

ACTGCTCGGAGTCAGCTGTGATAAAATCGCGGTCCGCTCGCCCAGACGTCCCGCTCCCTCGCACGACCCTCGCGCTCACTACCCATCCCGCTTCCACCGCCTCTGACCGGCGGCGTCTCGCTCGCGCACAGCGCCCGCTCCCGCTCACACTCTCGCAAGCTAGCTTAAAGCTGCACTTAAGCACTGAGCAATTGTTTGGGGGAGCAGACGAGTCGAGTCGGCGGCCTCTGGGCTCCACTTTTCATTTTCAGTTGCCTCCCAGACGTGCGCTCTCGCTGTCGTGTGTTCTCGATCGCGAGAAATCCAAATCGAAATCGCAGTCGAAATCGAACTTGGTTAATGCTTAACAC

>ID_76 P_76 Promoter_77 D1_Promoter for RH26328, -250bp and +50bp [1 ]

CATTAAAATTAAGTTAAAAACCACTACTTTGAATTGAAGTTTTTTGCCATGCCCATGAAGACCACTAAAATATGACGTCGGACATCGATTTTCAAGAAACGAGACCGTGTTCTTGTGTGGTTATCACGATCCGTAGGGCCAGCATTCGAGCAGAAATCGTCCCCAAAAAACAGTTTCCCTGCTGGCTGAGACAAGAAAAACGCGTAGATTTGCGAGCAGAGCTATAAAAGGGCGCGCAGACAACGCGCTCTACCCAGTTAAAAAGAGTGCGTTGTGTAGAACAGTCATAAGCAGTCCGCTAAGAGATTAGTAGCAACTACGAAGTGAAGGTGTGTGTATACCAAATTCTC

>ID_77 P_77 Promoter_78 D1_Promoter for RH72195, -250bp and +50bp []

AGATTTCACATTCTCAGTTTCCTGCCACATTGGTTTTTCATACGGACGGCATACTCGTACTGTGTCGATGGTTATGAGTAAACACCCGACTGATTACCGATACACGAATAAGGCGGGCGATTGGCTTTGTGGGGCGCCCTAATCTCAGACATTGACCCCTCCGTCGCCCCAGCGGCGCAGCACACTACACCACCCCACATCGTGGAGATAAGAAACCGCGGCTGCGAGCTCCGACTCTCAGCACTTTGCAGCCAGTTAGTGCGAGAATGTCGAGCTGGTCGCTTTGTCCGCTTTTACTGGCCTTCGCGGTTCACGCCGTGCTTGGATCTGTGGATTTGGCTGGTTCCCTG

>ID_78 P_78 Promoter_79 D1_Promoter for RE15249, -250bp and +50bp [6 ]

TTGTAATCGAATCGTTATCAATAGTGCCTTTTTCTATATTTATATATATTTTTACATATGCACAGTAATCAATGGATTGATTATTATTGTTTATTATTGTTTATTATTATTTTGTTTATCTCGAACCTCAGCTGAAATAATCGCAAAAGGGTCGCAACCTGCCAGCAATCACAAATCGTGCGATAAGAAGGTTATCTGCAGCATCAGCACCTTTCTGGGGGCTATAAAAAAGTGATCGCCACCGAATGGGGCGATCAGTTTGACTTTTGAATTCGTCGCGATGTTTTCGATTAACGCCGTGATCCTGGGCATTTTGGTTACTTCCGTTATGGCCCAGGATGCAAGGGGAA

>ID_80 P_80 Promoter_81 D1_Promoter for RH06583, -250bp and +50bp [9 ]

TAACATGTTTAACCAACTTACCTAGTAATTAGCAATCGGAAATAGTTGAAAAACACAACTTTTTTGCTAAAAAAAAAAATCCGCGCAATTTGCTGTTGCTCTGCTAGTGGTGGCACTTCTTCGATAGCCCACTCCGCGGCCAGTGTGACCGTTTGGCCGATGGCGCGCGTGTCACGTGTGCACGCATTTCGCAGCACTGAAAGCAGGGTTGTACCGATTTGACAGCCCGAAGTTGGCGTATAGCACGTTTCCGGTCTTTCTTTCCCTTTCTTTTACCAGCGTGTGCAGTGACTAGTAAAAGGTTAGTGCGATATTTTCAATAAAAATACCATCCAAAGTGAAAGGAAACC

>ID_81 P_81 Promoter_82 D1_Promoter for RE39031, -250bp and +50bp []

GGTGACGTCACAAAGAGTTTCCGCCGGGCATCCGCGGTTTGCATCTAAATTTGCTTTTCCCGCTCCTTCCCTGCGTCACCATGGTTTTATTTCGGGCGATTTTGGCATCGGGCAAATCTATATCTGCCCGCTGACGTCGACGTCGACAGAAGCGGCAGCGGCAGCGGCATTAGCCTAACCTAAAATCGTTTTCAGCATTTTCGTAAATTATCTTAGGCCAAATGGAAAACTGTTTCAACAAACCTGCGTCAGTTGAAATTTGTGTCTCAAACGCGCCACACGCTAATTTGCATTTGCATTTCACTTTCAAATCTAATAATTCACAATGGCTGTAAGTTCCACAAGCTTCG

>ID_82 P_82 Promoter_83 D1_Promoter for RH13368, -250bp and +50bp [9 ]

ATAAACGTAAATTTGTCATAGCTTTATGAGTAGACTATTGACTAATGCTTATTGGGTATTTTGATACTCGATCGATATTAAAATAGGTGATTTTACAATTTAGTGTATTGAGATTAACATAACTTACTTAAGATTTTTTATTATTCAAATATACACTACGATCTGGCAACAACAGTTGGACGAAAAATATCGATAGTCTTTAAATATCGATAGTATCGAATCATCGCCATTTCTTCCATCTCTGAAACGGCCATCTTCCTTTCTAATAAATCGCGTTCGCAAAACGACGTGTTTAAAACCTTTTCAAGTGTTTTAAAAGGTAAATTACTCTCAGAACTGCCTAAAAATGC

>ID_84 P_84 Promoter_85 D1_Promoter for RE43155, -250bp and +50bp []

GAGAGCGGATCGAGAGAACGGTATCCGTATCCGTAAGATAGTATTAAACATTTACCAATTAAGGTCTGAAACCGCCGGAGAAAAGCCAGGTAGAAGCCAAGCAAAGCTTACCGCGAGAGAAAGATAGAGGGAGGCCGAACGGAGAGCGGATGAGAATCGAGCGGGCGAGAGAGAGGGAGAGAGAGCGCCACAGTGGGGCTGTTGGCAAAGGCAAATTATACGGTAATGGTTAGAAGCAAATGGCGATGGCGCTCAGTCGACGAGCAACTTTGGCAGCGTGAGGACGCCCGTGTGGATGCGTGGCCCCGTAAAAACAACAACAAACAACCGAGTGGCAATGTGCAAAAATA

>ID_85 P_85 Promoter_86 D1_Promoter for RH01650, -250bp and +50bp [6 ]

TAGATGTGCATGTCCTAGGCTAAAAATCCGTGTCCTCGATGCATGTTTCCCACTGTTTCATTGTCTCTGCGAGACGCAAAAAAAAACCCTGATTTCCCTTTAACGTCTAACGGAAAAGAAATTTCAAATAAATGTATTTTTATACCGGACTCGAGAGGTCTTTAACCCAGTCGAGGGTGGGCTCGTTACGAGGCCCGGTCCACTTGTGCGGCTTCTGATGGCTCTGGGTTTATTGGATTTGCCAGCGAAATGGCCACAGTTGTGTTCTAACACTTGGCGGCACCAGACCGAGACGGTTCAAGCGGAAGGGGCTCAGGATCCTGTTAGAAGGACGAAACTATCGATGCTAG

>ID_86 P_86 Promoter_87 D1_Promoter for RH10682, -250bp and +50bp []

ATTCAAACTCAGAGCACACTTGTTCAGTTCCAATGTACCCGGTGTTTTTGGGGTCTGTTTGTTTAGAATGAGCGTATGAAAACTAATGGTTTCTATCAACAAATGCGAATCTGCAAAAGAAGCGGAAACTAAGCTAAATGGGAGGGAAAAGTAGAGAGCAGTTTTTCCCTCTCTGAGGTTCTCCATGTAATCCATTCGTCGGTCCCGAATGATGCACATCGTCCATCCAATAATCGACTCGGCGAAATTTGCATTCAGTTGCTGTTCGTGCGTCCACCGAGTAAGTTCTTAGTTCGGATTTCCCTCTAAAAGCATATATTTCGAGATTGTTTCATTTACGATCCATCAAT

>ID_87 P_87 Promoter_88 D1_Promoter for RH67373, -250bp and +50bp [3 ]

CCTAACCCGTAGTGCTTCTCAAGACAGTCCCCACTTATAATACTGGATCCGCTCTCCGACAGAGAGATATCTGGTCGGGCCAGATATGGGTAGAGCGGCGAGATATAGCCGGCCATACACAAAACACAGATACTCTATGCATATGTGTATGTATGTGTTGTGGAGAGCGAGAGCGGATCGGGTTTGTGGTATGTGGTATGCCGCAGCAGCGGTCGCAAGTGCAAACGATATTCGCACACGGTGATTGGATCAGTTGACATTTGACGGCGCGCGTGTTGGCACGTTTTCGGGGCCTCTTCCACGGACTCATGGTTAATCCATTGGGTCGTCGAAAGTGCCGTACGCAAAAT

>ID_90 P_90 Promoter_91 D1_Promoter for RH57682, -250bp and +50bp [3 ]

TGGACCACGAAAATTTTGCGGTTTTTAAATTTTTATTTTGTGTTTCCTTTATGATGATTTTTTTGATTTGTTTTATTTTATAATTTTTGTAATAAGGAAAAGTGTATTGGATATGGCTTCTGGTTCATCCCAGTTTATGTTCCATTCCACGCGTTCCAAAAATAAACGCGTAGTTCAAAATATAAACAAAAGTAAACTCCGAATGCAGCCCTGTGCTCAATTTTTCAACTTCCTGTCACGCTGTGTGTGTGTTCTTTCTTTCCTTTGATTCTGTTGCCGAAGCAAGGTAAGTTTTTGGGGTTGTTACATTTTAAGCATCGGTTAAGTGAAAATAAACATACAAATACATA

>ID_92 P_92 Promoter_93 D1_Promoter for RE45373, -250bp and +50bp []

TTTTAATATCTAAGCAAAAATTATCCAGATTGCAAATATGTACATACATACATATGTATTTATGTATATACTAGTATACTTTTTATGAGTCTGGCGATTTTGCGGTGATACTCATTTTCGGAACGCCTCGCTCTCTCCGCTCATTTCTCTTGGCTTTGCTTTTGCTTTAGCTTCGTTTGCTTTTCGGCGTTCGTTCAGTTCAGCGTTCGGCGTTCCGTTCAGTTCATTTCGGTTTGGTTCATCTTACTTCAGTTCGGAATCAAACGCGAGCCGAGTCGCAATCGCAACTTTGCCGCCGCGCAGCGCACGCTGTTTTTTTTTCAGTGTGTACGTTTTGATTGCAAAAAGAA

>ID_93 P_93 Promoter_94 D1_Promoter for RH61764, -250bp and +50bp [2 ]

ATCTAACTATGTCGGTGGGACACGGAGAGACTGGTCGGCGGGACTAGTACTAGACGACGGGAAAGAAAATTAATAAATGAAAAGGCTAATTTGACGCAATTCCCGGAATAATAGTTTTTACTTGGAAAATAGTTTGGGATTATTGCAAGTTTTAGTTTTACATAACTATTATATTGAATACAAATATCTTTACTGAACCGCTCCTGTTCAAAGGCAATATTTTTTCGCGTGCTAGCGTGCGGTCACACTGCAGTCGCATACGGTTGTTTCCATATGAACGAACAAACTGTTTTTTTAGCGGAGTGCGCTGCTGTGAAATTTTGAAAGCTCTTTTTGGACCCTTTAATTTT

>ID_94 P_94 Promoter_95 D1_Promoter for RE56857, -250bp and +50bp [5 ]

ACCGCGTTCAGCTGATTATCGATAGCTAGGAAATTTAAGACATTTCCGAATATTAGGAGTACGGACCGACGACGAGATATATTTAAAGTTCGTTTCAGCAGAATCTTAAATATTAAAAACTTAGCGGGCAAGTAAATACACCGCGTTCAGCTGATTATCGATAGCAACCAGGTGGTCACTCTAATAGACGTTTTCATGCGAGCCCGCTGCGGTCACACCAACTTCTCGACCGATTTTTCCCCTCCATCTAGGGTCACTCTAATTTTGGACGAGGTTAGGTAAGACACGCGCGGATGCCGCGCTGCGTAGGATAAATCCGTCATTAGCATAACCGCATTGAAGCTAAGTCT

>ID_96 P_96 Promoter_97 D1_Promoter for RH21243, -250bp and +50bp []

ATGTTCTCTCTGCGCCCCAAAACCACTCGACAGCCCACTCGATTTGCCCACTCACTCAGCTGTGTTGCATGCTTTTGCCCGGAGCTTTTTGGCGAGCTTACAGTCTGCGCACAGTGGCGCAACCAAAGGGGTATCTTCGAAAGGCTTGAAACATAACCCCTACGGGGAATCCGGACTACGCCACTGGGTCACAGCATAAGCACACGGGCAGAGCGGCACGTTTCCATGTGATGTTTTTCGGGTTTCGGCATTCAGTGTTGTTTGCCTTTTCCGCGGAGCTGGAGTTTTCCCAGCAGTTGGCATATAGTATATATATATATCTCCTGATATTCCGGTAGAAATGTTCAATT

>ID_97 P_97 Promoter_98 D1_Promoter for RH02345, -250bp and +50bp [8 ]

GGTTGTCAGGCCCAATAACTATGAATTTTAATAACTAAAAATTCTTTTACATAAAAAAGAGCAAATATATTTTGTAAAAGTTCAGTAGAAAGTTATAGAAAAATTAAATATTTTTAGTAACTACACGTAAGCAACAATTAAATGCCCTTCTAGTAGTTTCTTTAGCTAAATCCGGCAACTCTGACGGAGAGAGAGCTTTGGTTACGGAACAGAAAGCTTGCGCGCCTATAAAAGCCGCTGCTCGACCGAAAAAATTCAATTCCGTTCGCAGCTCAGCGAAAAGTGAACAAAAAAAAGCAGAAGAGAAATTTGGAAAGCAGCCTGCAAGTTTGCAGAGAAACAAAAAGCAG

>ID_98 P_98 Promoter_99 D1_Promoter for RE12106, -250bp and +50bp []

CGCTAAATCGGGATATTTTTGGGTTTAAGAACTTCTTGCTATGAGTTTTTGAGTAGTTACGTAACACAAAGAAACATTATAAATAATCTGCAATGCTGTTTTGGATAAGGTTTTTTTTAAAGCTAAAGATCTAGCTATTATCGGTAGTATCTAGATAATTAGATACTGCCAGCATCACTACCACCCACAATCCTTCACTATCGACCGACGGGTATAATTTTGCAACACTGTCCAAGATGCTCCACCTCCAATTTCACAGAATAACAAAAAGGAAAATGGTCGCTTTGTGGAACGGATTCCTGTAAAAATTGAATATATGCTAGCGCACCATGGAGTTCAGTGCGGATCTG

>ID_99 P_99 Promoter_100 D1_Promoter for RE75103, -250bp and +50bp [2 ]

TAGGGGTCTGACTCTTACGCGGCGGAAGCGTGGCCATTTCGATTATAGCCTCCTATGTAAGCCCAGGTGACCACCGTTTAGCAGCTTTTGGTTTTTCCTGGCTGTTCAATATTTTTCAGTGATAATTTCAACAAAAATAGGATACAATTCGTTAATTGGCCACAAAAAAGTGTTGGCTAACGACAGCCTAAAGATAGTCAACTTCTACCGATAGCTGTAACACGACTGCACTATCGATAACCAGTACTGACCAGTAGAGATGAGAGCGATTCTCCTAGCGAGACAACAACAAAATTCACTGCGTAATTAATTGAATATTTTTTAGTAAAACCGAAGCGCCCCACAAAGAA

>ID_100 P_100 Promoter_101 D1_Promoter for LD20229, -250bp and +50bp []

TCGATGGTCGACCATTTGATTTATAAATAAGCAGTATTATCTTTAATTAGGCTGCTTTTAACTGCAAGTGCAGTTTATTCATACGATTACAAAGCATGACGATAGATGATTATGCTATTAGGTTTGATTAAACCATTTTTATGAAATATAAATTATTATATTATAAATATAAATTAATTATAAATTTACAAACTCTTATTCCATTCCAGAATCGATAGTTCTGGAATGATACTAGTGCTCAGCAACGCCAAAACAGTCAATAATAGATATTTGAAGTGTCAGCGGAATCGACGTCTGACAAAACAACTGCTTCACTTCATTAGAATTGTGTGATATATTCCACAGACCAG

>ID_103 P_103 Promoter_104 D1_Promoter for RH30510, -250bp and +50bp []

GTTCTTTCGGAAACATATTTAACTCAAGAAACCAGTAGTAATTGAACGAAACAAACTTAGTTTTTATGTAATTTTATAAGAACCACCCTTGCTATAAAAAAAAAGTCTTAAATATTATATTATAATTTTTTGTAATGCCTCAAATATTTTTCGTTCTATTTCATCATTCCGTATTGCATGAAATTGTGATAAGTGAACTTATCGATATAAACCCACCACCAGCGCGTATTCTTTTCCGGTCTGGTTCTTTCTTTCCGGTGTGGCTCGTTGAAAAGATTGGACGAAATGGTGGGAAATTTTTCTTATTTGTGTTTTTGATCAAATGTTTTGAATATATCGAGATACTAAAC

>ID_104 P_104 Promoter_105 D1_Promoter for RE24107, -250bp and +50bp []

AATTATTTAAAAAAGAATACAAAAATATAAATTTAAAGCTAACTTAATAAAAAATAACATAATTTTCCCATTATTCATGTGTCGATTAAAACTCAAATGAAAATTCTTACACTTCTCCGCACTCTTTTTTACCCCATAACTCTCTATCTCTCTTCCAGTTCTCCGCGCTCACGATTTGGTAACTGTGGCCCAATGCCGCCGTTCCCAGTTTAGCGCGCTCTCCTTGCCTTTTCTTTCGCCTTTTCGACTCATTTCGAATCGGAAATTTGACGGGTTCGGTCGTAATCACGGATACAAATTTGACGCCCTGTTGGAAAAACCACAACGTCCAATTTTCCTTTTATCGCGCC

>ID_105 P_105 Promoter_106 D1_Promoter for RE39272, -250bp and +50bp [1 ]

CAGCTGGAAAGGTGTGTGACTGCGCTAATTGGACACCGAAGATATGAACCTTATTTGACAAACTATACTTTCTTATAAGTATTAATTTTGAAAAGAAACCCAGTTAATGTCAGTTCCTGGTGCAAATATCCAATTAGGCTTTACTAAATGCTCTCAGAGTCGAATAATTTTTTTCTAGTATTTTCCTAATCTCACGCTTTTGGTATTTTTAGACAGCACTGCGGTCAGCTGGTGCGCATCACATGACCGTCATAAGGTTTTTTTTCTCACTGGAATAGGCGACGTCGAGCAAGGCAAAGCGAAAATTTTCACCACCATTTTTCGTTTGTAGTTTCAGTTCACTTTTCGGT

>ID_107 P_107 Promoter_108 D1_Promoter for RH09244, -250bp and +50bp [7 ]

TGTCAATACAGGCATGGAAATTCTCTGAGTGCACTGATGCACTGTATAGTCTAGTCGTTTCCCTTCACTTCCCTCATCACTTAACCACACTCATCACTTTGCGATAATGACGTACGTATGCTTTGGGAGTCCCAACAAAAAAAACACACATGCGCTGCCCCGTCGACGCGGCAGCACAGCAGCAGAGGACGGCGTCAAGAGCGGCTTGAAGCTGGAGCTCGATTTTAAAATGGCCAGCCCGCTCGGCGCACCAGTCACTCTCAGTTGGAACGGCGCGTGAAACGGTTATCGCTCGCTCCACCGGCGCTCTCTCGCCGCTCGCCCCACCAAAGCCCGCATTTAACAGTTGT

>ID_108 P_108 Promoter_109 D1_Promoter for RE31242, -250bp and +50bp [1 ]

CGTTCCGATTTGTTGTGCACTCGAAGAAACAACTCTCAATATTTAATAGTAAAAATAATTTTAAGATCAAGGAATACTTTAGAACCCTAGATTAAGACACTTAAATAGTGTGATGGCACTTACTGAGATCTCCAAGACTTCCAAAACTAAAGTTATTGCACCACTGTGTGCCAAGATTTTTTAGAGTGTACTTATTGCTGACTGCTAATGTCGCATGTTGCTCGCGGAGATGGCGCAGTGCCATCGAAAACAGTTCCAGCTACAGCGCAACGGAGATCGAGCGTCTGAACCCGCCGGAAGATCAACGACAATAAATAATTGTAGCTCACCGTCATCCGGATTAATGGAGA

>ID_110 P_110 Promoter_111 D1_Promoter for RE03105, -250bp and +50bp []

TTCCGACTTCCGTTATAATTCATTTGTAATATCAAAATACAGTGGCCTCCTCCCGATCAAGCAAATTCAAAATTGTAAGAGAAGAGAAGAATTCAATTACATTACAACAAAAGAGAAGAAAGAAGACAACTTCAAAATGTTCTCTTTTGGTTTCTCTCTTGGTTCTCTGCTCTTTCTCATCAAGATTTTCGAAGCGCGCCCAAAAGTATGCACGAATTGATATAAGTACTACATTACGTTACTTGGCTCACTTCGCTTCGCGCCGCCGAGCAAGAAAGACGTGTGTGCGGAGTCTGCGCGAAACTATAACAGAGTTATATTTGAATATATATCTCAAAATAAAATCGTCG

>ID_112 P_112 Promoter_113 D1_Promoter for RH71493, -250bp and +50bp []

ATTCCGCAGTACCAAAGATAGTGAGTCATCGCTGCCAACAGGTAAACCCTAAAGCACATTGTGGGATTCCCCCGCGGTTCTAAGAAACACGTATTTTCAAAACCCCGCTTACAAAGCTCAAAAAGCTCAGCTCACTCTGGGATTGGGAGGCTGTGGAGCTTTCCAGAGAGAACTTTCTCCCGTCTCCAAGCTCCACCAATCCAATCCGAAGTGGAAAACGAGCGAAGCGAGTTTCGTGATGTGGCGAACCATCAGTTCGACTTTCGACGCGCTGCCAGAAACATCGTGAGCGCCGCTTATCCCAAAGTATATATCCAAGAAGTATATATATTATATATGCTGATTCCCAG

>ID_114 P_114 Promoter_115 D1_Promoter for RE44728, -250bp and +50bp [-1 ]

AAGCACTTCAGTAGTTTGTGCTTCAGTACGTAGCAAGCAAGAATTGAAATAAATTATTATGAGACAACAAACTGGTTTACCTACTGTCCCAAACACCTCCAAGCGTTACCAAAACAGTTCCCACTTGGGAGAACGAGAGTGCTTATCCGCGGCCTTGACTTTGCTCTCCAATCTGACAACAGAGACCAAAGCTCTCCGTTGATCGAGCTTTCTCCGCCGTCACTGGAATCGCAATCGTGCCCGCCTATCAGTTGCTTCACCTCCGCCGTTACTAGTGGACGACTGTCCTCGCGAAAAACACGTGCCACACAGTTGATTCGAGTTCAAAAAGTGCATCCAAACCAGAAAAG

>ID_115 P_115 Promoter_116 D1_Promoter for RH74191, -250bp and +50bp [6 ]

TTATGTTTCAGCTGCAGCCCACCTGAAAAATTCACCCAAAACACATCGAACACAGCCAACACAGGCAGGCGCACCCACCACCACCCACTTGTTGCCACATCGGCCAGCGATATGTGGCATATAGCGCTGGCCTGCTCGCACACATGGCCAGCTATTGGCTATATAGAAAGGCGATCGGTGTGTGCGTGGAAATGGCTTTTCCGCGAACGCGACGAAAAATTCTTTAACATAACGTGTAGCAGCGCTTTTCATCGTCAGTTTGGTTAGTCGAACCGCAGCATTCGCTCCTACGACGTCAGCTGCTCGGACTTCAATTCAATTTGTTTTTCTCCCTAAGTAAGTTTTTTTTT

>ID_116 P_116 Promoter_117 D1_Promoter for RH50135, -250bp and +50bp []

TTGCTGACACTAGCTGACTCCGGCAGTGCTTCCTTGATGAGCCGGCCAATCACGGCATTCGGCAGGTTAAGATCCTCGATGCGCTCGACCATTTTGCTGCTGCTTATCTGCTTTTACTTGGTGGGCACTTTTGATTTTCCGTCTAGATTTCGGCGTCTTTGCTTCCCGCGTAAAGAGTAAACAGCTGGCTGAACAGTGTTGGACAAGGCCAGCGTGCACGCATCTACACCAGCGATGTGGCAACTCCCCCACTCAATTCTGTTTGCGCCGCCTCGTTCGCATCAATTGTTTACACATTTTTATAAATAACAGCCAATTACCCCGGCCTAAATGTGCACGGAGCGATTCGG

>ID_119 P_119 Promoter_120 D1_Promoter for RE59078, -250bp and +50bp []

GATTATTAGCGACAGTGGAAATATAAATAAAATGTTTTCTAGAATTGTAAAAAAAAAAGTAGGAGTATATTCAATAAATATTTCATTTTAATATAATGCTATTTTGCTCGATAAAAATGTTAATATGAATTAAAGTTTACTCTTGCAATGCGTTTCAGCAGATACATTTTTAAAAGCGGCCTGTTATGCCAACTAAAACGAAATTGCCGGTGAGCCGTATGTATCCGATCGCATCTGATCAGCTGCTTTCAGTTCTACTTCGTCGCTCAATCGCACAGCAGTACAGAATCTCTGCGGGTTTTCAAATATTGGTTCTCTATGAGAAATAAATTATTATTATTTAGTGCGCA

>ID_120 P_120 Promoter_121 D1_Promoter for GM31428, -250bp and +50bp [10 ]

TATTATTAATACATAAGTACATCGATTTTTAAAGCTAACAAAGCCGTTATAAAATACTAAAGTTCGAGAATATACCAAACCCTTTGAACAGAATTCTGACTGGTTACCCTTTAAGTTATCGATAGAAGCAATCGATTGACCAGCCTAGCTACCCGTCCCTCAATATTATTGCCAGACATGCAAATGCAATTCACTAACGCCTGACACGATAGTTGCACAACAAAAATCGATAGGCCCATCGATAATTCTGCGCCATCTTTCTTTTTCGGGATCCGCACACCAAGTTAAGAGGTAATCGAGTGATTTTTGTCGAAATATAATAGAAAATAGCCCAGTTAAACCCAAAATAC

>ID_122 P_122 Promoter_123 D1_Promoter for RE63789, -250bp and +50bp [0 ]

TGCAGCCGCCTATCGATATATAGATAAGTTGGTTGCAATCTCGAAATTCTGAAAACAAGGAAATCCCCGTTTACAAAATAGGTTTTATGATAACAATTTTTTGTAATTTGATCCAATTTAAAAAGATAATATGGTTCTACTTTCAATAAAACATATAGCTACAGTTGCATTAAACTATCGCGCAGTTTTAATTAGCTATGCTGCCAACATCGTGTTTCCCTTATCAATATGTTATCGATTGTGCTGGTGCAATTGTAAACAAGCATCTCTAGCAAATGGTCGCTAAATTCGCAAAATTGTAGTTTGCTGGGATAACAATGATAAATACGGCCAAGTACTTTGCTCTTCTA

>ID_123 P_123 Promoter_124 D1_Promoter for RE74472, -250bp and +50bp []

GTGACAGCACGGGTTTAGATGTTTTACATATCCATGTACATAGCAGCATGTTCACTTTTAGTTATTAAAGTTCGACCAGTGATATTTTTATTGAACTGAAAATGTTATGCAACTATCGATATTATATATATTCAACAGATATTATTTCAATATAAATTCAACAGAGGAGTTAAACTTATTAGATGCAGGACATACGACCCAAAACTCAGTTGATGTATGAGTACTGCTTGAATGTGTCGATTGCTTGTCAGTGTTATCGTTGGCCTGCCACCTCTGGTACAATATTATAACAAACTAAACAAAACAACAAGACGGTGGAAAAAGGGAATCTTGTGGTGCGGATTGGTAAA

>ID_124 P_124 Promoter_125 D1_Promoter for RH73116, -250bp and +50bp []

AAATGGTGTGGATCATGTGTACATATGTACATATGTACGGACATGTGTGTGTTTGATTTATCAACTGAGACGGGCTTTTTCGCCCGTTTTTCGTTTCACTCAACTTTTTTGCTTGCACTATCAATAGACCCACATGAAAAGGCGGCAGCCCAGGCGCGTGGGTTTGTCTGGGTCTGGGTATAGGTCTGGCATGGGTATGGGTATGGGCATGCCTCTGGCTCTCTCGCTCGCTGGATTCGGGTTCGGAATCAGTTGGCATAGAGCTGGCGTGGCATACGGACGCTCTGACACCTTGGCTTCCCAACCAGTGAACGTTTTATTGGACAAAAGCCTTGCTTGGACGTCGGTCT

>ID_126 P_126 Promoter_127 D1_Promoter for RE39956, -250bp and +50bp []

GCAACGGACAGCAAATGTTATGCCATCGTCTGGCAATCTTCAAAAAAATATTGTCAGCTTGGATTGGTAGCCAATGGCGGAGTGTGTAAATCACGACCCCTTATCGCTATCGCAGCTTAATCTATAGACCAGGCGAACTTTATCTGGGCCGAAATAAAACGTTAAAGTTTGCAGATAAGCCGCTGATACTAGCAAAGTCGCAAAATGGTTTACATTCACCATATATACCGGGCCCAAAGCCAAAACTCACTTAGTTTTCGAATCGAATCGCTGGCCGAGTGGCGTGCGAAATACTAATTCTAGTCCGAGGTTCGCGATCGAACCCTTGAAAAGTGAGTTTAATAAGTGCT

>ID_128 P_128 Promoter_129 D1_Promoter for RE64788, -250bp and +50bp []

AGAAAAAAGAAGAAAAAATCGCAAAGTAGGGGAGACCACACCCCGCTGAATGGAAAATCGTCGAATCGCTGGGCAAGCGGAAAAGTCACACACGCCAGCATGAAACGCACAGAGCGGCGTTCGCATGCGCCGTCCCGCTCGCACGGCGATAGTGGCGGCCGATGGAGCGATGCGATTGGTCGCTGCCAGCGCTGCTGCTGCGCTGCCGCCTGCGCCGGTATAAAAGGACAATCATCGCCAGTAGCCGAGTCAATTAAGGCCAAAGACTCGAGCGGTGAGCGTGCGGCTCTACTCCCATATATACATATATATATATATATATATTCAACATCCTAACCGATAGAAAAGTA

>ID_129 P_129 Promoter_130 D1_Promoter for RE03208, -250bp and +50bp [0 ]

TTTTAAATCCTTTTAAAAAGACCCTTGGCTCGGGACTTTAAATTCACTATTTATTTATTTTTATTTTTTCTCACTTATACATTTACCCAACTTGCCTTCGTGACTATATACCCTGTGTAGTTCACATATAGAGGGTATAATAAATCTCCCACCGGCTTTTCTTGTGGTGGCCAACGACGTCGACTGCGCTGCCGGTGGCTCTGCATTGGCATTTAGGCATTTATGCCCACGGCAGTCGAACAGCCGCTTCAGATTCTCGTCATCGCTCGTGGAAGGCACATCGCAAACGCCGCTCTCGTTCTCGAATTTATTGTGAAAGCTCAGCCGCCCACCGCCCGCCGGAAAAGTCT

>ID_130 P_130 Promoter_131 D1_Promoter for RH06505, -250bp and +50bp [7 ]

TTGCATCAGCCGCTTTGTAAATTGTATTGATTTTAAAAAAAAGATGATAATTTATTATATTTTGAAAAGCACTCATTTCCTTTAATCTCCTACACAGGCAACAGTCAATAGTTCAGCTTTGTTTTAAAACAAAGGTGTACTCAATTCCAGCCAGATCGGTTTTAAATTTGTATACATTATATATATAGATTATACAGATTATAAATCTCAGCTAATCGGCACGGCTATAAAAAGAAGGCGACGCCGATCAATCGGCTTAGAGGGAATCATAATGAAGAGCCTGACGTTATTGGCGCTTTTGATCTTGGCCACGTTGGCCTTTGTCCACGGTCGGTTTGAAGGCCAAAAGG

>ID_131 P_131 Promoter_132 D1_Promoter for RH70179, -250bp and +50bp [2 ]

GATATTCAAATATTACGAACTATAATTAGTTATAATTATTATATAAGTGTCATTAATTTAATTCATACAATCATTCATTTAATTCAAATCAATCAAAGGCAAATAGAGTAGCTAGCATTTATACCTAACAATAAACATTTCTTAAGCACAGTTTGAAAAATATTTATATTCCTATATAATATTTAAATATGCGCGCCAATTCTGACAGTGCAGCGAGTTGGCAGGTTGGCGTGTAACAGCTGTTAAGTCTGCACTGAGTTGACAGCTCTGACTCAAATCCTAAAAATCTACGTGGAGCAAAAATAAACAAATAATGGCAAAAAATCCAAAAAAGGAGGAGGACGGCGGTA

>ID_132 P_132 Promoter_133 D1_Promoter for RE63456, -250bp and +50bp [11 ]

ATGGAAGTTTGGTAATCAACAGGCTATCGATAAGTTTAAGTAAAGTGGCGGGTCAGGGCTACACAAAGTCCAATAATGCATATTTGTACTTTTTGGAAGTTTACATACTAAAAAGTTTAGTAGAACGAATAAACAACTGGTTTTGGTACTAAATAATATATATTGTGTCAGTTAAATGTGTTCGAGATAAATGTTTTGAAATTATCGATAGCAATTAATATCATCAATTTTTCTTCCAGCTCTAGCTTCTCTTCTTCCTTCTTTTGATTTCCGGAGAGGTTTGTGTTTTAATTGTAATGATTATTTTGATTGCAAAAAGCGCTTTAGTGACGTTAATTGCGATGCAAAAC

>ID_133 P_133 Promoter_134 D1_Promoter for RH25914, -250bp and +50bp [7 ]

AAATAAGCGCAGAAACTGCAGTTATTCGGTTTTTTTTGGAAGTCTTTCCTCTGGCGTGGCTTTTAATTATAATATAAGTCTATCTGAAACTAATAAATGTTTCATAAAAGTGCTAAACCAATTAAAAGACGCACAAAAAAGAATGCATTTTTAAATCCACCTGGGGTTTGAAGCCCCGACACACTCCCTATCGATAAGCTATCGCCAAACATCGCAAAACTCAGCAACCCTGTCATTTTGACAGCTACGTCAACCTTCTTTTTTGATATCCGGTTAACGCAAGTGGTTCGTCTTTAATTTTTCCCTCTTAATTTTTGCGAAAAAAAACCCGACTTTGAGCCCCTAAACTT

>ID_134 P_134 Promoter_135 D1_Promoter for RH29296, -250bp and +50bp [11 ]

CTTTCTAGCACACGTAATTTCTTTTAAAAGAAAAGTTGCTGATTAAGTTTTTGGAATTTCCGGTGTGAACACGACATCTGATTGGCCAGACCTGCCAACTTGTTCGAGGTGGGTGTAAAACTCCGATGGTATGAGTACCTGCTAGGATTTTAGCAAAGTTCGAACCCAATCCGTGCTCATTCTTGGTTATAATTAGGTTATTTTTTCGATATTTTGAGGTATATTTCTACGATAGATCGGCGGTCACATCGTATTTCCCTCCTTTTCGTTTTCGTTTCCGGCGAAGTAAGTATAATAAAATCTCCACGTTTTTTTTAATGCTAATAATTGAAACATCTCATTGGCAAACT

>ID_135 P_135 Promoter_136 D1_Promoter for RH22779, -250bp and +50bp [3 ]

TACATATGTGCGTGTACACGTGTGTGTGAGCATCAAACTATAACCACTTGCTAATGTAAAGCACTTTTTGACGTGGTCTTCGCCTTCGCCTTCGTATATCAACAATCGCCACACCATGCCAATACCACACCACACCACCATACATTACCTCAAACCTATACAGATATATGTACATATATATTTCCATTCATGTATATGTGTAGAGAACAGTGGAGAATAACTGGCATCAGAAATGGGCGGCTGCGCTGCGTTCAGTTTCAGTGTAGCCACCGTTCGGAGAGCACGCGATCCGATCCCCGCACCAAATTCTCATCTCCGTATCCCCAAGATGTCCCTAGTGCGATTGATCG

>ID_136 P_136 Promoter_137 D1_Promoter for RE07977, -250bp and +50bp [8 ]

TATTAAATTAAAGGTATCAAATGGTTTAAGTAGATTACGGAAAAATTTTGAATTCTTTACATATTACTTACAGACTTTTTACATCGCGCCTTTCATGATCTTCCACAGTGGCGCCATCTGCAGTCGCATAGGAATAAGCTCTGTATGCTAGTGTTACCGCTCACCAAAACTGTAAAAATAATCGATATCTGGCACAAAACGATAGCGTTTCGATATGTTTGAGAGTCCAGGCCGTGCGCTAGCATTAACAGTCCCACCACTAACGCAAAAGTTTTCGGCTGTAAAAACGTAAATATTTAAACTTTAAGCAAGTTTAGTGTAAAAATAATCAAATCATGTGCGTTAATTTG

>ID_137 P_137 Promoter_138 D1_Promoter for RE22722, -250bp and +50bp []

ACCATCAACTCTGTCTTTTTAATAAATAGAAATATTAAAATGCCCACCAGCTGCAATGCAACTTGGTTAAAAACTAAAGCTACCAAATACTCTTACTTAAGAGCATATATTGATGTAGTTTAATAGCAAAATTCAAGTTTACATAACGCGACATTGAATAGACTTTCTTTCTCTTTAAAAATGCCGGAAACAGAGTCAGATGACTGATGAATGTCGGGTATAAAAGGCCGATGTCGCCGCTAAGATTTTCAGTTGTTCCGAAATGGCTGTTATGATCGTATTGCTGATCGGTGTTATCACCTTCGTCGCGTGGTATGTGCACCAGCATTTTAACTACTGGAAGCGCCGGG

>ID_138 P_138 Promoter_139 D1_Promoter for RE44427, -250bp and +50bp [3 ]

AATTTAAAGTACCTTCAATTCTATTTGGTTATAATTTTGTTTGGTTTTTCGAAACCTATATGTTTCAAATCAACTTATGAACTAATAATATAATTTTTGGTTATAAATATGTTTATTTTTCAACGGTTTTAAACTAATACCACTTTTTAAACTTAATCGAACTTTGGAATTGATATATATCGATTTTTGTGCTCGAACCGATGTATCGACAGCGGAATCGGTTTCGGTCACATCTCTAGGTCCACCGACTATCACTCATCTCTGAACTTTTTGGAACGCAGCTGCATGAATTGTCGTAGTTGAGCGAATTTAAACAAATTAACACAATACTTGTGGTATTACCTGGTAAA

>ID_139 P_139 Promoter_140 D1_Promoter for RH56632, -250bp and +50bp []

CGATCACCTGGAATATTCTGCTATAAAATGACCGTATATACAATTTATGAAAGCACATTTTTCTTGTATACTAGCATATTGTATATGTGAGAGCAGATATTTTGTGATTTGTTGGGATTCTTGTTCGGATGGCTAATGTATTTATTGTTGGGGCTATATAGTCATCCCTATCATTTTCTCTGGCGACAAAGTTGCCTTCGTGCCGTGTGCAGACAAAGACTGAAAGCGCTTGACAAATTTTTCAGCGCGATCAGTCGAAAATCGTTGTTAATCGAAGCTAAACTAAAATTAAGGATATTTCTCTTGCGAACCTTTTTATTACGAACGAAAAGTAAATATCAGTTTTTGTC

>ID_141 P_141 Promoter_142 D1_Promoter for GH21828, -250bp and +50bp []

AAATATGCTTTGAATATGTTCATACCTGCAGAAGAAGCACTTAGAAATATTTTAAAAGTTTCATATAAGTGATTGAATTTTTTATAAATTCGAAGTCAATAGGTTTTACTTGTACTGGTTACTGATCGTTGCGAAATTGTCCGAATTCTGGTCATGTTAACTTAACATTAACAAAGCTTCCAACTGGGAACTATCGGTGCTATCGGGGATCCGAGCAGTGTCATCGATAACATTGGCAATCGAGTTTTCTAAATTCTGAGTTTTCTTTTCGTCTGTCGTGTGTGCGTGTTGCCTTGCTTTCGAGTTGGCGTTGCAAAAATTCCCCTTTATTTTGCTGACGAGATATTCCA

>ID_142 P_142 Promoter_143 D1_Promoter for RE50843, -250bp and +50bp [8 ]

AAAATATTAAAAACTATACCAATAAAGTCAACTCTTCGAACTTACTTTGTTCCTTCGTACGAAAACCTTAACTTATGCTTTCGGCGTATTAACTATTTGAATTTGAATTTTCCGCCCGAATGTGTGATCATCAGACATCGTCATCGAATGGTCACACTTAAGCATCGATATTTCGACAAAAACATAAAAAACGATGGAAAACATCGATTGGCGATGCAACAAAGTTTTCTTACATCACTAACAGAAACCGAAAATTCAGTCGTGAAATATCCATTGGCGACGTCGCTTGAGGAATAAACTGAAGCGCTGTGAATATTTAGAACGATGAAGCTGTTCTTATCCGTTTTCGT

>ID_143 P_143 Promoter_144 D1_Promoter for RH63924, -250bp and +50bp [4 ]

GTGTTAAAACGATTAAATGGATCAATCTAAAGCCAGTTTAGCAATCTTTAAAGGAATACTTAAGAGCGCGCATGTACATACTTTATAAGCCTATCAGTATTTCAACCCGGCGAATTACCCGCACATACATACCTACAAACGATCCTAAGTACAAACATATGCATATGTACATATGTGGGAATACTTGCTCACTTTCTCAATTTTCGTACATTCTCAAGTAGGTCTCGAGCTGAGTTTACGATCTCAGGTTGAGCAGTCCGCATTGAGAGGCCGATGCGATCGTTTGGGTCGGTTGGAACTTTCTCTATAAGTCGGTTGTTCCCGCAACCTCTGTGTGCCTGTGTGTGTAT

>ID_144 P_144 Promoter_145 D1_Promoter for RH41914, -250bp and +50bp [1 ]

CTATCGGTACTTTAGTAAATATGAATTTAAAGTTAATTTGGAGAAGATATTATATGATAATCAACCAGCTTATATAAGTTATATAAATATATAATATATATAAGACACAAATTTAAAAAACCGTTTCACAAGTCATACCATAGATGGACCTTATCTTCCCGCCAATTTTGGCCAGCTTTTCGAGCTTATCAAAGCTTTTTTCAGAACTTTGTGAGAGGGCACACCATCGAGATTCGGATTCAGTTCGGTCAGTCAGCGAACAACTGTGGAACGGTCAAGATGTCCAGCAAAATTCCCTACGTAAAGCACAACAACGGCACCCAAATCCAGTCCATCGGACTGGGCACCTA

>ID_145 P_145 Promoter_146 D1_Promoter for RH36834, -250bp and +50bp [1 ]

GGATGGGGCATCTTTGGTAGTGGATCTGTTGTAAAAATATAAAATTGAAAGTTCTTTAGTTATTTTTCTTTTTTTTTTTTAATTAAAACATTGTTGAATGCACATTTGGAGCACTCGGAAGACCCTTCTTCGCTCTCGTATTCTTCTCTGCGCATTCCTCCCTTTCCCGGTCTCTCTTCTCAATTCTTTGTTTTTTTTTGAGTGCCAACGACGTGAGCTGGTATAAATGAAGCCAAAATCGTACGCAACCGTTTAGTCCGAAACGTTTCATTAAAGCCAACGCACGTTGAAGATCGCGCGTTCTTCTCCGATCTTTCGAAATCGAGTGATTCAAGCGATCCACAGAAATA

>ID_146 P_146 Promoter_147 D1_Promoter for RH44224, -250bp and +50bp [1 ]

GATAAGTTGGCAGCTCCGTTGGCATTAAAATGGAGCTCTCTTGATTAAATTTTCAACGTGGGGAAATTCTCTTAATGCTTTCTCTTTGCCGCATGAAAAGCTTAGCAACGCGAACTGCTGCTAAACTTTTGTTGACACACAGTTAGCTGTGATATTTGCCGTGAGTGTGTGTATGTGTGTGTGGGGTTTGGGGGGTGGGAGGGCTCTAGTTGGAGATTTCTCGAGAGCGTGTATTTTTGTAATAGCACAATAGTTGCTGCCGAGCGTCCAAACGCTGCGGACGCGATTTTCGCTTCGCTCCTCTCGGATTCGCAACGCTTTTCGAAAAAACCTCGCGTAACGCCAGAAGC

>ID_147 P_147 Promoter_149 D1_Promoter for SD08914, -250bp and +50bp []

GCATGGGCGTAGCCAGGTGCATTAAGGGGGATTGACAATTCTTGAATATTAAACAATAACTATTATTTGTCATTTTTAAACACCCATATTGTAAGGCATTATAGTACTTTCAGGTACTTTTGTTCAGGTAAAAATTTAAATATTAATAAAGAAAATGAATATAAAGAAAATAAAGGAGATGGGGTTCTCCACACACGTCAGTTCCCAGTTGTGAAATGTAATGACTGTCAGCAGGAACCAATGGAATGGAGCACTCTTTTCAGTTCCGATCAGGTTAGTTCTTTCCATTGTTCTAGCGCCTATCAAACAGAGAGCCGCGAGCACTATCAATCACCTCCCGTATCGATAAG

>ID_148 P_148 Promoter_150 D1_Promoter for RE46351, -250bp and +50bp [3 ]

CGGGATGGCAACTGTAAGATACGGGATTTTGTCCTCAAAGTACGTTATTTTATATTATGATTGGTATTTTTAGTGTACTTTTTGAACATGATATTTTAACAGAATTAATTGTTTTATAATTAATTGAAGTCTCCGCACTCTGAAAACTCTCTTACACATCGATATAACAACAACTTCGATGATCGTTGAAAATATCGATGCTTCGTCTATCGATACTGACGAACATCACTATCTTCAACAAGTGTGAACTCTCAGTTGAGTTTTGAACTTCAACAGGTGTTGAATACGCGCTAGACTAGAAAGCGATCCCACGATCAACCGACTCGGTAAAGCAAAACAAGCCATCGAAA

>ID_151 P_151 Promoter_153 D1_Promoter for RH12051, -250bp and +50bp [1 ]

CTATCTGGTGCAATTTGGGCTGCCAACTGTGGTCAAGTTAATCTATAAAATTCTTGATTCAAAATGCAATTTTAAGAACATTTTATTTCAAATTATATCAAATCAGATAAAAAACAAATGTCCATGAAATGATTAGGTTAGTTAAGGATATTTTCAATCAAAACACTATAAGAAATAGGACAAGGGAAGCCAAACATAAAATAAAGCCAAATTTAACTATAAACTAGCTTAAACGGCAGTGCAAACAGTTCATCTGGTCTGGCAACTCTCCGCCGAATTGTTGTTACGCGAGCGATTTCTTTGGTCTTATTTCTAGTTAAAATTATCAAAGAAATATTATAAAATGTTGG

>ID_153 P_153 Promoter_155 D1_Promoter for RH71856, -250bp and +50bp [1 ]

TCAAATAAGAGGCCATGAGCGTTGCAAAAAATAACTCATTTAATGGTCGAATTTTTCCATTGACTTGGTTTTTATGGCTATTTCAATTTCTTATAGATATTTATTTGTATAAAAAAAATAAACTGCATTCACTTTATTCAGGGCTGCACATATGTACGCAGGGCGCGTACTTGAATATTCGCTTCCCTAAATTTTTTGTTATGGCAATGTGGTCACCCTGTGGAAATCGGTATCGATAGCCCGATAGGTTCAGTCGTATGAAGGCTTAAGCCTCCTAATATCGCCAACAATCGCCATTCATTTTAACATTTAGTTAATTGCTCTTGAGCACTAGTTGTGGTATTACCTGT

>ID_154 P_154 Promoter_156 D1_Promoter for RE14453, -250bp and +50bp [6 ]

ACCCGCTTAGGAAATCGACGTAAAACTTCTATACTATATCCTCAAGTTATTTACAATTGTAAAAGCAGAAAAGGTACGCCTGTATAAAATTGGAACACGTAAATTAGATTTAAGAATCCCTTAAAGAAGTAACTGTTGTTTGTGACAATAGGTTGCTGGAGCTTGAACCTAGTGTGACCAAACTTAGCCGGCCAGAGATGTACCGAAAAAGTCGGCGAGAAACGTATAAAAGGAGTAGCACGTTGGTATTTTCTTCAGTTTTATACTAACTTTTGACGACATCAGATCACAAATTGCTGGGAGGTTTGCACCCCAATCAAAGCAAGTTTTACAACAAAAGTCAATTTTCG

>ID_155 P_155 Promoter_157 D1_Promoter for RH06006, -250bp and +50bp [6 ]

CGTACCGCCGCCTGCCTTTGTCTGGCTTTATGGACTCCACAGGAGCGCAGCCCAAAATGGGGGGCTTGGAGTCGAACACCCTCCCGGTGGAGAATCCGTCCCTGGAGCAACCGAGTTCGTTGTCCAGTCGGGCGAATTTGAGTCTGGTTTTCCGCACGCGTTTTGTTTACTGTCCGGTGACTTGTTGCTGTTCTCTGTGCCCGCCTTCGTCTCGTTTATCAGCGATTGCGAATACTCGCACGGACTGCTCAACACTTTAGTCAATATTTAGCCGCTTCCCGGAGAGGTTCATAGATCGCAGACGCACTACATAGTATCAGCATGGTTATCAACTTCGCCGCCGGTCCAGC

>ID_156 P_156 Promoter_158 D1_Promoter for RE43011, -250bp and +50bp []

AAAAGAGTATGAATCATTATCCAAAGTCATGAAACTCTAATTTCAAGCAATTTAAGTGATTTAAGTCATTAATTCCTCGAGTGCCTGCGTGGAATACAGCTCACCCGGTGTTCCTAAGCAGCCCGTGGATTTAAGAATGGAAATTGATGCGCTGTTTATGCGGAAAAGGAGTCATCGAAGGGAAGGGAACGGGGCACTGTTGTGGTTTTGTGGGCTTATTTTTAGCACTTCGGAATTGGGAAATATTTGAATAAACATTTCCTGGAACTGCTGCGCACTTGGCTAATTGATTGCTCTTGAGTCATTCTTTTCTTTTTCGGCTGCAGGAGGGTGCGGAGGTGGAGGAGGAG

>ID_157 P_157 Promoter_159 D1_Promoter for RH45888, -250bp and +50bp []

CTTAATGTATATAATAAATGTTGAATAAAGGAAAAATATAATTAGTCTTTTCAGAGAGCAGATTACGAATTTGACGTCACAGATTTATTGATACGCCTAGAAACTTATTTCTTGATTGTTTTAGAATTAAATGAACGTTATATCGTGAAGGAAATAATTAGTTATTAATAATTAGCCTAAAAACTAATTGCACCAAAAGGAACTAGTTCGATGAGCGACAATTTGTTGGTATTTTCTGTGCACCAGACAAGTACGGTCACTCTGCGAAATTGCAAGGGAATTTTCTCCAATAAAAGGCAAGAAAACTGAGGAAAATTGAAATTAAAGAAAAATTAATGGCACACAAGCGC

>ID_158 P_158 Promoter_161 D1_Promoter for RE06067, -250bp and +50bp []

AAACAAAAATCCAAAACACTCACTAAAAACGTGAAATACGTCGTCGCCATAAAAAAGACCCCTAAAAGTTGAGTCACGGCAATTCGTGAAACTGTATTAGCTGACAGGGCATCACAAACAAACGCAATAAAGTCGGAAAACATTGACAGGAACTACGTAATGCCTGAAAACCAGATAATGGCATCATCGGCAGAACAGTGAGATAAGCAAAAATACAGCAGCTAAGCTATAAAGCTTTTGAACGAGATTTCAGTTGCTAATTATCAGCCATAAAGTCAGGACGTGGTCTCTACCAAAAGATGAGATTCCAGTTGTTTTATATACTGGGCTTGCTCAGTGTGACAAGTTTG

>ID_160 P_160 Promoter_163 D1_Promoter for RE11167, -250bp and +50bp []

TATTAAGTTCGTGGTGAATGGCAAAACAAAAAATAAGAAGAAGAGTAGGCGGTGTAAGTTTAGTCATTCAAAAAGGTAATTTTAACAAAACGAATTCTAAATAAAAAGGTCCTTTTAACAAATAAAAAGTTCCGAAGTACTACATTGCGTGATAATTTAATAATTACATTCAGTTTTAATATCTTAAACAATTTCGGTGGCTTTGTGCGCGCATCACTGTGATTGGCATTTAATTTTACCGTGGCACACCTGGTATATTTCCGCTTCCCGAACTGCGGTCACACCGGCGTCTTTTTCTTGTGAAATTTTCTGTATTTTGTTGTGAAAATAATAATAAATATGGGTTCCTC

>ID_162 P_162 Promoter_165 D1_Promoter for RE43234, -250bp and +50bp []

ACAGCAACAACACGAGCACAGCAGCAATAACAGAAGCAGCAGCAGCGACAACAACGCTAATAACAGCAACAACACTTATATCAGCATAACAGAAGCAGCAGCAGCAACGAGAACGACGGCAGCAGCAACAACATTAATAACAGCAGCAACAACAGCAGTCGCTGCGACAACAGCAACAGCAACATCGACGGTGACGGCGACTGCGACTGCGGCGGAGTATAGAAAAAGCCGCAGACGAGCGGCCGCATTTTCAGTTCAGTTTCAACGCTCTTCCAACGAGCACGCAGCAACAGCAGAGCAACAACAGCAGCAACTGTCCACGGCAGCAACAGCGTTTTGAGTTTCGATTT

>ID_164 P_164 Promoter_167 D1_Promoter for RE68314, -250bp and +50bp []

CGGCTCCCAGTATATTTTGCATTTCCTACTTGTACCGATAGCTACGATGTGAAGTGGTCCAATCCCGCCTGAGATGCTAATTCCATAACAAATGATTAGAAACGGACACTACGAACTCAAGTGCACGGCATGAATGAATCCTCTAAGTATGGGTGAGATACGATTTCATTGTTGATTGATAATAGGAAGCCTTATCAATAAATATTGTCTCAAAAATCGTATAAAAGCAGGTTGCCTCGACTAGAAGAATAAGTTTCTTCACAGCCATGAAACTGTTCCTGCTAACTCTCTCCGCGGCTTTGGCCCTGGTGGCCGCCTCCCCGACCGGTCTGAACCGCACCACCCTCCTG

>ID_165 P_165 Promoter_168 D1_Promoter for RE02710, -250bp and +50bp []

ACGGTTTCGCGGGGAACCAGTATATCGAACTGGTGCTATCGACAGCAATGCAGAGTCTGGCAGCATTCGTTTAAAGTATCGTAATTTAAAATTTCACTCAACTTTTTAGTCATTAGCGATTGAATCTTAAATTATAACATGTACAAATTTTATTTTCTACTTTGTATATTTAAGTTATTTCGCTATCACCATAAGAGGAAGAAATCGATTTACCACCGCTAAAGCTATCGATATCTATCATCATCAGCTATCATTAGTGGTACTCCCATCTGGTTTGTTTATAAGCTTTTTTACGAAAATAAATTGAGGCGGAGGAGACCGAAGTTTTTCCACTTGGCCAAGATGCACCA

>ID_166 P_166 Promoter_169 D1_Promoter for LD38689, -250bp and +50bp []

AGCTCTCATGAGACAGACTTTTAATTCATATTTTTGTTTTATTCTTCCAATTGGATTAAGGTATATGAGAAATTTTTTGAAGCCCTGTGTGTTTTATTTTGGATTCGTGCTTAAGTCTTTTTTTGAAAACGTACTTTTGATAGTTTTATGTTCAAACCAGATCTCATAGAATATAGTGGCAACGGAACCGACTCCTAAAAGTGCAATTTTCCTCCGCGGCAGAAGAAGAAACATGCATCAACACGGCGTATACGGTCACTTTAACCGGGTCGTGCTCCAAGAGAATTCCTTTCTGCAAATGGACGGCATAAACAAGTACAACGAGTTTGTGGCGCTGCACAAGCCACAGC

>ID_168 P_168 Promoter_171 D1_Promoter for RE14589, -250bp and +50bp []

GAGAGAGAGAGAGATGGACTGGTGCAAGGATATTCGCTGGTGGTGGTAAGGCCTGTCGCTCTCTTTCATTCCGCCGGATCGCTGCGTCTCTGTTGCACCCATCACAACTTTGGGGGGCGGCGACAAGGATGCAAGAATACGGCTAGCTAGGCAATGTATGTATATGTACGTATTCACATACACATGTATGGGTATAGAGATGTTTCGTTCTGAAAGGCAAGTCCTGACGCCGTATTCCTCTATTCGATTCAGTCGTACGTGTTCGTTCGTTGCTTTCGTTCGGTTTGATTCCATTCGGTTGTGTTTCTCGTTCGAGAACATTCATTTATAATTTTTTACTCCTCTGCACC

>ID_171 P_171 Promoter_174 D1_Promoter for RH07189, -250bp and +50bp []

CGTTTTGCAATTCGTTTTGGAGTGCTTAATTGTGCGCCCATAACGGTAAACGTAGCGAAGGCCGAAGAATTTTAGGACTCCCCCTTTTCAGCCCCCACGAAAACGCATTTTATGAATGAAAAGCGGGAGGCGGAAAGCTCGACTTATCCCCTGAGCTTTGACTTTAGGCCTCTGGGGAGCTTCGCGGAGGAAACCCAAAAAGTGCGCGGAGCTTTAGTGCGAAAAACACATTTTCACCAAGCACATTTTCCATTCTGTTTGTTGTTCTCGCTGCGTGCGGTTTGTGTTCTATTTATCTCGAATTTGTAGGCTTTATCCACCAGTTATAGTTTGCCAAGTCGGTTGCCTTT

>ID_172 P_172 Promoter_175 D1_Promoter for RE34515, -250bp and +50bp []

TATTTACCCACTTTCCAGGAGGATTAGATTCGCGGAATAGCTTTAAAAAGTTTAACAGTAAAAAGTTTGGCGCATAACAAAACATTTTAATTTGGAATACATTTTTTGAATATTTTTTTATTTTTGTTGGACTTGCTGTTACTTAAAAATATTGCAGGCTTAAATAGCTCCATCGAGTTAATTCTATTTGGTCCCACTGCTCTGAGCAGTCGAAAGTTTAAAGCTGGGCCGAAAGCTTTCGCTAACTTCAGTGTCGAACGCGGATTTCAGACGAGCGGTGGCCGAGAAAGAGAAGAAGAAGTGATTTGAGCCGAGAAACTGAAGCTTGCAACACACCGAAAACACACACA

>ID_173 P_173 Promoter_176 D1_Promoter for SD16890, -250bp and +50bp []

ATGTTTTAAGCTGACGGGGGTTACGGTAAGCTAGTTTTTAGAAAGTACGATCTCGTAATGCCACAGATAATACGCAATTCTTGTACGTTTTCCAATCTGTTCGTATTTATGATGACTGGCTAGCGACAGTGTGGCCACTTTGTGGCCAGGGCTGGCGGAAATACCGAAATACCGCAAGGCTGCAATCGCCCTATCGATAACGATGCGCACTGCCGCTGGCCAATCGATAGGTATATGTATGTATGTAGAATTTGTCAGAAATTTTCTCGCACAAAGCAAAGTGTTTTGGAGGATAAACGCCAATTAATTGTCAAGCGATGTGCCAGCTCCTGTAGAATACACTCAGCATA

>ID_174 P_174 Promoter_177 D1_Promoter for RH24992, -250bp and +50bp [5 ]

TAGCCATTTTGACAAAAATTCCTTAAGTGGCATTTTGTTGTCTTTTTACTTAAGGTAGTTAAAAAAATATTTATAAATATTTATAATAAAAATCATAATTTTCCTAAAGGCATAATCTATACCTATAATTTATTACGTATTCTAAAATATAATATTTTTACATCTTGCCTTATAAAATAAAGTAAAATAAATGTCGGCAGCACTGTTGCGCTCTCTCAGTATTTATATTTAAGCAGGGACTGTGTTTCTCATTTTAGTTGCTGTTTGGTGTTCGCGCAGTCCGCACGGCTTTTTGTGTCGCGGGAGATATCTCCGATTTTTTAACTGCCCAAACTGTTGGCATTATTACA

>ID_175 P_175 Promoter_178 D1_Promoter for RH05466, -250bp and +50bp []

CTGCTGTTTTCTAGTGGTCATACTGCCGTCGATAGGCCAAAATAATCGAATATACTAAAATTTTGAAAAAACTCGACCGCCTTACGTTCAATATACCAAGCCGAATTTCGCGCGTTTTAATTTAAAATGTGTGCCCCTTGTTATCGAGTGTCAGCGTATGCGCGTTGCCAGACAGCTGGCAACTCCAATGAGTGTAGAAGGTGGCAGCCCTTTAAGGCACCAGCTGACCCCATAGAGCGTTTTTGTTGTTCTATCTTTTATCTTTTCTTTTACCGTTTTGTTTAGTAATTATCTTGCTTAATTTAACCGTAGTTAATTGTGATTTGCTAATTGCTTATCTTCAGTAAGTA

>ID_176 P_176 Promoter_179 D1_Promoter for RH42089, -250bp and +50bp [1 2 ]

TAGAATATTAAAGGCAATTAAATTATAACCCTGATCAAATGTCCCAGCTGTACGATCAATTGTGGCCCCAAGAAACCTGTAGCGCTGCGCAGTTTTAATCAGCGTAGAATCGGGAGAGGGAGAAAGAGACGGGGCCGGCCGCCAGAAGACTGAAGAGCGGGAGAATTGATAACGATAGCGCGGCACTCTCCACATTCGCGTAGAAAGCGGGAGAGGCGGCAGTATAAATTGGACCGACCTTTCTGCAGATGCATCAGTTCAGCTTTAAATGTTAAAAGCGACACATTAAATAAATAACAATTTTAACTAGTGAAATCATGTCTCTGACTATTACTTCCAGTGTCACTCAT

>ID_177 P_177 Promoter_180 D1_Promoter for RE08625, -250bp and +50bp []

GAAGTACTCGTTGATAGCACCAAATATAAACAATGGGTGCGAAGCACAAATGTTTGCTGTAGTCTCACCGCTCGCAAAGATTTTTCACTGAGAGCGGGCTAAGAGATAAGCGTTCTATCTAGCAAAAAAACCTGGCTGAATAAAGCTTGAAACCTGTCGAGAGCGTACATACTTTTCTTACCATATTCATACACACATATATTTGATAGCGGCGTGTATAAATTGGTGCGTCGGGTAATTGTGAAAATCAGTTTGCAGCGTACTGAACAGGTGGTGAAACGGATCTAGCCCAGAAATGGCACTCATCGAAATTTGTCTGGCCCTTGTGGTCATCGGTTATCTCATCTACA

>ID_178 P_178 Promoter_181 D1_Promoter for RE36919, -250bp and +50bp []

CTGTGGTTGTTGTTGCGGCGGCAATGGTGGTTGCTCGATATACCGATATATACCTGCCCCGGCACCCTTAAAGGCGCGCCTGTGTGCGTAGGTAGCCAACCACACCACCATACCGCCCAGGATAATGGGCGGGAAAGAGACGGCGCTAGCGCCGTGCAAGTGGGACGGGGCACAAACGCCGGCAGAGCCGTCGACAGAGGCGGCGAGCAGCGCATTCCACTTTAAATACTGCCAACGATCGCAGCAGAGAATCAGTTCACTCAGCGACTTTAGCGTGTCGAGAACGCACAGAGAAATATATCGTTTTCGACTTTGGAAAACAGAAACAGGCTCGAAAGAAGCAGAGTTCG

>ID_180 P_180 Promoter_183 D1_Promoter for RH59357, -250bp and +50bp []

AGCCCAGTCAAATATTTTAAGCGTAAAGTAACAAACCTAATTTGTATACGGCTATCGTTTTAATTCGCACACGCCTATCAACTGGATGATGTTCACACTAGAGTTACTCCCATTTCAAAGCCGGGCATTGGAAAACTAATCTCATGGCGTGCCCAGATCTCAATTGAGACTGGTTGACTGGTTGTTCAGGTGGCCGGGTGGCGATAAGCCGATCGATGGAATAAAAGGGGCCGCAATTGCCGCATCTCAAGATAGTTGCGGTCTGAATTCGCCGGAGTGAGGAGCAACATGAACTACGTGGGACTGGGACTTATCATTGTGCTGAGCTGCCTTTGGCTCGGTTCGAACGC

>ID_182 P_182 Promoter_185 D1_Promoter for RE36781, -250bp and +50bp []

TATAATGAAAGCATATATATTATGGAATTATGAAATGAGTTATTTAAATATAATATGTTAGGATTGATTTTAATGTAAGCTCATTTGAGCAGTTTTTTTTAGGTGTTGAATACATTCGCCTGATGAAAGGTTTTTTTAGTTTTGGATAACTTTCAATAGCTTTTGCACCACTGTATCTGAATCAAGCAAAGAGCGCCTGCGTTAAAATTGTTTGGACTAACGAAACAAATGTACCGCACTTCGCTGACCAGTTTATCTTCCTCTGCATCGACGGCTGGTGACTGTGTGCTCAGGATAGAGGTCCTTTGCCGAAAAAATAAGTTTAGCCTAATAGTAGTGAAGGTGAGTTT

>ID_183 P_183 Promoter_186 D1_Promoter for RE58155, -250bp and +50bp [6 ]

TCTGGCGGCTGCAGTAGAGTTTTGGGATGATGTGGATGCTGGGATGAGTGATTTGATCCTGGGGAGTAGTGTATTAGTAGTGTAGTATTTAATGGTGAGAAAACTTAAAAAAAAATATCGGGCACAGTTATTTTTGGGTGAAGATGCCGTTAATTTTAAGGTAGCCCTAGTTCTCGCCAGACTTGAGAATACCCCAGACCAAACTATGTGGTATTTCAATCAAGTCAGGTGGTCTGCCTCGTATTTTCCACAGTTATATGTACGGTCACACTGGCGAGCGGACGTACACTTCGCTTCGATTTGTCCGCCTGTGTTTTTTGTAACGGCCACGGAAGTGCATTTCTGGTGTT

>ID_184 P_184 Promoter_187 D1_Promoter for RH53041, -250bp and +50bp [2 ]

GACTAGGAGGCGTCCGACACACACACATGCACACACATAGACGAGCCCCATTCATTCATGCCTTCTTTTCGACCTTCGCTCGACGCTCTGTCCCCAAAAATACTCTCTCTCCAACTTACCCATACAGACAAGCGCTGAGCGAGAGAGGGCGAAGAGAGCGCCTGTTTTACCGTTGGGGAACAACAAAAAGCTTGAGCGAATAACTCTTCTTCTTCCGCGACGAAATCGGTGTGCGCTATATAAACGTTGCGCATTCGAATCTTGAACTTCAGTGCCACAATAGTGGATATCACATTTAGTAGTTTGTTCCAAAGGTAAAAGACCACAAAAATCGCCGCAAAAAATATATC

>ID_185 P_185 Promoter_188 D1_Promoter for RH69301, -250bp and +50bp [2 ]

CTGTGCTCTGCCATGTCAGTTTTGGTGGGATCTATTTTTGGATCTCACGCCCAGATACATAACCCCGGCCCCCGAGCCACTCTCGCTCTCTCTCGCTCTCGCCCGTTCGTGAAGGACGGTGGGCAGGTGGGCGGCTAACGTTTGGCTTTCGGAACATAACGGAACCGAGCCGAACGAGACGATGCGATACGATGCGATCCGATCAACAACGATCCGTATCTGTATCCGTTGGGTGGCTAGCCGCTAGAATATAGTCTGCCGTGGATCGTCGGACCGTTCAGTCTTTTTGTGTCGTTCAGTATCATCCGATTCGTGTGATCGACAGCATAGCCGAATTTTTTTTCTTTTTT

>ID_187 P_187 Promoter_190 D1_Promoter for RH65957, -250bp and +50bp []

TTTGTAGAACATCTGATTGGGGCGTTGCCACCCAACTCGTTTTATCTGCCGGTGGCAGCTCTGCCTTGTTTTTATTTTAGTGTGCGCGGTGTGTTGAAATCAGGTATTCAAATAAATGAATTTGAACCGTAGTTTCCGTGAAAAGCTGACCCGAAAGCTCGGCGCAAATAGCTTAAACCTGTTATCAGGGCAGCGAGCGTTTTGCTCGCAACTCGTGAGCGATACTACTTGTGTTCGCGCAGTGCTTCGTTCATTTTCGTTCCAGCAGTGGCGCACTAAAGTTCCCCGATTCGCTGCGTCGTTTTTCCATAAATTTGCATATATGTATTAGTTGGGTGGAATACATATAA

>ID_189 P_189 Promoter_192 D1_Promoter for RE53358, -250bp and +50bp []

CAAAATTTGATTACTATATTAAGAAACAGTGGATAAAAATAGTAATATCTTAGAAGTATCACACGTATACGTGTGTATATTGGTTGCATTCGCTTACTGAACAGGGGAATTAATTAATTTAAATATAAAAGTTTATTAAGTCTACCAAATTTCGTTTTGCACGTAATAATAATAAGAGCTAAAGGTTTTCGCGGTAATATTTAGCTTTGCAAATGCTGGTAGTTTTTTTGTCAGCCAGTTGGCAGCCCTCTCAGCTGTACTAGGTGGCAACGCTGCTACAGTGCTGACGTTCGGCAAAAAAGAGGAAACGTAGTAAAAACACTCGAAAAACTCCGTATATTCCGATAAAT

>ID_191 P_191 Promoter_194 D1_Promoter for RH26688, -250bp and +50bp [0 ]

ACATTTAAAGGACGTTGATTTGTTGAGGAGAATTTAGCATGGGTTATCAGATACCCTAACCTCCTAACCTCTATTGTTCGATCAATCGGCATTAGTTGCGGAGAGCGAAAAGCTTTGCAAGCTCTAAAAACCAAAACCGAGCGAGAGATGCTCTCGTGCGAACAGGTCTCCGGCAAGTTGCCATCGGATGGGATCGGATCGCACTGGTGGGGATCGGGATCGCTGGTGGCAAGTGCGGATCGTGGGCCTCAGTACCGCCCGAGCTAGCGACGCGTTAAGATCCGCATCCGAATCGTAAACACAACGAGAAACTTACACTGCGCGCACCGTTACACTTTTGTGCTGGGAGC

>ID_193 P_193 Promoter_196 D1_Promoter for RE17949, -250bp and +50bp []

TTTACAAAACATTGAATGACCTATTTAATTAATAATTGTAAACAATATTCGCATTTTGTTAACGGTTACAATGTACGTTTATGGCAGTTGATTGCATCAATCCGGGAGGATTATATCTTGCGGTACGATGTACAATGCTATGCGATGTTAATAAGAATAAGATGTACGTAATAAGAATGTGGTGACTAACGGTATAAACAAATGGTGTAAAAAGTCTGGCAACTCCTGCAAACAGCTGTTTGCTGTGCCAATTTCCTATAGGGTCTTCGCCGCTTGTAAACAAAAACGCAGCAGTATAATAATAAAAATAATTGTTTAACCTATAAAATTGTTTGCTTATCTAGCCTAAT

>ID_194 P_194 Promoter_197 D1_Promoter for RE50478, -250bp and +50bp []

TTACTTACTCCCGTTCGTTCGCCGGTTCACCCAGAGAGAAATCCACATAGAAGCACACACACACACACACACATGCACAGATGTGCGCGGTGTATCTGTGAGTGCCAGCGTGTTCGGTTTGACTGATAGCGGAATTGGAACTCACGCGATACAGCACGCGCTCTCTCCGGCGATTTGCGAGTGTTGGTGAGAGCGGCTGGGTCCGAGTTCGATCATATTTATAAAAGAATCGACCACCGCCAGCTACTCAAGCAGTCGCAGTCGTTCGCCAGAACGTTCAATACCCGCTCGTCCGCTGAACAACTAGTATACGCAGAGTTTTGAAAAGACCTACGGTCAGAATCAATTTC

>ID_195 P_195 Promoter_198 D1_Promoter for RH52792, -250bp and +50bp []

GAACGGGTTAGCCTCAGACGGAGACCGAGTTTGAGGTCAACGGGGGGAGGGAGTGGTGCGGGGGCGGTGCGAGGGCGGTTTCTGGCGGCTTGGATATGGTGCAGCTGGCACAGCGACATTGACGCTACTTGCTGGTAGAACGGTGACGCAGTTGCTCCGCCAGACATTGCACTCGGATCGCCGGAGATCGTATCGCATCGGAACGGAACGGATTGGTTCGGCGTATTTAAACCAGTTGTGGAAGCCGCTGGAGGGTCAGTAGAAAGTCCAGAGCAGAGCAGATCGGTAACGAGCTGAACGGAGCGTGAGCTGTGAAATTTCCTTTCTTTTCCATCCAATGTGCAATTTGC

>ID_196 P_196 Promoter_199 D1_Promoter for RH01847, -250bp and +50bp [6 ]

GCCAGCTTTTAATTGGCTTGGATTTCGCCGCCCAAATCATTTGTCTGCATTGCGCTCGATGATTTTGTATTAGAATAAATTGAATTTTGATTCGTCGGCACCCCGACTTGCCTCCATGCGGGAACTCATCCCCACTAAACATCTCCCATTTTAATTACTGAACGCATGAGCGGCTGAGAGCGGAGAGAGAGGATCCACTATCGGGGAGCAGCACGGATCCACAGCTGGTACGGAAAAGTCGGCTGGTCCAGCATCATTCCTCGTTCGCAACTCGAGCTGTTAGGACGTCCAGCTCCAAGTTTTCGAGCCAAGTGATCCAAGTGTGTGTGCAAAACAGAGGCTACTCTTCG

>ID_199 P_199 Promoter_202 D1_Promoter for RH62576, -250bp and +50bp []

TATTTTACTATCGAAACATTCTTCTGTTCTGCCAATAAATACAATTTTAATAAGTATTTTGTAATACAATACAAGGTATAAATTAAAATAGTTTCAATCAATGTAAAATTAATGAAAATACGTAAATTTTAATTTTAGCTTATTGATAGTCGGAATTGTACTACGATTTAAATAAAGGTATATTTTAGGTAGGTATTTATTTAACATGTGTCAATGTTAGTATATTTTACGAGCCTAAACAAAAACGGTCAGTTTGCAGTAGCCGACTTCTTAAAACTGTCATTATTTTAATATTTCAATAACCCGGAGCAAATCCCAGGCAGTTAATCCGATCCGCAATGACGAACGAA

>ID_200 P_200 Promoter_203 D1_Promoter for RH57724, -250bp and +50bp []

TTTTGGGCGCGATAAGACCAGTGCTCCAAAGCACCTCATTAATTTTGGCGCCCAATTTGAAATGTGAGAAAACCCTTTAGTGAAATAAACGATTCAAATGAAATGTACAAACAAAATTCGTAAGCAAATTTATTTTGGGCAGACCATTTCATTAATAAAAATGTATTTCGAGCGGTATCCCAGCGAACTGGTTCCACCAGCGACGAATGCGCAGACTCGGATCTTGTATTTACACTCAAGCCCTGCCTTTATTCCATACTCGGCAAAGGAGACTTCAAAGTCAAACGTATCCTGCTGTACGCCGTGCGGCTGCGGCGCTCCTTCAATAAAAACAGTCGACACGCATTGTG

>ID_201 P_201 Promoter_204 D1_Promoter for RE59985, -250bp and +50bp []

GTCCAATCATAACAAAGTCCAGCTGCGTATTGGAATTTAGGATTCACTTCTTGAGAACGCTATCGAAAAGATCGAAATATCACCTCACACCTGAAATGATAATATGCCAGATTATAAATATCGAATGCCGGACGAGCATTAAGAACTCATACTGCTTAAGACTGGAGTGGTCAACAAATCTTATCACCGGACAATTTGGGCTGTGATTGATGGCAAGTGCTATAAATTAGACCCGCTACACCGATCGTCAGTTAGTCGAACTCGAGTAGACTAGAAACCAAGAGAATGTTCAAGACCATCGCTGTAGTAGTGCTCCTGGCAGCCCTGGCCAGTGCCGAGCTCCATCGCGT

>ID_203 P_203 Promoter_206 D1_Promoter for RE48413, -250bp and +50bp []

TTATCCGCTGAAAACAAGACCGAAAAATGTTAGGCTCGTTTTTCCGGCTACACAGGACACCTTTACTTTTAAAGTGGCGGATGCGGCAGCCGAATCCTTGCTTTTATTCCCTTGTTTACTCATCTTTTCCACACAATATGCACATTATCAGCAAATACGAAAATAGGAAAACCAACAATTTGCCAGTGTGACCGACGTCGTACAACCAGTGCACAGAATGAACAGCGAACAAATCACGATGCAGCCGATAGTTGCGATGGGAGTTATCGACAGAGCTATGTAAATGACTACTTTTTATTATAGTTTTATCAAATAAATGCGAAATGGATATCTGCCGCCTCTGTTTACGC

>ID_205 P_205 Promoter_208 D1_Promoter for RH08734, -250bp and +50bp [0 ]

TTTTCTTATAAAAAAGAGTTTGTTTCATAACAAGTATTCTAGCCCATATTTAGGTACCATTGCACTGTACAATAGTCTCATTTAAAATGTTTAGTACAATAATGCGGTTTTTGTTAAATAATGTGTTCTAAGGTTCAAGTCCTCTCTTATTTCTATTTAATTCATCTACCTTCGAGATTTGCATAATTATGGTATATTTGCGGTATATTTTAGTATGTTTTTCACACAACCACCCTGGTCACACGGCATCCTTCTTTTTCTTTCGTTTCCGGCGAGGTATGTGCACAAATTTTGTTTCTCCACGTTTTTCTTAAAAATAATTGCTTATTTCTCATTTTAGCGGTGATTAA

>ID_206 P_206 Promoter_209 D1_Promoter for RH35407, -250bp and +50bp [5 ]

CAACATTAAATGGAGGAATCAGAAAAACTTCGCAGCGTTAAGTAAATTTAGGCAATATGGGTTTAAAAATAAAAATGTATTTCTTTGTGAAAATGTATTACCATTGACAAAGTATATCTGAGCTAATACCCACACTCATTAAGTGTCGAAAGCACTGATAACCCACGTTGGACTGGAGACTAATTACCCCCAGCCGTAAATAACACATGCAACCATTTCTTCTATAAAAAGCAAAAACCATTGCGATATAGATTTAATTTTAAGCATGTCTAAAAAGCTGGTATTGTTGCTATTGTTTGTTGCCACTGTCTGTGCCCACAGGAATCGCAATCGTACGGCTCATCATGGTG

>ID_207 P_207 Promoter_210 D1_Promoter for RH63285, -250bp and +50bp []

CTGTAAGTGACGCCGCATTCTTTTCAGTTTGAAAGCAGCTGGATTTTAACTTATTCACCCAATTTTCGGAGAGCAATCCGCGGAAGAAATCATAACAAATAAATTCAAGTTGCAGGAATGGCAAACTTATCTGGTATTTTAGTTTTTTTGGCACAGAATATCTGGTCACACCAAAGCTCAGAGGAGTAACTTAAGGGATTTAGCTTATTTAGACGCTAGGCCCCTCAAAATAGTTTTGTCTAACTACGCCCATGCAATCGGCAGCTGTTATTGATTAGAGGAGCGATAAAAGTCCAGCCACCTCAAATATTGACACTAGGACAAGCTCGAGGGCCATTAAGTTATCTAAC

>ID_211 P_211 Promoter_214 D1_Promoter for RE28182, -250bp and +50bp []

TCTTCTTTATATATATATATATATATGAAAATATATTGTTTAGTTTCTAAATTAAATTGACAAGTTCTTTTAAAAGAGTATCCATGAACAGAAAGTTTTAAAATATTTAACTCAAAATTTGATCATGTGGTTGGAGTGCTGGCTAAGAGCGCAGCGAAGTGCCCAACCTCGAACAAGGAACCGTTTTTTCGGCAACGGTCACTCTGTTTTCGATAGAAATAACTTTTCCTCGCAGGCCTGGTATTTTTTGGCATTTACCAGCAGTGCGGCCGCATTGAATCGTTAAAAAGTAAAAAGAGAAGCAACAGCGTGAGGTGTAAATAATGTAAGACAATGCAATATAGTAAAGG

>ID_212 P_212 Promoter_215 D1_Promoter for RH58138, -250bp and +50bp [4 ]

ATAGTGAGCTCTGTTACCGATGATATGGAAATATATAATTGTAATATTATTTGTAAATTGTACGGCAAAATTTATACCATGAAATATATACGGAATAGAAAAGACACGAGAGTAAAGAGGAATTATTGGAGGAGCCAATGGATGAAAGTCAAGACCATCTCGCGAGTAATGCTTGTAATCAGAGTTCCCGAAGCATCCGGCATCGGGTCGGTCGCTCGACTTGAAGATAGCTCTATCGCCTCTCCGGCTTGATTAGTCTGTTTTTGAGCCTCGCTGGAAACGGTCGTTGCTGCAGTTGAGCTTTGTAATCGAGTCAAGTGATAACGCGGGAAATAAAGCACTGGAGATGG

>ID_214 P_214 Promoter_217 D1_Promoter for RH41676, -250bp and +50bp [4 ]

GTAAGTAAACTTAGCAAGCATTATAGCTAATATAATTATAGCTATTAGAAGTAACTTACAACCAGCCTATAACCAGAAACCATATAAATAAATAATATAATTTTATACATAAGCAAAATATTGCATTTTAATCGTAATCTTTTCCAATTTCCCAAAACCAATAAGCAGTTCACATAAATAACCCCAGATAATGCATTCGCAGCCGCCAGGCAAACATAAAGCTCTTGATTTCTAGTTTTTCCAGCTCAAAATCATTTACAAATCTACTTTAAACGCGATCAGTTCGCAATGGGCACGCCTCGTCTGACCGTAGTACACGGTCTAATCCTTCTGCTCCTTGTGGAGCTAGG

>ID_215 P_215 Promoter_218 D1_Promoter for RH08410, -250bp and +50bp [10 ]

GAGATTAACTATTTGGCGGTTGCATAAAGCGGTTATCTTCTGTGACTAAACAAATGTAGACATTCTTGAGCCAAGCAAACGCTCAGCTGTTATGCGAAGCAATCTATTCCAGCATGCTACGGAAAAAAGTACGCTTATCACGGGTCAGCTCCGTAGCTCTAAACGCTCGGCCTTCCTAGTGCCGATAAGCCGGTCTCAAATTCGGCCGGCTGCGGCGCAAAACGAGGTGTCGTTATGAGTAGTTTGGCAGAGGTTCAATCAGTACTCAAGTCATCGTTCTCTAGTTCAGAGTTCCGCAGCAGTTACAAAAAACCAACACAAAAATGGCCAAGCTCGCAATTTGCATCCTC

>ID_217 P_217 Promoter_220 D1_Promoter for RE63452, -250bp and +50bp []

TGGAGATCTAGTTCTAGACCAAGTGTGGTATGCTCGAACTAGACAGAAAGTGACAACCTTTAAAAAAAAGTTTCAGTGGCAATAAAAGCTATAGCTATAGTAAATAATAATTTCGAACGTATAAAGTAAACTTTGAATTTCCCGCGGAGAATATATTTGTAGTATATTTAACCACGTTTTTGCTACGGTATTTATCGCCATACGGCGTAGGTTCAAAAGAACTACGTGTTATCGATAACACGAAGCGTTATTTGTCTGGACTTATCGCACCACCTATCGAATCAGCTGCAGCTGAAAATTAGACCTAAAAGTTATTCTAAATGTGCTTAAATCTGAATCACGCTTGACAA

>ID_218 P_218 Promoter_221 D1_Promoter for RH24023, -250bp and +50bp []

CGATAGGATACAGTGCCGCAACGGTCTGCTATCGATAGTTGTAGTTTCAAAAAAAACAAATAGAACTATCAACAGATTTTAAATTTGTTAGCTATGGAATAGGAATTTACAGTTAGTACTAAGGTAACTAGGTGTTTCTTAAAAATGTTTAGGATTTTTGTTTCTCAATTTTTATGAACTTAGGCAGCAATCGTTACAAAGTCGATAAGAACAATCGATGGTCCGATAGCTCTCACCCGCGCATCGAGGTGCTGTTGTTGTTTTTTGTTGTTGTTGGTGTTGTTGTGGTGGGCTCGTCCGTGAAAGATCACTTGCTTGCAAACTTATTTATTAATTACCGACTAAGGAAT

>ID_219 P_219 Promoter_222 D1_Promoter for RH03078, -250bp and +50bp [5 ]

ATTGATATTTCGATTTGATAATGTATTTAAATGTAAAATTTTAATCTAGTTTCTTGTGGCTTAAATTGTTATGCAAATCAGCTAGTTGGACAAAAATAGTTTCGTCTGGACATAAATTTTTCTTTCCGTGTGGTCGGTAATCATTTTTTCGTTCCGTGTGGTCAGTGTGAAAAGAGAGAACGACAAAAAGAGAGAAACCGCTTTTGTGTGCGTTGAGCGGGTTTTCTTAAAAGGGCGATCGACTGTTTTTGAGTCACTTGTGAAAAATCAGTCATCGCGAGCAGTTCGACTAAGGAAAACCCGCTGGCACACACAAACCGTAACCACTTATCAAACAAAAACAGCTGGCC

>ID_220 P_220 Promoter_223 D1_Promoter for RE38428, -250bp and +50bp [9 ]

AGTATTTTTCACTATTTTGTGGATAAAATCAAGTGGTATGAAAATGTATCCATGCGATAATGTTCCGTTATCTTCAATTGCTGAAATAAAGTCAAATAAACAGTTTCAATTACTGCTAAAGTTAATTAATGAAATGCTAGTTAAGTATGTCTTAAATTATAAATATTTAGCTACTCATGAGAATTTTTTTTGTTTGGCCCACTGTGCATAACCCGCGCGTGTAGCCCATTCCAGTATTTTACCATCTTGTACATTAGTATTTACCCGTCTTCCGCTACGGTCACACTGCCATATCGACGCAAAACAAATTGCGATTTTCACAACACAAGAAAAGCAGGAAAAACAGCAAA

>ID_221 P_221 Promoter_224 D1_Promoter for RH42548, -250bp and +50bp [1 ]

CGGAGCGATCTCCCCACATGTCCATGCAAAGATCGTGCTTGTGCTCCGAGAGTCGCGCACAAGTCAAGTGGCACGGGCTATGCGAGGAGGAGGAGGGTTAGGGTAGGAGGATCGTGTGCCGGAGAGGAAGTGCCGGCATTATCTCGGCTAAATTCGAATTCGTTTGGTTACGCTGCGTGGTCTTGGCCAAGAGCTCTCGTTCCTCATTTCTCGGTTTTCAGTTTTCGGTTCTCTGGCGGCCAAGAGCATTTAGTTGCCTTAGTTGTACCATGCCGAGCGGACCGCTGAGTTAGGATCGGTTGAGAAGTCTGGCATACGAGTGGAAGTGGAAGTGTAGATAGGATAGTGAC

>ID_222 P_222 Promoter_225 D1_Promoter for RE11434, -250bp and +50bp [6 ]

GCTCAATTCAATTAGTTAGGAGCAACGATTCCGGCAATATTCGCGCTAAACGATTTCAAATTGAATGCTGAACAACCAGCGCCGGCAGAGGCTCGACAGAGCGTCGCTCTGCATCCCCGAGATTCCGATTTCGATTCCGAACCCAAATCCGAAGCCGAATCAAAGTCAGAAACAGAATCTTTTCCTGAAGCAGTACGACGTCGACTGCGCCGGAGCTCCAGTTTATAAACCCTCGTCCACCAGACGGCAGCGTTCACTTTACAATCAGCGGTCGAGCAGGCAACAAACGTGAAGAAAGTGCACATAGTACCATACCACATATATGTGCATACCATATCATCGAGTTCTGG

>ID_224 P_224 Promoter_227 D1_Promoter for RE42318, -250bp and +50bp []

GAATATTTGCGATTACTCTTATAAACATAAGGGGGAAACATAAGATTTTAATTTAATGACATTAATCTGGAAGAATAACAAATCTCATTATCCTGAGCCCTTAAAAATGGCAAGCGGTTCAAAATTATAGAGCGAGGATTAATCAGATAAGGCCCCTCTTCTACGCCACTGATAATAAAGCTTGCATGCCATGGATTTGGTATTTTCCGCGAGTTCCTCCATGGCGATTTCCCAGATTTTGGCCACTTCAGTATGCGGTTATTTCTCACGGTTAAAAGTTCTGCTCTCCGAGTTGATTAAACTGGCTAAAGTGATATTTTATTTACTGTTGTGATAAACGCGTGTGAAAA

>ID_226 P_226 Promoter_229 D1_Promoter for RE59788, -250bp and +50bp [2 ]

GCCGAAGTCAACGGCTACAGGTGCTGCTCGAGCGTGTGTGTGAGGTGAGGTGGTGGCATAATCTTGGCCTTCTTGGCCATTTCGTCTCTCCATTTCTTTTCGTTTGGTTACCTTTGTGCGTCGGATCGGAAACTCCACTCCGTCAGTCAGTCGGTCAATCAGTCAACTTTGGCTCAGTCGCGTCGCTGCCCGGGTGAGCAGCTCCAGCAGCGGCATAGGTAGGTAGGAACTTACACATTTGGCTACGATCATTTGTCTACGGAACGGCGCCGCGTTGAGTTCGTTAGACGCGAAAAGGAAAGGAAGTCTTGAGACGCCGACGAAAACCCTAAGCGAGCCGCGTTCGCAGA

>ID_228 P_228 Promoter_231 D1_Promoter for RH23459, -250bp and +50bp [1 ]

TTTATATATTTTATATATATATATAATATATATATATATATATATTTTATGCATGTAATTATTATTATTCGCTGCCAATAAGATATGTCTATCATTTGAAGTATGTATGTTGTCAGGCAGTTACCTATTTTGCTGACCTATGGTGTGCTTGATAATGATCATCCGGCAAAATACTTTTGAGTATTAAAATGTAATCAAATGCTGTCGGCGTCTCTCCTGATTTAAGGTGCTATCACCGAATTTCTCCGATTAGTCGCACGGCATTATTCGCTCGGACCAGAAATACAGCGAAGCCCTCGAAACAAAATATTAGTAGATCCCGAATATAATCCTAGCAAATAGAAAAGAAC

>ID_229 P_229 Promoter_232 D1_Promoter for RH21615, -250bp and +50bp [5 ]

GGATTGTGGCTGGCGTTTTGGTGGCTTGGTTTGGTTGGGTTTGGTTTCCCTTGGCTTGGCGCCCAAATAGGTCGCTTCTCTATCTCTCGAGTGGATAGGAAAGTTCCGCCGACCACTGGGCTTCCAACTCGATTGGCAGCTACGAAAATATATATATATAAAAAAAATGGAAAAAAAAAAAACGGAAAATAACACAGAAGCACCACAAACCCTCGACGAGGCTATATAAGTTGCGGTGTCGCCGATTCCCTAACCGCAGTTGTGTGACCGCGTTCGGCGGGTGAGCACGGATAGTAAGACAGTGTCTCCACTGTCAGCTTAAAACCGTTCGAAAGCCACTAGATTTGTGT

>ID_230 P_230 Promoter_233 D1_Promoter for RH05643, -250bp and +50bp [8 ]

TTGTCATTAAAAAAGTGGCTTAAAACTAATAGTATGCCTGAAAGCAGGGTATATTTCTATGAAGTACATATAAATGTGCAACTCAAGTAGCAAACGCGTTCGAAAAAGTGATTAAGTCGAGGAATAATCCCCACTCATGGCCGCCTATCTCCGTTCGATAGTTCACTTGTTGCGCAGCCCTGGCAGCCGGAATGCAACCGTGTGCGTGTCGGCGGAAGCGGACTCTCTGGCGTTGCTGCACACTGCCGTAACATGTTCTTTTTCAGGCGTTTGCCTTTGCGCTGACATCGTATCTCGATTTCTTCTGTGACAAAGTAAGGGATTTTCAACGTTTAATTGTGTGGATTGTG

>ID_231 P_231 Promoter_234 D1_Promoter for RE70055, -250bp and +50bp [2 ]

GTTTATGTAGGCGTCGACGTCACTTATTGACGAAATGTAACTTTTCAACTAGTAAAAACTTTAACAAAGCTGGGATTAATTTGTGAAAAATAGAAACTCAAATCTCTTGGTTTTAAAGAAATAATTATTTATAGGTGACATTTTTATATTCGTTTTATTACCCACCTCACTACTTTTGCACCTTGCAGGACTGCTTATCGCAAGCTAGTGCGCATACTATCGATGGCTATCGATTGGCCGCAAATAATCAATGTTTCATCTCTAGCTCTTTTAAGTTTTGGTTGCTATTTATGTAAATGTTTAGCGAAATTGCGGAAAAAGTGCATATCAGTGCGCTCGATTGCTGAAAA

>ID_232 P_232 Promoter_235 D1_Promoter for RH44395, -250bp and +50bp [7 ]

ATTTGGATGTTATAGAACACTCTTAGTTTTAGCCTGCGTTTGGTTTTGCCCAACTTAAACTTTGGTTATAATATATATAATATATGGAAGTAAGTACTTTTTTTCAGTGTGCGGTTCCCACAGGTCTCTTTCTCGCCATCTCTGTTGCACTCACCGTACTTATGCGATTCATTTTAGCTTATAACGCCGAACACGGGGAACGTTGAGCTGCAGTTGTAGAAACGTTCTCAGCACTCAAATTTCGCATTCACATTCTCATTCGTCAATTAGTTGCCGGCGCGAGCGAATCGTGAGTGCGGAAAAAAGAAAATCTTTTCATCAGCTGGAAAACTGTTCTTCCCGATCAAAAC

>ID_233 P_233 Promoter_236 D1_Promoter for RH50620, -250bp and +50bp [2 ]

ACATATGTGGTACGCAAGTAAGAGTGCCTGCGCATGCCCCATGTGCCCCACCAAGAGCTTTGCATCCCATACAAGTCCCCAAAGTGGAGAACCGAACCAATTCTTCGCGGGCAGAACAAAAGCTTCTGCACACGTCTCCACTCGAATTTGGAGCCGGCCGGCGTGTGCAAAAGAGGTGAATCGAACGAAAGACCCGTGTGTAAAGCCGCGTTTCCAAAATGTATAAAACCGAGAGCATCTGGCCAATGTGCATCAGTTGTGGTCAGCAGCAAAATCAAGTGAATCATCTCAGTGCAACTAAAGGCCTAAATAGCCCATACCTACCTTTTTTGTAAACAAGTGAACAAGTT

>ID_234 P_234 Promoter_237 D1_Promoter for RE60675, -250bp and +50bp [7 ]

TAAATCGAAGCGAAGCCCCAGCTCACGTTCATCAAGTATCAAATGAAAATGATTAACAAGTACAAAAAGGAATGTAAAAACCGAATTCACATACGTACATATGTAATGAGATAAGGCAGGAGTGATGTCAGAGCGTACATACATATACATATAACCTATAGTGCTACATTACTACAAAGAGAAGCAAGCTTCCATGGGGTTCTCCACACACTGCTCTCCGGGTATAAAAAGCACCCGCCCTCAAGCAGCGGACTTTCAGTTAGCATCAGCCGCGATCCGCGAGTACAAGTGCCATACCCCATCGACTCGCGAGATACATACATACATACTATCGCCAACATGTTCGCCAA

>ID_236 P_236 Promoter_239 D1_Promoter for RE18078, -250bp and +50bp [4 ]

AAATAAATGTAGTATTAAAAACAATATATTTAACTAAAAATTCTGATATAACCATATTTAAGCCATATAACTTAAAGAATCGATGGACAATCCGTACACATTGTAGGGCACTTGTATATGCCGGCCGGCAAATGATAATTCAGAGGAGGACCACAAGCAAAGTGAGAATCTGATGAACGGCAGTGCAAAACAAATCGCTGATAGCCAGTTATCGGGGCTCTATATAAATCGGCATCTGGCAGCATTTCAGTTTCAGTTTCGCGACGTCTCCCAGACAGCCGGTTTCTATTCCAGTTCTTGAAGGTGTATATATCGCTCAATATGGCCGATCCCAACGCCAAGCCCAAGTA

>ID_237 P_237 Promoter_240 D1_Promoter for RH09938, -250bp and +50bp [5 ]

ATTCACGCACGCATACAATCTTCAAGAAGGTTGGCAACTACAAATATTCGAAGTCGAAAACGGCGTTACCAATCGTCATAACTTTGGAGGTTGTACTGGCTAAAAGATAGTTTCGGTGCGTGTCCACGCTAATTGTGGATTGTGTACACGCTAATTGGTATTTTAACCCTATTTGTATTAATTGGTGGTATTTTCTCGCATCCCCCCGTCGGTCACACCAACACAAACGTGGTACCCATTTCCGCCTGTTCTTCCTCTTTCTTTTGCCATTGTCAGCCGACGAAGGTGGGTGTTTTAATTTTTCATCCGGCTCCACAGAATCACTCGACTAACTAATTTCTATCGCCTGC

>ID_238 P_238 Promoter_241 D1_Promoter for RH09052, -250bp and +50bp [7 ]

ACAATGGTATATTATTTTATGCAAGCAATTAAAAGTATTTCTCTTATCATTTTGCGGCTCAACAACTTTTGCGGTATTGCACTTTTTGCTTTTTCAGAAGCTATCGACTAAACCACAACGTTTGGATTTGTGAGAAAAAAGGCTCGACCATTGCAGTTTTGCGGTTGGGACTTGATAATGAATATTTTTAAAAATATTATTTTTATATTCGGACTGCTAGTTAGCTATAAAATGAGTTCGGCTTCCTGGGAAAATCACAGTTCTGCAACTGGCTTCGTTGAGTGAAGTTAAAAGTGTAGTTGAAAGTGTACATCCATAGAGAAAACCATGACTAACACGGATCTGAAAGC

>ID_239 P_239 Promoter_242 D1_Promoter for RE54756, -250bp and +50bp []

GTCTTTATTTGCATATTATGTTAGTGTTGGTACGGCTTTGTACGGGTGGCTTGATTCACCAAGCCAAAACAAAAATAACATTTGTTTACGTTTGTACGACTTTATTCATTCGCTGAGAGAGTCTGCTGCCAGCACGATAGCCAGATGATGATGATGATGATTATCCAGATGATGAGGTTCCCTTGTGCGTGATAACGACCCGACTCATTCTCATTCTCATCGACTCGATTCTGTCGAGATTTGCCCCCAAGCTCAGTAGTCTACGAATCGCTGAGCCGTCGACACGGTTTAATTGGACTGAAAGCGCGGATTAGGTCCGATAGAGTCGCAGTTCGGATCACTTCCTTATC

>ID_240 P_240 Promoter_243 D1_Promoter for RH08745, -250bp and +50bp []

CTTAAAAAATGCTTCTTACCAATGATTCTACCGATTTACCTGAAATTAATAATTAATAATTAAAATAATAATTTATATAAAATTCTTATAAAAAAAAAGTCTGAAATATGCGTACTTTTACCTTCTTTGAAGACTTCATCTTAAGTCTACCGATTAAATGCTTGTGTTTTCCCAAATACACGGCTTATCACCAATTCGCGTGGCTCTCTAATCCGACGCTCGACTATATAAGATCGGCTCCAACTAGCCGAGATCATCAGTTCAATCCAATCAATTGCCCAGTGCACTCAGTATCCAAAACCGGAGAAATCCATCCAGAATCAATATGAAATTCTTCTCAGTCGTCACCG

>ID_241 P_241 Promoter_244 D1_Promoter for RH69634, -250bp and +50bp []

TTTACTGATTCGAGTATCTGTTGAACTTTAAACCGAAATGGTAAAAAAAAGTGAGTCAGTTTCGGGTTATCAACAAGATCGGAGAGGGTTTAAGTAAACTAACCGGTGTATTGCCGTGTGTATGTGTAATAATTGCTTATACAGTGTGATCAGCGGAGTAGAGCGAAAACAAAAAGCGATAATCAAAACAAAACTCTGACGAAGCTGCATGCAAAATCGTATAAAAGGCAGATGCTAACAGAATAAATTCAACAGTTTCGATACCAAAGGGTTTCCAACAACATCTCTACCGAAAATGAAGTTCCTGATTGTCCTCTCCGTCATTTTGGCCATTTCGGCTGCTGTGAGTT

>ID_242 P_242 Promoter_245 D1_Promoter for RH52349, -250bp and +50bp []

TTTCCAACAAATGAGATTTAGATTTAAATTAAGGGTACGTTTTCCAACTCGATGAATTGAATTTAGGAATTTTATTCCGAAAAGCAGCGTTTAATGTCTACAACATTGTTTGTAAACTCTACAAATTTCCTCCATGTGAGATGTCTCCATATTCCAGGGTTTCGGTCACCGCTAATAGTCTAGCAAGGGTTTCAACCCGTTGGCCAACGCAATGTTTTAACTCGTACCTTGTGGTACACTTGGTCACACTGCTGTTAATGGACACCCAATCTATCTCAGCTGTTTTACCTTGACTTTGTTTACATTTTTCGAGTTTATTAGCATTTAAACAAAGTTTAATCATAAATAAA

>ID_243 P_243 Promoter_246 D1_Promoter for RE45145, -250bp and +50bp []

AGACCCTTAGATTTTGCAATGCTTACGATTTCACAATCAAAGTTAGCTTTAAAGAATAAACCTCCCTTCTTTAAATGTTGTTATCAAACTGACTTAAGGCAATTGAAAACCAGTTTCGCTGACTTAAAACCAAAAAGCTCGGTAGTTGGCGGTATCGGTGGTTTGTCTTTTGGCTTACTATCGATACACGACTGAAAACCCACTTCAAATGCTTCGCAGCACTTTTCGAGCGGCTCTCCGCCGACTTTTCAGTCGTTGGCGGCATTTCGTTGCGTTCGGTTTGGCCATATTCACTCTGTTGATTGGACAAGAAGTACGGAGCTAACGCAGCTCGGAGTGTTTTTCGAATA

>ID_244 P_244 Promoter_247 D1_Promoter for RH56077, -250bp and +50bp [7 ]

CACAAATCTGTCAAAAATATTACCCATACGCCAGGTTGGTCGCACTATTAAAAGTTCATAATTAGAAATGTTTACCTAAAAGATGGGTTGACGAGGGCCTAGCCCACTGTGCCCACTCATGGCGAACCAGAAACTGACTTGAGGAGGTTGACTTGCTGCTTGACTTGCCACGCGAGAGAGAGGTAGAGAGAGAGAGCGGAACGCCAACGCCGACTCTACGGTTTGAGTTGTATCTTCTCGACCTTTTTTTTTGAATCAGTCTTTTGGAATTCGCGAAACAATGCGGACGCCGTACGAAGAAAGCGCTGCTGCCGCCGACCACAAATATCAATACCAATACACCGCGGCCA

>ID_245 P_245 Promoter_248 D1_Promoter for RE17462, -250bp and +50bp []

TACGTGATTAGAATGCCGGAGAAACAAACACTGTCCCAAACATGGCCTTATCCAACTTATCCAACTGGAAGCACGCAAACAAGGCGGCCGCAAAAGTTTAGCTTACGCTTTGTTTTTTATCATTTGTGATGCTATTTTTGAGTTGCTCATTATTTTTATTTTACTCCCCGCTGTTATTTATCGCCAAGCTGAGCAAGTAGCCGGCCTCGCTTTTGAGCAGGGGATCCGATTGTAATGTCAGCCAGAGCTCAGTTCCCAGTTGACAGTCGAGCGAGGCGAAATGCAATCTCAGCTGAAGCGGAACACTTTGTTGCTACTGCTACTTTCGGTGGTGATCTTGGCCAGCTGCA

>ID_246 P_246 Promoter_249 D1_Promoter for RE23622, -250bp and +50bp []

ATTTTCAACCCATGTTCTAAAGGTTTTGGAAGTTATACCAAAAAGCATAAAATGATTATTGGTTGCTGAGAGTAATCAATGGTGTATTTTGACTATATATAGCTCAAAGTTTACCGTTTATTTGAGCGTACATATGTATGTATTAACAAATTTAAGAAAATATATATATTAAAATTAATATATCGATACCCGAAAATATGCTAAGCATATTGCACATCGATATTTCTAACTGTATGTTTTTGACATCCCTAGTTCTGAGTAGAGCTGGCAACGCCGACATTGTGCAACTCGAAAACGGCGTGAAATACGTATTAAACAGCATTTATTGGCAGAAATCGGCGCATATAATG

>ID_247 P_247 Promoter_250 D1_Promoter for RH74493, -250bp and +50bp []

TATTTAAGAGCTTTTCCCCCATTTCTCTCTGCCTTCTTATCAGCGATCAGGTAGTGTCGCGGGAATTTCCCAATCTACACAAAATTTACATGCACTTTGCTACGTTTTTTAGGAGGCACGGTAATTAATGTAACGCCAGCCTTTTTGTGAGCCAATTTACACGACACTAGCTATTCAATTTCAATTGCCATTCAATTTCAATTAGCTTAGCTGCCTGGGCGGCCCTGGGGAAATTCGTCTAAGTTATATCCAGTTGTGGAGTCACTGCCGCTATGAGAGGTTTACTTATGATTCTGATGCTCTTCATCTTCGCCATTCTGACGGTTCGCGCTCTAAACTGCCAGCCGTGT

>ID_248 P_248 Promoter_251 D1_Promoter for RE47665, -250bp and +50bp []

GTTTTTCTATGGTCTTCCAATGGATTTCTTATTTATGTATTATAAGTACTTAAATTTACTTTTTTATAAATAAGTTTTCTGTATTATTGATTTTTGTGCACTTATTTATTTTTAAATTCTTAAAACCCATTAACTATAAATCGTTTTAACTGTTACAAGCCAATAATACAATTTGAAACAAATGCTCCCATATCACTAAAGAAGAATCCTTAAAGTTTCCTGAAAAATAATAGGTGCAACTACTAAATGGCCGTCAGCCGGGTGATGGACAGGGGGAACTACGCGTACCATTCGGAGCTACAGTGCCCGATGGACGTGCCGTAACTGTCTGTACACCCGGAAGTGCGACT

>ID_249 P_249 Promoter_252 D1_Promoter for RH07821, -250bp and +50bp [11 ]

GCATAGCCGCTCTTCTCTATTGCCAGTATCCCTGCAAACTCTTCAATCCTAGATATCATTTTCATATATCAGGTAATCCTTAATTTCTCGTCTCCTCAACAACCTCGACATTATCAAAAAATTCCATAATGAGATTTAAATTACGCCGGCGAGTGCAACTCAATTGAGCGACACTCAAAATCGCCCCACAGTTGAGTGCGACATACCCACATTGAGCGGGGCTCGAAGGTATAAAAGCTGGAGCAGCCCAGACCACAGTCAACAGTTTCCTCCGAGCGAGCATTCAGTCCTCACACAATCTATCACAGAAGCCAAAAAAAATAAGATTCACCATGAAGAACGCTGTTGCT

>ID_252 P_252 Promoter_255 D1_Promoter for RH03777, -250bp and +50bp []

TCGATACTATTCGCGGTCTGACTATCGATAGTTGAATGGGATGGCTAGAGCATTGCTCATTTGGAGGTATTACGACGAATTTGGAGAGACAAATGATTTTCCTCTTACCTTAGTTTAACATATTTCTCAAAGGTAAGCCAAATGGAGCAAAAGCTAAATATAGATAAAATTTCATCGATATATACCATCACTGCAGGTATCGATACATATCGATGACTCATGTGCTGTCATCGAAATTTTATTGTATTTACTTTATTCAATTGGAGTCACTTGCAGCGTTTGGACACCAAGGAGCTTTTCGAGGAGCATAGCGAGAAACCAGATAAGCCACCATGTCCACACAGCAAGCG

>ID_254 P_254 Promoter_257 D1_Promoter for RE03093, -250bp and +50bp [0 ]

TTTTGCCGCCCGCTGAGAACTAGAAACTTGTCGTGTCACACTGAGAAAGCATTGTACGATAATCTCATTGCGAGGAATCCTCCAGCCAACAGCAAACATATATACTGATCGAGCTACACTATAAGGGAATCGCTCGACTCGAGTGGATTTGTTTACGCCCGAGCCCAAAGATACGGTAATCACCCCAGCGATATGGCGAAATTGTTTGCCAACCTAACGACTATAAATCGGCAGTGGACTACGACTAGTCAGTTGCGTTGTAGACCGCTAGAAGTGCGGACATAGTAAACAGTATAGATTCAATATGATCGGAATATATTTGCTGATCGCTGCAGTCACGCTGCTATATG

>ID_255 P_255 Promoter_258 D1_Promoter for RE51323, -250bp and +50bp []

ACAACATTGACTATTTTGAGTAAAAACAATAGGTTGGTCATAAAAAATAATTACTAATTTACTAACCGCGGTCGCCGCATGTCTGAACAGGCCTCCAGCTTTTCCGGCTTGAGTATCCACTGATTCACCCGTGTCTCGAACCCAGAGTTGCCTAAACATATTTCGATGTATACATAAAGAGGGAAATTTATTTTTTAGGCTTGGCAATGCCGCTGCGGGCATATAAAAGTCCGCGCAGTCGTGCGCTTGTGTGAGTCATGGTGGAAAATTCGAGGCAAGCGTACCGATGCGCAAAGAACAAGCATAAGCGAAGAAGATAAAGATATCGATTAAAATTTCTGCTACAAAAA

>ID_256 P_256 Promoter_259 D1_Promoter for RE61692, -250bp and +50bp []

ATCGTTTTGCTTTCGATAAGTCACGATGGTTCACGTCTTGGAATTTCTACATCTATATGTAAAAAAAAAAAAAAAAAAATTATCAGAATAGTGAAACAGTACAACCAACTTTAAAGTTGTAATAATTAAAATTGTTTGTAAATGTGGTTTAATATCATGTATACCATTTATTTTATAAATATCCACCAGCTGGTTGGACATCTGGCAACTCCTGTCATTACATGCTTTCAGTGTCGAGCGTTTTCCAGCACTTGCATGTTTACCTTAAGAAGAAGAGTAGAAAAAATCGTTGTGTAGTGGCAAAATAAATAAATACAGATGTGAGTAGCAGTAAATAAAACATACAAAGT

>ID_257 P_257 Promoter_260 D1_Promoter for RE22770, -250bp and +50bp []

CTTCTTTCTTTTCTTCCCCTCAATTCCCGCGCAAAACGCTTTTTTCCAACGAGACAGTTGCAAAAAGCAATCAACTGTATCGTGGAGTGTCCCCATCTCGCTCGCACTCACTTGTTCTAGCGCACGAACGATCTTCGGTTTTTCGAATGCAGCAGAAAGAACAACAAAAGCAAAAACTTCGTGCTCACACACACACGCAAAGCCTATTTCTTTTTTCGACTTTTATTCGAACTTTTTTGCAGCGATTTTTCAGTAACAAACGGCAACTCTCAGGAATAAAATCGTCGTCGCTCCGCTCGTTAAATTCTCGTTGCTTTTCCACCCACATAAAAAATCAGGATAAATTGTTA

>ID_259 P_259 Promoter_262 D1_Promoter for RH51123, -250bp and +50bp [1 ]

CCCAGCGGCGCTGTCAGCGGTTCTAATGACGCCGCACTCCGTGTACCGCTTGACCCCGCGCCCGCGACCACTTTCGCGGTGAAGGGGGGCTAAACAGTAGTTTAGTTCACTGGCTCCGGCGACTTTCACGCTCTCCGGATACCTATTTGTTTGCTCAGGTTCTCTTTCGCTGCTGGCGCTCGCAAGGTGCGTGCTGTCCGCTTCGCAGTCGCTGCCGACTTTCAAGTCAGCAGCGCTGCCCCGAGTCCGTCAGTCTGAGACCGTCGGACTTACCATACAGTCGACGCTCCAATACGTTCCGCGCTACAACGATTCAACGATTGAACCATTCGGGATCCATTCCTCGATTT

>ID_260 P_260 Promoter_263 D1_Promoter for RE41295, -250bp and +50bp [8 ]

AGGGATGCCACACGGCAGGCTGTACTGAATTTCGGGCAGACGACATCGATATTCGACATCTGTTTATTCACAGCCAACTTTAATGTGGTTTTACGCCATACATATGATGCTCGTACAGCAATTATGTGACTGATGCAAGCGAGAGCATACAAATACGGGGATATGGGTGATTTGTAATTCCACCCCCATCTGAGCGCAGTCCAGGTGGCAACGACGCTGCCCCCGCATGCCTATCGACCGATATACCGCAGATTCGCCAGTTTTCCGCAAGGTGGCGACTACGGCGGGCGCAGACTAAAAAGACAGACTTTTTTCTATATAATATACGGCAGGAATCAGTGCAAAAACCG

>ID_264 P_264 Promoter_267 D1_Promoter for RE49835, -250bp and +50bp []

GAAATATGAATCGGTGAGCTAGTGCATAAAAAGTTTACTGGGTAGCAAAAATATATTATTTTAAAATTCGTTGTATACTTATATATCAATTTCTCTCATGGCCCTAACGGGAACTTATTCTTTAAGCTTAGAATAAAAATCATTGCAAATATTAACCATGTTAATATAATATAAACTAAGCTGTTACTTTAGGCGATAGTTCTGTACATTCGATACACACACTGTTTACGTTTGGGTATTTTCGGCACGCGTTAGTACATTTTCATATAACTGCTGTTTCACCCCGAAATTTTGTAGCTCGTTGTTATTGTTAATTTTATGGACTCCGCACACCGTACTTAATGAACGCC

>ID_268 P_268 Promoter_271 D1_Promoter for RE37019, -250bp and +50bp []

GAAAATACTCAAATAAAACATATCGATTTGGCATACCCCACTAATTTTTTGGCCCCAAGTGTGTGAGAGTGTGTGAGACGAAGCGCGCCACAAACATAAAAAAGCGGTGAAGTGAGCGGTTGTGGAACGTGAGTGGATGCTAAGAGCAAGCTCTCACATACGCGGACATAGGTCGCACACACACACGCACAGACCGCCTTTTTGCGCCGCCGAAACGAACACTTTTACGAAGGCGACGGCGAATCAGTTTCAGTTGTCAGTTCGCATCCAACTAGAAAGCAGTTAACGAGTAGTCTGTGTTTTTTCGCTTGCGGTTAAAAGCCACGAGGTCGTTCATCGTTCATCGTTTT

>ID_269 P_269 Promoter_272 D1_Promoter for RH66775, -250bp and +50bp []

CATCGCGACTCCTTGGCCAGCTACATGGGTCACTACGATATTCTCAACTACTTTGCCATAGCGGAAAATGAATCGAAGGCACGAGTGCGATTTAATCTGATGGAGCGAATGCTGCAGCCATGTGGGCCACCACCAGAAAAGCTAGAGGATTAAGAAAAACTATTTTAATAATAAAGAAAATAATTGACAAGACAATGTAATTTTCCAAGGGTGGATTTTCGGATAAATTCCAACTTCTGTAGTTATCGACATTCGGTATTTTGCGCTCAAGCGGCAAACGGTCGCACTGCAGGTCAGGAAAAGCTTTTCACTCGATATAATATAACTTATTTTCGTTTCAATTGTTCATT

>ID_270 P_270 Promoter_273 D1_Promoter for RE03815, -250bp and +50bp []

GTTCTCTTAGGGAGAATGTGACATGCGTATTGCATTGGGAGCACATCCAAAGAGACAAACACTTGTTTATGTTTTTGTTTGTCGGTTGTCGTTCACCTCCGACATCCACACACATACACGCACACAAAGGCGAACAATTGTCTAAAAAATGAAAACGAATTTTCAGATTTACATATTTATTTACCTTTCTGTTGTATTATATTATTAAAGCTTTTTCGGAAGAGCGACCCACGGATGGAATTCTCGACTCAGTCTGCATCGGGTTATCAGTGCGAATGGGTAACGGTTCTCCCTCTATCGGGTAAAAAAATAATGCTGAAACGGTTCAAAACGTAGTCTCCCAGGGATTT

>ID_271 P_271 Promoter_274 D1_Promoter for RH53151, -250bp and +50bp [2 ]

CGTTTTTGGCCTCCTTCACCGTGCGGTTGGCCATTTTCAGTTGAACCCTTGTTCCTTGTCAATCGTAACTCAAATAGGACTTTTCAATCCAGAAATTCGGGTATCTATTGGCAGCAGTGCAAAATGTGAAAATGCACCGAATATATTTATTTATTTTTCGGCGTTTGCTAACCAGAGTGGCCAGCTGCGTAAGGGAGAAATATGCCACTCCGCCAAGCGCATACACAAAGGCGATAACGATAGGGGACGGCAATCGGTATCGGGTGACGCATACGAACAACAGCTCCCAGACAACCAAGAAACGCAATAGCAGAAAAAAACTCACTTGTTCGCTAAATTCGGGTGAAAAA

>ID_273 P_273 Promoter_276 D1_Promoter for RH43845, -250bp and +50bp [2 ]

TAGTATAGGGAAGCTCATTTAATAAAGCTGTAAAATCTAATTGCCAAGCATTAATGTGGAGTAGCAGTGTAATGCAAGGACTTGGCGTTTCTCGCAGTGTAATTTCAATCCGCGCTCTCTCGCGCTCTTGCACTCTCTTCAATTCAATTGACGATAAGCTCGACTTGTTATTGCTGTTGTTGCGGCCGCTACTGCGCTGCAAGTGAAATTTCGCTGGATTTATTTTATTCGCTTTTTCGATTTCAGCTCTTCAGTGTTGTTGTGCTCGGCGAGCGGAGCGGGTGGGCGGCCGGTTGAAAATAGCGAATACACCGTACACACCCCCCTAACACAACAAATATAGCAAATAC

>ID_274 P_274 Promoter_277 D1_Promoter for RH52489, -250bp and +50bp []

AAAACGATTTGGAATTGGAATTGAAATTGCAATGCAACTAAAACAAAAACAATTTAAATATAATGGAACCACAACGACTTGGTCTCCAAGGGGGAGCTGTAAATAATTCAGCAAGAGCCGGAGCAGCGGCGACTGCAAATTAAATCAAATAGTTATATCATGCGAGTAAATCGCTGTGGATATCACACATACGCCCCGTTGACCGCAGACGCGAGTCGCAGCTACTTACACGCCGCGCTCATCGCTCCGGGCATCAGTGCGCGCCCAGTGCTCAGCCGCAGCAGATCGTGAATGTGAACGGATATATATAACATATATCTATACAGTTGAACAGTGTGCATCCCCATGTC

>ID_278 P_278 Promoter_281 D1_Promoter for RH35831, -250bp and +50bp []

ATTCCTAAACAATCGACAACATATGTAACTCATTATTTCTCTTCAGATCCCCGTAAAGATTATCCTTTCAAAATACCATTAGTAATATGAGGTACATAACATCTCCAAGAGTCCACTTCGTTTCGCGCAGTGCACAATGCCCATAAAGTGGACGCAGAGTGCAGTTACTGCTGCTCAGTAAGTGGGAATGGGATGGGGATGTTGGGGCAGCAGTGTGGCGCTGCTGCTCTCCACTGGCAGCTGGGCCTCTCATTCGTGCCAACGCGCTCAACTGCGACGGTCCGCTGAACAAAACGGTGTGCGGAATAAAAACGCGAAAGAATTCGTGTAATAAATGTGAATACCAGTGG

>ID_279 P_279 Promoter_282 D1_Promoter for RH32709, -250bp and +50bp [1 ]

ACCCCGTGCTGCACTGAAAGAAATTTCAAACCCATGCATACAATAATATATGGCATATAAACCAATTGGCAAGATTTCTCAACCGGAAAATATATACAATATATCATATTAACAAATTGAAATTGAAAGATGGATATAACAATTTTAAGTCGGATCATCTACTGCGTTCAGATTTTTCTCAGCGCAGCTTAATCTCAATCTCAGTCGCAGAGAGTGGGTGCGGAAGTGCGTCTTGTCGAGCTGCCCGTTCAGTCGCTCTCGGAGTGTTCGCCATGGAGAACTTTGGTATCGCACATGCCCTGCTCCAGCTACTGCTGCTCCATCTGGTGGGAAGTGGTCGAGGTCACCTG

>ID_280 P_280 Promoter_283 D1_Promoter for RE50527, -250bp and +50bp []

TGAAAGTGAAATTGAACTGGAGAGTGGGGAAGTGCCATTTAAGAGATGATTTCTGGGCCAAGTGCGCGGCGCCTCGTCTTCCCAGGCCGGCCCACCTGCACTTCAGGTCCATCAAAAGTGCCGAACCCGAGGAGCAGTTGGTCGCACACCTGGGCCGCCAGAAGAAGCGAAAGAAACCCAAGCCAAAAACCCGGGCCGGGAACAGTTTTTCAAACGCCTTAAAAGCCCGAGACGGAGCTGCAAACGGTTTAGTTCCTCGCCGCAAACAAAACTAGTCACAACAACGTGGAGCGGAAGCCAGCAGCCCCAAAGTGAACCAGTTAACATACAGAGATACGGTCTGAGGTAAA

>ID_281 P_281 Promoter_284 D1_Promoter for RH62716, -250bp and +50bp []

AGTTCTACCAATAAATTAAACAAAACAAAATGCAACAAAGAAAATAATAAGTGGAAAAACGAAATAGATATAATTTTACAGTTGCGCACATGCACATAAATATTTCAAGCTAAATAATATTTAAAAGTAAGTATTTAAAAACTAATTTTCTGCATTAGAATAAAATGTAAATTCGTAACGACAATTAACCCAGAGTGGCATTACTGCTGTCCAGCATTTTTTTTTTTAGCGGGATGGCAGCCCCGCCGATCAGCTGTGCCCAAGAACGCCAACGCGAATGAATCGTTTTTGCGACAACGAGCAAGACTCGCGAAATAGCCAAGGAGCAACAGACTAACCAGCACAACAGG

>ID_283 P_283 Promoter_286 D1_Promoter for RH21954, -250bp and +50bp []

ATTAAAGTTAAATTTTAATTTTTAAGAGCTTTAAAGCTTTAACTTAAAATGTAAAACAATTGAACTTTTATGCAGCTGTTTCACGCATTCCTTTGTTTCTATCTGAAGCGACTATTCAGCTGCACACCATAGTCTCGTATAGGTATGAATCTGAACGCACATCACATCAGTTTGCAATCGCATCTGGGTAAAGCCCCAAAAGCGATTTCAGCCACAAGATTTAGTATAAAAACGACAGCGGAAACTTTTACAAATCAGTTTAAGGTAGACCGGCAAGATGCTGATGTTATTCGTAGTGGAACTGCTGGTTCTACTGGCCAGTTCATCGGTCCTCTCCATCGAGGTGGACA

>ID_286 P_286 Promoter_289 D1_Promoter for RH18728, -250bp and +50bp [4 ]

AGATGTTCTGAGCAGCTCATCTCGTTGTTTTTGGATTTTGTTTTGCTGCTATATGGCTATATGGCTATATGGCTATATGGGCTGCCTTTTGCCTTTGCTGCTGCCAAAGCTTCGCTGCAGCAGCAGCTGCGGTCAGCGTCGCCGTCGCCGCTGGCGTCGCAGTCGCAGCTTTTTCAAAGCTGCACGAAATGTTGTTGGTGGGCAGCCAGCGCGGCTTCGACTATAAAACAGTTTGCTGCTGCCAATTGACTTTTGTTTTCGTTTGCGTCTGGTCTCGTCTGGTTGTCTGCTTGGACAATTCGGCGACTACAGTATAGAAACCGTGCTGTGCAGTGTTATTCCAAATAGCA

>ID_287 P_287 Promoter_290 D1_Promoter for RH19262, -250bp and +50bp [2 ]

CCTCCCTGGAGGATAATACGCCCCAAACCAGCGTGAAAAACCCATCCCCCAGTTCCGTAATCTACCCATCTCTTTTCAGCCCAGCGATGGGAGAGCGTGTTGAAAAGCGACAGAGAAAAATGGGGGGATGTGGATTACAGCTCGTCATACCATTGGTCACAATCCTCATCCTGGCTGCGCAGGCGCAGGCTGTGACGTCATCGCCCGTCCTGAAGGACGACACTTTGGGGGATGCTGCTCCGACGGCAGCGCATTCAGCAGCCAAAATCCTTGAAGACGTCCAAACCATCAGCGCAGTTCAGGATTGGAGTTTGCTGTGTAAAGAGCTCTGCGGGTAAGTTTCGGATAAA

>ID_289 P_289 Promoter_292 D1_Promoter for RH50904, -250bp and +50bp []

ATGACAGAAAATGAATTTCGGTTCAAATACAAATATTACAATAGAAAGTATGATATTTAGCTCCGATAAACATTTAACAATTTTAGTATTTTAATGGTGAAATGGTAAATTCCGCGCATGAAAAGAGATAGAAGAGATGACATATATGGAAACCTTAAAAACGTAACGGTTGCTTGGGTTTTATAACAATCAGTCAGTGACAGGCATTTCCAGAGTTGCCCTGTTCAACAATCGATAGCTGCCTTCGGTCGCTCGTCACTAACTTTCCTTTAGGCAACGGCCACACTGTCTGGCCACCAAAATCCCAAACTTAATTAAAGAATTAAATAATTCGAATAATAATTAAGCCC

>ID_290 P_290 Promoter_293 D1_Promoter for RH56102, -250bp and +50bp [2 ]

AAAATTATTATAGCTATAATATTGATGTTTCTTACGTATATTTATGGCCATTCTAACTTTTAATAATTTGAGACTGTTTCAGTAAAAAAGATAAATATGTGTAATATTCTCAATTGGAATAATATAAAAGTGGGAACAGAATTAATCATATAAAGTAATAACAATGTTTTACAGTTGATATAAAATCAATACTTTCGAGACTTATTTGTTTTTTACCAACGTAGTGGCATCCCTGTTTGCGGTAATATCGCGCAGTCAGTGTACGGTCCCATCAATACAAGTTCGAGTGAAAAACAGGGAAATGTACGTAATTTCTGTGATGGCACTGCAAAAACCCTGATTTGTGACTG

>ID_291 P_291 Promoter_294 D1_Promoter for RE62581, -250bp and +50bp [6 ]

CTCTCAGCGATTATTTGTATCTAGACATTTGTATGTAGTGCAATTTTATATATAATGTTTTTATATAATGTAAATATATGTATTACATAAATATTGCATTAAATCCTTATTAATACCAAATATTCAGACTTGGGACTATTTTGCTTCTCTTAAAAGATTAAGCTTATTGTGTAACTTAGTGTGGACGGTACATAATATTTAGTTTCTAAACTCTTAATTGCTATCAGTGGCAACGCTATTCAAGTAGCCATCTTTCTTTCTTTTCTCAACTATACGACCGCGTGGACGTAAGTTCAACGAGTTTTGTTGAATATTTCTTCATTATGACTTGAAAAAGATCTAATAAGCCT

>ID_292 P_292 Promoter_295 D1_Promoter for RH43962, -250bp and +50bp [5 ]

GAAAATCGGAGATGACGAAAAACAGCAACAGCAACGGCAATGGCAACAGCGACTAAGCAAAACATGGGCCAAACCAAAACAAAAACACACGATTTGCCGGCAAAATGCACGTGACACATCCTTTGGCGAGCTTCGTCGAGTGCGATCCTTCGTCGAGTGCCGAATGCAGCCGATTGCAGCCGAACGAAGCAGAGCCGAAGCTGCGCAGAATCCAACGAGAGGTATAAAAGCGAGACGCTCGCCGCACTCGTGGCATTCGATTGTCGTCGCTCGAGAGGATAGGTGCCCAGCTTCGAAAAGGATAAAACCCCGCAAAGGACTCTAAGTAAAAGTGAAACTTTAAAGCCAAA

>ID_294 P_294 Promoter_297 D1_Promoter for RH60072, -250bp and +50bp [4 ]

CTTATTTTTTATAGTTTAAATATATTTATATTTCATTATCGTTTGCTCGTATGTCTCCAGAGATTTCAATATCAGAACAAATTGGGTTTAGGGAAATAGTGAACAAAATAAAGCAAACAAGTGTGTTTCCAGATCAGCCATGAACATATTGCATGCATATTCGATTTTTTCTCAGTGCATTATTGCCGGCCGATTCTCGGGCAGTTCCTCTGCTCTTGGCTATAAAACTCGCACAATCTTACTCGAGCGTTCTCAGTTTTAGTTCAGACCGCTGGCGAACCGGTCACGTTCGATTGGTTGGGAAATATCGCCGGACAGAACGTAAGTTTCTCTTTCGAATTTTGAGAACT

>ID_296 P_296 Promoter_299 D1_Promoter for RE72489, -250bp and +50bp [5 ]

CCTTTAAGGGGTGTATTTTTACGATAATAAAAGTTTGTTAGATCGCATATTGTTTTAAATATTTCATATATGTTATTTTATTTTAACGAATTTATACAATTGACTTCAAAAATATCTGTGCACCATTCCATAAAATGTAAACCGAATTAATATGTTGAAAATTACTCTCGTATTAACTCTTTTTTGGGAATTGATAATTCATTTGCGATACGATATGAGTTCTATATAAGGCTTAAGCTTTGGGGCCAATACTTCAGTCGTGCGGCAAACGCAAAGCTGAAAAGTATTCTAGTTCTATAGATCTCACGGTAAGAGAAAGTATAAAGTCTAAATAAGAGAACATAAAAATG

>ID_297 P_297 Promoter_300 D1_Promoter for RE09539, -250bp and +50bp [6 ]

TCAAGAGGCAAAGCATATTTAATTATATAACAAACTCCACCAAGCTGAAAATAGTTCCATATTTTGTTGCACATAGTTGCAATCGTCGCAGTTGCTCGGATAGTGATTAAACCTGCAACACCTTATTTGACGCGCCCAAGTTTAAATTCATTACACGATGATCCGCAATATATCGCTTGGCATCGCCATGAACGAAACTATCGGCGAATCATCGACGCACGACGTATCGATGTTTTTTCACACCACCAGCTTTTTCTTTTCGCTTCTGGTTTCCGGCAAGGTATGTGCGTGATTTTGGGCCCACGTGTATTTCCATTAATTTTAAGCCGTAATTGTCGTTTTTGGCGGTT

>ID_299 P_299 Promoter_302 D1_Promoter for RH39661, -250bp and +50bp []

TCAATGTATTGTTAAAATGTTACACTTCCGAATAGCCTTAGAACCTTGACATACATTTTTAATGTAATATCAGATACTTATTTATTTACAAGAAAATATTTAATAAATACAATTGACTAGTGTTTCATAAGGCATTTCTATTAGGAAGTTGATTTTAACAGCTAAGAGACAATCGCTGTTAGATTTGAATACACTTGTGATCATATGGAAATAGATGGGATGGATTGAATCCACTCTGGGCGCTTTGGAGCAGTGGATGAGAGCAGCTCGTTGCGACTGGTTGTGCGACAGGTGTCGGAATAAATAAAAGCAAAAACATTCGACGGCTTTTGTTTGCGCTGATAAACAAC

>ID_302 P_302 Promoter_305 D1_Promoter for RE33992, -250bp and +50bp []

CTACTGACGTCACAAAGTGTTGAGTCACCATTAAAAGCATTAATTGTGCACAGTCTTAATTCCTGCCGATCCAAAACTTATTTTCAAAGTTCGAGCTATCCGGTTTGTTAAACAAAAGTACTTAATCTACTGCTCAGTTTGATTGCATACCACTGTGCGAAGGCTGTTCGCTCACGAACTATCGAACCGGTTCGCCATGCGTTCCCATCTTTAGACGGGTATTTAAGCGCGATTCGCTGCAAAAGTGGCAGTTTCGTTCCAAATTCCGAAGCGAGAGTTTGTCGTGGGAAGTTGTTGCGCGCGCCCTGTTCGCATTTATGTGTTTTTATCGTGATTCCGTCAACCGAAAT

>ID_303 P_303 Promoter_306 D1_Promoter for LD03373, -250bp and +50bp []

TCGAGCGGCACATCATTGTTATCCTCGGCTTCTTCCGAAATTTTTCTCAATTTCGAGAGCAAAGTTTGAAGTCTTCCTTCTTTGACGTCCATCTTGCTTTGCATCCTGATTTTAACTGAATTTCAATTGTGGAACTTTTCCCATGTGGCATCACCGCCGTAAGCTAGACGCTAGAGATGGCAAATGCGCCATCCGCGAAATCCACCACACCAATCGGTCATTGGCGGCTATCGAACGTGTTATCGAGCCGTTTGTTTACGCACGTGTTGTGTTTGATTTAATTTAGTTTTAATTTGTATTTAAACGCACAGAGAGCAGCTAGCAAATGGACAGCGACGGTGACGACTACG

>ID_304 P_304 Promoter_307 D1_Promoter for RH49748, -250bp and +50bp [2 ]

TCTATGATTTCCATATGTTGCAGCAGGGTTCAAAGTCCACCTATGTGCACCTCCTTTGAATTGAATTTTCACTAGCCCTACAGCCCTAATTAATTAAAGCGATCTATGGCACTAAATACTTTTGCATTTCCCTGTAATAATTTGTTGATGTTTTTTTTAAATAATAAATAACATTATAGCGACGCAGGTGTGAGAGCATTCGAATACCCTAAATTTGCCGCTCTGTGTGAATTGTCTGAAAACCAAGTTGGCAGCGCTATTTCGATGTTCTTGTCAACCGGCCGGAATTTCTTTCGTGTTTTCAGGTGAGTTCGTGCGAAATTGGCTTAATTATCTAAAGTGCCGCGATT

>ID_305 P_305 Promoter_308 D1_Promoter for RE42975, -250bp and +50bp []

ATCGTCATTCTCTTCCTCCGATGCCATTGTAGCACTCGAATGCAACTTTTACACTGTTTGAGCACACGAAATTGTCTTAATTTCCACAACAAATAATTAAGCACACACAGACACGACGAGAAAAAAATAGTCAATTGTAAGCGGCTTCAACAAATATGGCGGAGCGGAGAGCGACAAAAAGTGAGAGAACTAGGGTTGCACCCCGATAAAAGAAAGCGAAACGGTCACACTGGCGACATGACAACCCTGACTTTTATGTTTTTTTAACGACCCTGTCCTAACGTGTTTCGGAGAATTTTTCACCAAAGACCAAAAACCTTGTGTATTGTTTTTGAATTGCAAATCGCTGA

>ID_307 P_307 Promoter_310 D1_Promoter for RH48856, -250bp and +50bp []

ATAAATCTCCAATATCAACCAATATCTGAGGAATGCATTGTCTTGATGTTTACTTATGTCGAACTAATTATAATTAGCCACCAGTCAATAGGTCTGCGCCGAGCTAAGATAACACGCCTTAATCGCGATTTAATCAACTCAATACATACAAGAGAACGTAGCGACTTAGGGGCGATAAGATCAATCCGCAAATCACTCCCGGCAAAGCCGAATAGGTTTGGTCATCCTATATAAGCAGCTGCATCGACACACTTTTAATCAGAGTGAAATTTAGCTTCCACCTAAAATCATCATGTTTCTGGCCAAGAGCATAGTGTGCCTCTCCCTCCTGGCGGTGGCCAATGCCCAAT

>ID_308 P_308 Promoter_311 D1_Promoter for RE54076, -250bp and +50bp []

GGATTTGTGCAGGTCAATGATGATTTCTTTCACGACTACAGTTATCCAAAAATATGGGAAGCCTTTACTTGGCTTTATTGTAACCTTTGTTCTGCATCATATCTAGTATATGGGTGTTATGGGTACGCCTAAACAAAAACCCGTTTACGCTATCAGTTCGGCCCAAACACAGATACATATGCACACACACTAGTGCACTTGCCATGGGCGACTATTAGTATAAAAGTATGCGAACCGGACGGAGAGTTCAGTATTTGCTTGTCGTTAGAAATCGTCGGACACAAAAGCAGCGTTTTCTCCGAACGGGAATTTAAATTGAAAAGTCGGTCAGTAATAAATAACAATTGTAT

>ID_309 P_309 Promoter_312 D1_Promoter for RH70348, -250bp and +50bp [2 ]

GTAAGCACGAATGCTGGCTTATAGCACCTAAGCAGTGTGCTTGCTGTCTTTACCTTACGATTCTATATTATTATTCGGGAAAAGTAACTCGAAAGTTTTGCTCACGATGGCAAAAAAACGTCCACCAGTTGATAGCGATGTAAGTGAATCGATAACCGTCTTGATTCGCCGCGGTAAACACATGGCCGGCCTTGCCAGACTCGCTAGCAGCCAGTGTCATTTTTGGTGCTTTCCTCGCCGCACGAGTTGGCATTCCCATAGCTGTCTTTTATTTGTGAGGTTCGTATGTTCGTCTGCCGCCTCCGAAAATTACATAATTTTTGCGTAAATTGATTTCGTTTAATTCGACC

>ID_310 P_310 Promoter_313 D1_Promoter for RH07810, -250bp and +50bp []

ATGCGCTTGCTTTGTTGCGCCCTAACGAGCCGAGATTGAAGTAAAAGTACTGTATTGAGCACGATAAATGGCAGATGACTTGGTTCTATTGGCCTAAAGCCGCAATAAACATTTTCAATTTCCCACCTTTTGGCGCTGTTCGAAACCCCAACGCACTGCACTGGCGACTGTCTGGCAACGCCATGTCACACTAGCCGCACCGCTCACACCATGTTGCTCTTGCCATGCCATGCTGTCTCGCTCACGCCGAGAACATCATTATAGAGTCGAATTGCGAGAGCTGCGCAGCGTAGGAGCTACAAACTACGTTTTTCGTGTGTTTCCCTCTTGAAGATAATTAAAATTTAATT

>ID_312 P_312 Promoter_315 D1_Promoter for RH48101, -250bp and +50bp []

AACAACAAAAAAAGGAAACAACCACAGAAAGCCGGGCTAAGACGTCAGGTGAAACGCAGTAGCTTCACTCGCGACTCGGCGCTTCCACTCAAAGGTGCTACCGCTGCCCACTCAAATCTGCAGCTCGTAGATACGAAAACCAGATAGCGTCGAGCGGCTGGCGATCTTCACTCAATGGGGGGAAATACTGCTATAGAGTCGAAAGCTTGTACACGTAGTTTGGCATTCGCAGTCGCTTGTTGGCGTTTTTAGTCTGCTGCCTGATCTTCGACGCGCTGCAGCTGTTTTGGAGTCGCCGCGAGTGCCATATTTGCTTTGACCGCGAAAATTTCTGGGCTAAAAACAGAGAT

>ID_314 P_314 Promoter_317 D1_Promoter for RE21743, -250bp and +50bp []

AATATTATGCTAACTGTACCACCAGAATTGAACCAATTTCATATCAAAAGAAAAGTGTGTGTGCTGTTGTTTTAGACTGCGCCCTTTTTTCGAGAACTTGTCGAGCCAGCGCTGATCGATATCTGCCCTAACTCACACTTCCCACAATAGCTAATTTTTGTTATAGCTAAAAGAGGCTAAACATGCAACACTGATATGACACTTCGCTCTCTCCTGTTTTCACAATGAGTATGAGGAGAACGAAAAGAACAGTTGATTTCTTGTAACCGAATGGAACACTTGTGACTTTTAAGTGCGCATCGAATAGACTGGATGTTGAATGAAAAGAAAGACGTGTAAATAACGGAGTA

>ID_315 P_315 Promoter_318 D1_Promoter for RE62753, -250bp and +50bp []

CTAATGGGTCAGTGTGGCCAGATGGGGTTGCTGCGCAATTTCAGAGCGTTGCCATTAGACAAGAAACACCAGGGCTGTCGTTACATATGTAAACAAACAATAATTTAAAACTGCATTTAAAAATAAAACAAAAAATTGAATAAATCTTTAATAAATATTAAGCAAAAGGTCATTACAAGTTTATTTCACACAAAGTACGAAATCGATTAATTCCATAATCTAGTTCAACGAACCAGTTCTGCTCATCATCAATCAATTCACGTGCAGCCCTCGTCCAGCCGGTTAATTTTTTTGCACAAGCGCTGGACAGAAAAGTGTAATTAAGCGGCTTAAAACGGGTTTACATTTAT

>ID_316 P_316 Promoter_319 D1_Promoter for RH09887, -250bp and +50bp [4 ]

AAGTTAATCGGCAAACCGCTTTTGCATAGCAAATAGAGCTGAGCAAATGCTCACATCGAATCCAAGTCTGGGTCTCATCCCAGACTTATCAGCTGGCGGGGATGCAATCAAACAGTGTGGCTAGGAACTGGACTGGAGCGATGATAAGCAACTGAACTCTTGACATACAGAGCGATAGAAATATATAGACATACATATATAGACATCCAGCGATCTGAAGCTATAAAGTCAGGCACTTGGCCGTCGGCGCCTCAGTTCAGAACCTCAACTTCGCCGCGATCGTGCAACTATAACTAACTATATCTCAATATGCTCAAGTATCTGATCGTAGCTCTGGCCCTCTGTGCTGT

>ID_317 P_317 Promoter_320 D1_Promoter for RE65817, -250bp and +50bp []

TCGGATAGGCAGCTACCTTGAGGGTCAAGTGCGAATACAGCCCACGGAACGCGATTATCAGATGCGAGAGTGGGAGAATGGGAGGAAAATGAGCACTCAAATTAGAATTTAGGAATTTTAGGTCAGTCGCGTAATTGAGAGGCTGAGCAAATTCACTGCAACGGTTAATGGAACTAATTAACCAATCAAGCGGCGGTGGTGACGATAAGCCTCGGTTCAAGTCAAAAATCGACGCATCTCTCGATTCCCGCTCAGTTTCGTTTCGGCAATCATTCGGATCACACGGAAAACGTAGAAGCCTCATTTGCCGTACATTATGGCCTCTCAACCGCTTGGACTCCTGCTGGGAG

>ID_318 P_318 Promoter_321 D1_Promoter for RH07729, -250bp and +50bp []

TTTTACACGACTAAAAATGAATGCTTACAATTTAAAAAAAAAAATCAAAATATTATTTTTCAAAGAACTAGGAATTAAATAATGTATTAATGGGCTTTTATTCTCCTTATTCCGCTTACTGTTAAAATAAAAGTTCATTATATTTATAATTGAATAATAATTGAACAAAGTTAATATTTAATTCGAGTGCAGCTTTAATCGAGAAAAACTTGAATCGGGACAAGCTTTTGAGATCAATATTTTCAATCTGATGCAACAGTCCCACCGCAGAGCTTCTCAGATTAATACGCTAATCGGATTCTAGTTAGTACACATATTAAGAGTAATTAAAACTGCGTCATGAGGTGGGA

>ID_319 P_319 Promoter_322 D1_Promoter for RE10256, -250bp and +50bp []

CGCGTCGACCCCTTTAACCTCTTAACCCCCGGCTGTTTGCTTGTTTTATTTCGTTTATAGCGCGTTACATCTTTGTCTTAATTTTTAGTGATAAGCCAGCAGAACCTCCCGCTTCCCTCGAAGGCCGTCACCACATCCACCCGTCCACCTACAGTCGCAACATGTTCGATGAGATTCGTCATGCCAGCCAACACCAACATGTTCGATGCGACCGAGCTGCTTGGCTTTTCGGACATGGATTTTGGCTTCAGTTTGTCGTCGTCGCTCATACGCAACGTTGGCCGGGCGGAAAACAAGAGTTTCATTGAATTTCATTGAGTTTGTTGCTACTTTTGCCGCGAGTGCTGCGG

>ID_320 P_320 Promoter_323 D1_Promoter for RE21494, -250bp and +50bp [2 ]

GCGCCATTTGATTTTATGCTTATCGCCAAACATTACCCATTTAGCACAAAAATAATGTGAGAGCATACATATGTACTATATATTTTAAATTGAAAATTCCAATTTAGAATCTAATTTAGCTTTATGCTCATGAAACGCGTATTTAATACCTCCGCTACTCTCTGTACAAAGAGAGAGTGGAGAGAAAGCGGGAGAGTGAGCGCTTAGGTAAACAGCTGAGCGTTCTTAGAAATTGGACTTCGTTTGTTTAGTTTTCCAGCGGCAGCTGATGAGTGGGGTGGAAAATTGTGTGAGTGCGCTGCGTGCAGTGAATAAATGGTTCGGAAAAATGCCAGCAAATTTCAAAGTGC

>ID_321 P_321 Promoter_324 D1_Promoter for RH09431, -250bp and +50bp [4 ]

TGTATTAATCACGCCGCTAACGTCACATTTTTGCCCGCCATTAGTGGGTATGTGTGGGCAAGTCAGAATATAATAGAAGACAAAAAAAAAACCCAGGCACGACAACAACTAGCAGCCCCCTCTCTCTCTCTCTGTACCTCTCTCCCCACCTTGCCACGCCCCGCGGGGCGCTTCAAGCTACAACTCTTGTTTGCCGGCATTGTGGCGACCACAGCCGGCCCCTATAAAAGTGGGCTCGCTCTTCGAATTCCGTTCAGTGCTACTTTTCATTTGATACAGTGTACATCGCGAGTTTCACAACAGAAGAAAAAAATGTAAGTTCCCCACAGCAATTTCTATATATATTGTGT

>ID_323 P_323 Promoter_326 D1_Promoter for RH03206, -250bp and +50bp [6 ]

AGCATGAATCGTTTCTTGTTCACTTTACTTTCGTGGATTGATAAATGGAACTGCTTGATCATCTTCCTAAACTAAATGTAAATTTTAAGTACAAAAATTGCTCTCACTCAATTTGTTGCCAAAAGCTCGCCGAAATTCTCAAATTATTTGTCCAATCATGCTCGCATTGCATTGCCGTGTGGAATACGATCCACTTGAAATCCACAAGCCAACAAAAAGCTTTTGTTTGCCGGTAGCTTGCGCTTTACAAATCATTTGTGCTCGACGATCGTAAGCGAGGAGTTGCGCAGTTCGCCGATCTGTGTATATAGAATATATAAAGAATATAGCATAAACACACAATGGCGAAC

>ID_324 P_324 Promoter_327 D1_Promoter for RH23176, -250bp and +50bp []

GGCGAGAGCTTGGGGCATGGACATGGACATCGGCCTGGGCAAAACGCAAAATTTGCATTGCAATTTTATTCTTGATTTAATCAAGTGCCGTGGCACAAACGCCAATTTAACTACCCACACACTACCCTCACACATACACAAACGCTCACACACACCCAAACATCCGCAGGGCAATGAGAATGCCGTCAGACGTTGCGTATACGTAATGCTTTTGGTTTGTGTAGCATTATGGGATGGTGTGAGCTTAGCGCGTAGTTTGCACCCGCCAGCCGAGTCACCGAGATGTACCAGCTGGAGAAGATCTGGGTCCTGCTGTGCCTGGCCCTCGTCGGTGTCCTTGGCTTCGGCCA

>ID_326 P_326 Promoter_329 D1_Promoter for RE43310, -250bp and +50bp []

AGTCGATGCAAAAGCGTTCTCTTTTTTTACAGTGTTACCAGAGCCATCGGTTCCTGAAATATATCCTAAATGGATTCCGTTGTTTTTGGTATCCCAAAATGGATTCAATTTAAACGCAATACACCTGTGCATTGTTTCAAACGAAAAGAATCGTGTGCGAATGCCTCGCCGCAGAGTATCCAAGTTTAAGCCAACCGGCGGAGAGCTTATCTCTTTTGTTTTTATAAGGCATTGCGATGGAGCACCGGAGCTCAGTCTGTTTTCGTTCGTTGCCGTGTTAAGTTCACGCGATTCCACCTCTGATCCGCGGAGTTTGATTCAAATTCCGGGCTGCTGGTGGCTGGAGCGCT

>ID_327 P_327 Promoter_330 D1_Promoter for RE70558, -250bp and +50bp []

AGTCAGTGAATGTAAGTCTCGCTTCACTTGATTCCATTATTTTCCTATAACGTTTTTGGAAAGTGACGCACAAAGTGTCGGTGCATCCCACTGTGCAGTTCACCCCCTTGGTGTTCGCTCACTCACACACTCGCACGACCAGTCGGCCATCTCGCTCTGGCGCACGCACACCGCCAGCGGCACCAACCATTTTCTGCCTTTTGTCTCGGCCGTTTTCGGTTCGATTAGGTTTGAGAACCGGAGAGTTCTCAGTTGTTTTCGAGATTCCGTCGCGTGCGGTTCGCTGCCCGTTCTACCCACGTCGCTCGCTTAGTAATCCAGGCCGGATCCGGCGGTCTTTTGACGTCCCG

>ID_330 P_330 Promoter_333 D1_Promoter for RH35136, -250bp and +50bp [2 ]

TGTGAGATCAACAATTAAATAACAGTTTGTAAACATTCGGTTTATTATAATTAATAATGATCTTTAAGTCTTTCGCATTTGGCAATAAGACAGTAGAGGAGAGTAAGGTATAAAGATAGTAAATATAAATAAGTAAATAGCATAAGCAAAAGCTCGTTTTAGCTTTTAAACGGAGTCGGTAAAATCTTCCTAAAAGTAACTATCGAGGGTCGGCTGGGTCAGGCAACGCCGCCGCACGCCTGCAGCCCTGGTAACTCTATTTTCTATTGAGCACCGACAACGTTGCGTGTATAAGACAGTTTACATAAATTATTATTTACAATTGCACAGAGCGTTGATGTTGTGCGTTC

>ID_331 P_331 Promoter_334 D1_Promoter for RE27784, -250bp and +50bp []

TAAGAATCCGCTGTGGCCGAAATCTCATTCCTCAAGATTTATAGTACCAGATTGCTGTTCTGCCTGTGGCATTGAACTTAAAAATATGACGACCGGCACAGGGAAAGATAATTAAAAAGCAAAACTATGTAATCTTTTCCTGACATTCCCCATTGCTTTCCACACCTGATGGCTGGTGGTGTGCGTGTACGAGGGCCAGAATCGATGCTGGTGGGCAGTTAAATTGGCAACAGAGGGAAGCCGTAGCTTTAGTCTGCACTGAACAGCAGCCAAGTTCAGTTGGAATCGATTTGGAATAACCTATATTTTGGTACGGCGGTTGTGCGGGCTTATCAGGAAAATTATCTATA

>ID_333 P_333 Promoter_336 D1_Promoter for RE04871, -250bp and +50bp []

TTCGCCTTATGTTTTCCAAAGAGCCCCCTATGCTGAAGGATCTATTTTTAGGCGGATCGGCCGGTGGGCAGCATTATCGCATCCATGAGCATATTTCCTCGCTGTGTGTATTCGTTTGAGTATTTCTTTTGCCATTGGTATGGGGGCGTGGCGGGGGCGGTGGCGGCGGGGGGCATGGCGACGGCGCTTCTTGGACGAGATGGCATTTCGGCGGCGGTATAAAATCCGAGTACCAACGCGGCGATACTTTAGTCAGTCAGTTGTGTTCCAAGCCGTACACACCGTAACTCTTGATTTAATTCACCCGTGGTGCCGTAAAAAAAACAAAACTAAAACAAAATGTGTGACGA

>ID_336 P_336 Promoter_339 D1_Promoter for RH57736, -250bp and +50bp []

AGCAGTGATTTCCAAAGCATCTCAATTCTTCGAAATAGAAATAGTATTTCAATCCGATTTCAAAAAAAAGAAAAAAACCCTGCCTTACATTTTCGAAATGCGCTGTACCCGCCACAGATGAACCATATTTATTTTCGCAAAATCAGAACACCATGAATAGAATGGGATGGCAATTATATAGAACTGATGATCGTTATTTGAGCATTATAGAGGAGCTTATCTGGCCAGGCGTTTGGCGGTCAACGTTTGGAATCAGTTTCAGGTCGAACGTGAATCGTGGAGCACGCCAAATGTACGTCCGGAATTGTCTGCCGTTCCTCTGCAGCTATTTGCTGATCTCATTGGCGGCC

>ID_337 P_337 Promoter_340 D1_Promoter for RH05869, -250bp and +50bp [5 ]

AGTCGAGTGTGAGAAGTGCGAATTTGAGCGACGCCGATAATGAACTTCAAATTGCCGCCACTGGCTTTGCGAATCGAGATTCTAAAGCTGAGTAAGAAACCGGACAGGTGAAGCTCAGACTGCTGCTGGTCAGTGAAACAGTCGGCCAGCCAGCACATCTTCATTCAGATTTATGCAAAACGCAAAACAAAAAACAACAATACCAAGTAGATCTAAAAAGTTAGTATATAAGCAGTGCCTTTTGGTTACTCAGTTATTAGTAGCCCGCCAACTGGAGAATAGCGAATACACGTTGTGATACCTGGATAAACCACACATATAACTCATATTTCAAATCGAATCCACTATGA

>ID_338 P_338 Promoter_341 D1_Promoter for RE59367, -250bp and +50bp []

TTTAGATTTTTAATTATATAGCTATTTTTTAAGGTTTTTAATAGACTTCGACCTTATTACCTATAACATGAGGATTGTCTTAATGTTCCCTTATGATTTAAGATTAAATTGAATTAAAATATATTCTTTATGTGAAACGATTGCTTCCATTCAATTGAGCATCCCTTGTTCATACTTCGTATGCGTTTTAAAAAATACTGTTAGAGTGGTGGTATTTTTTCAACTCAGTGAACGCAGCCCCACTGAAAACGTTTAATTTGTTGTTCCTTGAACTTATACGGGTTTAGTTCCTAAATGTTACTAGTTAGTAGTTCCTAAAAAATCGATGCAATGCACCTAGACGAAAGTCA

>ID_340 P_340 Promoter_343 D1_Promoter for RE06314, -250bp and +50bp []

ATAATTGTCTACAAGGGGATCTGGTACCAACGCAGTGGGAATTGTGCCAGCGGCCGCCAAGTGGAGCGATGATCAATGGAACGTTCCCGGAACTGGAGACGTAGGTGTAGTAATTCTCCCGCCTGCCGGCCTAACTAAAGATAGTCGCATCCCCCTGGCCGCCGCAAGAACTAAATTAGCCCACAATTGATAATTGAGCGGAATTGAATTGAATCACAGCCGCCTAAGCAGAGGCACCCAAATCTGGGCTCAGTGAGCCACCGACCGACCAGAGAGTCGGACACATCCCACAACCAGCGGCCGCTACATACCACTAACGTAGCTAGTGATCATCATGTCGAATATCGAGG

>ID_342 P_342 Promoter_345 D1_Promoter for RE68882, -250bp and +50bp []

ACCCTCGTTTTCTTGTTTTCAGGCCGGTCTCTTGGTTTTAAAAAGAGAACCGGAGCGAGAACTTTATCTTTATATTGTGTGATATTGCTGCCTTCTCATTAATTCTAACCCACTGATAAAACATGAAGAAAGAAAACCAAGGGCTGATTGTGCAGCGAGCGATTGATTGAAGTCCGCCGTCGACGTCGGCGCTCCGACGAGGGATCATAGAGGGAGGTTCTCCGGTTCGAGTTCTGGATCGTTGGAATCAGTCGTTGGGAGGCACTTTTGGCGTGAAGATCGCGGCGAGGAAGTGCTCGGAATCTGAAATTTTAAGTCATATTGTGTGGTCTACGCTTGTGGTAAAGTTT

>ID_347 P_347 Promoter_350 D1_Promoter for RE03342, -250bp and +50bp []

CGACACAATTTTACTTCAAAATTTAATAGAGCATTTAAGTTTCAAACAAAACTCGAGATTAAATTGTTTGCATACATGGCGTGCATGTGCTAATTTTTAAGCCGCCATAATATGACTTAATATTTGTTCAGCAAGCGTTGACACACGTCTTGGGAATAATTCTCATTGGGAAATATTCTCTTATTTTTGGGCTGTGGCTCAATAGCTCGTTTGGCGGGCTTTGGATTCGGTTTTGGGTTCAGGTTACAACAGTTGTCGTTGGCGTGTCGTCGGTTTCAGAGGTTTTTTGAAAGACGGTTTTTAAAAAACCAATTTCCGCTCTGAGTCGCAGTTTAAACTGAACTACTGAA

>ID_349 P_349 Promoter_352 D1_Promoter for RH48279, -250bp and +50bp [6 ]

ATAACGATGGAGAATGTTCTTGATGGGTATAAATATGAAGTCATCCATAGATAGCGATCATAATTGGTAATGGTGCCGCTCTCTTGAAAATAAAATGAATTCCACTTAAGAATTTGTCTGGTCACTCGGCTCTCGAGAACCCCATTCAACTGGATTTATTGAAGCTATCTGTGTGTAAACTTTTCACCAAAAAGCTCCGCCTATTGATAACCGATTAACATCGATGTAGGGTATAAAGTAATAGACATTGAACCTAGTCTCCGTTGGGAAACCACATCAAACAGACTTGCCATGAACCGCTGGTTGAATCGCGTTGCCGTAGTCACTGGTGCCAGCGCCGGAATCGGAGC

>ID_350 P_350 Promoter_353 D1_Promoter for RH36709, -250bp and +50bp []

TAACAGTTATTAATTCTACATACTTGCTCTATCCTTTTAAATTTGATTAAAAACTAACGAACTCTTTGCTCAATTATTAATTGAATAAATGTTTTTTTTTTATAAACTACCAAATAAGCTCTCTGGAACCTGTTTATATGCCCGTTGTCTGTGGGGTCGCTATCTCTCTGTTTGTGTGCTCTTATCAACCAATTAAGCGGCAAAGCTCGCGCGGCCTTAGGATTTAAAGCCAGTAGAACGAACTCTCGTTTCACAGTTTTGTTTGGGCGGTGAGCCGGAAAAGTTCTAATCATCGCAAAAATGCTAAAAGTTCGCAGTGCTCTATCATTAATTCAGTCGCAAAAAGCGAC

>ID_351 P_351 Promoter_354 D1_Promoter for RE02758, -250bp and +50bp []

TTCAGTATATTTCACTGGTTCCTCTGCTGTATGCGTTGGTCTTACAAATGAAAAGAACAATATTTTGTATTGTGTTGCAATAGCCTTGATGTTTTATCACAGAAGCACATTAGCATAATCACTGATTTATCGCGATGCCGAGATGAATCATGCAGCTAACGCGACTATCGCTGGAGCCCTCGTTTGGTAATTTGAACGGGAAACTAGATCAAATGAGCGAAAAATCTGTGGCGAACCACTGGAGTGAATTATTTTTAGTGAGCTGAACGTGGATTGTGACAATGAAAAATTGAGGAATAGTGTTTACATTCATTTTATAATTTATTTTACCAGTTATCTTTTTTATCGCG

>ID_352 P_352 Promoter_355 D1_Promoter for RH09027, -250bp and +50bp [2 ]

TTCGTTTAAAAATTCGCTTCGAATTGCGCTTCGTGTAGCGATTGTCTATATCATCGAATATGTATATGTCAATTGCTGTACATAAGAGAAGCCGAGAGAGCAATGCTAAACAGATTTTAAAAATTAGGTATATAGGTGGCTGCCGTGGTAGCACATAGTTTTGTTCTGATAAGCTCTTATTTCTTGCCGACTCATCTTCTCAAATGCTGTCTACAAAAATTTATAAATATTATTCGTCTGTAAAAATCATCCGCAGTTGAACGCAGGTTGAGCAGGAAGCTAGTCGAGACTATAATCCATATCTTGTCTGATCCTTTGTTCAAAACCACAGTGAGTATAAAATAAAGGAT

>ID_353 P_353 Promoter_356 D1_Promoter for RH70249, -250bp and +50bp [-1 ]

GTAGTTTCCAGAACACAAGAGAAAAATCCATATGTATGTGCTTAACCGGCTTACCCACCTTTCTCACGAAATGAGGGTAGTTTTTGTAGCACAGTGATGTAGACACTCGTGAGAAACCGTAGGAGCACGTATACGATGTATGTATATAGTGTACTCGCTCCAACCCCAATGCCGCAGACCCGAGAGACAGGCAGATTTTTCCCGAACCCAGCGGTTGGGTATAAATAGAGCCGCCTCAGTCCGGTCACGTCACTCTCAAATGAAAAGTGTTCAAGTGCATTCAAAGTGAAGCTGAAAAAATAACCAGTTAAAAATAGTACAAAGAAATTTTCTTTCTTGCAATTTGCAAG

>ID_355 P_355 Promoter_358 D1_Promoter for RE09489, -250bp and +50bp []

GTATGTTAAATATATTTATCTTAATATGAAATTTGGATTTACGTTTGCATTACCTATTGATTAACTAATTTGTGCCATTAGCTTACTCACAAATAATTATTATTTATTTCAGGGAAGAATTAATATTTATATATGTGTTTATTACTATAACCAGTTCGGTTCCACTTCTTTTAAAAATAGTGCTCCAACTGGTTCTTGGGTTTCCAAGTATTTGCAGGCACGGTCACACCGACACAGTTTTGTTTCGATTAGTTTGGTTATCGATACCGCTGTTGGCTGCCTTCGTTGTTTACCTTTTGGTCATTAGCAATTTCGACGCCAACGCGAGAATACACAGATTATGAGTCCTG

>ID_357 P_357 Promoter_360 D1_Promoter for SD04538, -250bp and +50bp [6 ]

TAGTTGTTTTTAAAAGCCAATCTCCAAAAATGCAATGGATACATAATGCTAATTCAGCATATAACATGCTGAATACCTTATACTTGTAATAAAATTAAATTAAATACTTATACAATAATTTTGATTCTATTGCGGTGCCCGCTAGTGTAGAGAGAGATCTCTTTCTCTCCCTCTCTCTCTCTGTCGCACTCTCTCAACAAGTGGCTTAATACAAATATATACAAACAACTCAAAAGGCCAGCGAATGAGCCAAGTTAGTACGATTTCTGGTACCGGACTGACATGTACTAACCGCGCTTAAACAGCGGATGACGCTCGCGTGCGTTCCACAATAACAACAAAAACAGTAT

>ID_358 P_358 Promoter_361 D1_Promoter for RE46380, -250bp and +50bp [3 ]

GTATGCACTTATTTATTCGTCCAATTACAAGTAGATGGTTATTTATATTTAACAAGCTATTCACCTAAGATTAGCTTGAATACCTTATCATCACAGTTAAAGATTAATGTTAGGGAGCTAGTTTAAGTCTGCTTAATTGTCAGGAATCCCCCTGATGGCCCCTTATATCGATTCCTAAACCCAAGTTGGGGCTGTTCAGTATTTTTCCAGAGCCGGGCGGTATAAATTGCCGCTCCAGTGGCGGTCTTATTGCATTGAAGTTGGGCAATGGGAAGTGATCGCACGTTAAAAAAGCAAAAGATGTCGGTATTACAGGAACTCAAGAAAATCACCACAATTGTGGCTGACAC

>ID_360 P_360 Promoter_363 D1_Promoter for RE16541, -250bp and +50bp []

TTGCTAATAAAATTTCGATAAACATTAATATTACATTTGAAATAAATTTGACTATTGAATAAAGCAATCGATATATTAGAGAGGAGACAAATTCGGACGTACAACCATATAGCACAAGGAAACTGGCTATGTTTTTATATATTAAATGTACCTCTACCTATTTTTAAAATATCGACAGCACTTAGATAAAAAATTAAAACAAACAATTACCTTTCGCGATAATCGCTCAACTCCCGATTGTTCCATCCCCAGCCGATAGTTTGACAGCCCTGCCACTGAGAGAGCATAATGACGTGGTGAACTCTGTGTGAAAAAAGAACAATAAATTGCCGACAAATCTCTAGTGTAAT

>ID_361 P_361 Promoter_364 D1_Promoter for RH25164, -250bp and +50bp []

TATATTCTATAGCGCGTGTGCCGCCGATTATCCACGTGTTCTCCAGCTCTATATAGGTACAGATACAGATACAGATACATTTACAGTGTATCCACGGGCTTGGCACTCTCCTTCTCTCTATTTGCCTTCGAATGAATACCCATAACCAGTAGCTTTCGTCATGTGCTTTGCTCTTTCCGAGCGCGTATCTGTATCTCTCGCTCCGTTGAAGTTTTGTAGCTTCTCGCAATCGGGGGTTCCAATAGCGGTTCATTCGTTGCTAAGCTCTCCCCGCGAACGGTCGTGTGTGATCTCCGATCTCCGATCCCCGATTTTCGGTTCACGTTCTATACGGTACTCGATTCTCGCAG

>ID_362 P_362 Promoter_365 D1_Promoter for RE65433, -250bp and +50bp [1 ]

GATTAATCTGCTCATGATTGGGTTTTATTGCCAATATAGGCCAAATCGATTCACACTTCTTGGTTTGCTTGCCCTGCTTATCACTTGGAATATCGGTAGCATCGGCTCTATCAGTAGTGGCAAATAGACGATTATAAATCACCTCGGTTAACTACAGCCGGGTTAATTCACCAAAGATTATCAATGAATTACGATACCCACGCTCCTTTTAAAATCCGTATAAAAGTGCCCGAGTTCTTGGCCAACACTTTAGTTGTGATCAAAATGAAATTCGCCTGCGCTACGGTTCTGCTCTTGGCCACCATTCTTGGGGCCCAGGCTGTGGACTGGAATTCGGTCAAGAACCTGAA

>ID_363 P_363 Promoter_366 D1_Promoter for RE28313, -250bp and +50bp []

AAGCGAATAATGCAAGTTAAATAAATATAATACAAGAAGTGAGCTTACGATTATTTCTTAGCGTATTATAACTTGATCCCACAGTATTTCTGAATAAACTAAAAAAAAATACATTTAAATCTCTTACTTAAAATTAAATAAATATCCCTTAGCAAAATTTAAAAAAGAAAACAGTGTTCCAGTCCGCCGTTTCAAATTAGTGCGAAACAAGTGAAAAACATGAAACAACAGTAGTTGTAGGCGATATCGATTTTTGTTTTATGTAAATCGATGGCACATGCGGAATGTAAAATAAGACCAAGTTAATACAGAGGATTTCCGTAGTAATATGATTGAAATGAACAAAACTG

>ID_365 P_365 Promoter_368 D1_Promoter for GM19208, -250bp and +50bp [5 ]

GCTAACATTTCTGTTTTATTGGTAAGTTTTGATATTAATTAATTCATCATGGCTGAGCAATTTCTTACTAAAGCTTTTCATCCTGCAAAAAAAAAAGAACACTTACTCATGTAAAAGCCCTTATTCTGATAGCCACATAATATTTAACGGAAATCGCTTAGCAAGCCATCCGACATAGTAGGGCTATATAGAATTCGCATCGCGCCGCAACTCGAAAGTTCGCCGAGCAATGTTGGAGGAGCTCTCTGCGGTTTCAGTCGCTGTCGGCAATTCAAACGCGTAGCACACATCGGTCGGAATAATTAGAATGGAGAAGAACTTTCAATACGAGAATATGCAGCCTCAGGCTC

>ID_368 P_368 Promoter_371 D1_Promoter for RH58267, -250bp and +50bp []

GACGAATATTGTTAACAATAAAACCGGTTATCCCACATTCAAATGAAACAAGCTTTCATTCAAACAAACTAGCAAGTCATTCATTGTTTTTGTTAGATGAATTCAACCACAAATCGTAGCTAGTCGAACGCCAACAGCGGGAACGCCACCGTGCAATCTAATCAGTTCAAGCAGCTTTTAGGTGTAATTTTTGCTACAAAACCGGCTGATAGTATGAGCTGTTTATATAGAAATCTGCGATAGGTGCAATCAGTGTGAAACGAGAGTTCCGTCAACGCGGTTGTGCCAAAATGATTATCTTGTGGCTGATTCTGGCCCTAAGTGCCCTGCTCTACTGGCTCCACAGAGCC

>ID_371 P_371 Promoter_374 D1_Promoter for RE21622, -250bp and +50bp []

TAAGTTGGTCTGGATAGTGCGTGCCACGGAAAATTAGCCTTGCCTTTGTTTCACTTGCATTCCACAAAGACGCACGCACACACACACGTGCACAGAATGACCCTTACAGTGGGGCTTTGGTATTGGTATTTGTATTTGCGGGTCGAACGCCACGCTCTTTTGGAGCGAGAGTGAGAGAGCGAAGAGAGAGAGAAACATAACGGGCTAGTGTATTTATTTTAATATTAGCTTTGTGACGTTCGCTCACCAAATCAGTATTTTTCGTACCATCGGCGTTAAAACACATGTTCAGCGATTTAGTGCGGGAGTGTAAACTAATCTGAGTAACAACAACAGCATCGTCGGCAAAG

>ID_372 P_372 Promoter_375 D1_Promoter for RH23096, -250bp and +50bp [3 ]

CACTACCCAGATCTTTCTCTTACCGCTGGTAACTATGTATTATAGTTTGGCTCCACTCTGTGCTCGCTCTTTCGCTGCGAGTGAACCGTTCATGAAGACGATCCCAAAGAGGAGCCCGGAAAGCCGCTTGGCTAACCTGTTACCATGGCTATAAGTGCAATAACAAAATCACCGCGACCACGGCAATACCAAACCGGTGGAGACGATCGCGCATTCTCCGCAGCCTCGGCTTTATAATCGGAATTCCGACTTCAGTCTCCGATTAGTCGTTCAAGGCGAAGGTTGTGGCACTAGCTCTTGGATCCACCAATTCCATTTCGCAGCGCAGATATAGTCCAAAGTGCAGGTCA

>ID_373 P_373 Promoter_376 D1_Promoter for RE14074, -250bp and +50bp []

TGAAAGACCAAGCCCATGATGACCAACCTCTAAGAATCCTCGACGATGGCCGTTGGGGTTGTGAGTTTCCTATCGCCTGCGAAATGTGTAATTTGCTGTTCGCAAAAGGTTCTCAAAAAACGTGGTCCGCCGAAGGCCTTGGCTCCTGCTCTTAAGAACCTCTTATCGGAGTGCTTTTTGTGGGCGGGGGGGAATGAGATTCGGATGGCTCTCTCGTTTTCTGGCGATGCTGTAACCCCGAACTGCCCGTCAGTAGAACGAGAACTGTGGCCACGGAAACAAAGCGGGTCTTATAAACATCAATTTCGCACAGCAAATAACGTATATTAAAAGATATTAAAGTTTTTAAA

>ID_374 P_374 Promoter_377 D1_Promoter for GH07619, -250bp and +50bp []

AACAATAAATTGCTCCATTTCTTTCTGTGTATTAATATTTTCAATTATCGAATGTCTCAATTAGTTGCTGGCCTTAATTCTGGCTTTGCGAAGTCATCGCACTTCCGTCATTGCAATTCAATATTCACCATATATATGTAGTAAAACTTTCTTAATATTTCAAATTGCGGGCCTTGTTCGATAGCGTTGCCACTCTGGTTTTGCTGAGCTGCATAGGCATCGATAATATTTGTAAGCAAAGTATCGATTATTTTCAGATACTTTTGTCCATCTCTAATTTACCATACTTTTATTTAGGAAATATTTACAAGGAATAAATACAAAGATTTGCTTATTACCCGTGGATTATA

>ID_375 P_375 Promoter_378 D1_Promoter for RE38254, -250bp and +50bp []

AACTTTCCAATGTTCAAAAATTTCACCGTTCCGCCATCGACTCTAACTCATCGACGCTAATTTGATGGTCTTGCGTGCATTTTGGAACACTAAATAACGAAAAATTAATTTTTAAAAAACTTCCCCATGTTCAAAAATTCCACCGTTCCGTCATCGAAGTTATTGACGCACATCGTCCTTATTTCTCCAAATTCATAATTAAACGCATATTAAGATTAAAAATTCGCTACGAGAACGAGCACATGTATTCATCTATCTGGCAACGCCACCAGTTGATGCAACACATTTTTATCTTAAATTGCGAAGTTCCTCGGCCAGATATCAGTTACGAATGTAGTGATCAATCTATC

>ID_376 P_376 Promoter_379 D1_Promoter for RE14234, -250bp and +50bp []

TTAAAAAACGTTTTTTTCTAAAACTTTCTAAGTGCTATAAACGCAATAACGGAAACATGGAAAATTCATAGTTCGTGAAGAGAACATCCGGCGGAGGGGACTGGGGAAACCCGGATCAATTCCAAAAAACCGCCAAATGAGCATGACTGGTGTGCGGTGCGCACTGGGATTCGTTGTTGTGGAACCCAAGTCGCGGTCGACGTCGCGAATTAAGCGCTGGGCCATCGGGCCTGATAAGTCCGCGTCCGCAGTTCCCATTCAGACGCCGGTCCGTCCGGAATAAAGTACGTGTGTGTTCTCTTCATCTTTTGGGGCGGTCGTATATACGTCTGCGTGCTCGTCTCTTCGTC

>ID_377 P_377 Promoter_380 D1_Promoter for RE39686, -250bp and +50bp []

ACCAAGGGAACCGAACTGGTTCCGTTAAGTACGAACAATGCGGGGAAAGTGTGGCAACGCCGATTGTGATTCATCAGATGTATAACCGAAGTCAACATTTTAAAACAACAATTCAAATGTTAACCATTTAAATACAATAAACAAATTATTTTAAAATTTGGGACAATCTTTAACAATTGGGATACTAGTTTTGATCTAGGACTGGTCACACTATTAAGCGACCTGTCAACTCGTTGTATACAAAGTTGCCATATAGTCACAAAAATCTTTCGCATAAAGAAGTAAAACGTAAGAATTTTCATATGCGTTTTTCGCCAGGAAATCCGCATTAAAATAATGTAAAAAGTGGT

>ID_379 P_379 Promoter_382 D1_Promoter for RH32847, -250bp and +50bp [1 ]

TATTTGCGATGGATCCTAGGGAAATGATCAAAACCAACACATAAAATATTCGACTAACCATCTGAAAGTATGCTACTTTTTCATGACCACGCTGCACTCAACCTCTCAATTCTTTTAATTCAATTGATAAGAACCAGGGGAGCTCCCCTCCCTCCCTCTCTCCTAACGCATTCAATTCAGTTTGCGACTGCGATAAGCTGCCCACAAAAGCCGATTTGTCCCGCTGGTTCAGCATTCAGAATCCGCCGTCAGTTGATAATTGACTTGCGACTCGGCCAGTCAGTCGTTCCATCGATCCGGACACCTACCAAGTGAGTACCTTCTAGTGGAGATGCTATCAGTGGATCCGC

>ID_380 P_380 Promoter_383 D1_Promoter for RH13487, -250bp and +50bp [8 ]

TCTGATCCTGGTCCTTTGTGAAGCTAGCAAAAAACCCCCCGCCCGGTGAATGCCCACTTAGATCCTTTTGCAGGAGGATTTCGACGGCCCTGAAGGTGGGCGCCACCCGTTTCGTGATGATTCACTCTGAGCAATAATACCAAAAACATAGACCTTCCCTCTCTTATTGGCTCTCCCCAATGCCTCAAGCTTTTTCGAAGTTCGATTTCACAACAGGCGGATATAAAAGGGCTGCAATGTGGGAGAGCTGTTCAGTTCAAATCACCGGCCGCATTCGCTACACTGGCTTTGTCCGCCGACTGAACCAAGATTAATTTGATCACCTAACCTCACACAGCAGCGAAAATGGC

>ID_381 P_381 Promoter_384 D1_Promoter for RH41996, -250bp and +50bp []

CAATATATATTCCCAGCTGATCTCTGGACTTGCTAAAAAGCAAAGTATGAAGTCATAGTTATAACTGCAAAATTGCATCAGCCCCTCTAATGAACTTAACCCAAACTACACAGCGTGTATCTCGGTCATTAGTGGGTTAACTAACGGTCGGTAATCGATAACGCCGACGCCGACGCAGCCGCCGCCGCCGACGCTGCAGCAGCGCTCTCTGCCGACGCCTGCCTTCGTCGGCGCGAAAACAAAACAAATCAGTTTGAATTCTGCCTTCAAGCGCTCTAGAACAAATCAGCTGGCATTCTGATTGGAATACTAACTATTTTTTGTTTTTTTGTTTGTTTACAACTCACACT

>ID_382 P_382 Promoter_385 D1_Promoter for RH02301, -250bp and +50bp [5 ]

CCGCATTACAATATTGCAATTCATTCCGCTCGAGATTGCATTAATTAAGTAATCCACCTGTTCTAGTAAACAAATCCATGCAGGTTTAGCTTAAATATGCGATCAATAACCAGCTAGCTAAGATATTCCGATAATATGGCATTCGAAATAAACAAAATACTAGTTTGGCTTATATTTAAATTAGTACAGGATTTTCACCTGATCCACCTACCTGAGCGCCCTATAAAAAGCCTCCGACTGGCTGAGCTCAGGCCATAATCAATTTGAAGATCGTGTAAGATCGACGGAACGAAGAAATCTATATCGCGTGCGAGGAGAGGATTTGCATAAATTGAGTCTGAGTGATAATG

>ID_383 P_383 Promoter_386 D1_Promoter for RH66046, -250bp and +50bp [0 ]

TGCCGTCGCCAGCGTCTCTGTTATGTCACCGGCAACCGCGAGTGATAAGGCACGGGTCGGTGGATGAAACCGACTGTCGAACTCTCCTTACCCTTAGGGTTCCTCAGGCCCATGCTAGTCTGTTATTGCCGTGATGAGGGAAGCGACTTATCAGCGGCATGGCTCTCGCAAACAACCCAGCACACTCGACTGTAGCGCACATAGTGACGCCTTCCCGGGTCTATTATATCGCTGGGAGCTCGAGCCCCTCAGTTAGTCGTTGGTCAGGCGAGGAGTTAGACGTGTTAGAGGAAAAAGAAACTTTTAAAATTTGCTACCAATTGGGAATCCCAAAATGATCAAAACTTCAG

>ID_384 P_384 Promoter_387 D1_Promoter for RH27666, -250bp and +50bp []

GAGAGCGGGAAACTCGGAGAGCGCTGCTCACGCAACAGGTTAATTGAGAATTGACCGTTGGGCAGAGCTTTTCCGCCAGCATCAGAATGTATCCGTGAGATACACTCATGCGCACTCGCTCTCCGCTCTCCGCTCTCTTTGTATCTTCAAAACAATGCAACAACCTCTGCACACTTTCACTTGCCAAAGCTTTCTCCGCAAAGCTTGGCTGGCTCCGGACTTTTTCGCCACCTTACTGTGGCCCAAAGTTCATTCCGTTTCGGATTGTGGCATTTTTCAGGCGTGCGAAAAAATTCCTCAGCGCTCCAAGAATTTCCACAGCAATTTTGGTCACTGCTTTAAAGTGAGCA

>ID_385 P_385 Promoter_388 D1_Promoter for RE66512, -250bp and +50bp [8 ]

GGTGTTCTGTTAGTCGTGGAAATTGCAGTTTCCCTCTAACATTTTTATGCTGCGGGAAGGCCTCACACTCGCACACAAACACACATGTGTCTTATTTGGCTTTTCCAAACGCAGGTAGATGCCTTACATGCATATATACATACACAAAAACAAGAGAGAGAGAGTCGGAGAGGGAGAGCGCGTCGGTGTGTGTGTGTGTGTGTGTGGGTAACTGTAACTGGCCGTTTGCTTTTCGTTTCGTTTTTCGTGCGCTCAGTCTCTGATAGCCAACTCTCGGAAGCAGACCGATAAGCGCTTTTCGGGCAATTCCGCCAAACCAAAACAACTGAAAAATACCCAACAGCCAAGTG

>ID_386 P_386 Promoter_389 D1_Promoter for RE35907, -250bp and +50bp []

GGGCTAATGTTATGCAAATACGCAAAACGCCCACTAACATGAGCGCTCCAAGCGTTAAAGCCCTTGATAACAAGTACTCTCCCTGTCAGATATGTACATTTATATATTAGGATCTCTCAGACATTGACATATTATATTGGTCAAAACATTTACATCTTTATACACATATCTACCTAGAACCAGTGATATACCATCTGCAGAGTGTCCGCGAAGACCGACATAACTTTCACATATATCGCGACCTGGCAGCCCTATTCTTTACATAACAAGAAGAAGAAGACTCGTCGACCTCACCGGGATCCAAAAACTTAATTGCATTTTCACCGATAATAATGGCCGGGACACAGTCA

>ID_388 P_388 Promoter_391 D1_Promoter for RE03794, -250bp and +50bp []

GAACAAGCCATTGGAAAATCTACAGTGATGATTGTTTTAAACCGAAAATGCCTTCGGTTCGCATAAGCCCAAGATTATAAATTATTCATTAGTTTTAAAATGTTTTTGTTTACAAATTGTATTTTGTAAACAAAGACGACCGATAGTAATCGATAGTGGTTAATTTGAAATGAAAATTGTACTATCGCTGATGTATCCCATCGATAGTTTGTTTTCTACGCCTTGCCATCTCTAGCGTCTGATATTGATGGCGTTAGCCGGATACGGTCATACTGTCATAAGAGAAAAAAAATTATTTCGCGCTCATATTGTTATTTTTCTTGGATATTTGTTGCAATCGAGCGTGTGAA

>ID_389 P_389 Promoter_392 D1_Promoter for RH16704, -250bp and +50bp []

CGCGATTCTCGACCCACTCAGCTGGCTGGCTAGCTGGTTAGCTGGCTGGTGAGAGGAAACCCCACGGCATCGGCATCTTGGCTTTAACCCAGCTACAGCTGGGCAACTACGTCACAGTCGCGTCATGCGAAAACCAGTCATCGTCGTTTGGTGAAGCAAACAATCGTCGGCAATCGGCCGAAACGTTCGTTAACTTGGCCGTCGGAATCGGAATCTGGGGGTATTTAAGCGGCGGCAACTCGACATGAAGCATGCATTGATTCGCTATCATTCGCCCTAAAGACTTGTGCCCAGCCTCAAGTAGTTTGCTGACTCTGTTTGTTGTAATACAATTTAGCTGACAAGGATAA

>ID_391 P_391 Promoter_394 D1_Promoter for RH38008, -250bp and +50bp [7 ]

AATCGCTGCCTAATCGTGTTTACTTTATTCAGAACGTAGTTCCTAGATTTATTTGCTATTCCGGCATTATTTTCAGTACAAACACTAGCACTAGCACATAGTACTCGAAGTCTCGTCTGTTTATATACCAAATTCACGAGCACTCGTTAATGAACCGAAAGTTCATGAGCATATAGATAAGATCCCCAGCCAATATGTAGTTCCCATCTGCCGATCGGCATGGCGTATATAACCTAGTGCTACACCTCCGATTTCCATTAGTCGTTCCACGGAAGCTGGGAGATATACATAGTTTTCAAAATCGCAATCAGTATGAAATTCATTGCTGCCGTCTGTTTGATGTTCGCCCT

>ID_392 P_392 Promoter_395 D1_Promoter for RH52407, -250bp and +50bp []

CGTTCTTAGAGTAGGTGTGCGGTATGAATGGAGATGATTGGGAAACCTCTCCACTTGGCTCGGAGCTCTCTTCAGTGCTTGGCGCATATCTGTTTTGGACTGAACTCTCTGGGGGACAAGAGATACCCGCGACTCGAGATCTCTGAGAGCGGGAGCGCGAGATCGGGGCAGTGAAGTGGTGCTGGTGCTGGTGGCGGTGGTGGTGGTGGTGGTGGTGCTGCTGCTGCTGCTGCTGGTTCGGTGGATCGTTCAGTGAGTCCCCAAGCGTTGTCGCGTCCACTTTATCGCTGTTTCTGTCGCGGATTCGAACCACTGACTAACGGTAGTGCTGCACGCATCTGGCGGTAAAT

>ID_393 P_393 Promoter_396 D1_Promoter for RH26620, -250bp and +50bp []

TTCCTTACTCCCCATTTCCTTCCCACTTGTTTGGCGCTGCACTACCTCCAATTAGGCAAGACTTTCTTGTAAAATCACATTAAAATTAAATCGGATATGGCAGCACCAGAGCGCAAAGTATGTGGCATTGCTGCCAGGTGGCAGTCCTAGCTGGCAGCGCCACTATCGATACGCAGCTATCGCACCAAAGCAACCCTTCGGCGAACTGTCAAATAGCCACGCGAGCCTAGCACGCCGACTTCCGTTTTATATTCGTTCTTTCGTTCTCAAACCGTCGTCCAGACCAGAAGGAAAATGAGAGCTAAGGTAAATTATTGTGACAATTAAATTTTCCCTTTCAAACATAGTGA

>ID_394 P_394 Promoter_397 D1_Promoter for RH43343, -250bp and +50bp [5 ]

AAAATATAAAATATAAAGAAACATAAAATATGAAGTTTAATTTTTTAAATGTGCACACAGAGCATATCTAAATGGCACTTTAAGCATTTTCATTGAACAAAAGTTCGGCGCCTTTTGAATTTTTAAATTTGCATTAGTGATGGACCAGAGTGCGCCTAACAGCACACGGCGAACTATTAACAGCACGCGTCCCGAACGTGGCAGGCAATTTTAGGTTTTTTACTCCGCCTCAGGCAGCCACATTAATTCGGCCTTCTTTTCTTTTCCCGTTTCCGGTGAGTGGTGTGCTGCCCATTTGATTTTTCCAAAATTCTAAAACAAAATACCTCTTAATTCTTTACAGTGAGGAG

>ID_395 P_395 Promoter_398 D1_Promoter for RE54715, -250bp and +50bp []

CGCGCACTGTCGCTGGTTGCTTAAATGTTTATGCTAACGGCAGCAACAATTGGCCGGCCCACGTGGGTGGATTTGGATTCGGATCTGTATTTAGCTGCGGCCGTAGCTATGAGGTGGCATGGAATGGGGATGGTGATGCCGATGGTGATGGTGATGGTAATGTTAATGGGAAATGGGGCCGGACTCTGCCTCTGCCGGCGTCGCAGCGGCCGCATCTCCGGACGGCATCTCCGGCGGAAAGTGAGAATTCAGTCGGAAAGTGAACGCCGCGAACGCCGCCGCCTCCAAAAATTGTCGCGCCAAGTAGTAAACCCAACTTTCAATCTATTCTAGATTCAACTGCCAAATCA

>ID_397 P_397 Promoter_400 D1_Promoter for RE66075, -250bp and +50bp []

TATTGAATAAATAAGTAGATATATGTGCGTATTTACTTACGCGAGTGGGCACTTAAGAATTTACTATTTAAATGCACATATTTCAGTAAAATTGCAAACAACGAGTTATAGAAAAATACTAAAACAACGTTACAGTTGTGGTCACGCTAATTCCAACTGGATTGTAAAAAGATCTCTAGCGATAACGATAGGCGAAGAGTAAGACCTTTTCTGCTGGATTTCGCAAACGATAACGATGACCACAATCGTGAGTTAGGTTGCGAACTTTGCTTGGTGAAAACGCAGCGGATTACGAGGTTGGGAAAAACTTTTAATTCGCGTTAAAAAACGGGAGTGCAGTGAAAAACAAA

>ID_398 P_398 Promoter_401 D1_Promoter for RH50566, -250bp and +50bp [3 ]

ATTGTTAAGTGGCGTTATTTATTTGGGTGCATACATAAGCATAATAGAAATTTTATTTCACAACTTGAGAAAATAAAAATTGATTTCGGACTTTATGAAAATTTTCGGCATTTATGAAAATATAAAGGAGATTTCAAAATCTCGATAAATTTGGGTTCAGTTCTTAGCTAGTTGCGCATCCAAGTTTCACACCTTGTCGCGTCCACATTTTGAGGTATCATGCTTGTATCGAGCGGCGGTCACACTTTTCGCTCTTTTCGATTTTTCGTTTCCGGCGTGTAAGTGTACAATTTTGTTGCTCCACGTTTTTTTCCTATTAATTTATATTTATCTCATTTTCACGTGCACAG

>ID_399 P_399 Promoter_402 D1_Promoter for RE70318, -250bp and +50bp []

AAGCCAAGAAGACAGCTTTACATTCGGTCAGTCAGTCAGTCAGTAGCCCATGCAGATTCCAACTCTATATATACATATCTATATACATATATAGATATATATGTATCTGCCCAGATCGGCAGACTCTAAGTTCTCTGCCGACGTCGACGGCGGGATGGAGATCGGCTTGGTTTGCATCTCGTTTCTGCGATCTGCCATCTGCGATCAGCAGCTTTGGGGCTTTAAAATCGGCTATCGACCGGCAAGCAGCTCGCAGTTTAAGTTCTCAGCTTCGCATCGACGGACGTTCGAGCACTCCACCCGCAGAAAGTTGATAGCTTCAGATTCCGACTTCGTTGCGTGCTGCTGCA

>ID_400 P_400 Promoter_403 D1_Promoter for RH51233, -250bp and +50bp []

TATAAATATAAAAAATTTACAAAAGATATTAAACATGAATTTTAACCCATATAATATAGTACAGTTATACATAATAATAATAAAATCCGCAAAGATCTATTTATCGTAAATTTCAAACATATACGATATTCAAATATTTTGGCGCCATTACAGGTACTGAAAAATACCAAAAATACCGTAAGGTAACTATCGATGACGGCGATCAGCTGTGATCATCAATGGCGAACAGCTGATCTGGCTCGCGAATTTTCATCTTGTTGTTGGACTTTCTGTTGTGGACTTTGAAACGTCGCCTCTCGAGTTTAAACAACAAAAGTTACTCAATTTGGAAAGCCATCGAGTGGATAGCC

>ID_401 P_401 Promoter_404 D1_Promoter for RH68618, -250bp and +50bp []

GCGACTTTGACATATTTAATTGTTATTAATGGCTAATATTACATACCTTTAACAAGAACGTAGATCGAACTTGATATAATCGTCTGATTGACTGAGATGGCATTGGAAAAGTAAAAAAAACCAGGAAGCAATTTAAGGTATCATTTTTTAAAAAAAAAGTTGTTACCCTACGTTGGACGTAACGAATCCTTACGTTTTGCTCATTTAACGCCCCTCGAAGTGTGACCAGTCCGCGTAATGAATAGGTATTTCATTGATATCTGCTTACAGCCACATTTGACTAAACACTAAATATAAATAATAGTTTGTGACTCAGAATTTAGGCTTCAGGAGCTACGCAAAGCTTTATT

>ID_402 P_402 Promoter_405 D1_Promoter for RH70978, -250bp and +50bp []

AATTAAGATAAATTAAGTATAAAATAATATACACTTCGAGAAATGGCAAAACGTTAGTAATATTATGTAAATATTAATACTTTTTTTCCATAAAGCTAACAAATTTCCTCTCTTAACTCTTTCGTATCAATAGTTTTAAAATAAATATAAATTATACGTAAGACTTTAGCATTTATTAGTTTTATGTGCCTACGCCCCTGAAGTTTTGCTTGCCGTCTATCGATAGCATACAGCACTTATCGATTGTCTAGCCTTTATATTTGACTTCCAGCTGACGGGCGGCAATTATATTGAATTTGTTGTGGTTTTCGGTGGAGCGCGGTACTTTCGATGAAAACAAGAAGATGGAA

>ID_403 P_403 Promoter_406 D1_Promoter for RE01951, -250bp and +50bp []

ATCGTTATTTGCTATGGCGACAATCGTTTATCTAAAAAGAGGTAATCGATAACCGTGACTGTGAACGGATATCGGCTGTCACTTTATTTTGATAGAACATTACAAAACTTTGAAGTTTTTAAATACCTTGTAGGGCTTGAATATGCAATCTTATAATATCTACTTTTCCAATTTGTTTATCAAAATCTCTTGGAATAAATATTATTTTTCTGCCCAAACGATACGATACACTTGCTTCCAAAGAATAACTGCACTTCTGTTATCGGACATCGCTAGAAAATCCAACTTTTCTGCCCGCGCCGCGAATTGTAAGAGTTTGGGGAAAAGTATAAAATATCCAGATAGAAGCA

>ID_405 P_405 Promoter_408 D1_Promoter for RH03528, -250bp and +50bp []

CAAAGCCAAAACGTGAGAAAGGAATCACAGTGTTTATTTTTGGTATAACTGGCATAAATCGGCGGCGGTATCAAATTTTGTGCCCGACATGCGTTTCTCGATGCGGTTGTGGAACCACCGGAATCTTCCCGATCCCGCTCCGCCCGCCGAGATTGCATTCCCAGCTCAACTGATAACCACGACGAGTATATCAGCTACCAGCCATATATGTACGATGATCGGAGGTTTAATTAGGCGCGGCTGTAACATGAACATTCATAATTCCGCTGGCATTCAATCAATGCAGACGTGAACAATTCCAGTAGTGACCGAGTGTTCGACTTAGCCAGCGATCTCGTGCAAAGTGTACA

>ID_406 P_406 Promoter_409 D1_Promoter for RH14383, -250bp and +50bp []

TTGAGATGACACTTTCTAGGAACATCGGTAACGCAACTTGCAACAGCACATTTTACACAACACAGCTCCGCTCACTTCAAACGTATTATACGAAGTATTAAATTAAAAAAAGTATTTCCTTTAATATTATTTAAATATCCAATGTGACATTATAATTATATATAATTACCTTGTGCGATAGTTTTCTCTGACGAATCACGACCATACCAAAGCACACCATCAGCTCTCTGCGAGGCACTTTCCAGCACTTACAGTCAGCCATTTTCTATCGGGCATCACGGATGCTTTTTTTCCCTGACCAAAGGATATTTGGTTTTTCCATCAAAATTTGTTGATTTCGAGACGTCAAA

>ID_408 P_408 Promoter_411 D1_Promoter for RE19936, -250bp and +50bp []

TTTTCTGAGTATTTTTTATACGTTTGCTATATAAGTTTAAAGAACAATTCAAAGGAAGTGCATTGCTCTTTCGATATGATTAACATTAAAAGCGCATTCTTTTTCCCTATCTCTTTCGCTCCTGCTGCTTTTCCGATCTTTTCGAATTCGAGGGTTGCTACAGAGAGAGCGTGAACCGCTCTGAATTTCCAGTTGGCAACGCCTCTGGTGGTTGTTTGACGTTTGCTTTTGCCCAGTGTTTTATTTATCTTTTATTCGTTTCTCCGAGTTCGTCGAGAGAGGTTCGCTTCGATCGGTAGCGCTATATTTAAAAATCTAGTGCGCAGTGCGCTCATAGCCGAAAAGGTTAA

>ID_409 P_409 Promoter_412 D1_Promoter for RE11225, -250bp and +50bp [4 ]

ATATTTATCTTTTTTACTGAAACAGTCTCAAATTATTAAAAGTTAGAATGGCCATAAATATACGTAAGAAACATCAATATTATAGCTATAATAATTTTGTTCGCCTTGTTAATATTCCCTGCTCTTAACTAACAATAAATCGAAGAAAACATTGAAATTATCGATACGTGTATAGTAGGCGCTGCCACTCCCGGATCAATCGTTACCACTCCCAAGCGAGTTGGCAGTTCTTTCGCCATTTTTTCTCGCTCATTCTTTTTGCTGTTCGAGTGAGCGCGTGGCAGGTCGTTTCGCAGTGTTCCCGGTCCGGTGCAGTTGTAGTTGCATTTTCGCCAGCGATTTAGACGCCA

>ID_410 P_410 Promoter_413 D1_Promoter for RE35988, -250bp and +50bp []

GCAGGCGAATGCAGATTAGGAGAATCAAGGGATTATAATTAATCAGACGTCTTGGCTCTTACAGCAAATATCGCATGCGATTTTATTTCCAAGCGACTGTTGCGCGCTGTTCAACAAATTCTGCTTTTCTACCGAGCGCTGTTTCTCTTCCTGCTGTTTCAAGTTTTAATGTTCCAATCTCCAACGTTTCCATCTTTCCATTCGTTCCGGAACGAACAATTTCGAACGCACAGTTCGCGCTGAAAATTCAGTTCGTGTTGAAAATTCTGCCGCGTCGGTCGTGCCAATTCGAACGAGCGGAGAAAACTGGCCAGAACTAACGGTTCTTGCCGACGGCACATAACGGTAGC

>ID_412 P_412 Promoter_415 D1_Promoter for RE71693, -250bp and +50bp []

GTGTTGTAAAGCGATCAAGCTGCCGATTAATTATTTAAAACCTCTTGGATTCAGTCCATTTGGATGCAAATAAATGATAAAATGTATTAAATAAAACTATTTTTACCATTTAATACGTATTTTCCCACAAATATAATATTCGGCATATTTGTGTTCCATTCCCACACATTTCAATGATATATAGATAGATATTTCGATAGGCGCTACAAGACCCACACATACCAGCCCCCTTCGACGCACATCCCTAATTAATTTTCACACGCCATTTTACAATAGAATTTGTGACGATCTGACGCTTTCTTCAATTCGAGCAATTTGTACGCAGCGAGTTATTGAAACTCCTGCAATTC

>ID_413 P_413 Promoter_416 D1_Promoter for RE08920, -250bp and +50bp []

ATGAACAACGCCGGCTTGCCCGTCATGAAATTTGTATTTGTATCTCAGCGGCTGTTGCTGCCCTCGTTCCCTGCCTTTCTGCCGCTCTCTCTCCTTTCACTCTCTCTCCCTGTTACACTCACCCCCGCCCGACCAACACGCCGCACCACACCACTTCTTCTTCTTCTTCTGCTTCTGGTTCCACGCTCTGCTCTGCGTCTCGTGCTCATTTTCCGTTGTACTTTTTCGATGCAGTCGCCCCGTGGATTTCATTCCCTCTGCGCACTTCGAACCGATCGCTCGTATCGCTCCCGGGACTCAAAATCCGAGTACGGATTCGAATTCGAAACGAACCCATAACCGAAACTCAA

>ID_414 P_414 Promoter_417 D1_Promoter for RH41583, -250bp and +50bp []

TTTATGTATCGAATTGAAACTATTGTTATAAGGATAAAGATCATTTTGTGTACGAATGCGATCATGTTCATTTATTTCTACATATTTGCTCTATAGTTCGAGCCGCTCGACAACCCTAAACGGATCGCTCTCGCATTTTCCCACGAGATTCTTTTGGTTGTGTGGTGCAAAGAGAAGCGGAGAATCGCTTTCCAGAGAGCACAACTGTAATGAAGAGCTTTCGGCTGGCGCGGCGATTCATCAAGTCTTTAGTCTTCAACTGATCGCTCGCGCCGATGATTTAATTTTTAAGTTAAAGCCTGCGCCCAGTGTAGCAGCCGGCAGCGTTACTCACATCCATATACATACAT

>ID_417 P_417 Promoter_420 D1_Promoter for RH09802, -250bp and +50bp []

CCTAATCGATCCTACAAGCAAAGATAGCTTTGCTGAAAATGCTGATAATAGATAGGTGTTTTGTCTTCAAGAACACAGAATCCCTCTAAAAATGGCATATTCAAATAAATATTATGCCAATCAGAAGGAACGCTAATTATCTCGAACGACAAGAGTGAGTCAGCATGGAATACGCAAGTAGCGTATATAAATTGAAATAAAATGAGCTGTAAGTTCATCCGATCGATAATTGCGATAGTCGGAGCTGGTTTAGTTTCAAGTCAACAGCCAAGGCGAGCGGACACCTGAAGCGATGGGAAAATTAGTACTGTACGGCGTAGAGGCTAGTCCGCCGGTGCGAGCATGCAAAC

>ID_420 P_420 Promoter_423 D1_Promoter for RH04511, -250bp and +50bp []

AAGCAGACGACCCCCGCCAAGTGGTGATAACGCTTAAAATGGAAAAATATTTCCTTCCACACTCGCTTCACTCTTGGTTTCTATCGTCAAGTGAAATATAAGCGCGATAGTGCGAAAAATTTAGAACCCGCAGATTAAGATCGGGCATAACAACAATATTAAAGCGAGGTAAACAAAGTTGGCGAAGAAGAGCGAAGAGAGCGCGCGAAGAGCGGGCAGCCAAAAGCTCTCGCTGTAGATTTAAGCTTTCTTCAGTCTCTCGCCGAACGTCGAATCGAGCAGCTGTTCCGTGGAGAACCGGCTGATCACTTCCAACAGAAAAGACAGAACCGAATCGGAATTATGATTGC

>ID_422 P_422 Promoter_425 D1_Promoter for RH66581, -250bp and +50bp [6 ]

CCTGCCGCTGCCTTTGGCATTTTGCGAGCAGTGTGGTCACACTGGCGAGTACTGTAGTGTGGCGGAAATAGTAGCGGTATTCGAACTGAACCAAATGTGCGAATAAACAAAAAATCTGCAACAAACAAGAGTGTTAAGCGAATATTGAATTTGTGCTTAATATATATATAATTTGTATTTAATACAAATCAAATATTAATAATATTGGTGACCTTTTGGTGCCACTTAAGTTTGAAGTATTATTAGCATTCCTCTATTTGGTCACTCTGCAAGTCTTACAACGTGAGCAGACAACAACAAAATTACATGTTTATTTGGAATAATCACAAAAAACAAAAAACTTACCAACT

>ID_423 P_423 Promoter_426 D1_Promoter for RE72615, -250bp and +50bp [-1 ]

GCCGTTACAGGATATTTTTGAAGAATTATCGGTAATTATCGAAATGCCCTAAAAAGTGGAAACAGTGTATTAACGGATACTTTACAATGTATGTATCACTATTATACGTTGCATATATTTCTTCTATTGTCCTTTTGCTAATATATTATGTTGCATACAAAAACTTTAACCTATTCGGCAAAATATACAATAGCTCTTACATGACAAGTGTTTGCAGCTTAAAATTTGAAGTGTACATTTTTCGCTTTCAGTCGACTGGTCACACCGTTCAACCCAAACTGACTCGGGAATTTTAGGGTGAGTGCTTTTATTTTTATTTGAGGTCTAAAAAAAACTTATGGAAGTTTACC

>ID_426 P_426 Promoter_429 D1_Promoter for RH54083, -250bp and +50bp [0 ]

TCAGTTTTAGTTTAAAATTAATAAAGTTATGAAATTTTTTGGGACAACGCGGTCTTCAATTTTTGCAAATTTACCCATTCAGATGGCGTATTGGTGGATATAAACATTAAGCAGATAGGTGGCGCCTTAGCAAACGAAAATTCAGTGTTGTGAAGCGCAAAAATACGAAATATCGCAGTGGCATCAATGTAATCGATAGTGCCATCAATGTATTTCCCTATGTGCGATTTTTTCCTTGGCAGCCGTGGTCACTCTGCTGTTTATCGCCGAATTGAACGCACGTAGCGGACCGGACGGATATCGCATCTTCCGATCGGAAAAATCGTACAGTGCAGCCAATTCGCCGTCTA

>ID_428 P_428 Promoter_431 D1_Promoter for LD37564, -250bp and +50bp []

CCAGCCAGCGAACCGCTTACCTTCACAACTAATTTCGACTGAGAGTGGTGTGACCATAAACGGAAGACCACCAAATACCGTTTTAGGTGATATTTAGTACATTTGTCTGTTACAAAATATGGCAACGCTGGTGTAGTCGATGCACCCACCACCCCTTATTTAGACACAAACCATATGGGATTTCTAAACGCTTGTGGTATAATTTAGGAAATTATTGACATCAATTCGGTGGCAGGGCCGACGATAACGATTTAGTATAAAAGCACGCCTGTTATCGGCTAAATTTACAAAAAAAAAGGGAAAATTAAAAAATTAAAACACTTAAATAAACGCTTTCCTGGGTTAACCGC

>ID_429 P_429 Promoter_432 D1_Promoter for GH11843, -250bp and +50bp [5 ]

AGGAGCCCCAATCGAGTGGCACTGGGGGTGAGTGCTGGCGAGAACAAAACTGGTTGCCAGTCGGGTCGGCGGCTGGTCAATCATATGGCGAGAATAGTGGTTAAAGCCACCCCTGGCGTGGCAGCTTTATCGGGGGTTGCATGTGGTGTTGGGATTGCACGGTGTGCCGAGTGTATCCGATTCCGACTTCTATTCCGAATCCGAATCGGCGGACCTGGTCTCGAATTCGGAAACGCAATCGGTGGCTCGCGCACAGTCAGTCTGCAAACTTCGGAGCGTGCGGTTGTCAAGTTTCGGTCGTCTGCGTGTTTTCTCTTTCCCGATTTCAGCACTCTTCCCGCACACACACA

>ID_431 P_431 Promoter_434 D1_Promoter for RE48083, -250bp and +50bp []

AATAGCATTGTGCATTGCAAATAAATTATATGTGTACACATATTTTTGTCAATTATTTCGTTCTAATTCATTTTAGGGTCCCCCAATTTAGGTATGTATGCAACACGACATACTGCTTGCAGTCACTGCATAACTCGCCAAGCACTGTAGTAATACCACCCCACCACGTTTAAACTGGCCGAATGCGCGCGTCGGTGTGACCGCATGGCTTTGCTGGCGAAATACCGGCCGCCCAACTAAAAATCCATTAACTTGTATTAACGTGTCAGACTCTGACTATGCAATCGCAATTGCAAAATCTTTAAGTAGTGGCAATAAATTAGTTTTCCTTCTGCACAAGTCCATCCCTA

>ID_432 P_432 Promoter_435 D1_Promoter for RE56117, -250bp and +50bp []

AGTTATTTTGTAATTAATAATTAATAAACAGTGCACTACCGTTTTAATTTAGCCTAGCTTCCAATCGGAACTGATACTGATACGAAGAACAGCTGGTGCAATTCTCCAGATCTGAAATGAACGAACTATCGAGTTATTACATTATCGATAGAGAGTTCGGCTCAGTGAGAAAACCATTGAGCACCCCTATCAATTCGCAATGGCATATCCAGTATTGTTAATAGCCCCGCGGCCAGTGCTTGTAAACGCGGTCAGTCCAGCCATAGTAAACAAAACGCGACGTTCGGCTCGCTGCGCATTTTTAATGCAATAAAAGCGAAAGGATTGCAGAAATCACGCAACTGGCGTTG

>ID_433 P_433 Promoter_436 D1_Promoter for RH05303, -250bp and +50bp []

CATCCGCTGCTACCGTCCAACGTTTGCCAAGTGCACACATCGTGCTTCGGTCGGTGTCGGTGGCGTATGAGTGATGTTTGAGCACGCCCACCAAGTATCCTGCAAGGAGCGCAACATGTGGTCCCACGTCCCCCATCACCAAGTTTTGTGCGAATCCTGCGTGTATATTGCCGTCAGCCAATCAGAGGCCACCGCGGCATCTGCGCTCGTCCTCGGCGGAAGTAAATAAAAGCGGATGTCCTGGCTGGACAGGACATCATTCGAGTGCTTGCGTTCAACAAGTCCAGCAAACAGAGCAGCAGCTGAACCCCGGTGTTAACAACTAACAAGTTTGTCCATTAACTTCTTTG

>ID_434 P_434 Promoter_437 D1_Promoter for RE65325, -250bp and +50bp []

CATTGATCATTTTAAATGCATTGCGTACTATTTGAAAACCTTCGAGTATTAACAAATCGTGATGAATCCAAATGTGATCTAAATGTGATCCAAATGTCAAAGAAGATTTTTGTTGAAGCTCCTATTCTCACCACTTAAAGCGAATTCCAGCAAAGAGAGCAGCAGTGAGCATGCGCAGTGAGAGCGCAGAGAGCGCGGCAGTCTGGTACGATAGAAATTTTATTTAAGCCACAGACAACATGAAACGGGCACTATTTCTGTGGCGTCGCGTGTTCAGTTCACCGCGGGTAATTCAGAGAATCGCTTTGTGGATTGGATTTTTGCCTGTTTTCCGCCCGATACAAAAAAAA

>ID_435 P_435 Promoter_438 D1_Promoter for RE66660, -250bp and +50bp []

ACGCTCTCATAGGAGCGATTTCCTTTTAATGTCTTACTAGCTTTGTTTCATATAAAGCAGTTATCTCTGATGATCCTACTTCAAAAATCCGGCGAAAGAGGTGATAAGAATTATCTTATGCTTTTTTTCGATATTCTTGTGATCTTACCTATATTTATAGCTATATCTAGCTAGAAAAGAGGCCACCTCCGCTTGGGAGCAGTGAGAGTGGGTAAAGAAATCATAGGCGCGCATTCGCACGGCGCGTTTCAGTGCTAATTTTAACGTCGTCGCTGACTGTTTCGCCTTATTGCCCTGCTGCGGTTTGAGTTTCCCTATTTTCCACTTTTCCACTGGCCGCGAGCACTGAG

>ID_436 P_436 Promoter_439 D1_Promoter for RH70241, -250bp and +50bp [2 ]

AGCTTCCCAATTTGAATGCCAGTCACTCATTGAATCCTATTTTTCACTTGCTCTGTGGGCGACAAATTGGTGGCGCTTCCGTTTGGGATTTGCCGCTTGATTCGCTTGTTTACGCTGACCAGCGGGCTGCCTTTTGGCCGGTATTAGAGTGCATAACAAATCACTTAGCACCCGTCCATCAGGGACCTAATCAGGCTCGCATGGGGCCGCTAGCATTTGGCTATATAACTTGCTATCTGCCGCGCTGCTGGCATCAGTTGTCCCGCAAAGCAGTCCCAAAGCGCAACGCGGTTCCATTCGATCGTCCGACTATTAAGTTAATAGTTCCAGCGCTTCCGGAGTGTCCTGTC

>ID_437 P_437 Promoter_440 D1_Promoter for RE42646, -250bp and +50bp []

CGACTGTTGATGGTAAACATCAATAAAGAATTGTCATAATCGAACAGCATCTGGTTTGGAGTGCAGCCAGTGTGTGGATCCTATTCGGTATGGTATCTGGGGCCTCCAGAGAAACTCTCGCTTTCCGCCACTCACCCACACACACGTGCACAAACTAAACGGCACTCCGGCCAGCAGCTCACACAGATACTCGATGGTAACGGACGGATGAGCACCGAGTCGAGGAGAATCCACCGCGGAGACGACGGCCAATCAGTCGAGAATCTGTTGGCAAACCGTGCAGTTCGTTCGGGTTTCCTTGCTCGCGTGCTTAGGAAGCAGAAACCAAAAGTAAAGCAGAAAGCAGAAGC

>ID_440 P_440 Promoter_443 D1_Promoter for RE35249, -250bp and +50bp []

CGAGAAGGAGTGAGATCCTTCAGTATACCAGTTGCCAGTGGGTTTATAGCGCTGTTTTATTATCACAATGAGCCATTATCACCTGGCGTACAGGTATTTATCAGCCAAACGAAGCTCGGCCGGTGAATCAAATTCGAGCGCTTCGTGTTTTCTGGCGCCGACGTCGCTGCCTCGTCGACTGCGAGAAAAAAAAAACTGAAACTGAAACAGAAACAGAATTATAAAAACGTTCAATTAGCCGAGGTAGTAAGTCATTACAGTGCCAACGCTCGTGGCGAAAAGAGCGAGTGAAAAACTCCAGAGAAAAGCTTTCCTTCATCGGTTGGCAGTGCGTGGAAAACTCAGCTAAC

>ID_442 P_442 Promoter_445 D1_Promoter for RH51922, -250bp and +50bp [1 ]

TAGATTAAACATAGCTATTGTAGAATTATTATTATCTTTAGTCGACTTGCATTCAATAAATATTTCCCAACATTTTAAATAGTAATATGTTTTTTAAAAAGTTTTAAATTTCGATAACAGTTATCGAATTAGGTTCAATCGGTGTGTGTCTGTGCTTTAGTTGCGGCATATTTAGGTCTTAGAGCTCCGTCCAATTTTAGTGCATTTCCAGCGGCAACACGAGGTATGTTTCTCCCGTCCGCCAACGGTCACTCTGCTGCTGTTGTTGTAGGCGAGCGGTCGGAATTTGCGTCGGAATTGTGTTGAGTCGAGTCGCGCTATTTTGTTTCGAGTGCGCTGTGCTGTTTGGT

>ID_444 P_444 Promoter_447 D1_Promoter for RH39879, -250bp and +50bp []

GGCTAGTGGTGGACAAAACAAAGGGGCCTATCGGTCAAGGAGGTTCGATAGTTTCGGAACGACTATCGTCAATAAAAACCAATCGTATACCAATCGCGATTTTTTCAGATAAAATTCGAATGATTAAAAATAAATCACCAATTCATGGTACTTTATTTATTTAGCACAGTTGAAGTTAATATCATTCTTTTCTTTTCTACTTATACATTTCGAATATCCCCTTACATGCTCTGTAAAGTAACATTTCGAACTAGTTCCGTTCATGTTCTGATCCGCGGCGCACGCGGTTTTTGTATTTATAAACAACGAAAATCATGCTGGCGGGTATCAGCGAGAAAAGGTAACATAAA

>ID_445 P_445 Promoter_448 D1_Promoter for RH42049, -250bp and +50bp []

AATAAAAACTCTAAATAACCGAATGTAAGCTTAATGTGAGCTTTTTTTATTAAGTAGTCTTGAATAGAACGAATATCCTGCAGAAACTTTTACCATTTAAAATGTTACTTGCAATGCTTTCCCAGTTAGCAAAGGCAATAACCTAACGAATTTCGTTAGCGATAGCTTAAAGCTACACTTTCATCCCCAAACGACAGGGAAACCCCACTGAGAAAAGCCCCCAATGTTCTGAACTTTCGGAGAAATTGTCAGTGCGTCAGAAACCGTGGCCGCGCGCTCGTCGTTTTATCCACACATCCCCCTGGCACACAAACTTCTCGAAACAAACAGCGAAAGAGCAGGAGAGAGCA

>ID_449 P_449 Promoter_452 D1_Promoter for RE72230, -250bp and +50bp []

TTTTTCCCACTGGCATTTAAAAGATTTTCTCATTTGACAGTTAAACTATGCAACAGACAAATTGTATTGTGTGTGCATTTTAAAATCACCTCTAAAATTGTAGATGTCGGTATGTATGGCAGGCTTTTTATGGCACATTTGAGAGCATTGCTTAGATAAATGTCAGCTGTGTGCGGTGGAAAAATCGCATCGGGTTCACATAGCACTTATGATTTTTATCGATAGATCGACTTATCGACCACCTCTATTATGATTAATTGTGATTGTTCTTTCGCTTAAGGTGTCTCTTAAAACTTTTTATGTGTATGTGTGTTTAATTAATTTATAGATTTTGTCACCTTTGTGTAAAT

>ID_450 P_450 Promoter_453 D1_Promoter for RH09909, -250bp and +50bp [1 ]

ATTATTTCGTAAAGATCTTGTCTTAAAACTGTGTATGATTTTTTGACCCATCAAATGGCAGTCTTAACTTATTATTCTTATTCCAAATTTCGTTCTGTGCCGCCAACGACGCAGTTGCTCTCGATCTTGATGCCTTGATAAGCGGGAGAGCGCATCATTTACCACTTGTTCAAGTGCGTTCATCGGCGAAAATTGCGGCGATAAGCTGCGATCCGGTGCGCTATAAAGTGGCCAGCAGCCCGGCAAGCGTTTCAGTCCGATCTTGGCCAGCCACCGAGAGAGACAGCCCATAAAAACCAACTCCCATCACCAAACAGTAGTAACACCCATTCTACCTGGCACAGCGGATT

>ID_451 P_451 Promoter_454 D1_Promoter for RH17752, -250bp and +50bp []

AAACAAGTAACCAAATTCTGTTTCGATTTCCTGGAGTCCTCGGACCAATCAGCTGTCGAGCTTAAAACGGAGCCGAGAGCCAAAAGCTCCAAAGCCCAAAGTGCCCCCACAATAGCCGGAGAGCAATTACAGTTAACGCAAAATGATAAAACTGTCGAGCATTGCTCGCCCTCTCTCTCTCTCTCTCTCTCTCGCGGCAGAACTTTCGATTTTTCATAATAAAAAGGTTACAACTAAATCTCAGGCGGTTTAGTCGGCAGATAAATTCGGAGCGAGCGCGCCAAATACAAAAGTCCGCCACGCGTTCAGAGCCCCAATCTCAAGTCCCATAGTTCAAACAGTTCCGAGCC

>ID_453 P_453 Promoter_456 D1_Promoter for RH13154, -250bp and +50bp []

CTTTTCTTTCTTCCATAGCCGTCTCTTTCCTACGCAACTCCTATTTGCCAGATGTGCGCACGATGGATTTTGTTGTAATGCCTCTCTTCCTCATGTCTCTATAGCTATCTCTTTTCACGGCACGGAAAACAGCAACGTTGTTAGCTATCGCTGACTGCAAATGAAAAGAGACAACTAGAGCAACGCCGCCAAAATGGCTGGCCGATGTGTTAGCCGTTGCTCTTGCCAGTGTTTTTTCGCATGAGAGCTCATTAACGTTTCGCTTGGCTAAGTGCGCACCTCGTCACTGAAACCAGTTAGCAAAAGAAATTTTTGTTCAAGCTTAAACCGATACCTACGTCACTATTCGA

>ID_454 P_454 Promoter_457 D1_Promoter for RE68891, -250bp and +50bp []

TTTAATTAATTAAATTAAATTAATTATTGAAAAATTAATCATTCGACATGTCGGATGTTAGGGTGTTTTAATGTTGTGGTCTAAACATATATCATTTGACTATAAGTACATATTTTCTTTTAGTGGACTTGTCACTATTTCAAGTCGAGCTCTCCACAAAGAGAGTAAATCTGGGCTTCCGCGCTTGGGTCTCTCTGAGACTGGCGGCGGCAGCGACGTCGACGTCGCCGTTGGAGTCTAGAGCTGCGATCAGTTCATTTTGATCGGTCGTCGCAGCAGGTTCGCGAGTTCGAGCAGCGGCGCAAAGCGAGACAAAAGAGCAGCGTTCCAAGTCTTCGGCCCCCAGTCGC

>ID_455 P_455 Promoter_458 D1_Promoter for RH64511, -250bp and +50bp []

TAAACACTGTATGAAAAAATAAAATCACAATTGAAAAAGCTGATAAATTTTTTTTGTATTTTAATTTTTTATCGGCCAATAAATATGCATAATGTTTAAAGTTTTAACGTTACAAAAATTATGATTCTTAAATTTCAAAAGTAATGAATTTTAATGTATTGGGCTTCTAGGTCCACTCTTCGCTCTGTGTGGATATATAGACTGAGCCCCCTTAATCCGAAGCTGTCTATTGACTCAGATACGTCGGAGGTTTTATTTGACTCACAGTCGCCGGCATGGAACGTTGGCATAACTGCGTAGCAGTGGTGACCGGAGCCAGTTCGGGAATCGGAGCGGCAATCACCCGAAAA

>ID_456 P_456 Promoter_459 D1_Promoter for RE10568, -250bp and +50bp []

AATGGAACCACTGATGATCCACACGGTCGGCAACAAGTCCCCATACTCACGTGCTATAAATCTATAAAATTTGTAGCTGGCCGTCGTAAATAAAAGCACGGTTCGGACTAAGGGAATTAGGTGGCGTATTAGATTTGATGCGATTTCGCGTTTATGGGAGCAACACGTCTCTGCCTTATCGAATCTTATCGCAATCGGTTCGAGCCGAATCTCTCGATAGGCCAAAGTCGGAGTAACCACTAGCCAGGGTCAGTCTTGTGCAAGATGTTCCGCCTGTTGAACGCACTACTGATCGCCATCGTGCTGGTAGCCCATCCCGCTTGGTCCGCTCCGGAATGCGGCCGTACTCC

>ID_458 P_458 Promoter_461 D1_Promoter for RE03177, -250bp and +50bp [1 ]

ATAAATAGCCTAAAGCGAAGCGCAGCCGAAAGCAGCATAAGAACCGAGCCGCGAGCAAAACAAAAATAAGAAAAATATTAACAACTATAATCCCAGCCGAAGCTGCGGCTCAAGCTCGATGACGTCACAAGCGCGACTTCGGCCGAGCGGCGAGCGGTAAGCAGCGCAGTCGACGTCGACGCCGGCTATATATCCAATAGCGCGCCAAAATAAGAAGTCGAAATTCATTCCTTTCGAGCCCACAACGTTCAAATTCGTTTGCTACGCTCACGAGAGCGGACGTGCAGTGCGGAAAAATCCGACAAAATCAGGCAAAGTCCCCAGATCCAAAACTATATACGGAATAAGCA

>ID_459 P_459 Promoter_462 D1_Promoter for RE25361, -250bp and +50bp [5 ]

CCATAACAAAAAAACATAAGTTTGTGCAACTTAAAGGTAATTAAAATGGCTGTGCGCGTTTTGATTAAATCATAACTAAGTATACATAGAGACAAGTGCAGAAGTTTGATGGCTTAACCCTTTTGAAACTTGTTTAGCCACCACCACTGGGCCGCAGTTGTTGTTGTTGCCCCGATCATATGGTGGATTTGTTGCTGCTCTTCTTTGGCCGCTGCTTCCGTTTCGCCTTTGCTTCTGCTTCTCAGGCGCAGTTTCAGTCTCTCTTTTGCCACCGCAACGCTTACAACGTTAACATTGCGCGCGCGTCTTTGCATTTTCTTGTTTGCTGCTTCGTCGTCCGTCGTCCTTCC

>ID_460 P_460 Promoter_463 D1_Promoter for RE69168, -250bp and +50bp []

GTGTTTAGTATATTTTCTAAGCACCTAAGCCCCAAGTTTTTAATCAATTTTGCAGTGTCCCACATTTCAATGCCCCATTTTTGTTAACAAGTAAATGTTATGCTCTTACCAACAGCTGAGCAGCGAGTTGGCCTCTTGTAGCTTCGATCCGGCCAAAGAAAGCTGCCTGGCCGAAAGCTCCGTCTTAACCACGTTAACGTTAACTACAACGAGCATTGGCCTCGTCATTTGGATGGTATGCTATACCGCTGAGTTGAGCGCTGGGCGTCGATCGTCGAACGTCGTACCGCTGCTACGTACCGCTACCGACGCTTGTTTTTTTTTTTGGGGGGCTGCTCTTCCTAACGCAA

>ID_461 P_461 Promoter_464 D1_Promoter for RE26616, -250bp and +50bp [2 ]

TGATTGACAGAATATATTAAAAATTATTTTTATTAACATGAGGTTGTACCCAAACTTCGATGTTACTGATATTAAAATTATAAAAGCTCTAGGGTAAAATGTATTAATTTTAATTTAAATATTAACATTTATATTTTTATAAACATACATATTTAATTCCTGTTTAATTTTGTAAACATGATAAATAATAAATAACGATATTTTCTATCGATAGAGAAAGTGCATCTCAATGGCTTTCGATAGCTGTCGTGCAGTTCGATGTATGTTTTGGTTTTTAGTGCAACAGCTGAGAGCTCTTTTTTTGTGAAATTGTGCATATTTTTAGTTTGTTTTGTTTCGTTTAGCGTTTC

>ID_464 P_464 Promoter_467 D1_Promoter for RE67013, -250bp and +50bp []

GCTGCGAGCTGCGGCTCCATTCATTGTGGGTAAGCGAACACCGGGCCGTAAATACTTCCACTTGCCTGTCGATACGATAACATTGCGATTAGGCTTATCACCACTACTCGCTCGTTCTAACGCCTTTCGCCATCCTTCGACGTCGAGCAAGCGCGCAGTCGCAGCGGGCAGAGCAGTGCGGCAAAGCGACAAAGCGGCGAGCGGCGAAACCGTCAACGGCTTCTTAGCTAAGTTCGTTCACAGTCGACTTCAGTCTGAGGCTGTTGATCGCCGCTTGCGGACGTGGTCGCTCAATCCTGCTCGATTCATAACCGATTCAAAACCGATCCCATCCGAAACGACGTGCGTTC

>ID_467 P_467 Promoter_470 D1_Promoter for RE10981, -250bp and +50bp []

TCTCAGTAATTCTTTGAGGGAAAGACATTGAATGCAGCACTGTTTATCGAGCTAAATGATTGAAAAGAAATGCCAACAATTTTCGCTGCACTTATCAAAGGCGGGCCAAGATCGGGAACTCTCACCTGGACTCCATCTCTCTCTCTCTCTCTTGTTCCCTCTTTCTCTCGGTGGCCCACTCACACAGGCACGCAAGTGAATGTGAGTATCTGTGTGTCTGTATCTTAGACTCCCAATGCCAAGAAAGATTCAGTTCGTTTCTCAGCGCCGAACGCGACGGTTCAGTTTTATTGGTTTCTTCGGAAGAAGCGGATCGAATTCGAATTCCTTGCCCGCGATTTTTAATAATT

>ID_470 P_470 Promoter_473 D1_Promoter for RH53260, -250bp and +50bp [1 ]

CTAGGTAGTGGTGTCACAGCGTCGATAACTAACTTAGTTTCTCACCGAGCTGCAACAAGTTTAATTACAATCGATAACGAAGATTGGAGCTTTTAGTGAGTAAAATTTGACCCATTTCTGTTGTTTGAACGTTTATTACGCATTACGTATTTACTTAAGCGATTTTGTGAATTTTTTGTGTGTAGTTATGTGCACGAGAGATGGAATTTTTGACCGAAAGCTGCTATCGATAGTGACATTCCTTCGATTTATTTTAGCAACCATCGATAGCTAAGCAAGCGAAACAAAAAATTATTTTCAACTTGAAAGTTTTGCGACAAGTGCGTGCAGTTGGCATCGAAATAGTTAAC

>ID_471 P_471 Promoter_474 D1_Promoter for RH61862, -250bp and +50bp []

ATGCATTGTATGGGGGTTTCTTTTGCGCAGAGCAGTTGTAATATGGGATGGTATCGTTTTGTGGCGGTGCAGCCACAGCCATGACATCCCATCCCTTCCCCGAGTGAACTGGGGATTGTGGGGTAACGAGTATCGATTGCGCATGCCTGGGATGCGAGGTGGGATGTGCGAGATGCTGCCGAAGGCCTGAGATGACAACAATTTCAGCAGCGGACTCAGAATCGCCAGGCCGTAATCCCTACCAGCTTTTCAGTGTGTTTCTGGAGCAGTTCGCGTGCGGCGGTGTTTTGTGGAGTGTTGAGTGGCGAAAGTGCTGGCTTCTTTGAAGGCTTACAAACAATTTGTTTCCG

>ID_472 P_472 Promoter_475 D1_Promoter for RE45038, -250bp and +50bp []

GATTAAAACTGATTAAACTATTTTCAATGAATTCCGGATTTATCAGTTCGCAGTTTCGATGATACTCTTCGAATAATTACAATTTTAGCTACGCTTAGTAAGTTTTCTAAATTTATTATTACGTCGTAAAATTTTGGTTCTTTATCAAATCGATAATACCTAGAGAAGCTTATCTGTTTAATTAAGAAAAAGACACTTGCCTGGCTGATTAGGTGGTGCTATAAAAGCTAGTCCCGCAGATGACCCAGCCAACAGTCTTCAAGATGAGGTCTTCAGTACCGGTTTGTCTAATCCTGCTCGCTCTGGCGTTGGGATCGGCGTGGGCTAATGATAATCCCTGCCAGGATGTG

>ID_474 P_474 Promoter_477 D1_Promoter for RH25931, -250bp and +50bp [2 ]

GTCGAGTGCCAATAGTTAGTAGTCAGTAGCCGGCATTAATTGAATTGCGTAGAAAACCCAGACAGGTTCTCGCTTCACGTGAATTGCACTAAATCGCATGAACAACAGACAAACAAACGAATCTCACTGATTGACGAGCGAAGAGATAAAGCCGATCTTTTAACCTGTGTAATCCCCCAGGTTGTCTTGGAATCGCCCAGATCTCGGCTCGATCGATTCACTATAAAAGAGCTCCGGCACCTTAGTGGGGCATCAGTAACTCAGCATCAGTACTACCAAGCCAACCAACAACCACCAAACCTCAGAAATCAACATGAAGTTCTTCCAAGCCGCCGCCCTTCTCTTGGCCA

>ID_475 P_475 Promoter_478 D1_Promoter for RH34187, -250bp and +50bp []

TGTTTATTTAATACTTAGCACACTTTTGGGGGACCTATACAAGTTGACCAATGACCAATGTGTCTCGGACTTTAAGATAAGAGGCAAGATCTTGCACTGCTTTATTCTTTCGGTTCATAGTAAACAATTCAAACGGACGCGCCAAAATACAAAAACGTCCTTATCGATAACTCGGAAAGCAATTGGTTTTGATAATTAATCAAATTGATGTGTGCGATAGTTTCCGCTCACCGACCATCGCCCACCGCTAGCTGTCATTCGGCGACGCGACGAACTGAAATCAACGTGCTTATTTTCCGCTCGGTAATTTGCCAGAAAAAAACTAATCGAAATGGGCGTACAAGTAGTTC

>ID_477 P_477 Promoter_480 D1_Promoter for RH52894, -250bp and +50bp [2 ]

GTAGAGAGAACAGCTTGAAGCGAGAGCAGAGTTTATCTTGCTCTCGCTCTCGTACAGCCTCTCTCCACCACGCCCCCCCCCCCTACCAACCACCCCATCCACCTGTTTATCTAAAAATTTAATTTGATTTTTGTTCTTGCTCGAATTGTGTATTATTTCTTTTTTTTGGTTTTCGGTGTCGCGACTGCGCTGCCGACGTAGCCGCCGACTGAGAGTCAAGCTTAAAAGCGCAGCTTGCAAATGGACGGAGCATCATTTATTGTCGGCCAGCGACGGCGATCGTTTTATGGCGTCAACCTGTTGATCACCTCCCCCACAACCCCCTTTCATCAGTGTCTTTTTTATTGTAG

>ID_479 P_479 Promoter_482 D1_Promoter for RH42401, -250bp and +50bp []

GCTCTGCTGGAACCGAGGGCCAAGCTAACGCCTGCATAACACCTGATATTGGAAACTTTGTAGATAAGGGCCTGTATTTCCTTGTATCTATTTTAAGTGTTGTATGTATTGTATTTGTATTTGTGCTCGGATTAAGCTGAATGATAAACAAACGATTTTATTAAACCGTCACCTTGGTTATCCTCACCCTGTCAGCGCCCTTGGGCGAATGGCAAACAGCTGACGCCATATCCGCGGACGCGAATGGCACATTGCTAGTTGTGTTTTCTTGCTTTGCGTTTGCTGTTTATCAAACGCCTTTTGGCTAATGGTCCGCAGTCCGGTGGCGTTATCACGGAATTGACGAGGCC

>ID_480 P_480 Promoter_483 D1_Promoter for RE15012, -250bp and +50bp []

CCACTCCTGAATCACCGATCAGCAACAGTTTAAAGAGCAAATCGTAGGTTTTCTTTGCCATTTCCAAAACGCAACGGAACGCTCGCAGAACAAAGCACAAACACACGCGACACTCACACGCACGCAAACACACACACACACAAACGCACGGGCGCAGGTGACAAGAGATATTATTATTATTGAAGCGCCTGCCCTTTTTCGCTCTTTCTCTCGGTTATGGCCGCCTAAGCAATGATCCTGTATCTCGAGCTAGCTCTTTTTTCTCCGCGTGTCTTAAATGCGCCGAATTAATTAATATTTTCGCTTGAAATTGTTGCTATTAGAACTAGAGCTGGAACATGAATCGATAT

>ID_484 P_484 Promoter_487 D1_Promoter for RH44411, -250bp and +50bp [1 ]

CAAATTCGTTCTTAATATGTTAAGCATAGTATTTTATTACGATTTTAAAATAAGCTGAATTCACCCACAACTGCGCGTTTTCTTCAGTGTTCAAAAAGTATCTGGAGAGCGCATCGGATCTACTATCTGACTCGCTCGCACTCCCACAGACAGCCGATGCGCGGGCGAACAAGTGTCGAGGTGCCAGCGGTCGTCCCGCTCTAATCATCGTACCGTGTACCTCTTTCTCGGAAACGTCGCTCGCGTCGTTCAATCGTTTTACAAACTTCGCTCGGAACGGACGTGTGCGCGCTCTGAAAGGAAAAAGTGAAAAAGTGTGTGACAAAGTGCAAATAAGCCACAACGCGCAT

>ID_487 P_487 Promoter_490 D1_Promoter for RE24713, -250bp and +50bp []

TAACTACGAAAAGTCCGTGCGTAGATTTTCACGAAAACGCCCCGCCCGCGCCGCACTGAAGTTGTGTTAGTTAGTGGTGCGACAACGCACCGATGCAGCGCCTCGATTATCGTTCGATAAGCATCGATTTTCCGATTGACTTGGTTCGAATCAACCGTTTAGCCAGCGCAGCAACCGCAGTTAGCGGGCACAAGCACCGAAGCGCGTACGAGCGTGGTGAGTTCGTACGGTGGTGAACATCTGTGTTTTCCAGTATTTGCAGCACCCAGCCCTGGTAACCGGCGCCTTGTCGCAAAGCGAAGCAATCTTGCCGCGTTTTATAGTCAATAACTGCGAATCAGCAAGAACTG

>ID_488 P_488 Promoter_491 D1_Promoter for RE66405, -250bp and +50bp []

TTTTTTTTTAATAAAAAACAAGTTGTTCGATAATATCTACTTCTTAGTATGAGTGGTTTTTATATAAAATTAAACTAAAAAGCCAAACTTTTCTATTGGGAACATCTAAAAAGTCGATGAAATTATTGAACAGCCGCCAAATTCAAACCAATTAGCCAAGACACCAACCATAACAAGATGCATCGTTATCGACCGCGCATTTTGTTAATCGAAGCTTTCAACGCCGCAACATCGGGCAACGCTTGCCTTGCCTTCTCTTCCTCTTGCTTTTGCGCCGAATATTTTCTTTAAACTGTTTTCGTATTAAATTGTTTGTTTAAAATGAATCTGGAGGACATTTCGATGATTAT

>ID_489 P_489 Promoter_492 D1_Promoter for RH38235, -250bp and +50bp []

ACTGACCACAAAATAGCCCGTCATGTCTGAGTTGTTGCCAAATTCTCGCCGAGTTGTTTGAACAGGTGCTGGCCAAGTGCTAATAGGTCGTCGAAACAAAGTATCCGAAAATCTTGCATCATCATCATTGCAGCGTCCAGAGTTCCCTGAGTTCCAAAGATAATGGCATATAGTATGCATGGGAGGAGGCAGCAGCCAGAACCCTGCGCCCAGCTCCTATATAAATAGACCCACCATACTCGATTGCGACACATTTCCAGTTTAGCCAGGCATTACAAGCACCAGAAATTTCCCACTCCCCGCGGCAGAATTCAAGTTTAAGCCCGGCTCAAGCCACTCGACCACTCAGT

>ID_494 P_494 Promoter_497 D1_Promoter for RH50269, -250bp and +50bp []

CAGTTTAATTAATCTGAACATTTTTTTTGAAAACTTTAATGGATATTTCCAAAGAATTCGGAAATATCTCTTTCGCAATCTCTTAACTTAAGCGTTTTCTGTGCAATCTTTGGGAATAACACAATTTATGTGCATATGCATGTACATATGTACATGTACATATGTACTTCGAATACATCTTTGTCGGCTGATAAGCTTTTAAGCTGCTCAGCAAAGAAATATAATGATACCCACTGCCAGCCGTTCGTTCACTCAGTTTTCTGAAGCAAACATGAAGCATTGTTGGGCAATCGGAACTCTACTCTTAGCAAGTGTCCTTATGGGCAATGCCTGTCGACATACTTGCGTTA

>ID_495 P_495 Promoter_498 D1_Promoter for RE47829, -250bp and +50bp []

TGAATATGGGCCACTGGCTAACAAAACCAGCAGCTGTTAATCAGTCTATTATGGTGTAAGGATGTCTTCCCCGCCGTGCCCACTGCGATTTCCGCCTCGGGCTGGCACACTCAGCGTATAAATAATGCCAAGCTCGTAACGTATACGCAACGTTTGCTTGGAGAGATCGCTAGCTGGGTTTCTTTCCCAATTACAGATAAGCTCAATGCCCCGGACTTAGATTGATAAGCCTGCGGCTCGACTGGCAGTCTGGACTCAGTTCTGCTCGAGCTTTCGTCAGAGTCGGAAGCAAATCCCAAGTCGCATTCGAAAGCACTCCGATAACCTTATCAAGGCGAAACGGAAGCCAG

>ID_497 P_497 Promoter_500 D1_Promoter for RE35158, -250bp and +50bp []

CGCCAAATGGAAATCTAAAAAGCCAATACAGATCTTATTGCTGTTTTTATCGATAATATAAATGGTATTTTTATTGATTGTGTCTTTTTACATTTTAGCTAATATTAAACTAAATCATGATTGGAACATAAATAATAAATTACAAGGTAGCTTAGGAGACTTAGAGAATGCTTTTCTGTTATCGATATCGGTATCGATACATCGACCGAGCAGCGCGCCGCAGCGATCGTAATTTCTTTCCCGATCCCGCACAGTCAAAAAAAAAGGCTCCGAGTGAACGGACGTGCGCGCGCGCAAACTGAAAGTGTAAGCACAAACAGAGCGAAGCAAAGCAAGGCAAAAGCAAAAGC

>ID_500 P_500 Promoter_503 D1_Promoter for RE16118, -250bp and +50bp []

ATGTTTTTTGCTGCCTAATTTAAAATAATTTTTTAAAATCCTTTAGAGCCTCTTGGAATTCGTGCTTTTTCTTTCTGTGCCCCCTTAAGAGCGTGAGACAAGAGCGAGTTTCTTCTACTCTTACGTATTTTCATAAATCAACCGCGATCGCTGGCATATCCGCCCAAATGAAGAGAAGCTACTCTGTATTTTTGTGCTCTTTGTGCCCGCCTCTTCAAGTCGCTTCACGTCGAGGCAAGTCAGCAGTTCAGTCACATTTAGACATCCGCGCGGTAAACCCGCTTTCGGCGGTATAACGAGATTTTTTATTTCGAATTCGACAGAAACAAATAAATGTAAAAAGGAGAAAT

>ID_501 P_501 Promoter_504 D1_Promoter for RH64195, -250bp and +50bp []

ATTATGGCAAAATAAAAATTCTTTAATTAACAGAGCGCAACTTAGCTTGTTAATATAGATTCTTGTTCATGTTTTTGTATTAAATGTAAAAATATTTATTAAAAATTGCTTTAAATGTTTTTTATCGATAGGCATAGCTATACCGATATTAGTCGATAGATATGCAGCTGGTCTTAGTTTATCGATAGTACTATCGAAATTGTCATCGCCAATTGGATTCTGGTATATATTCGCGGTCGCCGGACGGTCACACTTATTGCACTTACTCCAGTTGGAAAGGCAACAGTGTTCGCGTCGCGGAAGAAACAAGAAAGAAAAACAGCGAGAAGCACTGCAAATGCATTGGCTGC

>ID_505 P_505 Promoter_508 D1_Promoter for RH54796, -250bp and +50bp []

CACTTATACTAACACGTACCCCGCTAAGTAGGCAACACACTTTTCGGCCACTTCCGTTGCACAAACGACAGTCGACAAGCGGATATCCATATCCCCCTGCCATTGTGTGCGTGCGCAGCCGGCATTCCTGTCCACACACACACACACACACACACACCTAAACGAAGCACCCTTCGTGGACACATGGATGGCCGTTCGGGCGTCGATTCGCTTCCGTTTTGATTTATAAGCGACCGCCGCGCCGGGCTGCGTATCATTAGCAGTTGATCAACAGGTTTGCGCACAGCCAACAGCCAGGACAGGACATCCAGTTGCCACGCTCCTCGAAATCTGTTTCACACCGGTCAAGT

>ID_508 P_508 Promoter_511 D1_Promoter for RH49114, -250bp and +50bp []

TCCGCACCTGATTCGCTTTCCCGATTCGCCCTGACCCGATTCCATGACCTCGCCCACTTCTAGATATACAACTCTTAGCAATCTATTGCATCTTTATTGAGCCGCTTAATAAACAAGACATCATGGCATTCCACAATCCCACTTTTGTTCTCAATTATTGTGGCTCGGCCTCCGGGCGAGAATCTCTCGCTGATTAGGCTTATCAGCCGCCCGGGAGAAGCTTTAAATGCCAGCTCCACGGCCGGAGCACCTCAGTTCGAGCAGATCCGGTGTTGAGTGCGAAAGTGAAACCGATTCCCGAGGAGATGGGCCTGCTGCTGCTTCTGGTGCTGATAGCGGGCATTGGCGTA

>ID_509 P_509 Promoter_512 D1_Promoter for RE26394, -250bp and +50bp []

GAACAACACAACAGCAACCGCCGTGAACAGATGTTTGCCGCGAGGATGGCCACACTGGTAACGTCGGTAGTGTTGTTAATAATAATAATTTATAAGTAATGACTAAATCTAAATTTTAAATTTGAAAATAATTCTTTAAATAGGTTCAATATTCAAAAATAACTTGTTTTGGAAATGCAAAAGAAACGTTGTTAAGCACTAAATGGTTACGGTCTCATTAAGCTTTACTTTCTTCCCCACACTGCCCGCAGTTATCGATAGCAGCACTATCGATTCCAAGTGATATTAATTAATCACTTCTGCTGTTTACTTTCTTAATTAAAACTCTGTTAATTAAATTATCAACAACG

>ID_512 P_512 Promoter_515 D1_Promoter for RH28801, -250bp and +50bp [4 ]

TCAAGTCCCAGCGACTAATTTCGGGGAAACGCATCTGGAACTGCATGTATGAGTTGGCTGAAAGTGTTCGTCAGAACGATATTTAAACCTTTCGGGATAAGAGGAAACTCGATGAAATCGTCTACCAAATGTATTCGTGCGAAAAAAGACCGAAGATCGCTGAGAACGCACTGAGAGATTGGGAGACGCAGCAGAGGAGGAGAGACCTGAAATCGAAATCGCTTGGAGAACGTTTCCCCAAACCGAGTAAGATCAGTTGAAGGTGGCGCGTTGCGCTCGACGGTTGTCGCTATTCTGGTTCTCGCTCAAGTTATCCGAACTCTTGGAACAGCTGGGTAGTCTGGTCTGGC

>ID_513 P_513 Promoter_516 D1_Promoter for RE38555, -250bp and +50bp [2 ]

AGTTATCGCTGCGAGCACGACGAATATTTAAAATGGTTCACACTGCAGCCGCATCTGCAGAAATATAAGCCCTGTATACCGCAAAATGGCAAGGCATTTTATTTTTTAATCTTTTTACCAACAAATTTAATCATAAAGGTGAAGACCGAATGGGTTTGCCGTACGTTGAAAGGGGATGAGCCGCACTGCTTTGAATATCGGATGTGAGTCCGCTGAACTATCGATAAGGCAATCGTCAGCCAACCCTGCTCCAATTTCGTCTTCTCATCCCTTTTGCCATGACGGAAAACACGAATTGATGTGGATTTCTACTAATTTATAACATAAAAATACGTCAAAAGTTGAACAAT

>ID_514 P_514 Promoter_517 D1_Promoter for RH68992, -250bp and +50bp []

CATTTTTTGATAATGAATGTTGCCTACTCCGCAATTTCGATTTTAAGTACGCATTAAATACTAGTCATAGATTGAATTTAATAGCTCGTTTAGTATATCAATTAAGCTTGTGGTAAGCTGTGTTGCTGTTAAGCTGGAATTTCCCGCACTCACCTCTGTCTAAAAGTATACATATCACCTTTAAACCAACCACTTAGAACTGCTGATAACCCAGGGCAAGGCTATAAAAGCCCAGCTCAAGAGCCGACTGACATTCAGTCAACTGTAGCCAGAGCTAATCGAAAGAAAAATGCAATCCATTCTCCGACTGCTGGTGCTGTTTGGATTAATCTATACCGCGATCGGTGCAT

>ID_520 P_520 Promoter_523 D1_Promoter for RE24635, -250bp and +50bp []

AGCACTATCTGACTCTTTCACTCGCTATCCGTCCCACTCTGGAGCGCTGTCCAGATCGGGGGAATCTATCTGTGATCTCCGATAATATTGAAATTATTTTTCGGCAGTGACTATTGTTTGCATGCTAATTTTTATCCGAAAGCGGAGGCTAGATTAGATTATTGTTTTTTTTTTTTATTTTTTTTTGTTTGTTTGCGTCTGCCTGCCGTCTGGCCACTATAAAAACCAGGCCCTGGGGCATTGGCCATTAAGTTTGTTCAGTCACTCCGATCAAGCAGATCACCATGAAGGTCTTTGTCGTTCTCGCCGTAATCCTGGCCGTGTCCAGTGCCGCCCCGCAGTTTAATAAT

>ID_521 P_521 Promoter_524 D1_Promoter for RH23805, -250bp and +50bp [2 ]

GAAATCCGCTTCCGCCCTGCTCGCACTTTTATCACAGTGTTTTTGCCGGCCATATTCTATACAAAGGTCAGCATGACCAGCATTTGGCCATTCCTCCAGTGAAGTAATGCTGCAAATGAGGCGAAAAGAGTGTGTGCCCAATGGTGACGGCGCCATCGAGCATTCCCGATATGTTACAATCCAATTAGCCGGATGCGCCAGTTGGCCAACTAGAGTACTTGGCCCGTCGCTGATTGATTTGCAGTTTGTCAGACGTGCGTGGAACGCGCAGCGGTTCGGTCGTGTTTTTTCCAGCCCTGTTTTTCCCCTGAAAAGCGAACGGAATTTCAAGCAAGGATTTCCACGCGAGT

>ID_522 P_522 Promoter_525 D1_Promoter for RE38986, -250bp and +50bp []

CGGGCAAGAAAACGGTAAACAATAATTGTACTTTTCTAATTATGAAATATAAATAGAAACAAACAATTTATTGAATTTTTGTGCACTTCTTTTAATATAAGAAACGTAAGTTCGAAACCCTTCGAACTCGAACTAAATCGGTTGCATCGAACCAAGAGAGTGACCGTTCGCGAGCTCGGCTGTGATGCTAAAAATGCCAGATGGAGCAAGCAACATATACGAATATATACGAACGCATTTATCGCCGCGCGGTCAGTCGTCGAAATGTTCGAGATCCGGTCGTTTTGAAAACCAGCTCGAGCGGTCTATTCGCGTGAAATTGAAACTGAAAAAGCCGGTAAAACTGAAGC

>ID_523 P_523 Promoter_526 D1_Promoter for RH55715, -250bp and +50bp []

GTGTGTATGTAGCAGGCAGAAATTCACAATTTGGTTTCAAAAGCTAAATATAAATATAAGTATGCTTAGGTAATGTAAAATTCAGAGTACGAATCACCGTTATAGTAAAAAACATTAAAGATAAATAAATCATATCAAGAACAGTAAAAGTTATAAAAGTTTATCATTTGGGCACACTGCCATCCGGCCCGATAGGCAGCAAAGCCGCCTGATATCGACGCGCAATAGGGTCCGATAACTTCTACAATTCATTGTTATCGAGGTATTGTAGAAATACAGCTGATTGAAAGGCGAATAGAGTTTTGAAAAGCGCACAGAGATTGAACACAGTCGAATGGCGGACAGTGAAG

>ID_525 P_525 Promoter_528 D1_Promoter for RH55307, -250bp and +50bp [2 ]

TAAAAATTATACAAAAATAAATAAATATTTTTAAATTTTGTTTTATATTTGAAAATATATATATGATTTGAAAATATGATATATTTTGCATGACTTTTATTTCATTAGTGTCTAGCTTATCCTTTATCTCTTATTTTTCCCATCGATCCGACAACGCTCGCCACTCTCGCATGTCATTTGCCAGATGTGGCAGCCCGCAGGCACAAATTTTGAAAGTGGTGCCCAGAATGCGATGAGGACAAGCAAGGCTCATTCGAAATTTGTGCGTGCAACGCGAAGGACGTGCCGCTCCGTAGCTCGCTGTCCCGCTCTCGCTCTCCCCGCTCTCTCGCTCTGTCGCACGCGTTCGC

>ID_526 P_526 Promoter_529 D1_Promoter for RH10437, -250bp and +50bp [6 ]

ACGAAATAGCCGCACCGTAAATACGCCTTCCTGACATGCGCATTAAGCGTGAGAGCTGATGGATCGCTCCACCTCCCCGCAGGAACCTTAAGAACTCGATCTCGACTCTAATTAAACTAAATACGGCACAAGAGTTTATCTTATCGGCGGATGCACCCTGTGCCGACCGGCCGTACCAGCAATGTTAACTATAGGGCAAACCGGGAGCCGAGCACTCAACTTTAAAAAACGAGACCCCATCTGGTTTCAGCAGACAGTCGTGTTTGCAACGATCTAGCGAGCGGTAGGTAGCCTCAGCGCGACTAGAATACTTGGTGGGCTAGAGAAAGGAATCCAAACCTGAATCGGAA

>ID_527 P_527 Promoter_530 D1_Promoter for RE46961, -250bp and +50bp [1 ]

GTGACGCCAAAAATGTATATATATACAAATATATATATATATATATATATATATATATATATATATATATAAGTGCATAAATTAAAACATACAAAGAGAGCGAACTGTGAATTTGTTTTGGTTTGAAATTAATAGGCCACTGTTTCGCTTCGTTGAACTTGCTTTGTGTGGGCAATGAGTAAATTTTGGGCGATTCTTCTAATCGTCATTCCCCGATAATTATATATGAAAAAGTTCTTATGTTAGATTAGTTGTCGTTCGTCAGCGAATCGCTGCGGTTGCCTTGCAAATGGGTGGAATATTTTCTGTAACTGGTCTTGGCATTCTGCTAATTGGCCTGATATGGCTGT

>ID_528 P_528 Promoter_531 D1_Promoter for RE70614, -250bp and +50bp [0 ]

TGTAAAAATCTGCTATCGAGACAGCGTTGGTATCGATAGTAGTTAGTTTGCAACGAGAGGGTTTGGTGCATTGATTACTGTATATTTCAAAGAATATTCGAATTGTTTTCTTTAAAGACAACAATTTTATTCGCCTTTGCGATTGGTAAAATAGTTGTTTACTTTTAATCGATAGACAAAAACAGTTATTGGGCTGCCATCTGGGGCGTCTATGCGAAGACCTCTTTGCGTATTTCAGTTATCGATTACTGTGACAGTGCCTAGACCTCCTAAACTCAACTTTATTGCCCGGCCGCGAATTGTCTTGATTGCAGATTTTCCGATTTTAAACAAAATTTACTTGGCATTAT

>ID_530 P_530 Promoter_533 D1_Promoter for RH13513, -250bp and +50bp []

GCTTTGAAAATAACGTATTTGAGGCAATAGGGGGCATTTAAATATTTTCTACATAAGTCGGATTTCTACATATATGTGCCTAATTTTACTATGGTATTTAATTATCGATCACTGGTAGTCACACAGCTGATATCAAGCTCCCCCACCATTGTTGGGATCTCTGCTAAGATAAAATTCAACCTGTTATCGATAACCGACATTTTTGATTGTGGACTGCCACCGCTATCGAAATGCACCATATTGCTTTGTTATTGTATTTTCCGCACGGCCCTCGAACAAAGTAAACAAATGTATAAATGCATGTGTTTTTGGCGAGCTTGTAACTGTTAGTGAAGCGACGCGCGGAGCGA

>ID_532 P_532 Promoter_535 D1_Promoter for RE70803, -250bp and +50bp []

ATGATGTACTTTCTCAAATAAATGCGTCGTTACATTAACGTCAAAAAGAGGAAAAATTAAATAAATATTTACAACGACAGGTTATTCTTTTCATTATTTTTGCAACACATTTCTTGTAGGTTAGCTTAATATGTATATTTTGATATAATAAAAAATATATATAGAAATATCGAAAACATTGGAAAAACAAGTTTAAAATTTCGAGCATTAAAATCCATTGCTAACCCTCTCCTCCCTAGCGTCATAAGCAATCGGTTATCGAGGTTATCGATAACGCCCGCATCGATACAATTCCGCCGGCTTGTCCGATTCGTGTTGTTGTGATTCGGGATATATACAGGATATAGGGC

>ID_533 P_533 Promoter_536 D1_Promoter for LD04388, -250bp and +50bp []

TATTTAAAACTGAAACTTTATTCCACGTGTTTAATAAAATTTTATATTAAGATCGCGATATACCGTTATTAGAGAGAACTACATTTGTTCTTGGAAATTAAATGATGTGCCTTTGTTTTTTATTTAATCATTTAATCAACAATAATCTATATACTTGGATCTGAACAAAAGCCGTTACTCCCAGAAACCGTGACTTTTCACCGATATATCGCCTACTCGTGCTTCACAGCACTGTTTTTCAGCACTACCCCATTTTAGAACATCCTCACGCACGCACGCACAGAATTTTCTTAGGTGATTAAAAGTGAGTGAAAATTGAGAATTTCCACACGCGTCGCACGCTATTGGTC

>ID_534 P_534 Promoter_537 D1_Promoter for RE03306, -250bp and +50bp []

CAAATAAACTCAAAATTCTTAGAACTTAAAGAGGCTAGCTTAAGGAAACTATTGCAATACAATGCAATAATACAAATGAATCCACAAAAACGTCAGCTCATAACAAATTTAACTTGTCACTGCATTGAGTGCTGAAAGCTTTTTGTTGATATTTTTCTGTGTTCGAATAAATACCAAAATATATGACAGAATATTTCGTACGGTGCATTTGCGTTGCATTTTTCGGCGTTCGCAACGCGGTCACCCTGCATTTAGTGTTTTGTGAACTGTTAAAGAATCCGATACGAATTTAATTGCTGGGAATAACAATGATGCAGATGAGCGAGTACAAGGTGCTCCCGCAGAATGCA

>ID_535 P_535 Promoter_538 D1_Promoter for RE04282, -250bp and +50bp []

AACTTACCGCCACGCACACCAACACACAGTGCGTATGCGCAATGTCACGTCTGCGGTGAAGAAGAGGGTTCCAGTTGTAAATGTAATGGTGTGTAGTTGTAATTGAGTTGTTACTGTACCCACATACGTAGTAGCCACCTTTCTTCGGGCTGCTCTGTGTGTGCGTGACTCTTTGGTTTCTCTGCCTTTTTGCATTCTTAATAAATCAACGCGAAGAATATATAAATTGGGCGGCTCGGTGGACGGTGGCAGTTAGTATTTTCCAGCCAACGGTGCCTCACAGGTGTGCAAAAGAGCCCGCAGTGCGAGTGCAAGCCTTGCGTCTTACCCCAATTATATAGCCGGGATAT

>ID_536 P_536 Promoter_539 D1_Promoter for RE02819, -250bp and +50bp []

ATTGAGTGTCGTGGACAATTATAGCCGCATCTCAAGTCCCCAATTGGCCAAAAAGCCGCTCGAGCATCTCGCCGGAGCGCCAAGAGATCGGAGAGAGAGTAAAACCAGCAGCAGCAGCAGCAATACATATCGCACCGCAGCAGCAACAGCGCTGCCGGCAGCAGCAACAGCAGTAGGCAGCAGCAGCAGCAGCAGCAGCAGCAGCTAGCAGCAGCACATACGAATCCTCAACGTTAGCATTTCCACTCCCAGTCGCAAAGAGACCGCCCTGCAATCTAGACGTGATCAGCAGCCCATCAATAAAAAAAAATACAATAGAGATATACTCGCGGTGTTCGACAAACAAACAG

>ID_537 P_537 Promoter_540 D1_Promoter for RH57730, -250bp and +50bp []

TTATTTATAACAAATATATACAATATATATGTTGATTCGAAGAAATTGCTGTTTGCATAAAAAAATACAAAATACGCCTATATATGATATCACAGGATCAAGAAGTAGGTGGATCTGTTATCCTGGTGTCGCTGTGTAACTAAATAGCGTTGGGAAAACCCACTCCTCTTCACTTCTCTCTTACGTGCTCTTCCGAGCTTAGATTCCCAGAAAGCAGGTTCTATAAATGCTTCGATTTCAACTCTACTTGATCAGTCGACATCGCGAGTCTGTAGCTACGAGATTGTTTCTAATCGGTGTTCTCTTTGCTTTAAACTGCACTAGCACATAGGTATAAATGGTAGCAGTAT

>ID_539 P_539 Promoter_543 D1_Promoter for RE50435, -250bp and +50bp []

TTCATAAATTTATTTGAGAGGAGTAAAACAAGAAGAATCCACCTAAACATTACAGACTTAAGTTCTAAATATCCCAAACGTTGTTTAATTTCTATTTATATTTGTAACGATTAGTTAGTTATTAGTAATAATGAAAGAACTAGTGATAAAATCCATTTGTAATCACTGCTAGAATTGGCTTGAAAATAGCTAGACTGACGTTAACAATACTGTTACCAGTTGGCATTTCCTGATCCGGCAACTCCACACGACGCCATTTCAAAACAAATGCGCGGCAACGATTTTATTTTACCCCGCAACATTTAGTGAGAAGTCGCAAAATAAGTCAGATTATTTGATTAAAATGGGAG

>ID_540 P_540 Promoter_544 D1_Promoter for RH71894, -250bp and +50bp [3 ]

AACACCAACCCTACCGGCGACCACCTTCATCCACCAAGTTGACACACATTTCGTGTGACTGGTATAATAAGTACACATGCCCGGCTAAATATCCAAGCGCATTGCTCCACTAACAACATATTTCTATTTGGTCACCTGTCCCACCATTCCCACCCAGCCAGAGCGACAAGCAGAAATCGCAAATCAATAAAATGGAAACCATAAAATTGCCAGAAGCGGCGCCACGCCAATTGGCACCACAAAGTTTGTGTCATTTGCGGAGCAGCAGTTGGCGAGGAAGCACCAGGCGGAGAGCACTTCCAGGACCAACTCCAATCAAATGCAGTGCTGTGCAGGCTGACCGACCAACT

>ID_541 P_541 Promoter_545 D1_Promoter for RH43215, -250bp and +50bp [5 ]

TGGTCGTTCATTAGTAGTAAAAATGGGATGATTTTATTTTATTCTGCTTTACTGTTATTTCTGTTTTCTGTTTTTATATACTGGCCTCTTGAATATATAACATTCTGATAACATTACAAACGCTCCACTAACATGTCTATATGTCTTGGAAGGGAATTCAAACAACCAATCTCTCTCTTTTTCTTTTTCTCTCTCTCTCTTTTTCTGTCGCCCCGATCAAGTCTTAACGGCAAGTTGCAGCAACAAACATTTTCATTCGTTTTCGCATCGTCGAAGCGTACGGTTCATAGGAGGAACGGAACGAAGCGGGGAACGCGACGGAAACTAGTTGCTGTTTTTTTGTCCGTGTT

>ID_543 P_543 Promoter_547 D1_Promoter for RH37907, -250bp and +50bp [1 ]

AATAGTACGGTTAGAAGGTATTGCCACTAGCGGAGAGGCGACTGTTCTGATTTGCTTCATGTATCAATCAATTGACATAAGAATCTAATAATTTGTTATTTTTAAAATCTGCACATTTTTCAAATATGTTATAAGGTCGAAAGAAGGAACAACATTGGTGCAAGTGTGAAACCGTTTAAGCATAATTGATGCGGCCTTGTGTGAACAATGATGTGTGCTTAAATATACCAAGCAGGCTGGCAGCCACCCCCACACAGTATTTAACCACCCCCGAGGAACGGTCACATTGCTGCGAGTCTGAATATTTTTTTGCTTTGTGTCGCGTTTTAATGTGAAAAGAAAAACACTTT

>ID_546 P_546 Promoter_550 D1_Promoter for RH54244, -250bp and +50bp [5 ]

TGTTCGATTGATTTTATTCGAGCGAGCAAAAAAGTATTTTTAAAGCATTAACAAGTAAATTCCAGATCTTCCTTCCGGTTATGTTGCTAATTTTAAGTCCCAGCCCATCGCAATAACCATTAACTTGCAGCTGGGCACTTGGTATAACAGTTTATAACTCCACGGGATCGAATATCTGTGTACTGTGAGCTTAAAGCTCCGCTTGTTTGGGATCCAAGCCAGCATATTAAGCCCGATTCCAAACTAAGCTCTTGCAGTTAGCGCGCTCAACTCGAACTGAACGGAGGTCCAAAAAAAAAGAAACGAGCAATCCAAAAAAAATCGAATAATTGAAAAATCCAAAATGAACC

>ID_549 P_549 Promoter_553 D1_Promoter for RH22541, -250bp and +50bp []

TCACTCGGATTCCGAGTCCAATTCCGATTCCGAGTGGATGTTCATGGGAGCACGCCCACGTTGTTCGGGTCCACTCCCCCTTATTACATGCGCCCACTTTGGCTACTGGTACTATCTCTGCTGCTGCTGCTGCTGCTTCTGCCTCTGCCACCGGCGACCGGCGGCGTTGGCGTCGGCGTCGCCTTTCTTTGGCCGCTCCGCTCTCGTTTGTTTTTTTCGGCTCGGCTGGCCGCTCGCTTCCGAGTTGCCGCTCAGTCTGAAAAGTTCATGTGACGCGTCGAGAACGTTGCGATTCGAGTCCGCTGCTCCCGACTTCTGATTGCCGAGGCAACAGCGAGGGAATTCCGCAT

>ID_550 P_550 Promoter_554 D1_Promoter for RH57501, -250bp and +50bp [5 ]

TGATATTTACTTACAAAACCAAGTGAAATTTTTTTTTAATTATCATTTAATTTTAAAATACATTTTGGTGCATAACCAAAATTGAAACATGCCTATTCATTAAAGGGGAATGGAAATATCTTGCAAAAATGTACAATTAAATAAATACCAAAACCTTTGCCAGCTGGTCACCCCAAAACCAGCTGGTACGCAAAACTTCCGACAGGGGGCGCCACGCGCAGCGCAGCCAACTTCACGTCCAGCTGTCATTTCTTTCTCTTTTTCGCCTTCACCGACGAGTTGCGGTCGCTAGATTTGTAAATTTCTACAAAAAAGTGAGTAAACCAGCAGTGGCCGCCACTAAAACTGAA

>ID_552 P_552 Promoter_556 D1_Promoter for RH57685, -250bp and +50bp []

TTTTTTTTGTTAGCATCTTGTTGTGATCGCAACGCTGCTCCGCTGGCTTGTTTTTGCGGCGCATTTAATGAATGCGCCCCGTTCACCCCTCCCCTAATACTTCACACACACCACACACCGTACACCAAACGCCATACACCATACACCATACACCACACACCACCTCACCCCACCCACCAGTTTGCTGCGCAGTCGGCGTCGACGCAGCGGCATTGCGTTTAAAATGTCGCAGCGACGGCGCTGCGGAGCTCAGTCTAGCAGCAAAAGTCCAGCGATACGGTTCCAAAGCGATCTGATCTCAGTTTCGATTTGAATCCCCCGATCTGGAATTTCGAATTTTGGATTGTACT

>ID_553 P_553 Promoter_557 D1_Promoter for RE62710, -250bp and +50bp []

TTCGCTCCTTTTTACCGTATTCATTAATGACCTAGTTATTATAGTTATATCACCTTCTCGTTTACTTATGTATGCAGATGAGAGGAAAGGATATATATACATCATTGGATCGGAAACGCTTTCTTTTACAAACTAGTCAACAAATCCTGCATACTGTGTAACTGTAGAGTAGAGTCTTACGATATTCAAAAAGTAGGTTGGCAGCGCCACGCCACTGTTATTTGGTATAATCTAACGGCCCATTATGCTGCCACACTTTTATTTTGCCAGGAAAGGAAATCGGCAGTTAAAGATTACAATTGGAAAATTAAGTTAAATTAATGAGATATTTAAGTGCGCCTAAGTGAATA

>ID_555 P_555 Promoter_559 D1_Promoter for RH46822, -250bp and +50bp [1 ]

ATAAAATAAAAAAAAAAAAAAAGAAACCGCCGCGCAACTTAAATTGGCCATTAACGCAACTTCGACTTGCATCGTATCGAACCCGGCCGAGTGACGCAAAAATCAACTAAAAAAAAAGGTTAAGTATACGCCGTGCGGGCCGTGCCGCGACTGCGCTGCCAGCGTCGCCAGCGACGGCGGCGTCAAATGTTGGCCGGCCTGCGAAGCGCGTTCATTTTGTTTATTTATACAGCCGAGCGGGTAGAACTCCATATTAGTGTCTTCTTGGCGTTTGCCGCAGTCGAGTCCGAGTCCGAGTCCGAATCCGAATTCGAGTTTCGAGTCCGTAAACTGTAACTGAATCTTCAGGC

>ID_556 P_556 Promoter_560 D1_Promoter for GH16758, -250bp and +50bp []

AGAACAATAACAAGAAATATCCGACTGACCATTATTATTGAAATGCATTGTTAAATAGTGTGCAACAAATTACGCTCAAAATATAATATTCGTATCACATATGAATATCTCTATCTACTTGTATCTTCACGAGTCACGAATGAGTCACGACCTAGTCTAGATTAGTTAGTGCCTTCGCCAAAAGCACTCACGCTGCCATCGCTAGCGGGGAACTCACAGCTACGCGTTAGTCCCTTTCAAACAGCCGTGTGATTCACTTCGCTTGAAATAAAATAAACAAAAAGCAGAGAAAAGCTTCTGCAAATCAGTTGTTAACTTGCATTTAACAAACAAACCAATTCCGCTGACCA

>ID_557 P_557 Promoter_561 D1_Promoter for RE12939, -250bp and +50bp []

CAAAAAAAATTGTTTCTTATAACACAATATAGATGCAAACGTCTTTTCGCTTTTCACCAAATAGTGAGTGCACACTGAAGCACAAATTTGTGTAAAACCTTGTGAGAACATAAGCTATTAAATTTAAAAAATATAATTAATGGAAAAGCTTATGTGATTCGAGGACTTCAGTATTTTTTCAGAATGGCTTGCCCCGTCGCCTGGTCACACCGCTTGACTCAACCTGGAATATTCGACCACCCCGCGAACGGTCACTCTTACTCGGTCACAAAACAATGCAATATTTCGCGGAGCAGTGAATTAATCCGGAAATAATCGTCCGTGCCCAGAGCTTTGGAGGCCAAGTACCA

>ID_558 P_558 Promoter_562 D1_Promoter for RH31535, -250bp and +50bp []

CGCCAACGGCCACTCTATAATGCAGCCATCGTTTGTTTTTCAAAACGTATAGCTAAATTTCGGGTTAATAATATCTATCAAATGTTTATTTGAAAATTTAAAATTCTACAAGTAATTTTTTCCCCTATAAGCACTTATATATATATTTATTTTTATATTCATATTTTATTCCTTTGCAAATTTTTTCGACTTAAGATTTCCTCGAATTTCCCACTGCAGCTACAAAGTAGCTAAGTTGCCTACACAGCCACGTAGTTAGTCATGGGAGCTTTGTTTGGAAAAACCAGCAAAAAGACGGCTCCTAGTCGGATCACCGACCAGGACAAGGCGGTTCTGGTAAGTTGGTGGTA

>ID_559 P_559 Promoter_563 D1_Promoter for RH01239, -250bp and +50bp [8 ]

CTTTTACGAGAAGCAGCGTTGCCGAGAAGCCGGCGCACTTGACGCCGATTTCCTCGGGTTATAATTAGCCAGACTTATCAATATCAACGTAGAAAAGCAGATTTACATTGTGAACGGGTTTCCGCGCACTTGGCGTGCATCTATTCGCAATTAGCACCGAATTACAAACTGGTTAACAGCCGCTGCCAACGGAGTGGAGAAGCCTGCGGTCGCCCATTTGGGTTTATAAAATGGGTCGCCACTTGGCTGAGATCAGCAGTTTGGGTCACATCTCCAAGCGGTCAGCATGAAGTCGGTTGTAAGTCAAGATCTCAGTTCTCTTGAAGATTATCCCTGAGTTTTCCATACAA

>ID_560 P_560 Promoter_564 D1_Promoter for RE74682, -250bp and +50bp []

AAGTTAAAATGATTCTTGTCTAGAAAATTGATTATATTTTTCTGTTGTATTTTACAGTTTTGGAATTTCTAGATATGTTTTAATAAATAAGGGCAGTAAATTATTCCAACTTTATATTATTATTAACCATATCAATGAATGTTGAATTAAAAATTAATATGTTAGATTAAGTAAATTCCTATTTGCGTACTTGAAATCGGTCTTAGGCAACACTGAATTTCAGGTATTTAAGCCTCGAACGTGATTGGTCACTCTAATGCCCACGTGCGTTTTGTTTGGCCTCCGCCGGCGGTAAATTCAACCAAATTACCTGAAATTCCAAGGATTCCCCGGTAAACAAGAGATCAGCG

>ID_562 P_562 Promoter_566 D1_Promoter for RE09295, -250bp and +50bp [3 ]

ATCTAAGCAATGCTCTCAAATGTGCCATAAAAAGCCTGCCATACATACCGACATCTACAATTTTAGAGGTGATTTTAAAATGCACACACAATACAATTTGTCTGTTGCATAGTTTAACTGTCAAATGAGAAAATCTTTTAAATGCCAGTGGGAAAAACGACATCCTAGAAGTGACTGTGAGGCGTGACATCACCAGCTGTTATCGTTATCGATAGGCGACGTGTGCACACTACTTGCCATCCATGTGGCATCGTTGTGTTGTTGTTATTAATCAGCGGAGCAACAGAAGTCACAATTTGGAAACATGTCCGAGCTAAATATGGAGCTGAAAAAACTCAGGGAGCTGGTAA

>ID_563 P_563 Promoter_567 D1_Promoter for RE28833, -250bp and +50bp [1 ]

AGTGCGAGCGAGACAGCATCCAAACAAAAGGCATAACAGCCCTGTCTTTCACTTCGCTCTCTTTGGCTCTCTGGCTCTCCCGCCGAGAGTGTCAAATTCAAGGTCGCCCCCAGTGCTATACTACAAAGCGTATAGCTGAATAAAACCTCCTCTCACACCATTCGAGCGAAAGAGCGCATATTCAGCGTGTGAGCAGGAGAACGATACGGTGAGAGAGCGGCTTGGAAAGAGCCCCATGTTTTCAGTTCCTCAGTTTGTCAGCGTATGCCCTACCATGCGGATGTGCTCGCGGATTGATTTCGACTCGAACTCCGGCTCCGTTTTCGTCTTTTAACGTAAGTCCCGTGCGA

>ID_565 P_565 Promoter_569 D1_Promoter for RH20252, -250bp and +50bp [1 ]

TTTAAATGATTCGAAAGCGAATTTAGATCATGCTTTGAAAGTTCGTTACGATTGCTATGAATTGAGTTTAAATATTTCAAGGCTACATAAATTAGTAATTAACTCAATAGCCACCACTGATTTGGCTTACAAACTCTTTTGTTTAATATTATTTTTAAAAGCTTTAAATATTTTAAATAGATTTAGCTAGAAAATAGCTGACAGACACATATCGATATATCGCTGCGATAGCCACAGCTGTTCACGCCCGCAGTTTAAGCGATGTGGCAGCCCTGGTCGGCCACCAAAAAATAAACATTGGAATTTTCCCTTAACTTTATAGAGTTTTATCCAATTTGAGCGATGCTGCC

>ID_567 P_567 Promoter_571 D1_Promoter for RE64996, -250bp and +50bp []

CAGGCGGAAATACTTGCCTCGGCTTCAATGGAGTTGATCAGCGCCTCGGCGCGCTCGTTGTTGCTTGCAATCTGCTGCAGATCGGCCATTGTTCTTTTGTTTCTCGCGAATTTTGAGCAATTCTGGCCGTAATTTTTACATGCGGATCCCACAGTCGTGGCAGCTCTACGCAGCATTGCAGTGCTTCTTCTTGCCACGCACAGTGGCGTCGCGCAGCGTTGGACAGGTAGTTCGTTGGCGGAGAAGCGAGAGTATTTTTACATCAGCCCTGCCAGGGTCACTCTAATCGGTGCAACGCAAAAACGGCTGGCAGTTTTGAGCGAAATTTGTTAAAAAATAAACAATAAAAA

>ID_568 P_568 Promoter_572 D1_Promoter for RE15245, -250bp and +50bp []

TCAAAGTTTCCTAATAGATTGGCGTTGCGCTTGAGGAAATTGTCATCGGGTCGCAAAGTAATTCCAGCTCAGGCCGATAGCTACGCTGCTCTTTCACTCTCCTGGATTTTCTCTCTCCACCCTCCCCACTCCATCCTCCGCCAATCTCACTCTACATTCATATGCATCGCCCTCTCTTTTGCTCCGGCGTAAAAAGCTTCGATCGGTTCGTGCTTGGGGCGTTTCGTTTGGGGCTTGTGATTTCAGTTTCAGTTTTGAAGATCACTCGTTGCGAAGCGGACGCTTAGCACAGCGGCAAGTGAAAGCTGGAAGATATAGTTGGCAAAAATAACAAATCGATCTTCACTTCC

>ID_569 P_569 Promoter_573 D1_Promoter for RE42883, -250bp and +50bp []

ATCAATCTGCAGTTGATAAAAAAAACTCAACGAGGGAGATAGAGCGATAGAGCGGTGAAGTAAGCCCGAGATAAACCTGAGAGCGGAACTCTCGCCTACGCGATCGTGCTGACCGCCAAATGCAATATCCGCCGCTCCACCTGGTGGCGCTCTCAATTCTCAATTCTGCGGTCTTCTTGCTCTCGCCAAATCTGCCCCAATGTTCTCTCAGCGAGTCGATTTGCGATGGCAATGCCAGAGGCTCGAAACAGTTCCGTCGCAGGTTTCCAACGGTACAGAGGTGCAATCGCTTAATTCTCGCAAAAAACAATCAGTACCCAATTATAAAATAGTTGCTAACAAGGTGAGTT

>ID_570 P_570 Promoter_574 D1_Promoter for RH39375, -250bp and +50bp []

ATCTTTTATTTATACTCGTTGGAAAAATTGTGAATAGCAAAAAACAAAATTCAGAGAAAATAAAGGCGAATGAAATTTTATATCAAGAAATTTTAATTAAATGATTATTAATTTACAAATGATTAAATGAACAATTTTCGATATCAAAGCGTTTTTATTTATAAAAGATATTATATGGAAGGGTATTTAATAGTTATATATATTTCTTAGCCCTTTTTTTTGGTATTTTTTGTGTGGTCGCCGTGCGGTCATACTGACCCAATCAGGTGAAAGCGACTTCGGAATTATCGCGCTACAGGGCGATAAGACTGTGACGACTGATTAGTTAACCAGCGGCGATCGGCAAGGTG

>ID_571 P_571 Promoter_575 D1_Promoter for RH12289, -250bp and +50bp [4 ]

CTCACGGAACCCAAGCAGATTTTGTATGGAATTAATCAGGAAACCCACTCAACTTGTCATTTGCGGATCTAGTTTTGATCGCAGCGCCTCGGGAAAAAGCCCAAAATTAAGCGGATACTACCAGCTATAGCCTCGATCAGTCTCTGAACCGATCCGAACAAAGGTATTCAAGTTATAGGCAAAAATGTCTGCAACCGATTTGGCGCAAGAAGAAGCAAGCGGCAATTTCTAGCCATCGAACGGACGGCAAGCTCAGTCTTGTACGAACCGTCGACGGGAGCACATATCGGTGGTTAGCAAACCAAAGGAAAACGGATATTCCATACATCAGTGGGGCCTACGAGTAAATC

>ID_572 P_572 Promoter_576 D1_Promoter for RH53063, -250bp and +50bp []

TATCAAATTAATAATGCAATTAATAATTATTATATTGTATATAATAAAATTGTAAATAAATATCGTACTCATGCTATTATTGAACCTGGCTATCTATAAGCGTCACGAAAAGCACACAGGCGCACTGTGGCGTTTTAGCTTTTTTAAAGCTTTCTTCGCCCTGCGTAAGACAGCTTTTTTTCTGTACAGCTAGAAACAGCTTTTTTGCTGCCAGCAAACTGGCAACACTGGCGGCAGGCGTCACTACTCTCATTGTTATTGTCATCACTATTGCATTGACATTGTTGCTGTACGTCGTGTTTTAATTAATTTCCATTGCGCTTTTTTGTCGAATTATGAAAATTTACAAG

>ID_573 P_573 Promoter_577 D1_Promoter for RH70420, -250bp and +50bp [6 ]

ATGTCAACCGGTGTGGCTAATTGTCGCGCACTTGAATTGTCGTCAATGAATTGTGGTTATTGATTCGGGAAGGTCAAGGGGTTCGGGTTGGGGTTGGGGTTGGGACTGAAGGTATTACGTGGTTTGTTCTCTGGCCCGGTTTCCGCATTTGTGCAGCAAACATACACATGGCTGATCAATCCGAGAACTGGCCTTATATGCTAAATGAAATTATGGATCGGAGCTATAAAAGCACTTGGCCAACGGGCAGATGTCAGTTAGTTTCGTTTCAAGCGTCACTGAGATCACAAAAGCCAACATGAAGGTGAGATTGAAGGGATATCGGATAACTGGAGCTTAGTTACAGCTAG

>ID_574 P_574 Promoter_578 D1_Promoter for RE20343, -250bp and +50bp []
[truncated: 1,753,820 more chars]
